# *GmFT2a* Polymorphism and Maturity Diversity in Soybeans

**DOI:** 10.1371/journal.pone.0077474

**Published:** 2013-10-14

**Authors:** Bingjun Jiang, Yanlei Yue, Youfei Gao, Liming Ma, Shi Sun, Cunxiang Wu, Wensheng Hou, Hon-Ming Lam, Tianfu Han

**Affiliations:** 1 MOA Key Laboratory of Soybean Biology (Beijing), Institute of Crop Sciences, The Chinese Academy of Agricultural Sciences, Beijing, China; 2 Mudanjiang Branch of Heilongjiang Academy of Agricultural Sciences, Mudanjiang, Heilongjiang, China; 3 Center for Soybean Research, State Key Laboratory of Agrobiotechnology and School of Life Sciences, The Chinese University of Hong Kong, Shatin, New Territories, Hong Kong; USDA-ARS-SRRC, United States of America

## Abstract

**Background:**

Soybean is a short-day crop of agricultural, ecological, and economic importance. The sensitive photoperiod responses significantly limit its breeding and adaptation. *GmFT2a*, a putative florigen gene with different transcription profiles in two cultivars (late-maturing Zigongdongdou and early-maturing Heihe 27) with different maturity profiles, is key to flowering and maturation. However, up to now, its role in the diverse patterns of maturation in soybeans has been poorly understood.

**Methods:**

Eighty varieties, including 19 wild accessions, covering 11 of all 13 maturity groups, were collected. They were planted in pots and maintained under different photoperiodicity conditions (SD, short day; LD, long day; and ND, natural day). The day to first flowering was recorded and the sensitivity to photoperiod was investigated. Polymorphisms in the *GmFT2a* coding sequence were explored by searching the known SNP database (NCBI dbSNP). The *GmFT2a* promoter regions were then cloned from these varieties and sequenced. Further polymorphism and association analyses were conducted.

**Results:**

These varieties varied greatly in time to first flowering under ND and exhibited a consecutive distribution of photoperiod sensitivity, which suggested that there is rich diversity in flowering time. Furthermore, although *GmFT2a* had only one known synonymous SNP in the coding sequence, there were 17 haplotypes of the *GmFT2a* promoter region, HT06 of which was extremely abundant. Further association analysis found some SNPs that might be associated with day to first flowering and photoperiod sensitivity.

**Conclusion:**

Although *GmFT2a* is a key flowering gene, *GmFT2a* polymorphism does not appear to be responsible for maturity diversity in soybean.

## Introduction

Different soybean cultivars exhibit different maturity pattern and sensitivity toward photoperiod, which are related to their adaptation to different ecological environments. For practical reasons, soybean breeders categorized soybean cultivars into different "maturity groups". For instance, soybeans in North America were classified into 13 maturity groups (MG): MG000 to MGX [1,2]. On the other hand, Chinese soybean researchers have divided cultivars into 12 MGs based on the environments and planting patterns in China [3,4]. Commercial cultivars of desirable traits but belonging to a particular MG are often limited by the geographical range of cultivation. It is therefore important to gain a better understand on the genetic control of photoperiodism and maturity in soybean.

Photoperiod responses and maturity patterns in soybean are quantitative traits controlled by multiple genes or loci. Up till now, nine maturity loci have been reported, including, E1-E8, and J [5-13]. These loci have been comprehensively reviewed by Xia et al. [14]. They play different roles under different photoperiods with stronger effects under long-day and weaker effects under short-day conditions [15]. Four of these loci were characterized at the molecular level, using map-based or candidate-based cloning. *E1* encodes a soybean-specific potential transcription factor Glyma06g23040 [16]; *E2* encodes a GIGANTEA homologue, GmGIa [17]; and *E3* and *E4* encode the phytochromes GmPhyA3 [18] and GmPhyA2 [19]. However, the genes corresponding to the five remaining loci have not been identified and the exact functions of the four identified loci remain unclear. 

The key flowering gene *Flowering Locus T* (*FT*) in *Arabidopsis thaliana* encodes a putative florigen that is an integrating factor of the flowering regulation network [20,21]. Soybean has at least 10 *FT*-like genes [22,23], among which, *GmFT2a* and *GmFT5a* could functionally promote flowering in *A. thaliana* [23,24]. Furthermore, it was observed that *GmFT2a* overexpression could induce early flowering in transgenic soybean [24]. Therefore, it is surprising that these two important flowering genes have not been considered as candidate genes for the five unidentified maturity loci. *GmFT2a* is regulated by photoperiod differentially in two cultivars exhibiting different photoperiod sensitivities (photoperiod-sensitive Zigongdongdou and photoperiod-insensitive Heihe 27) [24], suggesting that the function of *GmFT2a* might be related to the regulation of maturity. While it is speculated that the expression of *GmFT2a* may be developmentally regulated [24], the promoter region of *GmFT2a* has not been thoroughly analyzed.

The release of the soybean reference genome [25] has provided a new platform for breeding and molecular research. In addition, the resequencing of 31 wild and cultivated soybean genomes (designated as 31-Soybean Resequencing Project in this paper) has further characterized genome-wide genetic variations [26]. These studies may provide tools to address the question of how the polymorphisms in *GmFT2a* and its flanking sequences might function in the diversification of flowering and maturity time in soybeans.

In this study, soybean cultivars were originally cultivated/collected in China and North America, which cover most MGs, from MG000 to MGVIII. The sequence polymorphisms in the *GmFT2a* coding sequence and the *GmFT2a* promoter were analyzed. Possible roles of *GmFT2a* and its potential application in breeding were discussed.

## Materials and Methods

### 1: Plant materials and photoperiod treatments

Eighty varieties were collected in China and North America (Table S1 in File S1). Soybean seeds were planted in soil in 10-liter pots and grown under natural day (ND) conditions. After germination, seedlings of uniform size were selected so that each pot finally contained five uniform plants. The seedlings were grown in nature sunshine until the cotyledons opened, and were then separated into groups and grown under different photoperiods (LD, 16 h light/8 h dark; SD 12 h light/12 h dark; and ND). Additional details regarding plant growth and treatments were as reported before [27]. The day to first flowering of each plant was recorded as the number of days from the expansion of unifoliates to first flowering (DEUFF) and 15 plants in three pots were investigated for each variety of each treatment. Photoperiod sensitivity (PS) was calculated as described previously [28]. 

### 2: DNA Extraction, PCR, and Sequencing

Genomic DNA was isolated using the TianGen (Beijing, China) New Plant Genomic DNA Isolation Kit (DP320). Two PCR primers *GmFT2a-5-N2300* (5’-AAGTAAATTATTTTCCCCTTATTTCCTATC-3’) and *GmFT2a-3-P165* (5’-CAAAGTATAGAAGTTCCTGAGGTCATCA-3’) were used to amplify the *GmFT2a* promoter region. The resulting PCR product was cloned into the pMD18-T simple vector (Takara, Dalian, China) or the pZeroBack/blunt vector (TianGen, Beijing, China). Further Sanger Sequencing was done in the National Key Facility for Crop Gene Resources, Institute of Crop Science, The Chinese Academy of Agricultural Sciences, China. In addition to the vector-specific sequencing primers, the primers GmFT2a-5-N1655 (5’-ACAGTGCATGTGGGAGGCAAATCGGCATAT-3’) and GmFT2a-3-N350 (5’-CACATCCCTTCCATCTTCTCATTTTCTC-3’) were used in DNA sequencing. All new sequencing data (List S1 in File S1) has been deposited in GenBank.

### 3: Bioinformatics analysis

The genomic sequences were aligned using ClustalW 2.0.9 [29]. The alignment was adjusted manually and input into MEGA 5 [30] for calculation of nucleotide diversity and Tajima’s D statistics. It was also input into TASSEL [31] to estimate linkage disequilibrium and identify SNP-trait associations by generating a general linear model (GLM). The phylogenetic relationships among the 17 haplotypes were inferred using the NJ method in MEGA 5 [30].

## Results

### The selected soybean population exhibited a continuous spectrum of photoperiod sensitivities

**Figure 1:**
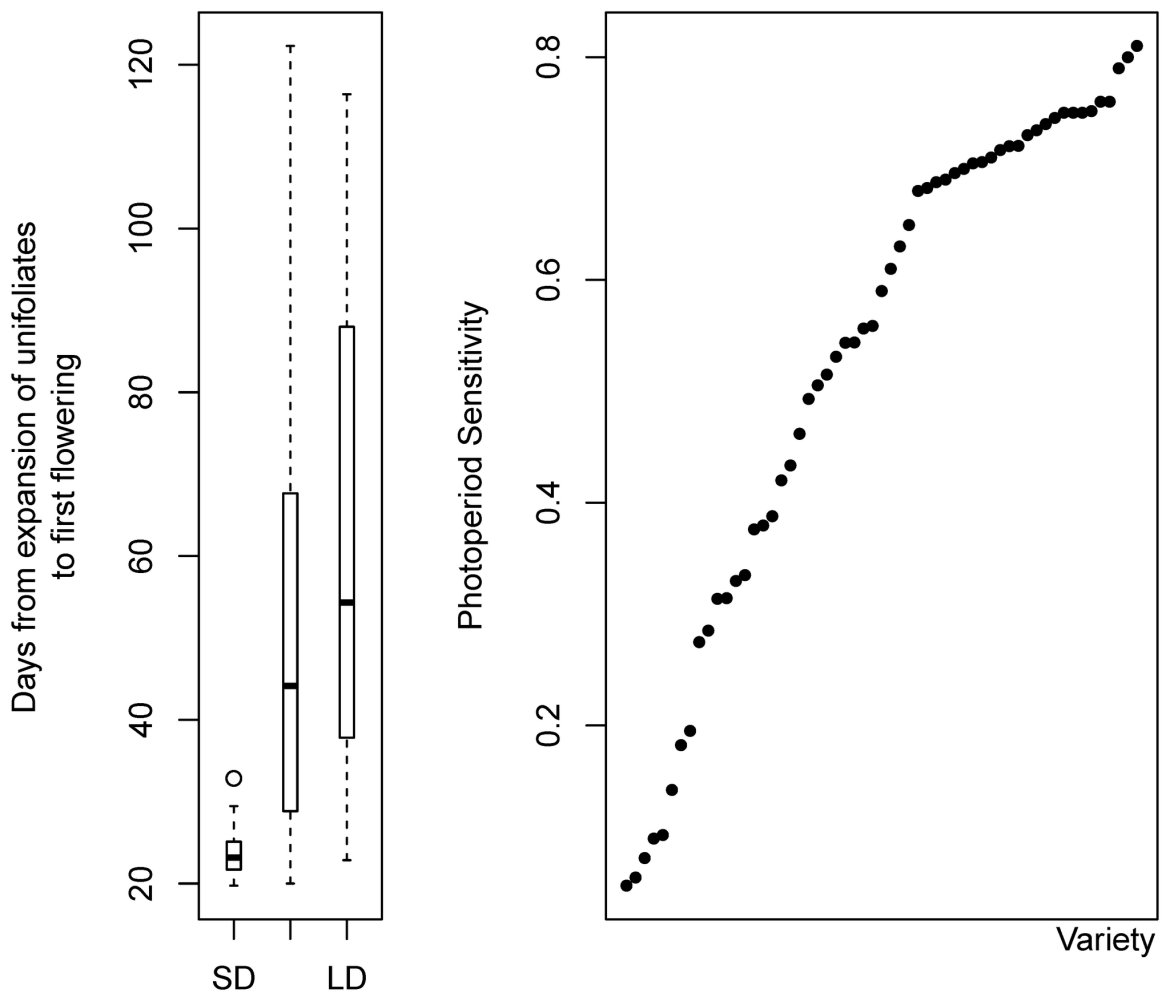
Days to first flowering under different photoperiod conditions and photoperiod sensitivities of different soybean varieties. Left, the number of days from expansion of unifoliates to first flowering (DEUFF) under different photoperiod conditions (SD, short day; ND, natural day; and LD, long day). Right, sorted photoperiod sensitivities of different soybean varieties.

The plants investigated in this study were collected/cultivated in China and North America. The selected cultivars include a wide range of maturity types, from MG000 to MGVIII, and comprising 11 of the 13 defined maturity groups [1], representing a diverse population adapted to different geographic regions. As shown in Table 1 and Figure 1, the DEUFF varied from 19.7 to 32.8 days under SD conditions, from 20.0 to 122.3 days under ND conditions, and from 22.9 to 116.4 days under LD conditions, except two accessions, CS36 and ZG, which totally failed to flower under LD conditions. The variation of DEUFF was reduced under SD conditions and enhanced under LD conditions (Figure 1), which was not surprising for the SD crop soybean. The population also exhibited a rich diversity of PS, increasing from 0.056 (CS02) to 0.81 (H05) (Figure 1).

**Table 1:** Days from expansion of unifoliates to first flowering (DEUFF) and photoperiod sensitivity (PS).

**Cultivar**	**DEUFF**			**PS**	**Cultivar**	**DEUFF**			**PS**
	**SD**	**ND**	**LD**			**SD**	**ND**	**LD**	
CS01^000^	21.4±1.1	20.4±0.7	22.9±2.4	0.063	CS38^VII^	24.9±0.4	73.9±2.2	88.9±1.9	0.720
CS02^000^	22.6±1.1	20.9±1.4	23.9±2.2	0.056	CS39^VII^	24.9±0.6	84.6±1.1	89.0±1.7	0.720
CS03^00^	21.4±1.5	24.7±1.7	26.2±3.3	0.182	CS40^VIII^	26.9±1.3	83.2±1.1	108.2±8.9	0.752
CS06^00^	21.4±2.0	20.0±0.6	23.2±0.9	0.081	CS41^VIII^	26.7±2.4	83.9±1.4	90.6±0.5	0.706
CS07^0^	21.7±2.1	22.0±2.5	27.0±5.4	0.195	CS43^VIII^	27.6±0.7	73.9±3.1	88.4±0.5	0.688
CS08^0^	20.6±1.3	20.1±0.6	22.9±1.7	0.098	CS47	23.1±0.9; 0.899735; 0.899735; 0.899735;	30.2±1.4	37. 1±5.0	0.376
CS09^0^	24.1±3.2	25.5±0.9	28.1±4.1	0.142	CS48	23.1±1.7	28.9±2.2; 2.21154	37.8±6.5	0.388
CS10	22.6±2.7	29.8±1.9	32.9±3.4	0.314	CS52	22.9±0.8	31.9±1.9	47.2±3.8	0.515
CS12^I^	22.8±1.7	28.5±2.1	33.2±1.6	0.314	CS54	24.1±1.1	44.1±1.8	54.3±3.0	0.556
CS13^I^	24.8±1.6	32.5±1.5	42.7±3.4	0.420	CS58	27.0±0.8	45.0±2.7	50.2±1.9	0.462
CS14^I^	25.1±3.0	27.9±2.3	34.6±3.1	0.275	CS59	28.0±1.7	49.5±1.4	61.3±3.2	0.543
CS15^II^	22.2±2.3	28.6±2.1	33.1±2.5	0.330	H01	20.7±0.7	48.3±2.0	82.6±0.9	0.750
CS16^II^	24.8±1.9	28.7±2.6	34.7±4.7	0.285	H02	23.5±0.5	71.1±2.1	80.1±2.1	0.710
CS19^II^	26.7±2.1	31.2±2.6	40.1±3.7	0.335	H03	21.2±1.1	29.5±1.0	82.1±0.8	0.740
CS20^III^	22.0±1.9	31.2±3.2	46.9±5.2	0.531	H04	25.5±1.2	71.4±1.6	94.6±1.0	0.730
CS21^III^	26.2±2.1	35.6±6.0	46.3±0.5	0.434	H05	22.4±1.0	75.9±1.6	116.4±1.2	0.810
CS22^III^	25.1±0.4	31.5±0.8	40.5±1.6	0.380	H06	19.9±0.8	28.8±1.1	54.2±2.3	0.630
CS23^III^	25.3±2.1	39.4±1.0	49.9±3.1	0.493	H07	22.6±0.6	78.6±0.7	115.1±1.3	0.800
CS24^IV^	21.3±1.6	34.1±6.0	46.7±4.2	0.544	H08	21.7±0.6	28.7±1.0	56.1±3.7	0.610
CS25^IV^	23.7±2.7	36.7±3.1	47.9±3.6	0.505	H09	22.1±0.7	58.9±0.9	72.0±5.3	0.690
CS26^IV^	23.2±2.2	45.2±2.5	52.5±5.0	0.559	H10	21.3±0.6	25.5±1.1	51.5±6.1	0.590
CS29^V^	25.0±0.8	60.2±1.2	83.3±0.6	0.700	H11	19.9±0.8	49.2±6.2	61.5±4.6	0.680
CS30^V^	27.9±1.4	67.1±1.4	87.9±1.1	0.683	H12	21.8±0.7	63.1±1.5	88.0±0.0	0.750
CS31^V^	26.4±1.3	67.2±0.8	86.9±0.8	0.696	H13	19.7±0.7	37.0±0.8	81.4±2.6	0.760
CS32^VI^	29.5±1.0	67.0±0.5	84.0±0.0	0.649	H14	20.1±0.8	58.5±1.1	79.0±0.7	0.750
CS33^VI^	26.3±2.0	68.1±1.3	89.0±2.3	0.705	H15	21.3±0.6	58.0±0.7	88.8±1.3	0.760
CS34^VI^	24.1±0.8	82.1±3.3	94.7±0.5	0.745	H16	22.5±0.7	64.5±1.2	106.3±1.1	0.790
CS35^VI^	25.0±0.8	83.8±1.4	94.1±0.6	0.734	HH	21.2±1.7	22.8±0.9	23.6±1.3	0.102
CS36^VII^	24.7±0.8	83.6±0.5	NaN	NaN	ZG	32.8±0.8	122.3±0.8	NaN	NaN
CS37^VII^	25.1±0.7	72.6±1.3	88.6±1.1	0.717					

The superscript indicates maturity group. SD, short day; ND, natural day; and LD, long day.

### The *GmFT2a* coding sequence is highly conserved

Investigation of the SNP data from the 31-Soybean Resequencing Project [26] revealed that there was only one SNP site in the *GmFT2a* coding sequence. It is a synonymous A/T SNP (named ss249156869) located at position 30746204 on chromosome Gm16 (Figure S1 in File S1). Further resequencing of over-100 cultivated soybean genomes did not identify any SNP in the *GmFT2a* coding sequence (unpublished data). Therefore, the *GmFT2a* coding sequence is highly conserved and the diversity in flowering time and maturation time in soybeans is not a result of polymorphism in the coding region of *GmFT2a*.

### The *GmFT2a* promoter region harbors rich polymorphisms

The *GmFT2a* promoter (about 2.3Kb) from each of the 80 soybean accessions used in this study was cloned and Sanger sequenced (available in GenBank with accession numbers of KF573201 - KF573362). Considering that the soybean genome is palaeopolyploid and contains 10 *FT*-like genes [23,32], we confirmed each sequencing result with BLAST, with reference to the genome of Williams 82 (www.phytozome.org). The nucleotide diversity was analyzed by Tassel v2.1 [31]. In total, 15 SNPs and 16 InDels were detected in the 2,489 aligned base pairs, with six InDels and two SNPs contained within two longer InDels (Table S2 in File S1). For the whole sequenced population, an average difference of 4.7 SNPs per kilobase (π=0.0047) were found between two samples (Table 2). For the subpopulations of North American cultivars, Chinese cultivars, and wild soybeans from China, the values were 4.4, 4.8, and 5.4 SNPs per kilobase, respectively (Table 2). This showed that the *GmFT2a* promoter region is more diverse in the soybeans from China than those from North America. Indeed, the Watterson estimator (θ) value was higher in the North American subpopulation than in the other two subpopulations (Table 2). Tajima’s *D* values were all negative, with differences reaching a significant level (P<0.001) in the population and subpopulations (Table 2). Without considering the two SNP sites located inside InDels, the 13 independent SNP sites were compared with those found in the 31-Soybean Resequencing Project [26]. Ten sites were common, three sites were newly found, and seven sites were missing. The missing sites were either close to an insertion and a deletion (Table 3). 

**Table 2:** Summary of DNA polymorphic sites in the *GmFT2a* promoter region.

	Whole	Cultivated		Wild
		*N. America*	*China*	
π	0.0047	0.00441	0.0048	0.0054
θ	0.04551	0.03129	0.01529	0.01735
Tajima's *D*	−2.91836	−2.93295	−2.60104	−2.52622

π, average nucleotide differences per site between the two sequences; θ, Watterson estimator; Tajima’s *D*, test for neutral selection (significant at P<0.001).

**Table 3:** Comparison of SNP sites from the 31-Soybean Resequencing Project and those seen in the *GmFT2a* promoter sequences from the present study.

**Position**	**31-Soybean Resequencing**	***GmFT2a* promoter sequence**
18	30739527	S17
163	30739672	S162
225	30739733	
321	30739826	S320
456	30739961	S455
676	30740180	
1150	30740650	S1149
1459	30740952	S1458
1494	30740984	
1581	30741026	S1580
1737	30741179	
1740	30741180	
1743	30741183	
1744	30741184	
1845	30741283	S1844
1912		S1912
1931	30741349	S1930
1945	30741363	S1944
2033		S2032
2229		S2228

### *GmFT2a* promoter region exhibits 17 haplotypes

**Figure 2:**
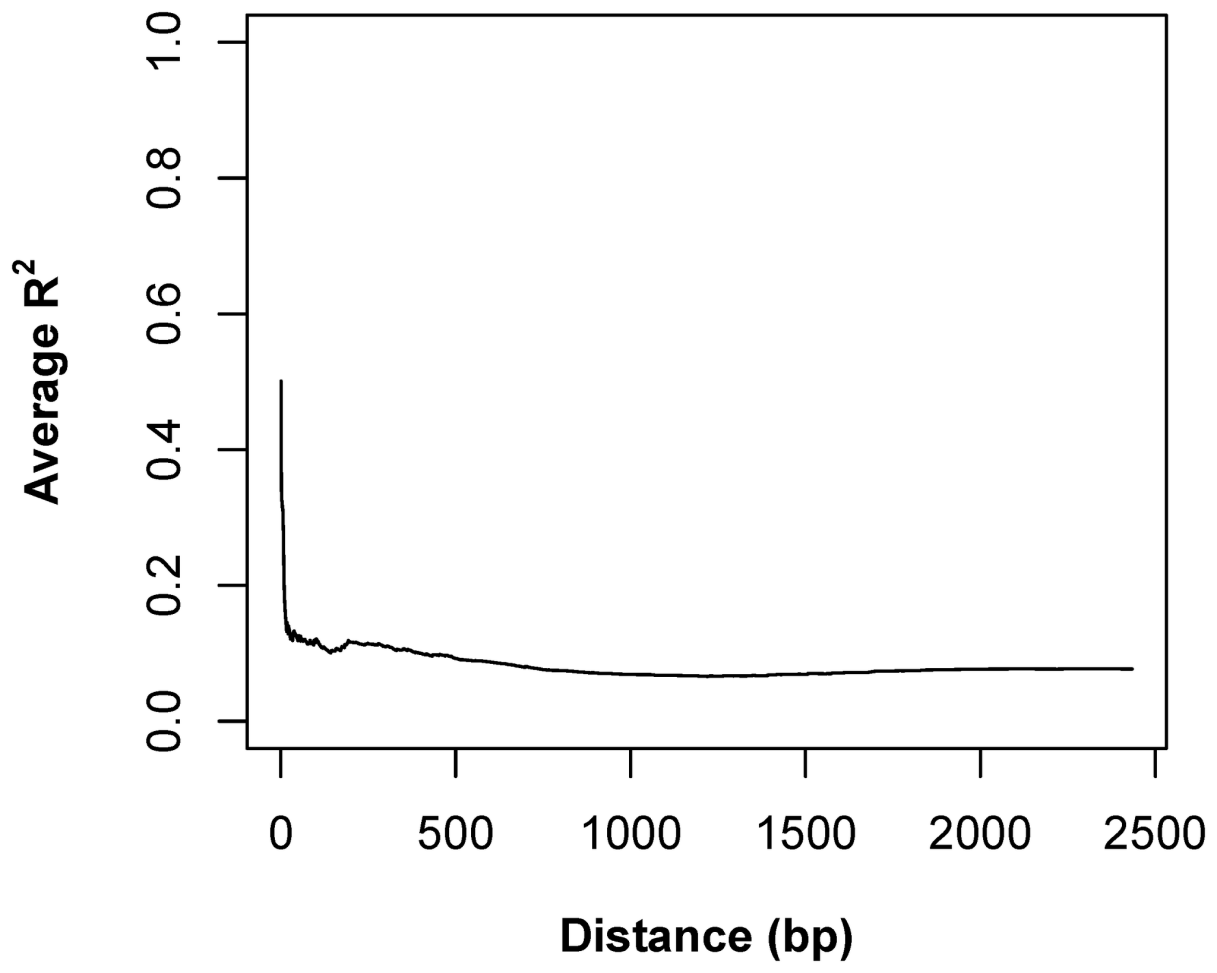
Linkage disequilibrium decay: *GmFT2a* promoter region.

**Figure 3:**
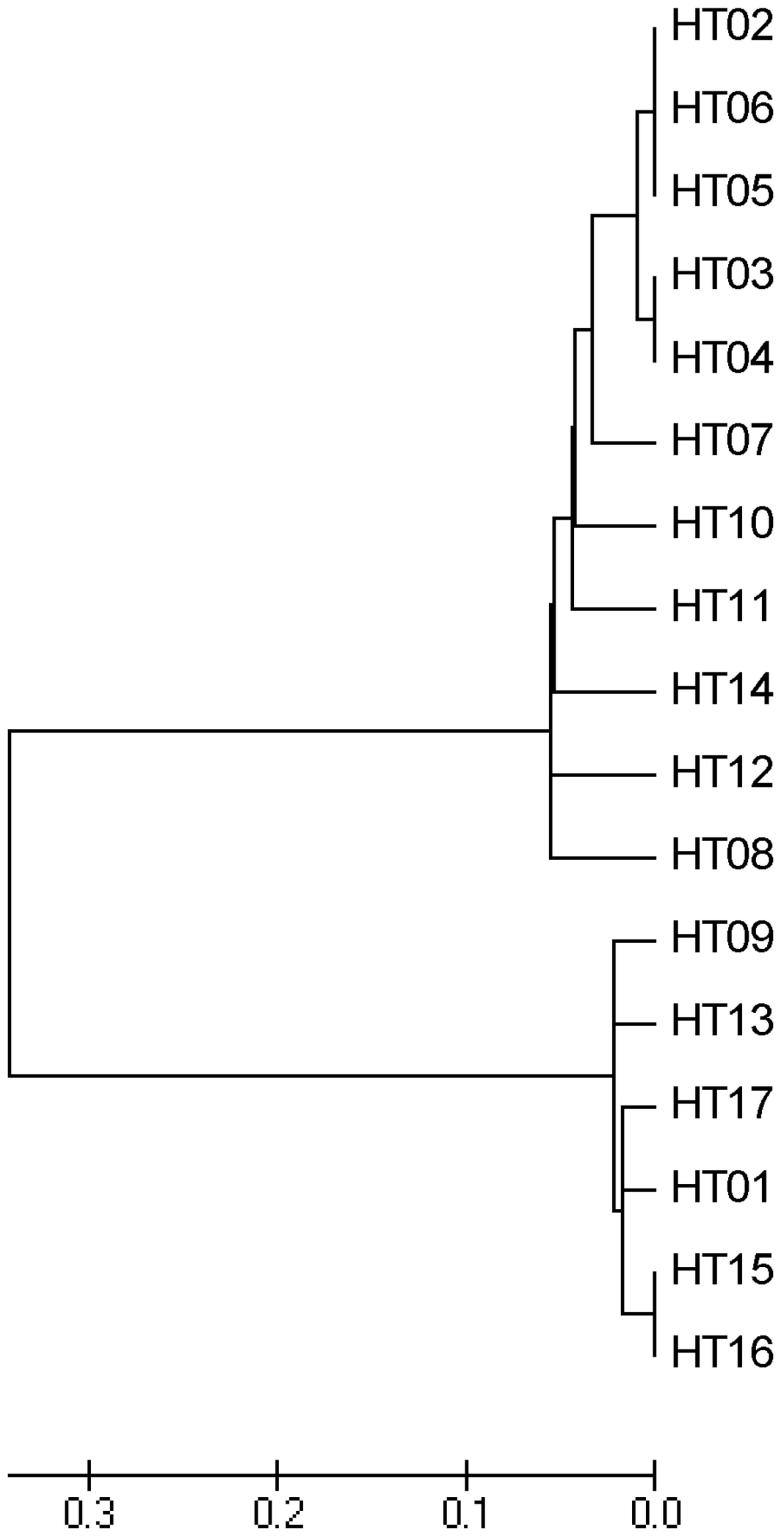
Neighbor-Joining tree depicting the phylogenetic relationships between 17 haplotypes of the *GmFT2a* promoter region.

Although there were many polymorphisms in the *GmFT2a* promoter region, no linkage disequilibrium was detected in this region (Figure 2 and Figure S2 in File S1). A total of 17 haplotypes (HT01-HT17), with ten SNP sites and six InDels, were found in these 80 accessions (Table 4). HT06 was the major haplotype, accounting for 62 accessions; more than two-thirds of the population. In the wild soybeans (H01-H16, J1-J3), 14 haplotypes were included; that is, HT01, HT02, HT04, HT05, HT06, HT07, HT08, HT09, HT10, HT11, HT12, HT13, HT14, and HT16 (Table 5). The cultivated soybeans included six haplotypes, HT02, HT03, HT04, HT06, HT15, and HT17, of which haplotypes HT03, HT15, and HT17 were not found in the wild accessions (Table 5). These haplotypes were further analyzed phylogenetically. An NJ tree showed the division of the haplotypes into two major clusters. One cluster contained 11 haplotypes; that is, HT02, HT03, HT04, HT05, HT06, HT07, HT08, HT10, HT11, HT12, and HT14; the other cluster included six haplotypes, HT01, HT09, HT13, HT15, HT16, and HT17 (Figure 3).

**Table 4:** Haplotypes of the *GmFT2a* promoter region in 80 varieties.

**Position**		S17	S162	D231	S320	S1149	S1458	D1496	S1580	D1737	S1844	S1849	D1849	S1912	S1930	D2014	D2263	**Variety Number**
**Haplotype**	HT01	A	A	4	A	T	T	1	A	0	G	-	20	G	G	3	0	***1***
	HT02	A	C	0	C	C	A	44	T	2	G	-	20	A	A	3	0	***1***
	HT03	A	C	0	C	T	A	44	-	2	G	-	20	A	A	3	0	***1***
	HT04	A	C	0	C	T	A	44	G	2	G	-	20	A	A	3	0	***2***
	HT05	A	C	0	C	T	A	44	T	0	G	-	20	A	A	3	0	***1***
	HT06	A	C	0	C	T	A	44	T	2	G	-	20	A	A	3	0	***62***
	HT07	A	C	4	C	C	A	44	T	0	G	-	20	G	G	3	0	***1***
	HT08	A	C	4	C	C	T	1	A	0	A	C	0	G	G	3	0	***2***
	HT09	A	C	4	C	C	T	1	A	0	G	-	20	G	G	4	0	***1***
	HT10	A	C	4	C	T	A	44	G	0	G	-	20	G	G	3	0	***1***
	HT11	A	C	4	C	T	A	1	A	0	G	-	20	G	G	3	0	***1***
	HT12	A	C	4	C	T	T	1	A	0	A	-	20	G	G	3	0	***1***
	HT13	A	C	4	C	T	T	1	A	0	G	-	20	G	G	0	0	***1***
	HT14	A	C	4	C	T	T	1	T	0	A	-	20	G	G	3	0	***1***
	HT15	G	T	4	A	T	T	1	A	0	G	-	20	G	G	4	10	***5***
	HT16	G	T	4	A	T	T	1	A	0	G	-	20	G	G	4	0	***1***
	HT17	G	T	4	C	T	T	1	A	0	A	T	0	G	G	3	0	***4***

**Table 5:** Haplotypes of the *GmFT2a* promoter region in 80 soybean varieties.

**Variety**		**Haplotype**	**Variety**		**Haplotype**	**Variety**		**Haplotype**
**N. American Cultivars**	CS01^000^	HT06	**N. American Cultivars**	CS29^V^	HT06	**Chinese Cultivars**	CS59	HT15
	CS02^000^	HT06		CS30^V^	HT06		CS60	HT17
	CS03^00^	HT06		CS31^V^	HT02, HT06		CS61	HT06
	CS04^00^	HT06		CS32^VI^	HT06		CS62	HT06
	CS05^00^	HT06		CS33^VI^	HT04, HT06		CS63	HT06
	CS06^00^	HT06		CS34^VI^	HT15		HH	HT06
	CS07^0^	HT06		CS35^VI^	HT06		ZG	HT17
	CS08^0^	HT06		CS36^VII^	HT06	**Wild Soybeans**	H01	HT05, HT06
	CS09^0^	HT06		CS37^VII^	HT06		H02	HT01
	CS10	HT06		CS38^VII^	HT06		H03	HT06
	CS12^I^	HT06		CS39^VII^	HT06		H04	HT06
	CS13^I^	HT06		CS40^VIII^	HT06		H05	HT06
	CS14^I^	HT06		CS41^VIII^	HT15		H06	HT06
	CS15^II^	HT06		CS42^VIII^	HT15		H07	HT10, HT11
	CS16^II^	HT06		CS43^VIII^	HT06		H08	HT13
	CS17^II^	HT06		JU	HT17		H09	HT07
	CS18^II^	HT06		WM82	HT06		H10	HT09
	CS19^II^	HT06	**Chinese Cultivars**	CS46	HT06		H11	HT06
	CS20^III^	HT06		CS47	HT06		H12	HT08
	CS21^III^	HT17		CS48	HT06		H13	HT06
	CS22^III^	HT06		CS49	HT06		H14	HT04, HT06
	CS23^III^	HT06		CS50	HT06		H15	HT06
	CS24^IV^	HT06		CS51	HT15		H16	HT08
	CS25^IV^	HT03, HT06		CS52	HT06		J1	HT12, HT14
	CS26IV	HT06		CS53	HT06		J2	HT16
	CS27	HT06		CS54	HT06		J3	HT06
	CS28^V^	HT06		CS58	HT06			

Superscript indicates maturity group.

### Several SNPs show some relationship with DEUFF and photoperiod sensitivity

Association analysis was done using the GLM (Table 6). At a significance level of p<0.01, SNP S17 showed a relationship with DEUFF under SD and ND; SNPs S162 and S1849 were associated with DEUFF under SD; and InDel D272 was associated with DEUFF under LD. Association with PS was also analyzed (Table 6). 

**Table 6:** General linear model association of SNP and InDel sites.

**Trait**	**DEUFF, SD**		**DEUFF, ND**		**DEUFF, LD**		**PS**	
	**p-value**	**R^2^**	**p-value**	**R^2^**	**p-value**	**R^2^**	**p-value**	**R^2^**
**S17**	0.0004	0.1972	0.0091	0.1135				
**S162**	0.0021	0.1972	0.0185	0.1328				
**D231**			0.0135	0.1023	0.0299	0.0829	0.0217	0.0921
**D272**			0.0254	0.2816	0.0037	0.3608	0.0001	0.4595
**S320**			0.0437	0.0695				
**D776**	0.0377	0.1105						
**S1458**			0.0416	0.0709				
**D1496**			0.0453	0.1047	0.0383	0.1138		
**D1737**			0.0482	0.1026			0.0371	0.1149
**S1844**			0.0465	0.0678				
**S1849**	0.0036	0.1818						
**D1849**			0.0465	0.0678				
**S1912**			0.0135	0.1023	0.0299	0.0829	0.0217	0.0921
**S1930**			0.0135	0.1023	0.0299	0.0829	0.0217	0.0921

DEUFF, days from expansion of unifoliates to first flowering; SD, short day; ND, natural day; LD, long day; PS, photoperiod sensitivity.

## Discussion

### The soybean population in the present study harbors rich diversity in DEUFF and PS

The soybean population investigated in this study was diverse in terms of geographic source, since the specimens were collected from North America and China and included 11 maturity groups, from MG000 to MGVIII. The samples therefore covered almost all of the 13 MGs [1]. The population showed a rich diversity of DEUFF under different photoperiod conditions; that is, LD, SD and ND. The days to first flowering diversified much more under LD than under the other two photoperiods (Table 1 and Figure 1). More importantly, the population exhibited a consecutive diversified day to first flowering. Individual plants within the population first-flowered every few days, from 20 to 85 days after the expansion of unifoliates, under natural day conditions (Table 1). As for PS, wild soybeans were on average more sensitive than cultivated ones. The PS of wild soybeans varied from 0.590 to 0.810, while that of cultivated soybeans varied from 0.056 to 0.752. This greater range of variation in cultivated soybeans facilitates their adaptation to different ecological environments. More importantly, the entire population showed a consecutive diversified spectrum of PS, from 0.056 to 0.81 (Table 1 and Figure 1). Therefore, the population studied here is richly diversified not only in terms of geographic source but also in terms of phenotype with regard to DEUFF and PS. 

### *GmFT2a* is under strong selection

The coding sequence of *GmFT2a* is highly conserved. A search of the known SNP data from the 31-Soybean Resequencing project [26] revealed only one synonymous A/T SNP site (ss249156869) in the *GmFT2a* coding sequence (Figure S1 in File S1). Furthermore, in an ongoing genome resequencing project involving over 100 cultivars, no SNP was found in the *GmFT2a* coding sequence (unpublished data). Therefore, the polymorphism of the *GmFT2a* coding sequence, which is under such strong selection, is probably not causally related to the diversity of flowering and maturity in soybeans. Adding that GmFT2a is involved in flowering transition and maintenance in soybean [24] and its homolog FT is an integrating factor of the flowering regulation network in *A. thaliana* [20,21], GmFT2a should be function essential for soybean adaptation.

Unlike the *GmFT2a* coding sequence, the *GmFT2a* promoter region is highly diversified. The degree of diversification of this region differs in different subpopulations (North American cultivars, Chinese cultivars, and wild soybeans). Wild soybeans were more diversified than cultivated ones, with a higher pairwise nucleotide diversity parameter (π) value (Table 2). Furthermore, the 19 wild soybeans examined in the present study included 14 of 17 possible haplotypes, while the 62 cultivated soybeans examined included only six of 17 possible haplotypes, supporting the assessment of a higher level of diversification in wild soybeans (Table 3). Examination of all 80 varieties revealed 31 polymorphic sites in the *GmFT2a* promoter region. It is interesting that there was no significant linkage disequilibrium in such a narrow region, since Lu et al. detected linkage disequilibrium in rice *Ghd7* [33]. This indicated that the *GmFT2a* promoter region was highly polymorphic. Considering that *GmFT2a* is a putative florigen gene that plays central roles in the flowering regulation network, this high degree of polymorphism might facilitate the adaptation of soybeans to different environments and requirements.

The *GmFT2a* promoter region is also under strong selection. The whole population and each of the three subpopulations considered individually all had significantly negative Tajima’s D values (Table 2). This suggested that, like cultivated soybeans, wild soybeans might also be under positive selection. However, the negative values might also result from low frequency mutations or population expansion. However, more evidence is required in order to define the selection model. Haplotype analysis also provides evidence for strong selection. A total of 17 haplotypes were set up using 16 stringent polymorphic sites. These haplotypes did not distribute equally. Haplotype HT06 was the most predominant one. It was found in 62 varieties (10 out of 19 wild soybeans, 13 out of 17 Chinese cultivars, and 39 out of 44 North American cultivars), covering all maturity groups, from MG000 to MGVIII. This is also suggested that *GmFT2a* might be under high selection pressure, indicating a high degree of risk when selecting *GmFT2a* haplotypes during breeding. On the other hand, high profit tends to stem from high risk. CS59, a currently predominant and widely adapted cultivar of Zhonghuang 13, includes HT15; its wide adaptation might have resulted from the selection of HT15. *GmFT2a* might function as an engine. The development of a strong and suitable engine might be the key to increasing production potential and adaptability.

### Polymorphism of *GmFT2a* is not related to maturity diversity

Further association analysis with GLM did not show a significant association between *GmFT2a* polymorphism and maturity diversity, which is consistent with the idea that *GmFT2a* is under strong selection. At the level of p<0.01, SNP S17 showed a relationship with the day to first flowering under SD and ND while SNPs S162 and S1849 showed such a relationship only under SD. The PLACE program (http://www.dna.affrc.go.jp/PLACE/) identified a CIACADIANLELHC element (CAANNNNATC, dark letter means SNP location) near SNP S17 (G /A). Near SNP S1849 (T/C), however, the program found an IBOXCORENT element (GATAAGR) [34,35]. Whereas the CIACADIANLELHC element is associated with circadian expression, the IBOXCORENT element is associated with light-responsive regulation; both are related to photoperiod responses in soybeans. However, more evidence is needed to elucidate how these SNPs function to regulate photoperiod reaction. Considering that *GmFT2a* is under high selection pressure, polymorphism in this gene does not appear to be responsible for maturity diversity.

There are nine maturity loci, *E1*-*E8* and *J* [14]. *E5*-*E8* and *J* have not been identified on the molecular level. *GmFT2a* is under highly stringent selection pressure, indicating that it probably does not correspond to one of the five unknown maturity loci. Indeed, *GmFT2a* also has nine paralogous genes, and little is known about these other nine genes [23,24]. Together, the nine maturity loci, *GmFT2a* and its relatives, and other flowering genes make up a complicated and elaborate flowering regulation network. There are many selection sites in the network that could be utilized in breeding new soybean varieties with good adaptation. *GmFT2a* functions downstream of other flowering genes, integrating flowering signals to regulate flowering. The predominance of HT06 indicates a core function of *GmFT2a* as an engine in the network. Therefore, it would be rather difficult to select *GmFT2a* directly in soybean breeding. However, future breeding should pay more attention to *GmFT2a* as a key element to be considered in approaches to breaking the bottleneck of soybean breeding. 

## Supporting Information

File S1 **A Word document with supplementary materials, including Table S1 and S2, Figure S1 and S2 and List S1.**
(DOC)
