# Supplementary material for: GmFT2a Polymorphism and Maturity Diversity in Soybeans
Source: PLoS One. 2013 Oct 14;8(10):e77474. doi: 10.1371/journal.pone.0077474 (PMC3796496; doi:10.1371/journal.pone.0077474)
Supplement: File S1 — A Word document with supplementary materials, including Table S1 and S2, Figure S1 and S2 and List S1. (DOC) [file pone.0077474.s001.doc]

**Table S1.** Varieties

| **Variety** | | **Variety** | | **Variety** | |
| --- | --- | --- | --- | --- | --- |
| **N. American Cultivars** | CS01^000^ | **N. American Cultivars** | CS29^V^ | **Chinese Cultivars** | CS59 |
|  | CS02^000^ |  | CS30^V^ |  | CS60 |
|  | CS03^00^ |  | CS31^V^ |  | CS61 |
|  | CS04^00^ |  | CS32^VI^ |  | CS62 |
|  | CS05^00^ |  | CS33^VI^ |  | CS63 |
|  | CS06^00^ |  | CS34^VI^ |  | HH |
|  | CS07^0^ |  | CS35^VI^ |  | ZG |
|  | CS08^0^ |  | CS36^VII^ | **Wild Soybeans** | H01 |
|  | CS09^0^ |  | CS37^VII^ |  | H02 |
|  | CS10 |  | CS38^VII^ |  | H03 |
|  | CS12^I^ |  | CS39^VII^ |  | H04 |
|  | CS13^I^ |  | CS40^VIII^ |  | H05 |
|  | CS14^I^ |  | CS41^VIII^ |  | H06 |
|  | CS15^II^ |  | CS42^VIII^ |  | H07 |
|  | CS16^II^ |  | CS43^VIII^ |  | H08 |
|  | CS17^II^ |  | JU |  | H09 |
|  | CS18^II^ |  | WM82 |  | H10 |
|  | CS19^II^ | **Chinese Cultivars** | CS46 |  | H11 |
|  | CS20^III^ |  | CS47 |  | H12 |
|  | CS21^III^ |  | CS48 |  | H13 |
|  | CS22^III^ |  | CS49 |  | H14 |
|  | CS23^III^ |  | CS50 |  | H15 |
|  | CS24^IV^ |  | CS51 |  | H16 |
|  | CS25^IV^ |  | CS52 |  | J1 |
|  | CS26^IV^ |  | CS53 |  | J2 |
|  | CS27 |  | CS54 |  | J3 |
|  | CS28^V^ |  | CS58 |  |  |

Superscript indicates maturity group.

1 ATG CCT AGT GGA AGT AGG GAT CCT CTC GTT GTT GGG GGA GTA ATT GGG GAT GTA TTG GAT

61 CCT TTT GAA TAT TCT ATT CCT ATG AGG GTT ACC TAC AAT AAC AGA GAT GTC AGC AAT GGA

121 TGT GAA TTC AAA CCC TCA CAA GTT GTC AAC CAA CCA AGG GTA AAT ATC GGT GGT GAT GAC

181 CTC AGG AAC TTC TAT ACT TTG ATT GCG GTT GAT CCC GAT GCA CCT AGC CCA AGT GAC CCC

241 AAT TTG AGA GAA TAC CTC CAT TGG TTG GTG ACT GAT ATC CCA GCA ACA ACA GGG GCT AGT

301 TTC GGC CAT GAG GTT GTA ACA TAT GAA AGT CCA AGA CCA ATG ATG GGG ATT CAT CGT TTG

361 GTG TTT GTG TTA TTT CGT CAA CTG GGT AGG GAG ACC GTG TAT GCA CC**Combin** GGA TGG CGC CAG

421 AAT TTC AAC ACT AAA GAA TTT GCT GAA CTT TAC AAC CTT GGA TTG CCA GTT GCT GCT GTC

481 TAT TTC AAC ATT CAG AGG GAA TCT GGT TCT GGT GGA AGG AGG TTA TAC TAA

**Figure S1. The SNP in the *GmFT2a* coding sequence (GenBank accession number: EU287455).** The yellow, bold-font letter represents the only SNP in the *GmFT2a* coding sequence. It is a synonymous A/T SNP designated ss249156869. The resulting codons (CCA or CCT/U) both encode the amino acid proline.

**Table S2. Polymorphisms in the *GmFT2a*** promoter region

| **SNP/Indel** | **Detail** | **SNP/Indel** | **Detail** |
| --- | --- | --- | --- |
| S17 | AG | D1524* | 1 |
| S162 | CTA | S1580 | TAG- |
| D231 | 4 | D1592 | 0-3 |
| D272 | 0-9 | D1608 | 1 |
| S320 | CA | D1737 | 2 |
| S455 | TA | S1844 | GA |
| D776 | 1 | D1849 | 20 |
| S1149 | TC | S1849* | -TC |
| S1458 | AT | S1912 | AG |
| D1496 | 44 | S1930 | AG |
| D1498* | 1 | S1944 | GA |
| D1518* | 0-2 | D2014 | 0/3/4 |
| D1520* | 1 | D2263 | 10 |
| D1522* | 1 | S2032 | AG |
| D1523* | 1 | S2228 | TC |
| S1523* | -CT |  |  |

S, SNP; D, InDel. Asterisks indicate SNPs or InDels located within a longer InDel. For SNPs, alleles are sorted descendingly by dominance. For InDels, a hyphen indicated a consecutive change, and a slash indicates possible alternative effects of the mutation.


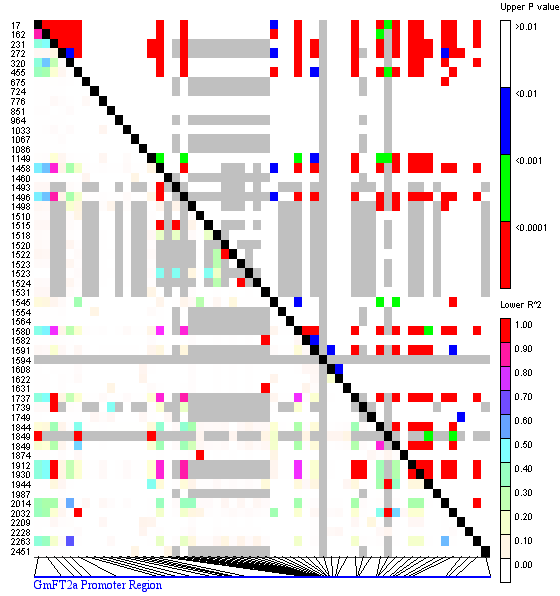


**Figure S2. Linkage disequilibrium over the *GmFT2a* promoter region.** Lower half shows the R2 between two polymorphism sites. Upper half shows the P value between two polymorphism sites.

**List S1. Individual sequences of the *GmFT2a* promoter region in the soybean plants examined in the present study**

>CS14.54

TATTAGACTCCTAATTTAATATTCTTGTTTTATTATAATGCTGATAAGTCTTGTAAATAA

GGGTGAGAAGCACGAATAATTAGTTCATGAGATGTGTATAATTATTATCCTACACGACTT

ATCTTTGATATTTCACACAAGTCTTTCAATGTATAACAAAAACTTTTTAGATACATTTAG

ACTATAGAACTAACAAGTTATATTTTGAACC-AAAAAACAAAGAGAGAGAGAGAGGAACA

TAGAGAAAAGAAGATATGAGAGTTTTTTTTTTTTTTTT---CTAAAACAGAAAGAAACTC

ATTATATAATAAACAAATTACTTTGAGACAAATTAACGTATGTAATAAAACAAAATCAAA

GTGGTAATTAAAATTATTTAATGGTAAACAGTCTAATAGTTAAAATAAAAATGGAAATCA

CATAAATTTTGTAATTGGCCCATTAAAAACAACACTAAGCTTTTAATTTGATTTTGAAAT

TCAAAATAATTTTATTAAATCACAAAGTAAAAGGTTTACAAAGCCGATCATGACAGTGCA

TGTGGGAGGCAAATCGGCATATTTGCACTACAAAAGG-ACCCATGAAGTCTCTGAACATG

CACGCAACACTTTAATCTCTTATTAGTTACTTTGAAAGCTTATTTATATATATAGACACG

CGTAAAAACTTCTTAACCAAGATTTTTTT-ACGTGCTTCC-TTCGCGTTTAATTTGGACC

ATCAAACCGTGCTCAACAGATAAAGAAAAGGGTGCTTTTGATTCAAGATATTGGCC-GAA

AAACACAAGATAGATCCTT-CGATAGATTAAGCCACGCATGAAACGCGAATCCAAAGTGA

TGAAGAAGTGCAGATAGATATTCGTTCACCATATAGAGGAGAATATATCATTCCTACAAA

CAAAATTGATGATGTTTCTAGAATAGAAACGTTAGAATCGAATATTCATCTGTTCCGGGT

GGGGAAGGGCTACTGAAAAGCTGAACTTTTTTAAGAGTTCGAACTTCGAAAGTCAATCAA

TGATTCTTAATTGAGAGGGAAAAGCTATAAAGGACGAGAAGGGAAGGAAATGTTTATAGG

GAATGAATTTCTAATGCATGATGGGAATGACAGATATGAGGAATATATAAAGGTGATTCT

TATATACATTGACATGGATCGGAATCTATTAACTTAAAGTTATTGGGGTCGTGAAATTTA

TTTAATTTTTCTAATTCGTGTCACATGTCACGACAATTAGTGGGG-AACATTTATGTATA

TAGAAATTTCAGAAATTTCTAGCAGCGTGATAAATATAAAGATTTTGGCACAATAAGTTT

TTGGATCATAATGAGTTT-CTATTTAATCAA-GGCA-TGTAA-TCTATTTTATTATTTAG

-TAGGG-AAACTGAAAACTTAGGGTTGCTATTTGTAGCTCCCACCCTTCTTAGTTCTTAC

TCTTTTCAATATTTTTTAAAAGTTTTA-TATAATACCT-AAATT-GCCCTCCTCCT----

----------------------------------------CACCCCATTGCTCTTCCGTT

GCTCCTCCATGTG-AGTTCTTGTCTTTTTTTTT-CTCTGGTAGCGTTTTGCTGCTCCTTT

TTTCACTCAAGTGTTGCCAATTAA-TTGACAAAAAATGGTTTCTGTTTCATATAGAAACT

ATGTTTTTGTTGTGTAGTCATACATTACGGAATCTAGTTT-CCATTAAATAAGTAAC--G

TGAAAAAAAA--TAAAAGGTGAAATATATATTGTTGGAAAAGAAGCTATGAGGTGCAAGA

ACCGATCACATGGAGAAGGCAATGAAAGACAAGGAGGAGCAATGGAAGA-----------

---------GAGAAAATGAGAAGATGGAAGGGATGTGAAAATGTTTGAAAAAAACGAGGT

GATCAGTTTTAAAATACGAATTTAGTATTTTCTTTTTAAGAAAATTCTTTCG-AAAGTCG

TGTTTTAAAACATGACTTTTATT-ATTTGAAGTCG---TGTTCTAAAACATGACTTA--T

TCATATCCTT-AATATTTTT---------AAAATTTATCCATTTGTAATATTTTTTAAAA

ATTGACCCATATATGTAAAATACCCGTCAAGATCTCTTTATTATTTTGAAAGCGAAAGCA

TATCACTTCAAACACAATGGAATCGAGGCTATTGACTAAGTATAAATAGAGAAGACTTCA

TATCGGGGTTCATAATTCATAACAAAGCAAACGAGTATATAAGAAAGCATAAGCCAAATT

TTGAGTAAACTAGTGTGCACACTATCCCATGCCTAGTGGAAGTAGGGATCCTCTCGTTGT

TGGGGGAGTAATTGGGGATGTATTGGATCCTTTTGAATATTCTATTCCTATGAGGGTTAC

CTACAATAACAGAGATGTCAGCAATGGATGTGAATTCAAACCCTCACAAGTTGTCAACCA

ACCAAGGGTAAATATCGGTGGTGATGACC

>CS27.21

TATTAGACTCCTAATTTAATATTCTTGTTTTATTATAATGCTGATAAGTCTTGTAAATAA

GGGTGAGAAGCACGAATAATTAGTTCATGAGATGCGTATAATTATTATCCTACACGACTT

ATCTTTGATATTTCACACAAGTCTTTCAATGTATAACAAAAACTTTTTAGATACATTTAG

ACTATAGAACTAACAAGTTATATTTTGAACC-AAAAAACAAAGAGAGAGAGAGAGGAACA

TAGAGAAAAGAAGATATGAGAGTTTTTTTTTTTTTTTT---CTAAAACAGAAAGAAACTC

ATTATATAATAAACAAATTACTTTGAGACAAATTAACGTATGTAATAAAACAAAATCAAA

GTGGTAATTAAAATTATTTAATGGTAAACAGTCTAATAGTTAAAATAAAAATGGAATTCA

CATAAATTTTGTAATTGGCCCATTAAAAACAACACTAAGCTTTTAATTTGATTTTGAAAT

TCAAAATAATTTTATTAAATCACAAAGTAAAAGGTTTACAAAGCCGATCATGACAGTGCA

TGTGGGAGGCAAATCGGCATATTTGCACTACAAAAGG-ACCCATGAAGTCTCTGAACATG

CACGCAACACTTTAATCTCTTATTAGTTACTTTGAAAGCTTATTTATATATATAGACACG

CGTAAAAACTTCTTAACCAAGATTTTTTT-ACGTGCTTCC-TTCGCGTTTAATTTGGACC

ATCAAACCGTGCTCAACAGATAAAGAAAAGGGTGCTTTTGATTCAAGATATTGGCC-GAA

AAACACAAGATAGATCCTT-CGATAGATTAAGCCACGCATGAAACGCGAATCCAAAGTGA

TGAAGAAGTGCAGATAGATATTCGTTCACCATATAGAGGAGAATATATCATTCCTACAAA

CAAAATTGATGATGTTTCTAGAATAGAAACGTTAGAATCGAATATTCATCTGTTCCGGGT

GGGGAAGGGCTACTGAAAAGCTGAACTTTTTTAAGAGTTCGAACTTCGAAAGTCAATCAA

TGATTCTTAATTGAGAGGGAAAAGCTATAAAGGACGAGAAGGGAAGGAAATGTTTATAGG

GAATGAATTTCTAATGCATGATGGGAATGACAGATATGAGGAATATATAAAGGTGATTCT

TATATACATTGACATGGATCGGAATCTATTAGCTTAAAGTTATTGGGGTCGTGAAATTTA

TTTAATTTTTCTAATTCGTGTCACATGTCACGACAATTAGTGGGG-AACATTTATGTATA

TAGAAATTTCAGAAATTTCTAGCAGCGTGATAAATATAAAGATTTTGGCACAATAAGTTT

TTGGATCATAATGAGTTT-CTATTTAATCAA-GGCA-TGTAA-TCTATTTTATTATTTAG

-TAGGG-AAACTGAAAACTTAGGGTTGCTATTTGTAGCTCCCACCCTTCTTAGTTCTTAC

TCTTTTCAATATTTTTTAAAAGTTTTA-TATAATACCT-AAATT-GCCCTCCTCCT----

----------------------------------------CACCCCATTGCTCTTCCGTT

GCTCCTCCATGTG-AGTTCTTGTCTTTTTTTTT-CTCTGGTAGCGTTTTGCTGCTCCTTT

TTTCACTCAAGTGTTGCCAATTAA-TTGACAAAAAATGGTTTCTGTTTCATATAGAAACT

ATGTTTTTGTTGTGTAGTCATACATTACGGAATCTAGTTT-CCATTAAATAAGTAAC--G

TGAAAAAAAA--TAAAAGGTGAAATATATATTGTTGGAAAAGAAGCTATGAGGTGCAAGA

ACCGATCACATGGAGAAGGCAATGAAAGACAAGGAGGAGCAATGGAAGA-----------

---------GAGAAAATGAGAAGATGGAAGGGATGTGAAAATGTTTGAAAAAAACGAGGT

GATCAGTTTTAAAATACGAATTTAGTATTTTCTTTTTAAGAAAATTCTTTCG-AAAGTCG

TGTTTTAAAACATGACTTTTATT-ATTTGAAGTCG---TGTTCTAAAACATGACTTA--T

TCATATCCTT-AATATTTTT---------AAAATTTATCCATTTGTAATATTTTTTAAAA

ATTGACCCATATATGTAAAATACCCGTCAAGATCTCTTTATTATTTTGAAAGCGAAAGCA

TATCACTTCAAACACAATGGAATCGAGGCTATTGACTAAGTATAAATAGAGAAGACTTCA

TATCGGGGTTCATAATTCATAACAAAGCAAACGAGTATATAAGAAAGCATAAGCCAAATT

TTGAGTAAACTAGTGTGCACACTATCCCATGCCTAGTGGAAGTAGGGATCCTCTCGTTGT

TGGGGGAGTAATTGGGGATGTATTGGATCCTTTTGAATATTCTATTCCTATGAGGGTTAC

CTACAATAACAGAGATGTCAGCAATGGATGTGAATTCAAACCCTCACAAGTTGTCAACCA

ACCAAGGGTAAATATCGGTGGTGATGACC

>CS13.20

TATTAGACTCCTAATTTAATATTCTTGTTTTATTATAATGCTGATAAGTCTTGTAAATAA

GGGTGAGAAACACGAATAATTAGTTCATGAGATGTGTATAATTATTATCCTACACGACTT

ATCTTTGATATTTCACACAAGTCTTTCAATGTATAACAAAAACTTTTTAGATACATTTAG

ACTATAGAACTAACAAGTTATATTTTGAACC-AAAAAACAAAGAGAGAGAGAGAGGAACA

TAGAGAAAAGAAGATATGAGAGTTTTTTTTTTTTTTTT---CTAAAACAGAAAGAAACTC

ATTATATAATAAACAAATTACTTTGAGACAAATTAACGTATGTAATAAAACAAAATCAAA

GTGGTAATTAAAATTATTTAATGGTAAACAGTCTAATAGTTAAAATAAAAATGGAAATCA

CATAAATTTTGTAATTGGCCCATTAAAAACAACACTAAGCTTTTAATTTGATTTTGAAAT

TCAAAATAATTTTATTAAATCACAAAGTAAAAGGTTTACAAAGCCGATCATGACAGTGCA

TGTGGGAGGCAAATCGGCATATTTGCACTACAAAAGG-ACCCATGAAGTCTCTGAACATG

CACGCAACACTTTAATCTCTTATTAGTTACTTTGAAAGCTTATTTATATATATAGACACG

CGTAAAAACTTCTTAACCAAGATTTTTTT-ACGTGCTTCC-TTCGCGTTTAATTTGGACC

ATCAAACCGTGCTCAACAGATAAAGAAAAGGGTGCTTTTGATTCAAGATATTGGCC-GAA

AAACACAAGATAGATCCTT-CGATAGATTAAGCCACGCATGAAACGCGAATCCAAAGTGA

TGAAGAAGTGCAGATAGATATTCGTTCACCATATAGAGGAGAATATATCATTCCTACAAA

CAAAATTGATGATGTTTCTAGAATAGAAACGTTAGAATCGAATATTCATCTGTTCCGGGT

GGGGAAGGGCTACTGAAAAGCTGAACTTTTTTAAGAGTTCGAACTTCGAAAGTCAATCAA

TGATTCTTAATTGAGAGGGAAAAGCTATAAAGGACGAGAAGGGAAGGAAATGTTTATAGG

GAATGAATTTCTAATGCATGATGGGAATGACAGATATGAGGAATATATAAAGGTGATTCT

TATATACATTGACATGGATCGGAATCTATTAACTTAAAGTTATTGGGGTCGTGAAATTTA

TTTAATTTTTCGAATTCGTGTCACATGTCACGACAATTAGTGGGG-AACATTTATGTATA

TAGAAATTTCAGAAATTTCTAGCAGCGTGATAAATATAAAGATTTTGGCACAATAAGTTT

TTGGATCATAATGAGTTT-CTATTTAATCAA-GGCA-TGTAA-TCTATTTTATTATTTAG

-TAGGG-AAACTGAAAACTTAGGGTTGCTATTTGTAGCTCCCACCCTTCTTAGTTCTTAC

TCTTTTCAATATTTTTTAAAAGTTTTA-TATAATACCT-AAATT-GCCCTCCTCCT----

----------------------------------------CACCCCATTGCTCTTCCGTT

GCTC-TCCATGTG-AGTTCTTGTCTTTTTTTTT-CTCTGGTAGCGTTTTGCTGCTCCTTT

TTTCACTCAAGTGTTGCCAATTAA-TTGACAAAAAATGGTTTCTGTTTCATATAGAAACT

ATGTTTTTGTTGTGTAGTCATACATTACGGAATCTAGTTT-CCATTAAATAAGTAAC--G

TGAAAAAAAA--TAAAAGGTGAAATATATATTGTTGGAAAAGAAGCTATGAGGTGCAAGA

ACCGATCACATGGAGAAGGCAATGAAAGACAAGGAGGAGCAATGGAAGA-----------

---------GAGAAAATGAGAAGATGGAAGGGATGTGAAAATGTTTGAAAAAAACGAGGT

GATCAGTTTTAAAATATGAATTTAGTATTTTCTTTTTAAGAAAATTCTTTCG-AAAGTCG

TGTTTTAAAACATGACTTTTATT-ATTTGAAGTCG---TGTTCTAAAACATGACTTA--T

TCATATCCTT-AATATTTTT---------AAAATTTATCCATTTGTAATATTTTTTAAAA

ATTGACCCATATATGTAAAATACCCGTCAAGATCTCTTTATTATTTTGAAAGCGAAAGCA

TATCACTTCAAACACAATGGAATCGAGGCTATTGACTAAGTATAAATAGAGAAGACTTCA

TATCGGGGTTCATAATTCATAACAAAGCAAACGAGTATATAAGAAAGCATAAGCCAAATT

TTGAGTAAACTAGTGTGCACACTATCCCATGCCTAGTGGAAGTAGGGATCCTCTCGTTGT

TGGGGGAGTAATTGGGGATGTATTGGATCCTTTTGAATATTCTATTCCTATGAGGGTTAC

CTACAATAACAGAGATGTCAGCAATGGATGTGAATTCAAACCCTCACAAGTTGTCAACCA

ACCAAGGGTAAATATCGGTGGTGATGACC

>CS26.15

TATTAGACTCCTAATTTAATATTCTTGTTTTATTATAATGCTGATAAGTCTTGTAAATAA

GGGTGAGAAGCACGAATAATTAGTTCATGAGATGTGTATAATTATTATCCTACACGACTT

ATCTTTGATATTTCACACAAGTCTTTCAATGTATAACAAAAACTTTTTAGATACATTTAG

ACTATAGAACTAACAAGTTATATTTTGAACC-AAAAAACAAAGAGAGAGAGAGAGGAACA

TAGAGAAAAGAAGATATGAGAGTTTTTTTTTTTTTTTT---CTAAAACAGAAAGAAACTC

ATTATATAATAAACAAATTACTTTGAGACAAATTAACGTATGTAATAAAACAAAATCAAA

GTGGTAATTAAAATTATTTAATGGTAAACAGTCTAATAGTTAAAATAAAAATGGAAATCA

CATAAATTTTGTAATTGGCCCATTAAAAACAACACTAAGCTTTTAATTTGATTTTGAAAT

TCAAAATAATTTTATTAAATCACAAAGTAAAAGGTTTACAAAGCCGATCATGACAGTGCA

TGTGGGAGGCAAATCGGCATATTTGCACTACAAAAGG-ACCCATGAAGTCTCTGAACATG

CACGCAACACTTTAATCTCTTATTAGTTACTTTGAAAGCTTATTTATATATATAGACACG

CGTAAAAACTTCTTAACCAAGATTTTTTT-ACGTGCTTCC-TTCGCGTTTAATTTGGACC

ATCAAACCGTGCTCAACAGATAAAGAAAAGGGCGCTTTTGATTCAAGATATTGGCC-GAA

AAACACAAGATAGATCCTT-CGATAGATTAAGCCACGCATGAAACGCGAATCCAAAGTGA

TGAAGAAGTGCAGATAGATATTCGTTCACCATATAGAGGAGAATATATCATTCCTACAAA

CAAAATTGATGATGTTTCTAGAATAGAAACGTTAGAATCGAATATTCATCTGTTCCGGGT

GGGGAAGGGCTACTGAAAAGCTGAACTTTTTTAAGAGTTCGAACTTCGAAAGTCAATCAA

TGATTCTTAATTGAGAGGGAAAAGCTATAAAGGACGAGAAGGGAAGGAAATGTTTATAGG

GAATGAATTTCTAATGCATGATGGGAATGACAGATATGAGGAATATATAAAGGTGATTCT

TATATACATTGACATGGATCGGAATCTATTAACTTAAAGTTATTGGGGTCGTGAAATTTA

TTTAATTTTTCTAATTCGTGTCACATGTCACGACAATTAGTGGGG-AACATTTATGTATA

TAGAAATTTCAGAAATTTCTAGCAGCGTGATAAATATAAAGATTTTGGCACAATAAGTTT

TTGGATCATAATGAGTTT-CTATTTAATCAA-GGCA-TGTAA-TCTATTTTATTATTTAG

-TAGGG-AAACTGAAAACTTAGGGTTGCTATTTGTAGCTCCCACCCTTCTTAGTTCTTAC

TCTTTTCAATATTTTTTAAAAGTTTTA-TATAATACCT-AAATT-GCCCTCCTCCT----

----------------------------------------CACCCCATTGCTCTTCCGTT

GCTCCTCCATGTG-AGTTCTTGTCTTTTTTTTT-CTCTGGTAGCGTTTTGCTGCTCCTTT

TTTCACTCAAGTGTTGCCAATTAA-TTGACAAAAAATGGTTTCTGTTTCATATAGAAACT

ATGTTTTTGTTGTGTAGTCATACATTACGGAATCTAGTTT-CCATTAAATAAGTAAC--G

TGAAAAAAAA--TAAAAAGTGAAATATATATTGTTGGAAAAGAAGCTATGAGGTGCAAGA

ACCGATCACATGGAGAAGGCAATGAAAGACAAGGAGGAGCAATGGAAGA-----------

---------GAGAAAATGAGAAGATGGAAGGGATGTGAAAATGTTTGAAAAAAACGAGGT

GATCAGTTTTAAAATACGAATTTAGTATTTTCTTTTTAAGAAAATTCTTTCG-AAAGTCG

TGTTTTAAAACATGACTTTTATT-ATTTGAAGTCG---TGTTCTAAAACATGACTTA--T

TCATATCCTT-AATATTTTT---------AAAATTTATCCATTTGTAATATTTTTTAAAA

ATTGACCCATATATGTAAAATACCCGTCAAGATCTCTTTATTATTTTGAAAGCGAAAGCA

TATCACTTCAAACACAATGGAATCGAGGCTATTGACTAAGTATAAATAGAGAAGACTTCA

TATCGGGGTTCATAATTCATAACAAAGCAAACGAGTATATAAGAAAGCATAAGCCAAATT

TTGAGTAAACTAGTGTGCACACTATCCCATGCCTAGTGGAAGTAGGGATCCTCTCGTTGT

TGGGGGAGTAATTGGGGATGTATTGGATCCTTTTGAATATTCTATTCCTATGAGGGTTAC

CTACAATAACAGAGATGTCAGCAATGGATGTGAATTCAAACCCTCACAAGTTGTCAACCA

ACCAAGGGTAAATATCGGTGGTGATGACC

>CS27.20

TATTAGACTCCTAATTTAATATTCTTGTTTTATTATAATGCTGATAAGTCTTGTAAATAG

GGGTGAGAAGCACGAATAATTAGTTCATGAGATGTGTATAATTATTATCCTACACGACTT

ATCTTTGATATTTCACACAAGTCTTTCAATGTATAACAAAAACTTTTTAGATACATTTAG

ACTATAGAACTAACAAGTTATATTTTGAACC-AAAAAACAAAGAGAGAGAGAGAGGAACA

TAGAGAAAAGAAGATATGAGAGTTTTTTTTTTTTTTTT---CTAAAACAGAAAGAAACTC

ATTATATAATAAACAAATTACTTTGAGACAAATTAACGTATGTAATAAAACAAAATCAAA

GTGGTAATTAAAATTATTTAATGGTAAACAGTCTAATAGTTAAAATAAAAATGGAAATCA

CATAAATTTTGTAATTGGCCCATTAAAAACAACACTAAGCTTTTAATTTGATTTTGAAAT

TCAAAATAATTTTATTAAATCACAAAGTAAAAGGTTTACAAAGCCGATCATGACAGTGCA

TGTGGGAGGCAAATCGGCATATTTGCACTACAAAAGG-ACCCATGAAGTCTCTGAACATG

CACGCAACACTTTAATCTCTTATTAGTTACTTTGAAAGCTTATTTATATATATAGACACG

CGTAAAAACTTCTTAACCAAGATTTTTTT-ACGTGCTTCC-TTCGCGTTTAATTTGGACC

ATCAAACCGTGCTCAACAGATAAAGAAAAGGGTGCTTTTGATTCAAGATATTGGCC-GAA

AAACACAAGATAGATCCTT-CGATAGATTAAGCCACGCATGAAACGCGAATCCAAAGTGA

TGAAGAAGTGCAGATAGATATTCGTTCACCATATAGAGGAGAATATATCATTCCTACAAA

CAAAATTGATGATGTTTCTAGAATAGAAACGTTAGAATCGAATATTCATCTGTTCCAGGT

GGGGAAGGGCTACTGAAAAGCTGAACTTTTTTAAGAGTTCGAACTTCGAAAGTCAATCAA

TGATTCTTAATTGAGAGGGAAAAGCTATAAAGGACGAGAAGGGAAGGAAATGTTTATAGG

GAATGAATTTCTAATGCATGATGGGAATGACAGATATGAGGAATATATAAAGGTGATTCT

TATATACATTGACATGGATCGGAATCTATTAACTTAAAGTTATTGGGGTCGTGAAATTTA

TTTAATTTTTCTAATTCGTGTCACATGTCACGACAATTAGTGGGG-AACATTTATGTATA

TAGAAATTTCAGAAATTTCTAGCAGCGTGATAAATATAAAGATTTTGGCACAATAAGTTT

TTGGATCATAATGAGTTT-CTATTTAATCAA-GGCA-TGTAA-TCTATTTTATTATTTAG

-TAGGG-AAACTGAAAACTTAGGGTTGCTATTTGTAGCTCCCACCCTTCTTAGTTCTTAC

TCTTTTCAATATTTTTTAAAAGTTTTA-TATAATACCT-AAATT-GCCCTCCTCCT----

----------------------------------------CACCCCATTGCTCTTCCGTT

GCTCCTCCATGTG-AGTTCTTGTCTCTTTTTTT-CTCTGGTAGCGTTTTGCTGCTCCTTT

TTTCACTCAAGTGTTGCCAATTAA-TTGACAAAAAATGGTTTCTGTTTCATATAGAAACT

ATGTTTTTGTTGTGTAGTCATACATTACGGAATCTAGTTT-CCATTAAATAAGTAAC--G

TGAAAAAAAA--TAAAAGGTGAAATATATATTGTTGGGAAAGAAGCTATGAGGTGCAAGA

ACCGATCACATGGAGAAGGCAATGAAAGACAAGGAGGAGCAATGGAAGA-----------

---------GAGAAAATGAGAAGATGGAAGGGATGTGAAAATGTTTGAAAAAAACGGGGT

GATCAGTTTTAAAATACGAATTTAGTATTTTCTTTTTAAGAAAATTCTTTCG-AAAGTCG

TGTTTTAAAACATGACTTTTATT-ATTTGAAGTCG---TGTTCTAAAACATGACTTA--T

TCATATCCTT-AATATTTTT---------AAAATTTATCCATTTGTAATATTTTTTAAAA

ATTGACCCATATATGTAAAATACCCGTCAAGACCTCTTTATTATTTTGAAAGCGAAAGCA

TATCACTTCAAACACAATGGAATCGAGGCTATTGACTAAGTATAAATAGAGAAGACTTCA

TATCGGGGTTCATAATTCATAACAAAGCAAACGAGTATATAAGAAAGCATAAGCCAAATT

TTGAGTAAACTAGTGTGCACACTATCTCATGCCTAGTGGGAGTAGGGATCCTCTCGTTGT

TGGGGGAGTAATTGGGGATGTATTGGATCCTTTTGAATATTCTATTCCTATGAGGGTTAC

CTACAATAACAGAGATGTCAGCAATGGATGTGAATTCAAACCCTCACAAGTTGTCAACCA

ACCAAGGGTAAATATCGGTGGTGATGACC

>CS17.10

TATTAGACTCCTAATTTAATATTCTTGTTTTATTATAATGCTGATAAGTCTTGTAAATAA

GGGTGAGAAGCACGAATAATTAGTTCATGAGATGTGTATAATTATTATCCTACACGACTT

ATCTTTGATATTTCACACAAGTCTTTCAATGTATAACAAAAACTTTTTAGATACATTTAG

ACTATAGAACTAACAAGTTATATTTTGAACC-AAAAAACAAAGAGAGAGAGAGAGGAACA

TAGAGAAAAGAAGATATGAGAGTTTTTTTTTTTTTTTTT--CTAAAACAGAAAGAAACTC

ATTATATAATAAACAAATTACTTTGAGACAAATTAACGTATGTAATAAAACAAAATCAAA

GTGGTAATTAAAATTATTTAATGGTAAACAGTCTAATAGTTAAAATAAAAATGGAAATCA

CATAAATTTTGTAATTGGCCCATTAAAAACAACACTAAGCTTTTAATTTGATTTTGAAAT

TCAAAATAATTTTATTAAATCACAAAGTAAAAGGTTTACAAAGCCGATCATGACAGTGCA

TGTGGGAGGCAAATCGGCATATTTGCACTACAAAAGG-ACCCATGAAGTCTCTGAACATG

CACGCAACACTTTAATCTCTTATTAGTTACTTTGAAAGCTTATTTATATATATAGACACG

CGTAAAAACTTCTTAACCAAGATTTTTTT-ACGTGCTTCC-TTCGCGTTTAATTTGGACC

ATCAAACCGTGCTCAACAGATAAAGAAAAGGGTGCTTTTGATTCAAGATATTGGCC-GAA

AAACACAAGATAGATCCTT-CGATAGATTAAGCCACGCATGAAACGCGAATCCAAAGTGA

TGAAGAAGTGCAGATAGATATTCGTTCACCATATAGAGGAGAATATATCATTCCTACAAA

CAAAATTGATGATGTTTCTAGAATAGAAACGTTAGAATCGAATATTCATCTGTTCCGGGT

GGGGAAGGGCTACTGAAAAGCTGAACTTTTTTAAGAGTTCGAACTTCGAAAGTCAATCAA

TGATTCTTAATTGAGAGGGAAAAGCTATAAAGGACGAGAAGGGAAGGAAATGTTTATAGG

GAATGAATTTCTAATGCATGATGGGAATGACAGATATGAGGAATATATAAAGGTGATTCT

TATATACATTGACATGGATCGGAATCTATTAACTTAAAGTTATTGGGGTCGTGAAATTTA

TTTAATTTTTCTAATTCGTGTCACATGTCACGACAATAAGTGGGGGAACATTTATGTATA

TAGAAATTTCAGAAATTTCTAGCAGCGTGATAAATATAAAGATTTTGGCACAATAAGTTT

TTGGATCATAATGAGTTTTCTATTTAATCAA-GGCAATGTAAATCTATTTTATTATTTAG

GTAGGG-AAACTGAAAACTTAGGGTTGCTATTTGTAGCTCCCACCCTTCT-AGTTCTTAC

TCTTTTCAATATTTTTTAAAAGTTTTAATATAATACCT-AA-TT-GCCCTCCTCCT----

----------------------------------------CACCCCATTGCTCTTTCGTT

GCTCCTCCATGTG-AGTTCTTGTCTTTTTTTTT-CTCTGGTAGCGTTT-GCTGCTCCTTT

TTTCACTCAAGTGTTGCCAATTAA-TTGACAAAAAATGGTTTCTGTTTCATATAGAAACT

ATGTTTTTGTTGTGTAGTCATACATTACGGAATCTAGTTT-CCATTAAATAAGTAAC--G

TGAAAAAAAA--TAAAAGGTGAAATATATATTGTTGGAAAAGAAGCTATGAGGTGCAAGA

ACCGATCACATGGAGAAGGCAATGAAAGACAAGGAGGAGCAATGGAAGA-----------

---------GAGAAAATGAGAAGATGGAAGGGATGTGAAAATGTTTGAAAAAAACGAGGT

GATCAGTTTTAAAATACGAATTTAGTATTTTCCTTTTAAGAAAATTCTTTCG-AAAGTCG

TGTTTTAAAACATGACTTTTATT-ATTTGAAGTCG---TGTTCTAAAACATGACTTA--T

TCATATCCTT-AATATTTTT---------AAAATTTATCCATTTGTAATATTTTTTAAAA

ATTGACCCATATATGTAAAATACCCGTCAAGATCTCTTTATTATTTTGAAAGCGAAAGCA

TATCACTTCAAACACAATGGAATCGAGGCTATTGACTAAGTATAAATAGAGAAGACTTCA

TATCGGGGTTCATAATTCATAACAAAGCAAACGAGTATATAAGAAAGCATAAGCCAAATT

TTGAGTAAACTAGTGTGCACACTATCCCATGCCTAGTGGAAGTAGGGATCCTCTCGTTGT

TGGGGGAGTAATTGGGGATGTATTGGATCCTTTTGAATATTCTATTCCTATGAGGGTTAC

CTACAATAACAGAGATGTCAGCAATGGATGTGAATTCAAACCCTCACAAGTTGTCAACCA

ACCAAGGGTAAATATCGGTGGTGATGACC

>CS18.43

TATTAGACTCCTAATTTAATATTCTTGTTTTATTATAATGCTGATAAGTCTTGTAAATAA

GGGTGAGAAGCACGAATAATTAGTTCATGAGATGTGTATAATTATTACCCTACACGACTT

ATCTTTGATATTTCACACAAGTCTTTCAATGTATAACAAAAACTTTTTAGATACATTTAG

ACTATAGAACTAACAAGTTATATTTTGAACC-AAAAAACAAAGAGAGAGAGAGAGGAACA

TAGAGAAAAGAAGATATGAGAGTTTTTTTTTTTTTTTT---CTAAAACAGAAAGAAACTC

ATTATATAATAAACAAATTACTTTGAGACAAATTAACGTATGTAATAAAACAAAATCAAA

GTGGTAATTAAAATTATTTAATGGTAAACAGTCTAATAGTTAAAATAAAAATGGAAATCA

CATAAATTTTGTAATTGGCCCATTAAAAACAACACTAAGCTTTTAATTTGATTTTAAAAT

TCAAAATAATTTTATTAAATCACAAAGTAAAAGGTTTACAAAGCCGATCATGACAGTGCA

TGTGGGAGGCAAATCGGCATATTTGCACTACAAAAGG-ACCCATGAAGTCTCTGAACATG

CACGCAACACTTTAATCTCTTATTAGTTACTTTGAAAGCTTATTTATATATATAGACACG

CGTAAAAACTTCTTAACCAAGATTTTTTT-ACGTGCTTCC-TTCGCGTTTAATTTGGACC

ATCAAACCGTGCTCAACAGATAAAGAAAAGGGTGCTTTTGATTCAAGATATTGGCC-GAA

AAACACAAGATAGATCCTT-CGATAGATTAAGCCACGCATGAAACGCGAATCCAAAGTGA

TGAAGAAGTGCAGATAGATATTCGTTCACCATATAGAGGAGAATATATCATTCCTACAAA

CAAAATTGATGATGTTTCTAGAATAGAAACGTTAGAATCGAATATTCATCTGTTCCGGGT

GGGGAAGGGCTACTGAAAAGCTGAACTTTTTTAAGAGTTCGGACTTCGAAAGTCAATCAA

TGATTCTTAATTGAGAGGGAAAAGCTATAAAGGACGAGAAGGGAAGGAAATGTTTATAGG

GAATGAATTTCTAATGCATGATGGGAATGACAGATATGAGGAATATATAAAGGTGATTCT

TATATACATTGACATGGATCGGAATCTATTAACTTAAAGTTATTGGGGTCGTGAAATTTA

TTTAATTTTTCTAATTCGTGTCACATGTCACGACAATTAGTGGGG-AACATTTATGTATA

TAGAAATTTCAGAAATTTCTAGCAGCGTGATAAATATAAAGATTTTGGCACAATAAGTTT

TTGGATCATAATGAGTTT-CTATTTAATCAA-GGCA-TGTAA-TCTATTTTATTATTTAG

-TAGGG-AAACTGAAAACTTAGGGTTGCTATTTGTAGCTCCCACCCTTCTTAGTTCTTAC

TCTTTTCAATATTTTTTAAAAGTTTTA-TATAATACCT-AAATT-GCCCTCCTCCT----

----------------------------------------CACCCCATTGCTCTTCCGTT

GCTCCTCCATGTG-AGTTCTTGTCTTTTTTTTT-CTCTGGTAGCGTTTTGCTGCTCCTTT

TTTCACTCAAGTGTTGCCAATTAA-TTGACAAAAAATGGTTTCTGTTTCATATAGAAACT

ATGTTTTTGTTGTGTAGTCATACATTACGGAATCTAGTTT-CCATTAAATAAGTAAC--G

TGAAAAAAAA--TAAAAGGTGAAATATATATTGTTGGAAAAGAAGCTATGAGGTGCAAGA

ACCGATCACATGGAGAAGGCAATGAAAGACAAGGAGGAGCAATGGAAGA-----------

---------GAGAAAATGAGAAGATGGAAGGGATGTGAAAATGTTTGAAAAAAACGAGGT

GATCAGTTTTAAAATACGAATTTAGTATTTTCTTTTTAAGAAAATTCTTTCG-AAAGTCG

TGTTTTAAAACATGACTTTTATT-ATTTGAAGTCG---TGTTCTAAAACATGACTTA--T

TCATATCCTT-AATATTTTT---------AAAATTTATCCATTTGTAATATTTTTTAAAA

GTTGACCCATATATGTAAAATACCCGTCAAGATCTCTTTATTATTTTGAAAGCGAAAGCA

TATCACTTCAAACACAATGGAATCGAGGCTATTGACTAAGTATAAATAGAGAAGACTTCA

TATCGGGGTTCATAATTCATAACAAAGCAAACGAGTATATAAGAAAGCATAAGCCAAATT

TTGAGTAAACTGGTGTGCACACTATCCCATGCCTAGTGGAAGTAGGGATCCTCTCGTTGT

TGGGGGAGTAATTGGGGATGTATTGGATCCTTTTGAATATTCTATTCCTATGAGGGTTAC

CTACAATAACAGAGATGTCAGCAATGGATGTGAATTCAAACCCTCACAAGTTGTCAACCA

ACCAAGGGTAAATATCGGTGGTGATGACC

>CS61.23

TATTAGACTCCTAATTTAATATTCTTGTTTTATTATAATGCTGATAAGTCTTGTAAATAA

GGGTGAGAAGCACGAATAATTAGTTCATGAGATGTGTATAATTATTATCCTACACGACTT

ATCTTTGATATTTCACACAAGTCTTTCAATGTATAACAAAAACTTTTTAGATACATTTAG

ACTATAGAACTAACAAGTTATATTTTGAACC-AAAAAACAAAGAGAGAGAGAGAGGAACA

TAGAGAAAAGAAGATATGAGAGTTTTTTTTTTTTTTTTT--CTAAAACAGAAAGAAACTC

ATTATATAATAAACAAATTACTTTGAGACAAATTAACGTATGTAATAAAACAAAATCAAA

GTGGTAATTAAAATTATTTAATGGTAAACAGTCTAATAGTTAAAATAAAAATGGAAATCA

CATAAATTTTGTAATTGGCCCATTAAAAACAACACTAAGCTTTTAATTTGATTTTGAAAT

TCAAAATAATTTTATTAAATCACAAAGTAAAAGGTTTACAAAGCCGATCATGACAGTGCA

TGTGGGAGGCAAATCGGCATATTTGCACTACAAAAGG-ACCCATGAAGTCTCTGAACATG

CACGCAACACTTTAATCTCTTATTAGTTACTTTGAAAGCTTATTTATATATATAGACACG

CGTAAAAACTTCTTAACCAAGATTTTTTT-ACGTGCTTCC-TTCGCGTTTAATTTGGACC

ATCAAACCGTGCTCAACAGATAAAGAAAAGGGTGCTTTTGATTCAAGATATTGGCC-GAA

AAACACAAGATAGATCCTT-CGATAGATTAAGCCACGCATGAAACGCGAATCCAAAGTGA

TGAAGAAGTGCAGATAGATATTCGTTCACCATATAGAGGAGAATATATCATTCCTACAAA

CAAAATTGATGATGTTTCTAGAATAGAAACGTTAGAATCGAATATTCATCTGTTCCGGGT

GGGGAAGGGCTACTGAAAAGCTGAACTTTTTTAAGAGTTCGAACTTCGAAAGTCAATCAA

TGATTCTTAATTGAGAGGGAAAAGCTATAAAGGACGAGAAGGGAAGGAAATGTTTATAGG

GAATGAATTTCTAATGCATGATGGGAACGACAGATATGAGGAATATATAAAGGTGATTCT

TATATACATTGACATGGATCGGAATCTATTAACTTAAAGTTATTGGGGTCGTGAAATTTA

TTTAATTTTTCTAATTCGTGTCACATGTCACGACAATTAGTGGGG-AACATTTATGTATA

TAGAAATTTCAGAAATTTCTAGCAGCGTGATAAATATAAAGATTTTGGCACAATAAGTTT

TTGGATCATAATGAGTTT-CTATTTAATCAA-GGCA-TGTAA-TCTATTTTATTATTTAG

-TAGGG-AAACTGAAAACTTAGGGTTGCTATTTGTAGCTCCCACCCTTCTTAGTTCTTAC

TCTTTTCAATATTTTTTAAAAGTTTTA-TATAATACCT-AAATT-GCCCTCCTCCT----

----------------------------------------CACCCCATTGCTCTTCCGTT

GCTCCTCCATGTG-AGTTCTTGTCTTTTTTTTT-CTCTGGTAGCGTTTTGCTGCTCCTTT

TTTCACTCAAGTGTTGCCAATTAA-TTGACAAAAAATGGTTTCTGTTTCATATAGAAACT

ATGTTTTTGTTGTGTAGTCATACATTACGGAATCTAGTTT-CCATTAAATAAGTAAC--G

TGAAAAAAAA--TAAAAGGTGAAATATATATTGTTGGAAAAGAAGCTATGAGGTGCAAGA

ACCGATCACATGGAGAAGGCAATGAAAGACAAGGAGGAGCAATGGAAGA-----------

---------GAGAAAATGAGAAGATGGAAGGGATGTGAAAATGTTTGAAAAAAACGAGGT

GATCAGTTTTAAAATACGAATTTAGTATTTTCTTTTTAAGAAAATTCTTTCG-AAAGTCG

TGTTTTAAAACATGACTTTTATT-ATTTGAAGTCG---TGTTCTAAAACATGACTTA--T

TCATATCCTT-AATATTTTT---------AAAATTTATCCATTTGTAATATTTTTTAAAA

ATTGACCCATATATGTAAAATACCCGTCAAGATCTCTTTATTATTTTGAAAGCGAAAGCA

TATCACTTCAAACACAATGGAATCGAGGCTATTGACTAAGTATAAATAGAGAAGACTTCA

TATCGGGGTTCGTAATTCATAACAAAGCAAACGAGTATATAAGAAAGCATAAGCCAAATT

TTGAGTAAACTAGTGTGCACACTATCCCATGCCTAGTGGAAGTAGGGATCCTCTCGTTGT

TGGGGGAGTAATTGGGGATGTATTGGATCCTTTTGAATATTCTATTCCTATGAGGGTTAC

CTACAATAACAGAGATGTCAGCAATGGATGTGAATTCAAACCCTCACAAGTTGTCAACCA

ACCAAGGGTAAATATCGGTGGTGATGACC

>CS06.46

TATTAGACTCCTAATTTAATATTCTTGTTTTATTATAATGCTGATAAGTCTTGTAAATAA

GGGTGAGAAGCACGAATAATTAGTTCATGAGATGTGTATAATCATTATCCTACACGACTT

ATCTTTGATATTTCACACAAGTCTTTCAATGTATAACAAAAACTTTTTAGATACATTTAG

ACTATAGAACTAACAAGTTATATTTTGAACC-AAAAAACAAAGAGAGAGAGAGAGGAACA

TAGAGAAAAGAAGATATGAGAGTTTTTTTTTTTTTTTTT--CTAAAACAGAAAGAAACTC

ATTATATAATAAACAAATTACTTTGAGACAAATTAACGTATGTAATAAAACAAAATCAAA

GTGGTAATTAAAATTATTTAATGGTAAACAGTCTAATAGTTAAAATAAAAATGGAAATCA

CATAAATTTTGTAATTGGCCCATTAAAAACAACACTAAGCTTTTAATTTGATTTTGAAAT

TCAAAATAATTTTATTAAATCACAAAGTAAAAGGTTTACAAAGCCGATCATGACAGTGCA

TGTGGGAGGCAAATCGGCATATTTGCACTACAAAAGG-ACCCATGAAGTCTCTGAACATG

CACGCAACACTTTAATCTCTTATTAGTTACTTTGAAAGCTTATTTATATATATAGACACG

CGTAAAAACTTCTTAACCAAGATTTTTTT-ACGTGCTTCC-TTCGCGTTTAATTTGGACC

ATCAAACCGTGCTCAACAGATAAAGAAAAGGGTGCTTTTGATTCAAGATATTGGTC-GAA

AAACACAAGATAGTTCCTT-CGATAGATTAAGCCACGCATGAAACGCGAATCCAAAGTGA

TGAAGAAGTGCAGATAGATATTCGTTCACCATATAGAGGAGAATATATCATTCCTACAAA

CAAAATTGATGATGTTTCTAGAATAGAAACGTTAGAATCGAATATTCATCTGTTCCGGGT

GGGGAAGGGCTACTGAAAAGCTGAACTTTTTTAAGAGTTCGAACTTCGAAAGTCAATCAA

TGATTCTTAATTGAGAGGGAAAAGCTATAAAGGACGAGAAGGGAAGGAAATGTTTATAGG

GAATGAATTTCTAATGCATGATGGGAATGACAGATATGAGGAATATATAAAGGTGATTCT

TATATACATTGACATGGATCGGAATCTATTAACTTAAAGTTATTGGGGTCGTGAAATTTA

TTTAATTTTTCTAATTCGTGTCACATGTCACGACAATTAGTGGGG-AACATTTATGTATA

TAGAAATTTCAGAAATTTCTAGCAGCGTGATAAATATAAAGATTTTGGCACAATAAGTTT

TTGGATCATAATGAGTTT-CTATTTAATCAA-GGCA-TGTAA-TCTATTTTATTATTTAG

-TAGGG-AAACTGAAAACTTAGGGTTGCTATTTGTAGCTCCCACCCTTCTTAGTTCTTAC

TCTTTTCAATATTTTTTAAAAGTTTTA-TATAATACCT-AAATT-GCCCTCCTCCT----

----------------------------------------CACCCCATTGCTCTTCCGTT

GCTCCTCCATGTG-AGTTCTTGTCTTTTTTTT--CTCTGGTAGCGTTTTGCCGCTCCTTT

TTTCACTCAAGTGTTGCCAATTAA-TTGACAAAAAATAGTTTCTGTTTCATATAGAAACT

ATGTTTTTGTTGTGTAGTCATACATTACGGAATCTAGTTT-CCATTAAATAAGTAAC--G

TGAAAAAAAA--TAAAAGGTGAAATATATATTGTTGGAAAAGAAGCTATGAGGTGCAAGA

ACCGATCACATGGAGAAGGCAATGAAAGACAAGGAGGAGCAATGGAAGA-----------

---------GAGAAAATGAGAAGATGGAAGGGATGTGAAAATGTTTGAAAAAAACGAGGT

GATCAGTTTTAAAATACGAATTTAGTATTTTCTTTTTAAGAAAATTCTTTCG-AAAGTCG

TGTTTTAAAACATGACTTTTATT-ATTTGAAGTCG---TGTTCTAAAACATGACTTA--T

TCATATCCTT-AATATTTTT---------AAAATTTATCCATTTGTAATATTTTTTAAAA

ATTGACCCATATATGTAAAATACCCGTCAAGATCTCTTTATTATTTTGAAAGCGAAAGCA

TATCACTTCAAACACAATGGAATCGAGGCTACTGACTAAGTATAAATAGAGAAGACTTCA

TATCGGGGTTCATAATTCATAACAAAGCAAACGAGTATATAAGAAAGCATAAGCCAAATT

TTGAGTAAACTAGTGTGCACACTATCCCATGCCTAGTGGAAGTAGGGATCCTCTCGTTGT

TGGGGGAGTAATTGGGGATGTATTGGATCCTTTTGAATATTCTATTCCTATGAGGGTTAC

CTACAATAACAGAGATGTCAGCAATGGATGTGAATTCAAACCCTCACAAGTTGTCAACCA

ACCAAGGGTAAATATCGGTGGTGATGACC

>CS61.19

TATTAGACTCCTAATTTAATATTCTTGTTTTATTATAATGCTGATAAGTCTTGTAAATAA

GGGTGAGAAGCACGAATAATTAGTTCATGAGATGTGTATAATTATTATCCTACACGACTT

ATCTTTGATATTTCACACAAGTCTTTCAATGTATAACAAAAACTTTTTAGATACATTTAG

ACTATAGAACTAACAAGTTATATTTTGAACC-AAAAAACAAAGAGAGAGAGAGAGGAACA

TAGAGAAAAGAAGATATGAGAGTTTTTTTTTTTTTTTTT--CTAAAACAGAAAGAAACTC

ATTATATAATAAACAAATTACTTTGAGACAAATTAACGTATGTAATAAAACAAAATCAAA

GTGGTAATTAAAATTATTTAATGGTAAACAGTCTAATAGTTAAAATAAAAATGGAAATCA

CATAAATTTTGTAATTGGCCCATTAAAAACAACACTAAGCTTTTAATTTGATTTTGAAAT

TCAAAATAATTTTATTAAATCACAAAGTAAAAGGTTTACAAAGCCGATCATGACAGTGCA

TGTGGGAGGCAAATCGGCATATTTGCACTACAAAAGG-ACCCATGAAGTCTCTGAACATG

CACGCAACACTTTAATCTCTTATTAGTTACTTTGAAAGCTTATTTATATATATAGACACG

CGTAAAAACTTCTTAACCAAGATTTTTTT-ACGTGCTTCC-TTCGCGTTTAATTTGGACC

ATCAAACCGTGCTCAACAGATAAAGAAAAGGGTGCTTTTGATTCAAGATATTGGCC-GAA

AAACACAAGATAGATCCTT-CGATAGATTAAGCCACGCATGAAACGCGAATCCAAAGTGA

TGAAGAAGTGCAGATAGATATTCGTTCACCATATAGAGGAGAATATATCATTCCTACAAA

CAAAATTGATGATGTTTCTAGAATAGAAACGTTAGAATCGAATATTCATCTGTTCCGGGT

GGGGAAGGGCTACTGAAAAGCTGAACTTTTTTAAGAGTTCGAACTTCGAAAGTCAATCAA

TGATTCTTAATTGAGAGGGAAAAGCTATAAAGGACGAGAAGGGAAGGAAATGTTTATAGG

GAATGAATTTCTAATGCATGATGGGAATGACAGATATGAGGAATATATAAAGGTGATTCT

TATATACATTGACATGGATCGGAATCTATTAACTTAAAGTTATTGGGGTCGTGAAATTTA

TTTAATTTTTCTAATTCGTGTCACATGTCACGACAATTAGTGGGG-AACATTTATGTATA

TAGAAATTTCAGAAATTTCTAGCAGCGTGATAAATATAAAGATTTTGGCACAATAAGTTT

TTGGATCATAATGAGTTT-CTATTTAATCAA-GGCA-TGTAA-TCTATTTTATTATTTAG

-TAGGG-AAACTGAAAACTTAGGGTTGCTGTTTGTAGCTCCCACCCTTCTTAGTTCTTAC

TCTTTTCAATATTTTTTAAAAGTTTTA-TATAATACCT-AAATT-GCCCTCCTCCT----

----------------------------------------CACCCCATTGCTCTTCCGTT

GCTCCTCCATGTG-AGTTCTTGTCTTTTTTTTT-CTCTGGTAGCGTTTTGCTGCTCCTTT

TTTCACTCAAGTGTTGCCAATTAA-TTGACAAAAAATGGTTTCTGTTTCATATAGAAACT

ATGTTTTTGTTGTGTAGTCATACATTACGGAATCTAGTTT-CCATTAAATAAGTAAC--G

TGAAAAAAAA--TAAAAGGTGAAATATATATTGTTGGAAAAGAAGCTATGAGGTGCAAGA

ACCGATCACATGGAGAAGGCAATGAAAGACAAGGAGGAGCAATGGAAGA-----------

---------GAGAAAATGAGAAGATGGAAGGGATGTGAAAATGTTTGAAAAAAACGAGGT

GATCAGTTTTAAAATACGAATTTAGTATTTTCCTTTTAAGAAAATTCTTTCG-AAAGTCG

TGTTTTAGAACATGACTTTTATT-ATTTGAAGTCG---TGTTCTAAAACATGACTTA--T

TCATATCCTT-AATATTTTT---------AAAATTTATCCATTTGTAATATTTTTTAAAA

ATTGACCCATATATGTAAAATACCCGTCAAGATCTCTTTATTATTTTGAAAGCGAAAGCA

TATCACTTCAAACACAATGGAATCGAGGCTATTGACTAAGTATAAATAGAGAAGACTTCA

TATCGGGGTTCATAATTCATAACAAAGCAAACGAGTATATAAGAAAGCATAAGCCAAATT

TTGAGTAAACTAGTGTGCACACTATCCCATGCCTAGTGGAAGTAGGGATCCTCTCGTTGT

TGGGGGAGTAATTGGGGATGTATTGGATCCTTTTGAATATTCTATTCCTATGAGGGTTAC

CTACAATAACAGAGATGTCAGCAATGGATGTGAATTCAAACCCTCACAAGTTGTCAACCA

ACCAAGGGTAAATATCGGTGGTGATGACC

>CS17.19

TATTAGACTCCTAATTTAATATTCTTGTTTTATTATAATGCTGATAAGTCTTGTAAATAA

GGGTGAGAAGCACGAATAATTAGTTCATGAGATGTGTATAATTATTATCCTACACGACTT

ATCTTTGATATTTCACACAAGTCTTTCAATGTATAACAAAAACTTTTTAGATACATTTAG

ACTATAGAACTAACAAGTTATATTTTGAACC-AAAAAACAAAGAGAGAGAGAGAGGAACA

TAGAGAAAAGAAGATATGAGAGTTTTTTTTTTTTTTTT---CTAAAACAGAAAGAAACTC

ATTATATAATAAACAAATTACTTTGAGACAAATTAACGTATGTAATAAAACAAAATCAAA

GTGGTAATTAAAATTATTTAATGGTAAACAGTCTAATAGTTAAAATAAAAATGGAAATCA

CATAAATTTTGTAATTGGCCCATTAAAAACAACACTAAGCCTTTAATTTGATTTTGAAAT

TCAAAATAATTTTATTAAATCACAAAGTAAAAGGTTTACAAAGCCGATCATGACAGTGCA

TGTGGGAGGCAAATCGGCATATTTGCACTACAAAAGG-ACCCATGAAGTCTCTGAACATG

CACGCAACACTTTAATCTCTTATTAGTTACTTTGAAAGCTTATTTATATATATAGACACG

CGTAAAAACTTCTTAACCAAGATTTTTTT-ACGTGCTTCC-TTCGCGTTTAATTTGGACC

ATCAAACCGTGCTCAACAGATGAAGAAAAGGGTGCTTTTGATTCAAGATATTGGCC-GAA

AAACACAAGATAGATCCTT-CGATAGATTAAGCCACGCATGAAACGCGAATCCAAAGTGA

TGAAGAAGTGCAGATAGATATTCGTTCACCATATAGAGGAGAATATATCATTCCTACAAA

CAAAATTGATGATGTTTCTAGAATAGAAACGTTAGAATCGAATATTCATCTGTTCCGGGT

GGGGAAGGGCTACTGAAAAGCTGAACTTTTTTAAGAGTTCGAACTTCGAAAGTCAATCAA

TGATTCTTAATTGAGAGGGAAAAGCTATAAAGGACGAGAAGGGAAGGAAATGTTTATAGG

AAATGAATTTCTAATGCATGATGGGAATGACAGATATGAGGAATATATAAAGGTGATTCT

TATATACATTGACATGGATCGGAATCTACTAACTTAAAGTTATTGGGGTCGTGAAATTTA

TTTAATTTTTCTAATTCGTGTCACATGTCACGACAATTAGTGGGG-AACATTTATGTATA

TAGAAATTTCAGAAATTTCTAGCAGCGTGATAAATATAAAGATTTTGGCACAATAAGTTT

TTGGATCATAATGAGTTT-CTATTTAATCAA-GGCA-TGTAA-TCTATTTTATTATTTAG

-TAGGG-AAACTGAAAACTTAGGGTTGCTATTTGTAGCTCCCACCCTTCTTAGTTCTTAC

TCTTTTCAATATTTTTTAAAAGTTTTA-TATAATACCT-AAATT-GCCCTCCTCCT----

----------------------------------------CACCCCATTGCTCTTCCGTT

GCTCCTCCATGTG-AGTTCTTGTCTTTTTTTTT-CTCTGGTAGCGTTTTGCTGCTCCTTT

TTTCACTCAAGTGTTGCCAATTAAATTGACAAAAAATGGTTTCTGTTTCATATAGAAACT

ATGTCTTTGTTGTGTAGTCATACATTACGGAATCTAGTTT-CCATTAAATAAGTAAC--G

TGAAAAAAAA--TAAAAGGTGAAATATATATTGTTGGAAAAGAAGCTATGAGGTGCAAGA

ACCGATCACATGGAGAAGGCAATGAAAGACAAGGAGGAGCAATGGAAGA-----------

---------GAGAAAATGAGAAGATGGAAGGGATGTGAAAATGTTTGAAAAAAACGAGGT

GATCAGTTTTAAAATACGAATTTAGTATTTTCTTTTTAAGAAAATTCTTTCG-AAAGTCG

TGTTTTAAAACATGACTTTTATT-ATTTGAAGTCG---TGTTCTAAAACATGACTTA--T

TCATATCCTT-AATATTTTT---------AAAATTTATCCATTTGTAATATTTTTTAAAA

ATTGACCCATATATGTAAAATACCCGTCAAGATCTCTTTATTATTTTGAAAGCGAAAGCA

TATCACTTCAAACACAATGGAATCGAGGCTATTGACTAAGTATAAATAGAGAAGACTTCA

TATCGGGGTTCATAATTCATAACAAAGCAAACGAGTATATAAGAAAGCATAAGCCAAATT

TTGAGTAAACTAGTGTGCACACTATCCCATGCCTAGTGGAAGTAGGGATCCTCTCGTTGT

TGGGGGAGTAATTGGGGATGTATTGGATCCTTTTGAATATTCTATTCCTATGAGGGTTAC

CTACAATAACAGAGATGTCAGCAATGGATGTGAATTCAAACCCTCACAAGTTGCCAACCA

ACCAAGGGTAAATATCGGTGGTGATGACC

>CS28.13

TATTAGACTCCTAATTTAATATTCTTGTTTTATTATAATGCTGATAAGTCTTGTAAATAA

GGGTGGGAAGCACGAATAATTAGTTCATGAGATGTGTATAATTATTATCCTACACGACTT

ATCTTTGATATTTCACACAAGTCTTTCAATGTATAACAAAAACTTTTTAGATACATTTAG

ACTATAGAACTAACAAGTTATATTTTGAACC-AAAAAACAAAGAGAGAGAGAGAGGAACA

TAGAGAAAAGAAGATATGAGAGTTTTTTTTTTTTTTT----CTAAAACGGAAAGAAACTC

ATTATATAATAAACAAATTACTTTGAGACAAATTAACGTATGTAATAAAACAAAATCAAA

GTGGTAATTAAAATTATTTAATGGTAAACAGTCTAATAGTTAAAATAAAAATGGAAATCA

CATAAATTTTGTAATTGGCCCATTAAAAACAACACTAAGCTTTTAATTTGATTTTGAAAT

TCAAAATAATTTTATTAAATCACAAAGTAAAAGGTTTACAAAGCCGATCATGACAGTGCA

TGTGGGAGGCAAATCGGCATATTTGCACTACAAAAGG-ACCCATGAAGTCTCTGAACATG

CACGCAACACTTTAATCTCTTATTAGTTACTTTGAAAGCTTATTTATATATATAGACACG

CGTAAAA-CTTCTTAACCAAGATTTTTTT-ACGTGCTTCC-TTCGCGTTTAATTTGGACC

ATCAAACCGTGCTCAACAGATAAAGAAAAGGGTGCTTTTGATTCAAGATATTGGCC-GAA

AAACACAAGATAGATCCTT-CGATAGATTAAGCCACGCATGAAACGCGAATCCAAAGTGA

TGAAGAAGTGCAGATAGATATTCGTTCACCATATAGAGGAGAATATATCATTCCTACAAA

CAAAATTGATGATGTTTCTAGAATAGAAACGTTAGAATCGAATATTCATCTGTTCCGGGT

GGGGAAGGGCTACTGAAAAGCTGAACTTTTTTAAGAGTTCGAACTTCGAAAGTCAATCAA

TGATTCTTAATTGAGAGGGAAAAGCTATAAAGGACGAGAAGGGAAGGAAATGTTTATAGG

GAATGAATTTCTAATGCATGATGGGAATGACAGATATGAGGAATATATAAAGGTGATTCT

TATATACATTGACATGGATCGGAATCTATTAACTTAAAGTTATTGGGGTCGTGAAATTTA

TTTAATTTTTCTAATTCGTGTCACATGTCACGACAATTAGTGGGG-AACATTTATGTATA

TAGAAATTTCAGAAATTTCTAGCAGCGTGATAAATATAAAGATTTTGGCACAATAAGTTT

TTGGATCATAATGAGTTT-CTATTTAATCAA-GGCA-TGTAA-TCTATTTTATTATTTAG

-TAGGG-AAACTGAAAACTTAGGGTTGCTATTTGTAGCTCCCACCCTTCTTAGTTCTTAC

TCTTTTCAATATTTTTTAAAAGTTTTA-TATAATACCT-AAATT-GCCCTCCTCCT----

----------------------------------------CACCCCATTGCTCTTCCGTT

GCTCCTCCATGTG-AGTTCTTGTCTTTTTTTTT-CTCTGGTAGCGTTTTGCTGCTCCTTT

TTTCACTCAAGTGTTGCCAATTAA-TTGACAAAAAATGGTTTCTGTTTCATATAGAAACT

ATGTTTTTGTTGTGTAGTCATACATTACGGAATCTAGTTT-CCATTAAATAAGTAAC--G

TGAAAAAAAA--TAAAAGGTGAAATATATATTGTTGGAAAAGAAGCTATGAGGTGCAAGA

ACCGATCACATGGAGAAGGCAATGAAAGACAAGGAGGAGCAATGGAAGA-----------

---------GAGAAAATGAGAAGATGGAAGGGATGTGAAAATGTTTGAAAAAAACGAGGT

GATCAGTTTTAAAATACGAATTTAGTATTTTCTTTTTAAGAAAATTCTTTCG-AAAGTCG

TGTTTTAAAACATGACTTTTATT-ATTTGAAGTCG---TGTTCTAAAACATGACTTA--T

TCATATCCTT-AATATTTTT---------AAAATTTATCCATTTGTAATATTTTTTAAAA

ATTGACCCATATATGTAAAATACCCGTCAAGATCTCTTTATTATTTTGAAAGCGAAAGCA

TATCACTTCAAACACAATGGAATCGAGGCTATTGACTAAGTATAAATAGAGAAGACTTCA

TATCGGGGTTCATAATTCATAACAAAGCAAACGAGTATATAAGAAAGCATAAGCCAAATT

TTGAGTAAACTAGTGTGCACACTATCCCATGCCTAGTGGAAGTAGGGATCCTCTCGTTGT

TGGGGGAGTAATTGGGGATGTATTGGATCCTTTTGAATATTCTATTCCTATGAGGGTTAC

CTACAATAACAGAGATGTCAGCAATGGATGTGAATTCAAACCCTCACAAGTTGTCAACCA

ACCAAGGGTAAATATCGGTGGTGATGACC

>CS61.61

TATTAGACTCCTAATTTAATATTCTTGTTTTATTATAATGCTGATAAGTCTTGTAAATAA

GGGTGAGAAGCACGAATAATTAGTTCATGAGATGTGTATAATTATTATCCTACACGACTT

ATCTTTGATATTTCACACAAGTCTTTCAATGTATAACAAAAACTTTTTAGATACATTTAG

ACTATAGAGCTAACAAGTTATATTTTGAACC-AAAAAACAAAGAGAGAGAGAGAGGAACA

TAGAGAAAAGAAGATATGAGAGTTTTTTTTTTTTTTT----CTAAAACAGAAAGAAACTC

ATTATATAATAAACAAATTACTTTGAGACAAATTAACGTATGTAATAAAACAAAATCAAA

GTGGTAATTAAAATTATTTAATGGTAAACAGTCTAATAGTTAAAATAAAAATGGAAATCA

CATAAATTTTGTAATTGGCCCATTAAAAACAACACTAAGCTTTTAATTTGATTTTGAAAT

TCAAAATAATTTTATTAAATCACAAAGTAAAAGGTTTACAAAGCCGATCATGACAGTGCA

TGTGGGAGGCAAATCGGCATATTTGCACTACAAAAGG-ACCCATGAAGTCTCTGAACATG

CACGCAACACTTTAATCTCTTATTAGTTACTTTGAAAGCTTATTTATATATATAGACACG

CGTAAAAACTTCTTAACCAAGATTTTTTT-ACGTGCTTCC-TTCGCGTTTAATTTGGACC

ATCAAACCGTGCTCAACAGATAAAGAAAAGGGTGCTTTTGATTCAAGATATTGGCC-GAA

AAACACAAGATAGATCCTT-CGATAGATTAAGCCACGCATGAAACGCGAATCCAAAGTGA

TGAAGAAGTGCAGATAGATATTCGTTCACCATATAGAGGAGAATATATCATTCCTACAAA

CAAAATTGATGATGTTTCTAGAATAGAAACGTTAGAATCGAATATTCATCTGTTCCGGGT

GGGGAAGGGCTACTGAAAAGCTGAACTTTTTTAAGAGTTCGAACTTCGAAAGTCAATCAA

TGATTCTTAATTGAGAGGGAAAAGCTATAAAGGACGAGAAGGGAAGGAAATGTTTATAGG

GAATGAATTTCTAATGCATGATGGGAATGACAGATATGAGGAATATATAAAGGTGATTCT

TATATACATTGACATGGATCGGAATCTATTAACTTAAAGTTATTGGGGTCGTGAAATTTA

TTTAATTTTTCTAATTCGTGTCACATGTCACGACAATTAGTGGGG-AACATTTATGTATA

TAGAAATTTCAGAAATTTCTAGCAGCGTGATAAATATAAAGATTTTGGCACAATAAGTTT

TTGGATCATAATGAGTTT-CTATTTAATCAA-GGCA-CGTAA-TCTATTTTATTATTTAG

-TAGGG-AAACTGAAAACTTAGGGTTGCTATTTGTAGCTCCCACCCTTCTTAGTTCTTAC

TCTTTTCAATATTTTTTAAAAGTTTTA-TATAATACCT-AAATT-GCCCTCCTCCT----

----------------------------------------CACCCCATTGCTCTTCCGCT

GCTCCTCCATGTG-AGTTCTTGTCTTTTTTTTT-CTCTGGTAGCGTTTTGCTGCTCCTTT

TTTCACTCAAGTGTTGCCAATTAA-TTGACAAAAAATGGTTTCTGTTTCATATAGAAACT

ATGTTTTTGTTGTGTAGTCATACATTACGGAATCTAGTTT-CCATTAAATAAGTAAC--G

TGAAAAAAAA--TAAAAGGTGAAATATATATTGTTGGAAAAGAAGCTATGAGGTGCAAGA

ACCGATCACATGGAGAAGGCAATGAAAGACAAGGAGGAGCAATGGAAGA-----------

---------GAGAAAATGAGAAGATGGAAGGGATGTGGAAATGTTTGAAAAAAACGAGGT

GATCAGTTTTAAAATACGAATTTAGTATTTTCTTTTTAAGAAAATTCTTTCG-AAAGTCG

TGTTTTAAAACATGACTTTTATT-ATTTGAAGTCG---TGTTCTAAAACATGACTTA--T

TCATATCCTT-AATATTTTT---------AAAATTTATCCATTTGTAATATTTTTTAAAA

ATTGACCCATATATGTAAAATACCCGTCAAGATCTCTTTATTATTTTGAAAGCGAAAGCA

TATCACTTCAAACACAATGGAATCGAGGCTATTGACTAAGTATAAACAGAGAAGACTTCA

TATCGGGGTTCATAATTCATAACAAAGCAAACGAGTATATAAGAAAGCATAAGCCAAATT

TTGAGTAAACTAGTGTGCACACTATCCCATGCCTAGTGGAAGTAGGGATCCTCTCGTTGT

TGGGGGAGTAATTGGGGATGTATTGGATCCTTTTGAATATTCTATTCCTATGAGGGTTAC

CTACAATAACAGAGATGTCAGCAATGGATGTGAATTCAAACCCTCACAAGTTGTCAACCA

ACCAAGGGTAAATATCGGTGGTGATGACC

>CS21.21

TATTAGACTCCTAATTTGATATTCTTGTTTTATTATAATGCTGATAAGTCTTGTAAATAA

GGGTGAGAAGCACGAATAATTAGTTCATGAGATGTGTATAATTATTATCCTACACGACTT

ATCTTTGATATTTCACACAAGTCTTTCAATGTATAACAAAAATTTTTTAGATACATTTAG

ACTATAGAACTAACAAGTTATATTTTGAACC-AAAAAACAAAGAGAGAGAG----GAACA

TAGAGAAAAGAAGATATGAGAGTTTTTTTTTTT--------CTAAAACAGAAAGAAACTC

ATTATATAATAAACAAATTACTTTGAGACAAATTAACGTATGTAATAAAACAAAATCAAA

GTGGTGATTAAAATTATTTAATGGTAAACAGTCTAATAGTTAAAATAAAAATGGAAATCA

CATAAATTTTGTAATTGGCCCATTAAAAACAACACAAAGCTTTTAATTTGATTTTGAAAT

TCAAAATAATTTTATTAAATCACAAAGTAAAAGGTTTACAAAGCCGATCATGACAGTGCA

TGTGGGAGGCAAATCGGCATATTTGCACTACAAAAGG-ACCCATGAAGTCTCTGAACATG

CACGCAACACTTTAATCTCTTATTAGTTACTTTGAAAGCTTATTTATATATATAGACACG

CGTAAAAACTTCTTAACCAAGATTTTTTT-ACGTGCTTCC-TTCGCGTTTAATTTGGACC

ATCAAACCGTGCTCAACAGATAAAGAAAAGGGTGCTTTTGATTCAAGATATTGGCC-GAA

AAACACAAGATAGATCCTT-CGATAGATTAAGCCACGCATGAAACGCGAATCCAAAGTGA

TGAAGAAGTGCAGATAGATATTCGTTCACCATATAGAGGAGAATATATCATTCCTACAAA

CAAAATTGATGATGTTTCTAGAATAGAAACGTTAGAATCGAATATTCATCTGTTCCGGGT

GGGGAAGGGCTACTGAAAAGCTGAACTTTTTTAAGAGTTCGAACTTCGAAAGTCAATCAA

TGATTCTTAATTGAGAGGGAAAAGCTATAAAGGACGAGAAGGGAAGGAAATGTTTATAGG

GAATGAATTTCTAATGCATGATGGGAATGACAGATATGAGGAATATATAAAGGTGATTCT

TATATACATTGACATGGATCGGAATCTATTAACTTAAAGTTATTGGGGTCGTGAAATTTA

TTTAATTTTTCTAATTCGTGTCACATGTCACGACAATTAGTGGGG-AACATTTATGTATA

TAGAAATTTCAGAAATTTCTAGCAGCGTGATAAATATAAAGATTTTGGCACAATAAGTTT

TTGGATCATAATGAGTTT-CTATTTAATCAA-GGCA-TGTAA-TCTATTTTATTATTTAG

-TAGGG-AAACTGAAAACTTAGGGTTGCTATTTGTAGCTCCCACCCTTCTTAGTTCTTAC

TCTTTTCAATATTTTTTATAAGTTTTA-TATAATACCT-AAATT-GCCCTCCTCCTCA-C

CCCCCCTGCTAATCTTCTTCCTCCTCACACATTGCTTCT-CACCCCATTGCTCTTCCGTT

GCTCCTCCATGTG-AGTTCTAGTCTTTTTTTTTT-TCTGGTAGCGTTTTGCTGCTCCTTT

TTTCACTCAAGTGTTGCCAATTAA-TTGACAAAAAATGGTTTCTGTTTCATATAGAAACT

ATGTTTTTGTTGTGTAGTCATACATTACGGAATCTAGTTT-CCATTAAATAAGTAACATG

TGAAAAAAAA--TAAAAGGTGAAATATATATTGTTGGAAAAGAAGCTATGAGGTGCAAGA

ACCGATCACATGGAGAAGGCAATGAAAGACAAGGAGGAGCAATGAAAGATAAGGAGGAGC

AATGAAAGAGAGAAAATGAGAAGATGGAAGGGATGTGAAAATGTTTGAAAAAGACGAGGT

GATCAGTTTTGAAATACGAATTTAGTATTTTCTTTTTAAGAAAATTCTTTCG-AAAGTCG

TGTTTTAAAACATGACTTTTATT-ATTTGAAGTCG---TGTTCTAAAACATGGCTTA--T

TCATATCCTT-AATATTTTT---------AAAATTTATCCATTTGTAATATTTTTTAAAA

ATTGACCCATATATGTAAAATACCCGTCAAGATCTCTTTATTATTTTGAAAGCGAAAGCA

TATCACTTCAAACACAATGGAATCGAGGCTATTGACTAAGTATAAATAGAGAAGACTTCA

TATCGGGGCTCATAATTCATAACAAAGCAAACGAGTATATAAGAAAGCATAAGCCAAATT

TTGAGTAAACTAGTGTGCACACTATCCCATGCCTAGTGGAAGTAGGGATCCTCTCGTTGT

TGGGGGAGTAATTGGGGATGTATTGGATCCTTTTGAATATTCTATTCCTATGAGGGTTAC

CTACAATAACAGAGATGTCAGCAATGGATGTGAATTCAAACCCTCACAAGTTGTCAACCA

ACCAAGGGTAAATATCGGTGGTGATGACC

>CS59.25

TATTAGACTCCTAATTTGATATTCTTGTTTTATTATAATGCTGATAAGTCTTGTAAATAA

GGGTGAGAAGCACGAATAATTAGTTCATGAGATGTGTATAATTATTATCCTACACGACTT

ATCTTTGATATTTCACACAAGTCTTTCAATGTATAACAAAAATTTTTTAGATACATTTAG

ACTATAGAACTAACAAGTTATATTTTGAACC-AAAAAACAAAGAGAGAGAG----GAACA

TAGAGAAAAGAAGATATGAGAGTTTTTTTTTTTTTTT----CTAAAACAGAAAGAAACTC

ATTATATAATAAACAAATTAATTTGAGACAAATTAACGTATGTAATAAAACAAAATCAAA

GTGGTAATTAAAATTATTTAATGGTAAACAGTCTAATAGTTAAAATAAAAATGGAAATCA

CATAAATTTTGTAATTGGCCCATTAAAAACAACACTAAGCTTTTAATTTGATTTTGAAAT

TCAAAATAATTTTATTAAGTCACAAAGTAAAAGGTTTACAAAGCCGATCAAGACAGTGCA

TGTGGGAGGCAAATCGGCATATTTGCACTACAAAAGG-ACCCATGAAGTCTCTGAACATG

CACGCAACACTTTAATCTCTTATTAGTTACTTTGAAAGCTTATTTATATATATAGACACG

CGTAAAAACTTCTTAACCAAGATTTTTTT-ACGTGCTTCC-TTCGCGTTTAATTTGGACC

ATCAAACCGTGCTCAACAGATAAAGAAAAGGGTGCTTTTGATTCAAGATATTGGCC-GAA

AAACACAAGATAGATCCTT-CGATAGATTAAGCCACGCATGAAACGCGAATCCAAAGTGA

TGAAGAAGTGCAGATAGATATTCGTTCACCATATAGAGGAGAATATATCATTCCTACAAA

CAAAATTGATGATGTTTCTAGAATAGAAACGTTAGAATCGAATATTCATCTGTTCCGGGT

GGGGAAGGGCTACTGAAAAGCTGAACTTTTTTAAGAGTTCGAACTTCGAAAGTCAATCAA

TGATTCTTAATTGAGAGGGAAAAGCTATAAAGGACGAGAAGGGAAGGAAATGTTTATAGG

GAATGAATTTCTAATGCATGATGGGAATGACAGATATGAGGAATATATAAAGGTGATTCT

TATATACATTGACATGGATCGGAATCTATTAACTTAAAGTTATTGGGGTCGTGAAATTTA

TTTAATTTTTCTAATTCGTGTCACATGTCACGACAATTAGTGGGG-AACATTTATGTATA

TAGAAATTTCAGAAATTTCTAGCAGCGTGATAAATATAAAGATTTTGGCACAATAAGTTT

TTGGATCATAATGAGTTT-CTATTTAATCAA-GGCA-TGTAA-TCTATTTTATTATTTAG

-TAGGG-AAACTGAAAACTTAGGGTTGCTATTTGTAGCTCCCACCCTTCTTAGTTCTTAC

TCTTTTCAATATTTTTTATAAGTTTTA-TATAATACCT-AAATT-GCCCTCCTCCTCACC

CCCCCCTGCTAATCTTCTTCCTCCTCACACATTGCTTCT-CACCCCATTGCTCTTCCGTT

GCTCCTCCATGTG-AGTTCTAGTCTTTTTTTTT-CTCTGGTAGCGTTTTGCTGCTCCTTT

TTTCACTCAAGTGTTGCCAATTAA-TTGACAAAAAATGGTTTCTGTTTCATATAGAAACT

ATGTTTTTGTTGTGTAGTCATACATTACGGAATCTAGTTT-CCATTAAATAAGTAACATG

TGAAAAAAAA--TAAAAGGTGAAATATATATTGTTGGAAAAGAAGCTATGAGGTGCAAGA

ACCGATCACATGGAGAAGGCAATGAAAGACAAGGAGGAGCAATGGAAGA-----------

---------GAGAAAATGAGAAGATGGAAGGGATGTGAAAATGTTTGAAAAAGACGAGGT

GATCAGTTTTGAAATACGAATTTAGTATTTTCTTTTTAAGAAAATTCTTTCG-AAAGTCG

TGTTTTAAAACATGACTTTTATT-ATTTGAAGTC----TGTTCTAAAACATGACTTA--T

TCATATCCTT-AATATTTTT---------AAAATTTATCCATTTGTAATATTTTTTAAAA

ATTGACCCATATATGTAAAATACCCGTCAAGATCTCTTTATTATTTTGAAAGCGAAAGCA

TATCACTTCAAACACAATGGAATCGAGGCTATTGACTAAGTATAAATAGAGAAGACTTCA

TATCGGGGTTCATAATTCATAACAAAGCAAACGAGTATATAAG----------CCAAATT

TTGAGTAAACTAGTGTGCACACTATCCCATGCCTAGTGGAAGTAGGGATCCTCTCGTTGT

TGGGGGAGTAATTGGGGATGTATTGGATCCTTTTGAATATTCTATTCCTATGAGGGTTAC

CTACAATAACAGAGATGTCAGCAATGGATGTGAATTCAAACCCTCACAAGTTGTCAACCA

ACCAAGGGTAAATATCGGTGGTGATGACC

>CS06.06

TATTAGACTCCTAATTTAATATTCTTGTTTTATTATAATGCTGATAAGTCTTGTAAATAA

GGGTGAGAAGCACGAATAATTAGTTCATGAGATGTGTATAATTATTATCCTACACGACTT

ATCTTTGATATTTCACACAAGTCTTTCAATGTATAACAAAAACTTTTTAGATACATTTAG

ACTATAGAACTAACAAGTTATATTTTGAACC-AAAAAACAAAGAGAGAGAGAGAGGAACA

TAGAGAAAAGAAGATATGAGAGTTTTTTTTTTTTTTTT---CTAAAACAGAAAGAAACTC

ATTATATAATAAACAAATTACTTTGAGACAAATTAACGTATGTAATAAAACAAAATCAAA

GTGGTAATTAAAATTATTTAATGGTAAACAGTCTAATAGTTAAAATAAAAATAGAAATCA

CATAAATTTTGTAATTGGCCCATTAAAAACAACACTAAGCTTTTAATTTGATTTTGAAAT

TCAAAATAATTTTATTAAATCACAAAGTAAAAGGTTTACAAAGCCGATCATGACAGTGCA

TGTGGGAGGCAAATCGGCATATTTGCACTACAAAAGG-ACCCATGAAGTCTCTGAACATG

CACGCAACACTTTAATCTCTTATTAGTTACTTTGAAAGCTTATTTATATATATAGACACG

CGTAAAAACTTCTTAACCAAGATTTTTTT-ACGTGCTTCC-TTCGCGTTTAATTTGGACC

ATCAAACCGTGCTCAACAGATAAAGAAAAGGGTGCTTTTGATTCAAGATATTGGCC-GAA

AAACACAAGATAGATCCTT-CGATAGATTAAGCCACGCATGAAACGCGAATCCAAAGTGA

TGAAGAAGTGCAGATAGATATTCGTTCACCATATAGAGGAGAATATATCATTCCTACAAA

CAAAATTGATGATGTTTCTAGAATAGAAACGTTAGAATCGAATATTCATCTGTTCCGGGT

GGGGGAGGGCTACTGAAAAGCTGAACTTTTTTAAGAGTTCGAACTTCGAAAGTCAATCAA

TGATTCTTAATTGAGAGGGAAAAGCTATAAAGGACGAGAAGGGAAGGAAATGTTTATAGG

GAATGAATTTCTAATGCATGATGGGAATGACAGATATGAGGAATATATAAAGGTGATTCT

TATATACATTGACATGGATCGGAATCTATTAACTTAAAGTTATTGGGGTCGTGAAATTTA

TTTAATTTTTCTAGTTCGTGTCACATGTCACGACAATTAGTGGGG-AACATTTATGTATA

TAGAAATTTCAGAAATTTCTAGCAGCGTGATAAATATAAAGATTTTGGCACAATAAGTTT

TTGGATCATAATGAGTTT-CTATTTAATCAA-GGCA-TGTAA-TCTATTTTATTATTTAG

-TAGGG-AAACTGAAAACTTAGGGTTGCTATTTGTAGCTCCCACCCTTCTTAGTTCTTAC

TCTTTTCAATATTTTTTAAAAGTTTTA-TATAATACCT-AAATT-GCCCTCCTCCT----

----------------------------------------CACCCCATTGCTCTTCCGTT

GCTCCTCCATGTG-AGTTCTTGTCTTTTTTTTT-CTCTGGTAGCGTTTTGCTGCTCCTTT

TTTCACTCAAGTGTTGCCAATTAA-TTGACAAAAAATAGTTTCTGTTTCATATAGAAACT

ATGTTTTTGTTGTGTAGTCATACATTACGGAATCTAGTTT-CCATTAAATAAGTAAC--G

TGAAAAAAAA--TAAAAGGTGAAATATATATTGTTGGAAAAGAAGCTATGAGGTGCAAGA

ACCGATCACATGGAGAAGGCAATGAAAGACAAGGAGGAGCAATGGAAGA-----------

---------GAGAAAATGAGAAGATGGAAGGGATGTGAAAATGTTTGAAAAAAACGAGGT

GATCAGTTTTAAAATACGAATTTAGTATTTTCTTTTTAAGAAAATTCTTTCG-AAAGTCG

TGTTTTAAAACATGACTTTTATT-ATTTGAAGTCG---TGTTCTAAAACATGACTTA--T

TCATATCCTT-AATATTTTT---------AAAATTTATCCATTTGTAATATTTTTTAAAA

ATTGACCCATATATGTAAAATACCCGTCAAGATCTCTTTATTATTTTGAAAGCGAAAGCA

TATCACTTCAAACACAATGGAATCGAGGCTACTGACTAAGTATAAATAGAGAAGACTTCA

TATCGGGGTTCATAATTCATAACAAAGCAAACGAGTATATAAGAAAGCATAAGCCAAATT

TTGAGTAAACTAGTGTGCACACTATCCCATGCCTAGTGGAAGTAGGGATCCTCTCGTTGT

TGGGGGAGTAATTGGGGATGTATTGGATCCTTTTGAATATTCTATTCCTATGAGGGTTAC

CTACAATAACAGAGATGTCAGCAATGGATGTGAATTCAAACCCTCACAAGTTGTCAACCA

ACCAAGGGTAAATATCGGTGGTGATGACC

>CS01.05

TATTAGACTCCTAATTTAATATTCTTGTTTTATTATAATGCTGATAAGTCTTGTAAATAA

GGGTGAGAAGCACGAATAATTAGTTCATGAGATGTGTATAATTATTATCCTACACGACTT

ATCTTTGATATTTCACACAAGTCTTTCAATGTATAACAAAAACTTTTTAGATACATTTAG

ACTATAGAACTAACAAGTTATATTTTGAACC-AAAAAACAAAGAGAGAGAGAGAGGAACA

TAGAGAAAAGAAGATATGAGAGTTTTTTTTTTTTTTTTT--CTAAAACAGAAAGAAACTC

ATTATATAATAAACAAATTACTTTGAGACAAATTAACGTATGTAATAAAACAAAATCAAA

GTGGTAATTAAAATTATTTAATGGTAAACAGTCTAATAGTTAAAATAAAAATGGAAATCA

CATAAATTTTGTAATTGGCCCATTAAAAACAACACTAAGCTTTTAATTTGATTTTGAAAT

TCAAAATAATTTTATTAAATCACAAAGTAAAAGGTTTACAAAGCCGATCATGACAGTGCA

TGTGGGAGGCAAATCGGCATATTTGCACTACAAAAGG-ACCCATGAAGTCTCTGAACATG

CACGCAACACTTTAATCTCTTATTAGTTACTTTGAAAGCTTATTTATATATATAGACACG

CGTAAAAACTTCTTAACCAAGATTTTTTT-ACGTGCTTCC-TTCGCGTTTAATTTGGACC

ATCAAACCGTGCTCAACAGATAAAGAAAAGGGTGCTTTTGATTCAAGATATTGGCC-GAA

AAACACAAGATAGATCCTT-CGATAGATTAAGCCACGCATGAAACGCGAATCCAAAGTGA

TGAAGAAGTGCAGATAGATATTCGTTCACCATATAGAGGAGAATATATCATTCCTACAAA

CAAAATTGATGATGTTTCTAGAATAGAAACGTTAGAATCGAATATTCATCTGTTCCGGGT

GGGGAAGGGCTACTGAAAAGCTGAACTTTTTTAAGAGTTCGAACTTCGAAAGTCAATCAA

TGATTCTTAATTGAGAGGGAAAAGCTATAAAGGACGAGAAGGGAAGGAAATGTTTATAGG

GAATGAATTTCTAATGCATGATGGGAATGACAGATATGAGGAATATATAAAGGTGATTCT

TATATACATTGACATGGATCGGAATCTATTAACTTAAAGTTATTGGGGTCGTGAAATTTA

TTTAATTTTTCTAATTCGTGTCACATGTCACGACAATTAGTGGGG-AACATTTATGTATA

TAGAAATTTCAGAAATTTCTAGCAGCGTGATAAATATAAAGATTTTGGCACAATAAGTTT

TTGGATCATAATGAGTTT-CTATTTAATCAA-GGCA-TGTAA-TCTATTTTATTATTTAG

-TAGGG-AAACTGAAAACTTAGGGTTGCTATTTGTAGCTCCCACCCTTCTTAGTTCTTAC

TCTTTTCAATATTTTTTAAAAGTTTTA-TATAATACCT-AAATT-GCCCTCCTCCT----

----------------------------------------CACCCCATTGCTCTTCCGTT

GCTCCTCCATGTG-AGTTCTTGTCTTTTTTTTT-CTCTGGTAGCGTTTTGCTGCTCCTTT

TTTCACTCAAGTGTTGCCAATTAA-TTGACAAAAAATGGTTTCTGTTTCATATAGAAACT

ATGTTTTGGTTGTGTAGTCATACATTACGGAATCTAGTTT-CCATTAAATAAGTAAC--G

TGAAAAAAAA--TAAAAGGTGAAATATATATTGTTGGAAAAGAAGCTATGAGGTGCAAGA

ACCGATCACATGGAGAAGGCAATGAAAGACAAGGAGGAGCAATGGAAGA-----------

---------GAGAAAATGAGAAGATGGAAGGGATGTGAAAATGTTTGAAAAAAACGAGGT

GATCAGTTTTAAAATACGAATTTAGTATTTTCTTTTTAAGAAAATTCTTTCG-AAAGTCG

TGTTTTAAAACATGACTTTTATT-ATTTGAAGTCG---TGTTCTAAAACATGACTTA--T

TCATATCCTT-AATATTTTT---------AAAATTTATCCATTTGTAATATTTTTTAAAA

ATTGACCCATATATGTAAAATACCCGTCAAGATCTCTTTATTATTTTGAAAGCGAAAGCA

TATCACTTCAAACACAATGGAATCGAGGCTATTGACTAAGTATAAATAGAGAAGACTTCA

TATCGGGGTTCATAATTCATAACAAAGCAAACGAGTATATAAGAAAGCATAAGCCAAATT

TTGAGTAAACTAGTGTGCACACTATCCCATGCCTAGTGGAAGTAGGGATCCTCTCGTTGT

TGGGGGAGTAATTGGGGATGTATTGGATCCTTTTGAATATTCTATTCCTATGAGGGTTAC

CTACAATAACAGAGATGTCAGCAATGGATGTGAATTCAAACCCTCACAAGTTGTCAACCA

ACCAAGGGTAAATATCGGTGGTGATGACC

>CS07.02

TATTAGACTCCTAATTTAATATTCTTGTTTTATTATAATGCTGATAAGTCTTGTAAATAA

GGGTGAGAAGCACGAATAATTAGTTCATGAGATGTGTATAATTATTATCCTACACGACTT

ACCTTTGATATTTCACACAAGTCTTTCAATGTATAACAAAAACTTTTTAGATACATTTAG

ACTATAGAACTAACAAGTTATATTTTGAACC-AAAAAACAAAGAGAGAGAGAGGGGAACA

TAGAGAAAAGAAGATATGAGAGTTTTTTTTTTTTTTTTT--CTAAAACAGAAAGAAACTC

ATTATATAATAAACAAATTACTTTGAGACAAATTAACGTATGTAATAAAACAAAATCAAA

GTGGTAATTAAAATTATTTAATGGTAAACAGTCTAATAGTTAAAATAAAAATGGAAATCA

CATAAATTTTGTAATTGGCCCATTAAAAACAACACTAAGCTTTTAATTTGATTTTGAAAT

TCAAAATAATTTTATTAAATCACAAAGTAAAAGGTTTACAAAGCCGATCATGACAGTGCA

TGTGGGAGGCAAATCGGCATATTTGCACTACAAAAGG-ACCCATGAAGTCTCTGAACATG

CACGCAACACTTTAATCTCTTATTAGTTACTTTGAAAGCTTATTTATATATATAGACACG

CGTAAAAACTTCTTAACCAAGATTTTTTT-ACGTGCTTCC-TTCGCGTTTAATTTGGACC

ATCAAGCCGTGCTCAACAGATAAAGAAAAGGGTGCTTTTGATTCAAGATATTGGCC-GAA

AAACACAAGATAGATCCTT-CGATAGATTAAGCCACGCATGAAACGCGAATCCAAAGTGA

TGAAGAAGTGCAGATAGATATTCGTTCACCATATAGAGGAGAATATATCATTCCTACAAA

CAAAATTGATGATGTTTCTAGAATAGAAACGTTAGAATCGAATATTCATCTGTTCCGGGT

GGGGAAGGGCTACTGAAAAGCTGAACTTTTTTAAGAGTTCGAACTTCGAAAGTCAATCAA

TGATTCTTAATTGAGAGGGAAAAGCTATAAAGGACGAGAAGGGAAGGAAATGTTTATAGG

GAATGAATTTCTAATGCATGATGGGAATGACAGATATGAGGAATATATAAAGGTGATTCT

TATATACATTGACATGGATCGGAATCTATTAACTTAAAGTTATTGGGGTCGTGAAATTTA

TTTAATTTTTCTAATTCGTGTCACATGTCACGACAATTAGTGGGG-AACATTTATGTATA

TAGAAATTTCAGAAATTTCTGGCAGCGTGATAAATATAAAGATTTTGGCACAATAAGTTT

TTGGATCATAATGAGTTT-CTATTTAATCAA-GGCA-TGTAA-TCTATTTTATTATTTAG

-TAGGG-AAACTGAAAACTTAGGGTTGCTATTTGTAGCTCCCACCCTTCTTAGTTCTTAC

TCTTTTCAATATTTTTTAAAAGTTTTA-TATAATACCT-AAATT-GCCCTCCTCCT----

----------------------------------------CACCCCATTGCTCTTCCGTT

GCTCCTCCATGTG-AGTTCTTGTCTTTTTTTTT-CTCTGGTAGCGTTTTGCTGCTCCTTT

TTTCACTCAAGTGTTGCCAATTAA-TTGACAAAAAATGGTTTCTGTTTCATATAGAAACT

ATGTTTTTGTTGTGTAGTCATACATTACGGAATCTAGCTT-CCATTAAATAAGTAAC--G

TGAAAAAAAA--TAAAAGGTGAGATATATATTGTTGGAAAAGAAGCTATGAGGTGCAAGA

ACCGATCACATGGAGAAGGCAATGAAAGACAAGGAGGAGCGATGGAAGA-----------

---------GAGAAAATGAGAAGATGGAAGGGATGTGAAAATGTTTGAAAAAAACGAGGT

GATCAGTTTTAAAATACGAATTTAGTATTTTCTTTTTAAGAAAATTCTTTCG-AAAGTCG

TGTTTTAAAACATGACTTTTATT-ATTTGAAGTCG---TGTTCTAAAACATGACTTA--T

TCATATCCTT-AATATTTTT---------AAAATTTATCCATTTGTAATATTTTTTAAAA

ATTGACCCATATATGTAAAATACCCGTCAAGATCTCTTTATTATTTTGAAAGCGAAAGCA

TATCACTTCAAACACAATGGAATCGAGGCTATTGACTAAGTATAAATAGAGAAGACTTCA

TATCGGGGTTCATAATTCATAACAAAGCAAACGAGTATATAAGAAAGCATAAGCCAAATT

TTGAGTAAACTAGTGTGCACACTATCCCATGCCTAGTGGAAGTAGGGATCCTCTCGTTGT

TGGGGGAGTAATTGGGGATGTATTGGATCCTTTTAAATATTCTATTCCTATGAGGGTTAC

CTACAATAACAGAGATGTCAGCAATGGATGTGAATTCAAACCCTCACAAGTTGTCAACCA

ACCAAGGGTAAATATCGGTGGTGATGACC

>CS26.14

TATTAGACTCCTAATTTAATATTCTTGTTTTATTATAATGCTGATAAGTCTTGTAAATAA

GGGTGAGAAGCACGAATAATTAGTTCATGAGATGTGTATAATTATTATCCTACACGACTT

ATCTTTGATATTTCACACAAGTCTTTCAACGTATAACAAAAACTTTTTAGATACATTTAG

ACTATAGAACTAACAAGTTATATTTTGAACC-AAAAAACAAAGAGAGAGAGAGAGGAACA

TAGAGAAAAGAAGATATGAGAGTTTTTTTTTTTTTTTTTT-CTAAAACAGAAAGAAACTC

ATTATATAATAAACAAATTACTTTGAGACAAATTAACGTATGTAATAAAACAAAATCAAA

GTGGTAATTAAAATTATTTAATGGTAAACAGTCTAATAGTTAAAATAAAAATGGAAATCA

CATAAATTTTGTAATTGGCCCATTAAAAACAGCACTAAGCTTTTAATTTGATTTTGAAAT

TCAAAATAATTTTATTAAATCACAAAGTAAAAGGTTTACAAAGCCGATCATGACAGTGCA

TGTGGGAGGCAAATCGGCATATTTGCACTACAAAAGG-ACCCATGAAGTCTCTGAACATG

CACGCAACACTTTAATCTCTTATTAGTTACTTTGAAAGCTTATTTATATATATAGACACG

CGTAAAAACTTCTTAACCAAGATTTTTTT-ACGTGCTTCC-TTCGCGTTTAATTTGGACC

ATCAAACCGTGCTCAACAGATAAAGAAAAGGGTGCTTTTGATTCAAGATATTGGCC-GAA

AAACACAAGATAGATCCTT-CGATAGATTAAGCCACGCATGAAACGCGAATCCAAAGTGA

TGAAGAAGTGCAGATGGATATTCGTTCACCATATAGAGGAGAATATATCATTCCTACAAA

CAAAATTGATGATGTTTCTAGAATAGAAACGTTAGAATCGAATATTCATCTGTTCCGGGT

GGGGAAGGGCTACTGAAAAGCTGAACTTTTTTAAGAGTTCGAACTTCGAAAGTCAATCAA

TGATTCTTAATTGAGAGGGAAAAGCTATAAAGGACGAGAAGGGAAGGAAATGTTTATAGG

GAATGAACTTCTAATGCATGATGGGAATGACAGATATGAGGAATATATAAAGGTGATTCT

TATATACATTGACATGGATCGGAATCTATTAACTTAAAGTTATTGGGGTCGTGAAATTTA

TTTAATTTTTCTAATTCGTGTCACATGTCACGACAATTAGTGGGG-AACATTTATGTATA

TAGAAATTTCAGAAATTTCTAGCAGCGTGATAAATATAAAGACTTTGGCACAATAAGTTT

TTGGATCATAATGAGTTT-CTATTTAATCAA-GGCA-TGTAA-TCTATTTTATTATTTAG

-TAGGG-AAACTGAAAACTTAGGGTTGCTATTTGTAGCTCCCACCCTTCTTAGTTCTTAC

TCTTTTCAATATTTTTTAAAAGTTTTA-TATAATACCT-AAATT-GCCCTCCTCCT----

----------------------------------------CACCCCATTGCTCTTCCGTT

GCTCCTCCATGTG-AGTTCTTGTCTTCTTTTTT-CTCTGGTAGCGTTTTGCTGCTCCTTT

TTTCACTCGAGTGTTGCCAATTAA-TTGACAAAAAATGGTTTCTGTTTCATATAGAAACT

ATGTTTTTGTTGTGTAGTCATACATTACGGAATCTAGTTT-CCATTAAATAAGTAAC--G

TGAAAAAAAA--TAAAAGGTGAAATATATATTGTTGGAAAAGAAGCTATGAGGTGCAAGA

ACCGATCACATGGAGAAGGCAATGAAAGACAAGGAGGAGCAATGGAAGA-----------

---------GAGAAAATGAGAAGATGGAAGGGATGTGAAAATGTTTGAAAAAAACGAGGT

GATCAGTTTTAAAATACGAATTTAGTATTTTCTTTTTAAGAAAATTCTTTCG-AAAGTCG

TGTTTTAAAACATGACTTTTATT-ATTTGAAGTCG---TGTTCTAAAACATGACTTA--T

TCATATCCTT-AATATTTTT---------AAAATTTATCCATTTGTAATATTTTTTAAAA

ATTGACCCACATATGTAAAATACCCGTCAAGATCTCTTTATTATTTTGAAAGCGAAAGCA

TATCGCTTCAAACACAATGGAATCGAGGCTATTGACTAAGTATAAATAGAGAAGACTTCA

TATCGGGGTTCATAATTCATAACAAAGCAAACGAGTATATAAGAAAGCATAAGCCAAATT

TTGAGTAAACTAGTGTGCACACTATCCCATGCCTAGTGGAAGTAGGGATCCTCTCGTTGT

CGGGGGAGTAATTGGGGATGTATTGGATCCTTTTGAATATTCTATTCCTATGAGGGTTAC

CTACAATAACAGAGATGTCAGCAATGGATGTGAATTCAAACCCTCACAAGTTGTCAACCA

ACCAAGGGTAAATATCGGTGGTGATGACC

>CS10.13

TATTAGACTCCTAATTTAATATTCTTGTTTTATTATAATGCTGATAAGTCTTGTAAATAA

GGGTGAGAAGCACGAATAATTAGTTCATGAGATGTGTATAATTATTATCCTACACGACTT

ATCTTTGATATTTCACACAAGTCTTTCAATGTATAACAAAAACTTTTTAGATACATTTAG

ACTATAGAACTAACAAGTTATATTTTGAACC-AAAAAACAAAGAGAGAGAGAGAGGAACA

TAGAGAAAAGAAGATATGAGAGTTTTTTTTTTTTTTTT---CTAAAACAGAAAGAAACTC

ATTATATAATAAACAAATTACTTTGAGACAAATTAACGTATGTAATAAAACAAAATCAAA

GTGGTAATTAAAATTATTTAATGGTAAACAGTCTAATAGTTAAAATAAAAATGGAAATCA

CATAAATTCTGTAATTGGCCCATTAAAAACAACACTAAGCTTTTAATTTGATTTTGAAAT

TCAAAATAATTTTATTAAATCACAAAGTAAAAGGTTTACAAAGCCGATCATGACAGTGCA

TGTGGGAGGCAAATCGGCATATTTGCACTACAAAAGG-ACCCACGAAGTCTCTGAACATG

CACGCAACACTTTAATCTCTTATTAGTTACTTTGAAAGCTTATTTATATATATAGACACG

CGTAAAAACTTCTTAACCAAGATTTTTTT-ACGTGCTTCC-TTCGCGTTTAATTTGGACC

ATCAAACCGTGCTCAACAGATAAAGAAAAGGGTGCTTTTGATTCAAGATATTGGCC-GAA

AAACACAAGATAGATCCTT-CGATAGATTAAGCCACGCATGAAACGCGAATCCAAAGTGA

TGAAGAAGTGCAGATAGATATTCGTTCACCATATAGAGGAGAATATATCATTCCTACAAA

CAAAATTGATGATGTTTCTAGAATAGAAACGTTAGAATCGAATATTCATCTGTTCCGGGT

GGGGAAGGGCTACTGAAAAGCTGAACTTTTTTAAGAGTTCGAACTTCGAAAGTCAATCAA

TGATTCTTAATTGAGAGGGAAAAGCTATAAAGGACGAGAAGGGAAGGAAATGTTTATAGG

GAATGAATTTCTAATGCATGATGGGAATGACAGATATGAGGAATATATAAAGGTGATTCT

TATATACATTGACATGGATCGGAATCTATTAACTTAAAGTTATTGGGGTCGTGAAATTTA

TTTAATTTTTCTAATTCGTGTCACATGTCACGACAATTAGTGGGG-AACATTTATGTATA

TAGAAATTTCAGAAATTTCTAGCAGCGTGATAAATATAAAGATTTTGGCACAATAAGTTT

TTGGATCATAATGAGTTT-CTATTTAATCAA-GGCA-TGTAA-TCTATTTTATTATTTAG

-TAGGG-AAACTGAAAACTTAGGGTTGCTATTTGTAGCTCCCACCCTTCTTAGTTCTTAC

TCTTTTCAATATTTTTTAAAAGTTTTA-TATAATACCT-AAATT-GCCCTCCTCCT----

----------------------------------------CACCCCATTGCTCTTCCGTT

GCTCCTCCATGTG-AGTTCTTGTCTTTTTTTTT-CTCTGGTAGCGTTTTGCTGCTCCTTT

TTTCACTCAAGTGTTGCCAATTAA-TTGACAAAAAATGGTTTCTGTTTCATATAGAAACT

ATGTTTTTGTTGTGTAGTCATACATTACGGAATCTAGTTT-CCATTAAATAAGTAAC--G

TGAAAAAAAA--TAAAAGGTGAAATATATATTGTTGGAAAAGAAGCTATGAGGTGCAAGA

ACCGATCACATGGAGAAGGCAATGAAAGACAAGGAGGAGCAATGGAAGA-----------

---------GAGAAAATGAGAAGATGGAAGGGATGTGAAAATGTTTGAAAAAAACGAGGT

GATCAGTTTTAAAATACGAATTTAGTATTTTCTTTTTAAGAAAATTCTTTCG-AAAGTCG

TGTTTTAAAACATGACTTTTATT-ATTTGAAGTCG---TGTTCTAAAACATGACTTA--T

TCATATCCTT-AATATTTTT---------AAAATTTATCCATTTGTAATATTTTTTAAAA

ATTGACCCATATATGTAAAATACCCGTCAAGATCTCTTTATTATTTTGAAAGCGAAAGCA

TATCACTTCAAACACAATGGAATCGAGGCTATTGACTAAGTATAAATAGAGAAGACTTCA

TATCGGGGCTCATAATTCATAACAAAGCAAACGAGTATATAAGAAAGCATAAGCCAAATT

TTGAGTAAACTAGTGTGCACACTATCCCATGCCTAGTGGAAGTAGGGATCCTCTCGTTGT

TGGGGGAGTAATTGGGGATGTATTGGATCCTTTTGAATATTCTATTCCTATGAGGGTTAC

CTACAATAACAGAGATGTCAGCAATGGATGTGAATTCAAACCCTCACAAGTTGTCAACCA

ACCAAGGGTAAATATCGGTGGTGATGACC

>CS10.16

TATTAGACTCCTAATTTAATATTCTTGTTTTATTATAATGCTGATAAGTCTTGTAAATAA

GGGTGAGAAGCACGAATAATTAGTTCATGAGATGTGTATAATTATTATCCTACACGACTT

ATCTTTGATATTTCACACAAGTCTTTCAATGTATAACAAAAACTTTTTAGATACATTTAG

ACTATAGAACTAACAAGTTATATTTTGAACC-AAAAAACAAAGAGAGAGAGAGAGGAACA

TAGAGAAAAGAAGATATGAGAGTTTTTTTTTTTTTTTT---CTAAAACAGAAAGAAACTC

ATTATATAATAAACAAATTACTTTGAGACAAATTAACGTATGTAATAAAACAAAATCAAA

GTGGTAATTAAAATTATTTAATGGTAAACAGTCTAATAGTTAAAATAAAAATGGAAATCA

CATAAATTTTGTAATTGGCCCATTAAAAACAACACTAAGCTTTTAATTTGATTTTGAAAT

TCAAAATAATTTTATTAAATCACAAAGTAAAAGGTTTACAAAGCCGATCATGACAGTGCA

TGTGGGAGGCAAATCGGCATATTTGCACTACAAAAGG-GCCCATGAAGTCTCTGAACATG

CACGCAACACTTTAATCTCTTATTAGTTACTTTGAAAGCTTATTTATATATATAGACACG

CGTAAAAACTTCTTAACCAAGATTTTTTT-ACGTGCTTCC-TTCGCGTTTAATTTGGACC

ATCAAACCGTGCTCAACAGATAAAGAAAAGGGTGCTTTTGATTCAAGATATTGGCC-GAA

AAACACAAGATAGATCCTT-CGATAGATTAAGCCACGCATGAAACGCGAATCCAAAGTGA

TGAAGAAGTGCAGATAGATATTCGTTCACCATATAGAGGAGAATATATCATTCCTACAAA

CAAAATTGATGATGTTTCTAGAATAGAAACGTTAGAATCGAATATTCATCTGTTCCGGGT

GGGGAAGGGCTACTGAAAAGCTGAACTTTTTTAAGAGTTCGAACTTCGAAAGTCAATCAA

TGATTCTTAATTGAGAGGGAAAAGCTATAAAGGACGAGAAGGGAAGGAAATGTTTATAGG

GAATGAATTTCTAATGCATGATGGGAATGACAGATATGAGGAATATATAAAGGTGATTCT

TATATACATTGACATGGATCGGAATCTATTAACTTAAAGTTATTGGGGTCGTGAAATTTA

TTTAATTTTTCTAATTCGTGTCACATGTCACGACAATTAGTGGGG-AACATTTATGTATA

TAGAAATTTCAGAAATTTCTAGCAGCGTGATAAATATAAAGATTTTGGCACAATAAGTTT

TTGGATCATAATGAGTTT-CTATTTAATCAA-GGCA-TGTAA-TCTATTTTATTATTTAG

-TAGGG-AAACTGAAAACTTAGGGTTACTATTTGTAGCTCCCACCCTTCTTAGTTCTTAC

TCTTTTCAATATTTTTTAAAAGTTTTA-TATAATACCT-AAATT-GCCCTCCTCCT----

----------------------------------------CACCCCATTGCTCTTCCGTT

GCTCCTCCATGTG-AGTTCTTGTCTTTTTTTTT-CTCTGGTAGCGTTTTGCTGCTCCTTT

TTTCACTCAAGTGTTGCCAATTAA-TTGACAAAAAATGGTTTCTGTTTCATATAGAAACT

ATGTTTTTGTTGTGTAGTCATACATTACGGAATCTAGTTT-CCATTAAATAAGTAAC--G

TGAAAAAAAA--TAAAAGGTGAAATATATATTGTTGGAAAAGAAGCTATGAGGTGCAAGA

ACCGATCACATGGAGAAGGCAATGAAAGACAAGGAGGAGCAATGGAAGA-----------

---------GAGAAAATGAGAAGATGGAAGGGATGTGAAAATGTTTGAAAAAAACGAGGT

GATCAGTTTTAAAATACGAATTTAGTATTTTCTTTTTAAGAAAATTCTTTCG-AAAGTCG

TGTTTTAAAACATGACTTTTATT-ATTTGAAGTCG---TGTTCTAAAACATGACTTA--T

TCATATCCTT-AATATTTTT---------AAAATTTATCCATTTGTAATATTTTTTAAAA

ATTGACCCATATATGTAAAATACCCGTCAAGATCTCTTTATTATTTTGAAAGCGAAAGCA

TATCACTTCAAACACAATGGAATCGAGGCTATTGACTAAGTATAAATAGAGAAGACTTCA

TATCGGGGTTCATAATTCATAACAAAGCAAACGAGTATATAAGAAAGCATAAGCCAAATT

TTGAGTAAACTAGTGTGCACACTATCCCATGCCTAGTGGAAGTAGGGATCCTCTCGTTGT

TGGGGGAGTAATTGGGGATGTATTGGATCCTTTTGAATATTCTATTCCTATGAGGGTTAC

CTACAATAACAGAGATGTCAGCAATGGATGTGAATTCAAACCCTCACAAGTTGTCAACCA

ACCAAGGGTAAATATCGGTGGTGATGACC

>CS28.11

TATTAGACTCCTAATTTAATATTCTTGTTTTATTATAATGCTGATAAGTCTTGTAAATAA

GGGTGAGAAGCACGAATAATTAGTTCATGAGATGTGTATAATTATTATCCTACACGACTT

ATCTTTGATATTTCACACAAGTCTTTCAATGTATAACAAAAACTTTTTAGATACATTTAG

ACTATAGAACTAACAAGTTATATTTTGAACC-AAAAAACAAAGAGAGAGAGAGAGGAACA

TAGAGAAAAGAAGATATGAGAGTTTTTTTTTTTTTTTTT--CTAAAACAGAAAGAAACTC

ATTATATAATAAACAAATTACTTTGAGACAAATTAACGTATGTAATAAAACAAAATCAAA

GTGGTAATTAAAATTATTTAATGGTAAACAGTCTGATAGTTAAAATAAAAATGGAAATCA

CATAAATTTTGTAATTGGCCCATTAAAAACAACACTAAGCTTTTAATTTGATTTTGAAAT

TCAAAATAATTTTATTAAATCACAAAGTAAAAGGTTTACAAAGCCGATCATGACAGTGCA

TGTGGGAGGCAAATCGGCATATTTGCACTACAAAAGG-ACCCATGAAGTCTCTGAACATG

CACGCAACACTTTAATCTCTTATTAGTTACTTTGAAAGCTTATTTATATATATAGACACG

CGTAAAAACTTCTTAACCAAGATTTTTTT-ACGTGCTTCC-TTCGCGTTTAATTTGGACC

ATCAAACCGTGCTCAACAGATAAAGAAAAGGGTGCTTTTGATTCAAGATATTGGCC-GAA

AAACACAAGATAGATCCTT-CGATAGATTAAGCCACGCATGAAACGCGAATCCAAAGTGA

TGAAGAAGTGCAGATAGATATTCGTTCACCATATAGAGGAGAATATATCATTCCTACAAA

CAAAATTGATGATGTTTCTAGAATAGAAACGTTAGAATCGAATATTCATCTGTTCCGGGT

GGGGAAGGGCTACTGAAAAGCTGAACTTTTTTAAGAGTTCGAACTTCGAAAGTCAATCAA

TGATTCTTAATTGAGAGGGAAAAGCTATAAAGGACGAGAAGGGAAGGGAATGTTTATAGG

GAATGAATTTCTAATGCATGATGGGAATGACAGATATGAGGAATATATAAAGGTGATTCT

TATATACATTGACATGGATCGGAATCTATTAACTTAAAGTTATTGGGGTCGTGAAATTTA

TTTAATTTTTCTAATTCGTGTCACATGTCACGACAATTAGTGGGG-AACATTTATGTATA

TAGAAATTTCAGAAATTTCTAGCAGCGTGATAAATATAAAGATTTTGGCACAATAAGTTT

TTGGATCATAATGAGTTT-CTATTTAATCAA-GGCA-TGTAA-TCTATTTTATTATTTAG

-TAGGG-AAACTGAAAACTTAGGGTTGCTATTTGTAGCTCCCACCCTTCTTAGTTCTTAC

TCTTTTCAATATTTTTTAAAAGTTTTA-TATAATACCT-AAATT-GCCCTCCTCCT----

----------------------------------------CACCCCATTGCTCTTCCGTT

GCTCCTCCATGTG-AGTTCTTGTCTTTTTTTTT-CTCTGGTAGCGTTTTGCTGCTCCTTT

TTTCACTCAAGTGTTGCCAATTAA-TTGACAAAAAATGGTTTCTGTTTCATATAGAAACT

ATGTTTTTGTTGTGTAGTCATACATTACGGAATCTAGTTT-CCATTAAATAAGTAAC--G

TGAAAAAAAA--TAAAAGGTGAAATATATATTGTTGGAAAAGAAGCTATGAGGTGCAAGA

ACCGATCACATGGAGAAGGCAATGAAAGACAAGGAGGAGCAATGGAAGA-----------

---------GAGAAAATGAGAAGATGGAAGGGATGTGAAAATGTTTGAAAAAAACGAGGT

GATCAGTTTTAAAATACGAATTTAGTATTTTCTTTTTAAGAAAATTCTTTCG-AAAGTCG

TGTTTTAAAACATGACTTTTATT-ATTTGAAGTCG---TGTTCTAAAACATGACTTA--T

TCATATCCTT-AATATTTTT---------AAAATTTATCCATTTGTAATATTTTTTAAAA

ATTGACCCATATATGTAAAATACCCGTCAAGATCTCTTTATTATTTTGAAAGCGAAAGCA

TATCACTTCAAACACAATGGAATCGAGGCTATTGACTAAGTATAAATAGAGAAGACTTCA

TATCGGGGTTCATAATTCATAACAAAGCAAACGAGTATATAAGAAAGCATAAGCCAAATT

TTGAGTAAACTAGTGTGCACACTATCCCATGCCTAGTGGAAGTAGGGATCCTCTCGTTGT

TGGGGGAGTAATTGGGGATGTATTGGATCCTTTTGAATATTCTATTCCTATGAGGGTTAC

CTACAATAACAGAGATGTCAGCAATGGATGTGAATTCAAACCCTCACAAGTTGTCAACCA

ACCAAGGGTAAATATCGGTGGTGATGACC

>CS08.03

TATTAGACTCCTAATTTAATATTCTTGTTTTATTATAATGCTGATAAGTCTTGTAAATAA

GGGTGAGAAGCACGAATAATTAGTTCATGAGATGTGTATAATTATTATCCTACACGACTT

ATCTTTGATATTTCACACAAGTCTTTCAATGTATAACAAAAACTTTTTAGATACATTTAG

ACTATAGAACTAACAAGTTATATTTTGAACC-AAAAAACAAAGAGAGAGAGAGAGGAACA

TAGAGAAAAGAAGATATGAGAGTTTTTTTTTTTTTTTT---CTAAAACAGAAAGAAACTC

ATTATATAATAAACAAATTACTTTGAGACAAATTAACGTATGTAATAAAACAAAATCAAA

GTAGTAATTAAAATTATTTAATGGTAAACAGTCTAATAGTTAAAACAAAAATGGAAATCA

CATAAATTTTGTAATTGGCCCATTAAAAACAACACTAAGCTTTTAATTTGATTTTGAAAT

TCAAAATAATTTTATTAAATCACAAAGTAAAAGGTTTACAAAGCCGATCATGACAGTGCA

TGTGGGAGGCAAATCGGCATATTTGCACTACAAAAGG-ACCCATGAAGTCTCTGAACATG

CACGCAACACTTTAATCTCTTATTAGTTACTTTGAAAGCTTATTTATATATATAGACACG

CGTAAAAACTTCTTAACCAAGATTTTTTT-ACGTGCTTCC-TTCGCGTTTAATTTGGACC

ATCAA-CCGTGCTCAACAGATAAAGAAAAGGGTGCTTTTGATTCAAGATATTGGCC-GAA

AAACACAAGATAGATCCTT-CGATAGATTAAGCCACGCATGAAACGCGAATCCAAAGTGA

TGAAGAAGTGCAGATAGATATTCGTTCACCATATAGAGGAGAATATATCATTCCTACAAA

CAAAATTGATGATGTTTCTAGAATAGAAACGTTAGAATCGAATATTCATCTGTTCCGGGT

GGGGAAGGGCTACTGAAAAGCTGAACTTTTTTAAGAGTTCGAACTTCGAAAGTCAATCAA

TGATTCTTAATTGAGAGGGAAAAGCTATAAAGGACGAGAAGGGAAGGAAATGTTTATAGG

GAATGAATTTCTAATGCATGATGGGAATGACAGATATGAGGAATATATAAAGGTGATTCT

TATATACATTGACATGGACCGGAATCTATTAACTTAAAGTTATTGGGGTCGTGAAATTTA

TTTAATTTTTCTAATTCGTGTCACATGTCACGACAATTAGTGGGG-AACATTTATGTATA

TAGAAATTTCAGAAATTTCTAGCAGCGTGATAAATATAAAGATTTTGGCACAATAAGTTT

TTGGATCATAATGAGTTT-CTATTTAATCAA-GGCA-TGTAA-TCTATTTTATTATTTAG

-TAGGG-AAACTGAAAACTTAGGGTTGCTATTTGTAGCTCCCACCCTTCTTAGTTCTTAC

TCTTTTCAATATTTTTTAAAAGTTTTA-TATAATACCT-AAATT-GCCCTCCTCCT----

----------------------------------------CACCCCATTGCTCTTCCGTT

GCTCCTCCATGTG-AGTTCTTGCCTTTTTTTTCTCTCTGGTAGCGTTTTGCTGCTCCTTT

TTTCACTCAAGTGTTGCCAATTAA-TTGACAAAAAATGGTTTCTGTTTCATATAGAAACT

ATGTTTTTGTTGTGTAGTCATACATTACGGAATCTAGTTT-CCATTAAATAAGTAAC--G

TGAAGAAAAA--TAAAAGGTGAAATATATATTGTTGGAAAAGAAGCTATGAGGTGCAAGA

ACCGATCACATGGAGAAGGCAATGAAAGACAAGGAGGAGCAATGGAAGA-----------

---------GAGAAAATGAGAAGATGGAAGGGATGTGAAAATGTTTGAAAAAAACGAGGT

GATCAGTTTTAAAATACGAATTTAGTATTTTCTTTTTAAGAAAATTCTTTCG-AAAGTCG

TGTTTTAAAACATGACTTTTATT-ATTTGAAGTCG---TGTTCTAAAACATGACTTA--T

TCATATCCTT-AATATTTTT---------AAAATTTATCCATTTGTAATATTTTTTAAAA

ATTGACCCATATATGTAAAATACCCGTCAAGATCTCTTTATTATTTTGAAAGCGAAAGCA

TATCACTTCAAACACAATGGAATCGAGGCTATTGACTAAGTATAAATAGAGAAGACTTCA

TATCGGGGTTCATAATTCATAACAAAGCAAACGAGTATATAAGAAAGCATAAGCCAAATT

TTGAGTAAACTAGTGTGCACACTATCCCATGCCTAGTGGAAGTAGGGATCCTCTCGTTGT

TGGGGGAGTAATTGGGGATGTATTGGATCCTTTTGAATATTCTATTCCTATGAGGGTTAC

CTACAATAACAGAGATGTCAGCAATGGATGTGAATTCAAACCCTCACAAGTTGTCAACCA

ACCAAGGGTAAATATCGGTGGTGATGACC

>CS18.18

TATTAGACTCCTAATTTAATATTCTTGTTTTATTACAATGCTGATAAGTCTTGTAAATAA

GGGTGAGAAGCACGAATAATTAGTTCATGAGATGTGTATAATTATTATCCTACACGGCTT

ATCTTTGATATTTCACACAAGTCTTTCAATGTATAACAAAAACTTTTTAGATACATTTAG

ACTATAGAACTAACAAGTTATATTTTGAACC-AAAAAACAAAGAGAGAGAGAGAGGAACA

TAGAGAAAAGAAGATATGAGAGTTTTTTTTTTTTTTTT---CTAAAACAGAAAGAAACTC

ATTATATAATAAACAAATTACTTTGAGACAAATTAACGTATGTAATAAAACAAAATCAAA

GTGGTAATTAAAATTATTTAATGGTAAACAGTCTAATAGTTAAAATAAAAATGGAAATCA

CATAAATTTTGTAATTGGCCCATTAAAAACAACACTAAGCTTTTAATTTGATTTTGAAAT

TCAAAATAATTTTATTAAATCACAAAGTAAAAGGTTTACAAAGCCGATCATGACAGTGCA

TGTGGGAGGCAAATCGGCATATTTGCACTACAAAAGG-ACCCATGAAGTCTCTGAACATG

CACGCAACACTTTAATCTCTTATTAGTTACTTTGAAAGCTTATTTATATATATAGACACG

CGTAAAAACTTCTTAACCAAGATTTTTTT-ACGTGCTTCC-TTCGCGTTTAATTTGGACC

ATCAAACCGTGCTCAACAGATAAAGAAAAGGGTGCTTTTGATTCAAGATATTGGCC-GAA

AAACACAAGATAGATCCTT-CGATAGATTAAGCCACGCATGAAACGCGAATCCAAAGTGA

TGAAGAAGTGCAGATAGATATTCGTTCACCATATAGAGGAGAATATATCATTCCTACAAA

CAAAATTGATGATGTTTCTAGAATAGAAACGTTAGAATCGAATATTCATCTGTTCCGGGT

GGGGAAGGGCTACTGAAAAGCTGAACTTTTTTAAGAGTTCGAACTTCGAAAGTCAATCAA

TGATTCTTAATTGAGAGGGAAAAGCTATAAAGGACGAGAAGGGAAGGAAATGTTTATAGG

GAATGAATTTCTAATGCATGATGGGAATGACAGATATGAGGAATATATAAAGGTGATTCT

TATATACATTGACATGGATCGGAATCTATTAACTTAAAGTTATTGGGGTCGTGAAATTTA

TTTAATTTTTCTAATTCGTGTCACATGTCACGACAATTAGTGGGG-AACATTTATGTATA

TAGAAATTTCAGAAATTTCTAGCAGCGTGATAAATATAAAGATTTTGGCACAATAAGTTT

TTGGATCATAATGAGTTT-CTATTTAATCAA-GGCA-TGTAA-TCTATTTTATTATTTAG

-TAGGG-AAACTGAAAACTTAGGGTTGCTATTTGTAGCTCCCACCCTTCTTAGTTCTTAC

TCTTTTCAATATTTTTTAAAAGTTTTA-TATAATACCT-AAATT-GCCCTCCTCCT----

----------------------------------------CACCCCATTGCTCTTCCGTT

GCTCCTCCATGTG-AGTTCTTGTCTTTTTTTTT-CTCTGGTAGCGTTTTGCTGCTCCTTT

TTTCCCTCAAGTGTTGCCAATTAA-TTGACAAAAAATGGTTTCTGTTTCATATAGAAACT

ATGTTTTTGTTGTGTAGTCATACATTACGGAATCTAGCTT-CCATTAAATAAGTAAC--G

TGAAAAAAAA--TAAAAGGTGAAATATATATTGTTGGAAAAGAAGCTATGAGGTGCAAGA

ACCGATCACATGGAGAAGGCAATGAAAGACGAGGAGGAGCAATGGAAGA-----------

---------GAGAAAATGAGAAGATGGAAGGGATGTGAAAATGTTTGAAAAGAACGAGGT

GATCAGTTTTAAAATACGAATTTAGTATTTTCTTTTTAAGAAAATTCTTTCG-AAAGTCG

TGTTTGAAAACATGACTTTTATT-ATTTGAAGTCG---TGTTCTAAAACATGACTTA--T

TCATATCCTT-AATATTTTT---------AAAATTTATCCATTTGTAATATTTTTTAAAA

ATTGACCCATATATGTAAAATACCCGTCAAGATCTCTTTATTATTTTGAAAGCGAAAGCA

TATCACTTCAAACACAATGGAATCGAGGCTATTGACTAAGTATAAATAGAGAAGACTTCA

TATCGGGGTTCATAATTCATAACAAAGCAAACGAGTATATAAGAAAGCATAAGCCAAATT

TTGAGTAAACTAGTGTGCACACTATCCCATGCCTAGTGGAAGTAGGGATCCTCTCGTTGT

TGGGGGAGTAATTGGGGATGTATTGGATCCTTTTGAATATTCTATTCCTATGAGGGTTAC

CTACAATAACAGAGATGTCAGCAATGGATGTGAATTCAAACCCTCACAAGTTGTCAACCA

ACCAAGGGTAAATATCGGTGGTGATGACC

>CS23.25

TATTAGACTCCTAATTTAATATTCTTGTTTTATTATAATGCTGATAAGTCTTGTAAATAA

GGGTGAGAAGCACGAATAATTAGTTCATGAGATGTGTATAATTATTATCCTACACGACTT

ATCTTTGATATTTCACACAAGTCTTTCAATGTATAACAAAAACTTTTTAGATACATTTAG

ACTATAGAACTAACAAGTTATATTTTGAACC-AAAAAACAAAGAGAGAGAGAGAGGAACA

TAGAGAAAAGAAGATATGAGAGTTTTTTTTTTTTTTTT---CTAAAACAGAAAGAAACTC

ATTATATAATAAACAAATTACTTTGAGACAAATTAACGTATGTAATAAAACAAAATCAAA

GTGGTAATTAAAATTATTTAATGGTAAACAGTCTAATAGTTAAAATAAAAATGGAAATCA

CATAAATTTTGTAATTGGCCCATTAAAAACAACACTAAGCTTTTAATTTGATTTTGAAAT

TCAAAATAATTTTATTAAATCACAAAGTAAAAGGTTTACAAAGCCGATCATGACAGTGCA

TGTGGGAGGCAAATCGGCATATTTGCACTACAAAAGG-ACCCATGAAGTCTCTGAACATG

CACGCAACACTTTAATCTCTTATTAGTTACTTTGAAAGCTTATTTATATATATAGACACG

CGTAAAAACTTCTTAACCAAGATTTTTTT-ACGTGCTTCC-TTCGCGTTTAATTTGGACC

ATCAAACCGTGCTCAACAGATAAAGAAAAGGGTGCTTTTGATTCAAGATATTGGCC-GAA

AAACACAAGATAGATCCTT-CGATAGATTAAGCCACGCATGAAACGCGAATCCAAAGTGA

TGGAGAAGTGCAGATAGATATTCGTTCACCATATAGAGGAGAATATATCATTCCTACAAA

CAAAATTGATGATGTTTCTAGAATAGAAACGTTAGAATCGAATATTCATCTGTTCCGGGT

GGGGAAGGGCTACTGAAAAGCTGAACTTTTTTAAGAGTTCGAACTTCGAAAGTCAATCAA

TGATTCTTAATTGAGAGGGAAAAGCTATAAAGGACGAGAAGGGAAGGAAATGTTTATAGG

GAATGAATTTCTAATGCATGATGGGGATGACAGATATGAGGAATATATAAAGGTGATTCT

TATATACATTGACATGGATCGGAATCTATTAACTTAAAGTTATTGGGGTCGTGAAATTTA

TCTAATTTTTCTAATTCGTGTCACATGTCACGACAATTAATGGGG-AACATTTATGTATA

TAGAAATTTCAGAAATTTCTAGCAGCGTGATAAATATAAAGATTTTGGCACAATAAGTTT

TTGGATCATAATGAGTTT-CTATTTAATCAA-GGCA-TGTAA-TCTATTTTATTATTTAG

-TAGGG-AAACTGAAAACTTAGGGTTGCTATTTGTAGCTCCCACCCTTCTTAGTTCTTAC

TCTTTTCAATATTTTTTAAAAGTTTTA-TATAATACCT-AAATT-GCCCTCCTCCT----

----------------------------------------CACCCCATTGCTCTTCCGTT

GCTCCTCCATGTG-AGTTCTTGTCTTTTTTTTT-CTCTGGTAGCGTTTTGCTGCTCCTTT

TTTCACTCAAGTGTTGCCAATTAA-TTGACAAAAAATGGTTTCTGTTTCATATAGAAACT

ATGTTTTTGTTGTGTAGTCATACATTACGGAATCTAGTTT-CCATTAAATAAGTAAC--G

TGAAAAAAAA--TAAAAGGTGAAATATATATTGTTGGAAAAGAAGCTATGAGGTGCAAGA

ACCGATCACATGGAGAAGGCAATGAAAGACAAGGAGGAGCAATGGAAGA-----------

---------GAGAAAATGAGAAGATGGAAGGGATGTGAAAATGTTTGAAAAAAACGAGGT

GATCAGTTTTAAAATACGAATTTAGTATTTTCTTTTTAAGAAAATTCTTTCG-AAAGTCG

TGTTTTAAAACATGACTTTTATT-ATTTGAAGTCG---TGTTCTAAAACATGACTTA--T

TCATATCCTT-AATATTTTT---------AAAATTTATCCATTTGTAATATTTTTTAAAA

ATTGACCCATATATGTAAAATACCCGTCAAGATCTCTTTATTATTTTGAAAGCGAAAGCA

TATCACTTCAAACACAATGGAATCGAGGCTATTGACTAAGTATAAATAGAGAAGACTTCA

TATCGGGGTTCATAATTCATAACAAAGCAAACGAGTATATAGGAAAGCATAAGCCAAATT

TTGAGTAAACTAGTGTGCACACTATCCCATGCCTAGTGGAAGTAGGGATCCTCTCGTTGT

TGGGGGAGTAATTGGGGATGTATTGGATCCTTTTGAATATTCTATTCCTATGAGGGTTAC

CTACAATAACAGAGATGTCAGCAATGGATGTGAATTCAAACCCTCACAAGTTGTCAACCA

ACCAAGGGTAAATATCGGTGGTGATGACC

>CS17.01

TATTAGACTCCTAATTTAATATTCTTGTTTTATTATAATGCTGATAAGTCTTGTAAATAA

GGGTGAGAAGCACGAATAATTAGTTCATGAGATGTGTATAATTATTATCCTACACGACTT

ATCTTTGATATTTCACACAAGTCTTTCAATGTATAACAAAAACTTTTTAGATACATTTAG

ACTATAGAACTAACAAGTTATATTTTGAACC-AAAAAACAAAGAGAGAGAGAGAGGAACA

TAGAGAAAAGAAGATATGAGAGTTTTTTTTTTTTTTTTT--CTAAAACAGAAAGAAACTC

ATTATATAATAAACAAATTACTTTGAGACAAATTAACGTATGTAATAAAACAAAATCAAA

GTGGTAATTAAAATTATTTAATGGTAAACAGTCTAATAGTTAAAATAAAAATGGAAATCA

CATAAATTTTGTAATTGGCCCATTAAAAACAACACTAAGCTTTTAATTTGATTTTGAAAT

TCAAAATAATTTTATTAAATCACAAAGTAAAAGGTTTACAGAGCCGATCATGACAGTGCA

TGTGGGAGGCAAATCGGCATATTTGCACTACAAAAGG-ACCCATGAAGTCTCTGAACATG

CACGCAACACTTTAATCTCTTATTAGTTACTTTGAAAGCTTATTTATATATATAGACACG

CGTAAAAACTTCTTAACCAAGATTTTTTT-ACGTGCTTCC-TTCGCGTTTAATTTGGACC

ATCAAACCGTGCTCAACGGATAAAGAAAAGGGTGCTTTTGATTCAAGATATTGGCC-GAA

AAACACAAGATAGATCCTT-CGATAGGTTAAGCCACGCATGAAACGCGAATCCAAAGTGA

CGAAGAAGTGCAGATAGATATTCGTTCACCATATAGAGGAGAATATATCATTCCTACAAA

CAAAATTGATGATGTTTCTAGAATAGAAACGTTAGAATCGAATATTCATCTGTTCCGGGT

GGGGAAGGGCTACTGAAAAGCTGAACTTTTTTAAGAGTTCGAACTTCGAAAGTCAATCAA

TGATTCTTAATTGAGAGGGAAAAGCTATAAAGGACGAGAAGGGAAGGAAATGTTTATAGG

GAATGAATTTCTAATGCATGATGGGAATGACAGATATGAGGAATATATAAAGGTGATTCT

TATATACATTGACATGGATCGGAATCTATTAACTTAAAGTTATTGGGGTCGTGAAATTTA

TTTAATTTTTCTAATTCGTGTCACATGTCACGACAATTAGTGGGG-AACATTTATGTATA

TAGAAATTTCAGAAATTTCTAGCAGCGTGATAAATATAAAGATCTTGGCACAATAAGTTT

TTGGATCATAATGAGTTT-CTATTTAATCAA-GGCA-TGTAA-TCTATTTTATTATTTAG

-TAGGG-AAACTGAAAACTTAGGGTCGCTATTTGTAGCTCCCACCCTTCTTAGTTCTTAC

TCTTTTCAATATTTTTTAAAAGTTTTA-TATAATACCT-AAATT-GCCCTCCTCCT----

----------------------------------------CACCCCATTGCTCTTCCGTT

GCTCCTCCATGTG-AGTTCTTGTCTTTTTTTTT-CTCTGGTAGCGTTTTGCTGCTCCTTT

TTTCACTCAAGTGTTGCCAATTAA-TTGACAAAAAATGGTTTCTGTTTCATATAGAAACT

ATGTTTTTGTTGTGTAGTCATACATTACGGAATCTAGTTT-CCATTAAATAAGTAAC--G

TGAAAAAAAA--TAAAAGGTGAAATATATATTGTTGGAAAAGAAGCTATGAGGTGCAAGA

ACCGATCACATGGAGAAGGCAATGAAAGACAAGGAGGAGCAATGGAAGA-----------

---------GAGAAAATGAGAAGATGGAAGGGATGTGAAAATGTTTGAAAAAAACGAGGT

GATCAGTTTTAAAATACGAATTTAGTATTTTCTTTTTAAGAAAATTCTTTCG-AAAGTCG

TGTTTTAAAACATGACTTTTATT-ATTTGAAGTCG---TGTTCTAAAACATGACTTA--T

TCATATCCTT-AATATTTTT---------AAAATTTATCCATTTGTAATATTTTTTAAAA

ATTGACCCATATATGTAAAATACCCGTCAAGATCTCTTTATTATTTTGAAAGCGAAAGCA

TATCACTTCAAACACAATGGAATCGAGGCTATTGACTAAGTATAAATAGAGAAGACTTCA

TATCGGGGTTCATAATTCATAACAAAGCAAACGAGTATATAAGAAAGCATAAGCCAAATT

TTGAGTAAACTAGTGTGCACACTATCCCATGCCTAGTGGAAGTAGGGATCCTCTCGTTGT

TGGGGGAGTAATTGGGGATGTATTGGATCCTTTTGAATATTCTATTCCTATGAGGGTTAC

CTACAATAACAGAGATGTCAGCAATGGATGTGAATTCAAACCCTCACAAGTTGTCAACCA

ACCAAGGGTAAATATCGGTGGTGATGACC

>CS27.18

TATTAGACTCCTAATTTAATATTCTTGTTTTATTATAATGCTGATAAGTCTTGTAAATAA

GGGTGAGAAGCACGAATAATTAGTTCATGAGATGTGTATAATTATTATCCTACACGACTT

ATCTTTGATATTTCACACAAGTCTTTCAATGTATAACAAAAACTTTTTAGATACATTTAG

ACTATAGAACTAACAAGTTATATTTTGAACC-AAAAAACAAAGAGAGAGAGAGAGGAACA

TAGAGAAAAGAAGATATGAGAGTTTTTTTTTTTTTTTT---CTAAAACAGAAAGAAACTC

ATTATATAATAAACAAATTACTTTGAGACAAATTAACGTATGTAATAAAACAAAATCAAA

GTGGTAATTAAAATTATTTAATGGTAAACAGTCTAATAGTTAAAATAAAAATGGAAATCA

CATAAATTTTGTAATTGGCCCATTAAAAACAACACTAAGCTTTTAATTTGATTTTGAAAT

TCAAAATAATTTTATTAAATCACAAAGTAAAAGGTTTACAAAGCCGATCATGACAGTGCA

TGTGGGAGGCAAATCGGCATATTTGCACTACAAAAGG-ACCCATGAAGTCTCTGAACATG

CACGCAACACTTTAATCTCTTATTAGTTACTTTGAAAGCTTATTTATATATATAGACACG

CGTAAAAACTTCTTAACCAAGATTTTTTT-ACGTGCTTCC-TTCGCGTTTAATTTGGACC

ATCAAACCGTGCTCAACAGATAAAGAAAAGGGTGCTTTTGATTCAAGATATTGGCC-GAA

AAACACAAGATAGATCCTT-CGATAGATTAAGCCACGCATGAAACGCGAATCCAAAGTGA

TGAAGAAGCGCAGATAGATATTCGTTCACCATATAGAGGAGAATATATCATTCCTACAAA

CAAAATTGATGATGTTTCTAGAATAGAAACGTTAGAATCGAATATTCATCTGTTCCGGGT

GGGGAAGGGCTACTGAAAAGCTGAACTTTTTTAAGAGTTCGAACTTCGAAAGTCAATCAA

TGATTCTTAATTGAGAGGGAAAAGCTATAAAGGACGAGAAGGGAAGGAAATGTTTATAGG

GAATGAATTTCTAATGCATGATGGGAATGACAGATATGAGGAATATATAAAGGTGATTCT

TATATACATTGACATGGATCGGAATCTATTAACTTAAAGTTATTGGGGTCGTGAAATTTA

TCTAATTTTTCTAATTCGTGTCACATGTCACGACAATTAGTGGGG-AACATTTATGTATA

TAGAAATTTCAGAAATTTCTAGCAGCGTGATAAATATAAAGATTTTGGCACAATAAGTTT

TTGGATCATAATGAGTTT-CTATTTAATCAA-GGCA-TGTAA-TCTATTTTATTATTTAG

-TAGGG-AAACTGAAAACTTAGGGTTGCTATTTGTAGCTCCCACCCTTCTTAGTTCTTAC

TCTTTTCAATATTTTTTAAAAGTTTTA-TATAATACCT-AAATT-GCCCTCCTCCT----

----------------------------------------CACCCCATTGCTCTTCCGTT

GCTCCTCCATGTG-AGTTCTTGTCTTTTTTTTT-CTCTGGTAGCGTTTTGCTGCTCCTTT

TTTCACTCAAGTGTTGCCAATTAA-TTGACAAAAAATGGTTTCTGTTTCATATAGAAACT

ATGTTTTTGTTGTGTAGTCATACATTACGGAATCTAGTTT-CCATTAAATAAGTAAC--G

TGAAAAAAAA--TAAAAGGTGAAATATATATTGTTGGAAAAGAAGCTATGAGGTGCAAGA

ACCGATCACATGGAGAAGGCAATGAAAGACAAGGAGGAGCAATGGAAGA-----------

---------GAGAAAATGAGAAGATGGAAGGGATGTGAAAATGTTTGAAAAAAACGAGGT

GATCAGTTTTAAAATACGAATTTAGTATTTTCTTTTTAAGAAAATTCTTTCG-AAAGTCG

TGTTTTAAAACATGACTTTTATT-ATTTGAAGTCG---TGTTCTAAAACATGACTTA--T

TCATATCCTT-AATATTTTT---------AAAATTTATCCATTTGTAATATTTTTTAAAA

ATTGACCCATATATGTAAAATACCCGTCAAGATCTCTTTATTATTTTGAAAGCGAAAGCA

TATCACTTCAAACACAATGGAATCGAGGCTATTGACTAAGTATAAATAGAGAAGACTTCA

TATCGGGGTTCATAATTCATAACAAAGCAAACGAGTATATAAGAAAGCATAAGCCAAATT

TTGAGTAAACTAGTGTGCACACTATCCCATGCCTAGTGGAAGTAGGGATCCTCTCGTTGT

TGGGGGAGTAATTGGGGATGTATTGGATCCTTTTGAATATTCTATTCCTATGAGGGTTAC

CTACAATAACAGAGATGTCAGCAATGGATGTGAATTCAAACCCTCACAAGTTGTCAACCA

ACCAAGGGTAAATATCGGTGGTGATGACC

>CS23.32

TATTAGACTCCTAATTTAATATTCTTGTTTTATTATAATGCTGATAAGTCTTGTAAATAA

GGGTGAGAAGCACGAATAATTAGTTCATGAGATGTGTATAATTATTATCCTACACGACTT

ATCTTTGATATTTCACACAAGTCTTTCAATGTATAACAAAAACTTTTTAGATACATTTAG

ACTATAGAACTAACAAGTTATATTTTGAACC-AAAAAACAAAGAGAGAGAGAGAGGAACA

TAGAGAAAAGAAGATATGAGAGTTTTTTTTTTTTTTTT---CTAAAACAGAAAGAAACTC

ATTATATAATAAACAAATTACTTTGAGACAAATTAACGTATGTAATAAAACAAAATCAAA

GTGGTAATTAAAATTATTTAATGGTAAACAGTCTAATAGTTAAAATAAAAATGGAAATCA

CATAAATTTTGTAATTGGCCCATTAAAAACAACACTAAGCTTTTAATTTGATTTTGAAAT

TCAAAATAATTTTATTAAATCACAAAGTAAAAGGTTTACAAAGCCGATCATGACAGTGCA

TGTGGGAGGCAAATCGGCATATTTGCACTACAAAAGG-ACCCATGAAGTCTCTGAACATG

CACGCAACACTTTAATCTCTTATTAGTTACTTTGAAAGCTTATTTATATATATAGACACG

CGTAAAAACTTCTTAACCAAGATTTTTTT-ACGTGCTTCC-TTCGCGTTTAATTTGGACC

ATCAAACCGTGCTCAACAGATAAAGAAAAGGGTGCTTTTGATTCAAGATATTGGCC-GAA

AAACACAAGATAGATCCTT-CGATAGATTAAGCCACGCATGAAACGCGAATCCAAAGTGA

TGAAGAAGTGCAGATAGATATTCGTTCACCATATAGAGGAGAATATATCATTCCTACAAA

CAAAATTGATGATGTTTCTAGAATAGAAACGTTAGAATCGAATATTCATCTGTTCCGGGT

GGGGAAGGGCTACTGAAAAGCCGAACTTTTTTAAGAGTTCGAACTTCGAAAGTCAATCAA

TGATTCTTAATTGAGAGGGAAAAGCTATAAAGGACGAGAAGGGAAGGAAATGTTTATAGG

GAATGAGTTTCTAATGCATGATGGGAATGACAGATATGAGGAATATATAAAGGTGATTCT

TATATACATTGACATGGATCGGAATCTATTAACTTAAAGTTATTGGGGTCGTGAAATTTA

TTTAATTTTTCTAATTCGTGTCACATGTCACGACAATTAGTGGGG-AACATTTATGTATA

TAGAAATTTCAGAAATTTCTAGCAGCGTGATAAATATAAAGATTTTGGCACAATAAGTTT

TTGGATCATAATGAGTTT-CTATTTAATCAA-GGCA-TGTAA-TCTATTTTATTATTTAG

-TAGGG-AAACTGAAAACTTAGGGTTGCTATTTGTAGCTCCCACCCTTCTTAGTTCTTAC

TCTTTTCAATATTTTTTAAAAGTTTTA-TATAATACCT-AAATT-GCCCTCCTCCT----

----------------------------------------CACCCCATTGCTCTTCCGTT

GCTCCTCCATGTG-AGTTCTTGTCTTTTTTTTT-CTCTGGTAGCGTTTTGCTGCTCCTTT

TTTCACTCAAGTGTTGCCAATTAA-TTGACAAAAAATGGTTTCTGTTTCATATAGAAACT

ATGTTTTTGTTGTGTAGTCATACATTACGGAATCTAGTTT-CCATTAAATAAGTAAC--G

TGAAAAAAA---TAAAAGGTGAAATATATATTGTTGGAAAAGAAGCTATGAGGTGCAAGA

ACCGATCACATGGAGAAGGCAATGAAAGACAAGGAGGAGCAATGGAAGA-----------

---------GAGAAAATGAGAAGATGGAAGGGATGTGAAAATGTTTGAAAAAAACGAGGT

GATCAGTTTTAAAATACGAATTTAGTATTTTCTTTTTAAGAAAATTCTTTCG-AAAGTCG

TGTTTTAAAACATGACTTTTATT-ATTTGAAGTCG---TGTTCTAAAACATGACTTA--T

TCATATCCTT-AATATTTTT---------AAAATTTATCCATTTGTAATATTTTTTAAAA

ATTGACCCATATATGTAAAATACCCGTCAAGATCTCTTTATTATTTTGAAAGCGAAAGCA

TATCACTTCAAACACAATGGAATCGAGGCTATTGACTAAGTATAAATAGAGAAGACTTCA

TATCGGGGTTCATAATTCATAACAAAGCAAACGAGTATATAAGAAAGCATAAGCCAAATT

TTGAGTAAACTAGTGTGCACACTATCCCATGCCTAGTGGAAGTAGGGATCCTCTCGTTGT

TGGGGGAGTAATTGGGGATGTATTGGATCCTTTTGAATGTTCTATTCCTATGAGGGTTAC

CTACAATAACAGAGATGTCAGCAATGAATGTGAATTCAAACCCTCACAAGTTGTCAACCA

ACCAAGGGTAAATATCGGTGGTGATGACC

>CS29.18

TATTAGACTCCTAATTTAATATTCTTGTTTTATTATAATGCTGATAAGTCTTGTAAATAA

GGGTGAGAAGCACGAATAATTAGTTCATGAGATGTGTATAATTATTATCCTACACGACTT

ATCTTTGATATTTCACACAAGTCTTTCAATGTATAACAAAAACTTTTTAGATACATTTAG

ACTGTAGAACTAACAAGTTATATTTTGAACC-AAAAAACAAAGAGAGAGAGAGAGGAACA

TAGAGAAAAGAAGATATGAGAGTTTTTTTTTTTTTTTT---CTAAAACAGAAAGAAACTC

ATTATATAATAAACAAATTACTTTGAGACAAATTAACGTATGTAATAAAACAAAATCAAA

GTGGTAATTAAAATTATTTAATGGTAAACAGTCTAATAGTTAAAATAAAAATGGAAATCA

CATAAATTTTGTAATTGGCCCATTAAAAACAACACTAAGCTTTTAATTTGATTTTGAAAT

TCAAAATAATTTTATTAAATCACAAAGTAAAAGGTTTACAAAGCCGATCATGACAGTGCA

TGTGGGAGGCAAATCGGCATATTTGCACTACAAAAGG-ACCCATGAAGTCTCTGAACATG

CACGCAACACTTTAATCTCTTATTAGTTACTTTGAAAGCTTATTTATATATATAGACACG

CGTAAAAACTTCTTAACCAAGATTTTTTT-ACGTGCTTCC-TTCGCGTTTAATTTGGACC

ATCAAACCGTGCTCAACAGATAAAGAAAAGGGTACTTTTGATTCAAGATATTGGCC-GAA

AAACACAAGATAGATCCTT-CGATAGATTAAGCCACGCATGAAACGCGAATCCAAAGTGA

TGAAGAAGTGCAGATAGATATTCGTTCACCATATAGAGGAGAATATATCATTCCTACAAA

CAAAATTGATGATGTTTCTAGAATAGAAACGTTAGAATCGAATATTCATCTGTTCCGGGT

GGGGAAGGGCTACTGAAAAGCTGAACTTTTTTAAGAGTTCGAACTTCGAAAGTCAATCAA

TGATTCTTAATTGAGAGGGAAAAGCTATAAAGGACGAGAAGGGAAGGAAATGTTTATAGG

GAATGAATTTCTAATGCATGATGGGAATGACAGATATGAGGAATATATAAAGGTGATTCT

TATATACATTGACATGGATCGGAATCTATTAACTTAAAGTTATTGGGGTCGTGAAATTTA

TTTAATTTTTCTAATTCGTGTCACATGTCACGACAATTAGTGGGG-AACATTTATGTATA

TAGAAATTTCAGAAATTTCTAGCAGCGTGATAAATATAAAGATTTTGGCACAATAAGTTT

TTGGATCATAATGAGTTT-CTATTTAATCAA-GGCA-TGTAA-TCTATTTTATTATTTAG

-TAGGG-AAACTGAAAACTTAGGGTTGCTATTTGTAGCTCCCACCCTTCTTAGTTCTTAC

TCTTTTCAATATTTTTTAAAAGTTTTA-TATAATACCT-AAATT-GCCCTCCTCCT----

----------------------------------------CACCCCATTGCTCTTCCGTT

GCTCCTCCATGTG-AGTTCTTGTCTTTTTTTTT-CTCTGGTAGCGTTTTGCTGCTCCTTT

TTTCACTCAAGTGTTGCCAATTAA-TTGACAAAAAATGGTTTCTGTTTCATATAGAAACT

ATGTTTTTGTTGTGTAGTCATACATTACGGAATCTAGTTT-CCTTTAAATAAGTAAC--G

TGAAAAAAAA--TAAAAGGTGAAATTTATATTGTTGGAAAAGAAGCTATGAGGTGCAAGA

ACCGATCACATGGAGAAGGCAATGAAAGACAAGGAGGAGCAATAGAAGA-----------

---------GAGAAAATGAGAAGATGGAAGGGATGTGAGAATGTTTGAAAAAAACGAGGT

GATCAGTTTTAAAATACGAATTTAGTATTTTCTTTTTAAGAAAATTCTTTCG-AAAGTCG

TGTTTTAAAACATGACTTTTATT-ATTTGAAGTCG---TGTTCTAAAACATGACTTA--T

TCATATCCTT-AATATTTTT---------AAAATTTATCCATTTGTAATATTTTTTAAAA

ATTGACCCATATATGTAAAATACCCGTCAAGATCTCTTTATTATTTTGAAAGCGAAAGCA

TATCACTTCAAACACAATGGAATCGAGGCTATTGACTAAGTATAAATAGAGAAGACTTCA

TATCGGGGTTCATAATTCATAACAAAGCAAACGAGTATATAAGAAAGCATAAGCCAAATT

TTGAGTAAACTAGTGTGCACACTATCCCATGCCTAGTGGAAGTAGGGATCCTCTCGTTGT

TGGGGGAGTAATTGGGGATGTATTGGATCCTTTTGAATATTCTATTCCTATGAGGGTTAC

CTACAATAACAGAGATGTCAGCAATGGATGTGAATTCAAACCCTCACAAGTTGTCAACCA

ACCAAGGGTAAATATCGGTGGTGATGACC

>CS08.02

TATTAGACTCCTAATTTAATATTCTTGTTTTATTATAATGCTGATAAGTCTTGTAAATAA

GGGTGAGAAGCACGAATAATTAGTTCATGAGATGTGTATAATTATTATCCTACACGACTT

ATCTTTGATATTTCACACAAGTCTTTCAATGTATAACAAAAACTTTTTAGATACATTTAG

ACTATAGAACTAACAAGTTATATTTTGAACC-AAAAAACAAAGAGAGAGAGAGAGGAACA

TAGAGAAAAGAAGATATGAGAGTTTTTTTTTTTTTTTTT--CTAAAACAGAAAGAAACTC

ATTATATAATAAACAAATTACTTTGAGACAAATTAACGTATGTAATAAAACAAAATCAAA

GTGGTAATTAAAATTATTTAATGGTAAACAGTCTAATAGTTAAAATAAAAATGGAAATCA

CATAAATTTTGTAATTGGCCCATTAAAAACAACACTAAGCTTTTAATTTGATTTTGAAAT

TCAAAATAATTTTATTAAATCACAAAGTAAAAGGTTTACAAAGCCGATCATGACAGTGCA

TGTGGGAGGCAAATCGGCATATTTGCACTACAAAAGG-ACCCATGAAGTCTCTGAACATG

CACGCAACACTTTAATCTCTTATTAGTTACTTTGAAAGCTTATTTATATATATAGACACG

CGTAAAAACTTCTTAACCAAGATTTTTT--ACGTGCTTCC-TTCGCGTTTAATTTGGACC

ATCAAACCGTGCTCAACAGATAAAGAAAAGGGTGCTTTTGATTCAAGATATTGGCC-GAA

AAACACAAGATAGATCCTT-CGATAGATTAAGCCACGCATGAAACGCGAATCCAAAGTGA

TGAAGAAATGCAGATAGATATTCGTTCACCATATAGAGGAGAATATATCATTCCTACAAA

CGAAATTGATGATGTTTCTAGAATAGAAACGTTAGAATCGAATATTCATCTGTTCCGGGT

GGGGAAGGGCTACTGAAAAGCTGAACTTTTTTAAGAGTTCGAACTTCGAAAGTCAATCAA

TGATTCTTAATTGAGAGGGAAAAGCTATAAAGGACGAGAAGGGAAGGAAATGTTTATAGG

GAATGAATTTCTAATGCATGATGGGAATGACAGATATGAGGAATATATAAAGGTGACTCT

TATATACATTGACATGGATCGGAATCTATTAACTTAAAGTTATTGGGGTCGTGAAATTTA

TTTAATTTTTCTAATTCGTGTCACATGTCACGACAATTAGTGGGG-AACATTTATGTATA

TAGAAATTTCAGAAATTTCTAGCAGCGTGATAAATATAAAGATTTTGGCACAATAAGTTT

TTGGATCATAATGAGTTT-CTATTTAATCAA-GGCA-TGTAA-TCTATTTTATTATTTAG

-TAGGG-AAACTGAAAACTTAGGGTTGCTATTTGTAGCTCCCACCCTTCTTAGTTCTTAC

TCTTTTCAATATTTTTTAAAAGTTTTA-TATAATACCT-AAATT-GCCCTCCTCCT----

----------------------------------------CACCCCATTGCTCTTCCGTT

GCTCCTCCATGTG-AGTTCTTGTCTTTTTTTTT-CTCTGGTAGCGTTTTGCTGCTCCTTT

TTTCACTCAAGTGTTGCCAATTAA-TTGACAAAAAATGGTTTCTGTTTCATATAGAAACT

ATGTTTTTGTTGTGTAGTCATACATTACGGAATCTAGTTT-CCATTAAATAAGTAAC--G

TGAAAAAAAA--TAAAAGGTGAAATATATATTGTTGGAAAAGAAGCTATGAGGTGCAAGA

ACCGATCACATGGAGAAGGCAATGAAAGACAAGGAGGAGCAATGGAAGA-----------

---------GAGAAAATGAGAAGATGGAAGGGATGTGAAAATGTTTGAAAAAAACGAGGT

GATCAGTTTTAAAATACGAATTTAGTATTTTCTTTTTAAGAAAATTCTTTCG-AAAGTCG

TGTTTTAAAACATGACTTTTATT-ATTTGAAGTCG---TGTTCTAAAACATGACTTA--T

TCATATCCTT-AATATTTTT---------AAAATTTATCCATTTGTAATATTTTTTAAAA

ATTGACCCATATATGTAAAATACCCGTCAAGATCTCTTTATTATTTTGAAAGCGGAAGCA

TATCACTTCAAACACAATGGAATCGAGGCTATTGACTAAGTATAAATAGAGAAGACTTCA

TATCGGGGTTCATAATTCATAACAAAGCAAACGAGTATATAAGAAAGCATAAGCCAAATT

TTGAGTAAACTAGTGTGCACACTATCCCATGCCTAGTGGAAGTAGGGATCCTCTCGTTGT

TGGGGGAGTAATTGGGGATGTATTGGATCCTTTTGAATATTCTATTCCTATGAGGGTTAC

CTACAATAACAGAGATGTCAGCAATGGATGTGAATTCAAACCCTCACAAGTTGTCAACCA

ACCAAGGGTAAATATCGGTGGTGATGACC

>CS29.39

TATTAGACTCCTAATTTAATATTCTTGTTTTATTATAATGCTGATAAGTCTTGTAAATAA

GGGTGAGAAGCACGAATAATTAGTTCATGAGATGTGTATAATTATTATCCTACACGACTT

ATCTTTGATATTTCACACAAGTCTTTCAATGTATAACAAAAACTTTTTAGATACATTTAG

ACTATAGAACTAACAAGTTATATTTTGAACC-AAAAAACAAAGAGAGAGAGAGAGGAACA

TAGAGAAAAGAAGATATGAGAGTTTTTTTTTTTTTTTT---CTAAAACAGAAAGAAACTC

ATTATATAATAAACAAATTACTTTGAGACAAATTAACGTATGTAATAAAACAAAATCAAA

GTGGTAATTAAAATTATTTAATGGTAAACAGTCTAATAGTTAAAATAAAAATGGAAATCA

CATAAATTTTGTAATTGGCCCATTAAAAACAACACTAAGCTTTTAATTTGATTTTGAAAT

TCAAAATAATTTTATTAAATCACAAAGTAAAAGGTTTACAAAGCCGATCATGACAGTGCA

TGTGGGAGGCAAATCGGCATATTTGCACTACAAAAGG-ACCCATGAAGTCTCTGAACATG

CACGCAACACTTTAATCTCTTATTAGTTACTTTGAAAGCTTATTTATATATATAGACACG

CGTAAAAACTTCTTAACCAAGATTTTTTT-ACGTGCTTCC-TTCGCGTTTAATTTGGACC

ATCAAACCGTGCTCAACAGATAAAGAAAAGGGTGCTTTTGATTCAAGATATTGGCC-GAA

AAACACAAGATAGATCCTT-CGATAGATTAAGCCACGCATGAAACGCGAATCCAAAGTGA

TGAAGAAGTGCAGATAGATATTCGTTCACCATATAGAGGAGAATATATCATTCCTACAAA

CAAAATTGATGATGTTTCTAGAATAGAAACGTTAGAATCGAATATTCATCTGTTCCGGGT

GGGGAAGGGCTACTGAAAAGCTGAACTTTTTTAAGAGTTCGAACTTCGAAAGTCAATCAA

TGATTCTTAATTGAGAGGGAAAAGCTATAAAGGACGAGAAGGGAAGGAAATGTTTATAGG

GAATGAATTTCTAATGCATGATGGGAATGACAGATATGAGGAATACATAAAGGTGATTCT

TATATACATTGACATGGATCGGAATCTATTAACTTAAAGTTATTGGGGTCGTGAAATTTA

TTTAATTTTTCTAATTCGTGTCACATGTCACGACAATTAGTGGG--AACATTTATGTATA

TAGAAATTTCAGAAATTTCTAGCAGCGTGATAAATATAAAGATTTTGGCACAATAAGTTT

TTGGATCATAATGAGTTT-CTATTTAATCAA-GGCA-TGTAA-TCTATTTTATTATTTAG

-TAGGG-AAACTGAAAACTTAGGGTTGCTATTTGTAGCTCCCACCCTTCTTAGT-CTTAC

TCTTTTCAATATTTTTTAAAAGTTTTA-TATA-TACCT-AGATT-GCCCTCCTCCT----

----------------------------------------CACCCCATTGCTCTTCCGTT

GCTCCTCCATGTG-AGTTCTTGTCTTTTTTTT--CTCTGGTAGCGTTTTGCTGCTCCTTT

TTTCACTCAAGTGTTGCCAATTAA-TTGACAAAAAATGGTTTCTGTTTCATATAGAAACT

ATGTTTTTGTTGTGTAGTCATACATTACGGAATCTAGTTT-CCATTAAATAAGTAAC--G

TGAAAAAAAA--TAAAAGGTGAAATATATATTGTTGGAAAAGAAGCTATGAGGTGCAAGA

ACCGATCACATGGAGAAGGCAATGAAAGACAAGGAGGAGCAATGGAAGA-----------

---------GAGAAAATGAGAAGATGGAAGGGATGTGAAAATGTTTGAAAAAAACGAGGT

GATCAGTTTTAAAATACGAATTTAGTATTTTCTTTTTAAGAAAATTCTTTCG-AAAGTCG

TGTTTTAAAACATGACTTTTATT-ATTTGAAGTCG---TGTTCTAAAACATGACTTA--T

TCATATCCTT-AATATTTTT---------AAAATTTATCCATTTGTAATATTTTTTAAAA

ATTGACCCATATATGTAAAATACCCGTCAAGATCTCTTTATTATTTTGAAAGCGAAAGCA

TATCACTTCAAACACAATGGAATCGAGGCTATTGACTAAGTATAAATAGAGAAGACTTCA

TATCGGGGTTCATAATTCATAACAAAGCAAACGAGTATATAAGAAAGCATAAGCCAAATT

TTGAGTAAACTAGTGTGCACACTATCCCATGCCTAGTGGAAGTAGGGATCCTCTCGTTGT

TGGGGGAGTAATTGGGGATGTATTGGATCCTTTTGAATATTCTATTCCTATGAGGGTTAC

CTACAATAACAGAGATGTCAGCAATGGATGTGAATTCAAACCCTCACAAGTTGTCAACCA

ACCAAGGGTAAATATCGGTGGTGATGACC

>CS13.01

TATTAGACTCCTAATTTAATATTCTTGTTTTATTATAATGCTGATAAGTCTTGTAAATAA

GGGTGAGAAGCACGAATAATTAGTTCATGAGATGTGTATAATTATTATCCTACACGACTT

ATCTTTGATATTTCACACAAGTCTTTCAATGTATAACAAAAACTTTTTAGATACATTTAG

ACTATAGAACTAACAAGTTATATTTTGAACC-AAAAAACAAAGAGAGAGAGAGAGGAACA

TAGAGAAAAGAAGATATGAGAGTTTTTTTTTTTTTTTTT--CTAAAACAGAAAGAAACTC

ATTATATAATAAACAAATTACTTTGAGACAAATTAACGTATGTAATAAAACAAAATCAAA

GTGGTAATTAAAATTATTTAATGGTAAACAGTCTAATAGTTAAAATAAAAATGGAAATCA

CATAAATTTTGTAATTGGCCCATTAAAAACAACACTAAGCTTTTAATTTGATTTTGAAAT

TCAAAATAATTTTATTAAATCACAAAGTAAAAGGTTTACAAAGCCGATCATGACAGTGCA

TGTGGGAGGCAAATCGGCATATTTGCACTACAAAAGG-ACCCATGAAGTCTCTGAACATG

CGCGCAACACTTTAATCTCTTATTAGTTACTTTGAAAGCTTATTTATATATATAGACACG

CGTAAAAACTTCTTAACCAAGATTTTTTT-ACGTGCTTCC-TTCGCGTTTAATTTGGACC

ATCAAACCGTGCTCAACAGATAAAGAAAAGGGTGCTTTTGATTCAAGATATTGGCC-GAA

AAACACAAGATAGATCCTT-CGATAGATTAAGCCACGCATGAAACGCGAATCCAAAGTGA

TGAAGAAGTGCAGATAGATATTCGTTCACCATATAGAGGAGAATATATCATTCCTACAAA

CAAAATTGATGATGTTTCTAGAATAGAAACGTTAGAATCGAATATTCATCTGTTCCGGGT

GGGGAAGGGCTACTGAAAAGCTGAACTTTTTTAAGAGTTCGAACCTCGAAAGTCAATCAA

TGATTCTTAATTGAGAGGGAAAAGCTATAAAGGACGAGAAGGGAAGGAAATGTTTATAGG

GAATGAATTTCTAATGCATGATGGGAATGACAGATATGAGGAATATATAAAGGTGATTCT

TATATACATTGACATGGATCGGAATCTATTAACTTAAAGTTATTGGGGTCGTGAAATTTA

TTTAATTTTTCTAATTCGTGTCACATGTCACGACAATTAGTGGGG-AACATTTATGTATA

TAGAAATTTCAGAAATTTCTAGCAGCGTGATAAATATAAAGATTTTGGCACAATAAGTTT

TTGGATCATAATGAGTTT-CTATTTAATCAA-GGCA-TGTAA-TCTATTTCATTATTTAG

-TAGGG-AAACTGAAAACTTAGGGTTGCTATTTGTAGCTCCCACCCTTCTTAGTTCTTAC

TCTTTTCGATATTTTTTAAAAGTTTTA-TATAATACCT-AAATT-GCCCTCCTCCT----

----------------------------------------CACCCCATTGCTCTTCCGTT

GCTCCTCCATGTG-AGTTCTTGTCTTTTTTTTT-CTCTGGTAGCGTTTTGCTGCTCCTTT

TTTCACTCAAGTGTTGCCAATTAA-TTGACAAAAAATGGTTTCTGTTTCATATAGAAACT

ATGTTTTTGTTGTGTAGTCATACATTACGGAATCTAGTTT-CCATTAAATAAGTAAC--G

TGAAAAAAAA--TAAAAGGTGAAATATATATTGTTGGAAAAGAAGCTATGAGGTGCAAGA

ACCGATCACATGGAGAAGGCAATGAAAGACAAGGAGGAGCAATGGAAGA-----------

---------GAGAAAATGAGAAGATGGAAGGGATGTGAAAATGTTTGAAAAAAACGAGGT

GATCAGTTTTAAAATACGAATTTAGTATTTTCTTTTTAAGAAAATTCTTTCG-AAAGTCG

TGTTTTAAAACATGACTTTTATT-ATTTGAAGTCG---TGTTCTAAAACATGACTTA--T

TCATATCCTT-AATATTTTT---------AAAATTTATCCATTTGTAATATTTTTTAAAA

ATTGACCCATATATGTAAAATACCCGTCAAGATCTCTTTATTATTTTGAAAGCGAAAGCA

TATCACTTCAAACACAATGGAATCGAGGCTATTGACTAAGTATAAATAGAGAAGACTTCA

TATCGGGGTTCATAATTCATAACAAAGCAAACGAGTATATAAGAAAGCATAAGCCAAATT

TTGAGTAAACTAGTGTGCACACTATCCCATGCCTAGTGGAAGTAGGGATCCTCTCGTTGT

TGGGGGAGTAATTGGGGATGTATTGGATCCTTTTGAATATTCTATTCCTATGAGGGTTAC

CTACAATAACAGAGATGTCAGCAATGGATGTGAATTCAAACCCTCACAAGTTGTCAACCA

ACCAAGGGTAAATATCGGTGGTGATGACC

>CS03.00

TATTAGACTCCTAATTTAATATTCTTGTTTTATTATAATGCTGATAAGTCTTGTAAATAA

GGGTGAGAAGCACGAATAATTAGTTCATGAGATGTGTATAATCATTATCCTACACGACTT

ATCTTTGATATTTCACACAAGTCTTTCAATGTATAACAAAAACTTTTTAGATACATTTAG

ACTATAGAACTAACAAGTTATATTTTGAACC-AAAAAACAAAGAGAGAGAGAGAGGAACA

TAGAGAAAAGAAGATATGAGAGTTTTTTTTTTTTTTTTTTTCTAAAACAGAAAGAAACTC

ATTATATAATAAACAAATTACTTTGAGACAAATTAACGTATGTAATAAAACAAAATCAAA

GTGGTAATTAAAATTATTTAATGGTAAACAGTCTAATAGTTAAAATAAAAATGGAAATCA

CATAAATTTTGTAATTGGCCCATTAAAAACAACACTAAGCTTTTAATTTGATTTTGAAAT

TCAAAATAATTTTATTAAATCACAAAGTAAAAGGTTTACAAAGCCGATCATGACAGTGCA

TGTGGGAGGCAAATCGGCATATTTGCACTACAAAAGG-ACCCATGAAGTCTCTGAACATG

CACGCAACACTTTAATCTCTTATTAGTTACTTTGAAAGCTTATTTATATATATAGACACG

CGTAAAAACTTCTTAACCAAGATTTTTTT-ACGTGCTTCC-TTCGCGTTTAATTTGGACC

ATCAAACCGTGCTCAACAGATAAAGAAAAGGGTGCTTTTGATTCAAGATATTGGCC-GAA

AAACACAAGATAGATCCTT-CGATAGATTAAGCCACGCATGAAACGCGAATCCAAAGTGA

TGAAGAAGTGCAGATAGATATTCGTTCACCATATAGAGGAGAATATATCATTCCTACAAA

CAAAATTGATGATGTTTCTAGAATAGAAACGTTAGAATCGAATATTCATCTGTTCCGGGT

GGGGAAGGGCTACTGAAAAGCTGAACTTTTTTAAGAGTTCGAACTTCGAAAGTCAATCAA

TGATTCTTAATTGAGAGGGAAAAGCTATAAAGGACGAGAAGGGAAGGAAATGTTTATAGG

GAATGAATTTCTAATGCATGATGGGAATGACAGATATGAGGAATATATAAAGGTGATTCT

TATATACATTGACATGGATCGGAATCTATTAACTTAAAGTTATTGGGGTCGTGAAATTTA

TTTAATTTTTCTAATTCGTGTCACATGTCACGACAATTAGTGGGG-AACATTTATGTATA

TAGAAATTTCAGAAATTTCTAGCAGCGTGATAAATATAAAGATTTTGGCACAATAAGTTT

TTGGATCATAATGAGTTT-CTATTTAATCAA-GGCA-TGTAA-TCTATTTTATTATTTAG

-TAGGG-AAACTGAAAGCTTAGGGTTGCTATTTGTAGCTCCCACCCTTCTTAGTTCTTAC

TCTTTTCAATATTTTTTAAAAGTTTTA-TATAATACCT-AAATT-GCCCTCCTCCT----

----------------------------------------CACCCCATTGCTCTTCCGTT

GCTCCTCCATGTG-AGTTCTTGTCTTTTTTTTT-CTCTGGTAGCGTTTTGCTGCTCCTTT

TTTCACTCAAGTGTTGCCAATTAA-TTGGCAAAAAATGGTTTCTGTTTCATATAGAAACT

ATGTTTTTGTTGTGTAGTCATACATTACGGAATCTAGTTT-CCATTAAATAAGTAAC--G

TGAAAAAAAA--TAAAAGGTGAAATATATATTGTTGGAAAAGAAGCTATGAGGTGCAAGA

ACCGATCACATGGAGAAGGCAATGAAAGACAAGGAGGAGCAATGGAAGA-----------

---------GAGAAAATGAGAAGATGGAAGGGATGTGAAAATGTTTGAAAAAAACGAGGT

GATCAGTTTTAAAATACGAATTTAGTATTTTCTTTTTAAGAAAATTCTTTCG-AAAGTCG

TGTTTTAAAACATGACTTTTATT-ATTTGAAGTCG---TGTTCTAAAACATGACTTA--T

TCATATCCTT-AATATTTTT---------AAAATTTATCCATTTGTAATATTTTTTAAAA

ATTGACCCATATATGTAAAATACCCGTCAAGATCTCTTTATTATTTTGAAAGCGAAAGCA

TATCACTTCAAACACAATGGAATCGAGGCTATTGACTAAGTATAAATAGAGAAGACTTCA

TATCGGGGTTCATAATTCATAACAAAGCAAACGAGTATATAAGAAAGCATAAGCCAAATT

TTGAGTAAACTAGTGTGCACACTATCCCATGCCTAGTGGAAGTAGGGATCCTCTCGTTGT

TGGGGGAGTAATTGGGGATGTATTGGATCCTTTTGAATATTCTATTCCTATGAGGGTTAC

CTACAATAACAGAGATGTCAGCAATGGATGTGAATTCAAACCCTCACAAGTTGTCAACCA

ACCAAGGGTAAATATCGGTGGTGATGACC

>CS21.06

TATTAGACTCCTAATTTGATATTCTTGTTTTATTATAATGCTGATAAGTCTTGTAAATAA

GGGTGAGAAGCACGAATAATTAGTTCATGAGATGTGTATAATTATTATCCTACACGACTT

ATCTTTGATATTTCACACAAGTCTTTCAATGTATAACAAAAATTTTTTAGATACATTTAG

ACTATAGAACTAACAAGTTATATTTTGAACC-AAAGAACAAAGAGAGAGAG----GAACA

TAGAGAAAAGAAGATATGAGAGTTTTTTTTTTT--------CTAAAACAGAAAGAAACTC

ATTATATAATAAACAAATTACTTTGAGACAAATTAACGTATGTAATAAAACAAAATCAAA

GTGGTAATTAAAATTATTTAATGGTAAACAGTCTAATAGTTAAAATAAAAATGGAAATCA

CATAAATTTTGTAATTGGCCCATTAAAAACAACACAAAGCTTTTAATTTGATTTTGAAAT

TCAAAATAATTTTATTAAATCACAAAGTAAAAGGTTTACAAAGCCGATCATGACAGTGCA

TGTGGGAGGCAAATCGGCATATCTGCACTACAAAAGG-ACCCATGAAGTCTCTGAACATG

CACGCAACACTTTAATCTCTTATTAGTTACTTTGAAAGCTTATTTATATATATAGACACG

CGTAAAAACTTCTTAACCAAGATTTTTTT-ACGTGCTTCC-TTCGCGTTTAATTTGGACC

ATCAAACCGTGCTCAACAGATAAAGAAAAGGGTGCTTTTGATTCAAGATATTGGCC-GAA

AAACACAAGATAGATCCTT-CGATAGATTAAGCCACGCATGAAACGCGAATCCAAAGTGA

TGAAGAAGTGCAGATAGATATTCGTTCACCATATAGAGGAGAATATATCATTCCTACAAA

CAAAATTGATGATGTTTCTAGAATAGAAACGTTAGAATCGAATATTCATCTGTTCCGGGT

GGGGAAGGGCTACTGAAAAGCTGAACTTTTTTAAGAGTTCGAACTTCGAAAGTCAATCAA

TGATTCTTAATTGAGAGGGAAAAGCTATAAAGGACGAGAAGGGAAGGAAATGTTTATAGG

GAATGAATTTCTAATGCATGATGGGAATGACAGATATGAGGAATATATAAAGGTGATTCT

TATATACATTGACATGGATCGGAATCTATTAACTTAAAGTTATTGGGGTCGTGAAATTTA

TTTAATTTTTCTAATTCGTGTCACATGTCACGACAATTAGTGGGG-AACATTTATGTATA

TAGAAATTTCAGAAATTTCTAGCAGCGTGATAAATATAAAGATTTTGGCACAATAAGTTT

TTGGATCATAATGAGTTT-CTATTTAATCAA-GGCA-TGTAA-TCTATTTTATTATTTAG

-TAGGG-AAACTGAAAACTTAGGGTTGCTATTTGTAGCTCCCACCCTTCTTAGTTCTTAC

TCTTTTCAATATTTTTTATAAGTTTTA-TATAATACCT-AAATT-GCCCTCCTCCTCA-C

CCCCCCTGCTAATCTTCTTCCTCCTCACACATTGCTTCT-CACCCCATTGCTCTTCCGTT

GCTCCTCCATGTG-AGTTCTAGTCTTTTTTT---CTCTGGTAGCGTTT-GCTGCTCCTTT

TTTCACTCAAGTGTTGCCAATTAA-TTGACAAAAAATGGTTTCTGTTTCATATAGAAACT

ATGTTTTTGTTGTGTAGTCATACATTACGGAATCTAGTTT-CCATTAAATAAGTAACATG

TGAAAAAAAA--TAAAAGGTGAAATATATATTGTTGGAAAAGAAGCTATGAGGTGCAAGA

ACCGATCACATGGAGAAGGCAATGAAAGACAAGGAGGAGCAATGAAAGATAAGGAGGAGC

AATGAAAGAGAGAAAATGAGAAGATGGAAGGGATGTGAAAATGTTTGAAAAAGACGAGGT

GATCAGTTTTGAAATACGAATTTAGTATTTTCTTTTTAAGAAAATTCTTTCG-AAAGTCG

TGTTTTAAAACATGACTTTTATT-ATTTGAAGTCG---TGTTCTAAAACATGGCTTA--T

TCATATCCTT-AATATTTTT---------AAAATTTATCCATTTGTAATATTTTTTAAAA

ATTGACCCATATATGTAAAATACCCGTCAAGATCTCTTTATTATTTTGAAGGCGAAAGCA

TATCACTTCAAACACAATGGAATCGAGGCTATTGACTAAGTATAAATAGAGAAGACTTCA

TATCGGGGTTCATAATTCATAACAAAGCAAACGAGTATATAAGAAAGCATAAGCCAAATT

TTGAGTAAACTAGTGTGCACACTATCCCATGCCTAGTGGAAGTAGGGATCCTCTCGTTGT

TGGGGGAGTAATTGGGGATGTATTGGATCCTTTTGAATATTCTATTCCTATGAGGGTTAC

CTACAATAACAGAGATGTCAGCAATGGATGTGAATTCAAACCCTCACAAGTTGTCAACCA

ACCAAGGGTAAATATCGGTGGTGATGACC

>CS21.66

TATTAGACTCCTAATTTGATATTCTTGTTTTATTATAATGCTGATAAGTCTTGTAAATAA

GGGTGAGAAGCACGAATAATTAGTTCATGAGATGTGTATAATTATTATCCTACACGACTT

ATCTTTGATATTTCACACAAGTCTTTCAATGTATAACAAAAATTTTTTAGATACATTTAG

ACTATAGAACTAACAAGTTATATTTTGAACC-AAAAAACAAAGAGAGAGAG----GAACA

TAGAGAAAAGAAGATATGAGAGTTTTTTTTTTT--------CTAAAACAGAAAGAAACTC

ATTATATAATAAACAAATTACTTTGAGACAAATTAACGTATGTAATAAAACAAAATCAAA

GTGGTAATTAAAATTATTTAATGGTAAACAGTCTAATAGTTAAAATAAAAATGGAAATCA

CATAAATTTTGTAATTGGCCCATTAAAAACAACACAAAGCTTTTAATTTGATTTTGAAAT

TCAAAATAATTTTATTAAATCACAAAGTAAAAGGTTTACAAAGCCGATCATGACAGTGCA

TGTGGGAGGCAAATCGGCATATTTGCACTACAAAAGG-ACCCATGAAGTCTCTGAACATG

CACGCAACACTTTAATCTCTTATTAGTTACTTTGAAAGCTTATTTATATATATAGACACG

CGTAAAAACTTCTTAACCAAGATTTTTTT-ACGTGCTTCC-TTCGCGTTTAATTTGGACC

ATCAAACCGTGCTCAACAGATAAAGAAAAGGGTGCTTTTGATTCAAGATGTTGGCC-GAA

AAACACAAGATAGATCCTT-CGATAGATTAAGCCACGCATGAAACGCGAATCCAAAGTGA

TGAAGAAGTGCAGATGGATATTCGTTCACCATATAGAGGAGAATATATCATTCCTACAAA

CAAAATTGATGATGTTTCTAGAATAGAAACGTTAGAATCGAATATTCATCTGTTCCGGGT

GGGGAAGGGCTACTGAAAAGCTGAACTTTTTTAAGAGTTCGAACTTCGAAAGTCAATCAA

TGATTCTTAATTGAGAGGGAAAAGCTATAAAGGACGAGAAGGGAAGGAAATGTTTATAGG

GAATGAATTTCTAATGCATGATGGGAATGACAGATATGAGGAATATATAAAGGTGATTCT

TATATACATTGACATGGATCGGAATCTATTAACTTAAAGTTATTGGGGTCGTGAAATTTA

TTTAATTTTTCTAATTCGTGTCACATGTCACGACAATTAGTGGGG-AACATTTATGTATA

TAGAAATTTCAGAAATTTCTAGCAGCGTGATAAATATAAAGATTTTGGCGCAATAAGTTT

TTGGATCATAATGAGTTT-CTATTTAATCAA-GGCA-TGTAA-TCTATTTTATTATTTAG

-TAGGG-AAACTGAAAACTTAGGGTTGCTATTTGTAGCTCCCACCCTTCTTAGTTCTTAC

TCTTTTCAATATTTTTTATAAGTTTTA-TATAATACCT-AAATT-GCCCTCCTCCTCA-C

CCCCCCTGCTAATCTTCTTCCTCCTCACACATTGCTTCT-CACCC-ATTGCTCTTCCGTT

GCTCCTCCATGTG-AGTTCTAGTCTTTTTTTT--CTCT-GTAGCGTTTTGCTGCTCCTTT

TT-CACTCAAGTGTTGCCAATTAA-TTGACAAAAAATGGTTTCTGTTTCATATAGAAACT

ATGTTTTTGTTGTGTAGTCATACATTACGGAATCTAGTTT-CCATTAAATAAGTAACATG

TGAAAAAAAA--TAAAAGGTGAAATATATATTGTTGGAAAAGAAGCTATGAGGTGCAAGA

ACCGATCACATGGAGAAGGCAATGAAAGACAAGGAGGAGCAATGAAAGATAAGGAGGAGC

AATGAAAGAGAGAAAATGAGAAGATGGAAGGGATGTGAAAATGTTTGAAAAAGACGAGGT

GATCAGTTTTGAAATACGAATTTAGTATTTTCTTTTTAAGAAAATTCTTTCG-AAAGTCG

TGTTTTAAAACATGACTTTTATT-ATTTGAAGTCG---TGTTCTAAAACATGGCTTA--T

TCATATCCTT-AATATTTTT---------AAAATTTATCCATTTGTAATATTTTTTAAAA

ATTGACCCATATATGTAAAATACCCGTCAAGATCTCTTTATTATTTTGAAAGCGAAAGCA

TATCACTTCAAACACAATGGAATCGAGGCTATTGACTAAGTATAAATAGAGAAGACTTCA

TATCGGGGTTCATAATTCATAACAAAGCAAACGAGTATATAAGAAAGCATAAGCCAAATT

TTGAGTAAACTAGTGTGCACACTATCCCATGCCTAGTGGAAGTAGGGATCCTCTCGTTGT

TGGGGGAGTAATTGGGGATGTATTGGATCCTTTTGAATATTCTATTCCTATGAGGGTTAC

CTACAATAACAGAGATGTCAGCAATGGATGTGAATTCAAACCCTCACAAGTTGTCAACCA

ACCAAGGGTAAATATCGGTGGTGATGACC

>WM82

TATTAGACTCCTAATTTAATATTCTTGTTTTATTATAATGCTGATAAGTCTTGTAAATAA

GGGTGAGAAGCACGAATAATTAGTTCATGAGATGTGTATAATTATTATCCTACACGACTT

ATCTTTGATATTTCACACAAGTCTTTCAATGTATAACAAAAACTTTTTAGATACATTTAG

ACTATAGAACTAACAAGTTATATTTTGAACC-AAAAAACAAAGAGAGAGAGAGAGGAACA

TAGAGAAAAGAAGATATGAGAGTTTTTTTTTTTTTTTT---CTAAAACAGAAAGAAACTC

ATTATATAATAAACAAATTACTTTGAGACAAATTAACGTATGTAATAAAACAAAATCAAA

GTGGTAATTAAAATTATTTAATGGTAAACAGTCTAATAGTTAAAATAAAAATGGAAATCA

CATAAATTTTGTAATTGGCCCATTAAAAACAACACTAAGCTTTTAATTTGATTTTGAAAT

TCAAAATAATTTTATTAAATCACAAAGTAAAAGGTTTACAAAGCCGATCATGACAGTGCA

TGTGGGAGGCAAATCGGCATATTTGCACTACAAAAGG-ACCCATGAAGTCTCTGAACATG

CACGCAACACTTTAATCTCTTATTAGTTACTTTGAAAGCTTATTTATATATATAGACACG

CGTAAAAACTTCTTAACCAAGATTTTTTT-ACGTGCTTCC-TTCGCGTTTAATTTGGACC

ATCAAACCGTGCTCAACAGATAAAGAAAAGGGTGCTTTTGATTCAAGATATTGGCC-GAA

AAACACAAGATAGATCCTT-CGATAGATTAAGCCACGCATGAAACGCGAATCCAAAGTGA

TGAAGAAGTGCAGATAGATATTCGTTCACCATATAGAGGAGAATATATCATTCCTACAAA

CAAAATTGATGATGTTTCTAGAATAGAAACGTTAGAATCGAATATTCATCTGTTCCGGGT

GGGGAAGGGCTACTGAAAAGCTGAACTTTTTTAAGAGTTCGAACTTCGAAAGTCAATCAA

TGATTCTTAATTGAGAGGGAAAAGCTATAAAGGACGAGAAGGGAAGGAAATGTTTATAGG

GAATGAATTTCTAATGCATGATGGGAATGACAGATATGAGGAATATATAAAGGTGATTCT

TATATACATTGACATGGATCGGAATCTATTAACTTAAAGTTATTGGGGTCGTGAAATTTA

TTTAATTTTTCTAATTCGTGTCACATGTCACGACAATTAGTGGGG-AACATTTATGTATA

TAGAAATTTCAGAAATTTCTAGCAGCGTGATAAATATAAAGATTTTGGCACAATAAGTTT

TTGGATCATAATGAGTTT-CTATTTAATCAA-GGCA-TGTAA-TCTATTTTATTATTTAG

-TAGGG-AAACTGAAAACTTAGGGTTGCTATTTGTAGCTCCCACCCTTCTTAGTTCTTAC

TCTTTTCAATATTTTTTAAAAGTTTTA-TATAATACCT-AAATT-GCCCTCCTCCT----

----------------------------------------CACCCCATTGCTCTTCCGTT

GCTCCTCCATGTG-AGTTCTTGTCTTTTTTTTT-CTCTGGTAGCGTTTTGCTGCTCCTTT

TTTCACTCAAGTGTTGCCAATTAA-TTGACAAAAAATGGTTTCTGTTTCATATAGAAACT

ATGTTTTTGTTGTGTAGTCATACATTACGGAATCTAGTTT-CCATTAAATAAGTAAC--G

TGAAAAAAAA--TAAAAGGTGAAATATATATTGTTGGAAAAGAAGCTATGAGGTGCAAGA

ACCGATCACATGGAGAAGGCAATGAAAGACAAGGAGGAGCAATGGAAGA-----------

---------GAGAAAATGAGAAGATGGAAGGGATGTGAAAATGTTTGAAAAAAACGAGGT

GATCAGTTTTAAAATACGAATTTAGTATTTTCTTTTTAAGAAAATTCTTTCGGAAAGTCG

TGTTTTAAAACATGACTTTTATTTATTTGAAGTCG---TGTTCTAAAACATGACTTTATT

TCATATCCTTTAATATTTTTTAAAAATTTAAAATTTATCCATTTGTAATATTTTTTAAAA

ATTGACCCATATATGTAAAATACCCGTCAAGATCTCTTTATTATTTTGAAAGCGAAAGCA

TATCACTTCAAACACAATGGAATCGAGGCTATTGACTAAGTATAAATAGAGAAGACTTCA

TATCGGGGTTCATAATTCATAACAAAGCAAACGAGTATATAAGAAAGCATAAGCCAAATT

TTGAGTAAACTAGTGTGCACACTATCCCATGCCTAGTGGAAGTAGGGATCCTCTCGTTGT

TGGGGGAGTAATTGGGGATGTATTGGATCCTTTTGAATATTCTATTCCTATGAGGGTTAC

CTACAATAACAGAGATGTCAGCAATGGATGTGAATTCAAACCCTCACAAGTTGTCAACCA

ACCAAGGGTAAATATCGGTGGTGATGACC

>CS31.19

TATTAGACTCCTAATTTAATATTCTTGTTTTATTATAATGCTGATAAGTCTTGTAAATAA

GGGTGAGAAGCACGAATAATTAGTTCATGAGATGTGTATAATTATTATCCTACACGACTT

ATCTTTGATATTTCACACAAGTCTTTCAATGTATAACAAAAACTTTTTAGATACATTTAG

ACTATAGAACTAACAAGTTATATTTTGAACC-AAAAAACAAAGAGAGAGAGAGAGGAACA

TAGAGAAAAGAAGATATGAGAGTTTTTTTTTTTTTTTT---CTAAAACAGAAAGAAACTC

ATTATACAATAAACAAATTACTTTGAGACAAATTAACGTATGTAATAAAACAAAATCAAA

GTGGTAATTAAAATTATTTAATGGTAAACAGTCTAATAGTTAAAATAAAAATGGAAATCA

CATAAATTTTGTAATTGGCCCATTAAAAACAACACTAAGCTTTTAATTTGATTTTGAAAT

TCAAAATAATTTTATTAAATCACAAAGTAAAAGGTTTACAAAGCCGATCATGACAGTGCA

TGTGGGAGGCAAATCGGCATATTTGCACTACAAAAGG-ACCCATGAAGTCTCTGAACATG

CACGCAACACTTTAATCTCTTATTAGTTACTTTGAAAGCTTATTTATATATATAGACACG

CGTAAAAACTTCTTAACCAAGATTTTTTT-ACGTGCTTCC-TTCGCGTTTAACTTGGACC

ATCAAACCGTGCTCAACAGATAAAGAAAAGGGTGCTTTTGATTCAAGATATTGGCC-GAA

AAACACAAGATAGATCCCT-CGATAGATTAAGCCACGCATGAAACGCGAATCCAAAGTGA

TGAAGAAGTGCAGATAGATATTCGTTCACCATACAGAGGAGAATATATCATTCCTACAAA

CAAAATTGATGATGTTTCTAGAATAGAAACGTTAGAATCGAATATTCATCTGTTCCGGGT

GGGGAAGGGCTACTGAAAAGCTGAACTTTTTTAAGAGTTCGAACTTCGAAAGTCAATCAA

TGATTCTTAATTGAGAGGGAAAAGCTATAAAGGACGAGAAGGGAAGGAAATGTTTATAGG

GAATGAATTTCTAATGCATGATGGGAATGACAGATATGAGGAATATATAAAGGTGATTCT

TATATACATCGACATGGATCGGAATCTATTAACTTAAAGTTATTGGGGTCGTGAAATTTA

TTTAATTTTTCTAATTCGTGTCACATGTCACGACAATTAGTGGGG-AACATTTATGTATA

TAGAAATTTCAGAAATTTCTAGCAGCGTGATAAATATAAAGATTTTGGCACAATAAGTTT

TTGGATCATAATGAGTTT-CTATTTAATCAA-GGCA-TGTAA-TCTATTTTATTATTTAG

-TAGGG-AAACTGAAAACTTAGGGTTGCTATTTGTAGCTCCCACCCTTCTTAGTTCTTAC

TCTTTTCAATATTTTTTAAAAGTTTTA-TATAATACCT-AAATT-GCCCTCCTCCT----

----------------------------------------CACCCCATTGCTCTTCCGTT

GCTCCTCCATGTG-AGTTCTTGTCTTTTTTTTT-CTCTGGTAGCGTTTTGCTGCTCCTTT

TTTCACTCAAGTGTTGCCAATTAA-TTGACAAAAAATGGTTTCTGTTTCATATAGAAACT

ATGTTTTTGTTGTGTAGTCATACATTACGGAATCTAGTTT-CCATTAAATAAGTAAC--G

TGAAAAAAAA--TAAAAGGTGAAATATATATTGTTGGAAAAGAAGCTATGAGGTGCAAGA

ACCGATCACATGGAGAAGGCAATGAAAGACAAGGAGGAGCAATGGAAGA-----------

---------GAGGAAATGAGAAGATGGAAGGGATGTGAAAATGTTTGAAAAAAACGAGGT

GATCAGTTTTAAAATACGAATTTAGTATTTTCTTTTTAAGAAAATTCTTTCG-AAAGTCG

TGTTTTAAAACATGACTTTTATT-ATTTGAAGTCG---TGTTCTAAAACATGACTTA--T

TCATATCCTT-AATATTTTT---------AAAATTTATCCATTTGTAATATTTTTTAAAA

ATTGACCCATATATGTAAAATACCCGTCAAGATCTCTTTATTATTTTGAAAGCGAAAGCA

TATCACTTCAAACACAATGGAATCGAGGCTATTGACTAAGTATAAATAGAGAAGACTTCA

TATCGGGGTTCATAATTCATAACAAAGCAAACGAGTATATAAGAAAGCATAAGCCAAATT

TTGAGTAAACTAGTGTGCACACTATCCCATGCCTAGTGGAAGTAGGGATCCTCTCGTTGT

TGGGGGAGTAATTGGGGATGTATTGGATCCTTTTGAATATTCTATTCCTATGAGGGTTAC

CTACAATAACAGAGATGTCAGCAATGGATGTGAATTCAAACCCTCACAAGTTGTCAACCA

ACCAAGGGTAAATATCGGTGGTGATGACC

>CS58.12

TATTAGACTCCTAATTTAATATTCTTGTTTTATTATAATGCTGATAAGTCTTGTAAATAA

GGGTGAGAAGCACGAATAATTAGTTCATGAGATGCGTATAATTATTATCCTACACGACTT

ATCTTTGATATTTCACACAAGTCTTTCAACGTATAACAAAAACTTTTTAGATACATTTAG

ACTATAGAACTAACAAGTTATATTTTGAACC-AAAAAACAAAGAGAGAGAGAGAGGAACA

TAGAGAAAAGAAGATATGAGAGTTTTTTTTTTTTTTTT---CTAAAACAGAAAGAAACTC

ATTATATAATAAACAAATTACTTTGAGACAAATTAACGTATGTAATAAAACAAAATCAAA

GTGGTAATTAAAATTATTTAATGGTAAACAGTCTAATAGTTAAAATAAAAATGGAAATCA

CATAAATTTTGTAATTGGCCCATTAAAAACAACACTAAGCTTTTAATTTGATTTTGAAAT

TCAAAATAATTTTATTAAATCACAAAGTAAAAGGTTTACAAAGCCGATCATGACAGTGCA

TGTGGGAGGCAAATCGGCATATTTGCACTACAAAAGG-ACCCATGAAGTCTCTGAACATG

CACGCAACACTTTAATCTCTTATTAGTTACTTTGAAAGCTTATTTATATATATAGACACG

CGTAAAAACTTCTTAACCAAGATTCTTTT-ACGTGCTTCC-TTCGCGTTTAATTTGGACC

ATCAAACCGTGCTCAACAGATAAAGAAAAGGGTGCTTTTGATTCAAGATATTGGCC-GAA

AAACACAAGATAGATCCTT-CGATAGATTAAGCCACGCATGAAACGCGAATCCAAAGTGA

TGAAGAAGTGCAGATAGATATTCGTTCACCATATAGAGGAGAATATATCATTCCTACAAA

CAAAATTGATGATGTTTCTAGAATAGAAACGTTAGAATCGAATATTCATCTGTTCCGGGT

GGGGAAGGGCTACTGAAAAGCTGAACTTTTTTAAGAGTTCGAACTTCGAAAGTCAATCAA

TGATTCTTAATTGAGAGGGAAAAGCTATAAAGGACGAGAAGGGAAGGAAATGTTTATAGG

GAATGAATTTCTAATGCATGATGGGAATGACAGATATGAGGAATATATAAAGGTGATTCT

TATATACATTGACATGGATCGGAATCTATTAACTTAAAGTTATTGGGGTCGTGAAATTTA

TTTAATTTTTCTAATTCGTGTCACATGTCACGACAATTAGTGGGG-AACATTTATGTATA

TAGAAATTTCAGAAATTTCTAGCAGCGTGATAAATATAAAGATTTTGGCACAATAAGTTT

TTGGATCATAATGAGTTT-CTATTTAATCAA-GGCA-TGTAA-TCTATTTTATTATTTAG

-TAGGG-AAACTGAAAACTTAGGGTTGCTATTTGTAGCTCCCACCCTTCTTAGTTCTTAC

TCTTTTCAATATTTTTTAAAAGTTTTA-TATAATACCT-AAATT-GCCCTCCTCCT----

----------------------------------------CACCCCATTGCTCTTCCGTT

GCTCCTCCATGTG-AGTTCTTGTCTTTTTTTTT-CTCTGGTAGCGTTTTGCTGCTCCTTT

TTTCACTCAAGTGTTGCCAATTAA-TTGACAAAAAATGGTTTCTGTTTCATATAGAAACT

ATGTTTTTGTTGTGTAGTCATACATTACGGAATCTAGTTT-CCATTAAATAAGTAAC--G

TGAAAAAAAA--TAAAAGGTGAAATATATATTGTTGGAAAAGAAGCTATGAGGTGCAAGA

ACCGATCACATGGAGAAGGCAATGAAAGACAAGGAGGAGCAATGGAAGA-----------

---------GAGAAAATGAGAAGATGGAAGGGATGTGAAAATGTTTGAAAAAAACGAGGT

GATCAGTTTTAAAATACGAATTTAGTATTTTCTTTTTAAGAAAATTCTTTCG-AAAGTCG

TGTTTTAAAACATGACTTTTATT-ATTTGAAGTCG---TGTTCTAAAACATGACTTA--T

TCATATCCTT-AATATTTTT---------AAAATTTATCCATTTGTAATATTTTTTAAAA

ATTGACCCATATATGTAAAATACCCGTCAAGATCTCTTTATTATTTTGAAAGCGAAAGCA

CATCACTTCAAACACAATGGAATCGAGGCTATTGACTAAGTATAAATAGAGAAGACTTCA

TATCGGGGTTCATAATTCATAACAAAGCAAACGAGTATATAAGAAAGCATAAGCCAAATT

TTGAGTAAACTAGTGTGCACACTATCCCATGCCTAGTGGAAGTAGGGATCCTCTCGTTGT

TGGGGGAGTAATTGGGGATGTATTGGATCCTTTTGAATATTCTATTCCTATGAGGGTTAC

CTACAATAACAGAGATGTCAGCAATGGATGTGAATTCAAACCCTCACAAGTTGTCAACCA

ACCAAGGGTAAATATCGGTGGTGATGACC

>CS47.C1

TATTAGACTCCTAATTTAATATTCTTGTTTTATTATAATGCTGATAAGTCTTGTAAATAA

GGGTGAGAAGCACGAATAATTAGTTCATGAGATGTGTATAATTATTATCCTACACGACTT

ATCTTTGATATTTCACACAAGTCTTTCAATGTATAACAAAAACTTTTTAGATACATTTAG

ACTATAGAACTAACAAGTTATATTTTGAACC-AAAAAACAAAGAGAGAGAGAGAGGAACA

TAGAGAAAAGAAGATATGAGAGTTTTTTTTTTTTTTTT---CTAAAACAGAAAGAAACTC

ATTATATAATAAACAAATTACTTTGAGACAAATTAACGTATGTAATAAAACAAAATCAAA

GTGGTAATTAAAATTATTTAATGGTAAACAGTCTAATAGTTAAAATAAAAATGGAAATCA

CATAAATTTTGTAATTGGCCCATTAAAAACGACACTAAGCTTTTAATTTGATTTTGAAAT

TCAAAATAATTTTATTAAATCACAAAGTAAAAGGTTTACAAAGCCGATCATGACAGTGCA

TGTGGGAGGCAAATCGGCATATTTGCACTACAAAAGG-ACCCATGAAGTCTCTGAACATG

CACGCAGCACTTTAATCTCTTATTAGTTACTTTGAAAGCTTATTTATATATATAGACACG

CGTAAAAACTTCTTAACCAAGATTTTTTT-ACGTGCTTCC-TTCGCGTTTAATTTGGACC

ATCAAACCGTGCTCAACAGATAAAGAAAAGGGTGCTTTTGATTCAAGATATTGGCC-GAA

AAACACAAGATAGATCCTT-CGATAGATTAAGCCACGCATGAAACGCGAATCCAAAGTGA

TGAAGAAGTGCAGATAGATATTCGTTCACCATATAGAGGAGAATATATCATTCCTACAAA

CAGAATTGATGATGTTTCTAGAATAGAAACGTTAGAATCGAATATTCATCTGTTCCGGGT

GGGGAAGGGCTACTGAAAAGCTGAACTTTTTTAAGAGTTCGAACTTCGAAAGTCAATCAA

TGATTCTTAATTGAGAGGGAAAAGCTATAAAGGACGAGAAGGGAAGGAAATGTTTATAGG

GAATGAATTTCTAATGCATGATGGGAATGACAGATATGAGGAATATATAAAGGTGATTCT

TATATACATTGACATGGATCGGAATCTATTAACTTAAAGTTATTGGGGTCGTGAAATTTA

TTTAATTTTTCTAATTCGTGTCACATGTCACGACAATTAGTGGGG-AACATTTATGTATA

TAGAAATTTCAGAAATTTCTAGCAGCGTGATAAATATAAAGATTTTGGCACAATAAGTTT

TTGGATCATAATGAGTTT-CTATTTAATCAA-GGCA-TGTAA-TCTATTTTATTATTTAG

-TAGGG-AAACTGAAAACTTAGGGTTGCTATTTGTAGCTCCCACCCTTCTTAGTTCTTAC

TCTTTTCAATATTTTTTAAAAGTTTTA-TATAATACCT-AAATT-GCCCTCCTCCT----

----------------------------------------CACCCCATTGCTCTTCCGTT

GCTCCTCCATGTG-AGTTCTTGTCTTTTTTTTT-CTCTGGTAGCGTTTTGCTGCTCCCTT

TTTCACTCAAGTGTTGCCAATTAA-TTGACAAAAAATGGTTTCTGTTTCATATAGAAACT

ATGTTTTTGTTGTGTAGTCATACATTACGGAATCTAGTTT-CCATTAAATAAGTAAC--G

TGAAAAAAAA--TAAAAGGTGAAATATATATTGTTGGAAAAGAAGCTATGAGGTGCAAGA

ACCGATCACATGGAGAAGGCAATGAAAGACAAGGAGGAGCAATGGAAGA-----------

---------GAGAAAATGAGAAGATGGAAGGGATGTGAAAATGTTTGAAAAAAACGAGGT

GATCAGTTTTAAAATACGAATTTAGTATTTTCTTTTTAAGAAAATTCTTTCG-AAAGTCG

TGTTTTAAAACATGACTTTTATT-ATTTGAAGTCG---TGTTCTAAAACATGACTTA--T

TCATATCCTT-AATATTTTT---------AAAATTTATCCATTTGTAATATTTTTTAAAA

ATTGACCCATATATGTAAAATACCCGTCAAGATCTCTTTATTATTTTGAAAGCGAAAGCA

TATCACTTCAAACACAATGGAATCGAGGCTATTGACTAAGTATAAATAGAGAAGACTTCA

TATCGGGGTTCATAATTCATAACAAAGCAAACGAGTATATAAGAAAGCATAAGCCAAATT

TTGAGTAAACTAGTGTGCACACTATCCCATGCCTAGTGGAAGTAGGGATCCTCTCGTTGT

TGGGGGAGTAATTGGGGATGTATTGGATCCTTTTGAATATTCTATTCCTATGAGGGTTAC

CTACAATAACAGAGATGTCAGCAATGGATGTGAATTCAAACCCTCACAAGTTGTCAACCA

ACCAAGGGTAAATATCGGTGGTGATGACC

>CS01.02

TATTAGACCCCTAATTTAATATTCTTGTTTTATTATAATGCTGATAAGTCTTGTAAATAA

GGGTGAGAAGCACGAATAATTAGTTCATGAGATGTGTATAATTATTATCCTACACGACTT

ATCTTTGATATTTCACACAAGTCTTTCAATGTATAACAAAAACTTTTTAGATACATTTAG

ACTATAGAACTAACAAGTTATATTTTGAACC-AAAAAACAAAGAGAGGGAGAGAGGAACA

TAGAGAAAAGAAGATATGAGAGTTTTTTTTTTTTTTTT---CTAAAACAGAAAGAAACTC

ATTATATAATAAACAAATTACTTTGAGACAAATTAACGTATGTAATAAAACAAAATCAAA

GTGGTAATTAAAATTATTTAATGGTAAACAGTCTAATAGTTAAAATAAAAATGGAAATCA

CATAAATTTTGTAATTGGCCCATTAAAAACAACACTAAGCTTTTAATTTGATTTTGAAAT

TCAAAATAATTTTATTAAATCACAAAGTAAAAGGTTTACAAAGCCGATCATGACAGTGCA

TGTGGGAGGCAAATCGGCATATTTGCACTACAAAAGG-ACCCATGAAGTCTCTGAACATG

CACGCAACACTTTAGTCTCTTATTAGTTACTTTGAAAGCTTATTTATATATATAGACACG

CGTAAAAACTTCTTAACCAAGATTTTTTT-ACGTGCTTCC-TTCGAGTTTAATTTGGACC

ATCAAACCGTGCTCAACAGATAAAGAAAAGGGTGCTTTTGATTCAAGATATTGGCC-GAA

AAACACAAGATAGATCCTT-CGATAGATTAAGCCACGCATGAAACGCGAATCCAAAGTGA

TGAAGAAGTGCAGATAGATATTCGTTCACCATATAGAGGAGAATATATCATTCCTACAAA

CAAAATTGATGATGTTTCTAGAATAGAAACGTTAGAATCGAATATTCATCTGTTCCGGGT

GGGGAAGGGCTACTGAAAAGCTGAACTTTTTTAAGAGTTCGAACTTCGAAAGTCAATCAA

TGATTCTTAATTGAGAGGGAAAAGCTATAAAGGACGAGAAGGGAAGGAAATGTTTATAGG

GAATGAATTTCTAATGCATGATGGGAATGACAGATATGAGGAATATATAAAGGTGATTCT

TATATACATTGACATGGATCGGAATCTATTAACTTAAAGTTATTGGGGTCGTGAAATTTA

TTTAATTTTTCTAATTCGTGTCACATGTCACGACAATTAGTGGGG-AACATTTATGTATA

TAGAAATTTCAGAAATTTCTAGCAGCGTGATAAATATAAAGATTTTGGCACAATAAGTTT

TTGGATCATAATGAGTTT-CTATTTAATCAA-GGCA-TGTAA-TCTATTTTATTATTTAG

-TAGGG-AAACTGAAAACTTAGGGTTGCTATTTGTAGCTCCCACCCTTCTTAGTTCTTAC

TCTTTTCAATATTTTTTAAAAGTTTTA-TATAATACCT-AAATT-GCCCTCCTCCT----

----------------------------------------CACCCCATTGCTCTTCCGTT

GCTCCTCCATGTG-AGTTCTTGTCTTTTTTTTT-CTCTGGTAGCGTTTTGCTGCTCCTTT

TTTCACTCAAGTGTTGCCAATTAA-TTGACAAAAAATGGTTTCTGTTTCATATAGAAACT

ATGTTTTTGTTGTGTAGTCATACATTACGGAATCTAGTTT-CCATTAAATAAGTAAC--G

TGAAAAAAAA--TAAAAGGTGAAATATATATTGTTGGAAAAGAAGCTATGAGGTGCAAGA

ACCGATCACATGGAGAAGGCAATGAAAGACAAGGAGGAGCAATGGAAGA-----------

---------GAGAAAATGAGAAGATGGAAGGGATGTGAAAATGTTTGAAAAAAACGAGGT

GATCAGTTTTAAAATACGAATTTAGTATTTTCTTTTTAAGAAAATTCTTTCG-AAAGTCG

TGTTTTAAAACATGACTTTTATT-ATTTGAAGTCG---TGTTCTAAAACATGACTTA--T

TCATATCCTT-AATATTTTT---------AAAATTTATCCATTTGTAATATTTTTTAAAA

ATTGACCCATATATGTAAAATACCCGTCAAGATCTCTTTATTATTTTGAAAGCGAAAGCA

TATCACTTCAAACACAATGGAATCGAGGCTATTGACTAAGTATAAATAGAGAAGACTTCA

TATCGGGGTTCATAATTCATAACAAAGCAAACGAGTATATAAGAAAGCATAAGCCAAATT

TTGAGTAAACTAGTGTGCACACTATCCCATGCCTAGTGGAAGTAGGGATCCTCTCGTTGT

TGGGGGAGTAATTGGGGATGTATTGGATCCTTTTGAATATTCTATTCCTATGAGGGTTAC

CTACAATAACAGAGATGTCAGCAATGGATGTGAATTCAAACCCTCACAAGTTGTCAACCA

ACCAAGGGTAAATATCGGTGGTGATGACC

>CS36.49

TATTAGACTCCTAATTTAATATTCTTGTTTTATTATAATGCTGATAAGTCTTGTAAATAA

GGGTGAGAAGCACGAATAATTAGTTCATGAGATGTGTATAATTATTATCCTACACGACTT

ATCTTTGATATTTCACACAAGTCTTTCAATGTATAACAAAAACTTTTTAGATACATTTAG

ACTATAGAACTAACAAGTTATATTTTGAACC-AAAAAACAAAGAGAGAGAGAGAGGAACA

TAGAGAAAAGAAGATATGAGAGTTTTTTTTTTTTTTTT---CTAAAACAGAAAGAAACTC

ATTATATAATAAACAAATTACTTTGAGACAAATTAACGTATGTAATAAAACAAAATCAAA

GTGGTAATTAAAATTATTTAATGGTAAACAGTCTAATAGTTAAAATAAAAATGGAAATCA

CATAAATTTTGTAATTGGCCCATTAAAAACAACACTAAGCTTTTAATTTGATTTTGAAAT

TCAAAATAATTTTATTAAATCACAAAGTAAAAGGTTTACAAAGCCGATCATGACAGTGCA

TGTGGGAGGCAAATCGGCATATTTGCACTACAAAAGG-ACCCATGAAGTCTCTGAACATG

CACGCAACACTTTAATCTCTTATTAGTTACTTTGAAAGCTTATTTATATATATAGACACG

CGTAAAAACTTCTTAACCAAGATTTTTTT-ACGTGCTTCC-TTCGCGTTTAATTTGGACC

ATCAAACCGTGCTCAACAGACAAAGAAAAGGGTGCTTTTGATTCAAGATATTGGCC-GAA

AAACACAAGATAGATCCTT-CGATAGATTAAGCCACGCATGAAACGCGAATCCAAAGTGA

TGAAGAAGTGCAGATAGATATTCGTTCACCATATAGAGGAGAATATATCATTCCTACAAA

CAAAATTGATGATGTTTCTAGAATAGAAACGTTAGAATCGAATATTCATCTGTTCCGGGT

GGGGAAGGGCTACTGAAAAGCTGAACTTTTTTAAGAGTTCGAACTTCGAAAGTCAATCAA

TGATTCTTAATTGAGAGGGAAAAGCTATAAAGGACGAGAAGGGAAGGAAATGTTTATAGG

GAATGAATTTCTAATGCATGATGGGAATGACAGATATGAGGAATATATAAAGGTGATTCT

TATATACATTGACATGGATCGGAATCTATTAACTTAAAGTTATTGGGGTCGTGAAATTTA

TTTAATTTTTCTAATTCGTGTCACATGTCACGACAATTAGTGGGG-AACATTTATGTATA

TAGAAATTTCAGAAATTTCTAGCAGCGTGATAAATATAAAGATTTTGGCACAATAAGTTT

TTGGATCATAATGAGTTT-CTATTTAATCAA-GGCA-TGTAA-TCTATTTTATTATTTAG

-TAGGG-AAACTGAAAACTTAGGGTTGCTATTTGTAGCTCCCACCCTTCTTAGTTCTTAC

TCTTTTCAATATTTTTTAAAAGTTTTA-TATAATACCT-AAATT-GCCCTCCTCCT----

----------------------------------------CACCCCATTGCTCTTCCGTT

GCTCCTCCATGTG-AGTTCTTGTCTTTTTTTT--CTCTGGTAGCGTTTTGCTGCTCCTTT

TTTCACTCAAGTGTTGCCAATTAA-TTGACAAAAAATGGTTTCTGTTTCATATAGAAACT

ATGTTTTTGTTGTGTAGTCATACATTACGGAATCTAGTTT-CCATTAAATAAGTAAC--G

TGAAAAAAAA--TAAAAGGTGAAATATATATTGCTGGAAAAGAAGCTATGAGGTGCAAGA

ACCGATCACATGGAGAAGGCAATGAAAGACAAGGAGGAGCAATGGAAGA-----------

---------GAGAAAATGAGAAGATGGAAGGGATGTGAAAATGTTTGAAAAAAACGAGGT

GATCAGTTTTAAAATACGAATTTAGTATTTTCTTTTTAAGAAAATTCTTTCG-AAAGTCG

TGTTTTAAAACATGACTTTTATT-ATTTGAAGTCG---TGTTCTAAAACATGACTTA--T

TCATATCCTT-AATATTTTT---------AAAATTTATCCATTTGTAATATTTTTTAAAA

ATTGACCCATATATGTAAAATACCCGTCAAGATCTCTTTATTATTTTGAAAGCGAAAGCA

TATCACTTCAAACACAATGGAATCGAGGCTATTGACTAAGTATAAATAGAGAAGACTTCA

TATCGGGGTTCATAATTCATAACAAAGCAAACGAGTATATAAGAAAGCATAAGCCAAATT

TTGAGTAAACTAGTGTGCACACTATCCCATGCCTAGTGGAAGTAGGGATCCTCTCGTTGT

TGGGGGAGTAATTGGGGATGTATTGGATCCTTTTGAATATTCTATTCCTATGAGGGTTAC

CTACAATAACAGAGATGTCAGCAATGGATGTGAATTCAAACCCTCACAAGTTGTCAACCA

ACCAAGGGTAAATATCGGTGGTGATGACC

>CS04.13

TATTAGACTCCTAATTTAATATTCTTGTTTTATTATAATGCTGATAAGTCTTGTAAATAA

GGGTGAGAAGCACGAATAATTAGTTCATGAGATGTGTATAATTATTATCCTACACGACTT

ATCTTTGATATTTCACACAAGTCTCTCAATGTATAACAAAAACTTTTTAGATACATTTAG

ACTATAGAACTAACAAGTTATATTTTGAACC-AAAAAACAAAGAGAGAGAGAGAGGAACA

TAGAGAAAAGAAGATATGAGAGTTTTTTTTTTTTTTTT---CTAAAACAGAAAGAAACTC

ATTATATAATAAACAAATTACTTTGAGACAAATTAACGTATGTAATAAAACAAAATCAAA

GTGGTAATTAAAATTATTTAATGGTAAACAGTCTAATAGTTAAAATAAAAATGGAAATCA

CATAAATTTTGTAATTGGCCCATTAAAAACAACACTAAGCTTTTAATTTGATTTTGAAAT

TCAAAATAATTTTATTAAATCACAAAGTAAAAGGTTTACAAAGCCGATCATGACAGTGCA

TGTGGGAGGCAAATCGGCATATTTGCACTACAAAAGG-ACCCATGAAGTCTCTGAACATG

CACGCAACACTTTAATCTCTTATTAGTTACTTTGAAAGCTTATTTATATATATAGACACG

CGTAAAAACTTCTTAACCAAGATTTTTTT-ACGTGCTTCC-TTCGCGTTTAATTTGGACC

ATCAAACCGTGCTCAACAGATAAAGAAAAGGGTGCTTTTGATTCAAGATATTGGCC-GAA

AAACACAAGATAGATCCTT-CGATAGATTAAGCCACGCATGAAACGCGAATCCAAAGTGA

TGAAGAAGTGCAGATAGATATTCGTTCACCATATAGAGGAGAATATATCATTCCTACAAA

CAAAATTGATGATGTTTCTAGAATAGAAACGTTAGAATCGAATATTCATCTGTTCCGGGT

GGGGAAGGGCTACTGAAAAGCTGAACTTTTTTAAGAGTTCGAACTTCGAAAGTCAATCAA

TGATTCTTAATTGGGAGGGAAAAGCTATAAAGGACGAGAAGGGAAGGAAATGTTTGTAGG

GAATGAATTTCTAATGCATGATGGGAATGACAGATATGAGGAATATATAAAGGTGATTCT

TATATACATTGACATGGATCGGAATCTATTAACTTAAAGTTATTGGGGTCGTGAAATTTA

TTTAATTTTTCTAATTCGTGTCACATGTCACGACAATTAGTGGGG-AACATTTATGTATA

TAGAAATTTCAGAAATTTCTAGCAGCGTGATAAATATAAAGATTTTGGCACAATAAGTTT

TTGGATCATAATGAGTTT-CTATTTAATCAA-GGCA-TGTAA-TCTATTTTATTATTTAG

-TAGGG-AAACTGAAAACTTAGGGTTGCTATTTGTAGCTCCCACCCTTCTTAGTTCTTAC

TCTTTTCAATATTTTTTAAAAGTTTTA-TATAATACCT-AAATT-GCCCTCCTCCT----

----------------------------------------CACCCCATTGCTCTTCCGTT

GCTCCTCCATGTG-AGTTCTTGTCTTTTTTTTT-CTCTGGTAGCGTTTTGCTGCTCCTTT

TTTCACTCAAGTGTTGCCAATTAA-TTGACAAAAAATGGTTCCTGTTTCATATAGAAACT

ATGTTTTTGTTGTGTAGTCATACATTACGGAATCTAGTTT-CCATTAAATAAGTAAC--G

TGAAAAAAAA--TAAAAGGTGAAATATATATTGTTGGAAAAGAAGCTATGAGGTGCAAGA

ACCGATCACATGGAGAAGGCAATGAAAGACAAGGAGGAGCCATGGAAGA-----------

---------GAGAAAATGAGAAGATGGAAGGGATGTGAAAATGTTTGAAAAAAACGAGGT

GATCAGTTTTAAAATACGAATTTAGTATTTTCTTTTTAAGAAAATTCTTTCG-AAAGTCG

TGTTTTAAAACATGACTTTTATT-ATTTGAAGTCG---TGTTCTAAAACATGACTTA--T

TCATATCCTT-AATATTTTT---------AAAATTTATCCATTTGTAATATTTTTTAAAA

ATTGACCCATATATGTAAAATACCCGTCAAGATCTCTTTATTATTTTGAAAGCGAAAGCA

TATCACTTCAAACACAATGGAATCGAGGCTATTGACTAAGTATAAATAGAGAAGACTTCA

TATCGGGGTTCATAATTCATAACAAAGCAAACGAGTATATAAGAAAGCATAAGCCAAATT

TTGAGTAAACTAGTGTGCACACTATCCCATGCCTAGTGGAAGTAGGGATCCTCTCGTTGT

TGGGGGAGTAATTGGGGATGTATTGGATCCTTTTGAATATTCTATTCCTATGAGGGTTAC

CTACAATAACAGAGATGTCAGCAATGGATGTGAATTCAAACCCTCACAAGTTGTCAACCA

ACCAAGGGTAAATATCGGTGGTGATGACC

>CS31.17

TATTAGACTCCTAATTTAATATTCTTGTTTTATTATAATGCTGATAAGTCTTGTAAATAA

GGGTGAGAAGCACGAATAATTAGTTCATGAGATGTGTATAATTATTATCCTACACGACTT

ATCTTTGATATTTCACACAAGTCTTTCAATGTATAACAAAAACTTTTTAGATACATTTAG

ACTATAGAACTAACAAGTTATATTTTGAACC-AAAAAACAAAGAGAGAGAGAGAGGAACA

TAGAGAAAAGAAGATATGAGAGTTTTTTTTTTTTTTT----CTAAAACAGAAAGAAACTC

ATTATATAATAAACAAATTACTTTGAGACAAATTAACGTATGTAATAAAACAAAATCAAA

GTGGTAATTAAAATTATTTAATGGTAAACAGTCTAATAGTTAAAATAAAAATGGAAATCA

CATAAATTTTGTAATTGGCCCATTAAAAACAACACTAAGCTTTTAATTTGATTTTGAAAT

TCAAAATAATTTTATTAAATCACAAAGTAAAAGGTTTACAAAGCCGATCATGACAGTGCA

TGTGGGAGGCAAATCGGCATATTTGCACTACAAAAGG-ACCCATGAAGTCTCTGAACATG

CACGCAACACTTTAATCTCTTATTAGTTACTTTGAAAGCTTATTTATATATATAGACACG

CGTAAAAACTTCTTAACCAAGATTTTTTT-ACGTGCTTCC-TTCGCGTTTAATTTGGACC

ATCAGACCGTGCTCAACAGATAAAGAAAAGGGTGCTTTTGATTCAAGATATTGGCC-GAA

AAACACAAGATAGATCCTT-CGATAGATTAAGCCACGCATGAAACGCGAATCCAAAGTGA

TGAGGAAGTGCAGATAGATATTCGTTCACCATATAGAGGAGAATATATCATTCCTACAAA

CAAAATTGATGATGTTTCTAGAATAGAAACGTTAGAATCGAATATTCATCTGTTCCGGGT

GGGGAAGGGCTACTGAAAAGCTGAACTTTTTTAAGAGTTCGAACTTCGAAAGTCAATCAA

TGATTCTTAATTGAGAGGGAAA-GCTATAAAGGACGAGAAGGGAAGGAAATGTTTATAGG

GAATGAATTTCTAATGCATGATGGGAATGACAGATATGAGGAATATATAAAGGTGATTCT

TATATACATTGACATGGATCGGAATCTATTAACTTAAAGTTATTGGGGTCGTGAAATTTA

TTTAATTTTTCTAATTCGTGTCACATGTCACGACAATTAGTGGGG-AACATTTATGTATA

TAGAAATTTCGGAAATTTCTAGCAGCGTGATAAATATAAAGATTTTGGCACAATAAGTTT

TTGGATCATAATGAGTTT-CTATTTAATCAA-GGCA-TGTAA-TCTATTTTATTATTTAG

-TAGGG-AAACTGAAAACTTAGGGTTGCTATTTGTAGCTCCCACCCTTCTTAGTTCTTAC

TCTTTTCAATATTTTTTAAAAGTTTTA-TATAATACCT-AAATT-GCCCTCCTCCT----

----------------------------------------CACCCCATTGCTCTTCCGTT

GCTCCTCCATGTG-AGTTCTTGTCTTTTTTTTT-CTCTGGTAGCGTTTTGCTGCTCCTTT

TTTCACTCAAGTGTTGCCAATTAA-TTGACAAAAAATGGTTTCTGTTTCATATAGAAACT

ATGTTTTTGTTGTGTAGTCATACATTACGGAATCTAGTTT-CCATTAAATAAGTAAC--G

TGAAAAAAAA--TAAAAGGTGAAATATATATTGTTGGAAAAGAAGCTATGAGGTGCAAGA

ACCGATCACATGGAGAAGGCAATGAAAGACAAGGAGGAGCAATGGAAGA-----------

---------GAGAAAATGAGAAGATGGAAGGGATGTGAAAATGTTTGAAAAAAACGAGGT

GATCAGTTTTAAAATACGAATTTAGTATTTTCTTTTTAAGAAAATTCTTTCG-AAAGTCG

TGTTTTAAAACATGACTTTTATT-ATTTGGAGTCG---TGTTCTAAAACTTGACTTA--T

TCATATCCTT-AATATTTTT---------AAAATTTATCCATTTGTAATATTTTTTAAAA

ATTGACCCATATATGTAAAATACCCGTCAAGATCTCTTTATTATTTTGAAAGCGAAAGCA

TATCACTTCAAACACAATGGAATCGAGGCTATTGACTAAGTATAAATAGAGAAGACTTCA

CATCGGGGTTCATAATTCATAACAAAGCAAACGAGTATATAAGAAAGCATAAGCCAAATT

TTGAGTAAACTAGTGTGCACACTATCCCATGCCTAGTGGAAGTAGGGATCCTCTCGTTGT

TGGGGGAGTAATTGGGGATGTATTGGATCCTTTTGAATATTCTATTCCTATGAGGGTTAC

CTACAATAACAGAGATGTCAGCAATGGATGTGAATTCAAACCCTCACAAGTTGTCAACCA

ACCAAGGGTAAATATCGGTGGTGATGACC

>CS39.80

TATTAGACTCCTAATTTAATATTCTTGTTTTATTATAATGCTGATAAGTCTTGTAAACAA

GGGTGAGAAGCACGAATAATTAGTTCATGAGATGTGTATAATTATTATCCTACACGACTT

ATCTTTGATATTTCACACAAGTCTTTCAATGTATAACAAAAACTTTTTAGATACATTTAG

ACTATAGAACTAACAAGTTATATTTTGAACC-AAAAAACAAAGAGAGAGAGAGAGGAACA

TAGAGAAAAGAAGATATGAGAGTTTTTTTTTTTTTTTT---CTAAAACAGAAAGAAACTC

ATTATATAATAAACAAATTACTTTGAGACAAATTAACGTATGTAATAAAACAAAATCAAA

GTGGTAATTAAAATTATTTAATGGTAAACAGTCTAATAGTTAAAATAAAAATGGAAATCA

CATAAATTTTGTAATTGGCCCATTAAAAACAACACTAAGCTTTTAATTTGATTTCGAAAT

TCAAAATAATTTTATTAAATCACAAAGTAAAAGGTTTACAAAGCCGATCATGACAGTGCG

CGTGGGAGGCAAATCGGCATATTTGCACTACAAAAGG-ACCCATGAAGTCTCTGAACATG

CACGCAACACTTTAATCTCTTATTAGTTACTTTGAAAGCTTATTTATATATATAGACACG

CGTAAAAACTTCTTAACCAAGATTTTTTT-ACGTGCTTCC-TTCGCGTTTAATTTGGACC

ATCAAACCGTGCTCAACAGATAAAGAAAAGGGTGCTTTTGATTCAAGATATTGGCC-GAA

AAACACAAGATAGATCCTT-CGATAGATTAAGCCACGCATGAAACGCGAATCCAAAGTGA

TGAAGAAGTGCAGATAGATATTCGTTCACCATATAGAGGAGAATATATCATTCCTACAAA

CAAAATTGATGGTGTTTCTAGAATAGAAACGTTAGAATCGAATATTCATCTGTTCCGGGT

GGGGAAGGGCTACTGAAAAGCTGAACTTTTTTAAGAGTTCGAACTTCGAAAGTCAATCAA

TGATTCTTAATTGAGAGGGAAAAGCTATAAAGGACGAGAAGGGAAGGAAATGTTTATAGG

GAATGAATTTCTAATGCATGATGGGAATGACAGATATGAGGAATATATAAAGGTGATTCT

TATATACATTGACATGGATCGGAATCTATTAACTTAAAGTTATTGGGGTCGTGAAATTTA

TTTAATTTTTCTAATTCGTGTCACATGTCACGACAATTAGTGGGG-AACATTTATGTATA

TAGAAATTTCAGAAATTTCTAGCAGCGTGATAAATATAAAGATTTTGGCACAATAAGTTT

TTGGATCATAATGAGTTT-CTATTTAATCAA-GGCA-TGTAA-TCTATTTTATTATTTAG

-TAGGG-AAACTGAAAACTTAGGGTTGCTATTTGTAGCTCCCACCCTTCTTAGTTCTTAC

TCTTTTCAATATTTTTTAAAAGTTTTA-TATAATACCT-AAATT-GCCCTCCTCCT----

----------------------------------------CACCCCATTGCTCTTCCGTT

GCTCCTCCATGTG-AGTTCTTGTCTTTTTTTTT-CTCTGGTAGCGTTTTGCTGCTCCTTT

TTTCACTCAAGTGTTGCCAATTAA-TTGACAAAAAATGGTTTCTGTTTCATATAGAAACT

ATGTTTTTGTTGTGTAGTCATACATTACGGAATCTAGTTT-CCATTAAATAAGTAAC--G

TGAAAAAAAA--TAAAAGGTGAAATATATATTGTCGGAAAAGAAGCTATGAGGTGCAAGA

ACCGATCACATGGAGAAGGCAATGAAAGACAAGGAGGAGCAATGGAAGA-----------

---------GAGAAAATGAGAAGATGGAAGGGATGTGAAAATGTTTGAAAAAAACGAGGT

GATCAGTTTTAAAATACGAATTTAGTATTTTCTTTTTAAGAAAATTCTTTCG-AAAGTCG

TGTTTTAAAACATGACTTTTATT-ATTTGAAGTCG---TGTTCTAAAACATGACTTA--T

TCATATCCTT-AATATTTTT---------AAAATTTATCCATTTGTAATATTTTTTAAAA

ATTGACCCATATATGTAAAATACCCATCAAGATCTCTTTATTATTTTGAAAGCGAAAGCA

TATCACTTCAAACACAATGGAATCGAGGCTATTGACTAAGTATAAATAGAGAAGACTTCA

TATCGGGGTTCATAATTCATAACAAAGCAAACGAGTATATAAGAAAGCATAAGCCAAATT

TTGAGTAAACTAGTGTGCACACTATCCCATGCCTAGTGGAAGTAGGGATCCTCTCGTTGT

TGGGGGAGTAATTGGGGATGTATTGGATCCTTTTGAATATTCTATTCCTATGAGGGTTAC

CTACAATAACAGGGATGTCAGCAATGGATGTGAATTCAAACCCTCACGAGTTGTCAACCA

ACCAAGGATAAATATCGGTGGTGATGACC

>J3.36

TATTAGACTCCTAATTTAATATTCTTGTTTTATTATAATGCTGATAAGTCTTGTAAATAA

GGGTGAGAAGCACGAATAATTAGTTCATGAGATGTGTATAATTATTATCCTACACGACTT

ATCTTTGATATTTCACACAAGTCTTTCAATGTATAACAAAAACTTTTTAGATACATTTAG

ACTATAGAACTAACAAGTTATATTTTGAACC-AAAAAACAAAGAGAGAGAGAGAGGAACA

TAGAGAAAAGAAGATATGAGAGTTTTTTTTTTTTTTTT---CTAAAACAGAAAGAAACTC

ATTATATAATAAACAAATTACTTTGAGACAAATTAACGTATGTAATAAAACAAAATCAAA

GTGGTAATTAAAATTATTTAATGGTAAACAGTCTAATAGTTAAAATAAAAATGGAAATCA

CATAAATTTTGTAATTGGCCCATTAAAAACAACACTAAGCTTTTAATTTGATTTTGAAAT

TCAAAATAATTTTATTAAATCACAAAGTAAAAGGTTTACAAAGCCGATCATGACAGTGCA

TGTGGGAGGCAAATCGGCATATTTGCACTACAAAAGG-ACCCATGAAGTCTCTGAACATG

CACGCAACACTTTAATCTCTTATTAGTTACTTTGAAAGCTTATTTATATATATAGACACG

CGTAAAAACTTCTTAACCAAGATTTTTTT-ACGTGCTTCC-TTCGCGTTTAATTTGGACC

ATCAAACCGTGCTCAACAGATAAAGAAAAGGGTGCTTTTGATTCAAGATATTGGCC-GAA

AAACACAAGATAGATCCTT-CGATAGATTAAGCCACGCATGAAACGCGAATCCAAAGTGA

TGAAGAAGTGCAGATAGATATTCGTTCACCATGTAGAGGAGAATATATCATTCCTACAAA

CAAAATTGATGATGTTTCTAGAATAGAAACGTTAGAATCGAATATTCATCTGTTCCGGGT

GGGGAAGGGCTACTGAAAAGCTGAACTTTTTTAAGAGTTCGAACTTCGAAAGTCAATCAA

TGATTCTTAATTGAGAGGGAAAAGCTATAAAGGACGAGAAGGGAAGGAAATGTTTATAGG

GAATGAATTTCTAATGCATGATGGGAATGACAGATATGAGGAATATATAAAGGTGATTCT

TATATACATTGACATGGATCGGAATCTATTAACTTAAAGTTATTGGGGTCGTGAAATTTA

TTTAATTTTTCTAATTCGTGTCACATGTCACGACAATTAGTGGGG-AACATTTATGTATA

TAGAAATTTCAGAAATTTCTAGCAGCGTGATAAATATAAAGATTTTGGCACAATAAGTTT

TTGGATCATAATGAGTTT-CTATTTAATCAA-GGCA-TGTAA-TCTATTTTATTATTTAG

-TAGGG-AAACTGAAAACTTAGGGTTGCTATTTGTAGCTCCCACCCTTCTTAGTTCTTAC

TCTTTTCAATATTTTTTAAAAGTTTTA-TATAATACCT-AAATT-GCCCTCCTCCT----

----------------------------------------CACCCCATTGCTCTTCCGTT

GCTCCTCCATGTG-AGTTCTTGTCTTTTTTTT--CTCTGGTAGCGTTTTGCTGCTCCTTT

TTTCACTCAAGTGTTGCCAATTAA-TTGACAAAAAATGGTTTCTGTTTCATATAGAAACC

ATGTTTTTGTTGTGTAGTCATACATTACGGAATCTAGTTT-CCATTAAATAAGTAAC--G

TGAAAAAAAA--TAAAAGGTGAAATATATATTGTTGGAAAAGAAGCTATGAGGTGCGAGA

ACCGATCACATGGAGAAGGCAATGAAAGACAAGGAGGAGCAATGGAAGA-----------

---------GAGAAAATGAGAAGATGGAAGGGATGTGAAAATGTTTGAAAAAAACGAGGT

GATCAGTTTTAAAATACGAATTTAGTATTTTCTTTTTAAGAAAATTCTTTCG-AAAGTCG

TGTTTTAAAACATGACTTTTATT-ATTTGAAGTCG---TGTTCTAAAACATGACTTA--T

TCATATCCTT-AATATTTTT---------AAAATTTATCCATTTGTAATATTTTTTAAAA

ATTGACCCATATATGTAAAATACCCGTCAAGATCTCTTTATTATTTTGAAAGCGAAAGCA

TATCACTTCAAACACAATGGAATCGAGGCTATTGACTAAGTATAAATAGAGAAGACTTCA

TATCGGGGTTCATAATTCATAACAAAGCAAACGAGTATATAAGAAAGCATAAGCCAAATT

TTGAGTAAACTAGTGTGCACACTATCCCATGCCTAGTGGAAGTAGGGATCCTCTCGTTGT

TGGGGGAGTAATTGGGGATGTATTGGATCCTTTTGAATATTCTATTCCTATGAGGGTTAC

CTACAATAACAGAGATGTCAGCAATGGATGTGAATTCAAACCCTCACAAGTTGTCAACCA

ACCAAGGGTAAATATCGGTGGTGATGACC

>CS33.25

TATTAGACTCCTAATTTAATATTCTTGTTTTATTATAATGCTGATAAGTCTTGTAAATAA

GGGTGAGAAGCACGAATAATTAGTTCATGAGATGTGTATAATTATTATCCTACACGACTT

ATCTTTGATATTTCACACAAGTCTTTCAATGTATAACAAAAACTTTTTAGATACATTTAG

ACTATAGAACTAACAAGTTATATTTTGAACC-AAAAAACAAAGAGAGAGAGAGAGGAACA

TAGAGAAAAGAAGATATGAGAGTTTTTTTTTTTTTT-----CTAAAACAGAAAGAAACTC

ATTATATAATAAACAAATTACTTTGAGACAAATTAACGTATGTAATAAAACAAAATCAAA

GTGGTAATTAAAATTATTTAATGGTAAACAGTCTAATAGTTAAAATAAAAATGGAAATCA

CATAAATTTTGTAATTGGCCCATTAAAAACAACACTAAGCTTTTAATTTGGTTTTGAAAT

TCAAAATAATTTTATTAAATCACAAAGTAAAAGGTTTACAAAGCCGATCATGACAGTGCA

TGTGGGAGGCAAATCGGCATATTTGCACTACAGAAGG-ACCCATGAAGTCTCTGAACATG

CACGCAACACTTTAATCTCTTATTAGTTACTTTGAAAGCTTATTTATATATATAGACACG

CGTAAAAACTTCTTAACCAAGATTTTTTT-ACGTGCTTCC-TTCGCGTTTAATTTGGACC

ATCAAACCGTGCTCAACAGATAAAGAAAAGGGTGCTTTTGATTCAAGATATTGGCC-GAA

AAACACAAGATAGATCCTT-CGATAGATTAAGCCACGCATGAAACGCGAATCCAAAGTGA

TGAAGAAGTGCAGATAGATATTCGTTCACCATATAGAGGAGAATATATCATTCCTACAAA

CAAAATTGATGATGTTTCTAGAATAGAAACGTTAGAATCGAATATTCATCTGTTCCGGGT

GGGGGAGGGCTACTGAAAAGCTGAACTTTTTTAAGAGTTCGAACTTCGAAAGTCAATCAA

TGATTCTTAATTGAGAGGGAAAAGCTATAAAGGACGAGAAGGGAAGGAAATGTTTATAGG

GAATGAATTTCTAATGCATGATGGGAATGACAGATATGAGGAATATATAAAGGTGATTCT

TATATACATTGACATGGATCGGAATCTATTAACTTAAAGTTATTGGGGTCGTGAAATTTA

TTTAATTTTTCTAATTCGTGTCACATGTCACGACAATTAGTGGGG-AACATTTATGTATA

TAGAAATTTCAGAAATTTCTAGCAGCGTGATAAATATAAAGATTTTGGCACAATAAGTTT

TTGGATCATAATGAGTTT-CTATTTAATCAA-GGCA-TGTAA-TCTATTTTATTATTTAG

-TAGGG-AAACTGAAAACTTAGGGTTGCTATTTGTAGCTCCCACCCTTCTTAGTTCTTAC

TCTTTTCAATATTTTTTAAAAGTTTTA-TATAATACCT-AAATT-GCCCTCCTCCT----

----------------------------------------CACCCCATTGCTCTTCCGTT

GCTCCTCCATGTG-AGTTCTG-TCTTTTTTT---CTCTGGTAGCGTTTTGCTGCTCCTTT

TTTCACTCAAGTGTTGCCAATTAA-TTGACAAAAAATGGTTTCTGTTTCATATAGAAACT

ATGTTTTTGTTGTGTAGTCATACATTACGGAATCTAGTTT-CCATTAAATAAGTAAC--G

TGAAAAAAAA--TAAAAGGTGAAATATATATTGTTGGAAAAGAAGCTATGAGGTGCAAGA

ACCGATCACATGGAGAAGGCAATGAAAGACAAGGAGGAGCAATGGAAGA-----------

---------GAGAAAATGAGAAGATGGAAGGGATGTGAAAATGTTTGAAAAAAACGAGGT

GATCAGTTTTAAAATACGAATTTAGTATTTTCTTTTTAAGAAAATTCTTTCG-AAAGTCG

TGTTTTAAAACATGACTTTTATT-ATTTGAAGTCG---TGTTCTAAAACATGACTTA--T

TCATATCCTT-AATATTTTT---------AAAATTTATCCATTTGTAATATTTTTTAAAA

ATTGACCCATATATGTAAAATACCCGTCAAGATCTCTTTATTATTTTGAAAGCGAAAGCA

TATCACTTCAAACACAATGGAATCGAGGCTATTGACTAAGTATAAATAGAGAAGACTTCA

TATCGGGGTTCATAATTCATAACAAAGCAAACGAGTATATAAGAAAGCATAAGCCAAATT

TTGAGTAAACTAGTGTGCACACTATCCCATGCCTAGTGGAAGTAGGGATCCTCTCGTTGT

TGGGGGAGTAATTGGGGATGTATTGGATCCTTTTGAATATTCTATTCCTATGAGGGTTAC

CTACAATAACAGAGATGTCAGCAATGGATGTGAATTCAAACCCTCACAAGTTGTCAACCA

ACCAAGGGTAAATATCGGTGGTGATGACC

>CS50.F1

TATTAGACTCCTAATTTAATATTCTTGTTTTATTATAATGCTGATAAGTCTTGTAAATAA

GGGTGAGAAGCACGAATAATTAGTTCATGAGATGTGTATAATTATTATCCTACACGACTT

ATCTTTGATATTTCACACAAGTCTTTCAATGTATAACAAAAACTTTTTAGATACATTTAG

ACTATAGAACTAACAAGTTATATTTTGAACC-AAAAAACAAAGAGAGAGAGAGAGGAACA

TAGAGAAAAGAAGATATGAGAGTTTTTTTTTTTTTTTT---CTAAAACAGAAAGAAACTC

ATTATATAATAAACAAATTACTTTGAGACAAATTAACGTATGTAATAAAACAAAATCAAA

GTGGTAATTAAAATTATTTAATGGTAAACAGTCTAATAGTTAAAATAAAAATGGAAATCA

CATAAATTTTGTAATTGGCCCATTAAAAACAACACTAAGCTTTTAATTTGATTTTGAAAT

TCAAAATAATTTTATTAAATCACAAAGTAAAAGGTTTACAAAGCCGATCATGACAGTGCA

TGTGGGAGGCAAATCGGCATATTTGCACTACAAAAGG-ACCCATGAAGTCTCTGAACATG

CACGCAACACTTTAATCTCTTATTAGTTACTTTGAAAGCTTATTTATATATATAGACACG

CGTAAAAACTTCTTAACCAAGATTTTTTT-ACGTGCTTCC-TTCGCGTTTAATTTGGACC

ATCAAACCGTGCTCAACAGATAAAGAAAAGGGTGCTTTTGATTCAAGATATTGGCC-GAA

AAACACAAGATAGATCCTT-CGATAGATTAAGCCACGCATGAAACGCGAATCCAAAGTGA

TGAAGAAGTGCAGATAGATATTCGTTCACCATATAGAGGAGAATATATCATTCCTACAAA

CAAAATTGATGATGTTTCTAGAATAGAAACGTTAGAATCGAATATTCATCTGTTCCGGGT

GGGGAAGGGCTACTGAAAAGCTGAACTTTTTTAAGAGTTCGAACTTCGAAAGTCAATCAA

TGATTCTTAATTGAGAGGGAAAAGCTATAAAGGACGAGAAGGGAAGGAAATGTTTATAGG

GAATGAATTTCTAATGCATGATGGGAATGACAGATATGAGGAATATATAAAGGTGATTCT

TATATACATTGACATGGATCGGAATCTATTAACTTAAAGTTATTGGGGTCGTGAAATTTA

TTTAATTTTTCTAATTCGTGTCACATGTCACGACAATTAGTGGGG-AACATTTATGTATA

TAGAAATTTCAGAAATTTCTAGCAGCGTGATAAATATAAAGATTTTGGCACAATAAGTTT

TTGGATCATAATGAGTTT-CTATTTAATCAA-GGCA-TGTAA-TCTATTTTATTATTTAG

-TAGGG-AAACTGAAAACTTAGGGTTGCTATTTGTAGCTCCCACCCTTCTTAGTTCTTAC

TCTTTTCAATATTTTTTAAAAGTTTTA-TATAATACCT-AAATT-GCCCTCCTCCT----

----------------------------------------CACCCCATTGCTCTTCCGTT

GCTCCTCCATGTG-AGTTCTTGTCTTTTTTTTT-CTCTGGTAGCGTTTTGCTGCTCCTTT

TTTCACTCAAGTGTTGCCAATTAA-TTGACAAAAAATGGTTTCTGTTTCATATAGAAACT

ATGTTTTTGTTGTGTAGTCATACATTACGGAATCTAGTTT-CCATTAAATAAGTAAC--G

TGAAAAAAAA--TAAAAGGTGAAATATATATTGTTGGAAAAGAAGCTATGAGGTGCAAGA

ACCGATCACATGGAGAAGGCAATGAAAGACAAGGAGGAGCAATGGAAGA-----------

---------GAGAAAATGAGAAGATGGAAGGGATGTGAAAATGTTTGAAAAAAACGAGGT

GATCAGTTTTAAAATACGAATTTAGTATTTTCTTTTTAAGAAAATTCTTTCG-AAAGTCG

TGTTTTAAAACATGACTTTTATT-ATTTGAAGTCG---TGTTCTAAAACATGACTTA--T

TCATATCCTT-AATATTTTT---------AAAATTTATCCATTTGTAATATTTTTTAAAA

ATTGACCCATATATGTAAAATACCCGTCAAGATCTCTTTATTATTTTGAAAGCGAAAGCA

TATCACTTCAAACACAATGGAATCGAGGCTATTGACTAAGTATAAATAGAGAAGACTTCA

TATCGGGGTTCATAATTCATAACAAAGCAAACGAGTATATAAGAAAGCATAAGCCAAATT

TTGAGTAAACTAGTGTGCACACTATCCCATGCCTAGTGGAAGTAGGGATCCTCTCGTTGT

TGGGGGAGTAATTGGGGATGTATTGGATCCTTTTGAATATTCTATTCCTATGAGGGTTAC

CTACAATAACAGAGATGTCAGCAATGGATGTGAATTCAAACCCTCACAAGTTGTCAACCA

ACCAAGGGTAAATATCGGTGGTGATGACC

>CS54.K8

TATTAGACTCCTAATTTAATATTCTTGTTTTATTATAATGCTGATAAGTCTTGTAAATAA

GGGTGAGAAGCACGAATAATTAGTTCATGAGATGTGTATAATTATTATCCTACACGACTT

ATCTTTGATATTTCACACAAGTCTTTCAATGTATAACAAAAACTTTTTAGATACATTTAG

ACTATAGAACTAACAAGTTATATTTTGAACC-AAAAAACAAAGAGAGAGAGAGAGGAACA

TAGAGAAAAGAAGATATGAGAGTTTTTTTTTTTTTTT----CTAAAACAGAAAGAAACTC

ATTATATAATAAACAAATTACTTTGAGACAAATTAACGTATGTAATAAAACAAAATCAAA

GTGGTAATTAAAATTATTTAATGGTAAACAGTCTAATAGTTAAAATAAAAATGGAAATCA

CATAAATTTTGTAATTGGCCCATTAAAAACAACACTAAGCTTTTAATTTGATTTTGAAAT

TCAAAATAATTTTATTAAATCACAAAGTAAAAGGTTTACAAAGCCGATCATGACAGTGCA

TGTGGGAGGCAAATCGGCATATTTGCACTACAAAAGG-ACCCATGAAGTCTCTGAACATG

CACGCAACACTTTAATCTCTTATTAGTTACTTTGAAAGCTTATTTATATATATAGACACG

CGTAAAAACTTCTTAACCAAGATTTTTTT-ACGTGCTTCC-TTCGCGTTTAATTTGGACC

ATCAAACCGTGCTCAACAGATAAAGAAAAGGGTGCTTTTGATTCAAGATATTGGCC-GAA

AAACACAAGATAGATCCTT-CGATAGATTAAGCCACGCATGAAACGCGAATCCAGAGTGA

TGAAGAAGTGCAGATAGATATTCGTTCACCATATAGAGGAGAATATATCATTCCTACAAA

CAAAATTGATGATGTTTCTAGAATAGAAACGTTAGAATCGAATATTCATCTGTTCCGGGT

GGGGAAGGGCTACTGAAAAGCTGAACTTTTTTAAGAGTTCGAACTTCGAAAGTCAATCAA

TGATTCTTAATTGAGAGGGAAAAGCTATAAAGGACGAGAAGGGAAGGAAATGTTTATAGG

GAATGAATTTCTAATGCATGATGGGAATGACAGATATGAGGAATATATAAAGGTGATTCT

TATATACATTGACATGGATCGGAATCTATTAACTTAAAGTTATTGGGGTCGTGAAATTTA

TTTAATTTTTCTAATTCGTGTCACATGTCACGACAATTAGTGGGG-AACATTTATGTATA

TAGAAATTTCAGAAATTTCTAGCAGCGTGATAAATATAAAGATTTTGGCACAATAAGTTT

TTGGATCATAATGAGTTT-CTATTTAATCAA-GGCA-TGTAA-TCTATTTTATTATTTAG

-TAGGG-AAACTGAAAACTTAGGGTTGCTATTTGTAGCTCCCACCCTTCTTAGTTCTTAC

TCTTTTCGATATTTTTTAAAAGTTTTA-TATAATACCT-AAATT-GCCCTCCTCCT----

----------------------------------------CACCCCATTGCTCTTCCGTT

GCTCCTCCATGTG-AGTTCTTGTCTTTTTTTTT-CTCTGGTAGCGTTTTGCTGCTCCTTT

TTTCACTCAAGTGTTGCCAATTAA-TTGACAAAAAATGGTTTCTGTTTCATATAGAAACT

ATGTTTTTGTTGTGTAGTCATACATTACGGAATCTAGTTT-CCATTAAATAAGTAAC--G

TGAAAAAAAA--TAAAAGGTGAAATATATATTGTTGGAAAAGAAGCTATGAGGTGCAAGA

ACCGATCACATGGAGAAGGCAATGAAAGACAAGGAGGAGCAATGGAAGA-----------

---------GAGAAAATGAGAAGATGGAAGGGATGTGAAAATGTTTGAAAAAAACGAGGT

GTTCAGTTTTAAAATACGAATTTAGTATTTTCTTTTTAAGAAAATTCTTTCG-AAAGTCG

TGTTTTAAAACATGACTTTTATT-ATTTGAAGTCG---TGTTCTAAAACATGACTTA--T

TCATATCCTT-AATATTTTT---------AAAATTTATCCATTTGTAATATTTTTTAAAA

ATTGACCCATATATGTAAAATACCCGTCAAGATCTCTTTATTATTTTGAAAGCGAAAGCA

TATCACTTCAAACACAATGGAATCGAGGCTATTGACTAAGTATAAATAGAGAAGACTTCA

TATCGGGGTTCATAATTCATAACAAAGCAAACGAGTATATAAGAAAGCATAAGCCAAATT

TTGAGTAAACTAGTGTGCACACTATCCCATGCCTAGTGGAAGTAGGGATCCTCTCGTTGT

TGGGGGAGTAATTGGGGATGTATTGGATCCTTTTGAATATTCTATTCCTATGAGGGTTAC

CTACAATAACAGAGATGTCAGCAATGGATGTGAATTCAAACCCTCACAAGTTGTCAACCA

ACCAAGGGTAAATATCGGTGGTGATGACC

>H03.03

TATTAGACTCCTAATTTAATATTCTTGTTTTATTATAATGCTGATAAGTCTTGTAAATAA

GGGTGAGAAGCACGAATAATTAGTTCATGAGATGTGTATAATTATTATCCTACACGACTT

ATCTTTGATATTTCACACAAGTCTTTCAATGTATAACAAAAACTTTTTAGATACATTTAG

ACTATAGAACTAACAAGTTATATTTTGAACC-AAAAAACAAAGAGAGAGAGAGAGGAACA

TAGAGAAAAGAAGATATGAGAGTTTTTTTTTTTTT------CTAAAACAGAAAGAAACTC

ATTATATAATAAACAAATTACTTTGAGACAAATTAACGTATGTAATAAAACAAAATCAAA

GTGGTAATTAAAATTATTTAATGGTAAACAGTCTAATAGTTAAAATAAAAATGGAAATCA

CATAAATTTTGTAATTGGCCCATTAAAAACAACACTAAGCTTTTAATTTGATTTTGAAAT

TCAAAATAATTTTATTAAATCACAAAGTAAAAGGTTTACAAAGCCGATCATGACAGTGCA

TGTGGGAGGCAAATCGGCATATTTGCACTACAAAAGG-ACCCATGAAGTCTCTGAACATG

CACGCAACACTTTAATCTCTTATTAGTTACTTTGAAAGCTTATTTATATATATAGACACG

CGTAAAAACTTCTTAACCAAGATTTTTTT-ACGTGCTTCC-TTCGCGTTTAATTTGGACC

ATCAAACCGTGCTCAACAGATAAAGAAAAGGGTGCTTTTGATTCAAGATATTGGCC-GAA

AAACACAAGATAGATCCTT-CGATAGATTAAGCCACGCATGAAACGCGAATCCAAAGTGA

TGAAGAAGTGCAGATAGATATTCGTTCACCATATAGAGGAGAATATATCATTCCTACAAA

CAAAATTGATGATGTTTCTAGAATAGAAACGTTAGAATCGAATATTCATCTGTTCCGGGT

GGGGAAGGGCTACTGAAAAGCTGAACTTTTTTAAGAGTTCGAACTTCGAAAGTCAATCAA

TGATTCTTAATTGAGAGGGAAAAGCTATAAAGGACGAGAAGGGAAGGAAATGTTTATAGG

GAATGAATTTCTAATGCATGATGGGAATGACAGATATGAGGAATATATAAAGGTGATTCT

TATATACATTGACATGGATCGGAATCTATTAACTTAAAGTTATTGGGGTCGTGAAATTTA

TTTAATTTTTCTAATTCGTGTCACATGTCACGACAATTAGTGGGG-AACATTTATGTATA

TAGAAATTTCAGAAATTTCTAGCAGCGTGATAAATATAAAGATTTTGGCACAATAAGTTT

TTGGATCATAATGAGTTT-CTATTTAATCAA-GGCA-TGTAA-TCTATTTTATTACTTAG

-TAGGG-AAACTGAAAACTTAGGGTTGCTATTTGTAGCTCCCACCCTTCTTAGTTCTTAC

TCTTTTCAATATTTTTTAAAAGTTTTA-TATAATACCT-AAATT-GCCCTCCTCCT----

----------------------------------------CACCCCATTGCTCTTCCGTT

GCTCCTCCATGTG-AGTTCTTGTCTTTTTTTTT-CTCTGGTAGCGTTTTGCTGCTCCTTT

TTTCACTCAAGTGTTGCCAATTAA-TTGACAAAAAATGGTTTCTGTTTCATATAGAAACT

ATGTTTTTGTTGTGTAGTCATACATTACGGAATCTAGTTT-CCATTAAATAAGTAAC--G

TGAAAAAAAA--TAAAAGGTGAAATATATATTGTTGGAAAAGAAGCTATGAGGTGCAAGA

ACCGATCACATGGAGAAGGCAATGAAAGACAAGGAGGAGCAATGGAAGA-----------

---------GAGAAAATGAGAAGATGGAAGGGATGTGAAAATGTTTGAAAAAAACGAGGT

GATCAGTTTTAAAATACGAATTTAGTATTTTCTTTTTAAGAAAATTCTTTCG-AAAGTCG

TGTTTTAAAACATGACTTTTATT-ATTTGAAGTCG---TGTTCTAAAACATGACTTA--T

TCATATCCTT-AATATTTTT---------AAAATTTGTCCATTTGTAATATTTTTTAAAA

ATTGACCCATATATGTAAAATACCCGTCAAGATCCCTTTATTATTTTGAAAGCGAAAGCA

TATCACTTCAAACACAATGGAATCGAGGCTATTGACTAAGTATAAATAGAGAAGACTTCA

TATCGGGGTTCATAATTCATAACAAAGCAAGCGAGTATATAAGAAAGCATAAGCCAAATT

TTGAGTAAACTAGTGTGCACACTATCCCATGCCTAGTGGAAGTAGGGATCCTCTCGTTGT

TGGGGGAGTAATTGGGGATGTATTGGATCCTTTTGAATATTCTATTCCTATGAGGGCTAC

CTACAATAACAGAGATGTCAGCAATGGATGTGAATTCAAACCCTCACAAGTTGTCAACCA

ACCAAGGGTAAATATCGGTGGTGATGACC

>H03.06c

TATTAGACTCCTAATTTAATATTCTTGTTTTATTATAATGCTGATAAGTCTTGTAAATAA

GGGTGAGAAGCACGAATAATTAGTTCATGAGATGTGTATAATTATTATCCTACACGACTT

ATCTTTGATATTTCACACAAGTCTTTCAATGTATAACAAAAACTTTTTAGATACATTTAG

ACTATAGAACTAACAAGTTATATTTTGAACC-AAAAAACAAAGAGAGAGAGAGAGGAACA

TAGAGAAAAGAAGATATGAGAGTTTTTTTTTTTTT------CTAAAACAGAAAGAAACTC

ATTATATAATAAACAAATTACTTTGAGACAAATTAACGTATGTAATAAAACAAAATCAAA

GTGGTAATTAAAATTATTTAATGGTAAACAGTCTAATAGTTAAAATAAAAATGGAAATCA

CATAAATTTTGTAATTGGCCCATTAAAAACAACACTAAGCTTTTAATTTGATTTTGAAAT

TCAAAATAATTTTATTAAATCACAAAGTAAAAGGTTTACAAAGCCGATCATGACAGTGCA

TGTGGGAGGCAAATCGGCATATTTGCACTACAAAAGG-ACCCATGAAGTCTCTGAACATG

CACGCAACACTTTAATCTCTTATTAGTTACTTTGAAAGCTTATTTATATATATAGACACG

CGTAAAAACTTCTTAACCAAGATTTTTTT-ACGTGCTTCC-TTCGCGTTTAATTTGGACC

ATCAAACCGTGCTCAACAGATAAAGAAAGGGGTGCTTTTGATTCAAGATATTGGCC-GAA

AAACACAAGATAGATCCTT-CGATAGATTAAGCCACGCATGAAACGCGAATCCAAAGTGA

TGAAGAAGTGCAGATAGATATTCGTTCACCATATAGAGGAGAATATATCATTCCTACAAA

CAAAATTGATGATGTTTCTAGAATAGAAACGTTAGAATCGAATATTCATCTGTTCCGGGT

GGGGAAGGGCTACTGAAAAGCTGAACTTTTTTAAGAGTTCGAACTTCGAAAGTCAATCAA

TGATTCTTAATTGAGAGGGAAAAGCTATAAAGGACGAGAAGGGAAGGAAATGTTTATAGG

GAATGAATTTCTAATGCATGATGGGAATGACAGATATGAGGAATATATAAAGGTGATTCT

TATATACATTGACATGGATCGGAATCTATTAACTTAAAGTTATTGGGGTCGTGAAATTTA

TTTAATTTTTCTAATTCGTGTCACATGTCACGACAATTAGTGGGG-AACATTTATGTATA

TAGAAATTTCAGAAATTTCTAGCAGCGTGATAAATATAAAGATTTTGGCACAATAAGTTT

TTGGATCATAATGAGTTT-CTATTTAATCAAAGGCA-TGTAA-TCTATTTTATTATTTAG

-TAGGG-AAACTGAAAACTTAGGGTTGCTATTTGTAGCTCCCACCCTTCTTAGTTCTTAC

TCTTTTCAATATTTTTTAAAAGTTTTA-TATAATACCT-AAATT-GCCCTCCTCCT----

----------------------------------------CACCCCATTGCTCTTCCGTT

GCTCCTCCATGTG-AGTTCTTGTCTTTTTTTTT-CTCTGGTAGCGTTTTGCTGCTCCTTT

TTTCACTCAAGTGTTGCCAATTAA-TTGACAAAAAATGGTTTCTGTTTCATATAGAAACT

ATGTTTTTGTTGTGTAGTCATACATTACGGAATCTAGTTT-CCATTAAATAAGTAAC--G

TGAAAAAAAA--TAAAAGGTGAAATATATATTGTTGGAAAAGAAGCTATGAGGTGCAAGA

ACCGATCACATGGAGAAGGCAATGAAAGACAAGGAGGAGCAATGGAAGA-----------

---------GAGAAAATGAGAAGATGGAAGGGATGTGAAAATGTTTGAAAAAAACGAGGA

GATCAGTTTTAAAATACGAATTTAGTATTTTCTTTTTAAGAAAATTCTTTCG-AAAGTCG

TGTTTTAAAACATGACTTTTATT-ATTTGAAGTCG---TGTTCTAAAACATGACTTA--T

TCATATCCTT-AATATTTTT---------AAAATTTATCCATTTGTAATATTTTTTAAAA

ATTGACCCATATATGTAAAATACCCGTCAAGATCTCTTTATTATTTTGAAAGCGAAAGCA

TATCACTTCAAACACAATGGAATCGAGGCTATTGACTAAGTATAAATAGAGAAGACTTCA

TATCGGGGTTCATAATTCATAACAAAGCAAACGAGTATATAAGAAAGCATAAGCCAAATT

TTGAGTAAACTAGTGTGCACACTATCCCATGCCTAGTGGAAGTAGGGATCCTCTCGTTGT

TGGGGGAGTAATTGGGGATGTATTGGATCCTTTTGAATATTCTATTCCTATGAGGGTTAC

CTACAATAACAGAGATGTCAGCAATGGATGTGAATTCAAACCCTCACAAGTTGTCAACCA

ACCAAGGGTAAATATCGGTGGTGATGACC

>CS35.42

TATTAGACTCCTAATTTAATATTCTTGTTTTATTATAATGCTGATAAGTCTTGTAAATAA

GGGTGAGAAGCACGAATAATTAGTTCATGAGATGTGTATAATTATTATCCTACACGACTT

ATCTTTGATATTTCACACAAGTCTTTCAATGTATAACAAAAACTTTTTAGATACATTTAG

ACTATAGAACTAACAAGTTATATTTTGAACC-AAAAAACAAAGAGAGAGAGAGAGGAACA

TAGAGAAAAGAAGATATGAGAGTTTTTTTTTTTTTTTTT--CTAAAACAGAAAGAAACTC

ATTATATAATAAACAAATTACTTTGAGACAAATTAACGTATGTAATAAAACAAAATCAAA

GTGGTAATTAAAATTATTTAATGGTAAACAGTCTAATAGTTAAAATAAAAATGGAAATCA

CATAAATTTTGTAATTGGCCCATTAAAAACAACACTAAGCTTTTAATTTGATTTTGAAAT

TCAAAATAATTTTATTAAATCACAAAGTAAAAGGTTTACAAAGCCGATCATGACAGTGCA

TGTGGGAGGCAAATCGGCATATTTGCACTACAAAAGG-ACCCATGAAGTCTCTGAACATG

CACGCAACACTTTAATCTCTTATTAGTTACTTTGAAAGCTTATTTATATATATAGACACG

CGTAAAAACTTCTTAACCAAGATTTTTTT-ACGTGCTTCC-TTCGCGTTTAATTTGGACC

ATCAAACCGTGCTCAACAGATAAAGAAAAGGGTGCTTTTGATTCAAGATATTGGCC-GAA

AAACACAAGATAGATCCTT-CGATAGATTAAGCCACGCATGAAACGCGAATCCAAAGTGA

TGAAGAAGTGCAGATAGATATTCGTTCACCATATAGAGGAGAATATATCATTCCTACAAA

CAAAATTGATGATGTTTCTAGAATAGAAACGTTAGAATCGAATATTCATCTGTTCCGGGT

GGGGAAGGGCTACTGAAAAGCTGAACTTTTTTAAGAGTTCGAACTTCGAAAGTCAATCAA

TGATTCTTAATTGAGAGGGGAAAGCTATAAAGGACGAGAAGGGAAGGAAATGTTTATAGG

GAATGAATTTCTAATGCATGATGGGAATGACAGATATGAGGAATATATAAAGGTGATTCT

TATATACATTGACATGGATCGGAATCTATTAACTTAAAGTTATTGGGGTCGTGAAATTTA

TTTAATTTTTCTAATTCGTGTCACATGTCACGACAATTAGTGGGG-AACATTTATGTATA

TAGAAATTTCAGAAATTTCTAGCAGCGTGATAAATATAAAGATTTTGGCACAATAAGTTT

TTGGATCATAATGAGTTT-CTATTTAATCAA-GGCA-TGTAA-TCTATTTTATTATTTAG

-TAGGG-AAACTGAAAACTTAGGGTTGCTATTTGTAGCTCCCACCCTTCTTAGTTCTTAC

TCTTTTCAATATTTTTTAAAAGTTTTA-TATAATACCT-AAATT-GCCCTCCTCCT----

----------------------------------------CACCCCATTGCTCTTCCGTT

GCTCCTCCATGTG-AGTTCTTGTCTTTTTTTT--CTCTGGCAGCGTTTTGCTGCTCCTTT

TTTCACTCAAGTGTTGCCAATTAA-TTGACAAAAAATGGTTTCTGTTTCATATAGAAACT

ATGTTTTTGTTGTGTAGTCATACATTACGGAATCTAGTTT-CCATTAAATAAGTAAC--G

TGAAAAAAAA--TAAAAGGTGAAATATATATTGTTGGAAAAGAAGCTATGAGGTGCAAGA

ACCGATCACATGGAGAAGGCAATGAAAGACAAGGAGGAGCAATGGAAGA-----------

---------GAGAAAATGAGAAGATGGAAGGGATGTGAAAATGTTTGAAAAAAACGAGGT

GATCAGTTTTAAAATACGAATTTAGTATTTTCTTTTTAAGAAAATTCTTTCG-AAAGTCG

TGTTTTAGAACATGACTTTTATT-ATTTGAAGTCG---TGTTCTAAAACATGACTTA--T

TCATATCCTT-AATATTTTT---------AAAATTTATCCATTTGTAATATTTTTTAAAA

ATTGACCCATATATGTAAAATACCCGTCAAGATCTCTTTATTATTTTGAAAGCGAAAGCA

TATCACTTCAAACACAATGGAATCGAGGCTATTGACTAAGTATAAATAGAGAAGACTTCA

TATCGGGGTTCATAATTCATAACAAAGCAAACGAGTATATAAGAAAGCATAAGCCAAATT

TTGAGTAAACTAGTGTGCACACTATCCCATGCCTAGTGGAAGTAGGGATCCTCTCGTTGT

TGGGGGAGTAATTGGGGATGTATTAGATCCTTTTGAATATTCTATTCCTATGAGGGTTAC

CTACAATAACAGAGATGTCAGCAATGGATGTGAATTCAAACCCTCACAAGTTGTCAACCA

ACCAAGGGTAAATATCGGTGGTGATGACC

>CS09.10

TATTAGACTCCTAATTTAATATTCTTGTTTTATTATAATGCTGATAAGTCTTGTAAATAA

GGGTGAGAAACACGAATAATTAGTTCATGAGATGTGTATAATTATTATCCTACACGACTT

ATCTTTGATATTTCACACAAGTCTTTCAATGTATAACAAAAACTTTTTAGATACATTTAG

ACTATAGAACTAACAAGTTATATTTTGAACC-AAAAAACAAAGAGAGAGAGAGAGGAACA

TAGAGAAAAGAAGATATGAGAGTTTTTTTTTTTTTTTTT--CTAAAACAGAAAGAAACTC

GTTATATAATAAACAAATTACTTTGAGACAAATTAACGTATGTAATAAAACAAAATCAAA

GTGGTAATTAAAATTATTTAATGGTAAACAGTCTAATAGTTAAAATAAAAATGGAAATCA

CATAAATTTTGTAATTGGCCCATTAAAAACAACACTAAGCTTTTAATTTGATTTTGAAAT

TCAAAATAATTTTATTAAATCACAAAGTAAAAGGTTTACAAAGCCGATCATGGCAGTGCA

TGTGGGAGGCAAATCGGCATATTTGCACTACAAAAGG-ACCCATGAAGTCTCTGAACATG

CACGCAACACTTTAATCTCTTATTAGTTACTTTGAAAGCTTATTTATATGTATAGACACG

CGTAAAAACTTCTTAACCAAGATTTTTTT-ACGTGCTTCC-TTCGCGTTTAATTTGGACC

ATCAAACCGTGCTCAACAGATAAAGAAAAGGGTGCTTTTGATTCAAGATATTGGCC-GAA

AAACACAAGATAGATCCTT-CGATAGATTAAGCCACGCATGAAACGCGAATCCAAAGTGA

TGAAGAAGTGCAGATAGATATTCGTTCACCATATAGAGGAGAATATATCATTCCTACAAA

CAAAATTGATGATGTTTCTAGAATAGAAACGTTAGAATCGAATATTCATCTGTTCCGGGT

GGGGAAGGGCTACTGAAAAGCTGAACTTTTTTAAGAGTTCGAACTTCGAAAGTCAATCAA

TGATTCTTAATTGAGAGGGAAAGGCTATAAAGGACGAGAAGGGAAGGAAATGTTTATAGG

GAATGAATTTCTAATGCATGATGGGAATGACAGATATGAGGAATATATAAAGGTGATTCT

TATATACATTGACATGGATCGGAATCTATTAACTTAAAGTTATTGGGGTCGTGAAATTTA

TTTAATTTTTCTAATTCGTGTCACATGTCACGACAATTAGTGGGG-AACATTTATGTATA

TAGAAATTTCAGAAATTTCTAGCAGCGTGATAAATATAAAGATTTTGGCACAATAAGTTT

TTGGATCATAATGAGTTT-CTATTTAATCAA-GGCA-TGTAA-TCTATTTTATTATTTAG

-TAGGG-AAACTGAAAACTTAGGGTTGCTATTTGTAGCTCCCACCCTTCTTAGTTCTTAC

TCTTTTCAATATTTTTTAAAAGTTTTA-TATAATACCT-AAATT-GCCCTCCTCCT----

----------------------------------------CACCCCATTGCTCTTCCGTT

GCTCCTCCATGTG-AGTTCTTGTCTTTTTTTTT-CTCTGGTAGCGCTTTGCTGCTCCTTT

TTTCACTCAAGTGTTGCCAATTAA-TTGACAAAAAATGGTTTCTGTTTCATATAGAAACT

ATGTTTTTGTTGTGTAGTCATACATTACGGAATCTAGTTT-CCATTAAATAAGTAAC--G

TGAAAAAAAA--TAAAAGGTGAAATATATATTGTTGGAAAAGAAGCTATGAGGTGCAAGA

ACCGATCACATGGAGAAGGCAATGAAAGACAAGGAGGAGCAATGGAAGA-----------

---------GAGAAAATGAGAAGATGGAAGGGATGTGAAAATGTTTGAAAAAAACGAGGT

GATCAGTTTTAAAATACGAATTTAGTATTTTCTTTTTAAGAAAATTCTTTCG-AAAGTCG

TGTTTTAAAACATGACTCTTATT-ATTTGAAGTCG---TGTTCTAAAACATGACTTA--T

TCATATCCTT-AATATTTTT---------AAAATTTATCCATTTGTAATATTTTTTAAAA

ATTGACCCATATATGTAAAATACCCGTCAAGATCTCTTTATTATTTTGAAAGCGAAAGCA

TATCACTTCAAACACAATGGAATCGAGGCTATTGACTAAGTATAAATAGAGAAGACTTCA

TATCGGGGTTCATAATTCATAACAAAGCAAACGAGTATATAAGAAAGCATAAGCCAAATT

TTGAGTAAACTAGTGTGCACACTATCCCATGCCTAGTGGAAGTAGGGATCCTCTCGTTGT

TGGGGGAGTAATTGGGGATGTATTGGATCCTTTTGAATATTCTATTCCTATGAGGGTTAC

CTACAATAACAGAGATGTCAGCAATGGATGTGAATTCAAACCCTCACAAGTTGTCAACCA

ACCAAGGGTAAATATCGGTGGTGATGACC

>CS15.18

TATTAGACTCCTAATTTAATATTCTTGTTTTATTATAATGCTGATAAGTCTTGTAAATAA

GGGTGAGAAGCACGAATAATTAGTTCATGAGATGTGTATAATTATTATCCTACACGACTT

ATCTTTGATATTTCACACAAGTCTTTCAATGTATAACAAAAACTTTTTAGATACATTTAG

ACTATAGAACTAACAAGTTATATTTTGAACCCAAAAAACAAAGAGAGAGAGAGAGGAACA

TAGAGAAAAGAAGATATGAGAGTTTTTTTTTTTTTTTTT--CTAAAACAGAAAGAAACTC

ATTATATAATAAACAAATTACTTTGAGACAAATTAACGTATGTAATAAAACAAAATCAAA

GTGGTAATTAAAATTATTTAATGGTAAACAGTCTAATAGTTAAAATAAAAATGGAAATCA

CATAAATTTTGTAATTGGCCCATTAAAAACAACACTAAGCTTTTAATTTGATTTTGAAAT

TCAAAATAATTTTATTAAATCACAAAGTAAAAGGTTTACAAAGCCGATCATGACAGTGCA

TGTGGGAGGCAAATCGGCATATTTGCACTACAAAAGG-ACCCATGAAGTCTCTGAACATG

CACGCAACACTTTAATCTCTTATTAGTTACTTTGAAAGCTTATTTATATATATAGACACG

CGTAAAAACTTCTTAACCAAGATTTTTTT-ACGTGCTTCC-TTCGCGTTTAATTTGGACC

ATCAAACCGTGCTCAACAGATAAAGAAAAGGGTGCTTTTGATTCAAGATATTGGCC-GAA

AAACACAAGATAGATCCTT-CGATAGATTAAGCCACGCATGAAACGCGAATCCAAAGTGA

TGAAGAAGTGCAGATAGATATTCGTTCACCATATAGAGGAGAATATATCATTCCTACAAA

CAAAATTGATGATGTTTCTAGAATAGAAACGTTAGAATCGAATATTCATCTGTTCCGGGT

GGGGAAGGGCTACTGAAAAGCTGAACTTTTT-AAGAGTTCGAACTTCGAAAGTCAATCAA

TGATTCTTAATTGAGAGGGAAAAGCTATAAAGGACGAGAAGGGAAGGAAATGTTTATAGG

GAATGAATTTCTAATGCATGATGGGAATGACAGATATGAGGAATATATAAAGGTGATTCT

TATATACATTGACATGGATCGGAATCTATTAACTTAAAGTTATTGGGGTCGTGAAATTTA

TTTAATTTTTCTAATTCGTGTCACATGTCACGACAATTAGTGGGG-AACATTTATGTATA

TAGAAATTTCAGAAATTTCTAGCAGCGTGATAAATATAAAGATTTTGGCACAATAAGTTT

TTGGATCATAATGAGTTT-CTATTTAATCAA-GGCA-TGTAA-TCTATTTTATTATTTAG

-TAGGG-AAACTGAAAACTTAGGGTTGCTATTTGTAGCTCCCACCCTTCTTAGTTCTTAC

TCTTTTCAATATTTTTTAAAAGTTTTA-TATAATACCT-AAATT-GCCCTCCTCCT----

----------------------------------------CACCCCATTGCTCTTCCGTT

GCTCCTCCATGTG-AGTTCTTGTCTTTTTTTT--CTCTGGTAGCGTTTTGCTGCTCCTTT

TTTCACTCAAGCGTTGCCGATTAA-TTGACAAAAAATGGTTTCTGTTTCATATAGAAACT

ATGTTTTTGTTGTGTAGTCATACATTACGGAATCTAGTTT-CCATTAAATAAGTAAC--G

TGAAAAAAAA--TAAAAGGTGAAATATATATTGTAGGAAAAGAAGCTATGAGGTGCAAGA

ACCGATCACATGGAGAAGGCAATGAAAGACAAGGAGGAGCAATGGAAGA-----------

---------GAGAAAATGAGAAGATGGAAGGGATGTGAAAACGTTTGAAAAAAACGAGGT

GATCAGTTTTAAAATACGAATTTAGTATTTTCTTTTTAAGAAAATTCTTTCG-AAAGTCG

TGTTTTAAAACATGACTTTTATT-ATTTGAAGTCG---TGTTCTAAAACATGACTTA--T

TCATATCCTT-AATATTTTT---------AAAATTTATCCATTTGTAATATTTTTTAAAA

ATTGACCCATATATGTAGAATACCCGTCAAGATCTCTTTATTATTTTGAAAGCGAAAGCA

TATCACTTCAAACACAATGGAATCGAGGCTATTGACTAAGTATAAATAGAGAAGACTTCA

TATCGGGGTTCATAATTCATAACAAAGCAAACGAGTATATAAGAAAGCATAAGCCAAATT

TTGAGTAAACTAGTGTGCACACTATCCCATGCCTAGTGGAAGTAGGGATCCTCTCGTTGT

TGGGGGAGTAATTGGGGATGTATTGGATCCTTTTGAATATTCTATTCCTATGAGGGTTAC

CTACAATAACAGAGATGTCAGCAATGGATGTGAATTCAAACCCTCACAAGTTGTCAACCA

ACCAAGGGTAAATATCGGTGGTGATGACC

>H13.E5

TATTAGACTCCTAATTTAATATTCTTGTTTTATTATAATGCTGATAAGTCTTGTAAATAA

GGGTGAGAAGCACGAATAATTAGTTCATGAGATGTGTATAATTATTATCCTACACGACTT

ATCTTTGATATTTCACACAAGTCTTTCAATGTATAACAAAAACTTTTTAGATACATTTAG

ACTATAGAACTAACAAGTTATATTTTGAACC-AAAAAACAAAGAGAGAGAGAGAGGAACA

TAGAGAAAAGAAGATATGAGAGTTTTTTTTTTTTTTT----CTAAAACAGAAAGAAACTC

ATTATATAATAAACAAATTACTTTGAGACAAATTAACGTATGTAATAAAACAAAATCAAA

GTGGTAATTAAAATTATTTAATGGTAAACAGTCTAATAGTTAAAATAAAAATGGAAATCA

CATAAATTTTGTAATTGGCCCATTAAAAACAACACTAAGCTTTTAATTTGATTTTGAAAT

TCAAAATAATTTTATTAAATCACAAAGTAAAAGGTTTACAAAGCCGATCATGACAGTGCA

TGTGGGAGGCAAATCGGCATATATGCACTACAAAAGG-ACCCATGAAGTCTCTGAACATG

CACGCAACACTTTAATCTCTTATTAGTTACTTTGAAAGCTTATTTATATATATAGACACG

CGTAAAAACTTCTTAACCAAGATTTTTTT-ACGTGCTTCC-TTCGCGTTTAATTTGGACC

ATCAAACCGTGCTCAACAGATAAAGAAAAGGGTGCTTTTGATTCAAGATATTGGCCCGAA

AAACACAAGATAGATCCTT-CGATAGATTAAGCCACGCATGAAACGCGAATCCAAAGTGA

TGAAGAAGTGCAGATAGATATTCGTTCACCATATAGAGGAGAATATATCATTCCTACAAA

CAAAATTGATGATGTTTCTAGAATAGAAACGTTAGAATCGAATATTCATCTGTTCCGGGT

GGGGAAGGGCTACTGAAAAGCTGAACTTTTTCAAGAGTTCGAACTTCGAAAGTCAATCAA

TGATTCTTAATTGAGAGGGAAAAGCTATAAAGGACGAGAAGGGAAGGAAATGTTTATAGG

GAATGAATTTCTAATGCATGATGGGAATGACAGATATGAGGAATATATAAAGGTGATTCT

TATATACATTGACATGGATCGGAATCTATTAACTTAAAGTTATTGGGGTCGTGAAATTTA

TTTAATTTTTCTAATTCGTGTCACATGTCACGACAATTAGTGGGG-AACATTTATGTATA

TAGAAATTTCAGAAATTTCTAGCAGCGTGATAAATATAAAGATTTTGGCACAATAAGTTT

TTGGATCATAATGAGTTT-CTATTTAATCAA-GGCA-TGTAA-TCTATTTTATTATTTAG

-TAGGG-AAACTGAAAACTTAGGGTTGCTATTTGTAGCTCCCACCCTTCTTAGTTCTTAC

TCTTTTCAATATTTTTTAAAAGTTTTA-TATAATACCT-AAATT-GCCCTCCTCCT----

----------------------------------------CACCCCATTGCTCTTCCGTT

GCTCCTCCATGTG-AGTTCTTGTCTTTTTTTTT-CTCTGGTAGCGTTTTGCTGCTCCTTT

TTTCACTCAAGTGTTGCCAATTAA-TTGACAAAAAATGGTTTCTGTTTCATATAGAAACT

ATGTTTTTGTTGTGTAGTCATACATTACGGAATCTAGTTT-CCATTAAATAAGTAAC--G

TGAAAAAAAA--TAAAAGGTGAAATATATATTGTTGGAAAGGAAGCTATGAGGTGCAAGA

ACCGATCACATGGAGAAGGCAATGAAAGACAAGGAGGAGCAATGGAAGA-----------

---------GAGAAAATGAGAAGATGGAAGGGATGTGAAAATGTTTGAAAAAAACGAGGT

GATCAGTTTTAAAATACGAATTTAGTATTTTCTTTTTAAGAAAATTCTTTCG-AAAGTCG

TGTTTTAAAACATGACTTTTATT-ATTTGAAGTCG---TGTTCTAAAACATGACTTA--T

TCATATCCTT-AATATTTTT---------AAAATTTATCCATTTGTAATATTTTTTAAAA

ATTGACCCATATATGTAAAATACCCGTCAAGATCTCTTTATTATTTTGAAAGCGAAAGCA

TATCACTTCAAACACAATGGAATCGAGGCTATTGACTAAGTATAAATAGAGAAGACTTCA

TATCGGGGTTCATAATTCATAACAAAGCAAACGAGTATATAAGAAAGCATAAGCCAAATT

TTGAGTAAACTAGTGTGCACACTATCCCATGCCTAGTGGAAGTAGGGATCCTCTCGTTGT

TGGGGGAGTAATTGGGGATGTATTGGATCCTTTTGAATATTCTATTCCTATGAGGGTTAC

CTACAATAACAGAGATGTCAGCAATGGATGTGAATTCAAACCCTCACAAGTTGTCAACCA

ACCAAGGGTAAATATCGGTGGTGATGACC

>CS09.16

TATTAGACTCCTAATTTAATATTCTTGTTTTATTATAATGCTGATAAGTCTTGTAAATAA

GGGTGAGAAGCACGAATAATTAGTTCATGAGATGTGTATAATTATTATCCTACACGACTT

ATCTTTGATATTTCACACAAGTCTTTCAATGTATAACAAAAACTTTTTAGATACATTTAG

ACTATAGAACTAACAAGTTATATTTTGAACC-AAAAAACAAAGAGAGAGAGAGAGGAACA

TAGAGAAAAGAAGATATGAGAGTTTTTTTTTTTTTTTTT--CTAAAACAGAAAGAAACTC

ATTATATAATAAACAAATTACTTTGAGACAAATTAACGTATGTAATAAAACAAAATCAAA

GTGGTAATTAAAATTATTTAATGGTAAACAGTCTAATAGTTAAAATAAAAATGGAAATCA

CATAAATTTTGTAATTGGCCCATTAAAAACAACACTAAGCTTTTAATTTGATTTTGAAAT

TCAAAATAATTTTATTAAATCACAAAGTAAAAGGTTTACAAAGCCGATCATGACAGTGCA

TGTGGGAGGCAAATCGGCATATTTGCACTACAAAAGG-ACCCATGAAGTCTCTGAACATG

CACGCAGCACTTTAATCTCTTATTAGTTACTTTGAAAGCTTATTTATATATATAGACACG

CGTAAAAACTTCTTAACCAGGATTTTTTT-ACGTGCTTCC-TTCGCGTTTAATTTGGGCC

ATCAAACCGTGCTCAACAGATAAAGAAAAGGGTGCTTTTGATTCAAGATATTGGCC-GAA

AAACACAAGATAGATCCTT-CGATAGATTAAGCCGCGCATGAAACGCGAATCCAAAGTGA

TGAAGAAGTGCAGATAGATATTCGTTCACCATATAGAGGAGAATATATCATTCCTACAAA

CAAAATTGATGATGTTTCTAGAATAGAAACGTTAGAATCGAATATTCATCTGTTCCGGGT

GGGGAAGGGCTACTGAAAAGCTGAACTTTTTTAAGAGTTCGAACTTCGAAAGTCAATCAA

TGATTCTTAATTGAGAGGGAAAAGCCATAAAGGACGAGAAGGGAAGGAAATGTTTATAGG

GAATGAATTTCTAATGCATGATGGGAATGACAGATATGAGGAATATATAAAGGTGATTCT

TATATACATTGACATGGATCGGAATCTATTAACTTAAAGTTATTGGGGTCGTGAAATTTA

TTTAATTTTTCTAATTCGTGTCACATGTCACGACAATTAGTGGGG-AACATTTATGTATA

TAGAAATTTCAGAAATTTCTAGCAGCGTGATAAATATAAAGATTTTGGCACAATAAGTTT

TTGGATCATAATGAGTTT-CTATTTAATCAA-GGCA-TGTAA-TCTATTTTATTATTTAG

-TAGGG-AAACTGAAAACTTATGGTTGCTATTTGTAGCTCCCACCCTTCTTAGTTCTTAC

TCTTTTCAATATTTTTTAAAAGTTTTA-TATAATACCT-AAATT-GCCCTCCTCCT----

----------------------------------------CACCCCATTGCTCTTCCGTT

GCTCCTCCATGTG-AGTTCTTGTCTTTTTTTT--CTCCGGTAGCGTTTTGCTGCTCCTTT

TTTCACTCAAGTGTTGCCAATTAA-TTGACAAAAAATGGTTTCTGTTTCATATAGAAACT

ATGTTTTTGTTGTGTAGTCATACATTACGGAATCTAGTTT-CCATTAAATAAGTAAC--G

TGAAAAAAAA--TAAAAGGTGAAATATATATTGTTGGAAAAGAAGCTATGAGGTGCAAGA

ACCGATCACATGGAGAAGGCAATGAAAGACAAGGAGGAGCAATGGAAGA-----------

---------GAGAAAATGAGAAGATGGAAGGGATGTGAAAATGTTTGAAAAAAACGAGGT

GACCAGTTTTAAAATACGAATTTAGTATTTTCTTTTTAAGAAAATTCTTTCG-AAAGTCG

TGTTTTAAAACATGACTTTTATT-ATTTGAAGTCG---TGTTCTAAAACATGACTTA--T

TCATATCCTT-AATATTTTT---------AAAATTTATCCATTTGTAATATTTTTTAAAA

ATTGACCCATATATGTAAAATACCCGTCAAGATCTCTTTATTATTTTGAAAGCGAAAGCA

TATCACTTCAAACACAATGGAATCGAGGCTATTGACTAAGTATAAATAGAGAAGACTTCA

TATCGGGGTTCATAATTCATAACAAAGCAAACGAGTATATAAGAAAGCATAAGCCAAATT

TTGAGTAAACTAGTGTGCACACTATCCCATGCCTAGTGGAAGTAGGGATCCTCTCGTTGT

TGGGGGAGTAATTGGGGATGTATTGGATCCTTTTGAATATTCTATTCCTATGAGGGTTAC

CTACAATAACAGAGATGTCAGCAATGGATGTGAATTCAAACCCTCACAAGTTGTCAACCA

ACCAAGGGTAAATATCGGTGGTGATGACC

>CS04.23

TATTAGACTCCTAATTTAATATTCTTGTTTTATTATAATGCTGATAAGTCTTGTAAATAA

GGGTGAGAAGCACGAATAATTAGTTCATGAGATGTGTATAATTATTATCCTACACGACTT

ATCTTTGATATTTCACACAAGTCTTTCAATGTATAACAAAAACTTTTTAGATACATTTAG

ACTATAGAACTAACAAGTTATATTTTGAACC-AAAAAACAAAGAGAGAGAGAGAGGAACA

TAGAGAAAAGAAGATATGAGAGTTTTTTTTTTTTTTTTT--CTAAAACAGAAAGAAACTC

ATTATATAATAAACAAATTACTTTGAGACAAATTAACGTATGTAATAAAACAAAATCAAA

GTGGTAATTAAAATTATTTAATGGTAAACAGTCTAATAGTTAAAATAAAAATGGAAATCA

CATAAATTTTGTAATTGGCCCATTAAAAACAACACTAAGCTTTTAATTTGATTTTGAAAT

TCAAAATAATTTTATTAAATCACAAAGTAAAAGGTTTACAAAGCCGATCATGACAGTGCA

TGTGGGAGGCAAATCGGCATATTTGCACTACAAAAGG-ACCCATGAAGTCTCTGAACATG

CACGCAACACTTTAATCTCTTATTAGTTACTTTGAAAGCTTATTTATATATATAGACACG

CGTAAAAACTTCTTAACCAAGATTTTTTT-ACGTGCTTCC-TTCGCGTTTAATTTGGACC

ATCAAACCGTGCTCAACAGATAAAGGAAAGG-TGCTTTTGATTCAAGATATTGGCC-GAA

AAACACAAGATAGATCCTT-CGATAGATTAAGCCACGCATGAAACGCGAATCCAAAGTGA

TGAAGAAGTGCAGATAGATATTCGTTCACCATATAGAGGAGAATATATCATTCCTACAAA

CAAAATTGATGATGTTTCTAGAATAGAAACGTTAGAATCGAATATTCATCTGTTCCGGGT

GGGGAAGGGCTACTGAAAAGCTGAACTTTTTTAAGAGTTCGAACTTCGAAAGTCAATCAA

TGATTCTTAATTGAGAGGGAAAAGCTATAAAGGACGAGAAGGGAAGGAAATGTTTATAGG

GAATGAATTTCTAATGCATGATGGGAATGACAGATATGAGGAATATATAAAGGTGATTCT

TATATACATTGACATGGATCGGAATCTATTAACTTAAAGTTATTGGGGTCGTGAAATTTA

TTTAATTTTTCTAATTCGTGTCACAAGTCACGACAATTAGTGGGG-AACATTTATGTATA

TAGAAATTTCAGAAATTTCTAGCAGTGTGATAAATATAAAGATTTTGGCACAATAAGTTT

TTGGATCATAATGAGTTT-CTATTTAATCAA-GGCA-TGTAA-TCTATTTTATTATTTAG

-TAGGG-AAACTGAAAACTTAGGGTTGCTATTTGTAGCTCCCACCCTTCTTAGTTCTTAC

TCTTTTCAATATTTTTTAAAAGTTTTA-TATAATACCT-AAATT-GCCCTCCTCCT----

----------------------------------------CACCCCATTGCTCTTCCGTT

GCTCCTCCATGTG-AGTTCTTGTCTTTTTTTT--CTCTGGTAGCGTTTTGCTGCTCCTTT

TTTCACTCAAGTGTTGCCAATTAA-TTGACAAAAAATGGTTTCTGTTTCATATAGAAACT

ATGTTTTTGTTGTGTAGTCATACATTACGGAATCTAGTTT-CCATTAAATAAGTAAC--G

TGAAAAAAAA--TAAAAGGTGAAATATATATTGTTGGAAAAGAAGCTATGAGGTGCAAGA

ACCGATCACATGGAGAAGGCAATGAAAGACAAGGAGGAGCAATGGAAGA-----------

---------GAGAAGATGAGAAGATGGAAGGGATGTGAAAATGTTTGAAAAAAACGAGGT

GATCAGTTTTAAAATACGAATTTAGTATTTTCTTTTTAAGAAAATTCTTTCG-AAAGTCG

TGTTTTAAAACATGACTTTTATT-ATTTGAAGTCG---TGTTCTAAAACATGACTTA--T

TCATATCCTT-AATATTTTT---------AAAATTTATCCATTTGTAATATTTTTTAAAA

ATTGACCCATATATGTAAAATACCCGTCAAGATCTCTTTATTATTTTGAAAGCGAAAGCA

TATCACTTCAAACACAACGGAATCGAGGCTATTGACTAAGTATAAATAGAGAAGACTTCA

TATCGGGGTTCATAATTCATAACAAAGCAAACGAGTATATAAGAAAGCATAAGCCAAATT

TTGAGTAAACTAGTGTGCACACTATCCCATGCCTAGTGGAAGTAGGGATCCTCTCGTTGT

TGGGGGAGTAATTGGGGATGTATTGGATCCTTTTGAATATTCTATTCCTATGAGGGTTAC

CTACAATAACAGAGATGTCAGCAATGGATGTGAATTCAAACCCTCACAAGTTGTCAACCA

ACCAAGGGTAAATATCGGTGGTGATGACC

>CS30.16

TATTAGACTCCTAATTTAATATTCTTGTTTTATTATAATGCTGATAAGTCTTGTAAATAA

GGGTGAGAAGCACGAATAATTAGTTCATGAGATGTGTATAATTATTATCCTACACGACTT

ATCTTTGATATTTCACACAAGTCTTTCAATGTATAACAAAAACTTTTTAGATACATTTAG

ACTATAGAACTAACAAGTTATATTTTGAACC-AAAAAACAAAGAGAGAGAGAGAGGAACA

TAGAGAAAAGAAGATATGAGAGTTTTTTTTTTTTTT-----CTAAAACAGAAAGAAACTC

ATTATATAATAAACAAATTACTTTGAGACAAATTAACGTATGTAATAAAACAAAATCAAA

GTGGTAATTAAAATTATTTAATGGTAAACAGTCTAATAGTTAAAATAAAAATGGAAATCA

CATAAATTTTGTAATTGGCCCATTAAAAACAACACTAAGCTTTTAATTTGATTTTGAAAT

TCAAAATAATTTTATTAAATCACAAAGTAAAAGGTTTACAAAGCCGATCATGACAGTGCA

TGAGGGAGGCAAATCGGCATATTTGCACTACAAAAGG-ACCCATGAAGTCTCTGAACATG

CACGCAACACTTTAATCTCTTATTAGTTACTTTGAAAGCTTATTTATATATATAGACACG

CGTAAAAACTTCTTAACCAAGATTTTTTT-ACGTGCTTCC-TTCGCGTTTAATTTGGACC

ATCAAACCGTGCTCAACAGATAAAGAAAAGGGTGCTTTTGATTCAAGATATTGGCC-GAA

AAACACAAGATAGATCCTT-CGATAGATTAAGCCACGCATGAAACGCGAATCCAAAGTGA

TGAAGAAGTGCAGATAGATATTCGTTCACCATATAGAGGAGAATATATCATTCCTACAAA

CAAAATTGATGATGTTTCTAGAATAGAAACGTTAGAATCGAATATTCATCTGTTCCGGGT

GGGGAAGGGCTACTGAAAGGCTGAACTTTTTTAAGAGTTCGAACTTCGAAAGTCAATCAA

TGATTCTTAATTGAGAGGGAAAAGCTATAAAGGACGAGAAGGGAAGGAAATGTTTATAGG

GAATGAATTTCTAATGCATGATGGGAATGACAGATATGAGGAATATATAAAGGTGATTCT

TATATACATTGACATGGATCGGAATCTATTAACTTAAAGTTATTGGGGTCGTGAAATTTA

TTTAATTTTTCTAATTCGTGTCACATGTCACGACAATTAGTGGGG-AACATTTATGTATA

TAGAAATTTCAGAAATTTCTAGCAGCGTGATAAATATAAAGATTTTGGCACAATAAGTTT

TTGGATCATAATGAGTTT-CTATTTAATCAA-GGCA-TGTAA-TCTATTTTATTATTTAG

-TAGGG-AAACTGAAAACTTAGGGTTGCTATTTGTAGCTCCCACCCTTCTTAGTTCTTAC

TCTTCTCAATATTTTTTAAAAGTTTTA-TATAATACCT-AAATT-GCCCTCCTCCT----

----------------------------------------CACCCCATTGCTCTTCCGTT

GCTCCTCCATGTG-AGTTCTTGTCTTTTTTTTT-CTCTGGTAGCGTTTTGCTGCTCCTTT

TTTCACTCAAGTGTTGCCAATTAA-TTGACAAAAAATGGTTTCTGTTTCATATAGAAACT

ATGTTTTTGTTGTGTAGTCATACATTACGGAATCTAGTTT-CCATTAAATAAGTAAC--G

TGAAAAAAAA--TAAAAGGTGAAATATATATTGTTGGAAAAGAAGCTATGAGGTGCAAGA

ACCGATCACATGGAGAAGGCAATGAAAGACAAGGAGGAGCAATGGAAGA-----------

---------GAGAAAATGAGAAGATGGAAGGGATGTGAAAATGTTTGAAAAAAACGAGGT

GATCAGTTTTAAAATACGAATTTAGTATTTTCTTTTTAAGAAGATTCTTTCG-AAAGTCG

TGTTTTAAAACATGACTTTTATT-ATTTGAAGTCG---TGCTCTAAAACATGACTTA--T

TCATATCCTT-AATATTTTT---------AAAATTTATCCATTTGTAATATTTTTTAAAA

ATTGACCCATATATGTAAAATACCCGTCAAGATCTCTTTATTATTTTGAAAGCGAAAGCA

TATCACTTCAAACACAATGGAATCGAGGCTATTGACTAAGTATAAATAGAGAAGACTTCA

TATCGGGGTTCATAATTCATAACAAAGCAAACGAGTATATAAGAAAGCATAAGCCAAATT

TTGAGTAAACTAGTGTGCACACTATCCCAGGCCTAGTGGAAGTAGGGATCCTCTCGTTGT

TGGGGGAGTAATTGGGGATGTATTGGATCCTTTTGAATATTCTATTCCTATGAGGGTTAC

CTACAATAACAGAGATGTCAGCAATGGATGTGAATTCAAACCCTCACAAGTCGTCAACCA

ACCAAGGGTAAATATCGGTGGTGATGACC

>CS48.D6

TATTAGACTCCTAATTTAATATTCTTGTTTTATTATAATGCTGATAAGTCTTGTAAATAA

GGGTGAGAAGCACGAATAATTAGTTCATGAGATGTGTATAATTATTATCCTACACGACTT

ATCTTTGATATTTCACACAAGTCTTTCAATGTATAACAAAAACTTTTTAGATACATTTAG

ACTATAGAACTAACAAGTTATATTTTGAACC-AAAAAACAAAGAGAGAGAGAGAGGAACA

TAGAGAAAAGAAGATATGAGAGTTTTTTTTTTTTTTTTT--CTAAAACAGAAAGAAACTC

ATTATATAATAAACAAATTACTTTGAGACAAATTAACGTATGTAATAAAACAAAATCAAA

GTGGTAATTAAAATTATTTAATGGTAAACAGTCTAATAGTTAAAATAAAAATGGAAATCA

CATAAATTTTGTAATTGGCCCATTAAAAACAACACTAAGCTTTTAATTTGATTTTGAAAT

TCAAAATAATTTTATTAAATCACAAAGTAAAAGGTTTACAAAGCCGATCATGACAGTGCA

TGTGGGAGGCAAATCGGCATATTTGCACTACAAAAGG-ACCCATGAAGTCTCTGAACATG

CACGCAACACTTTAATCTCTTATTAGTTACTTTGAAAGCTTATTTATATATATAGACACG

CGTAAAAACTTCTTAACCAAGATTTTTTT-ACGTGCTTCC-TTCGCGTTTAATTTGGACC

ATCAAACCGTGCTCAACAGATAGAGAAAAGGGTGCTTTTGATTCAAGATATTGGCC-GAA

AAACACAAGATAGATCCTT-CGATAGATTAAGCCACGCATGAAACGCGAATCCAAAGTGA

TGAAGAAGTGCAGATAGATATTCGTTCACCATATAGAGGAGAATATATCATTCCTACAAA

CAAAATTGATGATGTTTCTAGAATAGAAACGTTAGAATCGAATATTCATCTGTTCCGGGT

GGGGAAGGGCTACTGAAAAGCTGAACTTTTTTAAGAGTTCGAACTTCGAAAGTCAATCAA

TGATTCTTAATTGAGAGGGAAAAGCTATAAAGGACGAGAAGGGAAGGAAATGTTTATAGG

GAATGAATTTCTAATGCATGATGGGAATGACAGATATGAGGAATATATAAAGGTGATTCT

TATATACATTGACATGGATCGGAATCTATTAACTTAAAGTTATTGGGGTCGTGAAATTTA

TTTAATTTTTCTAATTCGTGTCACATGTCACGACAATTAGTGGGG-AACATTTATGTATA

TAGAAATTTCAGAAATTTCTAGCAGCGTGATAAATATAAAGATTTTGGCACAATAAGTTT

TTGGATCATAATGAGTTT-CTATTTAATCAA-GGCA-TGTAA-TCTATTTTATTATTTAG

-TAGGG-AAACTGAAAACTTAGGGTTGCTATTTGTAGCTCCCACCCTTCTTAGTTCTTAC

TCTTTTCAATATTTTTTAAAAGTTTTA-TATAATACCT-AAATT-GCCCTCCTCCT----

----------------------------------------CACCCCATTGCTCTTCCGTT

GCTCCTCCATGTG-AGTTCTTGTCTTTTTTTTT-CTCTGGTAGCGTTTTGCTGCTCCTTT

TTTCACTCAAGTGTTGCCAATTAA-TTGACAAAAAATGGTTTCTGTTTCATATAGAAACT

ATGTTTTTGTTGTGTAGTCATACATTACGGAATCTAGTTT-CCATTAAATAAGTAAC--G

TGAAAAAAA---TAAAAGGTGAAATATATATTGTTGGAAAAGAAGCTATGAGGTGCAAGA

ACCGATCACATGGAGAAGGCAATGAAAGACAAGGAGGAGCAATGGAAGA-----------

---------GAGAAGATGAGAAGATGGAAGGGATGTGAAAATGTTTGAAAAAAACGAGGT

GATCAGTTTTAAAATACGAATTTAGTATTTTCTTTTTAAGAAAATTCTTTCG-AAAGTCG

TGTTTTAAAACATGACTTTTATT-ATTTGAAGTCG---TGTTCTAAAACATGACTTA--T

TCATATCCTT-AATATTTTT---------AAAATTTATCCATTTGTAATATTTTTTAAAA

ATTGACCCATATATGTAAAATACCCGTCAAGATCTCTTTATTATTTTGAAAGCGAAAGCA

TATCACTTCAAACACAACGGAATCGAGGCTATTGACTAAGTATAAATAGAGAAGACTTCA

CATCGGGGTTCATAATTCATAACAAAGCAAACGAGTATATAAGAAAGCATAAGCCAAATT

TTGAGTAAACTAGTGTGCACACTATCCCATGCCTAGTGGAAGTAGGGATCCTCTCGTTGT

TGGGGGAGTAATTGGGGATGTATTGGATCCTTTTGAATATTCTATTCCTATGAGGGTTAC

CTACAATAACAGAGATGTCAGCAATGGATGTGAATTCAAACCCTCACAAGTTGTCAACCA

ACCAAGGGTAAATATCGGTGGTGATGACC

>CS25.01

TATTAGACTCCTAATTTAATATTCTTGTTTTATTATAATGCTGATAAGTCTTGTAAATAA

GGGTGAGAAGCACGAATAATTAGTTCATGAGATGTGTATAATTATTATCCTACACGACTT

ATCTTTGATATTTCACACAAGTCTTTCAATGTATAACAAAAACTTTTTAGATACATTTAG

ACTATAGAACTAACAAGTTATATTTTGAACC-AAAAAACAAAGAGAGAGAGAGAGGAACA

TAGAGAAAAGAAGATATGAGAGTTTTTTTTTTTTTTTT---CTAAAACAGAAAGAAACTC

ATTATATAATAAACAAATTACTTTGAGACAAATTAACGTATGTAATAAAACAAAATCAAA

GTGGTAATTAAAATTATTTAATGGTAAACAGTCTAATAGTTAAAATAAAAATGGAAATCA

CATAAATTTTGTAATTGGCCCATTAAAAACAACACTAAGCTTTTAATTTGATTTTGAAAT

TCAAAATAATTTTATTAAATCACAAAGTAAAAGGTTTACAAAGCCGATCATGACAGTGCA

TGTGGGAGGCAGATCGGCATATTTGCACTACAAAAGG-ACCCATGAAGTCTCTGAACATG

CACGCAACACTTTAATCTCTTATTAGTTACTTTGAAAGCTTATTTATATATATAGACACG

CGTAAAAACTTCTTAACCAAGATTTTTTT-ACGTGCTTCC-TTCGCGTTTAATTTGGACC

ATCAAACCGTGCTCAACAGATAAAGAAAAGGGTGCTTTTGATTCAAGATATTGGCC-GAA

AAACACAAGATAGATCCTT-CGATAGATTAAGCCACGCATGAAACGCGAATCCAAAGTGA

TGAAGAAGTGCAGATAGATATTCGTTCACCATATAGAGGAGAATATATCATTCCTACAAA

CAAAATTGATGATGTTTCTAGAATAGAAACGTTAGAATCGAATATTCATCTGTTCCGGGT

GGGGAAGGGCTACTGAAAAGCTGAACTTTTTTAAGAGTTCGAACTTCGAAAGTCAATCAA

TGATTCTTAATTGAGAGGGAAAAGCTATAAAGGACGAGAAGGGAAGGAAATGTTTATAGG

GAATGAATTTCTAATGCATGATGGGAATGACAGATATGAGGAATATATAAAGGTGATTCT

TATATACATTGACATGGATCGGAATCTATTAACTTAAAGTTATTGGGGTCGTGAAATTTA

TTTAATTTTTCTAATTCGTGTCACATGTCACGACAATTAGTGGGG-AACATTTATGTATA

TAGAAATTTCAGAAATTTCTAGCAGCGTGATAAATATAAAGATTTTGGCACAATAAGTTT

TTGGATCATAATGAGTTT-CTATTTAATCAA-GGCA-TGTAA-TCTATTTTATTATTTAG

-TAGGG-AAACTGAAAACTTAGGGTTGCTATTTGTAGCTCCCACCCTTCTTAGTTCTTAC

TCTTTTCAATATTTTTTAAAAGTTTTA-TATAATACCT-AAATT-GCCCTCCTCCT----

----------------------------------------CACCCCATTGCTCTTCCGTT

GCTCCCCCATGTG-AGTTCTTGTCTTTTTTTTT-CTCTGGTAGCGTTTTGCTGCTCCTTT

TTTCACTCAAGTGTTGCCAATTAA-TTGACAAAAAATGGTTTCTGTTTCATATAGAAACT

ATGTTTTTGTTGTGTAGTCATACATTACGGAATCTAGTTT-CCATTAAATAAGTAAC--G

TGAAAAAAAA--TAAAAGGTGAAATATATATTGTTGGAAAAGAAGCTATGAGGTGCAAGA

ACCGATCACATGGAGAAGGCAATGAAAGACAAGGAGGAGCAATGGAAGA-----------

---------GAGAAAATGAGAAGATGGAAGGGATGTGAAAATGTTTGAAAAAAACGAGGT

GATCAGTTTTAAAATACGAATTTAGTATTTTCTTTTTAAGAAAATTCTTTCG-AAAGTCG

TGTTTTAAAACATGACTTTTATT-ATTTGAAGTCG---TGTTCTAAAACATGACTTA--T

TCATATCCTT-AATATTTT----------AAAATTTATCCATTTGTAATATTTTTTAAAA

ATTGACCCATATATGTAAAATACCCGTCAAGATCTCTTTATTATTTTGAAAGCGAAAGCA

TATCACTTCAAACACAATGGAATCGAGGCTATTGACTAAGTATAAATAGAGAAGACTTCA

TATCGGGGTTCATAATTCATAACAAAGCAAACGAGTATATAAGAAAGCATAAGCCAAATT

TTGAGTAAACTAGTGTGCACACTATCCCATGCCTAGTGGAAGTAGGGATCCTCTCGTTGT

TGGGGGAGTAATTGGGGATGTATTGGATCCTTTTGAATATTCTATTCCTATGAGGGTTAC

CTACAATAACAGAGATGTCAGCAATGGATGTGAATTCAAACCCTCACAAGTTGTCAACCA

ACCAAGGGTAAATATCGGTGGTGATGACC

>CS62.01

TATTAGACTCCTAATTTAATATTCTTGTTTTATTATAATGCTGATAAGTCTTGTAAATAA

GGGTGAGAAGCACGAATAATTAGTTCATGAGATGTGTATAATTATTATCCTACACGACTT

ATCTTTGATATTTCACACAAGTCTTTCAATGTATAACAAAAACTTTTTAGATACATTTAG

ACTATAGAACTAACAAGTTATATTTTGAACC-AAAAAACAAAGAGAGAGAGAGAGGAACA

TAGAGAAAAGAAGATATGAGAGTTTTTTTTTTTTTTT----CTAAAACAGAAAGAAACTC

ATTATATAATAAACAAATTACTTTGAGACAAATTAACGTATGTAATAAAACAAAATCAAA

GTGGTAATTAAAATTATTTAATGGTAAACAGTCTAATAGTTAAAATAAAAATGGAAATCA

CATAAATTTTGTAATTGGCCCATTAAAAACAACACTAAGCTTTTAATTTGATTTTGAAAT

TCAAAATAATTTTATTAAATCACAAAGTAAAAGGTTTACAAAGCCGATCATGACAGTGCA

TGTGGGAGGCAAATCGGCATATTTGCACTACAAAAGG-ACCCATGAAGTCTCTGAACATG

CACGCAACACTTTAATCTCTTATTAGTTACTTTGAAAGCTTATTTATATATATAGACACG

CGTAAAAACTTCTTAACCAAGATTTTTTT-ACGTGCTTCC-TTCGCGTTTAATTTGGACC

ATCAAACCGTGCTCAACAGATAAAGAAAAGGGTGCTTTTGATTCAAGATATTGGCC-GAA

AAACACAAGATAGATCCTT-CGATAGATTAAGCCACGCATGAAACGCGAATCCAAAGTGA

TGAAGAAGTGCAGATAGATATTCGTTCACCATATAGAGGAGAATATATCATTCCTACAAA

CAAAATTGATGATGTTTCTAGAATAGAAACGTTAGAATCGAATATTCATCTGTTCCGGGT

GGGGAAGGGCTACTGAAAAGCTGAACTTTTTTAAGAGTTCGAACTTCGAAAGTCAATCAA

TGATTCTTAATTGAGAGGGAAAAGCTATAAAGGACGAGAAGGGAAGGAAATGTTTATAGG

GAATGAATTTCTAATGCATGATGGGAATGACAGATATGAGGAATATATAAAGGTGATTCT

TATATACATTGACATGGATCGGAATCTATTAACTTAAAGTTATTGGGGTCGTGAAATTTA

TTTAATTTTTCTAATTCGTGTCACATGTCACGACAATTAGTGGGG-AACATTTATGTATA

TAGAAATTTCAGAAATTTCTAGCAGCGTGATAAATATAAAGATTTTGGCACAATAAGTTT

TTGGATCATAATGAGTTT-CTATTTAATCAA-GGCA-TGTAA-TCTATTTTATTATTTAG

-TAGGG-AAACTGAAAACTTAGGGTTGCTATTTGTAGCTCCCACCCTTCTTAGTTCTTAC

TCTTTTCAATATTTTTTAAAAGTTTTA-TATAATACCT-AAATT-GCCCTCCTCCT----

----------------------------------------CACCCCATTGCTCTTCCGTT

GCTCCTCCATGTG-AGTTCTTGTCTTTTTTTTT-CTCTGGTAGCGTTTTGCTGCTCCTTT

TTTCACTCAAGTGTTGCCAATTAA-TTGACAAAAAATGGTTTCTGTTTCATATAGAAACT

ATGTTTTTGTTGTGTAGTCATACATTACGGAATCTAGTTT-CCATTAAATAAGTAAC--G

TGAAAAAAAA--TAAAAGGTGAAATATATATTGTTGGAAAAGAAGCTATGAGGTGCAAGA

ACCGATCACATGGAGAAGGCAATGAAAGACAAGGAGGAGCAATGGAAGA-----------

---------GAGAAAATGAGAAGATGGAAGGGATGTGAAAATGTTTGAAAAAAACGAGGT

GATCAGTTTTAAAATACGAATTTAGTATTTTCTTTTTAAGAAAATTCTTTCG-AAAGTCG

TGTTTTAAAACATGACTTTTATT-ATTTGAAGTCG---TGTTCTAAAACATGACTTA--T

TCATATCCTT-AATATTTTT---------AAAATTTATCCATTTGTAATATTTTTTAAAA

ATTGACCCATATATGTAGAATACCCGTCAAGATCTCTTTATTATTTTGAAAGCGAAAGCA

TATCACTTCAAACACAATGGAATCGAGGCTATTGACTAAGTATAAATAGAGAAGACTTCA

TATCGGGGTTCATAATTCATAACAAAGCAAACGAGTATATAAGAAAGCATAAGCCAGATT

TTGAGTAAACTAGTGTGCACACTATCCCATGCCTAGTGGAAGTAGGGATCCTCTCGTTGT

TGGGGGAGTAATTGGGGATGTATTGGATCCTTTTGAATATTCTATTCCTATGAGGGTTAC

CTACAATAACAGAGATGTCAGCAATGGATGTGAATTCAAACCCTCACAAGTTGTCAACCA

ACCAAGGGTAAATATCGGTGGTGATGACC

>CS16.27

TATTAGACTCCTAATTTAATATTCTTGTTTTATTATAATGCTGATAAGTCTTGTAAATAA

GGGTGAGAAGCACGAATAATTAGTTCATGAGATGTGTATAATTATTATCCTACACGACTT

ATCTTTGATATTTCACACAAGTCTTTCAATGTATAACAAAAACTTTTTAGATACATTTAG

ACTATAGAACTAACAAGTTATATTTTGAACC-AAAAAACAAAGAGAGAGAGAGAGGAACA

TAGAGAAAAGAAGATATGAGAGTTTTTTTTTTTTTTTTT--CTAAAACAGAAAGAAACTC

ATTATATAATAAACAAATTACTTTGAGACAAATTAACGTATGTAATAAAACAAAATCAAA

GTGGTAATTAAAATTATTTAATGGTAAACAGTCTAATAGTTAAAATAAAAATGGAAATCA

CATAAATTTTGTAATTGGCCCATTAAAAACAACACTAAGCTTTTAATTTGATTTTGAAAT

TCAAAATAATTTTATTAAATCACAAAGTAAAAGGTTTACAAAGCCGATCATGACAGTGCA

TGTGGGAGGCAAATCGGCATATTTGCACTACAAAAGG-ACCCATGAAGTCTCTGAACATG

CACGCAACACTTTAATCTCTTATTAGTTACTTTGAAAGCTTATTTATATATATAGACACG

CGTAAAAACTTCTTAACCAAGATTTTTTT-ACGTGCTTCC-TTCGCGTTTAATTTGGACC

ATCAAACCGTGCTCAACAGATAAAGAAAAGGGTGCTTTTGATTCAAGATATTGGCC-GAA

AAACACAAGATAGATCCTT-CGATAGATTAAGCCACGCATGAAACGCGAATCCAAAGTGA

TGAAGAAGTGCAGATAGATATTCGTTCACCATATAGAGGAGAATATATCATTCCTACAAA

CAAAATTGATGATGTTTCTAGAATAGAAACGTTAGAATCGAATATTCATCTGTTCCGGGT

GGGGAAGGGCTACTGAAAAGCTGAACTTTTTTAAGAGTTCGAACTTCGAAAGTCAATCAG

TGATTCTTAATTGAGAGGGAAAAGCTATAAAGGACGAGAAGGGAAGGAAATGTTTATAAG

GAATGAATTTCTAATGCATGATGGGAATGACAGATATGAGGAATATATAAAGGTGATTCT

TATATACATTGACATGGATCGGAATCTATTAACTTAAAGTTATTGGGGTCGTGAAATTTA

TTTAATTTTTCTAATTCGTGTCACATGTCACGACAATTCGTGGGG-AACATTTATGTATA

TAGAAATTTCAGAAATTTCTAGCAGCGTGATAAATATAAAGATTTTGGCACAATAAGTTT

TTGGATCATAATGAGTTT-CTATTTAATCAA-GGCA-TGTAA-TCTATTTTATTATTTAG

-TAGGG-AAACTGAAAACTTAGGGTTGCTATTTGTAGCTCCCACCCTTCTTAGTTCTTAC

TCTTTTCAATATTTTTTAAAAGTTTTA-TATAATACCT-AAATT-GCCCTCCTCCT----

----------------------------------------CACCCCATTGCTCTTCCGTT

GCTCCTCCATGTG-AGTTCTTGTCTTTTTTTTT-CTCTGGTAGCGTTTTGCTGCTCCTTT

TTTCACTCAAGTGTTGCCAATTAA-TTGACAAAAAATGGTTTCTGTTTCATATAGAAACT

ATGTTTTTGTTGTGTAGTCATACATTACGGAATCTAGTTT-CCATTAAATAAGTAAC--G

TGAAAAAAAA--TAAAAGGTGAAATATATATTGTTGGAAAAGAAGCTATGAGGTGCAAGA

ACCGATCACATGGAGAAGGCAATGAAAGACAAGGAGGAGCAATGGAAGA-----------

---------GAGAAAATGAGAAGATGGAGGGGATGTGAAAATGTTTGAAAAAAACGAGGT

GATCAGTTTTAAAATACGAATTTAGTATTTTCTTTTTAAGAAAATTCTTTCG-AAAGTCG

TGTTTTAAAACATGACTTTTATT-ATTTGAAGTCG---TGTTCTAAAACATGACTTA--T

TCATATCCTT-AATATTTTT---------AAAATTTATCCATTTGTAATATTTTTTAAAA

ATTGACCCATATATGTAAAATACCCGTCAAGATCTCTTTATTATTTTGAAAGCGAAAGCA

TATCACTTCAAACACAATGGAATCGAGGCTATTGACTAAGTATAAATAGAGAAGACTTCA

TATCGGGGTTCGTAATTCATAACAAAGCAAACGAGTATATAAGAAAGCATAAGCCAAATT

TTGAGTAAACTAGTGTGCACACTATCCCATGCCTAGTGGAAGTAGGGATCCTCTCGTTGT

TGGGGGGGTAATTGGGGATGTATTGGATCCTTTTGAATATTCTATTCCTATGAGGGTTAC

CTACAATAACAGAGATGTCAGCAATGGATGTGAATTCAAACCCTCACAAGTTGTCAACCA

ACCAAGGGTAAATATCGGTGGTGATGACC

>CS20.45

TATTAGACTCCTAATTTAATATTCTTGTTTTATTATAATGCTGATAAGTCTTGTAAATAA

GGGTGAGAAGCACGAATAATTAGTTCATGAGATGTGTATAATTATTATCCTACACGACTT

ATCTTTGATATTTCACACAAGTCTTTCAATGTATAACAAAAACTTTTTAGATACATTTAG

ACTATAGAACTAACAAGTTATATTTTGAACC-AAAAAACAAAGAGAGAGAGAGAGGAACA

TAGAGAAAAGAAGATATGAGAGTTTTTTTTTTTTTTTT---CTAAAACAGAAAGAAACTC

ATTATATAATAAACAAATTACTTTGAGACAAATTAACGTATGTAATAAAACAAAATCAAA

GTGGTAATTAAAATTATTTAATGGTAAACAGTCTAATAGTTAAAATAAAAATGGAAATCA

CATAAATTTTGTAATTGGCCCATTAAAAACAACACTAAGCTTTTAATTTGATTTTGAAAT

TCAAAATAATTTTATTAAATCACAAAGTAAAAGGTTTACAAAGCCGATCATGACAGTGCA

TGTGGGAGGCAAATCGGCATATTTGCACTACAAAAGG-ACCCATGAAGTCTCTGAACATG

CACGCAACACTTTAATCTCTTATTAGTTACTTTGAAAGCTTATTTATATATATAGACACG

CGTAAAAACTTCTTAACCAAGATTTTTTT-ACGTGCTTCC-TTCGCGTTTAATTTGGACC

ATCAAACCGTGCTCAACAGATAAAGAAAAGGGTGCTTTTGATTCAAGATATTGGCC-GAA

AAACACAAGATAGATCCTT-CGATAGATTAAGCCACGCATGAAACGCGAATCCAAAGTGA

TGAAGAAGTGCAGATAGATATTCGTTCACCATATAGAGGAGAATATATCATTCCTACAAA

CAAAATTGATGATGTTTCTAGAATAGAAACGTTAGAATCGAATATTCATCTGTTCCGGGT

GGGGAAGGGCTACTGAAAAGCTGAACTTTTTTAAGAGTTCGAACTTCGAAAGTCAATCAA

TGATTCTTAATTGAGAGGGAAAAGCTATAAAGGACGAGAAGGGAAGGAAATGTTTATAGG

GAATGAATTTCTAATGCATGATGGGAATGACAGATATGAGGAATATATAAAGGTGATTCT

TATATACATTGACATGGATCGGAATCTATTAACTTAAAGTTATTGGGGTCGTGAAATTTA

TTTAATTTTTCTAATTCGTGTCACATGTCACGGCAATTAGTGGGG-AACATTTATGTATA

TAGAAATTTCAGAAATTTCTAGCAGCGTGATAAATATAAAGATTTTGGCACAATAAGTTT

TTGGATCATAATGAGTTT-CTATTTAATCAA-GGCA-TGTAA-TCTATTTTATTATTTAG

-TAGGG-AAACTGAAAACTTAGGGTTGCTATTTGTAGCTCCCACCCTTCTTAGTTCTTAC

TCTTTTCAATATTTTTTAAAAGTTTTA-TATAATACCT-AAATT-GCCCTCCTCCT----

----------------------------------------CACCCCATTGCTCTTCCGTT

GCTCCTCCATGTG-AGTTCTTGTCTTTTTTTTT-CTCTGGTAGCGTTTTGCTGCTCCTTT

TTTCACTCAAGTGTTGCCAATTAA-TTGACAAAAAATGGTTTCTGTTTCATATAGAAACT

ATGTTTTTGTTGTGTAGTCATACATTACGGAATCTAGTTT-CCATTAAATAAGTAAC--G

TGAAAAAAAA--TAAAAGGTGAAATATATATTGTTGGAAAAGAAGCTATGAGGTGCAAGA

ACCGATCACATGGAGAAGGCAATGAAAGACAAGGAGGAGCAATGGAAGA-----------

---------GAGAAAATGAGAAGATGGAAGGGATGTGAAAATGTTTGAAAAAAACGAGGT

GATCAGTTTTAAAATACGAATTTAGTATTTTCTTTTTAAGAAAATTCTTTCG-AAAGTCG

TGTTTTAAAACATGACTTTTATT-ATTTGAAGTCG---TGTTCTAAAACATGACTTA--T

TCATATCCTT-AATATTTTT---------AAAATTTATCCATTTGTAATATTTTTTAAAA

ATTGACCCATATATGTAAAATACCCGTCAAGATCTCTTTATTATTTTGAAAGCGAAAGCA

TATCACTTCAAACACAATGGAATCGAGGCTATTGACTAGGTATAAATAGAGAAGACTTCA

TATCGGGGTTCATAATTCATAACAAAGCAAACGAGTATATAAGAAAGCATAAGCCAAATT

TTGAGTAAACTAGTGTGCACACTATCCCATGCCTAGTGGAAGTAGGGATCCTCTCGTTGT

TGGGGGAGTAATTGGGGATGTATTGGATCCTTTTGAATATTCTATTCCTATGAGGGTTAC

CTACAATAACAGAGATGTCAGCAATGGATGTGAATTCAAACCCTCACAAGTTGTCAACCA

ACCAAGGGTAAATATCGGTGGTGATGACC

>CS40.78

TATTAGACTCCTAATTTAATATTCTTGTTTTATTATAATGCTGATAAGTCTTGTAAATAA

GGGTGAGAAGCACGAATAATTAGTTCATGAGATGTGTATAATTACTATCCTACACGACTT

ATCTTTGATATTTCACACAAGTCTTTCAATGTATAACAAAAACTTTTTAGATACATTTAG

ACTATAGAACTAACAAGTTATATTTTGAACC-AAAAAACAAAGAGAGAGAGAGAGGAACA

TAGAGAAAAGAAGATATGAGAGTTTTTTTTTTTTTTTT---CTAAAACAGAAAGAAACTC

ATTATATAATAAACAAATTACTTTGAGACAAATTAACGTATGTAATAAAACAAAATCAAA

GTGGTAATTAAAATTATTTAATGGTAAACAGTCTAATAGTTAAAATAAAGATGGAAATCA

CATAAATTTTGTAATTGGCCCATTAAAAACAACACTAAGCTTTTAATTTGATTTTGAAAT

TCAAAATAATTTTATTAAATCACAAAGTAAAAGGTTTACAAAGCCGATCATGACAGTGCA

TGTGGGAGGCAAATCGGCATATTTGCACTACAAAAGG-ACCCATGAAGTCTCTGAACATG

CACGCAACACTTTAATCTCTTATTAGTTACTTTGAAAGCTTATTTATATATATAGACACG

CGTAAAAACTTCTTAACCAAGATTTTTTT-ACGTGCTTCC-TTCGCGTTTAATTTGGACC

ATCAAACCGTGCTCAACAGATAAAGAAAAGGGTGCTTTTGATTCAAGATATTGGCC-GAA

AAACACAAGATAGATCCTT-CGATAGATTAAGCCACGCATGAAACGCGAATCCAAAGTGA

TGAAGAAGTGCAGATAGATATTCGTTCACCATATAGAGGAGAATATATCATTCCTACAAA

CAAAATTGATGATGTTTCTAGAATAGAAACGTTAGAATCGAATATTCATCTGTTCCGGGT

GGGGAAGGGCTACTGAAAAGCTGAACTTTTTTAAGAGTTCGAACTTCGAAAGTCAATCAA

TGATTCTTAATTGAGAGGGAAAAGCTATAAAGGACGAGAAGGGAAGGAAATGTTTATAGG

GAATGAATTTCTAATGCATGATGGGAATGACAGATATGAGGAATATATAAAGGTGATTCT

TATATACATTGACATGGATCGGAATCTATTAACTTAAAGTTATTGGGGTCGTGAAATTTA

TTTAATTTTTCTAATTCGTGTCACATGTCACGACAATTAGTGGGG-AACATTTATGTATA

TAGAAATTTCAGAAATTTCTAGCAGCGTGATAAATATAAAGATTTTGGCACAATAAGTTT

TTGGATCATAATGAGTTT-CTATTTAATCAA-GGCA-TGTAA-TCTATTTTATTATTTAG

-TAGGG-AAACTGAAAACTTAGGGTTGCTATTTGTGGCTCCCACCCTTCTTAGTTCTTAC

TCTTTTCAATATTTTTTAAAAGTTTTA-TATAATACCT-AAATT-GCCCTCCTCCT----

----------------------------------------CACCCCATTGCTCTTCCGTT

GCTCCTCCATGTG-AGTTCTTGTCTTTTTTTTT-CTCTGGTAGCGTTTTGCTGCTCCTTT

TTTCACTCAAGTGTTGCCAATTAA-TTGACAAAAAATGGTTTCTGTTTCATATAGAAACT

ATGTTTTTGTTGTGTAGTCATACATTACGGAATCTAGTTT-CCATTAAATAAGTAAC--G

TGAAAAAAAA--TAAAAGGTGAAATATATATTGTTGGAAAAGAAGCTATGAGGTGCAAGA

ACCGATCACATGGAGAAGGCAATGAAAGACAAGGAGGAGCAATGGAAGA-----------

---------GAGAAAATGAGAAGATGGAAGGGATGTGAAAATGTTTGAAAAAAATGAGGT

GATCAGTTTTAAAATACGAATTTAGTATTTTCTTTTTAAGAAAATTCTTTCG-AAAGTCG

TGTTTTAAAACATGACTTTTATT-ATTTGAAGTCG---TGTTCTAAAACATGACTTA--T

TCATATCCTT-AATATTTTT---------AAAATTTATCCATTTGTAATATTTTTTAAAA

ATTGACCCATATATGTAAAATACCCGTCAAGATCTCTTTATTATTTTGAAAGCGAAAGCA

TATCACTTCAAACACAATGGAATCGAGGCTATTGACTAAGTATAAATAGAGAAGACTTCA

TATCGGGGTTCATAATTCATAACAAAGCAAACGAGTATATAAGAAAGCATAAGCCAAATT

TTGAGTAAACTAGTGTGCACACTATCCCATGCCTAGTGGAAGTAGGGATCCTCTCGTTGT

TGGGGGAGTAATTGGGGATGTATTGGATCCTTTTGAATATTCTATTCCTATGAGGGTTAC

CTACAATAACAGAGATGTCAGCAATGGATGTGAATTCAAACCCTCACAAGTTGTCAACCA

ACCAAGGGTAAATATCGGTGGTGATGACC

>CS49.E7

TATTAGACTCCTAATTTAATATTCTTGTTTTATTATAATGCTGATAAGTCTTGTAAATAA

GGGTGAGAAGCACGAATAATTAGTTCATGAGATGTGTATAATTATTATCCTACACGACCT

ATCTTTGATACTTCACACAAGTCTTTCAATGTATAACAAAAACTTTTTAGATACATTTAG

ACTATAGAACTAACAAGTTATATTTTGAACC-AAAAAACAAAGAGAGAGAGAGAGGAACA

TAGAGAAAAGAAGATATGAGAGTTTTTTTTTTTTTTTT---CTAAAACAGAAAGAAACTC

ATTATATAATAAACAAATTACTTTGAGACAAATTAACGTGTGTAATAAAACAAAATCAAA

GTGGTAATTAAAATTATTTAATGGTAAACAGTCTAATAGTTAAAATAAAAATGGAAATCA

CATAAATTTTGTAATTGGCCCATTAAAAACAACACTAAGCTTTTAATTTGATTTTGAAAT

TCAAAATAATTTTATTAAATCACAAAGTAAAAGGTTTACAAAGCCGATCATGACAGTGCA

TGTGGGAGGCAAATCGGCATATTTGCACTACAAAAGG-ACCCATGAAGTCTCTGAACATG

CACGCAACACTTTAATCTCTTATTAGTTACTTTGAAAGCTTATTTATATATATAGACACG

CGTAAAAACTTCTTAACCAAGATTTTTTT-ACGTGCTTCC-TTCGCGTTTAATTTGGACC

ATCAAACCGTGCTCAACAGATAAAGAAAAGGGTGCTTTTGATTCAAGATATTGGCC-GAA

AAACACAAGATAGATCCTT-CGATAGATTAAGCCACGCATGAAACGCGAATCCAAAGTGA

TGAAGAAGTGCAGATAGATATTCGTTCACCATATAGAGGAGAATATATCATTCCTACAAA

CAAAATTGATGATGTTTCTAGAATAGAAACGTTAGAATCGAATATTCATCTGTTCCGGGT

GGGGAAGGGCTACTGAAAAGCTGAACTTTTTTAAGAGTTCGAACTTCGAAAGTCAATCAA

TGATTCTTAATTGAGAGGGAAAAGCTATAAAGGACGAGAAGGGAAGGAAATGTTTATAGG

GAATGAATTTCTAATGCATGATGGGAATGACAGATATGAGGAATATATAAAGGTGATTCT

TATATACATTGACATGGATCGGAATCTATTAACTTAAAGTTATTGGGGTCGTGAAATTTA

TTTAATTTTTCTAATTCGTGTCACATGTCACGACAATTAGTGGGG-AACATTTATGTATA

TAGAAATTTCAGAAATTTCTAGCAGCGTGATAAATATAAAGATTTTGGCACAATAAGTTT

TTGGATCATAATGAGTTT-CTATTTAATCAA-GGCA-TGTAA-TCTATTTTATTATTTAG

-TAGGG-AAACTGAAAACTTAGGGTTGCTATTTGTAGCTCCCACCCTTCTTAGTTCTTAC

TCTTTTCAATATTTTTTAAAAGTTTTA-TATAATACCT-AAATT-GCCCTCCTCCT----

----------------------------------------CACCCCATTGCTCTTCCGTT

GCTCCTCCATGTG-AGTTCTTGTCTTTTTTTTT-CTCTGGTAGCGTTTTGCCGCTCCTTT

TTTCACTCAAGTGTTGCCAATTAA-TTGACAAAAAATGGTTTCTGCTTCATATGGAAACT

ATGTTTTTGTTGTGTAGTCATACATTACGGAATCTAGTTT-CCATTAAATAAGTAAC--G

TGAAAAAAAA--TAAAAGGTGAAATATATATTGTTGGAAAAGAAGCTATGAGGTGCAAGA

ACCGATCACATGGAGAAGGCAATGAAAGACAAGGAGGAGCAATGGAAGA-----------

---------GAGAAAATGAGAAGATGGAAGGGATGTGAAAATGTTTGAAAAAAACGAGGT

GATCAGTTTTAAAATACGAATTTAGTATTTTCTTTTTAAGAAAATTCTTTCG-AAAGTCG

TGTTTTAAAACATGACTTTTATT-ATTTGAAGTCG---TGTTCTAAAACATGACTTA--T

TCATATCCTT-AATATTTTT---------AAAATTTATCCATTTGTAATATTTTTTAAAA

ATTGACCCATATATGTAAAATACCCGTCAAGATCTCTTTATTATTTTGAAAGCGAAAGCA

TATCACTTCAAACACAATGGAATCGAGGCTATTGACTAAGTATAAATAGAGAAGACTTCA

TATCGGGGTTCATAATTCATAACAAAGCAAACGAGTATATAAGAAAGCATAAGCCAAATT

TTGAGTAAACTAGTGTGCACACTATCCCATGCCTAGTGGAAGTAGGGATCCTCTCGTTGT

CGGGGGAGTAATTGGGGATGTATTGGATCCTTTTGAATATTCTATTCCTATGAGGGTTAC

CTACAATAACAGAGATGTCAGCAATGGATGTGAATTCAAACCCCCACAAGTTGTCAACCA

ACCAAGGGTAAATATCGGTGGTGATGACC

>H11.B2

TATTAGACTCCTAATTTAATATTCTTGTTTTACTATAATGCTGATAAGTCTTGTAAATAA

GGGTGAGAAGCACGAATAATTAGTTCATGAGATGTGTATAATTATTATCCTACACGACTT

ATCTTTGATATTTCACACAAGTCTTTCAATGTATAACAAAAACTTTTTAGATACATTTAG

ACTATAGAACTAACGAGTTATATTTTGAACC-AAAAAACAAAGAGAGAGAGAGAGGAACA

TAGAGAAAAGAAGATATGAGAGTTTTTTTTTTTTTTTT---CTAAAACAGAAAGAAACTC

ATTATATAATAAACAAATTACTTTGAGACAAATTAACGTATGTAATAAAACAAAATCAAA

GTGGTAATTAAAATTATTTAATGGTAAACAGTCTAATAGTTAAAATAAAAATGGAAATCA

CATAAATTTTGTAATTGGCCCATTAAAAACAACACTAAGCTTTTAATTTGATTTTGAAAT

TCAAAATAATTTTATTAAATCACAAAGTAAAAGGTTTACAAAGCCGATCATGACAGTGCA

TGTGGGAGGCAAATCGGCATATTTGCACTACAAAAGG-ACCCATGAAGTCTCTGAACATG

CACGCAACACTTTAATCTCTTATTAGTTACTTTGAAAGCTTATTTATATATATAGACACG

CGTAAAAACTTCTTAACCAAGATTTTTTT-ACGTGCTTCC-TTCGCGTTTAATTTGGACC

ATCAAACCGTGCTCAACAGATAAAGAAAAGGGTGCTTTTGATTCAAGATATTGGCCCGAA

AAACACAAGACAGATCCTT-CGATAGATTAAGCCACGCACGAAACGCGAATCCAAAGTGA

TGAAGAAGTGCAGATAGATATTCGTTCGCCATATAGAGGAGAATATATCATTCCTACAAA

CAAAATTGATGATGTTTCTAGAATAGAAACGTTAGAATCGAATATTCATCTGTTCCGGGT

GGGGAAGGGCTACTGAAAAGCTGAACTTTTTTAAGAGTTCGAACTTCGAAAGTCAATCAA

TGATTCTTAATTGAGAGGGAAAAGCTATAAAGGACGAGAAGGGAAGGAAATGTTTATAGG

GAATGAATTTCTAATGCATGATGGGAATGACAGATATGAGGAATATATAAAGGTGATTCT

TATATACATTGACATGGATCGGAATCTATTAACTTAAAGTTATTGGGGTCGTGAAATTTA

TTTAATTTTTCTAATTCGTGTCACATGTCACGACAACTAGTGGGG-AACATTTATGTATA

TAGAAATTTCAGAAATTTCTAGCAGCGTGATAAATATAAAGATTTTGGCACAATAAGTTT

TTGGATCATAATGAGTTT-CTATTTAATCAA-GGCA-TGTAA-TCTATTTTATTATTTAG

-TAGGG-AAACTGAAAACTTAGGGTTGCTATTTGTAGCTCCCACCCTTCTTAGTTCTTAC

TCTTTTCAATATTTTTTAAAAGTTTTA-TATAATACCT-AAATT-GCCCTCCTCCT----

----------------------------------------CACCCCATTGCTCTTCCGTT

GCTCCTCCATGTG-AGTTCTTGTCTTTTTTTTT-CTCTGGTAGCGTTTTGCTGCTCCTTT

TTTCACTCAAGTGTTGCCAATTAA-TTGACAAAAAATGGTTTCTGTTTCATATAGAAACT

ATGTTTTTGTTGTGTAGTCATACATTACGGAATCTAGTTT-CCATTAAATAAGTAAC--G

TGAAAAAAAA--CAAAAGGTGAAATATATATTGTTGGAAAAGAAGCTATGAGGTGCAAGA

ACCGATCACATGGAGAAGGCAATGAAAGACAAGGAGGAGCAATGGAAGA-----------

---------GAGAAAATGAGAAGATGGAAGGGATGTGAAAATGTTTGAAAAAAACGAGGT

GATCAGTTTTAAAATACGAATTTAGTATTTTCTTTTTAAGAAAATTCTTTCG-AAAGTCG

TGTTTTAAAACATGACTTTTATT-ATTTGAAGTCG---TGTTCTAAAACATGACTTA--T

TCATATCCTT-AATATTTT----------AAAATTTATCCATTTGTAATATTTTTTAAAA

ATTGACCCATATATGTAAAATACCCGTCAGGATCTCTTTATTATTTTGAAAGCGAAAGCA

TATCACTTCAAACACAATGGAATCGAGGCTATTGACTAAGTATAAATAGAGAAGACTTCA

TATCGGGGTTCATAATTCATAACAAAGCAAACGAGTACATAAGAAAGCATAAGCCAAATT

TTGAGTAAATTAGTGTGCACACTATCCCATGCCTAGTGGAAGTAGGGATCCTCTCGTTGT

TGGGGGAGTAATTGGGGATGTATTGGATCCTTTTGAATATTCTATTCCTATGAGGGTTAC

CTACAATAACAGAGATGTCAGCAATGGATGTGAATTCAAACCCTCACAAGTTGTCAACCA

ACCAAGGGTAAATATCGGTGGTGATGACC

>CS43.A2

TATTAGACTCCTAATTTAATATTCTTGTTTTATTATAATGCTGATAAGTCTTGTAAATAA

GGGTGAGAAGCACGAATAATTAGTTCATGAGATGTGTATAATTATTATCCTACACGACTT

ATCTTTGATATTTCACACAAGTCTTTCAATGTATAACAAAAACTTTTTAGATACATTTAG

ACTATAGGACTAACAAGTTATATTTTGAACC-AAAAAACAAAGAGAGAGAGAGAGGAACA

TAGAGAAAAGAAGATATGAGAGTTTTTTTTTTTTTTTT---CTAAAACAGAAAGAAACTC

ATTATATAATAAACAAATTACTTTGAGACAAATTAACGTATGTAATAAAACAAAATCAAA

GTGGTAATTAAAATTATTTAATGGTAAACAGTCTAATAGTTAAAATAAAAATGGAAATCA

CATAAATTTTGTAATTGGCCCATTAAAAACAACACTAAGCTTTTAATTTGATTTTGAAAT

TCAAAATAATTTTATTAAATCACAAAGTAAAAGGTTTACAAAGCCGATCATGACAGTGCA

TGTGGGAGGCAAATCGGCATATTTGCACTACAAAAGG-ACCCATGAAGTCTCTGAACATG

CACGCAACACTTTAATCTCTTATTAGTTACTTTGAAAGCTTATTTATATATATAGACACG

CGTAAAAACTTCTTAACCAAGATTTTTTT-ACGTGCTTCC-TTCGCGTTTAATTTGGACC

ATCAAACCGTGCTCAACAGATAAAGAAAAGGGTGCTTTTGATTCAAGATATTGGCC-GAA

AAACACAAGATAGATCCTT-CGATAGATTAAGCCACGCATGAAACGCGAATCCAAAGTGA

TGAAGAAGTGCAGATAGATATTCGTTCACCATATAGAGGAGAATATATCATTCCTACAAA

CAAAATTGATGATGTTTCTAGAATAGAAACGTTAGAATCGAATATTCATCTGTTCCGGGT

GGGGAAGGGCTACTGAAAAGCTGAACTTTTTTAAGAGTTCGAACTTCGAAAGTCAATCAA

TGATTCTTAATTGAGAGGGAAAAGCTATAAAGGACGAGAAGGGAAGGAAATGTTTATAGG

GAATGAATTTCTAATGCATGATGGGAATGACAGATATGAGGAATATATAAAGGTGATTCT

TATATACATTGACATGGATCGGAATCTATTAACTTAAAGTTATTGGGGTCGTGAAATTTA

TTTAATTTTTCTAATTCGTGTCACATGTCACGACAATTAGTGGGG-AACATTTATGTATA

TAGAAATTTCAGAAATTTCTAGCAGCGTGATAAATATAAAGATTTTGGCGCAATAAGTTT

TTGGATCATAATGAGTTT-CTATTTAATCAA-GGCA-TGTAA-TCTATTTTATTATTTAG

-TAGGG-AAACTGAAAACTTAGGGTTGCTATTTGTAGCTCCCACCCTTCTTAGTTCTTAC

TCTTTTCAATATTTTTTAAAAGTTTTA-TATAATACCT-AAATT-GCCCTCCTCCT----

----------------------------------------CACCCCATTGCTCTTCCGTT

GCTCCTCCATGTG-AGTTCTTGTCTTTTTTTTT-CTCTGGTAGCGTTTTGCTGCTCCTTT

TTTCACTCAAGTGTTGCCAATTAA-TTGACAAAAAACGGTTTCTGTTTCATATAGAAACT

ATGTTTTTGTTGTGTAGTCATACATTACGGAATCTAGTTT-CCATTAAATAAGTAAC--G

TGAAAAAAAA--TAAAAGGTGAAATATATATTGTTGGAAAAGAAGCTATGAGGTGCAAGA

ACCGATCACATGGAGAAGGCAATGAAAGACAAGGAGGAGCAATGGAAGA-----------

---------GAGAAAATGAGAAGATGGAAGGGATGTGAAAATGTTTGAAAAAAACGAGGT

GATCAGTTTTAAAATACGAATTTAGTATTTTCTTTTTAAGAAAATTCTTTCG-AAAGTCG

TGTTTTAAAACATGACTTTTATT-ATTTGAAGTCG---TGTTCTAAAACATGACTTA--T

TCATATCCTT-AATATTTTT---------AAAATTTATCCATTTGTAATATTTTTTAAAA

ATTGACCCATATATGTAAAATACCCGTCAAGATCTCTTTATTATTTTGAAAGCGAAAGCA

TATCACTTCAAACACAATGGAATCGAGGCTATTGACTAAGTATAAATAGAGAAGACTTCA

TATCGGGGTTCATAGTTCATAACAAAGCAAACGAGTATATAAGAAAGCATAAGCCAAATT

TTGAGTAAACTAGTGTGCACACTATCCCATGCCTAGTGGAAGTAGGGATCCTCTCGTTGT

TGGGGGAGTAATTGGGGATGTATTGGATCCTTTTGAATATTCTATTCCTATGAGGGTTAC

CTACAATAACAGAGATGTCAGCAATGGATGTGAATTCAAACCCTCACAAGTTGTCAACCA

ACCAAGGGTAAATATCGGTGGTGATGACC

>CS50.F8

TATTAGACTCCTAATTTAATATTCTTGTTTTATTATAATGCTGATAAGTCTTGTAAATAA

GGGTGAGAAGCACGAATAATTAGTTCATGAGATGTGTATAATTATTATCCTACACGACTT

ATCTTTGATATTTCACACAAGTCTTTCAATGTATAACAAAAACTTTTTAGATACATTTAG

ACTATAGAACTAACAAGTTATATTTTGAACC-AAAAAACAAAGAGAGAGAGAGAAGAACA

TAGAGAAAAGAAGATATGAGAGTTTTTTTTTTTTTTTT---CTAAAACAGAAAGAAACTC

ATTATATAATAAACAAATTACTTTGAGACAAATTAACGTATGTAATAAAACAAAATCAAG

GTGGTAATTAAAATTATTTAATGGTAAACAGTCTAATAGTTAAAATAAAAATGGAAATCA

CATAAATTTTGTAATTGGCCCATTAAAAACAACACTAAGCTTTTAATTTGATTTTGAAAT

TCAAAATAATTTTATTAAATCACAAAGTAAAAGGTTTACAAAGCCGATCATGACAGTGCA

TGTGGGAGGCAAATCGGCATATTTGCACTACAAAAGG-ACCCATGAAGTCTCTGAACATG

CACGCAACACTTTAATCTCTTATTAGTTACTTTGAAAGCTTATTTATATATATAGACACG

CGTAAAAACTTCTTAACCAAGATTTTTTT-ACGTGCTTCC-TTCGCGTTTAATTTGGACC

ATCAAACCGTGCTCAACAGATAAAGAAAAGGGTGCTTTTGATTCAAGATATTGGCC-GAA

AAACACAAGATAGATCCTT-CGATAGATTAAGCCACGCATGAAACGCGAATCCAAAGTGA

TGAAGAAGTGCAGATAGATATTCGTTCACCATATAGAGGAGAATATATCATTCCTACAAA

CAAAATTGATGATGTTTCTAGAATAGAAACGTTAGAATCGAATATTCATCTGTTCCGGGT

GGGGAAGGGCTACTGAAAAGCTGAACTTTTTTAAGAGTTCGAACTTCGAAAGTCAATCAA

TGATTCTTAATTGAGAGGGAAAAGCTATAAAGGACGAGAAGGGAAGGAAATGTTTATAGG

GAATGAATTTCTAATGCATGATGGGAATGACAGATATGAGGAATATATAAAGGTGATTCT

TATATACATTGACATGGATCGGAATCTATTAACTTAAAGTTATTGGGGTCGTGAAATTTA

TTTAATTTTTCTAATTCGTGTCACATGTCACGACAATTAGTGGGG-AACATTTATGTATA

TAGAAATTTCAGAAATTTCTAGCAGCGTGATAAATATAAAGATTTTGGCACAATAAGTTT

TTGGATCATAATGAGTTT-CTATTTAATCAA-GGCA-TGTAA-TCTATTTTATTATTTAG

-TAGGG-AAACTGAAGACTTAGGGTTGCTATTTGTAGCTCCCACCCTTCTTAGTTCTTAC

TCTTTTCAATATTCTTTAAAAGTTTTA-TATAATACCT-AAATT-GCCCTCCTCCT----

----------------------------------------CACCCCATTGCTCTTCCGTT

GCTCCTCCATGTG-AGTTCTTGTCTTTTTTTTT-CTCTGGTAGCGTTTTGCTGCTCCTTT

TTTCACTCAAGTGTTGCCAATTAA-TTGACAAAAAATGGTTTCTGTTTCATATAGAAACT

ATGTTTTTGTTGTGTAGTCATACATTACGGAATCTAGTTT-CCATTAAATAAGTAAC--G

TGAAAAAAAA--TAAAAGGTGAAATATATATTGTTGGAAAAGAAGCTATGAGGTGCAAGA

ACCGATCACATGGAGAAGGCAATGAAAGACAAGGAGGAGCAATGGAAGA-----------

---------GAGAAAATGAGAAGATGGAAGGGATGTGAAAATGTTTGAAAAAAACGAGGT

GATCAGTTTTAAAATACGAATTTAGTATTTTCTTTTTAAGAAAATTCTTTCG-AAAGTCG

TGTTTTAAAACATGACTTTTATT-ATTTGAAGTCG---TGTTCTAAAACATGACTTA--T

TCATATCCTT-AATATTTTT---------AAAATTTATCCATTTGTAATATTTTTTAAAA

ATTGACCCATATATGTAAAATACCCGTCAAGATCTCTTTATTATTTTGAAAGCGAAAGCA

TATCACTTCAAACACAATGGAATCGAGGCTATTGACTAAGTATAAATAGAGAAGACTTCA

TATCGGGGTTCATAATTCATAACAAAGCAAACGAGTATATAAGAAAGCATAAGCCAAATT

TTGAGTAAACTAGTGTGCACACTATCCCATGCCTAGTGGAAGTAGGGATCCTCTCGTTGT

TGGGGGAGTAATTGGGGATGTATTGGATCCTTTTGAATATTCTATTCCTATGAGGGTTAC

CTACAATAACAGAGATGTCAGCAATGGATGTGAATTCAAACCCTCACAAGTTGTCAACCA

ACCAAGGGTAAATATCGGTGGTGATGACC

>CS19.34

TATTAGACTCCTAATTTAATATTCTTGTTTTATTATAATGCTGATAAGTCTTGTAAATAA

GGGTGAGAAGCACGAATAATTAGTTCATGAGATGTGTATAATTATTATCCTACACGACTT

ATCTTTGATATTTCACACAAGTCTTTCAATGTATAACAAAAACTTTTTAGATACATTTAG

ACTATAGAACTAACAAGTTATATTTTGAACC-AAAAAACAAAGAGAGAGGGAGAGGAACA

TAGAGAAAAGAAGATATGAGAGTTTTTTTTTTTTTTTTT--CTAAAACAGAAAGAAACTC

ATTATATAATAAACAAATTACTTTGAGACAAATTAACGTATGTAATAAAACAAAATCAAA

GTGGTAATTAAAATTATTTAATGGTAAACAGTCTAATAGTTAAAATAAAAATGGAAATCA

CATAAATTTTGTAATTGGCCCATTAAAAACAACACTAAGCTTTTAATTTGATTTTGAAAT

TCAAAATAATTTTATTAAATCACAAAGTAAAAGGTTTACAAAGCCGATCATGACAGTGCA

TGTGGGAGGCAAATCGGCATATTTGCACTACAAAAGG-ACCCATGAAGTCTCTGAACATG

CACGCAACACTTTAATCTCTTATTAGTTACTTTGAAAGCTTATTTATATATATAGACACA

CGTAAAAACTTCTTAACCAAGATTTTTTT-ACGTGCTTCC-TTCGCGTTTAATTTGGACC

ATCGAACCGTGCTCAACAGATAAAGAAAAGGGTGCTTTTGATTCAAGATATTGGCC-GAA

AAACACAAGATAGATCCTT-CGATAGATTAAGCCACGCATGAAACGCGAATCCAAAGTGA

TGAAGAAGTGCAGATAGATATTCGTTCACCATATAGAGGAGAATATATCATTCCTACAAA

CAAAATTGATGATGTTTCTAGAATAGAAACGTTAGAATCGAATATTCATCTGTTCCGGGT

GGGGAAGGGCTACTGAAAAGCTGAACTTTTTTAAGAGTTCGAACTTCGAAAGTCAATCAA

TGATTCTTAATTGAGAGGGAAAAGCTATAAAGGACGAGAAGGGAAGGAAATGTTTATAGG

GAATGAATTTCTAATGCATGATGGGAATGACAGATATGAGGAATATATAAAGGTGGTTCT

TATATACATTGACATGGATCGGAATCTATTAACTTAAAGTTATTGGGGTCGTGAAATTTA

TTTAATTTTTCTAATTCGTGTCACATGTCACGACAATTAGTGGGG-AACATTTATGTATA

TAGAAATTTCAGAAATTTCTAGCAGCGTGATAAATATAAAGATGTTGGCACAATAAGTTT

TTGGATCATAATGAGTTT-CTATTTAATCAA-GGCA-TGTAA-TCTATTTTATTATTTAG

-TAGGG-AAACTGAAAACTTAGGGTTGCTATTTGTAGCTCCCACCCTTCTTAGTTCTTAC

TCTTTTCAATATTTTTTAAAAGTTTTA-TATAATACCT-AAATT-GCCCTCCTCCT----

----------------------------------------CACCCCATTGCTCTTCCGTT

GCTCCTCCATGTG-AGTTCTTGTCTTTTTTTTT-CTCTGGTAGCGTTTCGCTGCTCCTTT

TTTCACTCAAGTGTTGCCAATTAA-TTGACAAAAAATGGTTTCTGTTTCATATAGAAACT

ATGTTTTTGTTGTGTAGTCATACATTACGGAATCTAGTTT-CCATTAAATAAGTAAC--G

TGAAAAAAAA--TAAAAGGTGAAATATATATTGTTGGAAAAGAAGCTATGAGGTGCAAGA

ACCGATCACATGGAGAAGGCAATGAAAGACAAGGAGGAGCAATGGAAGA-----------

---------GAGAAAATGAGAAGATGGAAGGGATGTGAAAATGTTTGAAAAAAACGAGGT

GATCAGTTTTAAAATACGAATTTAGTATTTTCTTTTTAAGAAAATTCTTTCG-AAAGTCG

TGTTTTAAAACATGACTTTTATT-ATTTGAAGTCG---TGTTCTAAAACATGACTTA--T

TCATATCCTT-AATATTTTT---------AAAATTTATCCATTTGTAATATTTTTTAAAA

ATTGACCCATATATGTAAAATACCCGTCAAGATCTCTTTATTATTTTGAAAGCGAAAGCA

TATCACTTCAAACACAATGGAATCGAGGCTATTGACTAAGTATAAATAGAGAAGACTTCA

TATCGGGGTTCATAATTCATAACAAAGCAAACGAGTATATAAGAAAGCATAAGCCAAATT

TTGAGTAAACTAGTGTGCACACTATCCCATGCCTAGTGGAAGTAGGGATCCTCTCGTTGT

TGGGGGAGTAATTGGGGATGTATTGGATCCTTTTGAATATTCTATTCCTATGAGGGTTAC

CTACAATAACAGAGATGTCAGCAATGGATGTGAATTCAAACCCTCACAAGTTGTCAACCA

ACCAAGGGTAAATATCGGTGGTGATGACC

>CS35.48

TATTAGACTCCTAATTTAATATTCTTGTTTTATTATAATGCTGATAAGTCTTGTAAATAA

GGGTGAGAAGCACGAATAATTAGTTCATGAGATGTGTATAATTATTATCCTACACGACTT

ATCTTTGATATTTCACACAAGTCTTTCAATGTATAACAAAAACTTTTTAGATACATTTAG

ACTATAGAACTAACAAGTTATATTTTGAACC-AAAAAACAAAGAGAGAGAGAGAGGAACA

TAGAGAAAAGAAGATATGAGAGTTTTTTTTTTTTTTT----CTAAAACAGAAAGAAACTC

ATTATATAATAAACAAATTACTTTGGGACAAATTAACGTATGTAATAAAACAAAATCAAA

GTGGTAATTAAAATTATTTAATGGTAAACAGTCTAATAGTTAAAATAAAAATGGAAATCA

CATAAATTTTGTAATTGGCCCATTAAAAACAACACTAAGCTTTTAATTTGATTTTGAAAT

TCAAAATAATTTTATTAAATCACAAAGTAAAAGGTTTACAAAGCCGATCATGACAGTGCA

TGTGGGAGGCAAATCGGCATATTTGCACTACAAAAGG-ACCCATGAAGTCTCTGAACATG

CACGCAACACTTTAATCTCTTATTAGTTACTTTGAAAGCTTATTTATATATATAGACGCG

CGTAAAAACTTCTTAACCAAGATTTTTTT-ACGTGCTTCC-TTCGCGTTTAATTTGGACC

ATCAAACCGTGCTCAACAGATAAAGAAAAGGGTGCTTTTGATTCAAGATATTGGCC-GAA

AAACACAAGATAGATCCTT-CGATAGATTAAGCCACGCATGAAACGCGAATCCAAAGTGA

TGAAGAAGTGCAGATAGATATTCGTTCACCATATAGAGGAGAATATATCATTCCTACAAA

CAAAATTGATGATGTTTCTAGAATAGAAACGTTAGAATCGAATATTCATCTGTTCCGGGT

GGGGAAGGGCTACTGAAAAGCTGAACTTTTTTAAGAGTTCGAACTTCGAAAGTCAATCAA

TGATTCTTAATTGAGAGGGAAAAGCTATAAAGGACGAGAAGGGAAGGAAATGTTTATAGG

GAATGAATTTCTAATGCATGATGGGAATGACAGATATGAGGAATATATAAAGGTGATTCT

TATATACATTGACATGGATCGGAATCTATTAACTTAAAGTTATTGGGGTCGTGAAATTTA

TTTAATTTTTCTAATTCGTGTCACACGTCACGACAATTAGTGGGG-AACATTTATGTATA

TAGAAATTTCAGAAATTTCTAGCAGCGTGATAAATATAAAGATTTTGGCACAATAAGTTT

TTGGATCATAATGAGTTT-CTATTTAATCAA-GGCA-TGTAA-TCTATTTTATTATTTAG

-TAGGG-AAACTGAAAACTTAGGGTTGCTATTTGTAGCTCCCACCCTTCTTAGTTCTTAC

TCTTTTCAATATTTTTTAAAAGTTTTA-TATAATACCTTAAATT-GCCCTCCTCCT----

----------------------------------------CACCCCATTGCTCTTCCGTT

GCTCCTCCATGTG-AGTTCTTGTCTTTTTTTTT-CTCTGGTAGCGTTTTGCTGCTCCTTT

TTTCACTCAAGTGTTGCCAATTAA-TTGACAAAAAATGGTTTCTGTTTCATATAGAAACT

ATGTTTTTGTTGTGTAGTCATACATTACGGAATCTAGTTT-CCATTAAATAAGTAAC--G

TGAAAAAAAA--TAAAAGGTGAAATATATATTGTTGGAAAAGAAGCTATGAGGTGCAAGA

ACCGATCACATGGAGAAGGCAATGAAAGACAAGGAGGAGCAATGGAAGA-----------

---------GAGAAAATGAGAAGATGGAAGGGATGTGAAAATGTTTGAAAAAAACGAGGT

GATCAGTTTTAAAATACGAATTTAGTATTTTCTTTTTAAGAAAATTCTTTCG-AAAGTCG

TGTTTTAAAACATGACTTTTATT-ATTTGAAGTCG---TGTTCTAAAACATGACTTA--T

TCATATCCTT-AATATTTTT---------AAAATTTATCCATTTGTAATATTTTTTAAAA

ATTGACCCATATATGTAAAATACCCGTCAAGATCTCTTTATTATTTTGAAAGCGAAAGCA

TATCACTTCAAACACAATGGAATCGAGGCTATTGACTAAGTATAAATAGAGAAGACTTCG

TATCGGGGTTCACAATTCATAACAAAGCAAACGAGTATATAAGAAAGCATAAGCCAAATT

TTGAGTAAACTAGTGTGCACACTATCCCATGCCTAGTGGAAGTAGGGATCCTCTCGTTGT

TGGGGGAGTAATTGGGGATGTATTGGATCCTTTTGAATATTCTATTCCTATGAGGGTTAC

CTACAATAACAGAGATGTCAGCAATGGATGTGAATTCAAACCCTCACAAGTTGTCAACCA

ACCAAGGGTAAATATCGGTGGTGATGACC

>CS19.37

TATTAGACTCCTAATTTAATATTCTTGTTTTATTATAATGCTGATAAGTCTTGTAAATAA

GGGTGAGAAGCACGAATAATTAGTTCATGGGATGTGTATAATTATTATCCTACACGACTT

ATCTTTGATATTTCACACAAGTCTTTCAATGTATAACAAAAACTTTTTAGATACATTTAG

ACTATAGAACTAACAAGTTATATTTTGAACC-AAAAAACAAAGAGAGAGAGAGAGGAACA

TAGAGAAAAGAAGATATGAGAGTTTTTTTTTTTTTTTTT--CTAAAACAGAAAGAAACTC

ATTATATAATAAACAAATTACTTTGAGACAAATTAACGTATGTAATAAAACAAAATCAAA

GTGGTAATTAAAATTATTTAATGGTAAACAGTCTAATAGTTAAAATAAAAATGGAAATCA

CATAAATTTTGTAATTGGCCCATTAAAAACAACACTAAGCTTTTAATTTGATTTTGAAAT

TCAAAATAATTTTATTAAATCACAAAGTAAAAGGTTTACAAAGCCGATCATGACAGTGCA

TGTGGGAGGCAAATCGGCATATTTGCACTACAAAAGG-ACCCATGAAGTCTCTGAACATG

CACGCAACACTTTAATCTCTTATTAGTTACTTTGAAAGCTTATTTATATATATAGACACG

CGTAAAAACTTCTTAACCAAGATTTTTTT-ACGTGCTTCC-TTCGCGTTTAATTTGGACC

ATCAAACCGTGCTCAACAGATAAAGAAAAGGGTGCTTTTGATTCAAGATATTGGCC-GAA

AAACACAAGATAGATCCTT-CGATAGATTAAGCCACGCATGAAACGCGAATCCAAAGTGA

TGAAGAAGTGCAGATAGATATTCGTTCACCATATAGAGGAGAATATATCATTCCTACAAA

CAAAATTGATGATGTTTCTAGAATAGAAACGTTAGAATCGAATATTCATCTGTTCCGGGT

GGGGAAGGGCTACTGAAAAGCTGAACTTTTTTAAGAGTTCGAACTTCGAAAGTCAATCAA

TGATTCTTAATTGAGAGGGAAAAGCTATAAAGGACGAGAAGGGAAGGAAATGTTTATAGG

GAATGAATTTCTAATGCATGATGGGAATGACAGATATGAGGAATATATAAAGGTGATTCT

TATATACATTGACATGGATCGGAATCTATTAACTTAAAGTTATTGGGGTCGTGAAATTTA

TTTAATTTTTCTAATTCGTGTCACATGTCACGACAATTAGTGGGG-AACATTTATGTATA

TAGAAATTTCAGAAATTTCTAGCAGCGTGATAAATATAAAGATTTTGGCACAATAAGTTT

TTGGATCATAATGAGTTT-CTATTTAATCAA-GGCA-TGTAA-TCTATTTTATTATTTAG

-TAGGG-AAACTGAAAACTTAGGGTTGCTATTTGTAGCTCCCACCCTTCTTAGTTCTTAC

TCTTTTCAATATTTTTTAAAAGTTTTA-TATAATACCT-AAATT-GCCCTCCTCCT----

----------------------------------------CACCCCATTGCTCTTCCGTT

GCTCCTCCATGTG-AGTTCTTGTCTTTTTTTTT-CTCTGGTAGCGTTTTGCTGCTCCTTT

TTTCACTCAAGTGTTGCCAATTAA-TTGACAAAAAATGGTTTCTGTTTCATATAGAAACT

ATGTTTTTGTTGTGTAGTCATACATTACGGAATCTAGTTT-CCATTAAATAAGTAAC--G

TGAAAAAAAA--TAAAAGGTGAAATATATATTGTTGGAAAAGAAGCTATGAGGTGCAAGA

ACCGATCACATGGAGAAGGCAATGAAAGACAAGGAGGAGCAATGGAAGA-----------

---------GAGAAAATGAGAAGATGGAAGGGATGTGAAAATGTTTGAAAAAAACGAGGT

GATCAGTTTTAAAATACGAATTTAGTATTTTCTTTTTAAGAAAATTCTTTCG-AAAGTCG

TGTTTTAAAACATGACTTTTATT-ATTTGAAGTCG---TGTTCTAAAACATGACTTA--T

TCATATCCTT-AATATTTTT---------AAAATTTATCCATTTGTAATATTTTTTAAAA

ATTGACCCATATATGTAAAATACCCGTCAAGATCTCTTTATTATTTTGAAAGCGAAAGCA

TATCACTTCAAACACAATGGAATCGAGGCTATTGACTAAGTATAAATAGAGAAGACTTCA

TATCGGGGTTCATAATTCATAACAAAGCAAACGAGTATATAAGAAAGCATAAGCCAAATT

TTGAGTAAACTAGTGTGCACACTATCCCATGCCTAGTGGAAGTAGGGATCCTCTCGTTGT

TGGGGGAGTAATTGGGGATGTATTGGATCCTTTTGAATATTCTATTCCTATGAGGGTTAC

CTACAATAACAGAGATGTCAGCAATGGATGTGAATTCAAACCCTCACAAGTTGTCAACCA

ACCAAGGGTAAATATCGGTGGTGATGACC

>CS22.03

TATTAGACTCCTAATTTAATATTCTTGTTTTATTATAATGCTGATAAGTCTTGTAAATAA

GGGTGAGAAGCACGAATAATTAGTTCATGAGATGTGTATAATTATTATCCTACACGACTT

ATCTTTGATATTTCACACAAGTCTTTCAATGTATAACAAAAACTTTTTAGATACATTTAG

ACTATAGAACTAACAAGTTATATTTTGAACC-AAAAAACAAAGAGAGAGAGAGAGGAACA

TAGAGAAAAGAAGATATGAGAGTTTTTTTTTTTTTTTT---CTAAAACAGAAAGAAACTC

ATTATATAATAAACAAATTACTTTGAGACAAATTAACGTATGTAATAAAACAAAATCAAA

GTGGTAATTAAAATTATTTAATGGTAAACAGTCTAATAGTTAAAATAAAAATGGAAATCA

CATAAATTTTGTAATTGGCCCATTAAAAACAACACTAAGCTTTTAATTTGATTTTGAAAT

TCAAAATAATTTTATTAAATCACAAAGTAAAAGGTTTACAAAGCCGATCATGACAGTGCA

TGTGGGAGGCAAATCGGCATATTTGCACTACAAAAGG-ACCCATGAAGTCTCTGAACATG

CACGCAACACTTTAATCTCTTATTAGTTACTTTGAAAGCTTATTTATATATATAGACACG

CGTAAAAACTTCTTAACCAAGATTTTTTT-ACGTGCTTCC-TTCGCGTTTAATTTGGACC

ATCAAACCGTGCTCAACAGATAAAGAAAAGGGTGCTTTTGATTCAAGATATTGGCC-GAA

AAACACAAGATAGATCCTT-CGATAGATTAAGCCACGCATGAAACGCGAATCCAAAGTGA

TGAAGAAGTGCAGATAGATATTCGTTCACCATATAGAGGAGAATATATCATTCCTACAAA

CAAAATTGATGATGTTTCTAGAATAGAAACGTTAGAATCGAATATTCATCTGTTCCGGGT

GGGGAAGGGCTACTGAAAAGCTGAACTTTTTTAAGAGTTCGAACTTCGAAAGTCAATCAA

TGATTCTTAATTGAGAGGGAAAAGCTATAAAGGACGAGAAGGGAAGGAAATGTTTATAGG

GAATGAATTTCTAATGCATGATGGGAATGACAGATATGAGGAATATATAAAGGTGATTCT

TATATACATTGACATGGATCGGAATCTATTAACTTAAAGTTATTGGGGTCGTGAAATTTA

TTTAATTTTTCTAATTCGTGTCACATGTCACGACAATTAGTGGGG-AACATTTATGTATA

TAGAAATTTCAGAAATTTCTAGCAGCGTGATAAATATAAAGATTTTGGCACAATAAGTTT

TTGGATCATAATGAGTTT-CTATTTAATCAA-GGCA-TGTAA-TCTATTTTATTATTTAG

-TAGGG-AAACTGAAAACTTAGGGTTGCTATTTGTAGCTCCCACCCTTCTTAGTTCTTAC

TCTTTTCAATATTTTTTAAAAGTTTTA-TATAATACCT-AAATT-GCCCTCCTCCT----

----------------------------------------CACCCCATTGCTCTTCCGTT

GCTCCTCCATGTG-AGTTCTTGTCTTTTTTTTT-CTCTGGTAGCGTTTTGCTGCTCCTTT

TTTCACTCAAGTGTTGCCAATTAA-TTGACAAAAAATGGTTTCTGTTTCATATAGAAACT

ATGTTTTTGTTGTGTAGTCATACATTACGGAATCTAGTTT-CCATTAAATAAGTAAC--G

TGAAAAAAAA--TAAAAGGTGAAATATATATTGTTGGAAAAGAAGCTATGAGGTGCAAGA

ACCGATCACATGGAGAAGGCAATGAAAGACAAGGAGGAGCAATGGAAGA-----------

---------GAGAAAATGAGAAGATGGAAGGGATGTGAAAATGTTTGAAAAAAACGAGGT

GATCAGTTTTAAAATACGAATTTAGTATTTTCTTTTTAAGAAAATTCTTTCG-AAAGTCG

TGTTTTAAAACATGACTTTTATT-ATTTGAAGTCG---TGTTCTAAAACATGACTTA--T

TCATATCCTT-AATATTTTT---------AAAATTTATCCATTTGTAATATTTTTTAAAA

ATTGACCCATATATGTAAAATACCCGTCAAGATCTCTTTATTATTTTGAAAGCGAAAGCA

TATCACTTCAAACACAATGGAATCGAGGCTATTGACTAAGTATAAATAGAGAAGACTTCA

TATCGGGGTTCATAATTCATAACAAAGCAAACGAGTATATAAGAAAGCATAAGCCAAATT

TTGAGTAAACTAGTGTGCACACTATCCCATGCCTAGTGGAAGTAGGGATCCTCTCGTTGT

TGGGGGAGTAATTGGGGATGTATTGGATCCTTTTGAATATTCTATTCCTATGAGGGTTAC

CTACAATAACAGAGATGTCAGCAATGGATGTGAATTCAAACCCTCACAAGTTGTCAACCA

ACCAAGGGTAAATATCGGTGGTGATGACC

>CS52.H1

TATTAGACTCCTAATTTAATATTCTCGTTTTATTATAATGCTGATAAGTCTTGTAAATAA

GGGTGAGAAGCACGAATAATTAGTTCATGAGATGTGTATAATTATTATCCTACACGACTT

ATCTTTGATATTTCACACAAGTCTTTCAATGTATAACAAAAACTTTTTAGATACATTTAG

ACTATAGAACTAACAAGTTATATTTTGAACC-AAAAAACAAAGAGAGAGAGAGAGGAACA

TAGAGAAAAGAAGATATGAGAGTTTTTTTTTTTTTTTTTTTCTAAAACAGAAAGAAACTC

ATTATATAATAAACAAATTACTTTGAGACAAATTAACGTATGTAATAAAACAAAATCAAA

GTGGTAATTAAAATTATTTAATGGTAAACAGTCTAATAGTTAAAATAAAAATGGAAATCA

CATAAATTTTGTAATTGGCCCATTAAAAACAACACTAAGCTTTTAATTTGATTTTGAAAT

TCAAAATAATTTTATTAAATCACAAAGTAAAAGGTTTACAAAGCCGATCATGACAGTGCA

TGTGGGAGGCAAATCGGCATATTTGCACTACAAAAGG-ACCCATGAAGTCTCTGAACATG

CACGCAACACTTTAATCTCTTATTAGTTACTTTGAAAGCTTATTTATATATATAGACACG

CGTAAAAACTTCTTAACCAAGATTTTTTT-ACGTGCTTCC-TTCGCGTTTAATTTGGACC

ATCAGACCGTGCTCAACAGATAAAGAAAAGGGTGCTTTTGATTCAAGATATTGGCC-GAA

AAACACAAGATAGATCCTT-CGATAGATTAAGCCACGCATGAAACGCGAATCCAAAGTGA

TGAAGAAGTGCAGATAGATATTCGTTCACCATATAGAGGAGAATATATCATTCCTACAAA

CAAAATTGATGATGTTTCTAGAATAGAAACGTTAGAATCGAATATTCATCTGTTCCGGGT

GGGGAAGGGCTACTGAAAAGCTGAACTTTTTTAAGAGTTCGAACTTCGAAAGTCAATCAA

TGATTCTTAATTGAGAGGGAAAAGCTATAAAGGACGAGAAGGGAAGGAAATGTTTATAGG

GAATGAATTTCTAATGCATGATGGGAATGACAGATATGAGGAATATATAAAGGTGATTCT

TATATACATTGACATGGATCGGAATCTATTAACTTAAAGTTATTGGGGTCGTGAAATTTA

TTTAATTTTTCTAATTCGTGTCACATGTCACGACAATTAGTGGGG-AACATTTATGTATA

TAGAAATTTCAGAAATTTCTAGCAGCGTGATAAATATAAAGATTTTGGCACAATAAGTTT

TTGGATCATAATGAGTTT-CTATTTAATCAA-GGCA-TGTAA-TCTATTTTATTATTTAG

-TAGGG-AAACTGAAAACTTAGGGTTGCTATTTGTAGCTCCCACCCTTCTTAGTTCTTAC

TCTTTTCAATATTTTTTAAAAGTTTTA-TATAATACCT-AAATT-GCCCTCCTCCT----

----------------------------------------CACCCCATTGCTCTTCCGTT

GCTCCTCCATGTG-AGTTCTTGTCTTTTTTTT--CTCTGGTAGCGTTTTGCTGCTCCTTT

TTTCACTCAAGTGTTGCCAATTAA-TTGACAAAAAATGGTTTCTGTTTCATATAGAAACT

ATGTTTTTGTTGTGTAGTCATACATTACGGAATCTAGTTT-CCATTAAATAAGTAAC--G

TGAAAAAAA---TAAAAGGTGAAATATATATTGTTGGAAAAGAAGCTATGAGGTGCAAGA

ACCGATCACATGGAGAAGGCAATGAAAGACAAGGAGGAGCAATGGAAGA-----------

---------GAGAAAATGAGAAGATGGAAGGGATGTGAAAATGTTTGAAAAAACCGAGGT

GATCAGTTTTAAAATACGAATTTAGTATTTTCTTTTTAAGAAAATTCTTTCG-AAAGTCG

TGTTTTAAAACATGACTTTTATT-ATTTGAAGTCG---TGTTCTAAAACATGACTTA--T

TCATATCCTT-AATATTTTT---------AAAATTTATCCATTTGTAATATTTTTTAAAA

ATTGACCCATATATGTAAAATACCCGTCAAGATCTCTTTATTATTTTGAAAGCGAAAGCA

TATCACTTCAAACACAATGGAATCGAGGCTATTGACTAAGTATAAATAGAGAAGACTTCA

TATCGGGGTTCATAATTCATAACAAAGCAAACGAGTATATAAGAAAGCATAAGCCAAATT

TTGAGTAAACTAGTGTGCACACTATCCCATGCCTAGTGGAAGTAGGGATCCTCTCGTTGT

TGGGGGAGTAATTGGGGATGTATTGGATCCTTTTGAATATTCTATTCCTATGAGGGTTAC

CTACAATAACAGAGATGTCAGCAATGGATGTGAATTCAAACCCTCACAAGTTGTCAACCA

ACCAAGGGTAAATATCGGTGGTGATGACC

>CS63.M4

TATTAGACTCCTAATTTAATATTCTTGTTTTATTATAATGCTGATAAGTCTTGTAAATAA

GGGTGAGAAGCACGAATAATTAGTTCATGAGATGTGTATAATTATTATCCTACACGACTT

ATCTTTGATATTTCACACAAGTCTTTCAATGTATAACAAAAACTTTTTAGATACATTTAG

ACTATAGAACTAACAAGTTATATTTTGAACC-AAAAAACAAAGAGAGAGAGAGAGGAACA

TAGAGAAAAGAAGATATGAGAGTTTTTTTTTTTTTTTTT--CTAAAACAGAAAGAAACTC

ATTATATAATAAACAAATTACTTTGAGACAAATTAACGTATGTAATAAAACAAAATCAAA

GTGGTAATTAAAATTATTTAATGGTAAACAGTCTAATAGTTAAAATAAAAATGGAAATCA

CATAAATTTTGTAATTGGCCCATTAAAAACAACACTAAGCTTTTAATTTGATTTTGAAAT

TCAAAATAATTTTATTAAATCACAAAGTAAAAGGTTTACAAAGCCGATCATGACAGTGCA

TGTGGGAGGCAAATCGGCATATTTGCACTACAAAAGG-ACCCATGAAGTCTCTGAACATG

CACGCAACACTTTAATCTCTTATTAGTTACTTTGAAAGCTTATTTATATATATAGACACG

CGTAAAAACTTCTTAACCAAGATTTTTTT-ACGTGCTTCC-TTCGCGTTTAATTTGGACC

ATCAAACCGTGCTCAACAGATAAAGAAAAGGGTGCTTTTGATTCAAGATATTGGCC-GAA

AAACACAAGATAGATCCTT-CGATAGATTAAGCCACGCATGAAACGCGAATCCAAAGTGA

TGAAGAAGTGCAGATAGATATTCGTTCACCATATAGAGGAGAATATATCATTCCTACAAA

CAAAATTGATGATGTTTCTAGAATAGAAACGTTAGAATCGAATATTCATCTGTTCCGGGT

GGGGAAGGGCTACTGAAAAGCTGAACTTTTTTAAGAGTTCGAACTTCGAAAGTCAATCAA

TGATTCTTAATTGAGAGGGAAAAGCTATAAAGGACGAGAAGGGAAGGAAATGTTTATAGG

GAATGAATTTCTAATGCATGATGGGAATGACAGATATGAGGAATATATAAAGGTGATTCT

TATATACATTGACATGGATCGGAATCTATTAACTTAAAGTTATTGGGGTCGTGAAATTTA

TTTAATTTTTCTAATTCGTGTCACATGTCACGACAATTAGTGGGG-AACATTTATGTATA

TAGAAATTTCAGAAATTTCTAGCAGCGTGATAAATATAAAGATTTTAGCACAATAAGTTT

TTGGATCATAATGAGTTT-CTATTTAATCAA-GGCA-TGTAA-TCTATTTTATCATTTAG

-TAGGG-AAACTGAAAACTTAGGGTTGCTATTTGTAGCTCCCACCCTTCTTAGTTCTTAC

TCTTTTCAATATTTTTTAAAAGTTTTA-TATAATACCT-AAATT-GCCCTCCTCCT----

----------------------------------------CACCCCATTGCTCTTCCGTT

GCTC-TCCATGTG-AGTTCTTGTCTTTTTTTTT-CTCTGGTAGCGTTTTGCTGCTCCTTT

TTTCACTCAAGTGTTGCCAATTAA-TTGACAAAAAATGGTTTCTGTTTCATATAGAAACT

ATGTTTTTGTTGTGTAGTCATACATTACGGAATCTAGTTT-CCATTAAATAAGTAAC--G

TGAAAAAAAA--TAAAAGGTGAAATATATATTGTTGGAAAAGAAGCTATGAGGTGCAAGA

ACCGATCACATGGAGAAGGCAATGAAAGACAAGGAGGAGCAATGGAAGA-----------

---------GAGAAAATGAGAAGATGGAAGGGATGTGAAAATGTTTGAAAAAAACGAGGT

GATCAGTTTTAAAATACGAATTTAGTATTTTCTTTTTAAGAAAATTCTTTCG-AAAGTCG

TGTTTTAAAACATGACTTTTATT-ATTTGAAGTCG---TGTTCTAAAACATGACTTA--T

TCATATCCTT-AATATTTTT---------AAAATTTATCCATTTGTAATATTTTTTAAAA

ATTGACCCATATATGTAAAATACCCGTCAAGATCTCTTTATTATTTTGAAAGCGAAAGCA

TATCACTTCAAACACAATGGAATCGAGGCTATTGACTAAGTATAAATAGAGAAGACTTCA

TATCGGGGTTCATAATTCATAACAAAGCAAACGAGTATATAAGAAAGCATAAGCCAAATT

TTGAGTAAACTAGTGTGCACACTATCCCATGCCTAGTGGAAGTAGGGATCCTCTCGTTGT

TGGGGGAGTAATTGGGGATGTATTGGATCCTTTTGAATATTCTATTCCTATGAGGGTTAC

CTACAATAACAGAGATGTCAGCAATGGATGTGAATTCAAACCCTCACAAGTTGTCAACCA

ACCAAGGGTAAATATCGGTGGTGATGACC

>CS02.24

TATTAGACTCCTAATTTAATATTCTTGTTTTATTATAATGCTGATAAGTCTTGTAAATAA

GGGTGAGAAGCACGAATAATTAGTTCATGAGATGTGTATAATTATTATCCTACACGACTT

ATCTTTGATATTTCACACAAGTCTTTCAATGTATAACAAAAACTTTTTAGATACATTTAG

ACTATAGAACTAACAAGTTATATTTTGAACC-AAAAAACAAAGAGAGAGAGAGAGGAACA

TAGAGAAAAGAAGATATGAGAGTTTTTTTTTTTTTTT----CTAAAACAGAAAGAAACTC

ATTATATAATAAACAAATTACTTTGAGACAAATTAACGTATGTAATAAAACAAAATCAAA

GTGGTAATTAAAATTATTTAATGGTAAACAGTCTAATAGTTAAAATAAAAATGGAAATCA

CATAAATTTTGTAATTGGCCCATTAAAAACAACACTAAGCTTTTAATTTGATTTTGAAAT

TCAAAATAATTTTATTAAATCACAAAGTAAAAGGTTTACAAAGCCGATCATGACAGTGCA

TGTGGGAGGCAAATCGGCATATTTGCACTACAAAAGG-ACCCATGAAGTCTCTGAACATG

CACGCAACACTTTAATCTCTTATTAGTTACTTTGAAAGCTTATTTATATATATAGACACG

CGTAAAAACTTCTTAACCAAGATTTTTTT-ACGTGCTTCC-TTCGCGTTTAATTTGGACC

ATCAAACCGTGCTCAACAGATAAAGAAAAGGGTGCTTTTGATTCAAGATATTGGCC-GAA

AAACACAAGATAGATCCTT-CGATAGATTAAGCCACGCATGAAACGCGAATCCAAAGTGA

TGAAGAAGTGCAGATAGATATTCGTTCACCATATAGAGGAGAATATATCATTCCTACAAA

CAAAATTGATGATGTTTCTAGAATAGAAACGTTAGAATCGAATATTCATCTGTTCCGGGT

GGGGAAGGGCTACTGAAAAGCTGAACTTTTTTAAGAGTTCGAACTTCGAAAGTCAATCAA

TGATTCTTAATTGAGAGGGAAAAGCTATAAAGGACGAGAAGGGAAGGAAATGTTTATAGG

GAATGAATTTCTAATGCATGATGGGAATGACAGATATGAGGAATATATAAAGGTGATTCT

TATATACATTGACATGGATCGGAATCTATTAACTTAAAGTTATTGGGGTCGTGAAATTTA

TTTAATTTTTCTAATTCGTGTCACATGTCACGACAATTAGTGGGG-AACATTTATGTATA

TAGAAATTTCAGAAATTTCTAGCAGCGTGATAAATATAAAGATTTTGGCACAATAAGTTT

TTGGATCATAATGAGTTT-CTATTTAATCAA-GGCA-TGTAA-TCTATTTTATTATTTAG

-TAGGG-AAACTGAAAACTTAGGGTTGCTATTTGTAGCTCCCACCCTTCTTAGTTCTTAC

TCTTTTCAATATTTTTTAAAAGTTTTA-TATAATACCT-AAATT-GCCCTCCTCCT----

----------------------------------------CACCCCATTGCTCTTCCGTT

GCTCCTCCATGTG-AGTTCTTGTCTTTTTTTTT-CTCTGGTAGCGTTTTGCTGCTCCTTT

TTTCACTCAAGTGTTGCCAATTAA-TTGACAAAAAATGGTTTCTGTTTCATATAGAAACT

ATGTTTTTGTTGTGTAGTCATACATTACGGAATCTAGTTT-CCATTAAATAAGTAAC--G

TGAAAAAAAA--TAAAAGGTGAAATATATATTGTTGGAAAAGAAGCTATGAGGTGCAAGA

ACCGATCACATGGAGAAGGCAATGAAAGACGAGGAGGAGCAATGGAAGA-----------

---------GAGAAAATGAGAAGATGGAAGGGATGTGAAAATGTTTGAAAAAAACGAGGT

GATCAGTTTTAAAATACGAATTTAGTATTTTCTTTTTAAGAAAATTCTTTCG-AGAGTCG

TGTTTTAAAACATGACCTTTATT-ATTTGAAGTCG---TGTTCTAAAACATGACTTA--T

TCATATCCTT-AATATTTTT---------AAAATTTATCCATTTGTAATATTTTTTAAAA

ATTGACCCATATATGTAAAATACCCGTCAAGATCTCTTTATTATTTTGAAAGCGAAAGCA

TATCACTTCAAACACAATGGAATCGAGGCTATTGACTAAGTATAAATAGAGAAGACTTCA

TATCGGGGTTCATAATTCATAACAAAGCAAACGAGTATATAAGAAAGCATAAGCCAAATT

TTGAGTAAACTAGTGTGCACACTATCCCATGCCTAGTGGAAGTAGGGATCCTCTCGTTGT

TGGGGGAGTAATTGGGGATGTATTGGATCCTTTTGAATATTCTATTCCTATGAGGGTTAC

CTACAATAACAGAGATGTCAGCAATGGATGTGAATTCAAACCCTCACAAGTTGTCAACCA

ACCAAGGGTAAATATCGGTGGTGATGACC

>CS33.32

TATTAGACTCCTAATTTAATATTCTTGTTTTATTATAATGCTGATAAGTCTTGTAAATAA

GGGTGAGAAGCACGAATAATTAGTTCATGAGATGTGTATAATTATTATCCTACACGACTT

ATCTTTGATATTTCACACAAGTCTTTCAATGTATAACAAAAACTTTTTAGATACATTTAG

ACTATAGAACTAACAAGTTATATTTTGAACC-AAAAAACAAAGAGAGAGAGAGAGGAACA

TAGAGAAAAGAAGATATGAGAGTTTTTTTTTTTTTTTT---CCAAAACAGAAAGAAACTC

ATTATATAATAAACAAATTACTTTGAGACAAATTAACGTATGTAATAAAACAAAATCAAA

GTGGTAATTAAAATTATTTAATGGTAAACAGTCTAATAGTTAAAATAAAAATGGAAATCA

CATAAATTTTGTAATTGGCCCATTAAAAACAACACTAAGCTTTTAATTTGATTTTGAAAT

TCAAAATAATTTTATTAAATCACAAAGTAAAAGGTTTACAAAGCCGATCATGACAGTGCA

TGTGGGAGGCAAATCGGCATATTTGCACTACAAAAGG-ACCCATGAAGTCTCTGAACATG

CACGCAACACTTTAATCTCTTATTAGTTACTTTGAAAGCTTATTTATATATATAGACACG

CGTAAAAACTTCTTAGCCAAGATTTTTTT-ACGTGCTTCC-TTCGCGTTTAATTTGGACC

ATCAAACCGTGCTCAACAGATAAAGAAAAGGGTGCTTTTGATTCAAGATATTGGCC-GAA

AAACACAAGATAGATCCTT-CGATAGATTAAGCCACGCATGAAACGCGAATCCAAAGTGA

TGAAGAAGTGCAGATAGATATTCGTTCACCATATAGAGGAGAATATATCATTCCTACAAA

CAAAATTGATGATGTTTCTAGAATAGAAACGTTAGAATCGAATATTCATCTGTTCCGGGT

GGGGAAGGGCTACTGAAAAGCTGAACTTTTTTAAGAGTTCGAACTTCGAAAGTCAATTAA

TGATTCTTAATTGAGAGGGAAAAGCTATAAAGGACGAGAAGGGAAGGAAATGTTTATAGG

GAATGAATTTCTAATGCATGATGGGAATGACAGATATGAGGAATATATAAAGGTGATTCT

TATATACATTGACATGGATCGGAATCTATTAACTTAAAGTTATTGGGGTCGTGAAATTTA

TTTAATTTTTCTAATTCGTGTCACATGTCACGACAATTAGTGGGG-AACATTTATGTATA

TAGAAATTTCAGAAATTTCTAGCAGCGTGATAAATATAAAGATTTTGGCACAATAAGTTT

TTGGATCATAATGAGTTT-CTATTTAATCAA-GGCA-TGTAA-TCTATTTTATTATTTAG

-TAGGG-AAACTGAAAACTTAGGGTTGCTATTTGTAGCTCCCACCCTTCTTAGTTCTTAC

TCTTTTCAATATTTTTTAAAAGTTTTA-TATAATACCT-AAATT-GCCCTCCTCCT----

----------------------------------------CACCCCATTGCTCTTCCGTT

GCTCCTCCATGTG-AGTTCTTGTCTTTTTTTTT-CTCTGGTAGCGTTTTGCTGCTCCTTT

TTTCACTCAAGTGTTGCCAATTAA-TTGACAAAAAATGGTTTCTGTTTCATATAGAAACT

ATGTTTTTGTTGTGTAGTCATACATTACGGAATCTAGTTT-CCATTAAATAAGTAAC--G

TGAAAAAAAA--TAAAAGGTGAAATATATATTGTTGGAAAAGAAGCTATGAGGTGCAAGA

ACCGATCACATGGAGAAGGCAATGAAAGACAAGGAGGAGCAATGGAAGA-----------

---------GAGAAAATGAGAAGATGGAAGGGATGTGAAAATGTTTGAAAAAAACGAGGT

GATCAGTTTTAAAATACGAATTTAGTATTTTCTTTTTAAGAAAATTCTTTCG-AAGGTCG

TGTTTTAAAACATGACTTTTATT-ATTTGAAGTCG---TGTTCTAAAACATGACTTA--T

TCATATCCTT-AATATTTTT---------AAAATTTATCCATTTGTAATATTTTTTAAAA

ATTGACCCATATATGTAAAATACCCGTCAAGATCTCTTTATTATTTTGAAAGCGAAAGCA

TATCACTTCAAACACAATGGAATCGAGGCTATTGACTAAGTATAAATAGAGAAGACTTCA

TATCGGGGTTCATAATTCATAACAAAGCAAACGAGTATATAAGAAAGCATAAGCCAAATT

TTGAGTAAACTAGTGTGCACACTATCCCATGCCTAGTGGAAGTAGGGATCCTCTCGTTGT

TGGGGGAGTAATTGGGGATGTATTGGATCCTTTTGAATATTCTATTCCTATGAGGGTTAC

CTACAATAACAGAGATGTCAGCAATGGATGTGAATTCAAACCCTCACAAGTTGTCAACCA

ACCAAGGGTAAATATCGGTGGTGATGACC

>CS52.H8

TATTAGACTCCTAATTTAATATTCTTGTTTTATTATAATGCTGATAAGTCTTGTAAATAA

GGGTGAGAAGCACGAATAATTAGTTCATGAGATGTGTATAATTATTATCCTACACGACTT

ATCTTTGATATTTCACACAAGTCTTTCAATGTATAACAAAAACTTTTTAGATACATTTAG

ACTATAGAACTAACAAGTTATATTTTGAACC-AAAAAACAAAGAGAGAGAGAGAGGAACA

TAGGGAAAAGAAGATATGAGAGTTTTTTTTTTTTTTTT---CTAAAACAGAAAGAAACTC

ATTATATAATAAACAAATTACTTTGAGACAAATTAACGTATGTAATAAAACAAAATCAAA

GTGGTAATTAAAATTATTTAATGGTAAACAGTCTAATAGTTAAAATAAAAATGGAAATCA

CATAAATTTTGTAATTGGCCCATTAAAAACAACACTAAGCTTTTAATTTGATTTTGAAAT

TCAAAATAATTTTATTAAATCACAAAGTAAAAGGTTTACAAAGCCGATCATGACAGTGCA

TGTGGGAGGCAAATCGGCATATTTGCACTACAAAAGG-ACCCATGAAGTCTCTGAACATG

CACGCAACACTTTAATCTCTTATTAGTTACTTTGAAAGCTTATTTATATATATAGACACG

CGTAAAAACTTCTTAACCAAGATTTTTTT-ACGTGCTTCC-TTCGCGTTTAATTTGGACC

ATCAAACCGTGCTCAACAGATAAAGAAAAGGGTGCTTTTGATTCAAGATATTGGCC-GAA

AAACACAAGATAGATCCTT-CGATAGATTAAGCCACGCATGAAACGCGAATCCAAAGTGA

TGAAGAAGTGCAGATAGATATTCGTTCACCATATAGAGGAGAATATATCATTCCTACAAA

CAAAATTGATGATGTTTCTAGAATAGAAACGTTAGAATCGAATATTCATCTGTTCCGGGT

GGGGAAGGGCTACTGAAAAGCTGAACTTTTTTAAGAGTTCGAACTTCGAAAGTCAATCAA

TGATTCTTAATTGAGAGGGAAAAGCTATAAAGGACGAGAAGGGAAGGAAATGTTTATAGG

GAATGAATTTCTAATGCATGATGGGAATGACAGATATGAGGAATATATAAAGGTGATTCT

TATATACATTGACATGGATCGGAATCTATTAACTTAAAGTTATTGGGGTCGTGAAATTTA

TTTAATTTTTCTAATTCGTGTCACATGTCACGACAATTAGTGGGG-AACATTTATGTATA

TAGAAATTTCAGAAATTTCTAGCAGCGTGATAAATATAAAGATTTTGGCACAATAAGTTT

TTGGATCATAATGAGTTT-CTATTTAATCAA-GGCA-TGTAA-TCTATTTTATTATTTAG

-TAGGG-AAACTGAAAACTTAGGGTTGCTATTTGTAGCTCCCACCCTTCTTAGTTCTTAC

TCTTTTCAATATTTTTTAAAAGTTTTA-TATAATACCT-AAATT-GCCCTCCTCCT----

----------------------------------------CACCCCATTGCTCTTCCGTT

GCTCCTCCATGTG-AGTTCTTGTCTTTTTTTTT-CTCTGGTAGCGTTTTGCTGCTCCTTT

TTTCACTCAAGTGTTGCCAATTAA-TTGACAAAAAATGGTTTCTGTTTCATATAGAAACT

ATGTTTTTGTTGTGTAGTCATACATTACGGAATCTAGTTT-CCATTAAATAAGTAAC--G

TGAAAAAAAA--TAAAAGGTGAAATATATATTGTTGGAAAAGAAGCTATGAGGTGCAAGA

ACCGATCACATGGAGAAGGCAATGAAAGACAAGGAGGAGCAATGGAAGA-----------

---------GAGAAAATGAGAAGATGGAAGGGATGTGAAAATGTTTGAAAAAAACGAGGT

GATCAGTTTTAAAATACGAATTTAGTATTTTCTTTTTAAGAAAATTCTTTCG-AAAGTCG

TGTTTTAAAACATGACTTTTATT-ATTTGAAGTCG---TGTTCTAAAACATGACTTA--T

TCATATCCTT-AATATTTTT---------AAAATTTATCCATTTGTAATATTTTTTAAAA

ATTGACCCATATATGTAAAATACCCGTCAAGATCTCTTTATTATTTTGAAAGCGAAAGCA

TATCACTTCAAACACAATGGAATCGAGGCTATTGACTAAGTATAAATAGAGAAGACTTCA

TATCGGGGTTCATAATTCATAACAAAGCAAACGAGTATATAAGAAAGCATAAGCCAAATT

TTGAGTAAACTAGTGTGCACACTATCCCATGCCTAGTGGAAGTAGGGATCCTCTCGTTGT

TGGGGGAGTAATTGGGGATGTATTGGATCCTTTTGAATATTCTATTCCTATGAGGGTTAC

CTACAATAACAGAGATGTCAGCAATGGATGTGAATTCAAACCCTCACAAGTTGTCAACCA

ACCAAGGGTAAATATCGGTGGTGATGACC

>CS37.63

TATTAGACTCCTAATTTAATATTCTTGTTTTATTATAATGCTGATAAGTCTTGTAAATAA

GGGTGAGAAGCACGAATAATTAGTTCATGAGATGTGTATAATTATTATCCTACACGACTT

ATCTTTGATATTTCACACAAGTCTTTCAATGTATAACAAAAACTTTTTAGATACATTTAG

ACTATAGAACTAACAAGTTATATTTTGAACC-AAAAAACAAAGAGAGAGAGAGAGGAACA

TAGAGAAAAGAAGATATGAGAGTTTTTTTTTTTTTTTT---CTAAAACAGAAAGAAACTC

ATTATATAATAAACAAATTACTTTGAGACAAATTAACGTATGTAATAAAACAAAATCAAA

GTGGTAATTAAAATTATTTAATGGTAAACAGTCTAATAGTTAAAATAAAAATGGAAATCA

CATAAATTTTGTAATTGGCCCATTAAAAACAACACTAAGCTTTTAATTCGATTTTGAAAT

TCAAAATAATTTTATTAAATCACAAAGTAAAAGGTTTACAAAGCCGATCATGACAGTGCA

TGTGGGAGGCAAATCGGCATATTTGCACTACAAAAGG-ACCCATGAAGTCTCTGAACATG

CACGCAACACTTTAATCTCTTATTAGTTACTTTGAAAGCTTATTTATATATATAGACACG

CGTAAAAACTTCTTAACCAAGATTTTTTT-ACGTGCTTCC-TTCGCGTTTAATTTGGACC

ATCAAACCGTGCTCAACAGATAAAGAAAAGGGTGCTTTTGATTCAAGATATTGGCC-GAA

AAACACAAGATAGATCCTT-CGATAGATTAAGCCACGCATGAAACGCGAATCCAAAGTGA

TGAAGAAGTGCAGATAGATATTCGTTCACCACATAGAGGAGAATATATCATTCCTACAAA

CAAAATTGATGATGTTTCTAGAATAGAAACGTTAGAATCGAATATTCATCTGTTCCGGGT

GGGGAAGGGCTACTGAAAAGCTGAACTTTTTTAAGAGTTCGAACTTCGAAAGTCAATCAA

TGATTCTTAATTGAGAGGGAAAAGCTATAAAGGACGAGAAGGGAAGGAAATGTTTATAGG

GAATGAATTTCTAATGCATGATGGGAATGACAGATATGAGGAATATATAAAGGTGATTCT

TATATACATTGACATGGATCGGAATCTATTAACTTAAAGTTATTGGGGTCGTGAAATTTA

TTTAATTTTTCTAATTCGTGTCACATGTCACGACAATTAGTGGGG-AACATTTATGTATA

TAGAAATTTCAGAAATTTCTAGCAGCGTGATAAATATAAAGATTTTGGCACAATAAGTTT

TTGGATCATAATGAGTTT-CTATTTAATCAA-GGCA-TGTAA-TCTATTTTATTATTTAG

-TAGGG-AAACTGAAAACTTAGGGTTGCTATTTGTAGCTCCCACCCTTCTTAGTTCTTAC

TCTTTTCAATATTTTTTAAAAGTTTTA-TATAATACCT-AAATT-GCCCTCCTCCT----

----------------------------------------CACCCCATTGCTCTTCCGTT

GCTCCTCCATGTG-AGTTCTTGTCTTTTTTTTT-CTCTGGTAGCGTTTTGCTGCTCCTTT

TTTCACTCAAGTGTTGCCAATTAA-TTGACAAAAAATGGTTTCTGTTTCATATAGAAACT

ATGTTTTTGTTGTGTAGTCATACATTACGGAATCTAGTTT-CCATTAAATAAGTAAC--G

TGAAAAAAAA--TAAAAGGTGAAATATATATTGTTGGAAAAGAAGCTATGAGGTGCAAGA

ACCGATCACATGGAGAAGGCAATGAAAGACAAGGAGGAGCAATGGAAGA-----------

---------GAGAAAATGAGAAGATGGAAGGGATGTGAAAATGTTTGAAAAAAACGAGGT

GATCAGTTTTAAAATACGAATTTAGTATTTTCTTTTTAAGAAAATTCTTTCG-AAAGTCG

TGTTTTAAAACATGACTTTTATT-ATTTGAAGTCG---TGTTCTAAAACATGACTTA--T

TCATATCCTT-AATATTTTT---------AAAATTTATCCATTTGTAATATTTTTTAAAA

ATTGACCCATATATGTAAAATACCCGTCAAGATCTCTTTATTATTTTGAAAGCGAAAGCA

TATCACTTCAAACACAATGGAATCGAGGCTATTGACTAAGTATAAATAGAGAAGACTTCA

TATCGGGGTTCATAATTCATAACAAAGCAAACGAGTATATAAGAAAGCATAAGCCAAATT

TTGAGTAAACTAGTGTGCACACTATCCCATGCCTAGTGGAAGTAGGGATCCTCTCGTTGT

TGGGGGAGTAATTGGGGATGTATTGGATCCTTTTGAATATTCTATTCCTATGAGGGTTAC

CTACAATAACAGAGATGTCAGCAATGGATGTGAATTCAAACCCTCACAAGTTGTCAACCA

ACCAAGGGTAAATATCGGTGGTGATGACC

>CS40.71

TATTAGACTCCTAATTTAATATTCTTGTTTTATTATAATGCTGATAAGTCTTGTAAATAA

GGGTGAGAAGCACGAATAATTAGTTCATGAGATGTGTATAATTATTATCCTACACGACTT

ATCTTTGATATTTCACACAAGTCTTTCAATGTATAACAAAAACTTTTTAGATACATTTAG

ACTATAGAACTAACAAGTTATATTTTGAACC-AAAAAACAAAGAGAGAGAGAGAGGAACA

TAGAGAAAAGAAGATATGAGAGTTTTTTTTTTTTTTT----CTAAAACAGAAAGAAACTC

ATTATATAATAAACAAATTACTTTGAGACAAATTAACGTATGTAATAAAACAAAATCAAA

GTGGTAATTAAAATTATTTAATGGTAAACAGTCTAATAGTTAAAATAAAAATGGAAATCA

CATAAATTTTGTAATTGGCCCATTAAAAACAACACTAAGCTTTTAATTTGATTTTGAAAT

TCAAAATAATTTTATTAAATCACAAAGTAAAAGGTTTACAAAGCCGATCATGACAGTGCA

TGTGGGAGGCAAATCGGCATATTTGCACTACAAAAGG-ACCCATGAAGTCTCTGAACATG

CACGCAACACTTTAATCTCTTATTAGTTACTTTGAAAGCTTATTTATATATATAGACACG

CGTAAAAACTTCTTAACCAAGATTTTTTT-ACGTGCTTCC-TTCGCGTTTAATTTGGACC

ATCAAACCGTGCTCAACAGATAAAGAAAAGGGTGCTTTTGATTCAAGATATTGGCC-GAA

AAACACAAGATAGATCCTT-CGATAGATTAAGCCACGCATGAAACGCGAATCCAAAGTGA

TGAAGAAGTGCAGATAGATATTCGTTCACCATATAGAGGAGAATATATCATTCCTACAAA

CAAAATTGATGATGTTTCTAGAATAGAAACGTTAGAATCGAATATTCATCTGTTCCGGGT

GGGGAAGGGCTACTGAAAAGCTGAACTTTTTTAAGAGTTCGAACTTCGAAAGTCAATCAA

TGATTCTTAATTGAGAGGGAAAAGCTATAAAGGACGAGAAGGGAAGGAAATGTTTATAGG

GAATGAATTTCTAATGCATGATGGGAATGACAGATATGAGGAATATATAAAGGTGATTCT

TATATACATTGACATGGATCGGAATCTATTAACTTAAAGTTATTGGGGTCGTGAAATTTA

TTTAATTTTTCTAATTCGTGTCACATGTCACGACAATTAGTGGGG-AACATTTATGTATA

TAGAAATTTCAGAAATTTCTAGCAGCGTGATAAATATAAAGATTTTGGCACAATAAGTTT

TTGGATCGTAATGAGTTT-CTATTTAATCAA-GGCA-TGTAA-TCTATTTTATTATTTAG

-TAGGG-AAACCGAAAACTTAGGGTTGCTATTTGTAGCTCCCACCCTTCTTAGTTCTTAC

TCTTTTCAATATTTTTTAAAAGTTTTA-TATAATACCT-AAATT-GCCCTCCTCCT----

----------------------------------------CACCCCATTGCTCTCCCGTT

GCTCCTCCATGTG-AGTTCTTGTCTTTTTTTTT-CTCTGGTAGCGTTTTGCTGCTCCTTT

TTTCACTCAAGTGTTGCCAATTAA-TTGACAAAAAATGGTTTCTGTTTCATATAGAAACT

ATGTTTTTGTTGTGTAGTCATACATTACGGAATCTAGTTT-CCATTAAATAAGTAAC--G

TGAAAAAAAA--TAAAAGGTGAAATATATATTGTTGGAAAAGAAGCTATGAGGTGCAAGA

ACCGATCACATGGAGGAGGCAATGAAAGACAAGGAGGAGCAATGGGAGA-----------

---------GAGAAAATGAGAAGATGGAAGGGATGTGAAAATGTTTGAAAAAAACGAGGT

GATCAGTTTTAAAATACGAATTTAGTATTTTCTTTTTAAGAAAATTCTTTCG-AAAGTCG

TGTTTTAAAACATGACTTTTATT-ATTTGAAGTCG---TGTTCTAAAACATGACTTA--T

TCATATCCTT-AATATTTTT---------AAAATTTATCCATTTGTAATATTTTTTAAAA

ATTGACCCATATATGTAAAATACCCGTCAAGATCTCTTTATTATTTTGAAAGCGAAAGCA

TATCACTTCAAACACAATGGAATCGAGGCTATTGACTAAGTATAAATAGAGAAGACTTCA

TATCGGGGTTCATAATTCATAACAAAGCAAACGAGTATATAAGAAAGCATAAGCCAAATT

TTGAGTAAACTAGTGTGCACACTATCCCATGCCTAGTGGAAGTAGGGATCCTCTCGTTGT

TGGGGGAGTAATTGGGGATGTATTGGATCCTTTTGAATATTCTATTCCTATGAGGGTTAC

CTACAATAACAGAGATGTCAGCAATGGATGTGAATTCAAACCCTCACAAGTTGTCAACCA

ACCAAGGGTAAATATCGGTGGTGATGACC

>CS49.E1

TATTAGACTCCTAATTTAATATTCTTGTTTTATTATAATGCTGATAAGTCTTGTAAATAA

GGGTGAGAAGCACGAATAATTAGTTCATGAGATGAGTATAATTATTATCCTACACGACTT

ATCTTTGATATTTCACACAAGTCTTTCAATGTATAACAAAAACTTTTTAGATACATTTAG

ACTATAGAACTAACAAGTTATATTTTGAACC-AAAAAACAAAGAGAGAGAGAGAGGAACA

TAGAGAAAAGAAGATATGAGAGTTTTTTCTTTTTTTTT---CTAAAACAGAAAGAAACTC

ATTATATAATAAACAAATTACTTTGAGACAAATTAACGTATGTAATAAAACAAAATCAAA

GTGGTAATTAAAATTATTTAATGGTAAACAGTCTAATAGTTAAAATAAAAATGGAAATCA

CATAAATTTTGTAATTGGCCCATTAAAAACAACACTAAGCTTTTAATTTGATTTTGAAAT

TCAAAATAATTTTATTAAATCACAAAGTAAAAGGTTTACAAAGCCGATCATGACAGTGCA

TGTGGGAGGCAAATCGGCATATTTGCACTACAAAAGG-ACCCATGAAGTCTCTGAACATG

CACGCAACACTTTAATCTCTTATTAGTTACTTTGAAAGCTTATTTATATATATAGACACG

CGTAAAAACTTCTTAACCAAGATTTTTTT-ACGTGCTTCC-TTCGCGTTTAATTTGGACC

ATCAGACCGTGCTCAACAGATAAAGAAAAGGGTGCTTTTGATTCAAGATATTGGCC-GAA

AAACACAAGATAGATCCTT-CGATAGATTAAGCCACGCATGAAACGCGAATCCAAAGTGA

TGAAGAAGTGCAGATAGATATTCGTTCACCATATAGAGGAGAATATATCATTCCTACAAA

CAAAATTGATGATGTTTCTAGAATAGAAACGTTAGAATCGAATATTCATCTGTTCCGGGT

GGGGAAGGGCTACTGAAAAGCTGAACTTTTTTAAGAGTTCGAACTTCGAAAGTCAACCAA

CGATTCTTAATTGAGAGGGAAAAGCTATAAAGGACGAGAAGGGAAGGAAATGTTTATAGG

GAATGAATTTCTAATGCATGATGGGAATGACAGATATGAGGAATATATAAAGGTGATTCT

TATATACATTGACATGGATCGGAATCTATTAACTTAAAGTTATTGGGGTCGTGAAATTTA

TTTAATTTTTCTAATTCGTGTCACATGTCACGACAATTAGTGGGG-AACATTTATGTATA

TAGAAATTTCAGAAATTTCTAGCAGCGTGATAAATATAAAGATTTTGGCACAATAAGTTT

TTGGATCATAATGAGTTT-CTATTTAATCAA-GGCA-TGTAA-TCTATTTTATTATTTAG

-TAGGG-AAACTGAAAACTTAGGGTTGCTATTTGTAGCTCCCACCCTTCTTAGTTCTTAC

TCTTTTCAATATTTTTTAAAAGTTTTA-TATAATACCT-AAATT-GCCCTCCTCCT----

----------------------------------------CACCCCATTGCTCTCCCGTT

GCTCCTCCATGTG-AGTTCTTGTCTTTTTTTTT-CTCTGGTAGCGTTTTGCTGCTCCTTT

TTTCACTCAAGTGTTGCCAATTAA-TTGACAAAAAATGGTTTCTGTTTCATATAGAAACT

ATGTTTTTGTTGTGTAGTCATACATTACGGAATCTAGTTT-CCATTAAATAAGTAAC--G

TGAAAAAAAA--TAAAAGGTGAAATATATATTGTTGGGAAAGAAGCTATGAGGTGCAAGA

ACCGATCACATGGAGAAGGCAATGAAAGACAAGGAGGAGCAATGGAAGA-----------

---------GAGAAAATGAGAAGATGGAAGGGATGTGAAAATGTTTGAAAAAAACGAGGT

GATCAGTCTTAAAATACGAATTTAGTATTTTCTTTTTAAGAAAATTCTTTCG-AAAGTCG

TGTTTTAAAACATGACTTTTATT-ATTTGAAGTCG---TGTTCTAAAACATGACTTA--T

TCATATCCTT-AATATTTTT---------AAAATTTATCCATTTGTAATATTTTTTAAAA

ATTGACCCATATATGTAAAATACCCGTCAAGATCTCTTTATTATTTTGAAAGCGAAAGCA

TATCACTTCAAACACAATGGAATCGAGGCTATTGACTAAGTATAAATAGAGAAGACTTCA

TATCGGGGTTCATAATTCATAACAAAGCAAACGAGTATATAAGAAAGCATAAGCCAAATT

TTGAGTAAACTAGTGTGCACACTATCCCATGCCTAGTGGAAGTAGGGATCCTCTCGTTGT

TGGGGGAGTAATTGGGGATGTATTGGATCCTTTTGAATATTCTATTCCTATGAGGGTTAC

CTACAATAACAGAAATGTCAGCAATGGATGTGAATTCAAACCCTCACAAGTTGTCAACCA

ACCAAGGGTAAATATCGGTGGTGATGACC

>CS53.J2

TATTAGACTCCTAATTTAATATTCTTGTTTTATTATAATGCTGATAAGTCTTGTAAATAA

GGGTGAGAAGCACGAATAATTAGTTCATGAGATGTGTATAATTATTATCCTACACGACTT

ATCTTTGATATTTCACACAAGTCTTTCAATGTATAACAAAAACTTTTTAGATACATTTAG

ACTATAGAACTAACAAGTTATATTTTGAACC-AAAAAACAAAGAGAGAGAGAGAGGAACA

TAGAGAAAAGAAGATATGAGAGTTTTTTTTTTTCT------CTAAAACAGAAAGAAACTC

ATTATATAATAAACAAATTACTTTGAGACAAATTAACGTATGTAATAAAACAAAATCAAA

GTGGTAATTAAAATTATTTAATGGTAAACAGTCTAATAGTTAAAATAAAAATGGAAATCA

CATAAATTTTGTAATTGGCCCATTAAAAACAACACTAAGCTTTTAATTTGATTTTGAAAT

TCAAAATAATTTTATTAAATCACAAAGTAAAAGGTTTACAAAGCCGATCATGACAGTGCA

TGTGGGAGGCAAATCGGCATATTTGCACTACAAAAGG-ACCCATGAAGTCTCTGAACATG

CACGCAACACTTTAATCTCTTATTAGTTACTTTGAAAGCTTATTTATATATATAGACACG

CGTAAAAACTTCTTAACCAAGATTTTTTT-ACGTGCTTCC-TTCGCGTTTAATTTGGACC

ATCAAACCGTGCTCAACAGATAAAGAAAAGGGTGCTTTTGATTCAAGATATTGGCC-GAA

AAACACGAGATAGATCCTT-CGATAGATTAAGCCACGCATGAAACGCGAATCCAAAGTGA

TGAAGAAGTGCAGATAGATATTCGTTCACCATATAGAGGAGAATATATCATTCCTACAAA

CAAAATTGATGATGTTTCTAGAATAGAAACGTTAGAATCGAATATTCATCTGTTCCGGGT

GGGGAAGGGCTACTGAAAAGCTGAACTTTTTTAAGAGTTCGAACTTCGAAAGTCAATCAA

TGATTCTTAATTGAGAGGGAAAAGCTATAAAGGACGAGAAGGGAAGGAAATGTTTATAGG

GAATGAATTTCTAATGCATGATGGGAATGACAGATATGAGGAATATATAAAGGTGATTCT

TATATACATTGACATGGATCGGAATCTATTAACTTAAAGTTATTGGGGTCGTGAAATTTA

TTTAATTTTTCTAATTCGTGTCACATGTCACGACAATTAGTGGGG-AACATTTATGTATA

TAGAAATTTCAGAAATTTCTAGCAGCGTGATAAATATAAAGATTTTGGCACAATAAGTTT

TTGGATCATAATGAGTTT-CTATTTAATCAA-GGCA-TGTAA-TCTATTTTATTATTTAG

-TAGGG-AAACTGAAAACTTAGGGTTGCTATTTGTAGCTCCCACCCTTCTTAGTTCTTAC

TCTTTTCAATATTTTTTAAAAGTTTTA-TATAATACCT-AAATT-GCCCTCCTCCT----

----------------------------------------CACCCCATTGCTCTTCCGTT

GCTCCTCCATGTG-AGTTCTTGTCTTTTTTTTT-CTCTGGTAGCGTTTTGCTGCTTCTTT

TTTCACTCAAGTGTTGCCAATTAA-TTGACAAAAAATGGTTTCTGTTTCATATAGAAACT

ATGTTTTTGTTGTGTAGTCATACATTACGGAATCTAGTTT-CCATTAAATAAGTAAC--G

TGAAAAAAAA--TAAAAGGTGAAATATATATTGTTGGAAAAGAAGCTATGAGGTGCAAGA

ACCGATCACATGGAGAAGGCAATGAAAGACAAGGAGGAGCAATGGAAGA-----------

---------GAGAAAATGAGAAGATGGAAGGGATGTGAAAATGTTTGAAAAAAACGAGGT

GATCAGTTTTAAAATACGAATTTAGTATTTTCTTTTTAAGAAAATTCTTTCG-AAAGTCG

TGTTTTAAAACATGACTTTTATT-ATTTGAAGTCG---TGTTCTAAAACATGACTTA--T

TCATATCCTT-AATATTTTT---------AAAATTTATCCATTTGTAATATTTTTTAAAA

ATTGACCCATACATGTAAAATACCCGTCAAGATCTCTTTATTATTTTGAAAGCGAAAGCA

TATCACTTCAAACACAATGGAGTCGAGGCTATTGACTAAGTATAAATAGAGAAGACTTCA

TATCGGGGTTCATAATTCATAACAAAGCAAACGAGTATATAAGAAAGCATAAGCCAAATT

TTGAGTAAACTAGTGTGCACACTATCCCATGCCTAGTGGAAGTAGGGATCCTCTCGTTGT

TGGGGGAGTAATTGGGGATGTATTGGATCCTTTTGAATATTCTATTCCTATGAGGGTTAC

CTACAATAACAGAGATGTCAGCAATGGATGTGAATTCAGACCCTCACAAGTTGTCAACCA

ACCAAGGGTAAATATCGGTGGTGATGACC

>CS20.48

TATTAGACTCCTAATTTAATATTCTTGTTTTATTATAATGCTGATAAGTCTTGTAAATAA

GGGTGAGAAGCACGAATAATTAGTTCATGAGATGTGTATAATTATTATCCTACACGACTT

ATCTTTGATATTTCACACAAGTCTTTCAATGTATAACAAAAACTTTTTAGATACATTTAG

ACTATAGAACTAACAAGTTATATTTTGAACC-AAAAAACAAAGAGAGAGAGAGAGGAACA

TAGAGAAAAGAAGATATGAGAGTTTTTTTTTTTTTTT----CTAAAACAGAAAGAAACTC

ATTATATAATAAACAAATTACTTTGAGACAAATTAACGTATGTAATAAAACAAAATCAAA

GTGGTAATTAAAATTATTTAATGGTAAACAGTCTAATAGTTAAAATAAAAATGGAAATCA

CATAAATCTTGTAATTGGCCCATTAAAAACAACACTAAGCTTTTAATTTGATTTTGAAAT

TCAAAATAATTTTATTAAATCACAAAGTAAAAGGTTTACAAAGCCGATCATGACAGTGCA

TGTGGGAGGCAAATCGGCATATTTGCACTACAAAAGG-ACCCATGAAGTCTCTGAACATG

CACGCAACACTTTAATCTCTTATTAGTTACTTTGAAAGCTTATTTATATATATAGACACG

CGTAAAAACTTCTTAACCAAGATTTTTTT-ACGTGCTTCC-TTCGCGTTTAATTTGGACC

ATCAAACCGTGCTCAACAGATAAAGAAAAGGGTGCTTTTGATTCAAGATATTGGCC-GAA

AAACACAAGATAGATCCTT-CGATAGATTAAGCCACGCATGAAACGCGAATCCAAAGTGA

TGAAGAAGTGCAGATAGATATTCGTTCACCATATAGAGGAGAATATATCATTCCTACAAA

CAAAATTGATGATGTTTCTAGAATAGAAACGTTAGAATCGAATATTCATCTGTTCCGGGT

GGGGAAGGGCTACTGAAAAGCTGAACTTTTTTAAGAGTTCGAACTTCGAGAGTCAATCAA

TGATTCTTAATTGAGAGGGAAAAGCTATAAAGGACGAGAAGGGAAGGAAATGTTTATAGG

GAATGAATTTCTAATGCATGATGGGAATGACAGATATGAGGAATATATAAAGGTGATTCT

TATATACATTGACATGGATCGGAATCTATTAACTTAAAGTTATTGGGGTCGTGAAATTTA

TTTAATTTTTCTAATTCGTGTCACATGTCACGACAATTAGTGGGG-AACATTTATGTATA

TAGAAATTTCAGAAATTTCTAGCAGCGTGATAAATATAAAGATTTTGGCACAATAAGTTT

TTGGATCATAATGAGTTT-CTATTTAATCAA-GGCA-TGTAA-TCTATTTTATTATTTAG

-TAGGG-AAACTGAAAACTTAGGGTTGCTATTTGTAGCTCCCACCCTTCTTAGTTCTTAC

TCTTTTCAATATTTTTTAAAAGTTTTA-TATAATACCT-AAATT-GCCCTCCTCCT----

----------------------------------------CACCCCATTGCTCTTCCGTT

GCTCCTCCATGTG-AGTTCTTGTCTTTTTTTTT-CTCTGGTAGCGTTTTGCTGCTCCTTT

TTTCACTCAAGTGTTGCCAATTAA-TTGACAAAAAATGGTTTCTGTTTCATATAGAAACT

ATGTTTTTGTTGTGTAGTCATACATTACGGAATCTAGTTT-CCATTAAATAAGTAAC--G

TGAAAAAAAA--TAAAAGGTGAAATATATATTGTTGGAAAAGAAGCTATGAGGTGCAAGA

ACCGATCACATGGAGAAGGCAATGAAAGACAAGGAGGAGCAATGGAAGA-----------

---------GAGAAAATGAGAAGATGGAAGGGATGTGAAAATGTTTGAAAAAAACGAGGT

GATCAGTTTTAAAATACGAATTTAGTATTTTCTTTTTAAGAAAATTCTTTCG-AAAGTCG

TGTTTTAAAACATGACTTTTATT-ATTTGAAGTCG---TGTTCTAAAACATGACTTA--T

TCATATCCTT-AATATTTTT---------AAAATTTATCCATTTGTAATATTTTTTAAAA

ATTGACCCATATATGTAAAATACCCGTCAAGATCTCTTTATTATTTTGAAAGCGAAAGCA

TATCACTTCAAACACAATGGAATCGAGGCTATTGACTAAGTATAAATAGAGAAGACTTCA

TATCGGGGTTCATAATTCATAACAAAGCAAACGAGTATATAAGAAAGCATAAGCCAAATT

TTGAGTAAACTAGTGTGCACACTATCCCATGCCTAGTGGAAGTAGGGATCCTCTCGTTGT

TGGGGGAGTAATTGGGGATGTATTGGATCCTTTTGAATATTCTATTCCTATGAGGGTTAC

CTACAATAACAGAGATGTCAGCAATGGATGTGAATTCAAACCCTCACAAGTTGTCAACCA

GCCAAGGGTAAATATCGGTGGTGATGACC

>H01.02

TATTAGACTCCTAATTTAATATTCTTGTTTTATTATAATGCTGATAAGTCTTGTAAATAA

GGGTGAGAAGCACGAATAATTAGTTCATGAGATGTGTATAATTATTATCCTACACGACTT

ATCTTTGATATTTCACACAAGTCTTTCAATGTATAACAAAAACTTTTTAGATACATTTAG

ACTATAGAACTAACAAGTTATATTTTGAACC-AAAAAACAAAGAGAGAGAGAGAGGAACA

TAGAGAAAAGAAGATATGAGAGTTTTTTTTTTTTTTTT---CTAAAACAGAAAGAAACTC

ATTATATAATAAACAAATTACTTTGAGACAAATTAACGTATGTAATAAAACAAAATCAAA

GTGGTAATTAAAATTATTTAATGGTAAACAGTCTAATAGTTAAAATAAAAATGGAAATCA

CATAAATTTTGTAATTGGCCCACTAAAAACAACACTGAGCTTTTAATTTGATTTTGAAAT

TCAAAATAATTTTATTAAATCACAAAGTAAAAGGTTTACAAAGCCGATCATGACAGTGCA

TGTGGGAGGCAAATCGGCATATTTGCACTACAAAAGG-ACCCATGAAGTCTCTGAACATG

CACGCAACACTTTAATCTCTTATTAGTTACTTTGAAAGCTTATTTATATATATAGACACG

CGTAAAAACTTCTTAACCAAGATTTTTTT-GCGTGCTTCC-TTCGCGTTTAATTTGGACC

ATCAAACCGTGCTCAACAGATAAAGAAAAGGGTGCTTTTGATTCAAGATATTGGCC-GAA

AAACACAAGATAGATCCTT-CGATAGATTAAGCCACGCATGAAACGCGAATCCAAAGTGA

TGAAGAAGTGCGGATAGATATTCGTTCACCATATAGAGGAGAATATATCATTCCTACAAA

CAAAATTGATGATGTTTCTAGAATAGAAACGTTAGAATCGAATATTCATCTGTTCCGGGT

GGGGAAGGGCTACTGAAAAGCTGAACTTTTTTAAGAGTTCGAACTTCGAAAGTCAATCAA

TGATTCTTAATTGAGAGGGAAAAGCTATAAAGGACGAGAAGGGAAGGAAATGTTTATAGG

GAATGAATTTCTAATGCATGATGGGAATGACAGATATGAGGAATATATAAAGGTGATTCT

TATATACATTGACATGGATCGGAATCTATTAACTTAAAGTTATTGGGGTCGTGAAATTTA

TTTAATTTTTCTAATTCGTGTCACATGTCACGACAATTAGTGGGG-AACATTTATGTATA

TAGAAATTTCAGAAATTTCTAGCAGCGTGATAAATATAAAGATTTTGGCACAATAAGTTT

TTGGATCATAATGAGTTT-CTATTTAATCAA-GGCA-TGTAA-TCTATTTTATTATTTAG

-TAGGG-AAACTGAAAACTTAGGGTTGCTATTTGTAGCTCCCACCCTTCTTAGTTCTTAC

TCTTTTCAATATTTTTTAAAAGTTTTA-TATAATACCT-AAATT-GCCCTCCTCCT----

----------------------------------------CACCCCATTGCTCTTCCGTT

GCTCCTCCATGTG-AGTTCTTGTCTTTTTTTTT-CTCTGGTAGCGTTTTGCAGCTCCTTT

TTTCACTCAAGTGTTGCCAATTAA-TTGACAAAAAATGGTTTCTGTTTCATATAGAAACT

ATGTTTTTGTTGTGTAGTCATACATTACGGAATCTAGTTT-CCATTAAATAAGTAACAT-

-GAAAAAAAAA-TAAAAGGTGAAATATATATTGTTGGAAAAGAAGCTATGAGGTGCAAGA

ACCGATCACATGGAGAAGGCAATGAAAGACAAGGAGGAGCAATGGAAGA-----------

---------GAGAAAATGAGAAGATGGAAGGGATGTGAAAATGTTTGAAAAAAACGAGGT

GATCAGTTTTAAAATACGAATTTAGTATTTTCTTTTTAAGAAAATTCTTTCG-AAAGTCG

TGTTTTAAAACATGACTTTTATT-ATTTGAAGTCG---TGTTCTAAAACATGACTTA--T

TCATATCCTT-AATATTTTT---------AAATTTTATCCATTTGTAATATTTTTTAAAA

ATTGACCCATATATGTAAAATACCCGTCAAGATCTCTTTATTATTTTGAAAGCGAAAGCA

TATCACTTCAAACACAATGGAATCGAGGCTATTGACTAAGTATAAATAGAGAAGACTTCA

TATCGGGGTTCATAATTCATAACAAAGCAAACGAGTATATAAGAAAGCATAAGCCAAATT

TTGAGTACACTAGTGTGCACACTATCCCATGCCTAGTGGAAGTAGGGATCCTCTCGTTGT

TGGGGGAGTAATTGGGGATGTATTGGATCCTTTTGAATATTCTATTCCTATGAGGGTTAC

CTACAATAACAGAGATGTCAGCAATGGATGTGAATTCAAACCCTCACAAGTTGTCAACCA

ACCAAGGGTAAATATCGGTGGTGATGACC

>CS25.14

TATTAGACTCCTAATTTAATATTCTTGTTTTATTATAATGCTGATAAGTCTTGTAAATAA

GGGTGAGAAGCACGAATAATTAGTTCATGAGATGTGTATAATTATTATCCTACACGACTT

ATCTTTGATATTTCACACAAGTCTTTCAATGTATAACAAAAACTTTTTAGATGCATTTAG

ACTATAGAACTAACAAGTTATATTTTGAACC-AAAAAACAAAGAGAGAGAGAGAGGAACA

TAGAGAAAAGAAGATATGAGAGTTTTTTTTTTTTTTTTT--CTAAAACAGAAAGAAACTC

ATTATATAATAAACAAATTACTTTGAGACAAATTAACGTATGTAATAAAACAAAATCAAA

GTGGTAATTAAAATTATTTAATGGTAAACAGTCTAATAGTTAAAATAAAAATGGAAATCA

CATAAATTTTGTAATTGGCCCATTAAAAACAACACTAAGCTTTTAATTTGATTTTGAAAT

TCAAAATAATTTTATTAAATCACAAAGTAAAAGGTTTACAAAGCCGATCATGACAGTGCA

TGTGGGAGGCAAATCGGCATATTTGCACTACAAAAGG-ACCCATGAAGTCTCTGAACATG

CACGCAACACTTTAATCTCTTATTAGTTACTTTGAAAGCTTATTTATATATATAGACACG

CGTAAAAACTTCTTAACCAAGATTTTTTT-ACGTGCTTCC-TTCGCGTTTAATTTGGACC

ATCAAACCGTGCTCAACAGATAAAGAAAAGGGTGCTTTTGATTCAAGATATTGGCC-GAA

AAACACAAGATAGATCCTT-CGATAGATTAAGCCACGCATGAAACGCGAATCCAAAGTGA

TGAAGAAGTGCAGATAGATATTCGTTCACCATATAGAGGAGAATATATCATTCCTACAAA

CAAAATTGATGATGTTTCTAGAATAGAAACGTTAGAATCGAATATTCATCTGTTCCGGGT

GGGGAAGGGCTACTGAAAAGCTGAACTTTTTTAAGAGTTCGAACTTCGAAAGTCAATCAA

TGATTCTTAATTGAGAGGGAAGAGCTATAAAGGACGAGAAGGGAAGGAAATGTTTATAGG

GAATGAATTTCTAATGCATGATGGGAATGACAGATATGAGGAATATATAAAGGTGATTCT

TATATACATTGACATGGATCGGAATCTATTAACTTAAAGTTATTGGGGTCGTGAAATTTA

TTTAATTTTTCTAATTCGTGTCACATGTCACGACAATTAGTGGGG-AACATTTATGTATA

TAGAAATTTCAGAAATTTCTAGCAGCGTGATAAATATAAAGATTTTGGCACAATAAGTTT

TTGGATCATAATGAGTTT-CTATTTAATCAA-GGCA-TGTAA-TCTATTTTATTATTTAG

-TAGGG-AAACTGAAAACTTAGGGTTGCTATTTGTAGCTCCCACCCTTCTTAGTTCTTAC

TCTTTTCAATATTTTTTAAAAGTTTTA-TATAATACCT-AAATT-GCCCTCCTCCT----

----------------------------------------CACCCCATTGCTCTTCCGTT

GCTCCTCCATGTG-AGTTCT-GTCTTTTTTTTT-CTCTGGTAGCGTTTTGCTGCTCCTTT

TTTCACTCAAGTGTTGCCAATTAA-TTGACAAAAAATGGTTTCTGTTTCATATAGAAACT

ATGTTTTTGTTGTGTAGTCATACATTACGGAATCTAGTTT-CCATTAAATAAGTAAC--G

TGAAAAAAAA--TAAAAGGTGAAATATATATTGTTGGAAAAGAAGCTATGAGGTGCAAGA

ACCGATCACATGGAGAAGGCAATGAAAGACAAGGAGGAGCAATGGAAGA-----------

---------GAGAAAATGAGAAGATGGAAGGGATGTGAAAATGTTTGAAAAAAACGAGGT

GATCAGTTTTAAAATACGAATTTAGTATTTTCTTTTTAAGAAAATTCTTTCG-AAAGTCG

TGTTTTAAAACATGACTTTTATT-ATTTGAAGTCG---TGTTCTAAAACATGACTTA--T

TCATATCCTT-AATATTTTT---------AAAATTTATCCATTTGTAATATTTTTTAAAA

ATTGACCCATATATGTAAAATACCCGTCAAGATCTCTTTATTATTTTGAAAGCGAAAGCA

TATCACTTCAAACACAATGGAATCGAGGCTATTGACTAAGTATAAATAGAGAAGACTTCA

TATCGGGGTTCATAATTCATAACAAAGCAAACGAGTATATAAGAAAGCATAAGCCAAATT

TTGAGTAAACTAGTGTGCACACTATCCCATGCCTAGTGGAAGTAGGGATCCTCTCGTTGT

TGGGGGAGTAATTGGGGATGTATTGGATCCTTTTGAATATTCTATTCCTATGAGGGTTAC

CTACAATAACAGAGATGTCAGCAATGGATGTGAATTCAAACCCTCACAAGTTGTCAACCA

ACCAAGGGTAAATATCGGTGGTGATGACC

>CS38.65

TATTAGACTCCTAATTTAATATTCTTGTTTTATTATAATGCTGATAAGTCTTGTAAATAA

GGGTGAGAAGCACGAATAATTAGTTCATGAGATGTGTATAATTATTATCCTACACGACTT

ATCTTTGATATTTCACACAAGTCTTTCAATGTATAACAAAAACTTTTTAGATACATTTAG

ACTATAGAACTAACAAGTTATATTTTGAACC-AAAAAACAAAGAGAGAGAGAGAGGAACA

TAGAGAAAAGAAGATATGAGAGTTTTTTTTTTTTTTTT---CTAAAACAGAAAGAAACTC

ATTATATAATAAACAAATTACTTTGAGACAAATTAACGTATGTAATAAAACAAAATCAAA

GTGGTAATTAAAATTATTTAATGGTAAACAGTCTAATAGTTAAAATAAAAATGGAAATCA

CATAAATTTTGTAATTGGCCCATTAAAAACAACACTAAGCTTTTAATTTGATTTTGAAAT

TCAAAATAATTTTATTAAATCACAAAGTAAAAGGTTTACAAAGCCGATCATGACAGTGCA

TGTGGGAGGCAAATCGGCATATTTGCACTACAAAAGG-ACCCATGAAGTCTCTGAACATG

CACGCAACACTTTAATCTCTTATTAGTTACTTTGAAAGCTTATTTATATATATAGACACG

CGTAAAAACTTCTTAGCCAAGATTTTTTT-ACGTGCTTCC-TTCGCGTTTAATTTGGACC

ATCAAACCGTGCTCAACAGATAAAGAAAAGGGTGCTTTTGATTCAAGATATTGGCC-GAA

AAACACAAGATAGATCCTT-CGATAGATTAAGCCACGCATGAAACGCGAATCCAAAGTGA

TGAAGAAGTGCAGATAGATATTCGTTCACCATATAGAGGAGAATATATCATTCCTACAAA

CAAAATTGATGATGTTTCTAGAATAGATACGTTAGAATCGAATATTCATCTGTTCCGGGT

GGGGAAGGGCTACTGAAAAGCTGAACTTTTTTAAGAGTTCGAACTTCGAAAGTCAATCAA

TGATTCTTAATTGAGAGGGAAAAGCTATAAAGGACGAGAAGGGAAGGAAATGTTTATAGG

GAATGAATTCCTAATGCATGATGGGAATGACAGATATGAGGAATATATAAAGGTGATTCT

TATATACATTGACATGGATCGGAATCTATTAACTTAAAGTTATTGGGGTCGTGAAATTTA

TTTAATTTTTCTAATTCGTGTCACATGTCACGACAATTAGTGGGG-AACATTTATGTATA

TAGAAATTTCAGAAATTTCTAGCAGCGTGATAAATATAAAGATTTTGGCACAATAAGTTT

TTGGATCATAATGAGTTT-CTATTTAATCAA-GGCA-TGTAA-TCTATTTTATTATTTAG

-TAGGG-AAACTGAAAACTTAGGGTTGCTATTTGTAGCTCCCACCCTTCTTAGTTCTTAC

TCTTTTCAATATTTTTTAAA-GTTTTA-TATAATACCT-AAATT-GCCCTCCTCCT----

----------------------------------------CACCCCATTGCTCTTCCGTT

GCTCCTCCATGTG-AGTTCTTGTCTTTTTTTTT-CTCTGGTAGCGTTTTGCTGCTCCTTT

TTTCACTCAAGTGTTGCCAATTAA-TTGACAAAAAATGGTTTCTGTTTCATATAGAAACT

ATGTTTTTGTTGTGTAGTCATACATTACGGAATCTAGTTT-CCATTAAATAAGTAAC--G

TGAAAAAAAA--TAAAAGGTGAAATATATATTGTTGGAAAAGAAGCTATGAGGTGCAAGA

ACCGATCACATGGAGAAGGCAATGAAAGACAAGGAGGAGCAACGGAAGA-----------

---------GAGAAAATGAGAAGATGGAAGGGATGTGAAAATGTTTGAAAAAAACGAGGT

GGTCAGTTTTAAAATACGAATTTAGTATTTTCTTTTTAAGAAAATTCTTTCG-AAAGTCG

TGTTTTAAAACATGACTTTTATT-ATTTGAAGTCG---TGTTCTAAAACATGACTTA--T

TCATATCCTT-AATATTTTT---------AAAATTTATCCATTTGTAATATTTTTTAAAA

ATTGACCCATATATGTAAAATACCCGTCAAGATCTCTTTATTATTTTGAAAGCGAAAGCA

TATCACTTCAAACACAATGGAATCGAGGCTATTGACTAAGTATAAATAGAGAAGACTTCA

TATCGGGGTTCATAATTCATAACAAAGCAAACGAGTATATAAGAAAGCATAAGCCAAATT

TTGAGTAAACTAGTGTGCACACTATCCCATGCCTAGTGGAAGTAGGGATCCTCTCGTTGT

TGGGGGAGTAATTGGGGATGTATTGGATCCTTTTGAATATTCTATTCCTATGAGGGTTAC

CTACAATAACAGAGATGTCAGCAATGGATGTGAATTCAAACCCTCACAAGTTGTCAACCA

ACCAAGGGTAAATATCGGTGGTGATGACC

>H06.03

TATTAGACTCCTAATTTAATATTCTTGTTTTATCATAATGCTGATAAGTCTTGTAAATAA

GGGTGAGAAGCACGAATAATTAGTTCATGAGATGTGTATAATTATTATCCTACACGACTT

ATCTTTGATATTTCACACAAGTCTTTCAATGTATAACAAAAACTTTTTAGATACATTTAG

ACTATAGAACTAACAAGTTATATTTTGAACC-AAAAAACAAAGAGAGAGAGAGAGGAACA

TAGAGAAAAGAAGATATGAGAGTTTTTTTTTTTTTTT----CTAAAACAGAAAGAAACTC

ATTATATAATAAACAAATTACTTTGAGACAAATTAACGTATGTAATAAAACAAAATCAAA

GTGGTAATTAAAATTATTTAATGGTAAACAGTCTAATAGTTAAAATAAAAATGGAAATCA

CATAAATTTTGTAATTGGCCCATTAAAAACAACACTAAGCTTTTAATTTGATTTTGAAAT

TCAAAATAATTTTATTAAATCACAAAGTAAAAGGTTTACAAAGCCGATCATGACAGTGCA

TGTGGGAGGCAAATCGGCATATTTGCACTACAAAAGG-ACCCATGAAGTCTCTGAACATG

CACGCAACACTTTAATCTCTTATTAGTTACTTTGAAAGCTTATTTATATATATAGACACG

CGTAAAAACTTCTTAACCAAGATTTTTTT-ACGTGCTTCC-TTCGCGTTTAATTTGGACC

ATCAAACCGTGCTCAACAGATAAAGAAAAGGGTGCTTTTGATTCAAGATATTGGCC-GAA

AAACACAAGATAGATCCTT-CGATAGATTAAGCCACGCATGAAACGCGAATCCAAAGTGA

TGAAGAAGTGCAGATAGATATTCGTTCACCATATAGAGGAGAATATATCATTCCTACAAA

CAAAATTGATGATGTTTCTAGAATAGAAACGTTAGAATCGAATATTCATCTGTTCCGGGT

GGGGAAGGGCTACTGAAAAGCTGAACTTTTTTAGGAGTTCGAACTTCGAAAGTCAATCAA

TGATTCTTAATTGAGAGGGAAAAGCTATAAAGGACGAGAAGGGAAGGAAATGTTTATAGG

GAATGAATTTCTAATGCATGATGGGAATGACAGATATGAGGAATATATAAAGGTGATTCT

TATATACATTGACATGGATCGGAATCTATTAACTTAAAGTTATTGGGGTCGTGAAATTTA

TTTAATTTTTCTAATTCGTGTCACATGTCACGACAATTAGTGGGG-AACATTTATGTATA

TAGAAATTTCAGAAATTTCTAGCAGCGTGATAAATATAAAGATTTTGGCACAATAAGTTT

TTGGATCATAATGAGTTT-CTATTTAATCAA-GGCA-TGTAA-TCTATTTTATTATTTAG

-TAGGG-AAACTGAAAACTTAGGGTTGCTATTTGTAGCTCCCACCCTTCTTAGTTCTTAC

TCTTTTCAATATTTTTTAAAAGTTTTA-TATAATACCT-AAATT-GCCCTCCTCCT----

----------------------------------------CACCCCATTGCTCTTCCGTT

GCTCCTCCATGTG-AGTTCTTGTCTTTTTTTTT-CTCTGGTAGCGTTTTGCTGCTCCTTT

TTTCACTCAAGTGTTGCCAATTAA-TTGACAAAAAATGGTTTCTGTTTCATATAGAAACT

ATGTTTTTGTTGTGTAGTCATACATTACGGAATCTAGTTT-CCATTAAATAAGTAAC--G

TGAAAAAAAA--TAAAAGGTGAAATATATATTGTTGGAAAAGAAGCTATGAGGTGCAAGA

ACCGATCACATGGAGAAGGCAATGAAAGACAAGGAGGAGCAATGGAAGA-----------

---------GAGAAAATGAGAAGATGGAAGGGATGTGAAAATGTTTGAAAAAAACGAGGT

GATCAGTTTTAAAATACGAATTTAGTATTTTCTTTTTAAGAAAATTCTTTCG-AAAGTCG

TGTTTTAAAACATGACTTTTATT-ATTTGAAGTCG---TGTTCTAAAACATGACTTA--T

TCATATCCTT-AATATTTTT---------AAAATTTATCTATTTGTAATATTTTTTAAAA

ATTGACCCATATATGTAAAATACCCGTCAAGATCTCTTTATTATTTTGAAAGCGAAAGCA

TATCACTTCAAACACAATGGAATCGAGGCTATTGACTAAGTATAAATAGAGAAGACTTCA

TATCGGGGTTCATAATTCATAACAAAGCAAACGAGTATATAAGAAAGCATAAGCCAAATT

TTGAGTAAACTAGTGTGCACACTATCCCATGCCTAGTGGAAGTAGGGATCCTCTCGTTGT

TGGGGGAGTAATTGGGGATGTATTGGATCCTTTTGAATATTCTATTCCTATGAGGGTTAC

CTACAATAACAGAGATGTCAGCAATGGATGTGAATTCAAACCCTCACAAGTTGTCAACCA

ACCAAGGGTAAATATCGGTGGTGATGACC

>H04.01

TATTAGACTCCTAATTTAATATTCTTGTTTTATTATAATGCTGATAAGTCTTGTAAATAA

GGGTGAGAAGCACGAATAATTAGTTCATGAGATGTGTATAATTATTATCCTACACGACTT

ATCTTTGATATTTCACACAAGTCTTTCAATGTATAACAAAAACTTTTTAGATACATTTAG

ACTATAGAACTAACAAGTTATATTTTGAACC-AAAAAACAAAGAGAGAGAGAGAGGAGCA

TAGAGAAAAGAAGATATGAGAGTTTTTTTTTTTTTTT----CTAAAACAGAAAGAAACTC

ATTATATAATAAACAAATTACTTTGAGACAAATTAACGTATGTAATAAAACAAGATCAAA

GTGGTAATTAAAATTATTTAATGGTAAACAGTCTAATAGTTAAAATAAAAATGGAAATCA

CATAAATTTTGTAATTGGCCCATTAAAAACAACACTAAGCTTTTAATTTGATTTTGAAAT

TCAAAATAATTTTATTAAATCACAAAGTAAAAGGTTTACAAAGCCGATCATGACAGTGCA

TGTGGGAGGCAAATCGGCATATTTGCACTACAAAAGG-ACCCATGAAGTCTCTGAACATG

CACGCAACACTTTAATCTCTTATTAGTTACTTTGAAAGCTTATTTATATATATAGACACG

CGTAAAAACTTCTTAACCAAGATTTTTTT-ACGTGCTTCC-TTCGCGTTTAATTTGGACC

ATCAAACCGTGCTCAACAGATAAAGAAAAGGGTGCTTTTGATTCAAGATATTGGCC-GAA

AAACACAAGATAGATCCTT-CGATAGATTAAGCCACGCATGAAACGCGAATCCAAAGTGA

TGAAGAAGTGCAGATAGATATTCGTTCACCATATAGAGGAGAATATATCATTCCTACAAA

CAAAATTGATGATGTTTCTAGAATAGAAACGTTAGAATCGAATATTCATCTGTTCCGGGT

GGGGAAGGGCTACTGAAAAGCTGAACTTTTTTAAGAGTTCGAACTTCGAAAGTCAATCAA

TGATTCTTAATTGAGAGGGAAAAGCTATAAAGGACGAGAAGGGAAGGGAATGTTTATAGG

GAATGAATTTCTAATGCATGATGGGAATGACAGATATGAGGAATATATAAAGGTGATTCT

TATATACATTGACATGGATCGGAATCTATTAACTTAAAGTTATTGGGGTCGTGAAATTTA

TTTAATTTTTCTAATTCGTGTCACATGTCACGACAATTAGTGGGG-AACATTTACGTATA

TAGAAATTTCAGAAATTTCTAGCAGCGTGATAAATATAAAGATTTTGGCACAATAAGTTT

TTGGATCATAATGAGTTT-CTATTTAATCAA-GGCA-TGTAA-TCTATTTTATTATTTAG

-TAGGG-AAACTGAAAACTTAGGGTTGCTATTTGTAGCTCCCACCCTTCTTAGTTCTTAC

TCTTTTCAATATTTTTTAAA-GTTTTA-TATAATACCT-AAATT-GCCCTCCTCCT----

----------------------------------------CACCCCATTGCTCTTCCGTT

GCTCCTCCATGTG-AGTTCTTGTCTTTTTTTTT-CTCTGGTAGCGTTTTGCTGCTCCTTT

TTTCACTCAAGTGTTACCAATTAA-TTGACAAAAAATGGTTTCTGTTTCATATAGAAACT

ATGTTTTTGTTGTGTAGTCATACATTACGGAATCTAGTTT-CCGTTAAATAAGTAAC--G

TGAAAAAAAA--TAAAAGGTGAAATATATATTGTTGGAAAAGAAGCTATGAGGTGCAAGA

ACCGATCACATGGAGAAGGCAATGAAAGACAAGGAGGAGCAATGGAAGA-----------

---------GAGAAAATGAGAAGATGGAAGGGATGTGAAAATGTTTGAAAAAAACGAGGT

GATCAGTTTTAAAATACGAATTTAGTATTTTCTTTTTAAGAAAATTCTTTCG-AAAGTCG

TGTTTTAAAACATGACTTTTATT-ATTTGAAGTCG---TGTTCTAAAACATGACTTA--T

TCATATCCTT-AATATTTTT---------AAAATTTATCCATTTGTAATATTTTTTAAAA

ATTGACCCATATATGTAAAATACCCGTCAAGATCTCTTTATTATTTTGAAAGCGAAAGCA

TATCACTTCAAACACAATGGAATCGAGGCTATTGACTAAGTATAAATAGAGAAGACTTCA

TATCGGGGTTCATAATTCATAACAAAGCAAACGAGTATATAAGAAAGCATAAGCCAAATT

TTGAGTAAACTAGTGTGCACACTATCCCATGCCTAGTGGAAGTAGGGATCCTCTCGTTGT

TGGGGGAGTAATTGGGGATGTATTGGATCCTTTTGAATATTCTATTCCTATGAGGGTTAC

CTACAATAACAGAGATGTCAGCAATGGATGTGAATTCAAACCCTCACAAGTCGTCAACCA

ACCAAGGGTAAATATCGGTGGTGATGACC

>H04.06c

TATTAGACTCCTAATTTAATATTCTTGTTTTATTATAATGCTGATAAGTCTTGTAAATAA

GGGTGAGAAGCACGAATAATTAGTTCATGAGATGTGTATAATTATTATCCTACACGACTT

ATCTTTGATATTTCACACAAGTCTTTCAATGTATAACAAAAACTTTTTAGATACATTTAG

ACTATAGAACTAACAAGTTATATTTTGAACC-AAAAAACAAAGAGAGAGAGAGAGGAACA

TAGAGAAAAGAAGATATGAGAGTTTTTTTTTTTTTTTT---CTAAAACAGAAAGAAACTC

ATTATATAATAAACAAATTACTTTGAGACAAATTAACGTATGTAATAAAACAAGATCAAA

GTGGTAATTAAAATTATTTAATGGTAAACAGTCTAATAGTTAAAATAAAAATGGAAATCA

CATAAATTTTGTAATTGGCCCATTAAAAACAACACTAAGCTTTTAATTTGATTTTGAAAT

TCAAAATAATTTTATTAAATCACAAAGTAAAAGGTTTACAAAGCCGATCATGACAGTGCA

TGTGGGAGGCAAATCGGCATATTTGCACTACAAAAGG-ACCCATGAAGTCTCTGAACATG

CACGCAACACTTTAATCTCTTATTAGTTACTTTGAAAGCTTATTTATATATATAGACACG

CGTAAAAACTTCTTAACCAAGATTTTTTT-ACGTGCTTCC-TTCGCGTTTAATTTGGACC

ATCAAACCGTGCTCAACAGATAAAGAAAAGGGTGCTTTTGATTCAAGATATTGGCC-GAA

AAACACAAGATAGATCCTT-CGATAGATTAAGCCACGCATGAAACGCGAATCCAAAGTGA

TGAAGAAGTGCAGATAGATATTCGTTCACCATATAGAGGAGGATATATCATTCCTACAAA

CAAAATTGATGATGTTTCTAGAATAGAAACGTTAGAATCGAATATTCATCTGTTTCGGGT

GGGGAAGGGCTACTGAAAAGCTGAACTTTTTTAAGAGTTCGAACTTCGAAAGTCAATCAA

TGATTCTTAATTGAGAGGGAAAAGCTATAAAGGACGAGAAGGGAAGGAAATGTTTATAGG

GAATGAATTTCTAATGCATGATGGGAATGACAGATATGAGGAATATATAAAGGTGATTCT

TATATACATTGACATGGATCGGAATCTATTAACTTAAAGTTATTGGGGTCGTGAAATTTA

TTTAATTTTTCTAATTCGTGTCACATGTCACGACAATTAGTGGGG-AACATTTATGTATA

TAGAAATTTCAGAAATTTCTAGCAGCGTGATAAATATAAAGATTTTGGCACAATAAGTTT

TTGGATCATAATGAGTTT-CTATTTAATCAA-GGCA-TGTAA-TCTATTTTATTATTTAG

-TAGGG-AAACTGAAAACTTAGGGTTGCTATTTGTAGCTCCCACCCTTCTTAGTTCTTAC

TCTTTTCAATATTTTTTAAAAGTTTTA-TATAATACCT-AAATT-GCCCTCCTCCT----

----------------------------------------CACCCCATTGCTCTTCCGTT

GCTCCTCCATGTG-AGTTCTTGTCTTTTTTTTT-CTCTGGTAGCGTTTTGCTGCTCCTTT

TTTCACTCAAGTGTTGCCAATTAA-TTGACAAAAAATGGTTTCTGTTTCATATAGAAACT

ATGTTTTTGTTGTGTAGTCATACATTACGGAATCTAGTTT-CCATTAAATAAGTAAC--G

TGAAAAAAAA--TAAAAGGTGAAATATATATTGTTGGAAAAGAAGCTATGAGGTGCAAGA

ACCGATCACATGGAGAAGGCAATGAAAGACAAGGAGGAGCAATGGAAGA-----------

---------GAGAAAATGAGAAGATGGAAGGGATGTGAAAATGTTTGAAAAAAACGAGGT

GATCAGTTTTAAAATACGAATTTAGTATTTTCTTTTTAAGAAAATTCTTTCG-AAAGTCG

TGTTTTAAAACATGACTTTTATT-ATTTGAAGTCG---TGTTCTAAAACATGACTTA--T

TCATATCCTT-AATATTTTT---------AAAATTTATCCATTTGTAATATTTTTTAAAA

ATTGACCCATATATGTAAAATACCCGTCAAGATCTCTTTATTATTTTGAAAGCGAAAGCA

TATCACTTCAAACACAATGGAATCGAGGCTATTGACTAAGTATAAATAGAGAAGACTTCA

TATCGGGGTTCATAATTCATAACAAAGCAAACGAGTATATAAGAAAGCATAAGCCAAATT

TTGAGTAAACTAGTGTGCACACTATCCCATGCCTAGTGGAAGTAGGGATCCTCTCGTTGT

TGGGGGAGTAATTGGGGATGTATTGGATCCTTTTGAATATTCTATTCCTATGAGGGTTAC

CTACAATAACAGAGATGTCAGCAATGGATGTGAATTCAAACCCTCACAAGTTGTCAACCA

ACCAAGGGTAAATATCGGTGGTGATGACC

>CS32.06

TATTAGACTCCTAATTTAATATTCTTGTTTTATTATAATGCTGATAAGTCTTGTAAATAA

GGGTGAGAAGCACGAATAATTAGTTCATGAGATGTGTATAATTATTATCCTACACGACTT

ATCTTTGATATTTCACACAAGTCTTTCAATGTATAACAAAAACTTTTTAGATACATTTAG

ACTATAGAACTAACAAGTTATATTTTGAACC-AAAAAACAAAGAGAGAGAGAGAGGAACA

TAGAGAAAAGAAGATATGAGAGTTTTTTTTTTTTTTTT---CTAAAACAGAAAGAAACTC

ATTATATAATAAACAAATTACTTTGAGACAAATTAACGTATGTAATAAAACAAAATCAAA

GTGGTAATTAAAATTATTTAATGGTAAACAGTCTAATAGTTAAAATAAAAATGGAAATCA

CATAAATTTTGTAATTGGCCCATTAAAAACAACACTAAGCTTTTAATTTGATTTTGAAAT

TCAAAATAATTTTATTAAATCACAAAGTAAAAGGTTTACAAAGCCGATCATGACAGTGCA

TGTGGGAGGCAAATCGGCATATTTGCACTACAAAAGG-ACCCATGAAGTCTCTGAACATG

CACGCAACACTTTAATCTCTTATTAGTTACTTTGAAAGCTTATTTATATATATAGACACG

CGTAAAAACTTCTTAACCAAGATTTTTTT-ACGTGCTTCC-TTCGCGTTTAATTTGGACC

ATCAAACCGTGCTCAACAGATAAAGAAAAGGGTGCTTTTGATTCAAGATATTGGCC-GAA

AAACACAAGATAGATCCTT-CGATAGATTAAGCCACGCATGAAACGCGAATCCAAAGTGA

TGAAGAAGTGCAGATAGATATTCGTTCACCATATAGAGGAGAATATATCATTCCTACAAA

CAAAATTGATGATGTTTCTAGAATAGAAACGTTAGAATCGAATATTCATCTGTTCCGGGT

GGGGAAGGGCTACTGAAAAGCTGAACTTTTTTAAGAGTTCGAACTTCGAAAGTCAATCAA

TGATTCTTAATTGAGAGGGAAAAGCTATAAAGGACGAGAAGGGAAGGAAATGTTTATAGG

GAATGAATTTCTAATGCATGATGGGAATGACGGATATGAGGAATATATAAAGGTGATTCT

TATATACATTGACATGGATCGGAATCTATTAACTTAAAGTTATTGGGGTCGTGAAATTTA

TTTAATTTTTCTAATTCGTGTCACATGTCACGACAATTAGTGGGG-AACATTTATGTATA

TAGAAATTTCAGAAATTTCTAGCAGCGTGATAAATATAAAGATTTTGGCACAATAAGTTT

TTGGATCATAATGAGTTT-CTATTTAATCAA-GGCA-TGTAA-TCTATTTTATTATTTAG

-TAGGG-AAACTGAAAACTTAGGGTTGCTATTTGTAGCTCCCACCCTTCTTAGTTCTTAC

TCTTTTCAATATTTTTTAAAAGTTTTA-TATAATACCT-AAATT-GCCCTCCTCCT----

----------------------------------------CACCCCATTGCTCTTCCGTT

GCTCCTCCATGTG-AGTTCTTGTCTTTTTTTTT-CTCTGGTAGCGTTTTGCTGCTCCTTT

TTTCACTCAAGTGTTGCCAATTAA-TTGACAAAAAATGGTTTCTGTTTCATATAGAAACT

ATGTTTTTGTTGTGTAGTCATACATTACGGAATCTAGTTT-CCATTAAATAAGTAAC--G

TGAAAAAAAA--TAAAAGGTGAAATATATATTGTTGGAAAAGAAGCTATGAGGTGCAAGA

ACCGATCACATGGAGAAGGCAATGAAAGACAAGGAGGAGCAATGGAAGA-----------

---------GAGAAAATGAGAAGATGGAAGGGATGTGAAAATGTTTGAAAAAAACGAGGT

GATCAGTTTTAAAATACGAATTTAGTATTTTCTTTTTAAGAAAATTCTCTCG-AAAGTCG

TGTTTTAGAACATGACTTTTATT-ATTTGAAGTCG---TGTTCTAAAACATGACTTA--T

TCATATCCTT-AATATTTTT---------AAAATTTATCCATTTGTAATATTTTTTAAAA

ATTGACCCATATATGTAAAATACCCGTCAAGATCTCTTTATTATTTTGAAAGCGAAAGCA

TATCACTTCAAACACAATGGAATCGGGGCTATTGACTGAGTATAAATAGAGAAGACTTCA

TATCGGGGTTCATAATTCATAACAAAGCAAACGAGTATATAAGAAAGCATAAGCCAAATT

TTGAGTAAACTAGTGTGCACACTATCCCATGCCTAGTGGAAGTAGGGATCCTCTCGTTGT

TGGGGGAGTAATTGGGGATGTATTGGATCCTTTTGAATATTCTATTCCTATGAGGGTTAC

CTACAATAACAGAGATGTCAGCAATGGATGTGAATTCAAACCCTCACAAGTTGTCAACCA

ACCAAGGGTAAATATCGGTGGTGATGACC

>CS46.B1

TATTAGACTCCTAATTTAATATTCTTGTTTTATTATAATGCTGATAAGTCTTGTAAATAA

GGGTGAGAAGCACGAATAATTAGTTCATGAGATGTGTATAATTATTATCCTACACGACTT

ATCTTTGATATTTCACACAAGTCTTTCAATGTATAACAAAAACTTTTTAGATACATTTAG

ACTATAGAACTAACAAGTTATATTTTGAACC-AAAAAACAAAGAGAGAGAGAGAGGAACA

TAGAGAAAAGAAGATATGAGAGTTTTTTTTTTTTTTTTT--CTAAAACAGAAAGAAACTC

ATTATATAATAAACAAATTACTTTGAGACAAATTAACGTATGTAATAAGACAAAATCAAA

GTGGTAATTAAAATTATTTAATGGTAAACAGTCTAATAGTTAAAATAAAAATGGAAATCA

CATAAATTTTGTAATTGGCCCATTAAAAACAACACTAAGCTTTTAATTTGATTTTGAAGT

TCAAAATAATTTTATTAAATCACAAAGTAAAAGGTTTACAAAGCCGATCATGACAGTGCA

TGTGGGAGGCAAATCGGCATATTTGCACTACAAAAGG-ACCCATGAAGTCTCTGAACATG

CACGCAACACTTTAATCTCTTATTAGTTACTTTGAAAGCTTATTTATATATATAGACACG

CGTAAAAACTTCTTAACCAAGATTTTTTT-ACGTGCTTCC-TTCGCGTTTAATTTGGACC

ATCAAACCGTGCTCAACAGATAAAGAAAAGGGTGCTTTTGACTCAAGATATTGGCC-GAA

AAACACAAGATAGATCCTT-CGATAGATTAAGCCACGCATGAAACGCGAATCCAAAGTGA

TGAAGAAGTGCAGATAGATATTCGTTCACCATATAGAGGAGAATATATCCTTCCTACAAA

CAAAATTGATGATGTTTCTAGAATAGAAACGTTAGAATCGAATATTCATCTGTTCCGGGT

GGGGAAGGGCTACTGAAAAGCTGAACTTTTTTAAGAGTTCGAACTTCGAAAGTCAATCAA

TGATTCTTAATTGAGAGGGAGAAGCTATAAAGGACGAGAAGAGAAGGAAATGTTTATAGG

GAATGAATTTCTAACGCATGATGGGAATGACGGATATGAGGAATATATAAAGGTGATTCT

TATATACATTGACATGGATCGGAATCTATTAACTTAAAGTTATTGGGGTCGTGAAATTTA

TTTAATTTTTCTAATTCGTGTCACATGTCACGACAATTAGTGGGG-AACATTTATGTATA

TAGAAATTTCAGAAATTTCTAGCAGCGTGATAAATATAAAGATTTTGGCACAATAAGTTT

TTGGATCATAATGAGTTT-CTATTTAATCAA-GGCA-TGTAA-TCTATTTTATTATTTAG

-TAGGG-AAACTGAAAACTTAGGGTTGCTATTTGTAGCTCCCACCCTTCTTAGTTCTTAC

TCTTTTCAATATTTTTTAAAAGTTTTA-TATAATACCT-AAATT-GCCCTCCTCCT----

----------------------------------------TACCCCATTGCTCTTCCGTT

GCTCCTCCATGTG-AGTTCTTGTCTTTTTTTTT-CTCTGGTAGCGTTTTGCTGCTCCTTT

TTTCACTCAAGTGTTGCCAATTAA-TTGACAAAAAATGGTTTCTGTTTCATATAGAAACT

ATGTTTTTGTTGTGTAGTCATACATTACGGAATCTAGTTT-CCATTAAATAAGTAAC--G

TGAAAAAAAA--TAAAAGGTGAAATATATATTGTTGGAAAAGAAGCTATGAGGTGCAAGA

ACCGATCACATGGAGAAGGCAATGAAAGACAGGGAGGAGCAATGGAAGA-----------

---------GAGAAAATGGGAAGATGGAAGGGATGTGAAAATGTTTGAAAAAAACGAGGT

GATCAGTTTTAAAATACGAATTTAGTATTTTCTTTTTAAGAAAATTCTTTCG-AAAGTCG

TGTTTTAAAACATGACTTTTATT-ATTTGAAGTCG---TGTTCTAAAACATGACTTA--T

TCATATCCTT-AGTATTTTT---------AAAATTTATCCATTTGTAATATTTTTTAAAA

ATTGACCCATATATGTAAAATACCCGTCAAGATCTCTTTATTATTTTGAAAGCGAAAGCA

TATCACTTCAAACACAATGGAATCGAGGCTATTGACTAAGTATAAATAGAGAAGACTTCA

TATCGGGGTTCATAATTCATAACAAAGCAAACGAGTATATAAGAAAGCATAAGCCAAATT

TTGAGTAAACTAGTGTGCACACTATCCCATGCCTAGTGGAAGTAGGGATCCTCTCGTTGT

TGGGGGAGTAATTGGGGATGTATTGGATCCTTTTGAATATTCTATTCCTATGAGGGTTAC

CTACAATAACAGAGATGTCAGCAATGGATGTGAATTCAAACCCTCACAAGTTGTCAACCA

ACCAAGGGTAAATATCGGTGGTGATGACC

>CS02.20

TATTAGACTCCTAATTTAATATTCTTGTTTTATTATAATGCTGATAAGTCTTGTAAATAA

GGGTGAGAAGCACGAATAATTAGTTCATGAGATGTGTATAATTATTATCCTACACGACTT

ATCTTTGATATTTCACACAAGTCTTTCAATGTATAACAAAAACTTTTTAGATACATTTAG

ACTATAGAACTAACAAGTTATATTTTGAACC-AAAAAACAAAGAGAGAGAGAGAGGAACA

TAGAGAAAAGAAGATATGAGAGTTTTTTTTTTTTTTTT---CTAAGACAGAAAGAAACTC

ATTATATAATAAACAAATTACTTTGAGACAAATTAACGTATGTAATAAAACAAAATCAAA

GTGGTAATTAAAATTATTTAATGGTAAACAGTCTAATAGTTAAAATAAAAATGGAAATCA

CATAAATTTTGTAATTGGCCCATTAAAAACAACACTAAGCTTTTAATTTGATTTTGAAAT

TCAAAATAATTTTATTAAATCACAAAGTAAAAGGTTTACAAAGCCGATCATGACAGTGCA

TGTGGGAGGCAAATCGGCATATTTGCACTACAAAAGG-ACCCATGAAGTCTCTGAACATG

CACGCAACACTTTAATCTCTTATTAGTTACTTTGAAAGCTTATTTATATATATAGACACG

CGTAAAAACTTCTTAACCAAGATTTTTTT-ACGTGCTTCC-TTCGCGTTTAATTTGGACC

ATCAAACCGTGCTCAACAGATAAAGAAAAGGGTGCTTTTGATTCAAGATATTGGCC-GAA

AAACACAAGATAGATCCTT-CGATAGATTAAGCCACGCATGAAACGCGAATCCAAAGTGA

TGAAGAAGTGCAGATAGATATTCGTTCACCATATAGAGGAGAATATATCATTCCTACAAA

CAAAATTGATGATGTTTCTAGAATAGAAACGTTAGAATCGAATATTCATCTGTTCCGGGT

GGGGAAGGGCTACTGAAAAGCTGAACTTTTTTAAGAGTTCGAACTTCGAAAGTCAATCAA

TGATTCTTAATTGAGAGGGAAAAGCTATAAAGGACGAGAAGGGAAGGAAATGTTTATAGG

GAATGAATTTCTAATGCATGATGGGAATGACAGATATGAGGAATATATAAAGGTGATTCT

TATATACATTGACATGGATCGAAATCTATTAACTTAAAGTTATTGGGGTCGTGAAATTTA

TTTAATTTTTCTAATTCGTGTCACATGTCACGACAATTAGTGGGG-AACATTTATGTATA

TAGAAATTTCAGAAATTTCTAGCAGCGTGATAAATATAAAGATTTTGGCACAATAAGTTT

TTGGATCATAATGAGTTT-CTATTTAATCAA-GGCA-TGTAA-TCTATTTTATTATTTAG

-TAGGG-AAACTGAAAACTTAGGGTTGCTATTTGTAGCTCCCACCCTTCTTAGTTCTTAC

TCTTTTCAATATTTTTTAAAAGTTTTA-TATAATACCT-AAATT-GCCCTCCTCCT----

----------------------------------------CACCCCATTGCTCTTCCGTT

GCTCCTCCATGTG-AGTTCTTGTCTTTTTTTT--CTCTGGTAGCGTTTTGCTGCTCCTTT

TTTCACTCAAGTGTTGCCAATTAA-TTGACAAAAAATGGTTTCTGTTTCATATAGAAACT

ATGTTTTTGTTGTGTAGTCATACATTACGGAATCTAGTTT-CCATTAAATAAGTAAC--G

TGAAAAAAAA--TAAAAGGTGAAATATATATTGTTGGAAAAGAAGCTATGAGGTGCAAGA

ACCGATCACATGGAGAAGGCAATGGAAGACAAGGAGGAGCAATGGAGGA-----------

---------GAGAAAATGAGAAGATGGAAGGGATGTGAAAATGTTTGAAAAAAACGAGGT

GATCAGTTTTAAAATACGAATTTAGTATTTTCTTTTTAAGAAAATTCTTTCG-AAAGTCG

TGTTTTAAAACATGACTTTTATT-ATTTGAAGTCG---TGTTCTAAAACATGACTTA--T

TCATATCCTT-AATATTTTT---------AAAATTTATCCATTTGTAATATTTTTTAAAA

ATTGACCCATATATGTAAAATACCCGTCAAGATCTCTTTATTATCTTGAAAGCGAAAGCA

TATCACTTCAAACACAATGGAATCGAGGCTATTGACTAAGTATAAATAGAGAAGACTTCA

TATCGGGGCTCATAATTCATAACAAAGCAAACGAGTATATAAGAAAGCATAAGCCAAATT

TTGAGTAAACTAGTGTGCACACTATCCCATGCCTAGTGGAAGTAGGGATCCTCTCGTTGT

TGGGGGAGTAATTGGGGATGTATTGGATCCCTTTGAATATTCTATTCCTATGAGGGTTAC

CTACAATAACAGAGATGTCAGCAATGGATGTGAATTCAAACCCTCACAAGTTGTCAACCA

ACCAAGGGTAAATATCGGTGGTGATGACC

>H14.54

TATTAGACTCCTAATTTAATATTCTTGTTTTATTATAATGCTGATAAGTCTTGTAAATAA

GGGTGAGAAGCACGAATAATTAGTTCATGAGATGTGTATAATTATTATCCTACACGACTT

ATCTTTGATATTTCACACAAGTCTTTCAATGTATAACAAAAACTTTTTAGATACATTTAG

ACTATAGAACTAACAAGTTATATTTTGAACC-AAAAAACAAAGAGAGAGAGAGAGGAACA

TAGAGAAAAGAAGATATGAGAGTTTTTTTTTTTTTTTT---CTAAAACAGAAAGAAACTC

ATTATATAATAAACAAATTACTTTGAGACAAATTAACGTATGTAATAAAACAAAATCAAA

GTGGTAATTAAAATTATTTAATGGTAAACAGTCTAATAGTTAAAATAAAAATGGAAATCA

CATAAATTTTGTAATTGGCCCATTAAAAACAACACTAAGCTTTTAATTTGATTTTGAAAT

TCAAAATAATTTTATTAAATCACAAAGTAAAAGGTTTACAAAGCCGATCATGACAGTGCA

TGTGGGAGGCAAATCGGCATATTTGCACTACAAAAGG-ACCCATGAAGTCTCTGAACATG

CACGCAACACTTTAATCTCTTATTAGTTACTTTGAAAGCTTATTTATATATATAGACACG

CGTAAAAACTTCTTAACCAAGATTTTTTT-ACGTGCTTCC-TTCGCGTTTAATTTGGACC

ATCAAACCGTGCTCAACAGATAAAGAAAAGGGTGCTTTTGATTCAAGATATTGGCC-GAA

AAACACAAGATAGATCCTT-CGATAGATTAAGCCACGCATGAAACGCGAATCCAAAGTGA

TGAAGAAGTGCAGATAGATATTCGTTCACCATATAGAGGAGAATACATCATTCCTACAAA

CAAAATTGATGATGTTTCTAGAATAGAAACGTTAGAATCGAATATTCATCTGTTCCGGGT

GGGGAAGGGCTACTGAAAAGCTGAACTTTTTTAAGAGTTCGAACTTCGAAAGTCAATCAA

TGATTCTTAATTGAGAGGGAAAAGCTATAAAGGACGAGAAGGGAAGGAAATGTTTATAGG

GAATGAATTTCTAATGCATGATGGGAATGACAGATATGAGGAATATATAAAGGTGATTCT

TATATACATTGACATGGATCGGAATCTATTAACTTAAAGTTATTGGGGTCGTGAAATTTA

TTTAATTTTTCTAATTCGTGTCACATGTCACGACAATTAGTGGGG-AACATTTATGTATA

TAGAAATTTCAGAAATTTCTAGCAGCGTGATAAATATAAAGATTTTGGCACAATAAGTTT

TTGGATCATAATGAGTTT-CTATTTAATCAA-GGCA-TGTAA-TCTATTTTATTATTTAG

-TAGGG-AAACTGAAAACTTAGGGTTGCTATTTGTAGCTCCCACCCTTCTTAGTTCTTAC

TCTTTTCAATATTTTTTAAAAGTTTTA-TATAATACCT-AAATT-GCCCTCCTCCT----

----------------------------------------CACCCCATTGCTCTTCCGTT

GCTCCTCCATGTG-AGTTCTTGTCTTTTTTTTT-CTCTGGTAGCGTTTTGCTGCTCCTTT

TTTCACTCAAGTGTTGCCAATTAA-TTGACAAAAAATGGTTTCTGTTTCATATAGAAACT

ATGTTTTTGTTGTGTAGTCATACATTACGGAATCTAGTTT-CCATTAAATAAGTAAC--G

TGAAAAAAAA--TAAAAGGTGAAATATATATTGTTGGAAAAGAAGCTATGAGGTGCAAGA

ACCGATCACATGGAGAAGGCAATGAAAGACAAGGAGGAGCAATGGAAGA-----------

---------GAGAAAATGAGAAGATGGGAGGGATGTGAAAATGTTTGAAAAAAACGAGGT

GATCAGTTTTAAAATACGAATTTAGTATTTTCTTTTTAAGAAAATTCTTTCG-AAAGTCG

TGTTTTAAAACATGACTTTTATT-ATTTGAAGTCG---TGTTCTAAAACATGACTTA--T

TCATACCCTT-AATATTTTT---------AAAATTTATCCATTTGTAATATTTTTTAAAA

ATTGACCCATATATGTAAAATACCCGTCAAGATCTCTTTATTATTTTGAAAGCGAAAGCA

TATCACTTCAAACACAATGGAATCGAGGCTATTGACTAAGTATAAATAGAGAAGACTTCA

TATCGGGGTTCATAATTCATAACAAAGCAAACGAGTATATAAGAAAGCATAAGCCAAATT

TTGAGTAAACTAGTGTGCACACTATCCCATGCCTAGTGGAAGTAGGGATCCTCTCGTTGT

TGGGGGAGTAATTGGGGATGTATTGGATCCTTTTGAATATTCTATTCCTATGAGGGTTAC

CTACAATAACAGAGATGTCAGCAATGGATGTGAATTCAAACCCTCACAAGTTGTCAACCA

ACCAAGGGTAAATATCGGTGGTGATGACC

>H15.G2

TATTAGACTCCTAATTTAATATTCTTGTTTTATTATAATGCTGATAAGTCTTGTAAATAA

GGGTGAGAAGCACGAATAATTAGTTCATGAGATGTGTATAATTATTATCCTACACGACTT

ATCTTTGATATTTCACACAAGTCTCTCAATGTATAACAAAAACTTTTTAGATACATTTAG

ACTATAGAACTAACAAGTTATATTTTGAACC-AAAAAACAAAGAGAGAGAGAGAGGAACA

TAGAGAAAAGAAGATATGAGAGTTTTTTTTTTTTTTT----CTAAAGCAGAAAGAAACTC

ATTATATAATAAACAAATTACTTTGAGACAAATTAACGTATGTAATAAAACAAAATCAAA

GTGGTAATTAAAATTATTTAATGGTAAACAGTCTAATAGTTAAAATAAAAATGGAAATCA

CATAAATTTTGTAATTGGCCCATTAAAAACAACACTAAGCTTTTAATTTGATTTTGAAAT

TCAAAATAATTTTATTAAATCACAAAGTAAAAGGTTTACAAAGCCGATCATGACAGTGCA

TGTGGGAGGCAAATCGGCATATTTGCACTACAAAAGG-ACCCATGAAGTCTCTGAACATG

CACGCAACACTTTAATCTCTTATTAGTTACTTTGAAAGCTTATTTATATATATAGACACG

CGTAAAAACTTCTTAACCAAGATTTTTTT-ACGTGCTTCC-TTCGCGTTTAATTTGGACC

ATCAAACCGTGCTCAACAGATAAAGAAAAGGGTGCTTTTGATTCAAGATATTGGCC-GAA

AAACACAGGATAGATCCTT-CGATAGATTAAGCCACGCATGAAACGCGAATCCAAAGTGA

TGAAGAAGTGCGGATAGATATTCGTTCACCATATAGAGGAGAATATATCATTCCTACAAA

CAAAATTGATGATGTTTCTAGAATAGAAACGTTAGAATCGAATATTCATCTGTTCCGGGT

GGGGAAGGGCTACTGAAAAGCTGAACTTTTTTAAGAGTTCGAACTTCGAAAGTCAATCAA

TGATTCTTAATTGAGAGGGAAAAGCTATAAAGGACGAGAAGGGAAGGAAATGTTTATAGG

GAATGAATTTCTAATGCATGATGGGAATGACAGATATGAGGAATATATAAAGGTGATTCT

TATATACATTGACATGGATCGGAATCTATTAACTTAAAGTTATTGGGGTCGTGAAATTTA

TTTAATTTTTCTAATTCGTGTCACATGTCACGACAATTAGTGGGG-AACATTTATGTATA

TAGAAATTTCAGAAATTTCTAGCAGCGTGATAAATATAAAGATTTTGGCACAATAAGTTT

TTGGATCATAATGAGTTT-CTATTTAATCAA-GGCA-TGTAA-TCTATTTTATTATTTAG

-TAGGG-AAACTGAAAACTTAGGGTTGCTATTTGTAGCTCCCACCCTTCTTAGTTCTTAC

TCTTTTCAATATTTTTTAAAAGTTTTA-TATAATACCT-AAATT-GCCCTCCTCCT----

----------------------------------------CACCCCATTGCTCTTCCGTT

GCTCCTCCATGTG-AGTTCTTGTCTTTTTTTTT-CTCTGGTAGCGTTTTGCTGCTCCTTT

TTTCACTCAAGCGTTGCCAATTAA-TTGACAAAAAATGGTTTCTGTTTCATATAGAAACT

ATGTTTTTGTTGTGTAGTCATACATTACGGAATCTAGTTT-CCATTAAATAAGTAAC--G

TGAAAAAAAA--TAAAAGGTGAAATATATATTGTTGGAAAAGAAGCTATGAGGTGCAAGA

ACCGATCGCATGGAGAAGGCAATGAAAGACAAGGAGGAGCAATGGAAGA-----------

---------GAGAAAATGAGAAGATGGAAGGGATGTGAAAATGTTTGAAAAAAACGAGGT

GATCAGTTTTAAAATACGAATTTAGTATTTTCTTTTTAAGAAAATTCTTTCG-AAAGTCG

TGTTTTGAAACATGACTTTTATT-ATTTGAAGTCG---TGTTCTAAAACATGACTTA--T

TCATATCCTT-AATATTTTT---------AAAATTTATCCATTTGTAATATTTTTTAAAA

ATTGACCCATATATGTAAAATACCCGTCAAGATCTCTTTATTATTTTGAAAGCGAAAGCA

TATCACTTCAAACACAATGGAATCGAGGCTATTGACTAAGTATAAATAGAGAAGACTTCA

TATCGGGGTTCATAATTCATAACAAAGCAAACGAGTATATAAGAAAGCATAAGCCAAATT

TTGAGTAAACTAGTGTGCACACTATCCCATGCCTAGTGGAAGTAGGGATCCTCTCGTTGT

TGGGGGAGTAATTGGGGATGTATTGGATCCTTTTGAATATTCTATTCCTATGAGGGTTAC

CTACAATAACAGAGATGTCAGCAATGGATGTGAATTCAAACCCTCACAAGTTGTCAACCA

ACCAAGGGTAAATATCGGTGGTGATGACC

>CS12.35

TATTAGACTCCTAATTTAATATTCTTGTTTTATTATAATGCTGATAAGTCTTGTAAATAA

GGGTGAGAAGCACAAATAATTAGTTCATGAGATGTGTATAATTATTATCCTACACGACTT

ATCTTTGATATTTCACACAAGTCTTTCAATGTATAACAAAAACTTTTTAGATACATTTAG

ACTATAGAACTAACAAGTTATATTTTGAACC-AAAAAACAAAGAGAGAGAGAGAGGAACA

TAGAGAAAAGAAGATATGAGAGTTTTTTTTTTTTTT-----CTAAAACAGAAAGAAACTC

ATTATATAATAAACAAATTACTTTGAGACAAATTAACGTATGTAATAAAACAAAATCAAA

GTGGTAATTAAAATTATTTAATGGTAAACAGTCTAATAGTTAAAATAAAAATGGAAATCA

CATAAATTTTGTAATTGGCCCATTAAAAACAACACTAAGCTTTTAATTTGATTTTGAAAT

TCAAAATAATTTTATTAAATCACAAAGTAAAAGGTTTACAAAGCCGATCATGACAGTGCA

TGTGGGAGGCAAATCGGCATATTTGCACTACAAAGGG-ACCCATGAAGTCTCTGAACATG

CACGCAACACTTTAATCTCTTATTAGTTACTTTGAAAGCTTATTTATATATATAGACACG

CGTAAAAACTTCTTAACCAAGATTTTTTT-ACGTGCTTCC-TTCGCGTTTAATTTGGACC

ATCAAACCGTGCTCAACAGATAAAGAAAAGGGTGCTTTTGATTCAAGATATTGGCC-GAA

AAACACATGATAGATCCTT-CGATAGATTAAGCCACGCATGAAACGCGAATCCAAAGTGA

TGAAGAAGTGCAGATAGATATTCGTTCACCATATAGAGGAGAATATATCATTCCTACAAA

CAAAATTGATGATGTTTCTAGAATAGAAACGTTAGAATCGAATATTCATCTGTTCCGGGT

GGGGAAGGGCTACTGAAAAGCTGAACTTTTTTAAGAGTTCGAACTTCGAAAGTCAATCAA

TGATTCTTAATTGAGAGGGAAAAGCTATAAAGGACGAGAAGGGAAGGAAATGTTTATAGG

GAATGAATTTCTAATGCATGATGGGAATGACAGATATGAGGAATATATAAAGGTGATTCT

TATATACATTGACATGGATCGGAATCTATTAACTTAAAGTTATTGGGGTCGTGAAATTTA

TTTAATTTTTCTAATTCGTGTCACATGTCACGACAATTAGTGGGG-AACATTTATGTATA

TAGAAATTTCAGAAATTTCTAGCAGCGTGATAAATATAAAGATTTTGGCACAATAAGTTT

TTGGATCATAATGAGTTT-CTATTTAATCAA-GGCA-TGTAA-TCTATTTTATTATTTAG

-TAGGG-AAACTGAAAACTTAGGGTTGCTATTTGTAGCTCCCACCCTTCTTAGTTCTTAC

TCTTTTCAATATTTTTTAAAAGTTTTA-TATAATACCT-AAATT-GCCCTCCTCCT----

----------------------------------------CACCCCATTGCTCTTCCGTT

GCTCCTCCATGTG-AGTTCTTGTCTTTTTTTT--CTCTGGTAGCGTTTTGCTGCTCCTTT

TTTCACTCAAGTGTTGCCAATTAA-TTGACAAAAAATGGTTTCTGTTTCATATAGAAACT

ATGTTTTTGTTGTGTAGTCATACATTACGGAATCTAGTTT-CCATTAAATAAGTAAC--G

TGAAAAAAAA--TAAAAGGTGAAATATATATTGTTGGAAAAGAAGCTATGAGGTGCAAGA

ACTGATCACATGGAGAAGGCAATGAAAGACAAGGAGGAGCAATGGAAGA-----------

---------GAGAAAATGAGAAGATGGAAGGGATGTGAAAATGTTTGAAAAAAACGAGGT

GATCAGTTTTAAAATACGAATTTAGTATTTTCTTTTTAAGAAAATTCTTTCG-AAAGTCG

TGTTTTAAAACATGACTTTTATT-ATTTGAAGTCG---TGTTCTAAAACATGACTTA--T

TCATATCCTT-AATATTTTT---------AAAATTTATCCATTTGTAATATTTTTTAAAA

ATTGACCCATATATGTAAAATACCCGTCAAGATCTCTTTATTATTTTGAAAGCGAAAGCA

TATCACTTCAAACACAATGGAATCGAGGCTATTGACTAAGTATAAATAGAGAAGACTTCA

TATCGGGGTTCATAATTCATAACAAAGCAAACGAGTATATAAGAAAGCATAAGCCAAATT

TTGAGTAAACTAGTGTGCACACTATCCCACGCCTAGTGGAAGTAGGGATCCTCTCGTTGT

TGGGGGAGTAATTGGGGATGTATTGGATCCTTTTGAATATTCTATTCCTATGAGGGTTAC

CTACAATAACAGAGATGTCAGCAATGGATGTGAATTCAAACCCTCACAAGTTGTCAACCA

ACCAAGGGTAAATATCGGTGGTGATGACC

>CS24.32

TATTAGACTCCTAATTTAATATTCTTGTTTTATTATAATGCTGATAAGTCTTGTAAATAA

GGGTGAGAAGCACGAATAATTAGTTCATGAGATGTGTATAATTATTATCCTACACGACTT

ATCTTTGATATTTCACACAAGTCTTTCAATGTATAACAAAAACTTTTTAGATACATTTAG

ACTATAGAACTAACAAGTTATATTTTGAACC-AAAAAACAAAGAGAGAGAGAGAGGAACA

TAGAGAAAAGAAGATATGAGAGTTTTTTTTTTTTTTTTT--CTAAAACAGAAAGAAACTC

ATTATATAATAAACAAATTACTTTGAGACAAATTAACGTATGTAATAAAACAAAATCAAA

GTGGTAATTAAAATTATTTAATGGTAAACAGTCTAATAGTTAAAATAAAAATGGAAATCA

CATAAATTTTGTAATTGGCCCATTAAAAACAACACTAAGCTTTTAATTTGATTTTGAAAT

TCAAAATAATTTTATTAAATCACAAAGTAAAAGGTTTACAAAGCCGATCATGACGGTGCA

TGTGGGAGGCAAATCGGCATATTTGCACTACAAAAGG-ACCCATGAAGTCTCTGAACATG

CACGCAACACTTTAATCTCTTATTAGTTACTTTGAAAGCTTATTTATATATATAGACACG

CGTAAAAACTTCTTAACCAAGATTTTTTT-ACGTGCTTCC-TTCGCGTTTAATTTGGACC

ATCAAACCGTGCTCAACAGATAAAGAAAAGGGTGCTTTTGATTCAAGATATTGGCC-GAA

AAACACAAGATAGATCCTT-CGATAGATTAAGCCACGCATGAAACGCGAATCCAAAGTGA

TGAAGAAGTGCAGATAGATATTCGTTCACCATATAGAGGAGAATATATCATTCCTACAAA

CAAAATTGATGATGTTTCTAGAATAGAAACGTTAGAATCGAATATTCATCTGTTCCGGGT

GGGGGAGGGCTACTGAAAAGCTGAACTTTTTTAAGAGTTCGAACTTCGAAAGTCAATCAA

TGATTCTTAATTGAGGGGGAAAAGCTATAAAGGACGAGAAGGGAAGGAAATGTTTATAGG

GAATGAATTTCTAATGCATGATGGGAATGACAGATATGAGGAATATATAAAGGTGATTCT

TATATACATTGACATGGATCGGAATCTATTAACTTAAAGTTATTGGGGTCGTGAAATTTA

TTTAATTTTTCTAATTCGTGTCACATGTCACGACAATTAGTGGGG-AACATTTATGTATA

TAGAAATTTCAGAAATTTCTAGCAGCGTGATAAATATAAAGATTTTGGCACAATAAGTTT

TTGGATCATAATGAGTTT-CTATTTAATCAA-GGCA-TGTAA-TCTATTTTATTATTTAG

-TAGGG-AAACTGAAAACTTAGGGTTGCTATTTGTAGCTCCCACCCTTCTTAGTTCTTAC

TCTTTTCAATATTTTTTAAAAGTTTTA-TATAATACCT-AAATT-GCCCTCCTCCT----

----------------------------------------CACCCCATTGCTCTTCCGTT

GCTCCTCCATGTG-AGTTCTTGTCTTTTTTTTT-CTCTGGTAGCGTTTTGCTGCTCCTTT

TTTCACTCAAGTGTTGCCAATTAA-TTGACAAAAAATGGTTTCTGTTTCATATAGAAACT

ATGTTTTTGTTGTGTAGTCATACATTACGGAATCTAGTTT-CCATTAAATAAGTAAC--G

TGAAAAAAAA--TAAAAGGTGAAATATATATTGTTGGAAAAGAAGCTATGAGGTGCAAGA

ACCGATCACATGGAGAAGGCAATGAAAGACAAGGAGGAGCAATGGAAGA-----------

---------GAGAAAATGAGAAGATGGAAGGGATGTGAAAATGTTTGAAAAAAACGAGGT

GATCAGTTTTAAAATACGAATTTAGTATTTTCTTTTTAAGAAAATTCTTTCG-AAAGTCG

TGTTTTAAAACATGACTTTTATT-ATTTGAAGTCG---TGTTCTAAAACATGACTTA--T

TCATATCCTT-AATATTTTT---------AAAATTTATCCATTTGTAATATTTTTTAAAA

ATTGACCCATATATGTAAAATACCCGTCAAGATCTCTTTATTATTTCGAAAGCGAAAGCA

TATCACTTCAAACACAATGGAATCGAGGCTATTGACTAAGTATAAATAGAGAAGACTTCA

TATCGGGGTTCATAATTCATAACAAAGCAAACGAGTATATAAGAAAGCATAAGCCAAATT

TTGAGTAAACTAGTGTGCACACTATCCCATGCCTAGTGGAAGTAGGGATCCTCTCGTTGT

TGGGGGAGTAATTGGGGATGTATTGGATCCTTTTGAATATTCTATTCCTATGAGGGTTAC

CTACAATAACAGAGATGTCAGCAATGGATGTGAATTCAAACCCTCACAAGTTGTCAACCA

ACCAAGGGTAAATATCGGTGGTGATGACC

>H01.07c

TATTAGACTCCTAATTTAATATTCTTGTTTTATTATAATGCTGATAAGTCTTGTAAATAA

GGGTGAGAAGCACGAATAATTAGTTCATGAGATGTGTATAATTATTATCCTACACGACTT

ATCTTTGATATTTCACACAAGTCTTTCAATGTATAACAAAAACTTTTTAGATACATTTAG

ACTATAGAACTAACAAGTTATATTTTGAACC-AAAAAACAAAGAGAGAGAGAGAGGAACA

TAGAGAAAAGAAGATATGAGAGTTTTTTTTTTTTTTTT---CTAAAACAGAAAGAAACTC

ATTATATAATAAACAAATTACTTTGAGACAAATTAACGTATGTAATAAAACAAAATCAAA

GTGGTAATTAAAATTATTTAATGGTAAACAGTCTAATAGTTAAAATAAAAATGGAAATCA

CATAAATTTTGTAATTGGCCCATTAAAAACAACACTAAGCTTTTAATTTGATTTTGAAAT

TCAAAATAATTTTATTAAATCACAAAGTAAAAGGTTTACAAAGCCGATCATGACAGTGCA

TGTGGGAGGCAAATCGGCATATTTGCACTACAAAAGG-ACCCATGAAGTCTCTGAACATG

CACGCAACACTTTAATCTCTTATTAGTTACTTTGAAAGCTTATTTATATATATAGACACG

CGTAAAAACTTCTTAACCAAGATTTTTTT-ACGTGCTTCC-TTCGCGTTTAATTTGGACC

ATCAAACCGTGCTCAACAGATAAAGGAAAGGGTGCTTTTGATTCAAGATATTGGCC-GAA

AAACACAAGATAGATCCTC-CGATAGATTAAGCCACGCATGAAACGCGAATCCAAAGTGA

TGAAGAAGTGCAGATAGATATTCGTTCACCATATAGAGGAGAATATATCATTCCTACAAA

CAAAATTGATGATGTTTCTAGAATAGAAACGTTAGAATCGAATATTCATCTGTTCCGGGT

GGGGAAGGGCTACTGAAAAGCTGAACTTTTTTAAGAGTTCGAACTTCGAAAGTCAATCAA

TGATTCTTAATTGAGAGGGAAAAGCTATAAAGGACGAGAAGGGAAGGAAATGTTTATAGG

GAATGAATTTCTAATGCATGATGGGAATGACAGATATGAGGAATATATAAAGGTGATTCT

TATATACATTGACATGGATCGGAATCTATTAACTTAAAGTTATTGGGGTCGTGAAATTTA

TTTAATTTTTCTAATTCGTGTCACATGTCACGACAATTAGTGGGG-AACATTTATGTATA

TAGAAATTTCAGAAATTTCTAGCAGCGTGATAAATATAAAGATTTTGGCACAATAAGTTT

TTGGATCATAATGAGTTT-CTATTTAATCAA-GGCA-TGTAA-TCTATTTTATTATTTAG

-TAGGG-AAACTGAAAACTTAGGGTTGCTATTTGTAGCTCCCACCCTTCTTAGTTCTTAC

TCTTTTCAATATTTTTTAAAAGTTTTA-TATAATACCT-AAATT-GCCCTCCTCCT----

----------------------------------------CACCCCATTGCTCTTCCGTT

GCTCCTCCATGTG-AGTTCTTGTCTTTTTTTTT-CTCTGGTAGCGTTTTGCAGCTCCTTT

TTTCACTCAAGTGTTGCCAATTAA-TTGACAAAAAATGGTTTCTGTTTCATATAGAAACT

ATGTTTTTGTTGTGTAGTCATACATTACGGAATCTAGTTT-CCATTAAATAAGTAAC--G

TGAAAAAAAA--TAAAAGGTGAAATATATATTGTTGGAAAAGAAGCTATGAGGTGCAAGA

ACCGATCACATGGAGAAGGCAATGAAAGACAAGGAGGAGCAATGGAAGA-----------

---------GAGAAAATGAGAAGATGGAAGGGATGTGAAAATGTTTGAAAAAAACGAGGT

GATCAGTTTTAAAATACGAATTTAGTATTTTCTTTTTAAGAAAATTCTTTCG-AAAGTCG

TGTTTTAAAACATGACTTTTATT-ATTTGAAGTCG---TGTTCTAAAACATGACTTA--T

TCATATCCTT-AATATTTTT---------AAAATTTATCCATTTGTAATATTTTTTAAAA

ATTGACCCATATATGTAAAATACCCGTCAAGATCTCTTTATTATTTTGAAAGCGAAAGCA

TATCACTTCAAACACAATGGAATCGAGGCTATTGACTAAGTATAAATAGAGAAGACTTCA

TATCGGGGTTCATAATTCATAACAAAGCAAACGAGTATATAAGAAAGCATAAGCCAAATT

TTGAGTACACTAGTGTGCACACTATCCCATGCCTAGTGGAAGTAGGGATCCTCTCGTTGT

TGGGGGAGTAATTGGGGATGTATTGGATCCTTTTGAATATTCTATTCCTATGAGGGTTAC

CTACAATAACAGAGATGTCAGCAATGGATGTGAATTCAAACCCTCACAAGTTGTCAACCA

ACCAAGGGTAAATATCGGTGGTGATGACC

>CS63.M8

TATTAGACTCCTAATTTAATATTCTTGTTTTATTATAATGCTGATAAGTCTTGTAAATAA

GGGTGAGAAGCACGAATAATTAGTTCATGAGATGTGTATAATTATTATCCTACACGACTT

ATCTTTGATATTTCACACAAGTCTTTCAATGTATAACAAAAACTTTTTAGATACATTTAG

ACTATAGAACTAACAAGTTATATTTTGAACC-AAAAAACAAAGAGAGAGAGAGAGGAACA

TAGAGAAAAGAAGATATGAGAGTTTTTTTTTTTTTTTTT--CTAAAACAGAAAGAAACTC

ATTATATAATAAACAAATTACTTTGAGACAAATTAACGTATGTAATAAAACAAAATCAAA

GTGGTAATTAAAATTATTTAATGGTAAACAGTCTAATAGTTAAAATAAAAATGGAAATCA

CATAAATTTTGTAATTGGCCCATTAAAAACAACACTAAGCTTTTAATTTGATTTTGAAAT

TCAAAATAATTTTATTAAATCACAAAGTAAAAGGTTTACAAAGCCGATCATGACAGTGCA

TGTGGGAGGCAAATCGGCATATTTGCACTACAAAAGG-ACCCATGAAGTCTCTGAACATG

CACGCAACACTTTAATCTCTTATTAGTTACTTTGAAAGCTTATTTATATATATAGACACG

CGTAAAAACTTCTTAACCAAGATTTTTTT-ACGTGCTTCC-TTCGCGTTTAATTTGGACC

ATCAAACCGTGCTCAACAGATAAAGAAAAGGGTGCTTTTGATTCAAGATATTGGCC-GAA

AAACACAAGATAGATCCTT-CGATAGATTAAGCCACGCATGAAACGCGAATCCAAAGTGA

TGAAGAAGTGCAGATAGATATTCGTTCACCATATAGAGGAGAATATATCATTCCTACAAA

CAAAATTGATGATGTTTCTAGAATAGAAACGTTAGAATCGAATATTCATCTGTTCCGGGT

GGGGAAGGGCTACTGAAAAGCTGAACTTTTTTAAGAGTTCGAACTTCGAAAGTCAATCAA

TGATTCTTAATTGAGAGGGAAAAGCTATAAAGGACGAGAAGGGAAGGAAATGTTTATAGG

GAATGAATTTCTAATGCATGATGGGAATGACAGATATGAGGAATATATAAAGGTGATTCT

TATATACATTGACATGGATCGGAATCTATTAACTTAAAGTTATTGGGGTCGTGAAATTTA

TTTAATTTTTCTAATTCGTGTCACATGTCACGACAATTAGTGGGG-AACATTTATGTATA

TAGAAATTTCAGAAATTTCTAGCAGCGTGATAAATATAAAGATTTTAGCACAATAAGTTT

TTGGATCATAATGAGTTT-CTATTTAATCAA-GGCA-TGTAA-TCTATTTTATCATTTAG

-TAGGG-AAACTGAAAACTTAGGGTTGCTATTTGTAGCTCCCACCCTTCTTAGTTCTTAC

TCTTTTCAATATTTTTTAAAAGTTTTA-TATAATACCT-AAATT-GCCCTCCTCCT----

----------------------------------------CACCCCATTGCTCTTCCGTT

GCTCCTCCATGTG-AGTTCTTGTCTTTTTTTTT-CTCTGGTAGCGTTTTGCTGCTCCTTT

TTTCACTCAAGTGTTGCCAATTAA-TTGACAAAAAATGGTTTCTGTTTCATATAGAAACT

ATGTTTTTGTTGTGTAGTCATACATTACGGAATCTAGTTT-CCATTAAATAAGTAAC--G

TGAAAAAAAA--TAAAAGGTGAAATATATATTGTTGGAAAAGAAGCTATGAGGTGCAAGA

ACCGATCACATGGAGAAGGCAATGAAAGACAAGGAGGAGCAATGGAAGA-----------

---------GAGAAAATGAGAAGATGGAAGGGATGTGAAAATGTTTGAAAAAAACGAGGT

GATCAGTTTTAAAATACGAATTTAGTATTTTCTTTTTAAGAAAATTCTTTCG-AAAGTCG

TGTTTTAAAACATGACTTTTATT-ATTTGAAGTCG---TGTTCTAAAACATGACTTA--T

TCATATCCTT-AATATTTTT---------AAAATTTATCCATTTGTAATATTTTTTAAAA

ATTGACCCATATATGTAAAATACCCGTCAAGATCTCTTTATTATTTTGAAAGCGAAAGCA

TATCACTTCAAACACAATGGAATCGAGGCTATTGACTAAGTATAAATAGAGAAGACTTCA

TATCGGGGTTCATAATTCATAACAAAGCAAACGAGTATATAAGAAAGCATAAGCCAAATT

TTGAGTAAACTAGTGTGCACACTATCCCATGCCTAGTGGAAGTAGGGATCCTCTCGTTGT

TGGGGGAGTAATTGGGGATGTATTGGATCCTTTTGAATATTCTATTCCTATGAGGGTTAC

CTACAATAACAGAGATGTCAGCAATGGATGTGAATTCAAACCCTCACAAGTTGTCAACCA

ACCAAGGGTAAATATCGGTGGTGATGACC

>CS34.40

TATTAGACTCCTAATTTGATATTCTTGTTTTATTATAATGCTGATAAGTCTTGTAAATAA

GGGTGAGAAGCACGAATAATTAGTTCATGAGATGTGTATAATTATTATCCTACACGACTT

ATCTTTGATATTTCACACAAGTCTTTCAATGTATAACAAAAATTTTTTAGATACATTTAG

ACTATAGAACTAACAAGTTATATTTTGAACC-AAAAAACAAAGAGAGAGAG----GAACA

TAGAGAAAAGAAGATATGAGAGTTTTTTTTTTTTTTT----CTAAAACAGAAAGAAACTC

ATTATATAATAAACAAATTAATTTGAGACAAATTAACGTATGTAATAAAACAAAATCAAA

GTGGTAATTAAAATTATTTAATGGTAAACAGTCTAATAGTTAAAATAAAAATGGAAATCA

CATAAATTTTGTAATTGGCCCATTAAAAACAACACTAAGCTTTTAATTTGATTTTGAAAT

TCAAAATAATTTTATTAAATCACAAAGTAAAAGGTTTACAAAGCCGATCATGACAGTGCA

TGTGGGAGGCAAATCGGCATATTTGCACTACAAAAGG-ACCCATGAAGTCTCTGAACATG

CACGCAACACTTTAATCTCTTATTAGTTACTTTGAAAGCTTATTTATATATATAGACACG

CGTAAAAACTTCTTAACCAAGATTTTTTT-ACGTGCTTCC-TTCGCGTTTAATTTGGACC

ATCAAACCGTGCTCAACAGATAAAGAAAAGGGTGCTTTTGATTCAAGATATTGGCC-GAA

AAACACAAGATAGATCCTT-CGATAGATTAAGCCACGCATGAAACGCGAATCCAAAGTGA

TGAAGAAGTGCAGATAGATATTCGTTCACCATATAGAGGAGAATATATCATTCCTACAAA

CAAAATTGATGATGTTTCTAGAATAGAAACGTTAGAATCGAATATTCATCTGTTCCGGGT

GGGGAAGGGCTACTGAAAAGCTGAACTTTTTTAAGAGTTCGAACTTCGAAAGTCAATCAA

TGATTCTTAATTGAGAGGGAAAAGCTATAAAGGACGAGAAGGGAAGGAAATGTTTATAGG

GAATGAATTTCTAATGCATGATGGGAATGACAGATATGAGGAATATATAAAGGTGATTCT

TATATACATTGACATGGATCGGAATCTATTAACTTAAAGTTATTGGGGTCGTGAAATTTA

TTTAATTTTTCTAATTCGTGTCACATGTCACGACAATTAGTGGGG-AACATTTATGTATA

TAGAAATTTCAGAAATTTCTAGCAGCGTGATAAATATAAAGATTTTGGCACAATAAGTTT

TTGGATCATAATGAGTTT-CTATTTAATCAA-GGCA-TGTAA-TCTATTTTATTATTTAG

-TAGGG-AAACTGAAAACTTAGGGTTGCTATTTGTAGCTCCCACCCTTCTTAGTTCTTAC

TCTTTTCAATATTTTTTATAAGTTTTA-TATAATACCT-AAATT-GCC-TCCTCCTCA-C

CCCCCCTGCTAATCTTCTTC-T-CTCACACATTGCTTCT-CACCCCAT-GCTCTTCCCGT

GCTCCTCCATGTG-AGTTCTAGTCTTTTTTTTT-CTCTGGTAGCGTTTTGCTGCTCCTTT

TTTCACTCAAGTGTTGCCAATTAA-TTGACAAAAAATGGTTTCTGTTTCATATAGAAACT

ATGTTTTTGTTGTGTAGTCATACATTACGGAATCTAGTTT-CCATTAAATAAGTAACATG

TGAAAAAAAA--TAAAAGGTGAAATATATATTGTTGGAAAAGAAGCTATGAGGTGCAAGA

ACCGATCACATGGAGAAGGCAATGAAAGACAAGGAGGAGCAATGGAAGA-----------

---------GAGAAAATGAGAAGATGGAAGGGATGTGAAAATGTTTGAAAAAGACGAGGT

GATCAGTTTTGAAGTACGAATTTAGTATTCTCTTTTTAAGAAAATTCTTTCG-AAAGTCG

TGTTTTAAAACATGACTTTTATT-ATTTGAAGTC----TGTTCTAAAACATGACTTA--T

TCATATCCTT-AATATTTTT---------AAAATTTATCCATTTGTAATATTTTTTAAAA

ATTGACCCATATATGTAAAATACCCGTCAAGATCTCTTTATTATTTTGAAAGCGAAAGCA

TATCACTTCAAACACAATGGAATCGAGGCTATTGACTAAGTATAAATAGAGAAGACTTCA

TATCGGGGTTCATAATTCATAACAAAGCAAACGAGTATATAAG----------CCAAATT

TTGAGTAAACTAGTGTGCACACTATCCCATGCCTAGTGGAAGTAGGGATCCTCTCGTTGT

TGGGGGAGTAATTGGGGATGTATTGGATCCTTTTGAATATTCTATTCCTATGAGGGTTAC

CTACAATAACAGAGATGTCAGCAATGGATGTGAATTCAAACCCTCACAAGTTGTCAACCA

ACCAAGGGTAAATATCGGTGGTGATGACC

>CS42.98

TATTAGACTCCTAATTTGATATTCTTGTTTTATTATAATGCTGATAAGTCTTGTAAATAA

GGGTGAGAAGCACGAATAATTAGTTCATGAGATGTGTATAATTATTATCCTACACGACTT

ATCTTTGATATTTCACACAAGTCTTTCAATGTATAACAAAAATTTTTTAGATACATTTAG

ACTATAGAACTAACAAGTTATATTTTGAACC-AAAAAACAAAGAGAGAGAG----GAACA

TAGAGAAAAGAAGATATGAGAGTTTTTTTTTTTTTTTT---CTAAAACAGAAAGAAACTC

ATTATATAATAAACAAATTAATTTGAGACAAGTTAACGTATGTAATAAAACAAAATCAAA

GCGGTAATTAAAATTATTTAATGGTAAACAGTCTAATAGTTAAAATAAAAATGGAAATCA

CATAAATTTTGTAATTGGCCCATTAAAAACAACACTAAGCTTTTAATTTGATTTTGAAAT

TCAAAATAATTTTATTAAATCACAAAGTAAAAGGTTTACAAAGCCGATCATGACAGTGCA

TGTGGGAGGCAAATCGGCATATTTGCACTACAAAAGG-ACCCATGAAGTCTCTGAACATG

CACGCAACACTTTAATCTCTTATTAGTTACTTTGAAAGCTTATTTATATATATAGACACG

CGTAAAAACTTCTTAACCAAGATTTTTTT-ACGTGCTTCC-TTCGCGTTTAATTTGGACC

ATCAAACCGTGCTCAACAGATAAAGAAAAGGGTGCTTTTGATTCAAGATATTGGCC-GAA

AAACACAAGATAGATCCTT-CGATAGATTAAGCCACGCATGAAACGCGAATCCAAAGTGA

TGAAGAAGTGCAGATAGATATTCGTTCACCATATAGAGGAGAATATATCATTCCTACAAA

CAAAATTGATGATGTTTCTAGAATAGAAACGTTAGAATCGAATATTCATCTGTTCCGGGT

GGGGAAGGGCTACTGAAAAGCTGGACTTTTTTAAGAGTTCGAACTTCGAAAGTCAATCAA

TGATTCTTAATTGAGAGGGAAAAGCTATAAAGGACGAGAAGGGAAGGAAATGTTTATAGG

GAATGAATTTCTAATGCATGATGGGAATGACAGATATGAGGAATATATAAAGGTGATTCT

TATATACATTGACATGGATCGGAATCTATTAACTTAAAGTTATTGGGGTCGTGAAATTTA

TTTAATTTTTCTAATTCGTGTCACATGTCACGACAATTAGTGGGG-AACATTTATGTATA

TAGAAATTTCAGAAATTTCTAGCAGCGTGATAAATATAAAGATTTTGGCACAATAAGTTT

TTGGATCATAATGAGTTT-CTATTTAATCAA-GGCA-TGTAA-TCTATTTTATTATTTAG

-TAGGG-AAACTGAAAACTTAGGGTTGCTATTTGTAGCTCCCACCCTTCTTAGTTCTTAC

TCTTTTCAATATTTTTTATAAGTTTTA-TATAATACCT-AAATT-GCCCTCCTCCTCACC

CCCCCCTGCTAATCTTCTTCCTCCTCACACATTGCTTCT-CACCCCATTGCTCTTCCGTT

GCTCCTCCATGTG-AGTTCTAGTCTTTTTTTTT-CTCTGGTAGCGTTTTGCTGCTCCTTT

TTTCACTCAAGTGTTGCCAATTAA-TTGACAAAAAATGGTTTCTGTTTCATATAGAAACT

ATGTTTTTGTTGTGTAGTCATACATTACGGAATCTAGTTT-CCATTAAATAAGTAACATG

TGAAAAAAAA--TAAAAGGTGAAATATATATTGTTGGAAAAGAAGCTATGAGGTGCAAGA

ACCGATCACATGGAGAAGGCAATGAAAGACAAGGAGGAGCAATGGAAGA-----------

---------GAGAAAATGAGAAGATGGAAGGGATGTGAAAATGTTTGAAAAAGACGAGGT

GATCAGTTTTGAAATACGAATTTAGTATTTTCTTTTTAAGAAAATTCTTTCG-AAAGTCG

TGTTTTAAAACATGACTTTTATT-ATTTGAAGTC----TGTTCTAAAACATGACCTA--T

TCATATCCTT-AATATTTTT---------AAAATTTATCCATTTGTAATATTTTTTAAAA

ATTGACCCATATATGTAAAATACCCGTCAAGATCTCTTTATTATTTTGAAAGCGAAAGCA

TATCACTTCAAACACAATGGAATCGAGGCTATTGACTAAGTATAAATAGAGAAGACTTCA

TATCGGGGTTCATAATTCATAACAAAGCAAACGAGTATATAAG----------CCAAATT

TTGAGTAAACTAGTGTGCACACTATCCCATGCCTAGTGGAAGTAGGGATCCTCTCGTTGT

TGGGGGAGTAATTGGGGATGTATTGGATCCTTTTGAATATTCTATTCCTATGAGGGTTAC

CTACAATAACAGAGATGTCAGCAATGGATGTGAATTCAAACCCTCACAAGTTGTCAACCA

ACCAAGGGTAAATATCGGTGGTGATGACC

>CS41.88

TATTAGACTCCTAATTTGACATTCTTGTTTTATTATAATGCTGATAAGTCTTGTAAATAA

GGGTGAGAAGCACGAATAATTAGTCCATGAGATGTGTATAATTATTATCCTACACGACTT

ATCTTTGATATTTCACACAAGTCTTTCAATGTATAACAAAAATTTTTTAGATACATTTAG

ACTATAGAACTAACAAGTTATATTTTGAACC-AAAAAACAAAGAGAGAGAG----GAACA

TAGAGAAAAGAAGATATGAGGGTTTTTTTTTTTTTTT----CTAAAACAGAAAGAAACTC

ATTATATAATAAACAAATTAATTTGAGACAAATTAACGTATGTAATAAAACAAAATCAAA

GTGGTAATTAAAATTATTTAATGGTAAACAGTCTAATAGTTAAAATAAAAATGGAAATCA

CATAAATTTTGTAATTGGCCCATTAAAAACGACACTAAGCTTTTAATTTGATTTTGAAAT

TCAAAATAATTTTATTAAATCACAAAGTAAAAGGTTTACAAAGCCGATCATGACAGTGCA

TGTGGGAGGCAAATCGGCATATTTGCACTACAAAAGG-ACCCATGAAGTCTCTGAACATG

CACGCAACACTTTAATCTCTTATTAGTTACTTTGAAAGCTTATTTATATATATAGACACG

CGTAAAAACTTCTTAACCAAGATTTTTTT-ACGTGCTTCC-TTCGCGTTTAATTTGGACC

ATCAAACCGTGCTCAACAGATAAAGAAAAGGGTGCTTTTGATTCAAGATATTGGCC-GAA

AAACACAAGATAGATCCTT-CGATAGATTAAGCCACGCATGAAACGCGAATCCAAAGTGA

TGAAGAAGTGCAGATAGATATTCGTTCACCATATAGAGGAGAATATATCATTCCTACAAA

CAAAATTGATGATGTTTCTAGAATAGAAACGTTAGAATCGAATATTCATCTGTTCCGGGT

GGGGAAGGGCTACTGAAAAGCTGAACTTTTTTAAGAGTTCGAACTTCGAAAGTCAATCAA

TGATTCTTAATTGAGAGGGAAAAGCTATAAAGGACGAGAAGGGAAGGAAATGTTTATAGG

GAATGAATTTCTAATGCATGATGGGAATGACAGATATGAGGAATATATAAAGGTGATTCT

TATATACATTGACATGGATCGGAATCTATTAACTTAAAGTTATTGGGGTCGTGAAATTTA

TTTAATTTTTCTAATTCGTGTCACATGTCACGACAATTAGTGGGG-AACATTTATGTATA

TAGAAATTTCAGAAATTTCTAGCAGCGTGATAAATATAAAGATTTTGGCACAATAAGTTT

TTGGATCATAATGAGTTT-CTATTTAATCAA-GGCA-TGTAA-TCTATTTTATTATTTAG

-TAGGG-AAACTGAAAACTTAGGGTTGCTATTTGTAGCTCCCACCCTTCTTAGTTCTTAC

TCTTTTCAATATTTTTTATAAGTTTTA-TATAATACCT-AAATT-GCCCTCCTCCTCACC

CCCCCCTGCTAATCTTCTTCCTCCTCACACATTGCTTCT-CACCCCATTGCTCTTCCGTT

GCTCCTCCATGTG-AGTTCTAGTCTTTTTTTT--CTCTGGTAGCGTTTTGCTGCTCCTTT

TTTCACTCAAGTGTTGCCAATTAA-TTGACAAAAAATGGTTTCTGTTTCATATAGAAACT

ATGTTTTTGTTGTGTAGTCATACATTACGGAATCTAGTTT-CCATTAAATAAGTAACATG

TGAAAAAAAA--TAAAAGGTGAAATATATATTGTTGGAAAAGAAGCTATGAGGTGCAAGA

ACCGATCACATGGAGAAGGCAATGAAAGACAAGGAGGAGCAATGGAAGA-----------

---------GAGAAAATGAGAAGATGGAAGGGATGTGAAAATGTTTGAAAAAGACGAGGT

GACCAGTTTTGAAATACGAATTTAGTATTTTCTTTTTAAGAAAATTCTTTCG-AAAGTCG

TGTTTTAAAACATGACTTTTATT-ATTTGAAGTC----TGTTCTAAAACATGACTTA--T

TCATATCCTT-AATATTTTT---------AAAATTTATCCATTTGTAATATTTTTTAAAA

ATTGACCCATATATGTAAAATACCCGTCAAGATCTCTTTATTATTTTGAAAGCGAAAGCA

TATCACTTCAAACACAATGGAATCGAGGCTATTGACTAAGTATAAATAGAGAAGACTTCA

TATCGGGGTTCATAATTCATAACAAAGCAAACGAGTATATAAG----------CCAAATT

TTGAGTAAACTAGTGTGCACACTATCCCATGCCTAGCGGAAGTAGGGATCCTCTCGTTGT

TGGGGGAGTAATTGGGGATGTATTGGATCCTTTTGATTATTCTATTCCTATGAGGGTTAC

CTACAATAACAGAGATGTCAGCAATGGATGTGAATTCAAACCCTCACAAGTTGTCAACCA

ACCAAGGGTAAATATCGGTGGTGATGACC

>CS51.G8

TATTAGACTCCTAATTTGATATTCTTGTTTTATTATAATGCTGATAAGTCTTGTAAATAA

GGGTGAGAAGCACGAATAATTAGTTCATGAGATGTGTATAATTATTATCCTACACGACTT

ATCTTTGATATTTCACACAAGTCTTTCAATGTATAACAAAAATTTTTTAGATACATTTAG

ACTATAGAACTAACAAGTTATATTTTGAACC-GAAAAACAAAGAGAGGGAG----GAACA

TAGAGAAAAGAAGATATGAGAGTTTTTTTTTTTTTTTT---CTAAAACAGAAGGAAACTC

ATTATATAATAAACAAATTAATTTGAGACAAATTAACGTATGTAATAAAACAAAATCAAA

GTGGTAATTAAAATTATTTAATGGTAAACAGTCTAATAGTTAAAATAAAAATGGAAATCA

CATAAATTTTGTAGTTGGCCCATTAAAAACAACACTAAGCTTTTAATTTGATTTTGAAAT

TCAAAATAATTTTATTAAATCACAAAGTAAAAGGTTTACAAAGCCGATCATGACAGTGCA

TGTGGGAGGCAAATCGGCATATTTGCACTACAAAAGG-ACCCATGAAGTCTCTGAACATG

CACGCAACACTTTAATCTCTTATTAGTTACTTTGAAAGCTTATTTATATATATAGACACG

CGTAAAAACTTCTTAACCAAGATTTTTTT-ACGTGCTTCC-TTCGCGTTTAATTTGGACC

ATCAAACCGTGCTCAACAGATAAAGAAAAGGGTGCTTTTGATTCAAGATATTGGCC-GAA

AAACACAAGATAGATCCTT-CGATAGATTAAGCCACGCATGAAACGCGAATCCAAAGTGA

TGAAGAAGTGCAGATAGATATTCGTTCACCATATAGAGGAGAATATATCATTCCTACAAA

CAAAATTGATGATGTTTCTAGAATAGAAACGTTAGAATCGAATATTCATCTGTTCCGGGT

GGGGAAGGGCTACTGAAAAGCTGAACTTTTTTAAGAGTTCGAACTTCGAAAGTCAATCAA

TGATTCTTAATTGAGAGGGAAAAGCTATAAAGGACGAGAAGGGAAGGAAATGTTTATAGG

GAATGAATTTCTAATGCATGATGGGAATGACAGATATGAGGAATATATAAAGGTGATTCT

TATATACATTGACATGGATCGGAATCTATTAACTTAAAGTTATTGGGGTCGTGAAATTTA

TTTAATTTTTCTAATTCGTGTCACATGTCACGACAATTAGTGGGG-AACATTTATGTATA

TAGAAATTTCAGAAATTTCTAGCAGCGTGATAAATATAAAGATTTTGGCACAATAAGTTT

TTGGATCATAATGAGTTT-CTATTTAATCAA-GGCA-TGTAA-TCTATTTTATTATTTAG

-TAGGG-AAACTGAAAACTTAGGGTTGCTATTTGTAGCTCCCACCCTTCTTAGTTCTCAC

TCTTTTCAATATTTTTTATAAGTTTTA-TATAATACCT-AAATT-GCCCTCCTCCTCA-C

CCCCCCTGCTAATCTTCTTCCTCCTCACACATTGCTTCT-CACCCCATTGCTCTTCCGTT

GCTCCTCCATGTG-AGTTCTAGTCTTTTTTTT--CTCTGGTAGCGTTTTGCTGCTCCTTT

TTTCACTCAAGTGTTGCCAATTAA-TTGACAAAAAATGGTTTCTGTTTCATATAGAAACT

ATGTTTTTGTTGTGTAGTCATACATTACGGAATCTGGTTT-CCATTAAATAAGTACCATG

TGAAAAAAAA--TAAAAGGTGAAATATATATTGTTGGAAAAGAAGCTATGAGGTGCAAGA

ACCGATCACATGGAGAAGGCAATGAAAGACAAGGAGGAGCAATGGAAGA-----------

---------GAGAAAATGAGAAGATGGAAGGGATGTGAAAATGTTTGAAAAAGACGAGGT

GATCAGTTTTGAAATACGAATTTAGTATTTTCTTTTTAAGAAAATTCTTTCG-AAAGTCG

TGTTTTAAAACATGACTTTTATT-ATTTGAAGTC----TGTTCTAAAACATGACTTA--T

TCATATCCTT-AATATTTTT---------AAAATTTATCCATTTGTAATATTTTTTAAAA

ATTGACCCATATATGTAAAATACCCGTCAAGATCTCTTTATTATTTTGAAAGCGAAAGCA

TATCACTTCAAACACAATGGAATCGAGGCTATTGACTAAGTATAAATAGAGAAGACTTCA

TATCGGGGTTCATAATTCATAACAAAGCAAACGAGTATATAAG----------CCAAATT

TTGAGTAAACTAGTGTGCACACTATCCCATGCCTAGTGGAAGTAGGGATCCTCTCGTTGT

TGGGGGAGTAATTGGGGATGTATTGGATCCTTTTGAATATTCTATTCCTATGAGGGTTAC

CTACAATAACAGAGATGTCAGCAATGGATGTGAATTCAAACCCTCACAAGTTGTCAACCA

ACCAAGGGTAAATATCGGTGGTGATGACC

>H12.C3

TATTAGACTCCTAATTTAATATTCTTGTTTTATTGTAATGCTGATAAGTCTTGTAAATAA

GGGTGAGAAGCACGAATAATTAGTTCATGAGATGTGTATAATTATTATCCTACACGACTT

ATCTTTGATATTTCACACAAGTCTTTCAATGTATAACAAAAACTTTTTAGATACATTTAG

ACTATAGAACTAACAAGTTATATTTTGAACC-AAAAAACAAAGAGAGAGAG----GAACA

TAGAGAAAAGAAGATATGAGAGTTTTTTTTTT---------CTAAAACAGAAAGAAACTC

ATTATATAATAAACAAATTACTTTGAGACAAATTAACGTATGTAATAAAACAAAATCAAA

GTGGTAATTAAAATTATTTAATGGTAAACAGTCTAATAGTTAAAATAAAAATGGAAATCA

CATAAATTTTGTAATTGGCCCATTAAAAACAACACTAAGCTTTTAATTTGATTTTGAAAT

TCAAGATAATTTTATTAAATCACAAAGTAAAAGGTTTACAAAGCCGATCATGACAGTGCA

TGTGGGAGGCAAATCGGCATATTTGCACTACAAAAGG-ACCCATGAAGTCTCTGAACATG

CACGCAACACTTTAATCTCTTATTAGTTACTTTGAAAGCTTATTTATATATATAGACACG

CGTAAAAACTTCTTAACCAAGATTTTTTT-ACGTGCTTCC-TTCGCGTTTAATTTGGACC

ATCAAACCGTGCTCAACAGATAAAGAAAAGGGTGCTTTTGATTCAAGATATTGGCC-GAA

AAACACAAGATAGATCCTT-CGATAGATTAAGCCACGCATGAAACGCGAATCCAAAGTGA

TGAAGAAGTGCAGATAGATATTCGTTCACCATATAGAGGAGAATATATCATTCCTACAAA

CAAAATTGATGATGTTTCTAGAATAGAAACGTTAGAATCGAATATTCATCTGTTCCGGGT

GGGGAAGGGCTACTGAAAAGCTGAACTTTTTTAAGAGTTCGAACTTCGAAAGTCAATCAA

TGATCCTTAATTGAGAGGGAAAAGCTATAAAGGACGAGAAGGGAAGGAAATGTTTATAGG

GAATGAATTTCTAATGCATGATGGGAATGACAGATATGAGGAATATATAAAGGTGATTCT

TATATACATCGACATGGATCGGAATCTATTAACTTAAAGTTATTGGGGTCGTGAAATTTA

TTTAATTTTTCTAATTCGTGTCACATGTCACGACAATTAGTGGGG-AACATTTATGTATA

TAGAAATTTCAGAAATTTCTAGCAGCGTGATAAATATAAAGATTTTGGCACAATAAGTTT

TTGGATCATAATGAGTTT-CTATTTAATCAA-GGCA-TGTAA-TCTATTTTATTATTTAG

-TAGGG-AAACTGAAAACTTAGGGTTGCTATTTGTAGCTCCCACCCTTCTTAGTTCTTAC

TCTTTTCAATATTTTTTATAAGTTTTA-TATAATACCT-AAATT-GCCCTCCTCCTCA-C

CCCCCCTGCTAATCTTCTTCCTCCTCACACATTGCTTCT-CACCCCATTGCTCTTCCGTT

GCTCCTCCATGTG-AGTTCTAGTCTTTTTTTT--CTCTGGTAGCGTTTTGCTGCACCTTT

TTTCACTCAAGTGTTGCCAATTAA-TTGACAAAAAATGGTTTCTGTTTCATATAGAAACT

ATGTTTTTGTTGTGTAGTCATACATTACGGAATCTAGTTT-CCATTAAATAAGTAACATG

TGAAAAAAAA--TAAAAGGTGAAATATATATTGTTGGAAAAGAAGCTATGAGGTGCAAGA

ACCGATCACATGGAGAAGGCAATGAAAGACAAGGAGGAGCAATGAAAGACAAGGAGGAGC

AATGAAAGAGAGAAAATGAGAAGATGGAAGGGATGTGAAAATGTTTGAAAAAGACGAGGT

GATCAGTTTTGAAATACGAATTTAATATTTTCTTTTTAAGAAAATTCTTTCG-AAAGTCG

TGTTTTAAAACATGACTTTTATT-ATTTGAAGTCG---TGTTCTAAAACATGACTTA--T

TCATATCCTT-AATATTTTT---------AAAATTTATCCATTTGTAATATTTTTTAAAA

ATTGACCCATATATGTAAAATACCCGTCAAGATCTCTTTATTATTTTGAAAGCGAAAGCA

TATCACTTCAAACACAATGGAATCGAGGCTATTGACTAAGTATAAATAGAGAAGACTTCA

TATCGGGGTTCATAATTCATAACAAAGCAAACGAGTATATAAGAAAGCATAAGCCAAATT

TTGAGTAAACTAGTGTGCACACTATCCCATGCCTAGTGGAAGTAGGGATCCTCTCGTTGT

TGGGGGAGTAATTGGGGATGTATTGGATCCTTTTGAATATTCTATTCCTATGAGGGTTAC

CTACAATAACAGAGATGTCAGCAATGGATGTGAATTCAAACCCTCACAAGTTGTCAGCCA

ACCAAGGGTAAATATCGGTGGTGATGACC

>H09.04

TATTAGACTCCTAATTTAATATTCTTGTTTTATTATAATGCTGATAAGTCTTGTAAATAA

GGGTGAGAAGCACGAATAATTAGTTCATGAGATGTGTATAATTATTATCCTACACGACTT

ATCTTTGATATTTCACACAAGTCTTTCAATGAATAACAAAAACTTTTTAGATACATTTAG

ACTATAGAACTAACAAGTTATATTTTGAACC-AAAAAACAAAGAGAGAGAG----GAACA

TAGAGAAAAGAAGATATGAGAGTTTTTTTTTT---------CTAAAACAGAAAGAAACTC

ATTATATAATAAACAAATTACTTTGAGACAAATTAACGTATGTAATAAAACAAAATCAAA

GTGGTAATTAAAATTATTTAATGGTAAACAGTCTAATAGTTAAAATAAAAATGGAAATCA

CATAAATTTTGTAATTGGCCCATTAAAAACAACACTAAGCTTTTAATTTGATTTTGAAAT

TCAAAATAATTTTATTAAATCACAAAGTAAAAGGTTTACAAAGCCGATCATGACAGTGCA

TGTGGGAGGCAAATCGGCATATTTGCACTACAGAAGG-ACCCATGAAGTCTCTGAACATG

CACGCAACACTTTAATCTCTTATTAGTTACTTTGAAAGCTTATTTATATATATAGACACG

CGTAAAAACTTCTTAACCAAGATTTTTTT-ACGTGCTTCC-TTCGCGTTTAATTTGGACC

ATCAAACCGTGCTCAACAGATAAAGAAAAGGGTGCTTTTGATTCAAGATATTGGCCCGAA

AAACACAAGATAGATCCTT-CGATAGATTAAGCCACGCATGAAACGCGAATCCAAAGTGA

TGAAGAAGTGCAGATAGATATTCGTTCACCATATAGAGGAGAATATATCATTCCTACAAA

CAAAATTGATGATGTTTCTAGAATAGAAACGTTAGAATCGAATATTCATCTGTTCCGGGT

GGGGAAGGGCTACTGAAAAGCTGAACTTTTTTAAGAGTTCGAACTTCGAAAGTCAATCAA

TGATTCTTAATTGAGAGGGAAAAGCTATAAAGGACGAGAAGGGAAGGAAATGTTTATAGG

GAATGAATTTCTAATGCATGATGGGAATGACAGATATGAGGAATATATAAAGGTGATTCT

TATATACATCGACATGGATCGGAATCTATTAACTTAAAGTTATTGGGGTCGTGAAATTTA

TTTAATTTTTCTAATTCGTGTCACATGTCACGACAATTAGTGGGG-AACATTTATGTATA

TAGGAATTTCAGAAATTTCTAGCAGCGTGATAAATATAAAGATTTTGGCACAATAAGTTT

TTGGATCATAATGAGTTT-CTATTTAATCAA-GGCA-TGTAA-TCTATTTTATTATTTAG

-TAGGG-AAACTGAAAACTTGGGGTTGCTATTTGTAGCTCCCACCCTTCTTAGTTCTTAC

TCCTTTCAATATTTTTTAAAAGTTTTA-TATAATACCT-AAATT-GCCCTCCTCCT----

----------------------------------------CACCCCATTTCTCTTCCGTT

GCTCCTCCATGTG-AGTTCTTGTCTTTTTTTT--CTCTGGTAGCGTTTTGCTGCTCCTTT

TTTCACTCAAGTGTTGCCAATTAA-TTGACAAAAAATGGTTTCTGTTTCATATAGAAACT

ATGTTTTTGTTGTGTAGTCATACATCACGGAATCTAGTTT-CCATTAAATAAGTAACATG

TGAAAAAAAAAATAAAAGGTGAAATATATATTGTTGGAAAAGAAGCTATGAGGTGCAAGA

ACCGATCACATGGAGAAGGCAATGAAAGACAAGGAGGAGCAATGGAAGA-----------

---------GAGAAAATGAGAAGATGGAAGGGATGTGAAAATGTTTGAAAAAGACGAGGG

GATCAGTTTTGAAATACGAATTTAGTATTTTTTTTTTAAGAAAATTCTTTCG-AAAGTCG

TGTTTTAAAACATGACTTTTATT-ATTTGAAGTCG---TGTTCTAAAACATGACTTA--T

TCATATCCTT-AATATTTTT---------AAAATTTATCCATTTGTAATATTTTTTAAAA

ATTGACCCATATATGTAAAATACCCGTCAAGATCTCTTTATTATTTTGAAAGCGAAAGCA

TATCACTTCAAACACGATGGAATCGAGGCTATTGACTAAGTATAAATAGGGAAGACTTCA

TATCGGGGTTCATAATTCATAACAAAGCAAACGAGTATATAAGAAAGCATAAGCCAAATT

TTGAGTAAACTAGTGTGCACACTATCCCATGCCTAGTGGAAGTAGGGATCCTCTCGTTGT

TGGGGGAGTAATTGGGGATGTATTGGATCCTTTTGAATATTCTATTCCTATGAGGGTTAC

CTACAATAACAGAGATGTCAGCAATGGATGTGAATTCAAACCCTCACAAGATGTCAACCA

ACCAAGGGTAAATATCGGTGGTGATGACC

>HH.ALL

TATTAGACTCCTAATTTAATATTCTTGTTTTATTATAATGCTGATAAGTCTTGTAAATAA

GGGTGAGAAGCACGAATAATTAGTTCATGAGATGTGTATAATTATTATCCTACACGACTT

ATCTTTGATATTTCACACAAGTCTTTCAATGTATAACAAAAACTTTTTAGATACATTTAG

ACTATAGAACTAACAAGTTATATTTTGAACC-AAAAAACAAAGAGAGAGAGAGAGGAACA

TAGAGAAAAGAAGATATGAGAGTTTTTTTTTTTTTTTTT--CTAAAACAGAAAGAAACTC

ATTATATAATAAACAAATTACTTTGAGACAAATTAACGTATGTAATAAAACAAAATCAAA

GTGGTAATTAAAATTATTTAATGGTAAACAGTCTAATAGTTAAAATAAAAATGGAAATCA

CATAAATTTTGTAATTGGCCCATTAAAAACAACACTAAGCTTTTAATTTGATTTTGAAAT

TCAAAATAATTTTATTAAATCACAAAGTAAAAGGTTTACAAAGCCGATCATGACAGTGCA

TGTGGGAGGCAAATCGGCATATTTGCACTACAAAAGG-ACCCATGAAGTCTCTGAACATG

CACGCAACACTTTAATCTCTTATTAGTTACTTTGAAAGCTTATTTATATATATAGACACG

CGTAAAAACTTCTTAACCAAGATTTTTTT-ACGTGCTTCC-TTCGCGTTTAATTTGGACC

ATCAAACCGTGCTCAACAGATAAAGAAAAGGGTGCTTTTGATTCAAGATATTGGCC-GAA

AAACACAAGATAGATCCTT-CGATAGATTAAGCCACGCATGAAACGCGAATCCAAAGTGA

TGAAGAAGTGCAGATAGATATTCGTTCACCATATAGAGGAGAATATATCATTCCTACAAA

CAAAATTGATGATGTTTCTAGAATAGAAACGTTAGAATCGAATATTCATCTGTTCCGGGT

GGGGAAGGGCTACTGAAAAGCTGAACTTTTTTAAGAGTTCGAACTTCGAAAGTCAATCAA

TGATTCTTAATTGAGAGGGAAAAGCTATAAAGGACGAGAAGGGAAGGAAATGTTTATAGG

GAATGAATTTCTAATGCATGATGGGAATGACAGATATGAGGAATATATAAAGGTGATTCT

TATATACATTGACATGGATCGGAATCTATTAACTTAAAGTTATTGGGGTCGTGAAATTTA

TTTAATTTTTCTAATTCGTGTCACATGTCACGACAATTAGTGGGG-AACATTTATGTATA

TAGAAATTTCAGAAATTTCTAGCAGCGTGATAAATATAAAGATTTTGGCACAATAAGTTT

TTGGATCATAATGAGTTT-CTATTTAATCAA-GGCA-TGTAA-TCTATTTTATTATTTAG

-TAGGG-AAACTGAAAACTTAGGGTTGCTATTTGTAGCTCCCACCCTTCTTAGTTCTTAC

TCTTTTCAATATTTTTTAAAAGTTTTA-TATAATACCT-AAATT-GCCCTCCTCCT----

----------------------------------------CACCCCATTGCTCTTCCGTT

GCTCCTCCATGTG-AGTTCTTGTCTTTTTTTTT-CTCTGGTAGCGTTTTGCTGCTCCTTT

TTTCACTCAAGTGTTGCCAATTAA-TTGACAAAAAATGGTTTCTGTTTCATATAGAAACT

ATGTTTTTGTTGTGTAGTCATACATTACGGAATCTAGTTT-CCATTAAATAAGTAAC--G

TGAAAAAAAA--TAAAAGGTGAAATATATATTGTTGGAAAAGAAGCTATGAGGTGCAAGA

ACCGATCACATGGAGAAGGCAATGAAAGACAAGGAGGAGCAATGGAAGA-----------

---------GAGAAAATGAGAAGATGGAAGGGATGTGAAAATGTTTGAAAAAAACGAGGT

GATCAGTTTTAAAATACGAATTTAGTATTTTCTTTTTAAGAAAATTCTTTCG-AAAGTCG

TGTTTTAAAACATGACTTTTATT-ATTTGAAGTCG---TGTTCTAAAACATGACTTA--T

TCATATCCTT-AATATTTTT---------AAAATTTATCCATTTGTAATATTTTTTAAAA

ATTGACCCATATATGTAAAATACCCGTCAAGATCTCTTTATTATTTTGAAAGCGAAAGCA

TATCACTTCAAACACAATGGAATCGAGGCTATTGACTAAGTATAAATAGAGAAGACTTCA

TATCGGGGTTCATAATTCATAACAAAGCAAACGAGTATATAAGAAAGCATAAGCCAAATT

TTGAGTAAACTAGTGTGCACACTATCCCATGCCTAGTGGAAGTAGGGATCCTCTCGTTGT

TGGGGGAGTAATTGGGGATGTATTGGATCCTTTTGAATATTCTATTCCTATGAGGGTTAC

CTACAATAACAGAGATGTCAGCAATGGATGTGAATTCAAACCCTCACAAGTTGTCAACCA

ACCAAGGGTAAATATCGGTGGTGATGACC

>CS03.12

TATTAGACTCCTAATTTAATATTCTTGTTTTATTATAATGCTGATAAGTCTTGTAAATAA

GGGTGAGAAGCACGAATAATTAGTTCATGAGATGTGTATAATTATTATCCTACACGACTT

ATCTTTGATATTTCACACAAGTCTTTCAATGTATAACAAAAACTTTTTAGATACATTTAG

ACTATAGAACTAACAAGTTATATTTTGAACC-AAAAAACAAAGAGAGAGAGAGAGGAACA

TAGAGAAAAGAAGATATGAGAGTTTTTTTTTTTTTTTT---CTAAAACAGAAAGAAACTC

ATTATATAATAAACAAATTACTTTGAGACAAATTAACGTATGTAATAAAACAAAATCAAA

GTGGTAATTAAAATTATTTAATGGTAAACAGTCTAATAGTTAAAATAAAAATGGAAATCA

CATAAATTTTGTAATTGGCCCATTAAAAACAACACTAAGCTTTTAATTTGATTTTGAAAT

TCAAAATAATTTTATTAAATCACAAAGTAAAAGGTTTACAAAGCCGATCATGACAGTGCA

TGTGGGAGGCAAATCGGCATATTTGCACTACAAAAGG-ACCCATGAAGTCTCTGAACATG

CACGCAACACTTTAATCTCTTATTAGTTACTTTGAAAGCTTATTTATATATATAGACACG

CGTAAAAACTTCTTAACCAAGATTTTTTT-ACGTGCTTCC-TTCGCGTTTAATTTGGACC

ATCAAACCGTGCTCAACAGATAAAGAAAAGGGTGCTTTTGATTCAAGATATTGGCC-GAA

AAACACAAGATAGATCCTT-CGATAGATTAAGCCACGCATGAAACGCGAATCCAAAGTGA

TGAAGAAGTGCAGATAGATATTCGTTCACCATATAGAGGAGAATATATCATTCCTACAAA

CAAAATTGATGATGTTTCTAGAATAGAAACGTTAGAATCGAATATTCATCTGTTCCGGGT

GGGGAAGGGCTACTGAAAAGCTGAACTTTTTTAAGAGTTCGAACTTCGAAAGTCAATCAA

TGATTCTTAATTGAGAGGGAAAAGCTATAAAGGACGAGAAGGGAAGGAAATGTTTATAGG

GAATGAATTTCTAATGCATGATGGGAATGACAGATATGAGGAATATATAAAGGTGATTCT

TATATACATTGACATGGATCGGAATCTATTAACTTAAAGTTATTGGGGTCGTGAAATTTA

TTTAATTTTTCTAATTCGTGTCACATGTCACGACAATTAGTGGGG-AACATTTATGTATA

TAGAAATTTCAGAAATTTCTAGCAGCGTGATAAATATAAAGATTTTGGCACAATAAGTTT

TTGGATCATAATGAGTTT-CTATTTAATCAA-GGCA-TGTAA-TCTATTTTATTATTTAG

-TAGGG-AAACTGAAAACTTAGGGTTGCTATTTGTAGCTCCCACCCTTCTTAGTTCTTAC

TCTTTTCAATATTTTTTAAAAGTTTTA-TATAATACCT-AAATT-GCCCTCCTCCT----

----------------------------------------CACCCCATTGCTCTTCCGTT

GCTCCTCCATGTG-AGTTCTTGTCTTTTTTTTT-CTCTGGTAGCGTTTTGCTGCTCCTTT

TTTCACTCAAGTGTTGCCAATTAA-TTGACAAAAAATGGTTTCTGTTTCATATAGAAACT

ATGTTTTTGTTGTGTAGTCATACATTACGGAATCTAGTTT-CCATTAAATAAGTAAC--G

TGAAAAAAAA--TAAAAGGTGAAATATATATTGTTGGAAAAGAAGCTATGAGGTGCAAGA

ACCGATCACATGGAGAAGGCAATGAAAGACAAGGAGGAGCAATGGAAGA-----------

---------GAGAAAATGAGAAGATGGAAGGGATGTGAAAATGTTTGAAAAAAACGAGGT

GATCAGTTTTAAAATACGAATTTAGTATTTTCTTTTTAAGAAAATTCTTTCG-AAAGTCG

TGTTTTAAAACATGACTTTTATT-ATTTGAAGTCG---TGTTCTAAAACATGACTTA--T

TCATATCCTT-AATATTTTT---------AAAATTTATCCATTTGTAATATTTTTTAAAA

ATTGACCCATATATGTAAAATACCCGTCAAGATCTCTTTATTATTTTGAAAGCGAAAGCA

TATCACTTCAAACACAATGGAATCGAGGCTATTGACTAAGTATAAATAGAGAAGACTTCA

TATCGGGGTTCATAATTCATAACAAAGCAAACGAGTATATAAGAAAGCATAAGCCAAATT

TTGAGTAAACTAGTGTGCACACTATCCCATGCCTAGTGGAAGTAGGGATCCTCTCGTTGT

TGGGGGAGTAATTGGGGATGTATTGGATCCTTTTGAATATTCTATTCCTATGAGGGTTAC

CTACAATAACAGAGATGTCAGCAATGGATGTGAATTCAAACCCTCACAAGTTGTCAACCA

ACCAAGGGTAAATATCGGTGGTGATGACC

>CS32.08

TATTAGACTCCTAATTTAATATTCTTGCTTTATTATAATGCTGATAAGTCTTGTAAATAA

GGGTGAGAAGCACGAGTAATTAGTTCATGAGATGTGTATAATTATTATCCTACACGACTT

ATCTTTGATATTTCACACAAGTCTTTCAATGTATAACAAAAACTTTTTAGATACATTTAG

ACTATAGAACTAACAAGTTATATTTTGAACC-AAAAAACAAAGAGAGAGAGAGAGGAACA

TAGAGAAAAGAAGATATGAGAGTTTTTTTTTTTTTT-----CTAAAACAGAAAGAAACTC

ATTATATAATAAACAAATTACTTTGAGACAAATTAACGTATGTAATAAAACAAAATCAAA

GTGGTAATTAAAATTATTTAATGGTAAACAGTCTAGTAGTTAAAATAAAAATGGAAATCA

CATAAATTTTGTAATTGGCCCATTAAAAACAACACTAAGCTTTTAATTTGATTTTGAAAT

TCAAAATAATTTTATTAAATCACAAAGTAAAAGGTTTACAAAGCCGATCATGACAGTGCA

TGTGGGAGGCAAATCGGCATATTTGCACTACAAAAGG-ACCCATGAAGTCTCTGAACATG

CACGCAACACTTTAATCTCTTATTAGTTACTTTGAAAGCTTATTTATATATATAGACACG

CGTAAAAACTTCTTAACCAAGATTTTTTT-ACGTGCTTCC-TTCGCGTTTAATTTGGACC

ATCAAACCGTGCTCAACAGATAAAGAAAAGGGTGCTTTTGATTCAAGATATTGGCC-GAA

AAACACAAGATAGATCCTT-CGATAGATTAAGCCACGCATGAAACGCGAATCCAAAGTGA

TGAAGAAGTGCAGATAGATATTCGTTCACCATATAGAGGAGAATATATCATTCCTACAAA

CAAAATTGATGATGTTTCTAGAATAGAAACGTTAGAATCGAATATTCATCTGTTCCGGGT

GGGGAAGGGCTACTGAAAAGCTGAACTTTTTTAAGAGTTCGAACTTCGAAAGTCAATCAA

TGATTCTTAATTGAGAGGGAAAAGCTATAAAGGACGAGAAGGGAAGGAAATGTTTATAGG

GAATGAATTTCTAATGCATGATGGGAATGACAGATATGAGGAATATATAAAGGTGATTCT

TATATACATTGACATGGATCGGAATCTATTAACTTAAAGTTATTGGGGTCGTGAAATTTA

TTTAATTTTTCTAATTCGTGTCACATGTCACGACAATTAGTGGGG-AACATTTATGTATA

TAGAAATTTCAGAAATTTCTAGCAGCGTGATAAATATAAAGATTTTGGCACAATAAGTTT

TTGGATCATAATGAGTTT-CTATTTAATCAA-GGCA-TGTAA-TCTATTTTATTATTTAG

-TAGGG-AAACTGAAAACTTAGGGTTGCTATTTGTAGCTCCCACCCTTCTTAGTTCTTAC

TCTTTTCAATATTTTTTAAAAGTTTTA-TATAATACCT-AAATT-GCCCTCCTCCT----

----------------------------------------CACCCCATTGCTCTTCCGTT

GCTCCTCCATGTG-AGTTCTTGTCTTTTTTTTT-CTCTGGTAGCGTTTTGCTGCTCCTTT

TTTCACTCAAGTGTTGCCAATTAA-TTGACAAAAAATGGTTTCTGTTTCATATAGAAACT

ATGTTTTTGTTGTGTAGTCATACATTACGGAATCTAGTTT-CCATTAAATAAGTAAC--G

TGAAAAAAAA--TAAAAGGTGAAATATATATTGTTGGAAAAGAAGCTATGAGGTGCAAGA

ACCGATCACATGGAGAAGGCAATGAAAGACAAGGAGGAGCAATGGAAGA-----------

---------GAGAAAATGAGAAGATGGAAGGGATGTGAAAATGTTTGAAAAAAACGAGGT

GATCAGTTTTAAAATACGAATTTAGTATTTTCTTTTTAAGAAAATTCTTTCG-AAAGTCG

TGTTTTAAAACATGACTTTTATT-ATTTGAAGTCG---TGTTCTAAAACATGACTTA--T

TCATATCCTT-AATATTTTT---------AAAATTTATCCATTTGTAATATTTTTTAAAA

ATTGACCCATATATGTAAAATACCCGTCAAGATCTCTTTATTATTTTGAAAGCGAAAGCA

TATCACTTCAAACACAATGGAATCGAGGCTATTGACTAAGTATAAATAGAGAAGACTTCA

TATCGGGGTTCATAATTCATAACAAAGCAAACGAGTATATAAGAAAGCATAAGCCAAATT

TTGAGTAAACTAGTGTGCACACTATCCCATGCCTAGTGGAAGTAGGGATCCTCTCGTTGT

TGGGGGAGTAATTGGGGATGTATTGGATCCTTTTGAATATTCTATTCCTATGAGGGTTAC

CTACAATAACAGAGATGTCAGCAATGGATGTGAATTCAAACCCTCACAAGTTGTCAACCA

ACCAAGGGTAAATATCGGTGGTGATGACC

>CS43.A8

TATTAGACTCCTAGTTTAATATTCTTGTTTTATTATAATGCTGATAAGTCTTGTAAATAA

GGGTGAGAAGCACGAATAATTAGTTCATGAGATGTGTATAATTATTATCCTACACGACTT

ATCTTTGATATTTCACACAAGTCTTTCAATGTATAACAAAAACTTTTTAGATACATTTAG

ACTATAGAACTAACAAGTTATATTTTGAACC-AAAAAACAAAGAGAGAGAGAGAGGAACA

TAGAGAAAAGAAGATATGAGAGTTTTTTTTTTTTTTTT---CTAAAACAGAAAGAAACTC

ATTATATAATAAACAAATTACTTTGAGACAAATTAACGTACGTAATAAAACAAAATCAAA

GTGGTAATTAAAATTATTTAATGGTAAACAGTCTAATAGTTAAAATAAAAATGGAAATCA

CATAAATTTTGTAATTGGCCCATTAAAAACAACACTAAGCTTTTAATTTGATTTTGAAAT

TCAAAATAATTTTATTAAATCACAAAGTAAAAGGTTTACAAAGCCGATCATGACAGTGCA

TGTGGGAGGCAAATCGGCATATTTGCACTACAAAAGG-ACCCATGAAGTCTCTGAACATG

CACGCAACACTTTAATCTCTTATTAGTTACTTTGAAAGCTTATTTATATATATAGACACG

CGTAAAAACTTCTTAACCAAGATTTTTTT-ACGTGCTTCC-TTCGCGTTTAATTTGGACC

ATCAAACCGTGCTCAACAGATAAAGAAAAGGGTGCTTTTGATTCAAGATATTGGCC-GAA

AAACACAAGATAGATCCTT-CGATAGATTAAGCCACGCATGAAACGCGAATCCAAAGTGA

TGAAGAAGTGCAGATAGATATTCGTTCACCATATAGAGGAGAATATATCATTCCTACAAA

CAAAATTGATGATGTTTCTAGAATAGAAACGTTAGAATCGAATATTCATCTGTTCCGGGT

GGGGAAGGGCTACTGAAAAGCTGAACTTTTTTAAGAGTTCGAACTTCGAAAGTCAATCAA

TGATTCTTAATTGAGAGGGAAAAGCTATAAAGGACGAGAAGGGAAGGAAATGTTTATAGG

GAATGAATTTCTAATGCATGATGGGAATGACAGATATGAGGAATATATAAAGGTGATTCT

TATATACATTGACATGGATCGGAATCTATTAACTTAAAGTTATTGGGGTCGTGAAATTTA

TTTAATTTTTCTAATTCGTGTCACATGTCACGACAATTAGTGGGG-AACATTTATGTATA

TAGAAATTTCAGAAATTTCTAGCAGCGTGATAAATATAAAGATTTTGGCACAATAAGTTT

TTGGGTCATAATGAGTTT-CTATTTAATCAA-GGCA-TGTAA-TCTATTTTATTATTTAG

-TAGGG-AAACTGAAAACTTAGGGTTGCTATTTGTAGCTCCCACCCTTCTTAGTTCTTAC

TCTTTTCAATATTTTTTAAAAGTTTTA-TATAATACCT-AAATT-GCCCTCCTCCT----

----------------------------------------CACCCCATTGCTCTTCCGTT

GCTCCTCCATGTG-AGTTCTTGTCTTTTTTTTT-CTCTGGTAGCGTTTTGCTGCTCCTTT

TTTCACTCAAGTGTTGCCAATTAA-TTGACAAAAAATGGTTTCTGTTTCATATAGAAACT

ATGTTTTTGTTGTGTAGTCATACATTACGGAATCTAGTTT-CCATTAAATAAGTAAC--G

TGAAAAAAAA--TAAAAGGTGAAATATATATTGTTGGAAAAGAAGCTATGAGGTGCAAGA

ACCGATCGCATGGAGAAGGCAATGAAAGACAAGGAGGAGCAATGGAAGA-----------

---------GAGAAAATGAGAAGATGGAAGGGATGTGAAAATGTTTGAAAAAAACGAGGT

GATCAGTTCTAAAATACGAATTTAGTATTTTCTTTTTAAGAAAATTCTTTCG-AAAGTCG

TGTTTTAAAACATGACTTTTATT-ATTTGAAGTCG---TGTTCTAAAACATGACTTA--T

TCATATCCTT-AATATTTTT---------AAAATTTATCCATTTGTAATATTTTTTAAAA

ATTGACCCATATATGTAAAATACCCGTCAAGATCTCTTTATTATTTTGAAAGCGAAAGCA

TATCACTTCAAACACAATGGAATCGAGGCTATTGACTAAGTATAAATAGAGAAGACTTCA

TATCGGGGTTCATAATTCATAACAAAGCAAACGAGTATATAAGAAAGCATAAGCCAAATT

TTGAGTAAACTAGTGTGCACACTATCCCATGCCTAGTGGAAGTAGGGATCCTCTCGTTGT

TGGGGGAGTAATTGGGGATGTATTGGATCCTTTTGAATATTCTATTCCTATGAGGGTTAC

CTACAATAACAGAGATGTCAGCAATGGATGTGAATTCAAACCCTCACAAGTTGTCAACCA

ACCAAGGGTAAATATCGGTGGTGATGACC

>H05.08

TATTAGACTCCTAATTTAATATTCTTGTTTTATTATAATGCTGATAAGTCTTGTAAATAA

GGGTGAGAAGCACGAATAATTAGTTCATGAGATGTGTATAATTATTATCCTACACGACTT

ATCTTTGATATTTCACACAAGTCTTTCAATGTATAACAAAAACTTTTTAGATACATTTAG

ACTATAGAACTAACAAGTTATATTTTGAACC-AAAAAACAAAGAGAGAGAGAGAGGAACA

TAGAGAAAAGAAGATATGAGAGTTTTTTTTTTTTTTTT---CTAAAACAGAAAGAAACTC

ATTATATAATAAACAAATTACTTTGAGACAAATTAACGTATGTAATAAAACAAAATCAAA

GTGGTAATTAAAATTATTTAATGGTAAACAGTCTAATAGTTAAAATAAAAATGGAAATCA

CATAAATTTTGTAATTGGCCCATTAAAAACAACACTAAGCTTTTAATTTGATTTTGAAAT

TCAAAATAATTTTATTAAATCACAAAGTAAAAGGTTTACAAAGCCGATCATGACAGTGCA

TGTGGGAGGCAAATCGGCATATTTGCACTACAAAAGGGACCCATGAAGTCTCTGAACATG

CACGCAACACTTTAATCTCTTATTAGTTACTTTGAAAGCTTATTTATATATATAGACACG

CGTAAAAACTTCTTAACCAAGATTTTTTT-ACGTGCTTCC-TTCGCGTTTAATTTGGACC

ATCAAACCGTGCTCAACAGATAAAGAAAAGGGTGCTTTTGATTCAAGATATTGGCC-GAA

AAACACAAGATAGATCCTT-CGATAGATTAAGCCACGCATGAAACGCGAATCCAAAGTGA

TGAAGAAGTGCAGATAGATATTCGTTCACCATATAGAGGAGAATATATCATTCCTACAAA

CAAAATTGATGATGTTTCTAGAATAGAAACGTTAGAATCGAATATTCATCTGTTCCGGGT

GGGGAAGGGCTACTGAAAAGCTGAACTTTTTTAAGAGTTCGAACTTCGAAAGTCAATCAA

TGATTCTTAATTGAGAGGGAAAAGCTATAAAGGACGAGAAGGGAAGGAAATGTTTATAGG

GAATGAATTTCTAATGCATGATGGGAATGACAGATATGAGGAATATATAAAGGTGATTCT

TATATACATTGACATGGATCGGAATCTATTAACTTAAAGTTATTGGGGTCGTGAAATTTA

TTTAATTTTTCTAATTCGTGTCACATGTCACGACAATTAGTGGGG-AACATTTATGTATA

TAGAAATTTCAGAAATTTCTAGCAGCGTGATAAATATAAAGATTTTGGCACAATAAGTTT

TTGGATCATAATGAGTTT-CTATTTAATCAA-GGCA-TGTAA-TCTATTTTATTATTTAG

-TAGGG-AAACTGAAAACTTAGGGTTGCTATTTGTAGCTCCCACCCTTCTTAGTTCTTAC

TCTTTTCAATATTTTTTAAAAGTTTTA-TATAATACCT-AAATT-GCCCTCCTCCT----

----------------------------------------CACCCCATTGCTCTTCCGTT

GCTCCTCCATGTG-AGTTCTTGTCTTTTTTTT--CTCTGGTAGCGTTTTGCTGCTCCTTT

TTTCACTCAAGTGTTGCCAATTAA-TTGACAAAAAATGGTTTCTGTTTCATATAGAAACT

ATGTTTTTGTTGTGTAGTCATACATTACGGAATCTAGTTT-CCATTAAATAAGTAAC--G

TGAAAAAAAA--TAAAAGGTGAAATATATATTGTTGGAAAAGAAGCTATGAGGTGCAAGA

ACCGATCACATGGAGAAGGCAATGAAAGACAAGGAGGAGCAATGGAAGA-----------

---------GAGAAAATGAGAAGATGGAAGGGATGTGAAA-TGTTTGAAAAAAACGAGGT

GATCAGTTTTAAAATACGAATTTAGTATTTTCTTTTTAAGAAAATTCTTTCG-AAAGTCG

TGTTTTAAAGCATGACTTTTATT-ATTTGAAGTCG---TGTTCTAAAACATGACTTA--T

TCATATCCTT-AATATTTTT---------AAAATTTATCCATTTGTAATATTTTTTAAAA

ATTGACCCATATATGTAAAATACCCGTCAAGATCTCTTTATTATTTTGAAAGCGAAAGCA

TATCACTTCAAACACAATGGAATCGAGGCTATTGACTAAGTATAAATAGAGAAGACTTCA

TATCGGGGTTCATAATTCATAACAAAGCAAACGAGTATATAAGAAAGCATAAGCCAAATT

TTGAGTAAACTAGTGTGCACACTATCCCATGCCTAGTGGAAGTAGGGATCCTCTCGTTGT

TGGGGGAGTAATTGGGGATGTATTGGATCCTTTTGAATATTCTATTCCTATGAGGGTTAC

CTACAATAACAGAGATGTCAGCAATGGATGTGAATTTAAACCCTCACAAGTTGTCAACCA

ACCAAGGGTAAATATCGGTGGTGATGACC

>CS47.C8

TATTAGACTCCTAATTTAATATTCTTGTTTTATTATAATGCTGATAAGTCTTGTAAATAA

GGGTGAGAAGCACGAATAATTAGTTCATGAGATGTGTATAATTATTATCCTACACGACTT

ATCTTTGATATTTCACACAAGTCTTTCAATGTATAACAAAAACTTTTTAGATACATTTAG

ACTATAGAACTAACAAGTTATATTTTGAACC-AAAAAACAAAGAGAGAGAGAGAGGAACA

TAGAGAAAAGAAGATATGAGAGTTTTTTTTTTTTTTTTT--CTAAAACAGAAAGAAACTC

ATTATATAATAAACAAATTACTTTGAGACAAATTAACGTATGTAATAAAACAAAATCAAA

GTGGTAATTAAAATTATTTAATGGTAAACAGTCTAATAGTTAAAATAAAAATGGAAATCA

CATAAATTTTGTAATTGGCCCATTAAAAACAACACTAAGCTTTTAATTTGATTTTGAAAT

TCAAAATAATTTTATTAAATCACAAAGTAAAAGGTTTACAAAGCCGATCATGACAGTGCA

TGTGGGAGGCAAATCGGCATATTTGCACTACAAAAGG-GCCCATGAAGTCTCTGAACATG

CACGCAACACTTTAATCTCTTATTAGTTACTTTGAAAGCTTATTTATATATATAGACACG

CGTAAAAACTTCTTGACCAAGATTTTTTT-ACGTGCTTCC-TTCGCGTTTAATTTGGACC

ATCAAACCGTGCTCAACAGATAAAGAAAAGGGTGCTTTTGATTCAAGATATTGGCC-GAA

AAACACAAGATAGATCCTT-CGATAGATTAAGCCACGCATGAAACGCGAATCCAAAGTGA

TGAAGAAGTGCAGATAGATATTCGTTCACCATATAGAGGAGAATATATCATTCCTACAAA

CAAAATTGATGACGTTTCTAGAATAGAAACGTTAGAATCGAATATTCATCTGTTCCGGGT

GGGGAAGGGCTACTGAAAAGCTGAACTTTTTTAAGAGTTCGAACTTCGAAAGTCAATCAA

TGATTCTTAATTGAGAGGGAAAAGCTATAAAGGACGAGAAGGGAAGGAAATGTTTATAGG

GAATGAATTTCTAATGCATGATGGGAATGACAGATATGAGGAATATATAAAGGTGATTCT

TATATACATTGACATGGATCGGAATCTATTAACTTAAAGTTATTGGGGTCGTGAAATTTA

TTTAATTTTTCTAATTCGTGTCACATGTCACGACAATTAGTGGGG-AACATTTATGTATA

TAGAAATTTCAGAAATTTCTAGCAGCGTGATAAATATAAAGATTTTGGCACAATAAGTTT

TTGGATCATAATGAGTTT-CTATTTAATCAA-GGCA-TGTAA-TCTATTTTATTATTTAG

-TAGGG-AAACTGAAAACTTAGGGTTGCTATTTGTAGCTCCCACCCTTCCTAGTTCTTAC

TCTTTTCAATATTTTTTAAAAGTTTTA-TATAATACCT-AAATT-GCCCTCCTCCT----

----------------------------------------CACCCCATTGCTCTTCCGTT

GCTCCTCCATGTG-AGTTCTTGTCTTTTTTTTT-CTCTGGTAGCGTTTTGCTGCTTCTTT

TTTCACTCAAGTGTTGCCAATTAA-TTGACAAAAAATGGTTTCTGTTTCATATAGAAACT

ATGTTTTTGTTGTGTAGTCATACATTATGGAATCTAGTTT-CCATTAAATAAGTAAC--G

TGAAAAAAAA--TAAAAGGTGAAATATATATTGTTGGAAAAGAAGCTATGAGGTGCAAGA

ACCGATCACATGGAGAAGGCAATGAAAGACAAGGAGGAGCAATGGAAGA-----------

---------GAGAAAATGAGAAGATGGAAGGGATGTGAAAATGTTTGAAAAAAACGAGGT

GATCAGTTTTAAAATACGAATTTAGTATTTTCTTTTTAAGAAAATTCTTTCG-AAAGTCG

TGTTTTAAAACATGACTTTTATT-ATTTGAAGTCG---TGTTCTAAAACATGACTTA--T

TCATATCCTT-AATATTTTT---------AAAATTTATCCATTTGTAATATTTTTTAAAA

ATTGACCCATATATGTAAAATACCCGTCAAGATCTCTTTATTATTTTGAAAGCGAAAGCA

TATCACTTCAAACACAATGGAATCGAGGCTATTGACTAAGTATAAATAGAGAAGACTTCA

TATCGGGGTTCATAATTCATAACAAAGCAAACGAGTATATAAGAAAGCATAAGCCAAATT

TTGAGTAAACTAGTGTGCACACTATCCCATGCCTAGTGGAAGTAGGGATCCTCTCGTTGT

TGGGGGAGTAATTGGGGATGTATTGGATCCTTTTGAATATTCTATTCCTATGAGGGTTAC

CTACAATAACAGAGATGTCAGCAATGGATGTGAATTCAAACCCTCACAAGTTGTCAACCA

ACCAAGGGTAAATATCGGTGGTGATGACC

>CS05.45

TATTAGACTCCTAATTTAATATTCTTGTTTTATTATAATGCTGATAAGTCTTGTAAATAA

GGGTGAGAAGCACGAATAATTAGTTCATGAGATGTGTATAATTATTATCCTACACGACTT

ATCTTTGATATTTCACACAAGTCTTTCAATGTATAACAAAAACTTTTTAGATACATTTAG

ACTATAGAACTAACAAGTTATATTTTGAACC-AAAAAACAAAGAGAGAGAGAGAGGAACA

TAGAGAAAAGAAGGTATGAGAGTTTTTTTTTTTTTTTTTT-CTAAAACAGAAAGAAACTC

ATTATATAATAAACAAATTACTTTGAGACAAATTAACGTATGTAATAAAACAAAATCAAA

GTGGTAATTAAAATTATTTAATGGTAAACAGTCTAATAGTTAAAATAAAAATGGAAATCA

CATAAATTTTGTAATTGGCCCATTAAAAACAACACTAAGCTTTTAATTTGATTTTGAAAT

TCAAAATAATTTTATTAAATCACAAAGTAAAAGGTTTACAAAGCCGATCATGACAGTGCA

TGTGGGAGGCAAATCGGCATATTTGCACTACAAAAGG-ACCCATGAAGTCTCTGAACATG

CACGCAACACTTTAATCTCTTATTAGTTACTTTGAAAGCTTATTTATATATATAGACACG

CGTAAAAACTTCTTAACCAAGATTTTTTT-ACGTGCTTCC-TTCGCGTTTAATTTGGACC

ATCAAACCGTGCTCAACAGATAAGGAAAAGGGTGCTTTTGATTCAAGATATTGGCC-GAA

AAACACAAGATAGATCCTT-CGATAGATTAAGCCACGCATGAAACGCGAATCCAAAGTGA

TGAAGAAGTGCAGATAGATATTCGTTCACCATATAGAGGAGAATATATCATTCCTACAAA

CAAAATTGATGATGTTTCTAGAATAGAAACGTTAGAATCGAATATTCATCTGTTCCGGGT

GGGGAAGGGCTACTGAAAAGCTGAACTTTTTTAAGAGTTCGAACTTCGAAAGTCAATCAA

TGATTCTTAATTGAGAGGGAAAAGCTATAAAGGACGAGAAGGGAAGGAAATGTTTATAGG

GAATGAATTTCTAATGCATGATGGGAATGACAGATATGAGGAATATATAAAGGTGATTCT

TATATACATTGACATGGATCGGAATCTATTAACTTAAAGTTATTGGGGTCGTGAAATTTA

TTTAATTTTTCTAATTCGTGTCACATGTCACGACAATTAGTGGGG-AACATTTATGTATA

TAGAAATTTCAGAAATTTCTAGCAGCGTGATAAATATAAAGATTTTGGCACAATAAGTTT

TTGGATCATAATGAGTTT-CTATTTAATCAA-GGCA-TGTAA-TCTATTTTATTATTTAG

-TAGGG-AAACTGAAAACTTAGGGTTGCTATTTGTAGCTCCCACCCTTCTTAGTTCTTAC

TCTTTTCAATATTTTTTAAAAGTTTTA-TATAATACCT-AAATT-GCCCTCCTCCT----

----------------------------------------CACCCCATTGCTCTTCCGTT

GCTCCTCCATGTG-AGTTCTTGTCTTTTTTTTT-CTCTGGTAGCGTTTTGCTGCTCCTTT

TTTCACTCAAGTGTTGCCAATTAA-TTGACAAAAAATGGTTTCTGTTTCATATAGAAACT

ATGTTTTTGTTGTGTAGTCATACATTACGGAATCTAGTTT-CCATTAAATAAGTAAC--G

TGAAAAAAAA--TAAAAGGTGAAATATATATTGTTGGAAAAGAAGCTATGAGGTGCAAGA

ACCGATCACATGGAGAAGGCAATGAAAGACAAGGAGGAGCAATGGAAGA-----------

---------GAGAAAATGAGAAGATGGAAGGGATGTGAAAATGTTTGAAAAAAACGAGGT

GATCAGTTTTAAAATACGAACTTAGTATTTTCTTTTTAAGAAAATTCTTTCG-AAAGTCG

TGTTTTAAAACATGACTTTTATT-ATTTGAAGTCG---TGTTCTAAAACATGACTTA--T

TCATATCCTT-AATATTTTT---------AAAATTTATCCATTTGTAATATTTTTTAAAA

ATTGACCCATATATGTAAAATACCCGTCAAGATCTCTTTATTATTTTGAAAGCGAAAGCA

TATCACTTCAAACACAATGGAATCGAGGCTATTGACTAAGTATAAATAGAGAAGACTTCA

TATCGGGGTTCATAATTCATAACAAAGCAAACGAGTATATAAGAAAGCATAAGCCAAATT

TTGAGTAAACTAGTGTGCACACTATCCCATGCCTAGTGGAAGTAGGGATCCTCTCGTTGT

TGGGGGAGTAATTGGGGATGTATTGGATCCTTTTGAATATTCTATTCCTATGAGGGTTAC

CTACAATAACAGAGATGTCAGCAATGGATGTGAATTCAAACCCTCACAAGTTGTCAACCA

ACCAAGGGTAAATATCGGTGGTGATGACC

>CS15.11

TATTAGACTCCTAATTTAATATTCTTGTTTTATTATAATGCTGATAAGTCTTGTGAATAA

GGGTGAGAAGCACGAATAATTAGTTCATGAGATGTGTATAATTATTATCCTACACGACTT

ATCTTTGATATTTCACACAAGTCTTTCAATGTATAACAAAAACTTTTTAGATACATTTAG

ACTATAGAACTAACAAGTTATATTTTGAACC-AAAAAACAAAGAGAGAGAGAGAGGAACA

TAGAGAAAAGAAGATATGAGAGTCTTTTTTTTTTTTTTTT-CTAAAACAGAAAGAAACTC

ATTATATAATAAACAAATTACTTTGAGACAAATTAACGTATGTAATAAAACAAAATCAAA

GTGGTAATTAAAATTATTTAATGGTAAACAGTCTAATAGTTAAAATAAAAATGGAAATCA

CATAAATTTTGTAATTGGCCCATTAAAAACAACACTAAGCTTTTAATTTGATTTTGAAAT

TCAAAATAATTTTATTAAATCACAAAGTAAAAGGTTCACAAAGCCGATCATGACAGTGCA

TGTGGGAGGCAAATCGGCATATTTGCACTACAAAAGG-ACCCATGAAGTCTCTGAACATG

CACGCAACACTTTAATCTCTTATTAGTTACTTTGAAAGCTTATTTATATATATAGACACG

CGTAAAAACTTCTTAACCAAGATTTTTTT-ACGTGCTTCC-TCCGCGTTTAATTTGGACC

ATCAAACCGTGCTCAACAGATAAAGAAAAGGGTGCTTTTGATTCAAGATATTGGCC-GAA

AAACACAAGATAGATCCTT-CGATAGATTAAGCCACGCATGAAACGCGAATCCAAAGTGA

TGAAGAAGTGCAGATAGATATTCGTTCACCATATAGAGGAGAATATATCATTCCTACAAA

CAAAATTGATGATGTTTCTAGAATAGAAACGTTAGAATCGAATATTCATCTGTTCCGGGT

GGGGAAGGGCTACTGAAAAGCTGAACTTTTTTAAGAGTTCGAACTTCGAAAGTCAATCAA

TGATTCTTAATTGAGAGGGAAAAGCTATAAAGGACGAGAAGGGAAGGAAATGTTTATAGG

GAATGAATTTCTAATGCATGATGGGAATGACAGATATGAGGAATATATAAAGGTGATTCT

TATATACATTGACATGGATCGGAATCTATTAACTTAAAGTTATTGGGGTCGTGAAATTTA

TTTAATTTTTCTAATTCGTGTCACATGTCACGACAATTAGTGGGG-AACATTTATGTATA

TAGAAATTTCAGAAATTTCTAGCAGCGTGATAAATATAAAGATTTTGGCACAATAAGTTT

TTGGATCATAATGAGTTT-CTATTTAATCAA-GGCA-TGTAA-TCTATTTTATTATTTAG

-TAGGG-AAACTGAAAACTTAGGGTTGCTATTTGCAGCTCCCACCCTTCTTAGTCCTTAC

TCTTTTCAATATTTTTTAAAAGTTTTA-TATAATACCT-AAATT-GCCCTCCTCCT----

----------------------------------------CACCCCATTGCTCTTCCGCT

GCTCCTCCATGTG-AGTTCTTGTCTTTTTTTT--CTCTGGTAGCGTTT-GCTGCTCCTTT

TT-CACTCAAGTGTTGCCAATTAA-TTGACAAAAAATGGTTTCTGTTTCATATAGAAACT

ATGTTTTTGTTGTGTAGTCATACATTACGGAATCTAGTTT-CCATTAAATAAGTAAC--G

TGAAAAAAAA--TAAAAGGTGAAATATATATTGTTGGAAAAGAAGCTATGAGGTGCAAGA

ACCGATCACATGGAGAAGGCAATGAAAGACAAGGAGGAGCAATGGAAGA-----------

---------GAGAAAATGAGAAGATGGAAGGGATGTGAAAATGTTTGAAAAAAACGAGGT

GATCAGTTTTAAAATACGAATTTAGTATTTTCTTTTTAAGAAAATTCTTTCG-AAAGTCG

TGTTTTAAAACATGACTTTTATT-ATTTGAAGTCG---TGTTCTAAAACATGACTTA--T

TCATATCCTT-AATATTTTT---------AAAATTTATCCATTTGTAATATTTTTTAAAA

ATTGACCCATATATGTAAAATACCCGTCAAGATCTCTTTATTATTTTGAAAGCGAAAGCA

TATCACTTCAAACACAATGGAATCGAGGCTATTGACTAAGTATAAATAGAGAAGACTTCA

TATCGGGGTTCATAATTCATAACAAAGCAAACGAGTATATAAGAAAGCATAAGCCAAATT

TTGAGTAAACTAGTGTGCACACTATCCCATGCCTAGTGGAAGTAGGGATCCTCTCGTTGT

TGGGGGAGTAATTGGGGATGTATTGGATCCTTTTGAATATTCTATTCCTATGAGGGTTAC

CTACAATAACAGAGATGTCAGCAATGGATGTGAATTCAAACCCTCACAAGTTGTCAACCA

ACCAAGGGTAAATATCGGCGGTGATGACC

>CS16.23

TATTAGACTCCTAATTTAATATTCTTGTTTTATTATAATGCTGATAAGTCTTGTAAATAA

GGGTGAGAAGCACGAATAATTAGTTCATGAGATGTGTATAATTATTATCCTACACGACTC

ATCTTTGATATTTCACACAAGTCTTTCAATGTATAACAAAAACTTTTTAGATACATTTAG

ACTATAGAACTAACAAGTTATATTTTGAACC-AAAAAGCAAAGAGAGAGAGAGAGGAACA

TAGAGAAAAGAAGATATGAGAGTTTTTTTTTTTTTTTT---CTAAAACAGAAAGAAACTC

ATTATATAGTAAACAAATTACTTTGAGACAAATTAACGTATGTAATAAAACAAAATCAAA

GTGGTAATTAAAATTATTTAATGGTAAACAGTCTAATAGTTAAAATAAAAATGGAAATCA

CATAAATTTTGTAATTGGCCCATTAAAAACAACACTAAGCTTTTAATTTGATTTTGAAAT

TCAAAATAATTTTATTAAATCACAAAGTAAAAGGTTTACAAAGCCGATCATGACAGTGCA

TGTGGGAGGCAAATCGGCATATTTGCACTACAAAAGG-ACCCATGAAGTCTCTGAACATG

CACGCAACACTTTAATCTCTTATTAGTTACTTTGAAAGCTTATTTATATATATAGACACG

CGTAAAAACTTCTTAACCAAGATTTTTTT-ACGTGCTTCC-TTCGCGTTTAATTTGGACC

GTCAAACCGTGCTCAACAGATAAAGAAAAGGGTGCTTTTGATTCAAGATATTGGCC-GAA

AAACACAAGATAGGTCCTT-CGATAGATTAAGCCACGCATGAAACGCGAATCCAAAGTGA

TGAAGAAGTGCAGATAGATATTCGTTCACCATATAGAGGAGAATATATCATTCCTACAAA

CAAAATTGATGATGTTTCTAGAATAGAAACGTTAGAATCGAATATTCATCTGTTCCGGGT

GGGGAAGGGCTACTGAAAAGCTGAACTTTTTTAAGAGTTCGAACTTCGAAAGTCAATCAA

TGATTCTTAATTGAGAGGGAAAAGCTTTAAAGGACGAGAAGGGAAGGAAATGTTTATAGG

GAATGAATTTCTAATGCATGATGGGAATGACAGATATGAGGAATATATAAAGGTGATTCT

TATATACATTGACATGGATCGGAATCTATTAACTTAATGTTATTGGGGTCGTGAAGTTTA

TTTAATTTTTCTAATTCGTGTCACATGTCACGACAATTAGTGGGG-AACATTTATGTATA

TAGAAATTTCAGAAATTTCTAGCAGCGTGATAAATATAAAGATTTTGGCACAATAAGTTT

TTGGATCATAATGAGTTT-CTATTTAATCAA-GGCA-TGTAA-TCTATTTTATTATTTAG

-TAGGG-AAACTGAAAACTTAGGGTTGCTATTTGTAGCTCCCACCCTTCTTAGTTCTTAC

TCTTTTCAATATTTTTTAAAAGTTTTA-TATAATACCT-AAATT-GCCCTCCTCCT----

----------------------------------------CACCCCATTGCTCTTCCGTT

GCTCCTCCATGTG-AGTTCTTGTCTTTTTTTT--CTCTGGTAGCGTTTTGCTGCTCCTTT

TTTCACTCAAGTGTTGCCAATTAA-TTGACAAAAAATGGTTTCTGTTTCATATAGAAACT

ATGTTTTTGTTGTGTAGTCATACATTACGGAATCTGGTTT-CCATTAAATAAGTAAC--G

TGAAAAAAAA--TAAAAGGTGAAATATATATTGTTGGAAAAGAAGCTATGAGGTGCAAGA

ACCGATCACATGGAGAAGGCAATGAAAGACAAGGAGGAGCAATGGAAGA-----------

---------GAGAAAATGAGAAGATGGAAGGGATGTGAAAATGTTTGAAAAAAACGAGGT

GATCAGTTTTAAAATACGAATTTAGTATTTTCTTTTTAAGAAAATTCTTTCG-AAAGTCG

TGTTTTAAAACATGACTTTTATT-ATTTGAAGTCG---TGTTCTAAAACATGACTTA--T

TCATATCCTC-AATATTTTT---------AAAATTTATCCATTTGTAATATTTTTTAAAA

ATTGACCCATATATGTAAAATACCCGTCAAGATCTCTTTATTATTCTGAAAGCGAAAGCA

TATCACTTCAAACACAATGGAATCGAGGCTATTGACTAAGTATAAATAGAGAAGACTTCA

TATCGGGGTTCATAATTCATAACAAAGCAAACGAGTATATAAGAAAGCATAAGCCAAATT

TTGAGTAAACTAGTGTGCACACTATCCCATGCCTAGTGGAAGTAGGGATCCTCTCGTTGT

TGGGGGAGTAATTGGGGATGTATTGGATCCTTTTGAATATTCTATTCCTATGAGGGTTAC

CTACAATAACAGAGATGTCAGCAGTGGATGTGAATTCAAACCCTCACAAGTTGTCAACCA

ACCAAGGGTAAATATCGGTGGTGATGACC

>CS46.B7

TATTAGACTCCTAATTTAATATTCTTGTTTTATTATAATGCTGATAAGTCTTGTAAATAA

GGGTGAGAAGCACGAATAATTAGTCCATGAGATGTGTATAATTATTATCCTACACGACTT

ATCTTTGATATTTCACACAAGTCTTTCAATGTATAACAAAAACTTTTTAGATACATTTAG

ACTATAGAACTAACAAGTTATATTTTGAACC-AAAAAACAAAGAGAGAGAGAGAGGAACA

TAGAGAAAAGAAGATATGAGAGTTTTTTTTTTTTTTTT---CTAAAACAGAAAGAAACTC

ATTATATAATAAACAAATTACTTTGAGACAAATTAACGTATGTAATAAAACAAAATCAAA

GTGGTAATTAAAATTATTTAATGGTAAACAGTCTAATAGTTAAAATAAAAATGGAAATCA

CATAAATTTTGTAATTGGCCCATTAAAAACAACACTAAGCTTTTAATTTGATTTTGAAAT

TCAAAATAATTTTATTAAATCACAAAGTAAAAGGTTTACAAAGCCGATCATGACAGTGCA

TGTGGGAGGCAAATCGGCATATTTGCACTACAAAAGG-ACCCATGAAGTCTCTGAACATG

CACGCAACACTTTAATCTCTTATTAGTTACTTTGAAAGCTTATTTATATATATAGACACG

CGTAAAAACTTCTTAACCAAGATTTTTTT-ACGTGCTTCC-TTCGCGTTTAATTTGGACC

ATCAAACCGTGCTCAACAGATAAAGAAAAGGGTGCTTTTGATTCAAGATATTGGCC-GAA

AAACACAAGATAGACCCTT-CGATAGATTAAGCCACGCATGAAACGCGAATCCAAAGTGA

TGAAGAAGTGCAGATAGATATTCGTTCACCATATAGAGGAGAATATATCATTCCTACAAA

CAAAATTGATGATGTTTCTAGAATAGAAACGTTAGAATCGAATATTCATCTGTTCCGGGT

GGGGAAGGGCTACTGAAAAGCTGAACTTTTTTAAGAGTTCGAACTTCGAAAGTCAATCAA

TGATTCTTAATTGAGAGGGAAAAGCTATAAAGGACGAGAAGGGAAGGAAATGTTTATAGG

GAATGAATTTCTAATGCATGATGGGAATGACAGATATGAGGAATATATAAAGGTGATTCT

TATATACATTGACATGGATCGGAATCTATTAACTTAAAGTTATTGGGGTCGTGAAATTTA

TTTAATTTTTCTAATTCGTGTCACATGTCACGACAATTAGTGGGG-AACATTTATGTATA

TAGAAATTTCAGAAATTTCTAGCAGCGTGATAAATATAAAGATTTTGGCACAATAAGTTT

TTGGATCATAATGAGTTT-CTATTTAATCAA-GGCA-TGTAA-TCTATTTTATTATTTAG

-TAGGG-AAACTGAAAACTTAGGGTTGCTATTTGTAGCTCCCACCCTTCTTAGTTCTTAC

TCTTTTCAATATTTTTTAAAAGTTTTA-TATAATACCT-AAATT-GCCCTCCTCCT----

----------------------------------------CACCCCATTGCTCTTCCGTT

GCTCCTCCATGTG-AGTTCTTGTCTTTTTTTTT-CTCTGGTAGCGTTTTGCTGCTCCTTT

TTTCACTCAAGTGTTGCCAATTAA-TTGACAAAAAATGGTTTCTGTTTCATATAGAAACT

ATGTTTTTGTTGTGTAGTCATACATTACGGAATCTAGTTT-CCATTAAATAAGTAAC--G

TGAAAAAAAA--TAAAAGGTGAAATATATATTGTTGGAAAAGAAGCTATGAGGTGCAAGA

ACCGATCACATGGAGAAGGCAATGGAAGACAAGGAGGAGCAATGGAAGA-----------

---------GAGAAAATGAGAAGATGGAAGGGATGTGAAAATGTTTGAAAAAAACGAGGT

GATCAGTTTTAAAATACGAATTTAGTATTTTCTTTTTAAGAAAATTCTTTCG-AAAGTCG

TGTTTTAAAACATGACTTTTATT-ATTTGAAGTCG---TGTTCTAAAACATGACTTA--T

TCATATCCTT-AATATTTTT---------AAAATTTATCCATTTGTAATATTTTTTAAAA

ATTGACCCATATATGTAAAATACCCGTCAAGATCTCTTTATTATTTTGAAAGCGAAAGCA

TATCACTTCAAACACAATGGAATCGAGGCTATTGACTAAGTATAAATAGAGAAGACTTCA

TATCGGGGTTCATAATTCATAACAAAGCAAACGAGTATATAAGAAAGCATAAGCCAAATT

TTGAGTAAACTAGTGTGCACACTATCCCATGCCTAGTGGAAGTAGGGATCCTCTCGTTGT

TGGGGGAGTAATTGGGGATGTATTGGATCCTTTTGAATATTCTATTCCTATGAGGGTTAC

CTACAATAACAGAGATGTCAGCAATGGATGTGAATTCAAACCCTCACAAGTTGTCAACCA

ACCAAGGGTAAATATCGGTGGTGATGACC

>CS53.J8

TATTAGACTCCTAATTTAATATTCTTGTTTTATTATAATACTGATAAGTCTTGTAAATAA

GGGTGAGAAGCACGAATAATTAGTTCATGAGATGTGTATAATTATTATCCTACACGACTT

ATCTTTGATATTTCACACAAGTCTTTCAATGTATAACAAAAACTTTTTAGATACATTTAG

ACTATAGAACTAACAAGTTATATTTTGAACC-AAAAAACAAAGAGAGAGAGAGAGGAACA

TAGAGAAAAGAAGATATGAGAGTTTTTTTTTTTTTTTT---CTAAAACAGAAAGAAACTC

ATTATATAATAAACAAATTACTTTGAGACAAATTAACGTATGTAATAAAACAAAATCAAA

GTGGTAATTAAAATTATTTAATGGTAAACAGTCTAATAGTTAAAATAAAAATGGAAATCA

CATAAATTTTGTAATTGGCCCATTAAAAACAACACTAAGCTTTTAATTTGATTTTGAAAT

TCAAAATAATTTTATTAAATCACAAAGTAAAAGGTTTACAAAGCCGATCATGACAGTGCA

TGTGGGAGGCAAATCGGCATATTTGCACTACAAAAGG-ACCCATGAAGTCTCTGAACATG

CACGCAACACTTTAATCTCTTATTAGTTACTTTGAAAGCTTATTTATATATATAGACACG

CGTAAAAACTTCTTAACCAAGATTTTTTT-ACGTGCTTCC-TTCGCGTTTAATTTGGACC

ATCAAACCGTGCTCAACAGATAAAGAAAAGGGTGCTTTTGATTCAAGATATTGGCC-GAA

AAACACAAGATAGATCCTT-CGATAGATTAAGCCACGCATGAAACGCGAATCCAAAGTGA

TGAAGAAGTGCAGATAGATATTCGTTCACCATATAGAGGGGAATATATCATTCCTACAAG

CAAAATTGATGATGTTTCTAGAATAGAAACGTTAGAATCGAATATTCATCTGTTCCGGGT

GGGGAAGGGCTACTGAAAAGCTGAACTTTTTTAAGAGTTCGAACTTCGAAAGTCAATCAA

TGATTCTTAATTGAGAGGGAAAAGCTATGAAGGACGAGAAGGGAAGGAAATGTTTATAGG

GAATGAATTTCTAATGCATGATGGGAATGACAGATATGAGGAATATATAAAGGTGATTCT

TATATACATTGACATGGATCGGAATCTATTAACTTAAAGTTATTGGGGTCGTGAAATTTA

TTTAATTTTTCTAATTCGTGTCACATGTCACGACAATTAGTGGGG-AACATTTATGTATA

TAGAAATTTCAGAAATTTCTAGCAGCGTGATAAATATAAAGATTTTGGCACAATAAGTTT

TTGGATCATAATGAGTTT-CTATTTAATCAA-GGCA-TGTAA-TCTATTTTATTATTTAG

-TAGGG-AAACTGAAAACTTAGGGTTGCTATTTGTAGCTCCCACCCTTCTTAGTTCTTAC

TCTTTTCAATATTTTTTAAAAGTTTTA-TATAATACCT-AAATT-GCCCTCCTCCT----

----------------------------------------CACCCCATTGCTCTTCCGTT

GCTCCTCCATGTG-AGTTCTTGTCTTTTTTTTT-CTCTGGTAGCGTTTTGCTGCTCCTTT

TTTCACTCAAGTGTTGCCAATTAA-TTGACAAAAAATGGTTTCTGTTTCATATAGAAACT

ATGTTTTTGTTGTGTAGTCATACATTACGGAATCTAGTTT-CCATTAAATAAGTAAC--G

TGAAAAAAAA--TAAAAGGTGAAATATATATTGTTGGAAAAGAAGCTATGAGGTGCAAGA

ACCGATCACATGGAGAAGGCAATGAAAGACAAGGAGGAGCAATGGAAGA-----------

---------GAGAAAATGAGAAGATGGAAGGGATGTGAAAATGTTTGAAAAAAACGAGGT

GATCAGTTTTAAAATACGAATTTAGTATTTTCTTTTTAAGAAAATTCTTTCG-AAAGTCG

TGTTTTAAAACATGACTTTTATT-ATTTGAAGTCG---TGTTCTAAAACATGACTTA--T

TCATATCCTT-AATATTTTT---------AAAATTTATCCATTTGTAATATTTTTTAAAA

ATTGACCCATATATGTAAAATACCCGTCAAGATCTCTTTATTATTTTGAAAGCGAAAGCA

TATCACTTCAAGCACAATGGAATCGAGGCTATTGACTAAGTATAAATAGAGAAGACTTCA

TATCGGGGTTCATAATTCATAACAAAGCAAACGAGTATATAAGAAAGCATAAGCCAAATT

TTGAGTAAACTAGTGTGCACACTATCCCATGCCTAGTGGAAGTAGGGATCCTCTCGTTGT

TGGGGGAGTAATTGGGGATGTATTGGATCCTTTTGAATATTCTATTCCTATGAGGGTTAC

CTACAATAACAGAGATGTCAGCAATGGATGTGAATTCAAACCCTCACAAGTTGTCAACCA

ACCAAGGGTAAATATCGGTGGTGATGACC

>CS22.06

TATTAGACTCCTAATTTAATATTCTTGTTTTATTATAATGCTGGTAAGTCTTGTAAATAA

GGGTGAGAAGCACGAATAATTAGTTCATGAGATGTGTATAATTATTATCCTACACGACTT

ATCTTTGATATTTCACACAAGTCTTTCAATGTATAACAAAAACTTTTTAGATACATTTAG

ACTATAGAACTAACAAGTTATATTTTGAACC-AAAAAACAAAGAGAGAGAGAGAGGAACA

TAGAGAAAAGAAGATATGAGAGTTTTTTTTTTTTTTTTT--CTAAAACAGAAAGAAACTC

ATTATATAATAAACAAATTACTTTGAGACAAATTAACGTATGTAATAAAACAAAATCAAA

GTGGTAATTAAAATTATTTAATGGTAAACAGTCTAATAGTTAAAATAAAAATGGAAATCA

CATAAATTTTGTAATTGGCCCATTAAAAACAACACTAAGCTTTTAATTTGATTTTGAAAT

TCAAAATAATTTTATTAAATCACAAAGTAAAAGGTTTACAAAGCCGATCATGACAGTGCA

TGTGGGAGGCAAATCGGCATATTTGCACTACAAAAGG-ACCCATGAAGTCTCTGAACATG

CACGCAACACTTTAATCTCTTATTAGTTACTTTGAAAGCTTATTTATATATATAGACACG

CGTAAAAACTTCTTAACCAAGATTTTTTT-ACGTGCTTCC-TTCGCGTTTAATTTGGACC

ATCAAACCGTGCTCAACAGATAAAGAAAAGGGTGCTTTTGATTCAAGATATTGGCC-GAA

AAACACAAGATAGATCCTT-CGATAGATTAAGCCACGCATGAAACGCGAATCCAAAGTGA

TGAAGAAGTGCAGATAGATATTCGTTCACCATATAGAGGAGAATATATCATTCCTACAAA

CAAAATTGATGATGTTTCTAGAATAGAAACGTTAGAATCGAATATTCATCTGTTCCGGGT

GGGGAAGGGCTACTGAAAAGCTGAACTTTTTTAAGAGTTCGAACTTCGAAAGTCAATCAA

TGATTCTTAATTGAGAGGGAAAAGCTATAAAGGACGAGAAGGGAGGGAAATGTTTATAGG

GAATGAATTTCTAATGCATGATGGGAATGACAGATATGAGGAATATATAAAGGTGATTCT

TATATACATTGACATGGATCGGAATCTATTAACTTAAAGTTATTGGGGTCGTGAAATTTA

TTTAATTTTTCTAATTCGTGTCACATGTCACGACAATTAGTGGGG-AACATTTATGTATA

TAGAAATTTCAGAAATTTCTAGCAGCGTGATAAATATAAAGATTTTGGCACAATAAGTTT

TTGGATCATAATGAGTTT-CTATTTAATCAA-GGCA-TGTAA-TCTATTTTATTATTTAG

-TAGGG-AAACTGAAAACTTAGGGTTGCTATTTGTAGCTCCCACCCTTCTTAGTTCTTAC

TCTTTTCAATATTTTTTAAAAGTTTTA-TATAATACCT-AAATT-GCCCTCCTCCT----

----------------------------------------CACCCCATTGCTCTTCCGTT

GCTCCTCCATGTG-AGTTCTTGTCTTTTTTTTT-CTCTGGTAGCGTTTTGCTGCTCCTTT

TTCCACTCAAGTGTTGCCAATTAA-TTGACAAAAAATGGTTTCTGTTTCATATAGAAACT

ATGTTTTTGTTGTGTAGTCATACATTACGGAATCTAGTTT-CCATTAAATAAGTAAC--G

TGAAAAAAAA--TAAAAGGTGAAATATATATTGTTGGAAAAGAAGCTATGAGGTGCAAGA

ACCGATCACATGGAGAAGGCAATGAAAGACAAGGAGGAGCAATGGAAGA-----------

---------GAGAAAATGAGAAGATGGAAGGGATGTGAAAATGTTTGAAAAAAACGAGGT

GATCAGTTTTAAAATACGAATTTAGTATTTTCTTTTTAAGAAAATTCTTTCG-AAAGTCG

TGTTTTAAAACATGACTTTTATT-ATTTGAAGTCG---TGTTCTAAAACATGACTTA--T

TCGTATCCTT-AATATTTTT---------AAAATTTATCCATTTGTAATATTTTTTAAAA

ATTGACCCATATATGTAAAATACCCGTCAAGATCTCTTTATTATTTTGAAAGCGAAAGCA

TATCACTTCAAACACAATGGAATCGAGGCTATTGACTAAGTATAAATAGAGAAGACTTCA

TATCGGGGTTCATAATTCATAACAAAGCAAACGAGTATATAAGAAAGCATAAGCCAAATT

TTGAGTAAACTAGTGTGCGCACTATCCCATGCCTAGTGGAAGTAGGGATCCTCTCGTTGT

TGGGGGAGTAATTGGGGATGTATTGGATCCTTTTGAATATTCTATTCCTATGAGGGTTAC

CTACAATAACAGAGATGTCAGCAATGGATGTGAATTCAAACCCTCACAAGTTGTCAACCA

ACCAAGGGTAAATATCGGTGGTGATGACC

>CS30.12

TATTAGACTCCTAATTTAATATTCTTGTTTTATTATAATGCTGATAAGTCTTGTAAATAA

GGGTGAGAAGCACGAATAATTAGTTCATGAGATGTGTATAATTATTATCCTACACGACTT

ATCTTTGATATTTCACACAAGTCTTTCAATGTATAACAAAAACTTTTTAGATGCATTTAG

ACTATAGACCTAACAAGTTATATTTTGAACC-AAAAAACAAAGAGAGAGAGAGAGGAACA

TAGAGAAAAGAAGATATGAGAGTTTTTTTTTTTTTTTTT--CTAAAACAGAAAGAAACTC

ATTATATAATAAACAAATTACTTTGAGACAAATTAACGTATGTAATAAAACAAAATCAAA

GTGGTAATTAAAATTATTTAATGGTAAACAGTCTAATAGTTAAAATAAAAATGGAAATCA

CATAAATTTTGTAATTGGCCCATTAAAAACAACACTAAGCTTTTAATTTGATTTTGAAAT

TCAAAATAATTTTATTAAATCACAAAGTAAAAGGTTTACAAAGCCGATCATGACAGTGCA

TGTGGGAGGCAAATCGGCATATTTGCACTACAAAAGG-ACCCATGAAGTCTCTGAACATG

CACGCAACACTTTAATCTCTTATTAGTTACTTTGAAAGCTTATTTATATATATAGACACG

CGTAAAAACTTCTTAACCAAGATTTTTTT-ACGTGCTTCC-TTCGCGTTTAATTTGGACC

ATCAAACCGTGCTCAACAGATAAAGAAAAGGGTGCTTTTGATTCAAGATATTGGCC-GAA

AAACACAAGATAGATCCTT-CGATAGATTAAGCCACGCATGAGACGCGAATCCAAAGTGA

TGAAGAAGTGCAGATAGATATTCGTTCACCATATAGAGGAGAATATATCATTCCTACAAA

CAAAATTGATGATGTTTCTAGAATAGAAACGTTAGAATCGAATATTCATCTGTTCCGGGT

GGGGAAGGGCTACTGAAAAGCTGAACTTTTTTAAGAGTTCGAACTTCGAAAGTCAATCAA

TGATTCTTAATTGAGAGGGAAAAGCTATAAAGGACGAGAAGGGAAGGAAATGTTTATAGG

GAATGAATTTCTAATGCATGATGGGAATGACAGATATGAGGAATATATAAAGGTGATTCT

TATATACATTGACATGGATCGGAATCTATTAACTTAAAGTTATTGGGGTCGTGAAATTTA

TTTAATTTTTCTAATTCGTGTCACATGTCACGACAATTAGTGGGG-AACATTTATGTATA

TAGAAATTTCAGAAATTTCTAGCAGCGTGATAAATATAAAGATTTTGGCACAATAAGTTT

TTGGATCATAATGAGTTT-CTATTTAATCAA-GGCA-TGTAA-TCTATTTTATTATTTAG

-TAGGG-AAACTGAAAACTTAGGGTTGCTATTTGTAGCTCCCACCCTTCTTAGTTCTTAC

TCTTTTCAATATTTTTTAAAAGTTTTA-TATAATACCT-AAATT-GCCCTCCTCCT----

----------------------------------------CACCCCATTGCTCTTCCGTT

GCTCCTCCATGTG-AGTTCTTGTCTTTTTTTTT-CTCTGGTAGCGTTTTGCTGCTCCTTT

TTTCACTCAAGTGTTGCCAATTAA-TTGACAAAAAATGGTTTCTGTTTCATATAGAAACT

ATGTTTTTGTTGTGTAGTCATACATTACGGAATCTAGTTT-CCATTAAATAAGTAAC--G

TGAAAAAAAA--TAAAAGGTGAAATATATATTGTTGGAAAAGAAGCTATGAGGTGCAAGA

ACCGATCACATGGAGAAGGCAATGAAAGACAAGGAGGAGCAATGGAAGA-----------

---------GAGAAAATGAGAAGATGGAAGGGATGTGAAAATGTTTGAAAAAAACGAGGT

GATCAGTTTTAAAATACGAATTTAGTATTTTCTTTTTAAGAAAATTCTTTCG-AAAGTCG

TGTTTTAAAACATGACTTTTATT-ATTTGAAGTCG---TGTTCTAAAACATGACTTA--T

TCATATCCTT-AATATTTTT---------AAAATTTATCCATTTGTAATATTTTTTAAAA

ATTGACCCATATATGTAAAATACCCGTCAAGATCTCTTTATTATTTTGAAAGCGAAAGCA

TATCACTTCAAACACAATGGAATCGAGGCTATTGACTAAGTATAAATAGAGAAGACTTCA

TATCGGGGTTCATAATTCATAACAAAGCAAACGAGTATATAAGAAAGCATAAGCCAAATT

TTGAGTAAACTAGTGTGCACACTATCCCATGCCTAGTGGAAGTAGGGATCCTCTCGTTGT

TGGGGGAGTAATTGGGGATGTATTGGATCCTTTTGAATATTCTATTCCTATGAGGGTTAC

CTACAATAACAGAGATGTCAGCAATGGATGTGAATTCAAACCCTCACAAGTTGTCAACCA

ACCAAGGGTAAATATCGGTGGTGATGACC

>CS41.83

TATTAGACTCCTAATTTGATATTCTTGTTTTATTATAATGCTGATAAGTCTTGTAAATAA

GGGTGAGAAGCACGAATAATCAGTTCATGAGATGTGTATAATTATTATCCTACACGACTT

ATCTTTGATATTTCACACAAGTCTTTCAATGTATAACAAAAATTTTTTAGATACATTTAG

ACTATAGAACTAACAAGTTATATTTTGAACC-AAAAAACAAAGAGAGAGAG----GAACA

TAGAGAAAAGAAGATATGAGAGTTTTTTTTTTTTTTT----CTAAAACAGAAAGAAACTC

ATTATATAATAAACAAATTAATTTGAGACAAATTAACGTATGTAATAAAACAAAATCAAA

GTGGTAATTAAAATTATTTAATGGTAAACAGTCTAATAGTTAAAATAAAAATGGAAATCA

CATAAATTTTGTAATTGGCCCATTAAAAACAACACTAAGCTTTTAATTTGATTTTGAAAT

TCAAAATAATTTTATTAAATCACAAAGTAAAAGGTTTACAAAGCCGATCATGACAGTGCA

TGTGGGAGGCAAATCGGCATATTTGCACTACAAAAGG-ACCCATGAAGCCTCTGAACATG

CACGCAACACTTTAATCTCTTATTAGTTACTTTGAAAGCTTATTTATATATATAGACACG

CGTAAAAACTTCTTAACCAAGATTTTTTT-ACGTGCTTCC-TTCGCGTTTAATTTGGACC

ATCAAGCCGTGCTCAACAGATAAAGAAAAGGGTGCTTTTGATTCAAGATATTGGCC-GAA

AAACACAAGATAGATCCTT-CGATAGATTAAGCCACGCATGAAACGCGAATCCAAAGTGA

TGAAGAAGTGCAGATAGATATTCGTTCACCATATAGAGGAGAATATATCATTCCTACAAA

CAAAATTGATGATGTTTCTAGAATAGAAACGTTAGAATCGAATATTCATCTGTTCCGGGT

GGGGAAGGGCTACTGAAAAGCTGAACTTTTTTAAGAGTTCGAACTTCGAAAGTCAATCAA

TGATTCTTAATTGAGAGGGAAAAGCTATAAAGGACGAGAAGGGAAGGAAATGTTTATAGG

GAATGAATTTCTAATGCATGATGGGAATGACAGATATGAGGAATATATAAAGGTGATTCT

TATATACATTGACATGGATCGGAATCTATTAACTTAAAGTTATTGGGGTCGTGAAATTTA

TTTAATTTTTCTAATTCGTGTCACATGTCACGACAATTAGTGGGG-AACATTTATGTATA

TAGAAATTTCAGAAATTTCTAGCAGCGTGATAAATATAAAGATTTTGGCACAATAAGTTT

TTGGATCATAATGAGTTT-CTATTTAATCAA-GGCT-TGTAA-TCTATTTTATTATTTAG

-TAGGG-AAACTGAAAACTTAGGGTTGCTATTTGTAGCTCCCACCCTTCTTAGTTCTTAC

TCTTTTCAATATTTTTTATAAGTTTTA-TATAATACCT-AAATT-GCCCTCCTCCTCA-C

CCCCCCTGCTAATCTTCT--CTCT-CACACATTGCTTCT-CACCCCATTGCTCT-CCGTT

GCTCCTCCATGTG-AGTTCTAGTCTTTTTTTTT-CTCTGGTAGCGTTTTGCTGCTCCTTT

TTTCACTCAAGTGTTGCCAATTAA-TTGACAAAAAATGGTTTCTGTTTCATATAGAAACT

ATGTTTTTGCTGTGTAGTCATACATTACGGAATCTAGTTT-CCATTAAATAAGTAACATG

TGAAAAAAAA--TAAAAGGTGAAATATATATTGTTGGAAAAGAAGCTATGAGGTGCAAGA

ACCGATCACATGGAGAAGGCAATGAAAGACAAGGAGGAGCAATGGAAGA-----------

---------GAGAAAATGAGAAGATGGAAGGGATGTGAAAATGTTTGAAAAAGACGAGGT

GATCAGTTTTGAAATACGAATTTAGTATTTTCTTTTTAAGAAAATTCTTTCG-AAAGTCG

TGTTTTAAAACATGACTTTTATT-ATTTGAAGTC----TGTTCTAAAACATGACTTA--T

TCATATCCTT-AATATTTTT---------AAAATTTATCCATTTGTAATATTTTTTAAAA

ATTGACCCATATATGTAAAATACCCGTCAAGATCTCTTTATTATTTTGAAAGCGAAAGCA

TATCACTTCAAACACAATGGAATCGAGGCTATTGACTAAGTATAAATAGAGAAGACTTCA

TATCGGGGTTCATAATTCATAACAAAGCAAACGAGTATATAAG----------CCAAATT

TTGAGTAAACTAGTGTGCACACTATCCCATGCCTAGTGGAAGTAGGGATCCTCTCGTTGT

TGGGGGAGTAATTGGGGATGTATTGGATCCTTTTGATTATTCTATTCCTATGAGGGTTAC

CTACAATAACAGAGATGTCAGCAATGGATGTGAATTCAAACCCTCACAAGTTGTCAACCA

ACCAAGGGTAAATATCGGTGGTGATGACC

>CS51.G2

TATTAGACTCCTAATTTGATATTCTTGTTTTATTATAATGCTGATAAGTCTTGTAAATAA

GGGTGAGAAGCACGAATAATTAGTTCATGAGATGTGTATAATTATTATCCTACACGACTT

ATCTTTGATATTTCACACAAGTCTTTCAATGTATAACAAAAATTTTTTAGATACATTTAG

ACTATAGAACTAACAAGTTATATTTTGAACC-AAAAAACAAAGGGAGAGAG----GAACA

TAGAGAAAAGAAGATATGAGAGTTTTTTTTTTTTTTT----CTAAAACAGAAAGAAACTC

ATTATATAATAAACAAATTAATTTGAGACAAATTAACGTATGTAATAAAACAAAATCAAA

GTGGTAATTAAAATTATTTAATGGTAAACAGTCTAATAGTTAAAATAAAAATGGAAATCA

CATAAATTTTGTAATTGGCCCATTAAAAACAACACTAAGCTTTTAATTTGATTTTGAAAT

TCAAAATAATTTTATTAAATCACAAAGTAAAAGGTTTACAAAGCCGATCATGACAGTGCA

TGTGGGAGGCAAATCGGCATATTTGCACTACAAAAGG-ACCCATGAAGTCTCTGAACATG

CACGCAACACTTTAATCTCTTATTAGTTACTTTGAAAGCTTATTTATATATATAGACACG

CGTAAAAACTTCTTAACCAAGATTTTTTT-ACGTGCTTCC-TTCGCGTTTAATTTGGACC

ATCAAACCGTGCTCAACAGATAAAGAAAAGGGTGCTTTTGATTCAAGATATTGGCC-GAA

AAACACAAGATAGATCCTT-CGATAGATTAAGCCACGCATGAAACGCGAATCCAAAGTGA

TGAAGAAGTGCAGATAGATATTCGTTCACCATATAGAGGAGAATATATCATTCCTACAAA

CAAAATTGATGATGTTTCTAGAATAGAAACGTTAGAATCGAATATTCATCTGTTCCGGGT

GGGGAAGGGCTACTGAGAAGCTGAACTTTTTTAAGAGTTCGAACTTCGAAAGTCAATCAA

TGATTCTTAATTGAGAGGGAAAAGCTATAAAGGACGAGAAGGGAAGGAAATGTTTATAGG

GAATGAATTTCTAATGCATGATGGGAATGACAGATATGAGGAATATATAAAGGTGATTCT

TATATACATTGACATGGATCGGAATCTATTAACTTAAAGTTATTGGGGTCGTGAAATTTA

TTTAATTTTTCTAATTCGTGTCACATGTCACGACAATTAGTGGGG-AACATTTATGTATA

TAGAAATTTCAGAAATTTCTAGCAGCGTGATAAATATAAAGATTTTGGCACAATAAGTTT

TTGGATCATAATGAGTTT-CTATTTAATCAA-GGCA-TGTAA-TCTATTTTGTTATTTAG

-TAGGG-AAACTGAAAACTTAGGGTTGCTATTTGTAGCTCCCACCCTTCTTAGTTCTTAC

TCTTTTCAATATTTTTTATAAGTTTTA-TATAATACCT-AAATT-GCCCTCCTCCTCACC

CCCCCCTGCTAATCTTCTTCCTCCTCACACATTGCTTCT-CACCCCATTGCTCTTCCGTT

GCTCCTCCATGTG-AGTTCTAGTCTTTTTTTT--CTCTGGTAGCGTTTTGCTGCTCCTTT

TTTCACTCAAGTGTTGCCAATTAA-TTGACAAAAAATGGTTTCTGTTTCATATAGAAACT

ATGTTTTTGTTGTGTAGTCATACATTACGGAATCTAGTTT-CCATTAAATAAGTAACATG

TGAAAAAAAA--TAAAAGGTGAAATATATATTGTTGGAAAAGAAGCTATGAGGTGCAAGA

ACCGATCACATGGAGAGGGCAATGAAAGACAAGGAGGAGCAATGGAAGA-----------

---------GAGAAAATGAGAAGATGGAAGGGATGTGAAAATGTTTGAAAAAGACGAGGT

GATCAGTTTTGAAATACGAATTTAGTATTTTCTTTTTAAGAAAATTCTTTCG-AAAGTCG

TGTTTTAAAACATGACTTTTATT-ATTTGAAGTC----TGTTCTAAAACATGACTTA--T

TCATATCCTT-AATATTTTT---------AAAATTTATCCATTTGTAATATTTTTTAAAA

ATTGACCCATATATGTAAAATACCCGTCAAGATCTCTTTATTATTTTGAAAGCGAAAGCA

TATCACTTCAAACACAATGGAATCGAGGCTATTGACTAAGTATAAATAGAGAAGACTTCA

TATCGGGGTTCATAATTCATAACAAAGCAAACGAGTATATAAG----------CCAAATT

TTGAGTAAACTAGTGTGCACACTATCCCATGCCTAGTGGAAGTAGGGATCCTCTCGTTGT

TGGGGGAGTAATTGGGGATGTATTGGATCCTTTTGAATATTCTATTCCTATGAGGGTTAC

CTACAATAACAGAGATGTCAGCAATGGATGTGAATTCAAACCCTCACAAGTTGTCAACCA

ACCAAGGGTAAATATCGGTGGTGATGACC

>CS42.92

TATTAGACTCCTAATTTGATATTCTTGTTTTATTATAATGCTGATAAGTCTTGTAAATAA

GGGTGAGAAGCACGAATAATTAGTTCATGAGATGTGTATAATTATTATCCTACACGACTT

ATCTTTGATATTTCACACAAGTCCTTCAATGTATAACAAAAATTTTTTAGATACATTTAG

ACTATAGAACTAACAAGTTATATTTTGAACC-AAAAAACAAAGAGAGAGAG----GAACA

TAGAGAAAAGAAGATATGAGAGTTTTTTTTTTTTTT-----CTAAAACAGAAAGAAACTC

ATTATATAATAAACAAATTAATTTGAGACAAATTAACGTATGTAATAAAACAAAATCAAA

GCGGTAATTAAAATTATTTAATGGTAAACAGTCTAATAGTTAAAATAAAAATGGAAATCA

CATAAATTTTGTAATTGGCCCATTAAAAACAACACTAAGCTTTTAATTTGATTTTGAAAT

TCAAAATAATTTTATTAAATCACAAAGTAAAAGGTTTACAAAGCCGATCATGACAGTGCA

TGTGGGAGGCAAATCGGCATATTTGCACTACAAAAGG-ACCCATGAAGTCTCTGAACATG

CACGCAACACTTTAATCTCTTATTAGTTACTTTGAAAGCTTATTTATATATATAGACACG

CGTAAAAACTTCTTAACCAAGATTTTTTT-ACGTGCTTCC-TTCGCGTTTAATTTGGACC

ATCAAACCGTGCTCAACAGATAAAGAAAAGGGTGCTTTTGATTCAAGATATTGGCC-GAA

AAACACAAGATAGATCCTT-CGATAGATTAAGCCACGCATGAAACGCGAATCCAAAGTGA

TGAAGAAGTGCGGATAGATATTCGTTCACCATATAGAGGAGAATATATCATTCCTACAAA

CAAAATTGATGATGTTTCTAGAATAGAAACGTTAGAATCGAATATTCATCTGTTCCGGGT

GGGGAAGGGCTACTGAAAAGCTGAACTTTTTTAAGGGTTCGAACTTCGAAAGTCAATCAA

TGATTCTTAATTGAGAGGGAAAAGCTATAAAGGACGAGAAGGGAAGGAAATGTTTATAGG

GAATGAATTTCTAATGCATGATGGGAATGACAGATATGAGGAATATATAAAGGTGATTCT

TATATACATTGACATGGATCGGAATCTATTAACTTAAAGTTATTGGGGTCGTGAAATTTA

TTTAATTTTTCTAATTCGTGTCACATGTCACGACAATTAGTGGGG-AACATTTATGTATA

TAGAAATTTCAGAAATTTCTAGCAGCGTGATAAATATAAAGATTTTGGCACAATAAGTTT

TTGGATCATAATGAGTTT-CTATTTAATCAA-GGCA-TGTAA-TCTATTTTATTATTTAG

-TAGGG-AAACTGAAAACTTAGGGTTGCTATTTGTAGCTCCCACCCTTCTTAGTTCTTAC

TCTTTCCAATATTTTTTATAAGTTTTA-TATAATACCT-AAATT-GCCCTCCTCCTCACC

CCCCCCTGCTAATCTTCTTCCTCCTCACACATTGCTTCT-CACCCCATTGCTCTTCCGTT

GCTCCTCCATGTG-AGTTCTAGTCTTTTTTTTT-CTCTGGTAGCGTTTTGCTGCTCCTTT

TTTCACTCAAGTGTTGCCAATTAA-TTGACAAAAAATGGTTTCTGTTTCATATAGAAACT

ATGTTTTTGTTGTGTAGTCATACATTACGGAATCTAGTTT-CCATTAAATAAGTAACATG

TGAAAAAAAA--TAAAAGGTGAAATATATATTGTTGGAAAAGAAGCTATGAGGTGCAAGA

ACCGATCACATGGAGAAGGCAATGAAAGACAAGGAGGAGCAATGGAAGA-----------

---------GAGAAAATGAGAAGATGGAAGGGATGTGAAAATGTTTGAAAAAGACGAGGT

GATCAGTTTTGAAATACGAATTTAGTATTTTCTTTTTAAGAAAATTCTTTCG-AAAGTCG

TGTTTTAAAACATGACTTTTATT-ATTTGAAGTC----TGTTCTAAAACATGACTTA--T

TCATATCCTT-AATATTTTT---------AAAATTTATCCATTTGTAATATTTTTTAAAA

ATTGACCCATATATGTAAAATACCCGTCAAGATCTCTTTATTATTTTGAAAGCGAAAGCA

TATCACTTCAAACACAATGGAATCGAGGCTATTGACTAAGTATAAATAGAGAAGACTTCA

TATCGGGGTTCATAATTCATAACAAAGCAAACGAGTATATAAG----------CCAAATT

TTGAGTAAACTAGTGTGCACACTATCCCATGCCTAGTGGAAGTAGGGATCCTCTCGTTGT

TGGGGGAGTAATTGGGGATGTATTGGATCCTTTTGAATATTCTATTCCTATGAGGGTTAC

CTACAATAACAGAGATGTCAGCAATGGATGTGAATTCAAACCCTCACAAGTTGTCAACCA

ACCAAGGGTAAATATCGGTGGTGATGACC

>CS34.34

TATTAGACTCCTAATTTGATATTCTTGTTTTATTATAATGCTGATAAGTCTTGTAGATAA

GGGTGAGAAGCACGAACAATTAGTTCATGAGATGTGTATAATTATTATCCTACACGACTT

ATCTTTGATATTTCACACAAGTCTTTCAATGTATAACAAAAATTTTTTAGATACATTTAG

ACTATAGAACTAACAAGTTATATTTTGAACC-AAAAAACAAAGAGAGAGAG----GAACA

TAGAGAAAAGAAGATATGAGAGTTTTTTTTTTTTTTT----CTAAAACAGAAAGAAACTC

ATTATATAATAAACAAATTAATTTGAGACAAATTAACGTATGTAATAAAACAAAATCAAA

GTGGTAATTAAAATTATTTAATGGTAAACAGTCTAATAGTTAAAATAAAAATGGAAATCA

CATAAATTTTGTAATTGGCCCATTAAAAACAACACTAAGCTTTTAATTTGATTTTGAAAT

TCAAAATAATTTTATTAAATCACAAAGTAAAAGGTTTACAAAGCCGATCATGACAGTGCA

TGTGGGAGGCAAATCGGCATATTTGCACTACAAAAGG-ACCCATGAAGTCTCTGAACATG

CACGCAACACTTTAATCTCTTATTAGTTACTTTGAAAGCTTATTTATATATATAGACACG

CGTAAAAACTTCTTAACCAAGATTTTTTT-ACGTGCTTCC-TTCGCGTTTAATTTGGACC

ATCAAACCGTGCTCAACAGATAAAGAAAAGGGTGCTTTTGATTCAAGATATTGGCC-GAA

AAACACAAGATAGATCCTT-CGATAGATTAAGCCACGCATGAAACGCGAATCCAAAGTGA

TGAAGAAGTGCAGATAGATATTCGTTCACCATATAGAGGAGAATATATCATTCCTACAAA

CAAAATTGATGATGTTTCTAGAATAGAAACGTTAGAATCGAATATTCATCTGTTCCGGGT

GGGGAAGGGCTACTGAAAAGCTGAACTTTTTTAAGAGTTCGAACTTCGAAAGTCAATCAA

TGATTCTTAATTGAGAGGGAAAAGCTATAAAGGACGAGAAGGGAAGGAAATGTTTATAGG

GAATGAATTTCTAATGCATGATGGGAATGACAGATATGAGGAATATATAAAGGTGATTCT

TATATACATTGACATGGATCGGAATCTATTAACTTAAAGTTATTGGGGTCGTGAAATTTA

TTTAATTTTTCTAATTCGTGTCACATGTCACGACAATTAGTGGGA-AACATTTATGTATA

TAGAGATTTCAGAAATTTCTAGCAGCGTGATAAATATAAAGATTTTGGCACAATAAGTTT

TTGGATCATAATGAGTTT-CTATTTAATCAA-GGCA-TGTAA-TCTATTTTATTATTTAG

-TAGGG-AAACTGAAAACTTAGGGTTGCTATTTGTAGCTCCCACCCTTCTTAGTTCTTAC

TCTTTTCAATATTTTTTATAAGTTTTA-TATAATACCT-AAATT-GCCCTCCTCCTCA-C

CCCCCCTGCTAATCTTCTT-CTCCTCACACATTGCTTCT-CACCCCATTGCTCT-CCGTT

GCTCCTCCATGTG-AGTTCTAGTCTTTTTTTT--CTCTGGTAGCGTTTTGCTGCTCCTTT

TTTCACTCAAGTGTTGCCAATTAA-TTGACAAAAAATGGTTTCTGTTTCATATAGAAACT

ATGTTTTTGTTGTGTAGTCATACATTACGGAATCTAGTTT-CCATTAAATAAGTAACATG

TGAAAAAAAA--TAAAAGGTGAAATATATATTGTTGGAAAAGAAGCTATGAGGTGCAAGA

ACCGATCACATGGAGAAGGCAATGAAAGACAAGGAGGAGCAATGGAAGA-----------

---------GAGAAAATGAGAAGATGGAAGGGATGTGAAAATGTTTGAAAAAGACGAGGT

GATCAGTTTTGAAATACGAATTTAGTATTTTCTTTTTAAGAAAATTCTTTCG-AAAGTCG

TGTTTTAAAACATGACTTTTATT-ATTTGAAGTC----TGTTCTAAAACATGACTTA--T

TCATATCCTT-AATATTTTT---------AAAATTTATCCATTTGTAATATTTTTTAAAA

ATTGACCCATATATGTAAAATACCCGTCAAGATCTCTTTATTATTTTGAAAGCGAAAGCA

TATCACTTCAAACACAATGGAATCGAGGCTATTGACTAAGTATAAATAGAGAAGACTTCA

TATCGGGGTTCATAATTCATAACAAAGCAAACGAGTATATAAG----------CCAAATT

TTGAGTAAACTAGTGTGCACACTATCCCATGCCTAGTGGAAGTAGGGATCCTCTCGTTGT

TGGGGGAGTAATTGGGGATGTATTGGATCCTTTTGAATATTCTATTCCTATGAGGGTTAC

CTACAATAACAGAGATGTCAGCAATGGATGTGAATTCAAACCCTCACAAGTTGTCAACCA

ACCAAGGGTAAATATCGGTGGTGATGACC

>J2.22

TATTAGACTCCTAATTTGATATTCTTGTTTTATTATAATGCTGATAAGTCTTGTAAATAA

GGGTGAGAAGCACGAATAATTAGTTCATGAGATGTGTATAATTATTATCCTACACGACTT

ATCTTTGATATTTCACACAAGTCTTTCAATGTATAACAAAAATTTTTTAGATACATTTAG

ACTATAGAACTAACAAGTTATATTTTGAACC-AAAAAACAAAGAGAGAGAG----GAACA

TAGAGAAAAGAAGATATGAGAGTTTTTTTTTTTTT------CTAAAACAGAAAGAAACTC

ATTATATAATAAACAAATTAATTTGAGACAAATTAACGTATGTAATAAAACAAAATCAAA

GTGGTAATTAAAATTATTTAATGGTAAACAGTCTAATAGTTAAAATAAAAATGGAAATCA

CATAAATTTTGTAATTGGCCCATTAAAAACAACACTAAGCTTTTAATTTGATTTTGAAAT

TCAAAATAATTTTATTAAATCACAAAGTAAAAGGTTTACAAAGCCGATCATGACAGTGCA

TGTGGGAGGCAAATCGGCATATTTGCACTACAAAAGG-ACCCATGAAGTCTCTGAACATG

CACGCAACACTTTAATCTCTTATTAGTTACTTTGAAAGCTTATTTATATATATAGACACG

CGTAAAAACTTCTTAACCAAGATTTTTTT-ACGTGCTTCC-TTCGCGTTTAATTTGGACC

ATCAAACCGTGCTCAACAGATAAAGAAAAGGGTGCTTTTGATTCAAGATATTGGCC-GAA

AAACACAAGATAGATCCTT-CGATAGATTAAGCCACGCATGAAACGCGAATCCAAAGTGA

TGAAGAAGTGCAGATAGATATTCGTTCACCATATAGAGGAGAATATATCATTCCTACAAA

CAAAATTGATGATGTTTCTAGAATAGAAACGTTAGAATCGAATATTCATCTGTTCCGGGT

GGGGAAGGGCTACTGAAAAGCTGAACTTTTTTAAGAGTTCGAACTTCGAAAGTCAATCAA

TGATTCTTAATTGAGAGGGAAAAGCTATAAAGGACGAGAAGGGAAGGAAATGTTTATAGG

GAATGAATTTCTAATGCATGATGGGAATGACAGATATGAGGAATATATAAAGGTGATTCT

TATATGCATTGACATGGATCGGAATCTATTAACTTAAAGTTATTGGGGTCGTGAAATTTA

TTTAATTTTTCTAATTCGTGTCACATGTCACGACAATTAGTGGGG-AACATTTATGTATA

TAGAAATTTCAGAAATTTCTAGCAGCGTGATAAATATAAAGATTTTGGCACAATAAGTTT

TTGGATCATAATGAGTTT-CTATTTAATCAA-GGCA-TGTAA-TCTATTTTATTATTTAG

-TAGGG-AAACTGAAAACTTAGGGTTGCTATTTGTAGCTCCCACCCTTCTTAGTTCTTAC

TCTTTTCAATATTTTTTATAAGTTTTA-TATAATACCT-AAATT-GCCCTCCTCCTCACC

CCCCCCTGCTAATCTTCTTCCTCCTCACACATTGCTTCT-CACCCCATTGCTCTTCCGTT

GCTCCTCCATGTG-AGTTCTAGTCTTTTTTTTT-CTCTGGTAGCGCTTTGCTGCTCCTTT

TTTCACTCAAGTGTTGCCAATTAA-TTGACAAAAAATGGTTTCTGTTTCATATAGAAACT

ATGTTTTTGTTGTGTAGTCATACATTACGGAATCTAGTTT-CCATTAAATAAGTAACATG

TGAAAAAAAA--TAAAAGGTGAAATATATATTGTTGGAAAAGAAGCTATGAGGTGCAAGA

ACCGATCACATGGAGAAGGCAATGAAAGACAAGGAGGAGCAATGGAAGA-----------

---------GAGAAAATGAGAAGATGGAAGGGATGTGAAAATGTTTGAAAAAGACGAGGT

GATCAGTTTTGAAATACGAATTTAGTATTTTCTTTTTAAGAAAATTCTTTCG-AAAGTCG

TGTTTTAAAACATGACTTTTATT-ATTTGAAGTC----TGTTCTAAAACATGACTTA--T

TCATATCCTT-AATATTTTT---------AAAATTTATCCATTTGTAATATTTTTTAAAA

ATTGACCCATATATGTAAAATACCCGTCAAGATCTCTTTATTATTTTGAAAGCGAAAGCA

TATCACTTCAAACACAATGGAATCGAGGCTATTGACTAAGTATAAATAGAGAAGACTTCA

TATCGGGGTTCATAATTCATAACAAAGCAAACGAGTATATAAGAAAGCATAAGCCAAATT

TTGAGTAAACTAGTGTGCACACTATCCCATGCCTAGTGGAAGTAGGGATCCTCTCGTTGT

TGGGGGAGTAATTGGGGATGTATTGGATCCTTTTGAATATTCTATCCCTATGAGGGTTAC

CTACAATAACAGAGATGTCAGCAATGGATGTGAATTCAAACCCTCACAAGTTGTCAACCA

ACCAAGGGTAGATATCGGTGGTGATGACC

>J2.23

TATTAGACCCCTAATTTGATATTCTTGTTTTATTATAATGCTGATAAGTCTTGTAAATAA

GGGTGAGAAGCACGAATAATTAGTTCATGAGATGTGTATAATTATTATCCTACACGACTT

ATCTTTGATATTTCACACAAGTCTTTCAATGTATAACAAAAATTTTTTAGATACATTTAG

ACTATAGAACTAACAAGTTATATTTTGAACC-AAAAAACAAAGAGAGAGAG----GAACA

TAGAGAAAAGAAGATATGAGAGTTTTTTTTTTTTT------CTAAAACAGAAAGAAACTC

ATTATATAATAAACAAATTAATTTGAGACAAATTAACGTATGTAATAAAACAAAATCAAA

GTGGTAATTAAAATTATTTAATGGTAAACAGTCTAATAGTTAAAATAAAAATGGAAATCA

CATAAATTTTGTAATTGGCCCATTAAAAACAACACTAAGCTTTTAATTTGATTTTGAAAT

TCAAAATAATTTTATTAAATCACAAAGTAAAAGGTTTACAAAGCCGATCATGACAGTGCA

TGTGGGAGGCAAATCGGCATATTTGCACTACAAAAGG-ACCCATGAAGTCTCTGAACATG

CACGCAACACTTTAATCTCTTATTAGTTACTTTGAAAGCTTATTTATATATATAGACACG

CGTAAAAACTTCTTAACCAAGATTTTTTT-ACGTGCTTCC-TTCGCGTTTAATTTGGACC

ATCAAACCGTGCTCAACAGATAAAGAAAAGGGTGCTTTTGATTCAAGATATTGGCC-GAA

AAACACAAGATAGATCCTT-CGATAGATTAAGCCACGCATGAAACGCGAATCCAAAGTGA

TGAAGAAGTGCAGATAGATATTCGTTCACCATATAGAGGAGAATATATCATTCCTACAAA

CAAAATTGATGATGTTTCTAGAATAGAAACGTTAGAATCGAATATTCATCTGTTCCGGGT

GGGGAAGGGCTACTGAAAAGCTGAACTTTTTTAAGAGTTCGAACTTCGAAAGTCAATCAA

TGATTCTTAATTGAGAGGGAAAAGCTATAAAGGACGAGAAGGGAAGGAAATGTCTATAGG

GAATGAATTTCTAATGCATGATGGGAATGACAGATATGAGGAATATATAAAGGTGATTCT

TATATACATTGACATGGATCGGAATCTATTAACTTAAAGTTATTGGGGTCGTGAAATTTA

TTTAATTTTTCTAATTCGTGTCACATGTCACGACAATTAGTGGGG-AACATTTATGTATA

TAGAAATTTCAGAAATTTCTAGCAGCGTGATAAATATAAAGATTTTGGCACAATAAGTTT

TTGGATCATAATGAATTT-CTATTTAATCAA-GGCA-TGTAA-TCTATTTTATTATTTAG

-TAGGG-AAACTGAAAACTTAGGGTTGCTATTTGTAGCTCCCACCCTTCTTAGTTCTTAC

TCTTTTCAATATTTTTTATAAGTTTTA-TATAATACCT-AAATT-GCCCTCCTCCTCACC

CCCCCCTGCTAATCTTCTTCCTC-TCACACATTGCTTCT-CACCCCATTGCTCTTCCGTT

GCTCCTCCATGTG-AGTTCTAGTCTTTTTTTTT-CTCTGGTAGCGTTTTGCTGCTCCTTT

TTTCACTCAAGTGTTGCCAATTAA-TTGACAAAAAATGGTTTCTGTTTCATATAGAAACT

ATGTTTTTGTTGTGTAGTCATGCATTACGGAATCTAGTTT-CCATTAAATAAGTAACATG

TGAAAAAAAA--TAAAAGGTGAAATATATATTGTTGGAAAAGAAGCTATGAGGTGCAAGA

ACCGATCACATGGAGAAGGCAATGAAAGACAAGGAGGAGCAATGGAAGA-----------

---------GAGAAAATGAGAAGATGGAAGGGATGTGAAAATGTTTGAAAAAGACGAGGT

GATCAGTTTTGAAATACGAGTTTAGTATTTTCTTTTTAAGAAAATTCTTTCG-AAAGTCG

TGTTTTAAAACATGACTTTTATT-ATTTGAAGTC----TGTTCTAAAACATGACTTA--T

TCATATCCTT-AATATTTTT---------AAAATTTATCCATTTGTAATATTTTTTAAAA

ATTGACCCATATATGTAAAATACCCGTCAAGATCTCTTTATTATTTTGAAAGCGAAAGCA

TATCACTTCAAACACAATGGAATCGAGGCTATTGACTAAGTATAAATAGAGAAGACTTCA

TATCGGGGTTCATAATTCATAACAAAGCAAACGAGTATATAAGAAAGCATAAGCCAAATT

TTGAGTAAACTAGTGTGCACACTATCCCATGCCTAGTGGAAGTAGGGATCCTCTCGTTGT

TGGGGGAGTAATTGGGGATGTATTGGATCCTTTTGAATATTCTATTCCTATGAGGGTTAC

CTACAATAACAGAGATGTCAGCAATGGATGTGAATTCAAACCCTCACAAGTTGTCAACCA

ACCAAGGGTAAATATCGGTGGTGATGACC

>H02.01

TATTAGACTCCTAATTTAATATTCTTGTTTTATTATAATGCTGATAAGTCTTGTAAATAA

GGGTGAGAAGCACGAATAATTAGTTCATGAGATGTGTATAATTATTATCCTACACGACTT

ATCTTTGATATTTCACACAAGTCTTTCAATGTATAACAAAAAATTTTTAGATACATTTAG

ACTATAGAACTAACAAGTTATATTTTGAACC-AAAAAACAAAGAGAGAGAG----GAACA

TAGAGAAAAGAAGATATGAGAGTTTTTTTTTTTTTTTTT--CTAAAACAGAAAGAAACTC

ATTATATAATAAACAAATTAATTTGAGACAAATTAACGTATGTAATAAAACAAAATCAAA

GTGGTAATTAAAATTATTTAATGGTAAACAGTCTAATAGTTGAAATAAAAATGGAAATCA

CATAAATTTTGTAATTGGCCCATTAAAAACAACACTAAGCTTTTAATTTGATTTTGAAAT

TCAAAATAATTTTATTAAATCACAAAGTAAAAGGTTTACAAAGCCGATCATGACAGTGCA

TGTGGGAGGCAAATCGGCATATTTGCACTACAAAAGG-ACCCATGAAGTCTCTGAACATG

CACGCAACACTTTAATCTCTTATTAGTTACTTTGAAAGCTTATTTATATATATAGACACG

CGTAAAAACTTCTTAACCAAGATTTTTTT-ACGTGCTTCC-TTCGCGTTTAATTTGGACC

ATCAAACCGTGCTCAACAGATAAAGAAAAGGGTGCTTTTGATTCAAGATATTGGCC-GAA

AAACACAAGATAGATCCTT-CGATAGATTAAGCCACGCATGAAACGCGAATCCAAAGTGA

TGAAGAAGTGCAGATAGATATTCGTTCACCATATAGAGGAGAATATATCATTCCTACAAA

CAAAATTGATGATGTTTCTAGAATAGAAACGTTAGAATCGAATATTCATCTGTTCCGGGT

GGGGAAGGGCTACTGAAAAGCTGAACTTTTTTAAGAGTTCGAACTTCGAAAGTCAATCAA

TGATTCTTAATTGGGAGGGAAAAGCTATAAAGGACGAGAAGGGAAGGAAATGTTTATAGG

GAATGAATTTCTAATGCATGATGGGAATGACAGATATGAGGAATATATAAAGGTGATTCT

TATATACATTGACATGGATCGGAATCTATTAACTTAAAGTTATTGGGGTCGTGAAATTTA

TTTAATTTTTCTAATTCGTGTCACATGTCACGACAATTAGTGGGG-AACATTTATGTATA

TAGAAATTTCAGAAATTTCTAGCAGCGTGATAAATATAAAGATTTTGGCACAATAAGTTT

TTGGATCATAATGAGTTT-CTATTTAATCAA-GGCA-TGTAA-TCTATTTTATTATTTAG

-TAGGG-AAACTGAAAACTTAGGGTTGCTATTTGTAGCTCCCACCCTTCTTAGTTCTTAC

TCTTTTCAATATTTTTTATAAGTTTTA-TATAATACCT-AAATT-GCCCTCCTCCTCA-C

CCCCCCTGCTAATCTTCTTCCTCCTCACACATTGCTTCT-CACCCCATTGCTCTTCCGTT

GCTCCTCCATGTG-AGTTCTAGTCTTTTTTTTT-CTCTGGTAGCGTTTTGCTGCTCCTTT

TTTCACTCAAGTGTTGCCAATTAA-TTGACAAAAAATGGTTTCTGTTTCATATAGAAACT

ATGTTTTTGTTGTGTAGTCATACATTACGGAAACTAGTTT-CCATTAAATAAGTAACATG

TGAAGAAAAA--TAAAAGGTGAAATATATATTGTTGGAAAAGAAGCTATGAGGTGCAAGA

ACCGATCACATGGAGAAGGCAATGAAAGACAAGGAGGAGCAATGGAAGA-----------

---------GAGAAAATGAGAAGATCGAAGGGATGTGAAAATGTTTGAAAAAGACGAGGT

GATCAGTTTTGAAATACGAATTTAGTATTTTCTTTTTAAGAAAATTCTTTCG-AAAGTCG

TGTTTTAAAACATGACTTTTATT-ATTTGAAGTCG---TGTTCTAAAACATGACTTA--T

TCATATCCTT-AATATTTTT---------AAAATTTATCCATTTGTAATATTTTTTAAAA

ATTGACCCATATATGTAAAATACCCGTCAAGATCTCTTTATTATTTTGAAAGCGAAAGCA

TATCACTTCAAACACAATGGAATCGAGGCTATTGACTAAGTATAAATAGAGAAGACTTCA

TATCGGGGTTCATAATTCATAACAAAGCAAACGAGTATATAAGAAAGCATAAGCCAAATT

TTGAGTAAACTAGTGTGCACACTATCCCATGCCTAGTGGAAGTAGGGATCCTCTCGTTGT

TGGGGGAGTAATTGGGGATGTATTGGATCCTTTTGAATATTCTATTCCTATGAGGGTTAC

CTACAATAACAGAGATGTCAGCAATGGATGTGAATTCAAACCCTCACAAGTTGTCAACCA

ACCAAGGGTAAATATCGGTGGTGATGACC

>H02.07c

TATTAGACTCCTAATTTAATATTCTTGTTTTATTATAATGCTGATAAGTCTTGTAAATAA

GGGTGAGAAGCACGAATAATTAGTTCATGAGATGTGTATAATTATTATCCTACACGACTT

ATCTTTGATATTTCACACAAGTCTTTCAATGTATAACAAAAAATTTTTAGATACATTTAG

ACTATAGAACTAACAAGTTATATTTTGAACC-AAAAAACAAAGAGAGAGAG----GAACA

TAGAGAAAAGAAGATATGAGAGTTTTTTTTTTTTTTTTTT-CTAAAACAGAAAGAAACTC

ATTATATAATAAACAAATTAATTTGAGACAAATTAACGTATGTAATAAAACAAAATCAAA

GTGGTAATTAAAATTATTTAATGGTAAACAGTCTAATAGTTAAAATAAAAATGGAAATCA

CATAAATTTTGTAATTGGCCCATTAAAAACAACACTAAGCTTTTAATTTGATTTTGAAAT

TCAAAATAATTTTATTAAATCACAAAGTAAAAGGTTTACAAAGCCGATCATGACAGTGCA

TGTGGGAGGCAAATCGGCATATTTGCACTACAAAAGG-ACCCATGAAGTCTCTGAACATG

CACGCAACACTTTAATCTCTTATTAGTTACTTTGAAAGCTTATTTATATATATAGACACG

CGTAAAAACTTCTTAACCAAGATTTTTTT-ACGTGCTTCC-TTCGCGTTTAATTTAGACC

ATCAAACCGGGCTCAACAGATAAAGAAAAGGGTGCTTTTGATTCAAGATATTGGCC-GAA

AAACACAAGATAGATCCTT-CGATAGATTAAGCCACGCATGAAACGCGAATCCAAAGTGA

TGAAGAAGTGCAGATAGATATTCGTTCACCATATAGAGGAGAATATATCATTCCTACAAA

CAAAATTGATGATGTTTCTAGAATAGAAACGTTAGAATCGAATATTCATCTGTTCCGGGT

GGGGAAGGGCTACTGAAAAGCTGAACTTTTTTAAGAGTTCGAACTTCGAAAGTCAATCAA

TGATTCTTAATTGAGAGGGAAAAGCTATAAAGGACGAGAAGGGAAGGAAATGTTTATAGG

GAATGAATTTCTAATGCATGATGGGAATGACAGATATGAGGAATATATAAAGGTGATTCT

TATATACATTGACATGGATCGGAATCTATTAACTTAAGGTTATTGGGGTCGTGAAATTTA

TTTAATTTTTCTAATTCGTGTCACATGTCACGACAATTAGTGGGG-AACATTTATGTATA

TAGAAATTTCAGAAATTTCTAGCAGCGTGATAAATATAAAGATTTTGGCACAATAAGTTT

TTGGATCATAATGAGTTT-CTATTTAATCAA-GGCA-TGTAA-TCTATTTTATTATTTAG

-TAGGG-AAACTGAAAACTTAGGGTTGCTATTTGTAGCTCCCACCCTTCTTAGTTCTTAC

TCTTTTCAATATTTTTTATAAGTTTTA-TATAATACCT-AAATT-GCCCTCCTCCTCA-C

CCCCCCTGCTAATCTTCTTCCTCCTCACACATTGCTTCT-CACCCCATTGCTCTTCCGTT

GCTCCTCCATGTG-AGTTCTAGTCTTTTTTTTT-CTCTGGTAGCGTTTTGCTGCTCCTTT

TTTCACTCAAGTGTTGCCAATTAA-TTGACAAAAAATGGTTTCTGTTTCATATAGAAACT

ATGTTTTTGTTGTGTAGTCATACATTACGGAAACTAGTTT-CCATTAAATAAGTAACATG

TGAAAAAAAAA-TAAAAGGTGAAATATATATTGTTGGAAAAGAAGCTATGAGGTGCAAGA

ACCGATCACATGGAGAAGGCGATGAAAGACAAGGAGGAGCAATGGAAGA-----------

---------GAGAAAATGAGAAGATGGAAGGGATGTGAAAATGTTTGAAAAAGACGAGGT

GATCAGTTTTGAAATACGAATTTAGTATTTTCTTTTTAAGAAAATTCTTTCG-AAAGTCG

TGTTTTAAAACATGACTTTTATT-ATTTGAAGTCG---TGTTCTAAAACATGACTTA--T

TCATATCCTT-AATATTTTT---------AAAATTTATCCATTTGTAATATTTTTTAAAA

ATTGACCCATATATGTAAAATACCCGTCAAGATCTCTTTATTATTTTGAAAGCGAAAGCA

TATCACTTCAAACGCAATGGAATCGAGGCTATTGACTAAGTATAAATAGGGAAGACTTCA

TATCGGGGTTCATAATTCATAACAAAGCAAACGAGTATATAAGAAAGCATAAGCCAAATT

TTGAGTAAACTAGTGTGCACACTATCCCATGCCTAGTGGAAGTAGGGATCCTCTCGTTGT

TGGGGGAGTAATTGGGGATGTATTGGATCCTTTTGAATATTCTATTCCTATGAGGGTTAC

CTACAATAACAGAGATGTCAGCAATGGATGTGAATTCAAACCCTCACAAGTTGTCAACCA

ACCAAGGGTAAATATCGGTGGTGATGACC

>H07.05

TATTAGACTCCTAATTTAATATTCTTGTTTTATTATAATGCTGATAAGTCTTGTAAATAA

GGGTGAGAAGCACGAATAATTAGTTCATGAGATGTGTATAATTATTATCCTACACGACTT

ATCTTTGATATTTCACACAAGTCTTTCAATGTATAACAAAAACTTTTTAGATACATTTAG

ACTATAGAACTAACAAGTTATATTTTGAACC-AAAAAACAAAGAGAGAGAG----GAACA

TAGAGAAAAGAAGATATGAGAGTTTTTTTTTT---------CTAAAACAGAAAGAAACTC

ATTATATAATAAACAAATTACTTTGAGACAAATTAACGTATGTAATAAAACAAAATCAAA

GTGGTAATTAAAATTATTTAATGGTAAACAGTCTAATAGTTAAAATAAAAACGGAAATCA

CATAAATTTTGTAATTGGCCCATTAAAAACAACACTAAGCTTTTAATTTGATTTTGAAAT

TCAAAATAATTTTATTAAATCACAAAGTAAAAGGTTTACAAAGCCGATCATGACAGTGCA

TGTGGGAGGCAAATCGGCATATTTGCACTACAAAAGG-ACCCATGAAGCCTCTGAACATG

CACGCAACACTTTAATCTCTTATTAGTTACTTTGAAAGCTTATTTATATATATAGACACG

CGTAAAAACTTCTTAACCAAGATTTTTTT-ACGTGCTTCC-TTCGCGTTTAATTTGGACC

ATCAAACCGTGCTCAACAGATAAAGAAAAGGGTGCTTTTGATTCAAGATATTGGCC-GAA

AAACACAAGATAGATCCTT-CGATAGATTAAGCCACGCATGAAACGCGAATCCAAAGTGA

TGAAGAAGTGCAGATAGATATTCGTTCACCATATAGAGGAGAATATATCATTCCTACAAA

CAAAATTGATGATGTTTCTAGAATAGAAACGTTAGAATCGAATATTCATCTGTTCCGGGT

GGGGAAGGGCTACTGAAAAGCTGAACTTTTTTAAGAGTTCGAACTTCGAAAGTCAATCAA

TGATTCTTAATTGAGAGGGAAAAGCTATAAAGGACGAGAAGGGAAGGAAATGTTTATAGG

GAATGAATTTCTAATGCATGATGGGAATGACAGATATGAGGAATATATAAAGGTGATTCT

TATATACATTGACATGGATCGGAATCTATTAACTTAAAGTTATTGGGGTCGTGAAATTTA

TTTAATTTTTCTAATTCGTGTCACATGTCACGACAATTAGTGGGG-AACATTTATGTATA

TAGAAATTTCAGAAATTTCTAGCAGCGTGATAAATATAAAGATTTTGGCACAATAAGTTT

TTGGATCATAATGAGTTT-CTATTTAATCAA-GGCA-TGTAA-TCTATTTTATTATTTAG

-TAGGG-AAACTGAAAACTTAGGGTTGCTATTTGTAGCTCCCACCCTTCTTAGTTCTTAC

TCTTTTCAATATTTTTTAAAAGTTTTA-TATAATACCT-AAATT-GCCCTCCT---CA--

CCCCCCTGCTAATCT-CT--CTC-TCACACATTGCTTCTCCACCCCATTGCTCTTCCGT-

GCTCCTCCATGTG-AGTTCTAGTCTTTTTTTT--CTCTGGTAGCGTTTTGCTGCTCCTTT

TTTCACTCAAGTGTTGCCAATTAA-TTGACAAAAAGTGGTTTCTGTTTCATGTAGAAACT

ATGTTTTTGTTGTGTAGTCATACATTACGGAATCTAGTTT-CCATTAAATAAGTAACATG

TGAAAAAAAA--TAAAAGGTGAAATACATATTGTTGGAAAAGAAGCTATGAGGTGCAAGA

ACCGATCACATGGAGAAGGAAATGAAAGACAAGGAGGAGCAATGGAAGA-----------

---------GAGGAAATGAGAAGATGGAAGGGATGTGAAAATGTTTGAAAAAGACGAGGT

GGTCAGTTTTGAAATACGAATTTAGTATTTTCTTTTTAAGAAAATTCTTTCG-AAAGTCG

TGTTTTAAAACATGACTTTTATT-ATTTGAAGTCG---TGTTCTAAAACATGACTTA--T

TCATATCCTT-AATATTTTT---------AAAATTTATCCATTTGTAATATTTTTTAAAA

ATTGACCCATATATGTAAAATACCCGTCAAGATCTCTTTATTATTTTGAAAGCGAAAGCA

TATCACTTCAAACACAATGGAATCGAGGCTATTGACTAAGTATAAATAGAGAAGACTTCA

TATCGGGGTTCATAATTCATAACAAAGCAAACGAGTATATAAGAAAGCATAAGCCAAATT

TTGAGTAAACTAGTGTGCACACTATCCCATGCCTAGTGGAAGTAGGGATCCTCTCGTTGT

TGGGGGAGTAATTGGGGATGTATTGGATCCTTTTGAATATTCTATTCCTATGAGGGTTAC

CTACAATAACAGAGATGTCAGCAATGGATGTGAATTCAAACCCTCACAAGTTGTCAACCA

ACCAAGGGTAAATATCGGTGGTGATGACC

>H08.02

TATTAGACTCCTAATTTAATATTCTTGTTTTATTATAATGCTGATAAGTCTTGCAAATAA

GGGTGAGAAGCACGAATAATTAGTTCATGAGATGTGTATAATTATTATCCTACACGACTT

ATCTTTGATATTTCACACAAGTCTTTCAATGTATAACAAAAACTTTTTAGATACATTTAG

ACTATAGAACTAACAAGTTATATTTTGAACC-AAAAAACAAAGAGAGAGAG----GAACA

TAGAGAAAAGAAGATATGAGAGTTTTTTTTTT---------CTAAAACAGAAAGAAACTC

ATTATATAATAAACAAATTACTTTGAGACAAATTAACGTATGTAATAAAACAAAATCAAA

GTGGTAATTAAAATTATTTAATGGTAAACAGTCTAATAGTTAAAATAAAAATGGAAATCA

CATAAATTTTGTAATTGGCCCATTAAAAACAACACTAAGCTTTTAATTTGATTTTGAAAT

TCAAAATAATTTTATTAAATCACAAAGTAAAAGGTTTACAAAGCCGATCATGACAGTGCA

TGTGGGAGGCAAATCGGCATATTTGCACTACAAAAGG-ACCCATGAAGTCTCTGAACATG

CACGCAACACTTTAATCTCTTATTAGTTACTTTGAAAGCTTATTTATATATATAGACACG

CGTAAAAACTTCTTATCCAAGATTTTTTT-ACGTGCTTCC-TTCGCGTTTAATTTGGACC

ATCAAACCGTGCTCAACAGATAAAGAAAAGGGTGCTTTTGATTCAAGATATTGGCCCGAA

AAACACAAGATAGATCCTT-CGATAGATTAAGCCACGCATGAAACGCGAATCCAAAGTGA

TGAAGAAGTGCAGATAGATATTCGTTCACCATATAGAGGAGAATATATCATTCCTACAAA

CAAAATTGATGATGTTTCTAGAATAGAAACGTTAGAACCGAATATTCATCTGTTCCGGGT

GGGGAAGGGCTACTGAAAAGCTGAACTTTTTTAAGAGTTCGAACTTCGAAAGTCAATCAA

TGATTCTTAATTGAGAGGGAAAAGCTATAAAGGACGAGAAGGGAAGGAAATGTTTATAGG

GAATGAATTTCTAATGCATGATGGGAATGACAGATATGAGGAATATATAAAGGTGATTCT

TATATACATTGACATGGATCGGAATCTATTAACTTAAAGTTATTGGGGTCGTGAAATTTA

TTTAATTTTTCTAATTCGTGTCACATGTCACGACAATTAGTGGGG-AACATTTATGTATA

TAGAAATTTCAGAAATTTCTAGCAGCGTGATAAATATAAAGATTTTGGCACAATAAGTTT

TTGGATCATAATGAGTTT-CTATTTAATCAA-GGCA-TGTAA-TCTATTTTATTATTTAG

-TAGGG-AAACTGAAAACTTAGGGTTGCTATTTGTAGCTCCCACCCTTCTTAGTTCTTAC

TCTTTTCAATATTTTTTATAAGTTTTA-TATAATACCT-AAACT-GCCCTCCTCCTCA-C

CCCCCCTGCTAATCTTCTTCCTCCTCACACATTGCTTCT-CACCCCATTGCTCTTCCGTT

GCTCCTCCATGTG-AGTTCTAGTCTTTTTTTT--CTCTGGTAGCGTTTTGCTGCTCCTTT

TTTCACTCAAGTGTTGCCAATTAA-TTGACAAAAAATGGTTTCTGTTTCATATAGAAACT

ATGTTTTTGTTGTGTAGTCATACATTACGGAATCTAGTTT-CCATTAAATAAGTAACATG

TGAAAAAAAA--TAAAAGGTGAAATATATATTGTTGGAAAAGAAGCTATGAGGTGCAAGA

ACCGATCACATGGAGAAGGCAATGAAAGACAAGGAGGAGCAATGGAAGA-----------

---------GAGAAAATGAGAAGATGGAAGGGATGTGAAAATGTTTGAAAAAGACGAGGT

GATCAGTTTTGAAATACGAATTTAGTATTTTCTTTTTAAGAAAATTCTTTCG-AAAGTCG

TGTTTTAAAACATGACTTTTATT-ATTTGAAGTCTGTTCGATCTAAAACATGACTTA--T

TCATATCCTT-AATATTTTT---------AAAATTTATCCATTTGTAATATTTTTTAAAA

ATTGACCCATATATGTAAAATACCCGTCGAGATCTCTTTATTATTTTGAAAGCGAAAGCA

TATCACTCCAAACACAATGGAATCGAGGCTATTGACTAAGTATAAATAGAGAAGACTTCA

TATCGGGGTTCATAATTCATAACAAAGCAAACGAGTATATAAGAAAGCATAAGCCAAATT

TTGAGTAAACTAGTGTGCACACTATCCCATGCCTAGTGGAAGTAGGGATCCTCTCGTTGT

TGGGGGAGTAATTGGGGATGTATTGGATCCTTTTGAATATTCTATTCCTATGAGGGTTAC

CTTCAATAACAGAGATGTCAGCAATGGATGTGAATTCAAACCCTCACAAGTTGTCAACCA

ACCAAGGGTAAATATCGGTGGTGATGACC

>H08.03

TATTAGACTCCTAATTTAATATTCTTGTTTTATTATAATGCTGATAAGTCTTGTAAATAA

GGGTGAGAAGCACGAATAATTAGTTCATGAGATGTGTATAATTATTATCCTACACGACTT

ATCTTTGATATTTCACACAAGTCTTTCAATGTATAACAAAAACTTTTTAGATACATTTAG

ACTATAGAACTAACAAGTTATATTTTGAACC-AAAAAACAAAGAGAGAGAG----GAACA

TAGAGAAAAGAAGATATGAGAGTTTTTTTTTT---------CTAAAACAGAAAGAAACTC

ATTATATAATAAACAAATTACTTTGAGACAAATTAACGTATGTAATAAAACAAAATCAAA

GTGGTAATTAAAATTATTTAATGGTAAACAGTCTAATAGTTAAAATAAAAATGGAAATCA

CATAAATTTTGTAATTGGCCCATTAAAAACAACACTAAGCTTTTAATTTGATTTTGAAAT

TCAAAATAATTTTATTAAATCACAAAGTAAAAGGTTTACAAAGCCGATCATGACAGTGCA

TGTGGGAGGCAAATCGGCATATTTGCACTACAAAAGG-ACCCATGAAGTCTCTGAACATG

CACGCAACACTTTAATCTCTTATTAGTTACTTTGAAAGCTTATTTATATATATAGACACG

CGTAAAAACTTCTTATCCAAGATTTTTTT-ACGTGCTTCC-TTCGCGTTTAATTTGGACC

ATCAAACCGTGCTCAACAGATAAAGAAAAGGGTGCTTTTGATTCAAGATATTGGCC-GAA

AAACACAAGATAGATCCTT-CGATAGATTAAGCCACGCATGAAACGCGAATCCAAAGTGA

TGAAGAAGTGCAGATAGATATTCGTTCACCATATAGAGGAGAATATATCATTCCTACAAA

CAAAATTGATGATGTTTCTAGAATAGAAACGTTAGAATCGAATATTCATCTGTTCCGGGT

GGGGAAGGGCTACTGAAAAGCTGAACTTTTTTAAGAGTTCGAACTTCGAAAGTCAATCAA

TGATTCTTAATTGAGAGGGAAAAGCTATAAAGGACGAGAAGGGAAGGAAATGTTTATAGG

GAATGAATTTCTAATGCATGATGGGAATGACAGATATGAGGAATATATAAAGGTGATTCT

TATATACATTGACATGGATCGGAATCTATTAACTTAAAGTTATTGGGGTCGTGAAATTTA

TTTAATTTTTCTAATTCGTGTCACATGTCACGACAATTAGTGGGG-AACATTTATGTATA

TAGAAATTTCAGAAATTTCTAGCAGCGTGATAAATATAAAGATTTTGGCACAATAAGTTT

TTGGATCATAATGAGTTT-CTATTTAATCAA-GGCA-TGTAA-TCTATTTTATTATTTAG

-TAGGG-AAACTGAAAACTTAGGGTTGCTATTTGTAGCTCCCACCCTTCTTAGTTCTTAC

TCTTTTCAATATTTTTTATAAGTTTTA-TATAATACCT-AAATT-GCCCTCCTCCTCA-C

CCCCCCTGCTAATCTTCTTCCTCCTCACACATTGCTTCT-CACCCCATTGCTCTTCCGTT

GCTCCTCCATGTG-AGTTCTAGTCTTTTTTTT--CTCTGGTAGCGTTTTGCTGCTCCTTT

TTTCACTCAAGTGTTGCCAATTAA-TTGACAAAAAATGGTTTCTGTTTCGTATAGAAACT

ATGTTTTTGTTGTGTAGTCATACATTACGGAATCTAGTTT-CCATTAAATAAGTAACATG

TGAAAAAAAA--TAAAAGGTGAAATATATATTGTTGGAAAAGAAGCTATGAGGTGCAAGA

ACCGATCACATGGAGAAGGCAATGAAAGACAAGGAGGAGCAATGGAAGA-----------

---------GAGAAAATGAGAAGATGGAAGGGATGTGAAAATGTTTGAAAAAGACGAGGT

GATCAGTTTTGAAATACGAATTTAGTATTTTCTTTTTAAGAAAATTCTTTCG-AAAGTCG

TGTTTTAAAACATGACTTTTATT-ATTTGAAGTCTGTTCGATCTAAAACATGACTTA--T

TCATATCCTT-AATATTTTT---------AAAATTTATCCATTTGTAATATTTTTTAAAA

ATTGACCCATATATGTAAAATACCCGTCAAGATCTCTTTATTATTTTGAAAGCGAAAGCA

TATCACTTCAAACACAATGGAATCGAGGCTATTGACTAAGTATAAATAGAGAAGACTTCA

TATCGGGGTTCATAATTCATAACAAAGCAAACGAGTATATAAGAAAGCATAAGCCAAATT

TTGAGTAAACTAGTGTGCACACTATCCCATGCCTAGTGGAAGTAGGGATCCTCTCGTTGT

TGGGGGAGTAATTGGGGATGTATTGGATCCTTTTGAATATTCTATTCCTATGAGGGTTAC

CTACAATAACAGAGATGTCAGCAATGGATGTGAATTCAAACCCTCACAAGTTGTCAACCA

ACCAAGGGTAAATATCGGTGGTGATGACC

>H10.A8

TATTAGACTCCTAATTTAATATTCTTGTTTTATTATAATGCTGATAAGTCTTGTAAATAA

GGGTGAGAAGCACGAATAATTAGTTCATGAGATGTGTATAATTATTATCCTACACGACTT

ATCTTTGATATTTCACACAAGTCTTTCAATGTATAACAAAAACTTTTTGGATACATTTAG

ACTATAGAACTAACAAGTTATATTTTGAACC-AAAAAACAAAGAGAGAGAG----GAACA

TAGAGAAAAGAAGATATGAGAGTTTTTTTTTT---------CTAAAACAGAAAGAAACTC

ATTATATAATAAACAAATTACTTTGAGACAAATTAACGTATGTAATAAAACAAAATCAAA

GTGGTAATTAAAATTATTTAATGGTAAACAGTCTAATAGTTAAAATAAAAATGGAAATCA

CATAAATTTTGTAATTGGCCCATTAAAAACAACACTAAGCTTTTAATTTGATTTTGAAAT

TCAAAATAATTTTATTAAATCACAAAGTAAAAGGTTTACAAAGCCGATCATGACAGTGCA

TGTGGGAGGCAAATCGGCATATTTGCACTACAAAAGG-ACCCATGAAGTCTCTGAACATG

CACGCAACACTTTAATCTCTTATTAGTTACTTTGAAAGCTTATTTATATATATAGACACG

CGTAAAAACTTCTTAACCAAGATTTTTTT-ACGTGCTTCC-TTCGCGTTTAATTTGGACC

ATCAAACCGTGCTCAACAGATAAAGAAAAGGGTGCTTTTGATTCAAGATATTGGCC-GAA

AAACACAAGGTAGATCCTT-CGATAGATTAAGCCACGCATGAAACGCGAATCCAAAGTGA

TGAAGAAGTGCAGATAGATATTCGTTCACCATATAGAGGAGAATATATCATTCCTACAAA

CAAAATTGATGATGTTTCTAGAATAGAAACGTTAGAATCGAATATTCATCTGTTCCGGGT

GGGGAAGGGCTACTGAAAAGCTGAACTTTTTTAAGAGTTCGAACTTCGAAAGTCAATCAA

TGATTCTTAATTGAGAGGGAAAAGCTATAAAGGACGAGAAGGGAAGGAAATGTTTATAGG

GAATGAATTTCTAATGCATGATGGGAATGACAGATATGAGGAATATATAAAGGTGATTCT

TATATACATCGACATGGATCGGAATCTATTAACTTAAAGTTATTGGGGTCGTGAAATTTA

TTTAATTTTTCTAATTCGTGTCACATGTCACGACAATTAGTGGGG-AACATTTATGTATA

TAGAAATTTCAGAAATTTCTAGCAGCGTGATAAATATAAAGATTTTGGCACAATAAGTTT

TTGGATCATAATGAGTTT-CTATTTAATCAA-GGCA-TGTAA-TCTATTTTATTATTTAG

-TAGGG-AAACTGAAAACTTAGGGTTGCTATTTGTAGCTCCCACCCTTCTTAGTTCTTAC

TCTTTTCAATATTTTTTATAAGTTTTA-TATAATACCT-AAATT-GCCCTCCTCCTCACC

CCCCCCTGCTAATCTTCTTCCTCCTCACACATTGCTTCT-CACCCCATTGCTCTTCCGTT

GCTCCTCCATGTG-AGTTCTAGTCTTTTTTTTT-CTCTGGTAGCGTTTTGCTGCTCCTTT

TTTCACTCAAGTGTTGCCAATTAA-TTGACAAAAAATGGTTTCTGTTTCATATAGAAACT

ATGTTTTTGTTGTGTAGTCATACATTACGGAATCTAGTTT-CCATTAAATAAGTAACATG

TGAAAAAAAA--TAAAAGGTGAAATATATATTGTTGGAAAAGAAGCTATGAGGTGCAAGA

ACCGATCACATGGAGAAGGCAATGAAAGACAAGGAGGAGCAATGGAAGA-----------

---------GAGAAAATGAGAAGATGGAAGGGATGTGAAAATGTTTGAAAAAGACGAGGT

GATCAGTTTTGAAATACGAATTTAGTATTTTCTTTTTAAGAAA-TTCTTTCG-AAAGTCG

TGTTTTAAAACATGACTTTTATT-ATTTGAAGTC----TGTTCTAAAACATGACTTA--T

TCATATCCTT-AATATTTTT---------AAAATTTATCCATTTGTAATATTTTTTAAAA

ATTGACCCATATATGTAAAATACCCGTCAAGATCTCTTTATTATTTTGAAAGCGAAAGCA

TATCACTTCAAACACAATGGAATCGAGGCTATTGACTAAGTATAAATAGAGAAGACTTCA

TATCGGGGTTCATAATTCATAACAAAGCAAACGAGTATATAAGAAAGCATAAGCCAAATT

TTGAGTAAACTAGTGTGCACACTATCCCATGCCTAGTGGAAGTAGGGATCCTCTCGTTGT

TGGGGGAGTAATTGGGGATGTATTGGATCCTTTTGAATATTCTATTCCTATGAGGGTTAC

CTACAATAACAGAGATGTCAGCAATGGATGTGAATTCAAACCCTCACAAGTTGTCAACCA

ACCAAGGGTAAATATCGGTGGTGATGACC

>H10.A1

TATTAGACTCCTAATTTAATATTCTTGTTTTATTATAATGCTGATAAGTCTTGTAAATAA

GGGTGAGAAGCACGAATAATTAGTTCATGAGATGTGTATAATTATTATCCTACACGACTT

ATCTTTGATATTTCACACAAGTCTTTCAATGTATAACAAAAACTTTTTAGATACATTTAG

ACTATAGAACTAACAAGTTATATTTTGAACC-AAAAAACAAAGAGAGAGAG----GAACA

TAGAGAAAAGAAGATATGAGAGTTTTTTTTTT---------CTAAAACAGAAAGAAACTC

ATTATATAATAAACAAATTACTTTGAGACAAATTAACGTATGTAATAAAACAAAATCAAA

GTGGTAATTAAAATTATTTAATGGTAAACAGTCTAATAGTTAAAATAAAAATGGAAATCA

CATAAATTTTGTAATTGGCCCATTAAAAACAACACTAAGCTTTTAATTTGATTTTGAAAT

TCAAAATAATTTTATTAAATCACAAAGTAAAAGGTTTACAAAGCCGATCATGACAGTGCA

TGTGGGAGGCAAATCGGCATATTTGCACTACAAAAGG-ACCCATGAAGTCTCTGAACATG

CACGCAACACTTTAATCTCTTATTAGTTACTTTGAAAGCTTATTTATATATATAGACACG

CGTAAAAACTTCTTAACCAAGATTTTTTT-ACGTGCTTCC-TTCGCGTTTAATTTGGACC

ATCAAACCGTGCTCAACAGATAAAGAAAAGGGTGCTTTTGATTCAAGATATTGGCCCGAA

AAACACAAGATAGATCCTT-CGATAGATTAAGCCACGCATGAAACGCGAATCCAAAGTGA

TGAAGAAGTGCAGATAGATATTCGTTCACCATATAGAGGAGAATATATCATTCCTACAAA

CAAAATTGATGATGTTTCTAGAATAGAAACGTTAGAATCGAATATTCATCTGTTCCGGGT

GGGGAAGGGCTACTGAAAAGCTGAACTTTTTTAAGAGTTCGAACTTCGAAAGTCAATCAA

TGATTCTTAATTGGGAGGGAAAAGCTATAAAGGACGAGAAGGGAAGGAAATGTTTATAGG

GAATGAATTTCTAATGCATGATGGGAATGACAGATATGAGGAATATATAAAGGTGATTCT

TATATACATCGGCATGGATCGGAATCTATTAACTTAAAGTTATTGGGGTCGTGAAATTTA

TTTAATTTTTCTAATTCGTGTCACATGTCACGACAATTAGTGGGG-AACATTTATGTATA

TAGAAATTTCAGAAATTTCTAGCAGCGTGATAAATATAAAGATTTTGGCACAATAAGTTT

TTGGATCATAATGAGTTT-CTATTTAATCAA-GGCA-TGTAA-TCTATTTTATTATTTAG

-TAGGG-AAACTGAAAACTTAGGGTTGCTATTTGTAGCTCCCACCCTTCTTAGTTCTTAC

TCTTTTCAATATTTTTTATAAGTTTTA-TATAATACCT-AAATT-GCCCTCCTCCTCACC

CCCCCCTGCTAATCTTCTTCCTCCTCACACATTGCTTCT-CACCCCATTGCTCTTCCGTT

GCTCCTCCATGTG-AGTTCTAGTCTTTTTTTTT-CTCTGGTAGCGTTTTGCTGCTCCTTT

TTTCACTCAAGTGTTGCCAATTAA-TTGACAAAAAATGGTTTCTGTTTCATATAGAAACT

ATGTTTTTGTTGTGTAGTCATACATTACGGAATCTAGTTT-CCATTAAATAAGTAACATG

TGAAAAAAAA--TAAAAGGTGAAATATATATTGTTGGAAAAGAAGCTATGAGGTGCAAGA

ACCGATCACATGGAGAAGGCAATGAAAGACAAGGAGGAGCAATGGAAGA-----------

---------GAGAAAATGAGAAGATGGAAGGGATGTGAAAATGTTTGAAAAAGACGAGGT

GATCAGTTTTGAAATACGAATTTAGTATTTTCTTTTTAAGAAA-TTCTTTCG-AAAGTCG

TGTTTTAAAACATGACTTTTATT-ATTTGAAGTC----TGTTCTAAAACATGACTTA--T

TCATATCCTT-AATATTTTT---------AAAATTTATCCATTTGTAATATTTTTTAAAA

ATTGACCCATATATGTAAAATACCCGTCAAGATCTCTTTATTATTTTGAAAGCGAAAGCA

TATCACTTCAAACACAATGGAATCGAGGCTATTGACTAAGTATAAATAGAGAAGACTTCA

TATCGGGGTTCATAATTCATAACAAAGCAAACGAGTATATAAGAAAGCATAAGCCAAATT

TTGAGTAAACTAGTGTGCACACTATCCCATGCCTAGTGGAAGTAGGGATCCTCTCGTTGT

TGGGGGAGTAATTGGGGATGTATTGGATCCTTTTGAATATTCTATTCCTATGAGGGTTAC

CTACAATAACAGAGATGTCAGCAATGGATGTGAATTCAAACCCTCACAAGTTGTTAACCA

ACCAAGGGTAAATATCGGTGGTGATGACC

>CS60.L7

TATTAGACTCCTAATTTGATATTCTTGTTTTATTATAATGCTGATAAGTCTTGTAAATAA

GGGTGAGAAGCACGAATAATTAGTTCATGAGATGTGTATAATTATTATCCTACACGACTT

ATCTTTGATATTTCACACAAGTCTTTCAATGTATAACAAAAATTTTTTAGATACATTTAG

ACTATAGAACTAACAAGCTATATTTTGAACC-AAAAAACAAAGAGAGAGAG----GAACA

TAGAGAAAAGAAGATATGAGAGTTTTTTTTTTT--------CTAAAACAGAAAGAAACTC

ATTATATAATAAACAAATTACTTTGAGACAAATTAACGTATGTAATAAAACAAAATCAAA

GTGGTAATTAAAATTATTTAATGGTAAACAGTCTAATAGTTAAAATAAAAATGGAAATCA

CATAAATTTTGTAATTGGCCCATTAAAAACAACACAAAGCTTTTAATTTGATTTTGAAAT

TCAAAATAATTTTATTAAATCACAAAGTAAAAGGTTTACAAAGCCGATCATGACAGTGCA

TGTGGGAGGCAAATCGGCATATTTGCACTACAAAAGG-ACCCATGAAGTCTCTGAACATG

CACGCAACACTTTAATCTCTTATTAGTTACTTTGAAAGCTTATTTATATATATAGACACG

CGTAAAAACTTCTTAACCAAGATTTTTTT-ACGTGCTTCC-TTCGCGTTTAATTTGGACC

ATCAAACCGTGCTCAACAGATAAAGAAAAGGGTGCTTTTGATTCAAGATATTGGCC-GAA

AAACACAAGATAGATCCTT-CGATAGATTAAGCCACGCATGAAACGCGAATCCAAAGTGA

TGAAGAAGTGCAGATAGATATTCGTTCACCATATAGAGGAGAATATATCATTCCTACAAA

CAAAATTGATGATGTTTCTAGAATAGAAACGTTAGAATCGAATATTCATCTGTTCCGGGT

GGGGAAGGGCTACTGAAAAGCTGAACTTTTTTAAGAGTTCGAACTTCGAAAGTCAATCAA

TGATTCTTAATTGAGAGGGAAAAGCTATAAAGGACGAGAAGGGAAGGAAATGTTTATAGG

GAATGAATTTCTAATGCATGATGGGAATGACAGATATGAGGAATATATAAAGGTGATTCT

TATATACATTGACATGGATCGGAATCTATTAACTTAAAGTTATTGGGGTCGTGAAATTTA

TTTAATTTTTCTAATTCGTGTCACATGTCACGACAATTAGTGGGG-AACATTTATGTATA

TAGAAATTTCAGAAATTTCTAGCAGCGTGATAAATATAAAGATTTTGGCACAATAAGTTT

TTGGATCATAATGAGTTT-CTATTTAATCAA-GGCA-TGTAA-TCTATTTTATTATTTAG

-TAGGG-AAACTGAAAACTTAGGGTTGCTATTTGTAGCTCCCACCCTTCTTAGTTCTTAC

TCTTTTCAATATTTTTTATAAGTTTTA-TATAATACCT-AAATT-GCCCTCCTCCTCA-C

CCCCCCTGCTAATCTTCTTCCTCCTCACACATTGCTTCT-CACCCCATTGCTCTTCCGTT

GCTCCTCCATGGGGAGTTCTAGTCTTTTTTTTT-CTCTGGTAGCGTTTTGCTGCTCCTTT

TTTCACTCAAGTGTTGCCAATTAA-TTGGCAAAAAATGGTTTCTGTTTCATATAGAAACT

ATGTTTTTGTTGTGTAGTCATACATTACGGAATCTAGTTT-CCATTAAATAAGTAACATG

TGAAAAAAAA--TAAAAGGTGAAATATATATTGTTGGAAAAGAAGCTATGAGGTGCAAGA

ACCGATCACATGGAGAAGGCAATGAAAGACAAGGAGGAGCAATGAAAGATAAGGAGGAGC

AATGAAAGAGAGAAAATGAGAAGATGGAAGGGACGTGAAAATGTTTGAAAAAGACGAGGT

GATCAGTTTTGAAATACGAATTTAGTATTTTCTTTTTAAGAAAATTCTTTCG-AAAGTCG

TGTTTTAAAACATGACTTTTATT-ATTTGAAGTCG---TGTTCTAAAACATGGCTTA--T

TCATATCCTT-AATATTTTT---------AAAATTTATCCATTTGTAATATTTTTTAAAA

ATTGACCCATATATGTAAAATACCCGTCAAGATCTCTTTATTATTTTGAAAGCGAAAGCA

TATCACTTCAAACACAATGGAATCGAGGCTATTGACTAAGTATAAATAGAGAAGACTTCA

TATCGGGGTTCATAATTCATAACAAAGCAAACGAGTATATAAGAAAGCATAAGCCAAATT

TTGAGTAAACTAGTGTGCACACTATCCCATGCCTAGTGGAAGTAGGGATCCTCTCGTTGT

TGGGGGAGTAATTGGGGATGTATTGGATCCTTTTGAATATTCTATTCCTATGAGGGTTAC

CTACAATAACAGAGATGTCAGCAATGGATGTGAATTCAAACCCTCACAAGTTGTCAACCA

ACCAAGGGTAAATATCGGTGGTGATGACC

>ZG.ALL

TATTAGACTCCTAATTTGATATTCTTGTTTTATTATAATGCTGATAAGTCTTGTAAATAA

GGGTGAGAAGCACGAATAATTAGTTCATGAGATGTGTATAATTATTATCCTACACGACTT

ATCTTTGATATTTCACACAAGTCTTTCAATGTATAACAAAAATTTTTTAGATACATTTAG

ACTATAGAACTAACAAGTTATATTTTGAACC-AAAAAACAAAGAGAGAGAG----GAACA

TAGAGAAAAGAAGATATGAGAGTTTTTTTTTTT--------CTAAAACAGAAAGAAACTC

ATTATATAATAAACAAATTACTTTGAGACAAATTAACGTATGTAATAAAACAAAATCAAA

GTGGTAATTAAAATTATTTAATGGTAAACAGTCTAATAGTTAAAATAAAAATGGAAATCA

CATAAATTTTGTAATTGGCCCATTAAAAACAACACAAAGCTTTTAATTTGATTTTGAAAT

TCAAAATAATTTTATTAAATCACAAAGTAAAAGGTTTACAAAGCCGATCATGACAGTGCA

TGTGGGAGGCAAATCGGCATATTTGCACTACAAAAGG-ACCCATGAAGTCTCTGAACATG

CACGCAACACTTTAATCTCTTATTAGTTACTTTGAAAGCTTATTTATATATATAGACACG

CGTAAAAACTTCTTAACCAAGATTTTTTT-ACGTGCTTCC-TTCGCGTTTAATTTGGACC

ATCAAACCGTGCTCAACAGATAAAGAAAAGGGTGCTTTTGATTCAAGATATTGGCC-GAA

AAACACAAGATAGATCCTT-CGATAGATTAAGCCACGCATGAAACGCGAATCCAAAGTGA

TGAAGAAGTGCAGATAGATATTCGTTCACCATATAGAGGAGAATATATCATTCCTACAAA

CAAAATTGATGATGTTTCTAGAATAGAAACGTTAGAATCGAATATTCATCTGTTCCGGGT

GGGGAAGGGCTACTGAAAAGCTGAACTTTTTTAAGAGTTCGAACTTCGAAAGTCAATCAA

TGATTCTTAATTGAGAGGGAAAAGCTATAAAGGACGAGAAGGGAAGGAAATGTTTATAGG

GAATGAATTTCTAATGCATGATGGGAATGACAGATATGAGGAATATATAAAGGTGATTCT

TATATACATTGACATGGATCGGAATCTATTAACTTAAAGTTATTGGGGTCGTGAAATTTA

TTTAATTTTTCTAATTCGTGTCACATGTCACGACAATTAGTGGGG-AACATTTATGTATA

TAGAAATTTCAGAAATTTCTAGCAGCGTGATAAATATAAAGATTTTGGCACAATAAGTTT

TTGGATCATAATGAGTTT-CTATTTAATCAA-GGCA-TGTAA-TCTATTTTATTATTTAG

-TAGGG-AAACTGAAAACTTAGGGTTGCTATTTGTAGCTCCCACCCTTCTTAGTTCTTAC

TCTTTTCAATATTTTTTATAAGTTTTA-TATAATACCT-AAATT-GCCCTCCTCCTCA-C

CCCCCCTGCTAATCTTCTTCCTCCTCACACATTGCTTCT-CACCCCATTGCTCTTCCGTT

GCTCCTCCATGTG-AGTTCTAGTCTTTTTTTT--CTCTGGTAGCGTTTTGCTGCTCCTTT

TTTCACTCAAGTGTTGCCAATTAA-TTGACAAAAAATGGTTTCTGTTTCATATAGAAACT

ATGTTTTTGTTGTGTAGTCATACATTACGGAATCTAGTTT-CCATTAAATAAGTAACATG

TGAAAAAAAA--TAAAAGGTGAAATATATATTGTTGGAAAAGAAGCTATGAGGTGCAAGA

ACCGATCACATGGAGAAGGCAATGAAAGACAAGGAGGAGCAATGAAAGATAAGGAGGAGC

AATGAAAGAGAGAAAATGAGAAGATGGAAGGGATGTGAAAATGTTTGAAAAAGACGAGGT

GATCAGTTTTGAAATACGAATTTAGTATTTTCTTTTTAAGAAAATTCTTTCG-AAAGTCG

TGTTTTAAAACATGACTTTTATT-ATTTGAAGTCG---TGTTCTAAAACATGGCTTA--T

TCATATCCTT-AATATTTTT---------AAAATTTATCCATTTGTAATATTTTTTAAAA

ATTGACCCATATATGTAAAATACCCGTCAAGATCTCTTTATTATTTTGAAAGCGAAAGCA

TATCACTTCAAACACAATGGAATCGAGGCTATTGACTAAGTATAAATAGAGAAGACTTCA

TATCGGGGTTCATAATTCATAACAAAGCAAACGAGTATATAAGAAAGCATAAGCCAAATT

TTGAGTAAACTAGTGTGCACACTATCCCATGCCTAGTGGAAGTAGGGATCCTCTCGTTGT

TGGGGGAGTAATTGGGGATGTATTGGATCCTTTTGAATATTCTATTCCTATGAGGGTTAC

CTACAATAACAGAGATGTCAGCAATGGATGTGAATTCAAACCCTCACAAGTTGTCAACCA

ACCAAGGGTAAATATCGGTGGTGATGACC

>CS60.L1

TATTAGACTCCTAATTTGATATTCTTGTTTTATTATAATGCTGATAAGTCTTGTAAATAA

GGGTGAGGAGCACGAATAATTAGTTCATGAGATGTGTATAATTATTATCCTACACGACTT

ATCTTTGATATTTCACACAAGTCTTTCAATGTATAACAAAAATTTTTTAGATACATTTAG

ACTATAGAACTAACAAGTTATATTTTGAACC-AAAAAACAAAGAGAGAGAG----GAACA

TAGAGAAAAGAAGATATGAGAGTTTTTTTTTTT--------CTAAAACAGAAAGAAACTC

ATTATATAATAAACAAATTACTTTGAGACAAATTAACGTATGTAATAAAACAAAATCAAA

GTGGTAATTAAAATTATTTAATGGTAAACAGTCAAATAGTTAAAATAAAAATGGAAATCA

CATAAATTTTGTAATTGGCCCATTAAAAACAACACAAAGCTTTTAATTTGATTTTGAAAT

TCAAAATAATTTTATTAAATCACAAAGTAAAAGGTTTACAAAGCCGATCATGACAGTGCA

TGTGGGAGGCAAATCGGCATATTTGCACTACAAAAGG-ACCCATGAAGTCTCTGAACATG

CACGCGACACTTTAATCTCTTATTAGTTACTTTGAAAGCTTACTTATATATATAGACACG

CGTAAAAACTTCTTAACCAAGATTTTTTT-ACGTGCTTCC-TTCGCGTTTAATTTGGACC

ATCAAACCGTGCTCAACAGATAAAGAAAAGGGTGCTTTTGATTCAAGATATTGGCC-GAA

AAACACAAGATAGATCCTT-CGATAGATTAAGCCACGCATGAAACGCGAATCCAAAGTGA

TGAAGAAGTGCAGATAGATATTCGTTCACCATATAGAGGAGAATATATCATTCCTACAAA

CAAAATTGATGATGTTTCTAGAATAGAAACGTTAGAATCGAATATTCATCTGTTCCGGGT

GGGGAAGGGCTACTGAAAAGCTGAACTTTTTTAAGAGTTCGAACTTCGAAAGTCAATCAA

TGATTCTTAATTGAGAGGGAAAAGCTATAAAGGACGAGAAGGGAAGGAAGTGTTTATAGG

GAATGAATTTCTAATGCATGATGGGAATGACAGATATGAGGAATATATAAAGGTGATTCT

TATATACATTGACATGGATCGGAATCTATTAACTTAAAGTTATTGGGGTCGTGAAATTTA

TTTAATTTCTCTAATTCGTGTCACATGTCACGACAATTAGTGGGG-AACATTTATGTATA

TAGAAATTTCAGAAATTTCTGGCAGCGTGATAAATATAAAGATTTTGGCACAATAAGTTT

TTGGATCATAATGAGTTT-CTATTTAATCAA-GGCA-TGTAA-TCTATTTTATTATTTAG

-TAGGG-AAACTGAAAACTTAGGGTTGCTATTTGTAGCTCCCACCCTTCTTAGTTCTTAC

TCTTTTCAATATTTTTTATAAGTTTTA-TATAATACCT-AAATT-GCCCTCCTCCTCA-C

CCCCCCTGCTGATCTTCTTCCTCCTCACACATTGCTTCT-CACCC-ATTGCTCTTCCGTT

GCTCCTCCATGTG-AGTTCTAGTCTTTTTTTTT-CTCTGGTAGCGTTTTGCTGCTCCTTT

TTTCACTCAAGTGTTGCCAATTAA-TTGACAAAAAATGGTTTCTGTTTCATATAGAAACT

ATGTTTTTGTTGTGTAGTCATACATTACGGAATCTAGTTT-CCATTAAATAAGTAACATG

TGAAAAAAAA--TAAAAGGTGAGATATATATTGTTGGAAAAGAAGCTATGAGGTGCAAGA

ACCGATCACATGGAGAAGGCAATGAAAGACAAGGAGGAGCAATGAAAGATAAGGAGGAGC

AATGAAAGAGAGAAGATGAGAAGATGGAAGGGATGTGAAAATGTTTGAAAAAGACGAGGT

GATCAGTTTTGAAATACGAATTTAGTATTTTCTTTTTAAGAAAATTCTTTCG-AAAGTCG

TGTTTTAAAACATGACTTTTATT-ATTTGAAGTCG---TGTTCTAAAACATGGCTTA--T

TCATATCCTT-AATATTTTT---------AAAATTTATCCATTTGTAATATTTTTTAAAA

ATTGACCCATATATGTAAAATACCCGTCAAGATCTCTTTATTATTTTGAAAGCGAAAGCA

TATCACTTCAAACACAATGGAATCGAGGCTATTGACTAAGTATAAATAGAGAAGACTTCA

TATCGGGGTTCATAATTCATAACAAAGCAAGCGAGTATATAAGAAAGCATAAGCCAAATT

TTGAGTAAACTAGTGTGCACACTATCCCATGCCTAGTGGAAGTAGGGATCCTCTCGTTGT

TGGGGGAGTAATTGGGGATGTATTGGATCCTTTTGAATATTCTATTCCTATGAGGGTTAC

CTACAATAACAGAGATGTCAGCAATGGATGCGAATTCAAACCCTCACAAGTTGTCAACCA

ACCAAGGGTAAATATCGGTGGTGATGACC

>JU.63

TATTAGACTCCTAATTTGATATTCTTGTTTTATTATAATGCTGATAAGTCTTGTAAATAA

GGGTGAGAAGCACGAATAATTAGTTCATGAGATGTGTATAATTATTATCCTACACGACTT

ATCTTTGATATTTCACACAAGTCTTTCAATGTATAACAAAAATTTTTTAGATACATTTAG

ACTATAGAACTAACAAGTTATATTTTGAACC-AAAAAACAAAGAGAGAGAG----GAACA

TAGAGAAAAGAAGATATGAGAGTTTTTTTTTT---------CTAAAACAGAAAGAAACTC

ATTATATAATAAACAAATTACTTTGAGACAAATTAACGTATGTAATAAAACAAAATCAAA

GTGGTAATTAAAATTATTTAATGGTAAACAGTCTAATAGTTAAAATAAAAATGGAAATCA

CATAAATTTTGTAATTGGCCCATTAAAAACAACACAAAGCTTTTAATTTGATTTTGAAAT

TCAAAATAATTTTATTAAATCACAAAGTAAAAGGTTTACAAAGCCGATCATGACAGTGCA

TGTGGGAGGCAAATCGGCATATTTGCACTACAAAAGG-ACCCATGAAGTCTCTGAACATG

CACGCAACACTTTAATCTCTTATTAGTTACTTTGAAAGCTTATTTATATATATAGACACG

CGTAAAAACTTCTTAACCAAGATTTTTTT-ACGTGCTTCC-TTCGCGTTTAATTTGGACC

ATCAAACCGTGCTCAACAGATAAAGAAAAGGGTGCTTTTGATTCAAGATATTGGCC-GAA

AAACACAAGATAGATCCTT-CGATAGATTAAGCCACGCATGAAACGCGAATCCAAAGTGA

TGAAGAAGTGCAGATAGATATTCGTTCACCATATAGAGGAGAATATATCATTCCTACAAA

CAAAATTGATGATGTTTCTAGAATAGAAACGTTAGAATCGAATATTCATCTGTTCCGGGT

GGGGAAGGGCTACTGAAAAGCTGAACTTTTTTAAGAGTTCGAACTTCGAAAGTCAATCAA

TGATTCTTAATTGAGAGGGAAAAGCTATAAAGGACGAGAAGGGAAGGAAGTGTTTATAGG

GAATGAATTTCTAATGCATGATGGGAATGACAGATATGAGGAATATATAAAGGTGATTCT

TATATACATTGACATGGATCGGAATCTATTAACTTAAAGTTATTGGGGTCGTGAAATTTA

TTTAATTTTTCTAATTCGTGTCACATGTCACGACAATTAGTGGGG-AACATTTATGTATA

TAGAAATTTCAGAAATTTCTAGCAGCGTGATAAATATAAAGATTTTGGCACAATAAGTTT

TTGGATCATAATGAGTTT-CTATTTAATCAA-GGCA-TGTAA-TCTATTTTATTATTTAG

-TAGGG-AAACTGAAAACTTAGGGTTGCTATTCGTAGCTCCCACCCTTCTTAGTTCTTAC

TCTTTTCAATATTTTTTATAAGTTTTA-TATAATACCT-AAATT-GCCCTCCTCCTCA-C

CCCCCCTGCTAATCTTCTTCCTCCTCACACATTGCTTCT-CACCCCATTGCTCTTCCGTT

GCTCCTC-ATGTG-AGTTCTAGTCTTTTTTT---CTCTGGTAGCGTTTTGCTGCTCCTTT

TTTCACTCAAGTGTTGCCAATTAA-TTGACAAAAAATGGTTTCTGTTTCATATAGAAACT

ATGTTTTTGTTGTGTAGTCATACATTACGGAATCTAGTTT-CCATTAAATAAGTAACATG

TGAAAAAAAA--TAAAAGGTGAAATATATATTGTTGGAAAAGAAGCTATGAGGTGCAAGA

ACCGATCACATGGAGAAGGCAATGAAAGACAAGGAGGAGCAATGAAAGATAAGGAGGAGC

AATGAAAGAGAGAAAATGAGAAGATGGAAGGGATGTGAAAATGTTTGAAAAAGACGAGGT

GATCAGTTTTGAAATACGAATTTAGTATTTTCTTTTTAAGAAAATTCTTTCG-AAAGTCG

TGTTTTAAAACATGACTTTTATT-ATTTGAAGTCG---TGTTCTAAAGCATGGCTTA--T

TCATATCCTT-AATATTTTT---------AAAATTTATCCATTTGTAATATTTTTTAAAA

ATTGACCCATATATGTAAAATACCCGTCAAGATCTCTTTATTATTTTGAAAGCGAAAGCA

TATCACTTCAAACACAATGGGATCGAGGCTATTGACTAAGTATAAATAGAGAAGACTTCA

TATCGGGGTTCATAATTCATAACAAAGCAAACGAGTATATAAGAAAGCATAAGCCAAATT

TTGAGTAAACTAGTGTGCACACTATCCCATGCCTAGTGGAAGTAGGGATCCTCTCGTTGT

TGGGGGAGTAATTGGGGATGTATTGGATCCTTTCGAATATTCTATTCCTATGAGGGTTAC

CTACAATAACAGAGATGTCAGCAATGGATGTGAATTCAAACCCTCACAAGTTGTCAACCA

ACCAAGGGTAAATATCGGTGGTGATGACC

>JU.68

TATTAGACTCCTAATTTGATATTCTTGTTTTATTATAATGCTGATAAGTCTTGTAAATAA

GGGTGAGAAGCACGAATAATTAGTTCATGAGATGTGTATAATTATTATCCTACACGACTT

ATCTTTGATATTTCACACAAGTCTTTCAATGTATAACAAAAATTTTTTAGATACATTTAG

ACTATAGAACTAACAAGTTATATTTTGAACC-AAAAAACAAAGAGAGAGAG----GAACA

TAGAGAAAAGAAGATATGAGAGTTTTTTTTTTT--------CTAAAACAGAAAGAAACTC

ATTATATAATAAACAAATTACTTTGAGACAAATTAACGTATGTAATAAAACAAAATCAAA

GTGGTAATTAAAATTATTTAATGGTAAACAGTCTAATAGTTAAAATAAAAATGGAAATCA

CATAAATTTTGTAATTGGCCCATTAAAAACAACACAAAGCTTTTAATTTGATTTTGAAAT

TCAAAATAATTTTATTAAATCACAAAGTAAAAGGTTTACAAAGCCGATCATGACAGTGCA

TGTGGGAGGCAAATCGGCATATTTGCACTACAAAAGG-ACCCATGAAGTCTCTGAACATG

CACGCAACACTTTAATCTCTTATTAGTTACTTTGAAAGCTTATTTATATATATAGACACG

CGTAAAAACTTCTTAACCAAGATTTTTTT-ACGTGCTTCC-TTCGCGTTTAATTTGGACC

ATCAAACCGTGCTCAACAGATAAAGAAAAGGGTGCTTTTGATTCAAGATATTGGCC-GAA

AAACACAAGATAGATCCTT-CGATAGATTAAGCCACGCATGAAACGCGAATCCAAAGTGA

TGAAGAAGTGCAGATAGATATTCGTTCACCATATAGAGGAGAATATATCATTCCTACAAA

CAAAATTGATGATGTTTCTAGAATAGAAACGTTAGAATCGAATATTCATCTGTTCCGGGT

GGGGAAGGGCTACTGAAGAGCTGAACTTTTTTAAGAGTTCGAACTTCGAAAGTCAATCAA

TGATTCTTAATTGAGAGGGAAAAGCTATAAAGGACGAGAAGGGAAGGAAATGTTTATAGG

GAATGAATTTCTAATGCATGATGGGAATGACAGATATGAGGAATATATAAAGGTGATTCT

TATATACATTGACATGGATCGGAATCTATTAACTTAAAGTTATTGGGGTCGTGAAATTTA

TTCAATTTTTCTAATTCGTGTCACATGTCACGACAATTAGTGGGG-AACATTTATGTATA

TAGAAATTTCAGAAATTTCTAGCAGCGTGATAAATATAAAGATTTTGGCACAATAAGTTT

TTGGATCATAATGAGTTT-CTATTTAATCAA-GGCA-TGTAA-TCTATTTTATTATTTAG

-TAGGG-AAACTGAAAACTTAGGGTTGCTATTTGTAGCTCCCACCCTTCTTAGTTCTTAC

TCTTTTCAATATTTTTTATAAGTTTTA-TATAATACCT-AAATT-GCCCTCCTCCTCA-C

CCCCCCTGCTAATCTTCTT-CT-CTCACACATTGCTTCT-CACCC-ATTGCTCTTCCGTT

GCTCCTCCATGTG-AGTTCTAGTCTTTTTTTT--CTCTGGTAGCGTTTTGCTGCTCCTTT

TTTCACTCAAGTGTTGCCAATTAA-TTGACAAAAAATGGCTTCTGTTTCATATAGAAACT

ATGTTTTTGTTGTGTAGTCATGCATTACGGAATCTAGTTT-CCATTAAATAAGTAACATG

TGAAAAAAAA--TAAAAGGTGAAATATATATTGTTGGAAAAGAAGCTATGAGGTGCAAGA

ACCGATCACATGGAGAAGGCAATGAAAGACAAGGAGGAGCAATGAAAGATAAGGAGGAGC

AATGAAAGAGAGAAAATGAGAAGATGGAAGGGATGTGAAAATGTTTGAAAAAGACGAGGT

GATCAGTTTTGAAATACGAATTTAGTATTTTCTTTTTAAGAAAATTCTTTCG-AAAGTCG

TGTTTTAAAACATGACTTTTATT-ATTTGAAGTCG---TGTTCTAAAACATGGCTTA--T

TCATATCCTT-AATATTTTT---------AAAATTTATCCATTTGTAATATTTTTTAAAA

ATTGACCCATATATGTAAAATACCCGTCAAGATCTCTTTATTATTTTGAAAGCGAAAGCA

TATCACTTCAAACACAATGGAATCGAGGCTATTGACTAAGTATAAATAGAGAAGACCTCA

TATCGGGGTTCATAATTCATAACAAAGCAAACGAGTATATAAGAAAGCATAAGCCAAATT

TTGAGTAAACTAGTGTGCACACTATCCCATGCCTAGTGGAAGTAGGGATCCTCTCGTTGT

TGGGGGAGTAATTGGGGACGTATTGGATCCTTTTGAATATTCTATTCCCATGAGGGTTAC

CTACAATAACAGAGATGTCAGCAATGGATGTGAATTCAAACCCTCACAAGTTGTCAACCA

ACCAAGGGTAAATATCGGTGGTGATGACC

>H12.C7

TATTAGACTCCTAATTTAATATTCTTGTTTTGTTATAATGCTGATAAGTCTTGTAAATAA

GGGTGAGAAGCACGAATAATTAGTTCATGAGATGTGTATAATTATTATCCTACACGACTT

ATCTTTGATATTTCACACAAGTCTTTCAATGTATAACAAAAACTTTTTAGATACATTTAG

ACTATAGAACTAACAAGTTATATTTTGAACC-AAAAAACAAAGAGAGAGAG----GAACA

TAGAGAAAAGAAGATATGAGAGTTTTTTTTTT---------CTAAAACAGAAAGAAACTC

ATTATATAATAAACAAATTACTTTGAGACAAATTAACGTATGTAATAAAACAAAATCAAA

GTGGTAATTAAAATTATTTAATGGTAAACAGTCTAATAGTTAAAATAAAAATGGAAATCA

CATAAATTTTGTAATTGGCCCATTAAAAACAACACTAAGCTTTTAATTTGATTTTGAAAT

TCAAAATAATTTTATTAAATCACAAAGTAAAAGGTTTACAAAGCCGATCATGACAGTGCA

TGTGGGAGGCAAATCGGCATATTTGCACTACAAAAGG-ACCCATGAAGTCTCTGAACATG

CACGCAACACTTTAATCTCTTATTAGTTACTTTGAAAGCTTATTTATATATATAGACACG

CGTAAAAACTTCTTAACCAAGATTTTTTT-ACGTGCTTCC-TTCGCGTTTAATTTGGACC

ATCAAACCGTGCTCAACAGATAAAGAAAAGGGTGCTTTTGATTCAAGATATTGGCC-GAA

AAACACAAGATAGATCCTT-CGATAGATTAAGCCACGCATGAAACGCGAATCCAAAGTGA

TGAAGAAGTGCAGATAGATATTCGTTCACCATATAGAGGAGAATATATCATTCCTACAAA

CAAAATTGATGATGTTTCTAGAATAGAAACGTTAGAATCGAATATTCATCTGTTCCGGGT

GGGGAAGGGCTACTGAAAAGCTGAACTTTTTTAAGAGTTCGAACCTCGAAAGTCAATCAA

TGATTCTTAATTGAGAGGGAAAAGCTATAAAGGACGAGAAGGGAAGGAAATGTTTATAGG

GAATGAATTTCTAATGCATGATGGGAATGACAGATATGAGGAATATATAAAGGTGATTCT

TATATACATCGACATGGATCGGAATCTATTAACTTAAAGTTATTGGGGTCGTGAAATTTA

TTTAATTTTTCTAATTCGTGTCACATGTCACGACAATTAGTGGGG-AACATTTATGTATA

TAGAAATTTCAGAAATTTCTAGCAGCGTGATAAATATAAAGATTTTGGCACAATAAGTTT

TTGGATCATAATGAGTTT-CTATTTAATCAA-GGCA-TGTAA-TCTATTTTATTATTTAG

-TAGGG-AAACTGAAAACTTAGGGTTGCTATTTGTAGCTCCCACCCTTCTTAGTTCTTAC

TCTTTTCAATATTTTTTATAAGTTTTA-TATAATACCT-AAATT-GCCCTCCTCCTCA-C

CCCCCCTGCTAATCTTCTTCCTCCTCACACACTGCTTCT-CACCCCATTGCTCTTCCGTT

GCTCCTCCATGTG-AGTTCTAGCCTTTTTTTT--CTCTGGTAGCGTTTTGCTGCTCCTTT

TTTCACTCAAGCGTTGCCAATTAA-TTGACAAAAAATGGTTTCTGTTTCATATAGAAACT

ATGTTTTTGTTGTGTAGTCATACATTACGGAATCTAGTTT-CCATTAAATAAGTAACATG

TGAAAAAAAA--TAAAAGGTGAAATATATATTGTTGGAAAAGAAGCTATGAGGTGCAAGA

ACCGATCACATGGAGAAGGCAATGAAAGACAAGGAGGAGCAATGAAAGACAAGGAGGAGC

AATGAAAGAGAGAAAATGAGAAGATGGAAGGGATGTGAAAATGTTTGAAAAAGACGAGGT

GATCAGTTTTGAAATACGAATTTAATATTTTCTTTTTAAGAAAATTCTTTCG-AAAGTCG

TGTTTTAAAACATGACTTTTATT-ATTTGAAGTCG---TGTTCTAAAACATGACTTA--T

TCATATCCTT-AATATTTTT---------AAAATTTATCCATTTGTAATATTTTTTAAAA

ATTGACCCATATATGTAAAATACCCGTCAAGATCTCTTTATTATTTTGAAAGCGAAAGCA

TATCACTTCAAACACAATGGAATCGAGGCTATTGACTAAGTATAAATAGAGAAGACTTCA

TATCGGGGTTCATAATTCATAACAAAGCAAACGAGTATATAAGAAAGCATAAGCCAAATT

TTGAGTAAACTAGTGTGCACACTACCCCATGCCTAGTGGAAGTAGGGATCCTCTCGTTGT

TGGGGGAGTAATTGGGGATGTATTGGATCCTTTTGAATATTCTATTCCTATGAGGGTTAC

CTACAATAACAGAGATGTCAGCAATGGATGTGAATTCAAACCCTCACAAGTTGTCAACCA

ACCAAGGGTAAATATCGGTGGTGATGACC

>H16.31

TATTAGACTCCTAATTTAATATTCTTGTTTTATTATAATGCTGATAAGTCTTGTAAATAA

GGGTGAGAAGCACGAATAATTAGTTCATGAGATGTGTATAATTATTATCCTACACGACTT

ATCTTTGATATTTCACACAAGTCTTTCAATGTATAACAAAAACTTTTTAGATACATTTAG

ACTATAGAACTAACAAGTTATATTTTGAACC-AAAAAACAAAGAGAGAGAG----GAACA

TAGAGAAAAGAAGATATGAGAGTTTTTTTTTT---------CTAAAACAGAAAGAAACTC

ATTATATAATAAACAAATTACTTTGAGACAAATTAACGTATGTAATAAAACAAAATCAAA

GTGGTAATTAAAATTATTTAATGGTAAGCAGTCTAATAGTTAAAATAAAAATGGAAATCA

CATGAATTTTGTAATTGGCCCATTAAAAACAACACTAAGCTTTTAATTTGATTTTGAAAT

TCAAAATAATTTTATTAAATCACAAAGTAAAAGGTTTACAAAGCCGATCATGACAGTGCA

TGTGGGAGGCAAATCGGCATATTTGCACTACAAAAGG-ACCCATGAAGTCTCTGAACATG

CACGCAACACTTTAATCTCTTATTAGTTACTTTGAAAGCTTATTTATATATATAGACACG

CGTAAAAACTTCTTAACCAAGATTTTTTT-ACGTGCTTCC-TTCGCGCTTAATTTGGACC

ATCAAACCGTGCTCAACAGATAAAGAAAAGGGTGCTTTTGATTCAAGATATTGGCC-GAA

AAACACAAGATAGATCCTT-CGATAGATTAAGCCACGCATGAAACGCGAATCCAAAGTGA

TGAAGAAGTGCAGATAGATATTCGTTCGCCATATAGAGGAGAATATATCATTCCTACAAA

CAAAATTGATGATGTTTCTAGAATAGAAACGTTAGAATCGAATATTCATCTGTTCCGGGT

GGGGAAGGGCTACTGAAAAGCTGAACTTTTTTAAGAGTTCGAACTTCGAAAGTCAATCAA

TGATTCTTAATTGAGAGGGAAAAGCTATAAAGGACGAGAAGGGAAGGAAATGTTTATAGG

GAATGAATTTCTAATGCATGATGGGAATGACAGATATGAGGAATATATAAAGGTGATTCT

TATATACATCGACATGGATCGGAATCTATTAACTTAAAGTTATTGGGGTCGTGAAATTTA

TTTAATTTTTCTAATTCGTGTCACATGTCACGACAATTAGTGGGG-AACATTTATGTATA

TAGAAATTTCAGAAATTTCTAGCAGCGTGATAAATATAAAGATTTTGGCACAATAAGTTT

TTGGATCATAATGAGTTT-CTATTTAATCAA-GGCA-TGTAA-TCTATTTTATTATTTAG

-TAGGG-AAACTGAAAACTTAGGGTTGCTATTTGTAGCTCCCACCCTTCTTAGTTCTTAC

TCTTTTCAATATTTTTTATAAGTTTTA-TATAATACCT-AAATT-GCCCTCCTCCTCA-C

CCCCCCTGCTAATCTTCTTCCTCCTCACACATTGCTTCT-CACCCCATTGCTCTTCCGTT

GCTCCTCCATGTG-AGTTCTAGTCTTTTTTTT--CTCTGGTAGCGTTTTGCTGCTCCTTT

TTTCACTCAAGTGTTGCCAATTAA-TTGACAAAAAATGGTTTCTGTTTCATATAGAAACT

ATGTTTTTGTTGTGTAGTCATACATTACGGAATCTAGTTT-CCATTAAATAAGTAACATG

TGAAAAAAAA--TAAAAGGTGAAATATATATTGTTGGAAAAGAAGCTATGAGGTGCAAGA

ACCGATCACATGGAGAAGGCAATGAAAGACAAGGAGGAGCAATGAAAGACAAGGAGGAGC

AATGAAAGAGAGAAAATGAGAAGATGGAAGGGATGTGAAAATGTTTGAAAAAGACGAGGT

GATCAGTTTTGAAATACGAATTTAATATTTTCTTTTTAAGAAAATTCTTTCG-AAAGTCG

TGTTTTAAAACATGACTTTTATT-ATTTGAAGTCG---TGTTCTAAAACATGACTTA--T

TCATATCCTT-AATATTTTT---------AAAATTTATCCATTTGTAATATTTTTTAAAA

ATTGACCCATATATGTAAAATACCCGTCAAGATCTCTTTATTATTTTGAAAGCGAAAGCA

TATCACTTCAAACACAATGGAATCGAGGCTATTGACTAAGTATAAATAGAGAAGACTTCA

TATCGGGGTTCATAATTCATAACAAAGCAAACGAGTATATAAGAAAGCATAAGCCAAATT

TTGAGTAAACTAGTGTGCACACTATCCCATGCCTAGTGGAAGTAGGGATCCTCTCGTTGT

TGGGGGAGTAATTGGGGATGTATTGGATCCTTTTGAATATTCTATTCCTATGAGGGTTAC

CTACAATAACAGAGATGTCAGCAATGGATGTGAATTCAAACCCTCACAAGTTGTCAACCA

ACCAAGGGTAAATATCGGTGGTGATGACC

>H17.X8

TATTAGACTCCTAATTTAATATTCTTGTTTTATTATAATGCTGATAAGTCTTGTAAATAA

GGGTGAGAAGCACGAATAATTAGTTCATGAGATGTGTATAATTATTATCCTACACGACTT

ATCTTTGATATTTCACACAAGTCTTTCAATGTATAACAAAAACTTTTTAGATACATTTAG

ACTATAGAACTAACAAGTTATATTTTGAACC-AAAAAACAAAGAGAGAGAG----GAACA

TAGAGAAAAGAAGATATGAGAGTTTTTTTTTT---------CTAAAACAGAAAGAAACTC

ATTATATAATAAACAAATTACTTTGAGACAAATTAACGTATGTAATAAAACAAAATCAAA

GTGGTAATTAAAATTATTTAATGGTAAACAGTCTAATAGTTAAAATAAAAATGGAAATCA

CATAAATTTTGTAATTGGCCCATTAAAAACAACACTAAGCTTTTAATTTGATTTTGAAAT

TCAAAATAATTTTATTAAATCACAAAGTAAAAGGTTTACAAAGCCGATCATGACAGTGCA

TGTGGGAGGCAAATCGGCATATTTGCACTACAAAAGG-ACCCATGAAGTCTCTGAACATG

CACGCAACACTTTAATCTCTTATTAGTTACTTTGAAAGCTTATTTATATATATAGACACG

CGTAAAAACTTCTTAACCAAGATTTTTTT-ACGTGCTTCC-TTCGCGTTTAATTTGGACC

ATCAAACCGTGCTCAACAGATAAAGAAAAGGGTGCTTTTGATTCAAGATATTGGCC-GAA

AAACACAAGATAGATCCTTTCGATAGATTAAGCCACGCATGAAACGCGAATCCAAAGTGA

TGAAGAGGTGCAGATAGATATTCGTTCACCATATAGAGGAGAATATATCATTCCTACAAA

CAAAATTGATGATGTTTCTAGAATAGAAACGTTAGAATCGAATATTCATCTGTTCCGGGT

GGGGAAGGGCTACTGAAAAGCTGAACTTTTTTAAGAGTTCGAACTTCGAAAGTCAATCAA

TGATTCTTAATTGAGAGGGAAAAGCTATAAAGGACGAGAAGGGAAGGAAATGTTTATAGG

GAATGAATTTCTAATGCATGATGGGAATGACAGATATGAGGAATATATAAAGGTGATTCT

TATATACATCGACATGGATCGGAATCTATTAACTTAAAGTTATTGGGGTCGTGAAATTTA

TTTAATTTTTCTAATTCGTGTCACATGTCACGACAATTAGTGGGG-AACATTTATGTATA

TAGAAATTTCAGAAATTTCTAGCAGCGTGATAAATATAAAGATTTTGGCACAATAAGTTT

TTGGATCATAATGAGTTT-CTATTTAATCAA-GGCA-TGTAA-TCTATTTTATTATTTAG

-TAGGG-AAACTGAAAACTTAGGGTTGCTATTTGTAGCTCCCACCCTTCTTAGTTCTTAC

TCTTTTCAATATTTTTTATAAGTTTTA-TATAATACCT-AAATTTGCCCTCCTCCTCA-C

CCCCCCTGCTAATCTTCTTCCTCCTCACACATTGCTTCT-CACCCCATTGCTCTTCCGTT

GCTCCTCCATGTG-AGTTCTAGTCTTTTTTTT--CTCTGGTAGCGTTTTGCTGCTCCTTT

TTTCACTCAAGTGTTGCCAATTAA-TTGACAAAAAATGGTTTCTGTTTCATATAGAAACT

ATGTTTTTGTTGTGTAGTCATACATTACGGAATCTAGTTT-CCATTAAATAAGTAACATG

TGAAAAAAAA--TAAAAGGTGAAATATATATTGTTGGAAAAGAAGCTATGAGGTGCAAGA

ACCGATCACATGGAGAAGGCAATGAAAGACAAGGAGGAGCAATGAAAGACAAGGAGGAGC

AATGAAAGAGAGAAAATGAGAAGATGGAAGGGATGTGAAAATGTTTGAAAAAGACGAGGT

GATCAGTTTTGAAATACGAATTTAATATTTTCTTTTTAAGAAAATTCTTTCG-AAAGTCG

TGTTTTAAAACATGACTTTTATT-ATTTGAAGTCG---TGTTCTAAAACATGACTTA--T

TCATATCCTT-AATATTTTT---------AAAATTTATCCATTTGTAATATTTTTTAAAA

ATTGACCCATATATGTAAAATACCCGTCAAGATCTCTTTATTATTTTGAAAGCGAAAGCA

TATCACTTCAAACACAATGGAATCGAGGCTATTGACTAAGTATAAATAGAGAAGACTTCA

TATCGGGGTTCATAATTCATAACAAAGCAAACGAGTATATAAGAAAGCATAAGCCAAATT

TTGAGTAAACTAGTGTGCACACTATCCCATGCCTAGTGGAAGTAGGGATCCTCTCGTTGT

TGGGGGAGTAATTGGGGATGTATTGGATCCTTTTGAATATTCTATTCCTATGAGGGTTAC

CTACAATAACAGAGATGTCAGCAATGGATGTGAATTCAAACCCTCACAAGTTGTCAACCA

ACCAAGGGTAAATATCGGTGGTGATGACC

>H16.28c

TATTAGACTCCTAATTTAATATTCTTGTTTTATTATAATGCTGATAAGTCTTGTAAATAA

GGGTGAGAAGCACGAATAATTAGTTCATGAGATGTGTATAATTATTATCCTACACGACTT

ATCTTTGATATTTCACACAAGTCTTTCAATGTATAACAAAAACTTTTTAGATACATTTAG

ACTATAGAACTAACAAGTTATATTTTGAACC-AAAAAACAAAGAGAGAGAG----GAACA

TAGAGAAAAGAAGATATGAGAGTTTTTTTTTT---------CTAAAACAGAAAGAAACTC

ATTATATAATAAACAAATTACTTTGAGACAAATTAACGTATGTAATAAAACAAAATCAAA

GTGGTAATTAAAATTATTTAATGGTAAACAGTCTAATAGTTAAAATAAAAATGGGAATCA

CATAAATTTTGTAATTGGCCCATTAAAAACAACACTAAGCTTTTAATTTGATTTTGAAAT

TCAAAATAATTTTATTAAATCACAAAGTAAAAGGTTTACAAAGCCGATCATGACAGTGCA

TGTGGGAGGCAAATCGGCATATTTGCACTACAAAAGG-ACCCATGAAGTCTCTGAACATG

CACGCAACACTTTAATCTCTTATTAGTTACTTTGAAAGCTTATTTATATATATAGACACG

CGTAAAAACTTCTTAACCAAGATTTTTTT-ACGTGCTTCC-TTCGCGTTTAATTTGGACC

ATCAAACCGTGCTCAACAGATAAAGAAAAGGGTGCTTTTGATTCAAGATATTGGCC-GAA

AAACACAAGATAGATCCTT-CGATAGATTAAGCCACGCATGAAACGCGAATCCAAAGTGA

TGAAGAAGTGCAGATAGATATTCGTTCACCATATAGAGGAGAATATATCATTCCTACAAA

CAAAATTGATGATGTTTCTAGAATAGAAACGTTAGAATCGAATATTCATCTGTTCCGGGT

GGGGAAGGGCTACTGAAAAGCTGAACTTTTTTAAGAGTTCGAACTTCGAAAGTCAATCAA

TGATTCTTAATTGAGAGGGAAAAGCTATAAAGGACGAGAAGGGAAGGAAATGTTTATAGG

GAATGAATTTCTAATGCATGATGGGAATGACAGATATGAGGAATATATAAAGGTGATTCT

TATATACATCGACATGGATCGGAATCTATTAACTTAAAGTTATTGGGGTCGTGAAATTTA

TTTAATTTTTCTAATTCGTGTCACATGTCACGACAATTAGTGGGG-AACATTTATGTATA

TAGAAATTTCAGAAATTTCTAGCAGCGTGATAAATATAAAGATTTTGGCACAATAAGTTT

TTGGATCATAATGAGTTT-CTATTTAATCAA-GGCA-TGTAA-TCTATTTTATTATTTAG

-TAGGG-AAACTGAAAACTTAGGGTTGCTATTTGTAGCTCCCACCCTTCTTAGTTCTTAC

TCTTTTCAATATTTTTTATAAGTTTTA-TATAATACCT-AAATT-GCCCTCCTCCTCA-C

CCCCCCTGCTAATCTTCTTCCTCCTCACACATTGCTTCT-CACCCCATTGCTCTTCCGTT

GCTCCTCCATGTG-AGTTCTAGTCTTTTTTTT--CTCTGGTAGCGTTTTGCTGCTCCTTT

TTTCACTCAAGTGTTGCCAATTAA-TTGACAAAAAATGGTTTCTGTTTCATATAGAAACT

ATGTTTTTGTTGTGTAGTCATACATTACGGAATCTAGTTT-CCATTAAATAAGTAACATG

TGAAAAAAAA--TAAAAGGTGAAATATATATTGTTGGAAAAGAAGCTATGAGGTGCAAGA

ACCGATCACATGGAGAAGGCAATGAAAGACAAGGAGGAGCAATGAAAGACAAGGAGGAGC

AATGAAAGAGAGAAAATGAGAAGATGGAAGGGATGTGAAAATGTTTGAAAAAGACGAGGT

GATCAGTTTTGAAATACGAATTTAATACTTTCTTTTTAAGAAAATTCTTTCG-AAAGTCG

TGTTTTAAAACATGACTTTTATT-ATTTGAAGTCG---TGTTCTAAAACATGACTTA--T

TCATATCCTT-AATATTTTT---------AAAATTTATCCATTTGTAATATTTTTTAAAA

ATTGACCCATATATGTAAAATACCCGTCAAGATCTCTTTATTGTTTTGAAAGCGAAAGCA

TATCACTTCAAACACAATGGAATCGAGGCTATTGACTAAGTATAAATAGAGAAGACTTCA

TATCGGGGTTCATAATTCATAACAAAGCAAACGAGTATATAAGAAAGCATAAGCCAAATT

TTGAGTAAACTAGTGTGCACACTATCCCATGCCTAGTGGAAGTAGGGATCCTCTCGTTGT

TGGGGGAGTAATTGGGGATGTATTGGATCCTTTTGAATATTCTATTCCTATGAGGGTTAC

CTACAATAACAGAGATGTCAGCAATGGATGTGAATTCAAACCCTCACAAGTTGTCAACCA

ACCAAGGGTAAATATCGGTGGTGATGACC

>J1.14

TATTAGACTCCTAATTTAATATTCTTGTTTTATTATAATGCTGATAAGTCTTGTAAATAA

GGGTGAGAAGCACGAATAATTAGTTCATGAGATGTGTATAATTATTATCCTACACGACTT

ATCTTTGATATTTCACACAAGTCTTTCAATGTGTAACAAAAACTTTTTAGATACATTTAG

ACTATAGAACTAACAAGTTATATTTTGAACC-AAAAAACAAAGAGAGAGAG----GAACA

TAGAGAAAAGAAGATATGAGAGTTTTTTTTTT---------CTAAAACAGAAAGAAACTC

ATTATATAATAAACAAATTACTTTGAGACAAATTAACGTATGTAATAAAACAAAATCAAA

GTGGTAATTAAAATTATTTAATGGTAAACAGTCTAATAGTTAAAATAAAAATGGAAATCA

CATAAATTTTGTAATTGGCCCATTAAAAACAACACTAAGCTTTTAATTTGATTTTGAAAT

TCAAAATAATTTTATTAAATCACAAAGTAAAAGGTTTACAAAGCCGATCATGACAGTGCA

TGTGGGAGGCAAGTCGGCATATTTGCACTACAAAAGG-ACCCATGAAGTCTCTGAACATG

CACGCAACACTTTAATCTCTTATTAGTTACTTTGAAAGCTTATTTATATATATAGACACG

CGTAAAAACTTCTTAACCAAGATTTTTTT-ACGTGCTTCC-TTCGCGTTTAATTTGGACC

ATCAAACCGTGCTCAACAGATAAAGAAAAGGGTGCTTTTGATTCAAGATATTGGCC-GAA

AAACACAAGATAGATCCTT-CGATAGATTAAGCCACGCATGAAACGCGAATCCAAAGTGA

TGAAGAAGTGCAGATAGATATTCGTTCACCATATAGAGGAGAATATATCATTCCTACAAA

CAAAATTGATGATGTTTCTAGAATAGAAACGTTAGAATCGAATATTCATCTGTTCCGGGT

GGGGAAGGGCTACTGAAAAGCTGAACTTTTTTAAGAGTTCGAACTTCGAAAGTCAATCAA

TGATTCTTAATTGAGAGGGAAAAGCTATAAAGGACGAGAAGGGAAGGAAATGTTTATAGG

GAATGAATTTCTAATGCATGATGGGAATGACAGATATGAGGAATATATAAAGGTGATTCT

TATATACATTGACATGGATCGGAATCTATTAACTTAAAGTTATTGGGGTCGTGAAATTTG

TTTAATTTTTCTAATTCGTGTCACATGTCACGACAATTAGTGGGG-AACATTTATGTATA

TAGAAATTTCAGAAATTTCTAGCAGCGTGATAAATATAAAGATTTTGGCACAATAAGTTT

TTGGATCATAATGAGTTT-CTATTTAATCAA-GGCA-TGTAA-TCTATTTTATTATTTAG

-TAGGG-AAACTGAAAACTTAGGGTTGCTATTTGTAGCTCCCACCCTTCTTAGTTCTTAC

TCTTTTCAATATTTTTTATAAGTTTTA-TATAATACCT-AAATT-GCCCTCCTCCTCA-C

CCCCCCTGCTAATCTTCTTCCTCCTCACACATTGCTTCT-CACCCCATTGCTCTTCCGTT

GCTCCTCCATGTG-AGTTCTAGTCTTTTTTTT--CTCTGGTAGCGTTTTGCTGCTCCTTT

TTTCACTCAAGTGTTGCCAATTAA-TTGACAAAAAATGGTTTCTGTTTCATATAGAAACT

ATGTTTTTGTTGTGTAGTCATACATTACGGAATCTAGTTT-CCATTAAATAAGTAACATG

TGAAAAAAAA--TAAAAGGTGAAATATATATTGTTGGAAAAGAAGCTATGAGGTGCAAGA

ACCGATCACATGGAGAAGGCAATGAAAGACAAGGAGGAGCAATGAAAGA-----------

---------GAGAAAATGAGAAGATGGAAAGGATGTGAAAATGTTTGAAAAAGACGAGGT

GATCAGTTTTGAAATACGAATTTAGTATTTTCTTTTTAAGAAAATTCTTTCG-AAAGTCG

TGTTTTAAAACATGACTTTTATT-ATTTGAAGTTG---TGTTCTAAAACATGACTTA--T

TCATATCCTT-AATATTTTT---------AAAATTTATCCATTTGTAATATTTTTTAAAA

ATTGACCCATATATGTAAAATACCCGTCAAGATCTCTTTATTATTTTGAAAGCGAAAGCA

TATCACTTCAAACACAATGGAATCGAGGCTATTGACTAAGTATAAATAGAGAAGACTTCA

TATCGGGGTTCATAATTCATAACAAAGCAAACGAGTATATAAGAAAGCATAAGCCAAATT

TTGGGTAAACTAGTGTGCACACTATCCCATGCCTAGTGGAAGTAGGGATCCTCTCGTTGT

TGGGGGAGTAATTGGGGATGTATTGGATCCTTTTGAATATTCTATTCCTATGAGGGTTAC

CTACAATAACAGAGATGTCAGCAATGGATGTGAATTCAAACCCTCACAAGTTGTCAACCA

ACCAAGGGTAAATATCGGTGGTGATGACC

>J1.15

TATTAGACTCCTAATTTAATATTCTTGTTTTATTATAATGCTGATAAGTCTTGTAAATAA

GGGTGAGAAGCACGAATAATTAGTTCATGAGATGTGTATAATTATTATCCTACACGACTT

ATCTTTGATATTTCACACAAGTCTTTCAATGTATAACAAAAACTTTTTAGATACATTTAG

ACTATAGAACTAACAAGTTATATTTTGAACC-AAAAAACAAAGAGAGAGAG----GAACA

TAGAGAAAAGAAGATATGAGAGTTTTTTTTTT---------CTAAAACAGAAAGAAACTC

ATTATATAATAAACAAATTACTTTGAGACAAATTAACGTATGTAGTAAAACAAAATCAAA

GTGGTAATTAAAATTATTTAATGGTAAACAGTCTAATAGTTAAAATAAAAATGGAAATCA

CATAAATTTTGTAATTGGCCCATTAAAAACAACACTAAGCTTTTAATTTGATTTTGAAAT

TCAAAATAATTTTATTAAATCACAAAGTAAAAGGTTTACAAAGCCGATCATGACAGTGCA

TGTGGGAGGCAAATCGGCATATTTGCACTACAAAAGG-ACCCATGAAGTCTCTGAACATG

CACGCAACACTTTAATCTCTTATTAGTTACTTTGAAAGCTTATTTATATATATAGACACG

CGTAAAAACTTCTTAACCAAGATTTTTTT-ACGTGCTTCC-TTCGCGTTTAATTTGGACC

ATCAAACCGTGCTCAACAGATAAAGAAAAGGGTGCTTTTGATTCAAGATATTGGCC-GAA

AAACACAAGATAGATCCTT-CGATAGATTAAGCCACGCATGAAACGCGAATCCAAAGTGA

TGAAGAAGTGCAGATAGATATTCGTTCACCATATAGAGGAGAATATATCATTCCTACAAA

CAAAATTGATGATGTTTCTAGAATAGAAACGTTAGAATCGAATATTCATCTGTTCCGAGT

GGGGAAGGGCTACTGAAAAGCCGAACTTTTTTAAGAGTTCGAGCTTCGAAAGTCAATCAA

TGATTCTTAATTGAGAGGGAAAAGCTATAAAGGACGAGAAGGGAAGGAAATGTTTATAGG

GAATGAATTTCCAATGCATGATGGGAATGACAGATATGAGGAATATATAAAGGTGATTCT

TATATACATTGACATGGATCGGAATCTATTAACTTAAAGTTATTGGGGTCGTGAAATTTA

TTTAATTTTTCTAATTCGTGTCACATGTCACGACAATTAGTGGGG-AACATTTATGTATA

TAGAAATTTCAGAAATTTCTAGCAGCGTGATAAATATAAAGATTTTGGCACAATAAGTTT

TTGGATCATAATGAGTTT-CTATTTAATCAA-GGCA-TGTAA-TCTATTTTATTATTTAG

-TAGGG-AAACTGAAAACTTAGGGTTGCTATTTGTAGCTCCCACCCTTCTTAGTTCTTAC

TCTTTTCAATATTTTTTATAAGTTTTA-TATAATACCT-AAATT-GCCCTCCTCCTCA-C

CCCCCCTGCTAATCTTCTTCCTCCTCACACATTGCTTCT-CACCCCATTGCTCTTCCGTT

GCTCCTCCATGTG-AGTTCTTGTCTTTTTTTTT-CTCTGGTAGCGTTTTGCTGCTCCTTT

TTTCACTCAAGTGTTGCCAATTAA-TTGACAAAAAATGGTTTCTGTTTCATATAGAAACT

ATGTTTTTGTTGTGTAGTCATACATTACGGAATCTAGTTT-CCATTAAATAAGTAACATG

TGAAAAAAAA--TAAAAGGTGAAATATATATTGTTGGAAAAGAAGCTATGAGGTGCAAGA

ACCGATCACATGGAGAAGGCAATGAAAGACAAGGAGGAGCAATGAAAGA-----------

---------GAGAAAATGAGAAGATGGAAAGGATGTGAAAATGTTTGAAAAAGACGAGGT

GATCAGTTTTGAAATACGAATTTAGTATTTTCTTTTTAAGAAAATTCTTTCG-AAAGTCG

TGTTTTAAAACATGACTTTTATT-ATTTGAAGTTG---TGTTCTAAAACATGACTTA--T

TCATATCCTT-AATATTTTT---------AAAATTTATCCATTTGTAATATTTTTTAAAA

ATTGACCCATATATGTAAAATACCCGTCAAGATCTCTTTATTATTTTGAAAGCGAAAGCA

TATCACTTCAAACACAATGGAATCGAGGCTATTGACTAAGTATAAATAGAGAAGACTTCA

TATCGGGGCTCATAATTCATAACAAAGCAAACGAGTATATAAGAAAGCATAAGCCAAATT

TTGAGTAAACTAGTGTGCACACTATCCCATGCCTAGTGGAAGTAGGGATCCTCTCGTTGT

TGGGGGAGTAATTGGGGATGTATTGGATCCTTTTGAATATTCTATTCCTATGAGGGTTAC

CTACAATAACAGAGATGTCAGCAATGGATGTGAATTCAAACCCTCACAAGTTGTCAACCA

ACCAAGGGTAAATATCGGTGGTGATGACC

>H07.02

TATTAGACTCCTAATTTAATATTCTTGTTTTATTATAATGCTGATAAGTCTTGTAAATAA

GGGTGAGAAGCACGAATAATTAGTTCATGAGATGTGTATAATTATTATCCTACACGACTT

ATCTTTGATATTTCACACAAGTCTTTCAATGTATAACAAAAACTTTTTAGATACATTTAG

ACTATAGAACTAACAAGTTATATTTTGAACC-AAAAAACAAAGAGAGAGAG----GAACA

TAGAGAAAAGAAGATATGAGAGTTTTTTTTTT---------CTAAAACAGAAAGAAACTC

ATTATATAATAAACAAATTACTTTGAGACAAATTAACGTATGTAATAAAACAAAATCAAA

GTGGTAATTAAAATTATTTAATGGTAAACAGTCTAATAGTTAAAATAAAAATGGAAATCA

CATAAATTTTGTAATTGGCCCATTAAAAACAACACTAAGCTTTTAATTTGATTTTGAAAT

TCAAAATAATTTTATTAAATCACAAAGTAAAAGGTTTACAAAGCCGATCATGACAGTGCA

TGTGGGAGGCAAATCGGCATATTTGCACTACAAAAGG-ACCCGTGAAGTCTCTGAACATG

CACGCAACACTTTAATCTCTTATTAGTTACTTTGAAAGCTTATTTATATATATAGACACG

CGTAAAAACTTCTTAACCAAGATTTTTTT-ACGTGCTTCC-TTCGCGTTTAATTTGGACC

ATCAAACCGTGCTCAACAGATAAAGAAAAGGGTGCTTTTGATTCAAGATATTGGCC-GAA

AAACACAAGATAGATCCTT-CGATAGATTAAGCCACGCATGAAACGCGAATCCAAAGTGA

TGAAGAAGTGCAGATAGATATTCGTTCACCATATAGAGGAGAATATATCATTCCTACAAA

CAAAATTGATGATGTTTCTAGAATAGAAACGTTAGAATCGAATATTCATCTGTTCCGGGT

GGGGAAGGGCTACTGAAAAGCTGAACTTTTTTAAGAGTTCGAACTTCGAAAGTCAATCAA

TGATTCTTAATTGAGAGGGAAAAGCTATAAAGGACGAGAAGGGAAGGAAATGTTTATAGG

GAATGAATTTCTAATGCATGATGGGAGTGACAGATATGAGGAATATATAAAGGTGATTCT

TATATACATTGACATGGATCGGAATCTATTAACTTAAAGTTATTGGGGTCGTGAAATTTA

TTTAATTTTTCTAATTCGTGTCACATGTCACGACAATTAGTGGGG-AACATTTATGTATA

TAGAAATTTCAGAAATTTCTAGCAGCGTGATAAATATAAAGATTTTGGCACAATAAGTTT

TTGGATCATAATGAGTTT-CTATTTAATCAA-GGCA-TGTAA-TCTATTTTATTATTTAG

-TAGGG-AAACTGAAAACTTAGGGTTGCTATTTGTAGCTCCCACCCTTCTTAGTTCTTAC

TCTTTTCAATATTTTTTAAAAGTTTTA-TATAATACCT-AAATT-GCCCTCCTCCT----

----------------------------------------CACCCCATG-CTCTTCG--T

GCTCCTCCATGTG-AGTCTAGTCTTTTTTTT---CTCTGGTAGCGTTT-GCTGCTCCTTT

TTTCACTCAAGTGTTGCCAATTAA-TTGACAAAAAATGGTTTCTGTTTCATGTAGAAACT

ATGTTTTTGTTGTGTAGTCATACATTACGGAATCTAGTTT-CCATTAAATAAGTAACATG

TGAAAAAAAA--TAAAAGGTGAAATATATATTGTTGGAAAAGAAGCTATGAGGTGCAAGA

ACCGATCACATGGAGAAGGCAATGAAAGACAAGGAGGAGCAATGGAAGA-----------

---------GAGAAAATGAGAAGATGGAAGGGATGTGAAAATGTTTGAAAAAGACGAGGT

GATCAGTTTTGAAATACGAATTTAGTATTTTCTTTTTAAGAAAATTCTTTCG-AAAGTCG

TGTTTTAAAACATGACTTTTATT-ATTTGAAGTCG---TGTTCTAAAACATGACTTA--T

TCATATCCTT-AATATTTTT---------AAAATTTATCCATTTGTAATATTTTTTAAAA

ATTGACCCATATATGTAAAATACCCGTCAAGATCTCTTTATTATTTTGAAAGCGAAAGCA

TATCACTTCAAACACAATGGAATCGAGGCTATTGACTAAGTATAAATAGAGAAGACTTCA

TATCGGGGTTCATAATTCATAACAAAGCAAACGAGTATATAAGAAAGCATAAGCCAAATT

TTGAGTAAACTAGTGTGCACACTATCCCATGCCTAGTGGAAGTAGGGATCCTCTCGTTGC

TGGGGGAGTAATTGGGGATGTATTGGATCCTTTTGAATATTCTATTCCTATGAGGGTTAC

CTACAATAACAGAGATGTCAGCAATGGATGTGAATTCAAACCCTCACAAGTTGTCAACCA

ACCAAGGGTAAATATCGGTGGTGATGACC

>H09.05

TATTAGACTCCTAATTTAATATTCTTGTTTTATTATAATGCTGATAAGTCTTGTAAATAA

GGGTGAGAAGCACGAATAATTAGTTCATGAGATGTGTATAATTATTATCCTACACGACTT

ATCTTTGATATTTCACACAAGTCTTTCAATGAATAACAAAAACTTTTTAGATACATTTAG

ACTATAGAACTAACAAGTTATATTTTGAACC-AAAAAACAAAGAGAGAGAG----GAACA

TAGAGAAAAGAAGATATGAGAGTTTTTTTTTAT--------CTAAAACAGAAAGAAACTC

ATTATATAATAAACAAATTACTTTGAGACAAATTAACGTATGTAATAAAACAAAATCAAA

GTGGTAATTAAAATTATTTAATGGTAAACAGTCTAATAGTTAAAATAAAAATGGAAATCA

CATAAATTTTGTAATTGGCCCATTAAAAACAACACTAAGCTTTTAATTTGATTTTGAAAT

TCAAAATAATTTTATTAAATCACAAAGTAAAAGGTTTACAAAGCCGATCATGACAGTGCA

TGTGGGAGGCAAATCGGCATATTTGCACTACAAAAGG-ACCCATGAAGTCTCTGAACATG

CACGCAACACTTTAATCTCTTATTAGTTACTTTGAAAGCTTATTTATATATATAGACACG

CGTAAAAACTTCTTAACCAAGATTTTTT--ACGTGCTTCCCTTCGCGTTTAATTTGGACC

ATCAAACCGTGCTCAACAGATAAAGAAAAGGGTGCTTTTGATTCAAGATATTGGCC-GAA

AAACACAAGATAGATCCTT-CGATAGATTAAGCCACGCATGAAACGCGAATCCAAAGTGA

TGAAGAAGTGCAGATAGATATTCGTTCACCATATAGAGGAGAATATATCATTCCTACAAA

CAAAATTGATGATGTTTCTAGAATAGAAACGTTAGAATCGAATATTCATCTGTTCCGGGT

GGGGAAGGGCTACTGAAAAGCTGAACTTTTTTAAGAGTTCGAACTTCGAAAGTCAATCAA

TGATTCTTAATTGAGAGGGAAAAGCTATAAAGGACGAGAAGGGAAGGAAATGTTTATAGG

GAATGAATTTCTAATGCATGATGGGAATGACAGATATGAGGAATATATAAAGGTGATTCT

TATATACATCGACATGGATCGGAATCTATTAACTTAAAGTTATTGGGGTCGTGAAATTTA

TTTAATTTTTCTAATTCGTGTCACATGTCACGACAATTAGTGGGG-AACATTTATGTATA

TAGAAATTTCAGAAATTTCTAGCAGCGTGATAAATATAAAGATTTTGGCACAATAAGTTT

TTGGATCATAATGAGTTT-CTATTTAATCAA-GGCA-TGTAA-TCTATTTTATTATTTAG

-TAGGG-AAACTGAAAACTTAGGGTTGCTATTTGTAGCTCCCACCCTTCTTAGTTCTTAC

TCTTTTCAATATTTTTTAAAAGTTTTA-TATAATACCT-AAATT-GCCCTCCTCCT----

----------------------------------------CACCCCATTTCTCTTCCGTT

GCTCCTCCATGTG-AGTTCTTGTCTTTTTTT---CTCTGGTAGCGTTTTGCTGCTCCTTT

TTTCACTCAAGTGTTGCCAATTAA-TTGACAAAAAATGGTTTCTGTTTCATATAGAAACT

ATGTTTTTGTTGTGTAGTCATACATTACGGAATCTAGTTT-CCATTAAATAAGTAACATG

TGAAAAAAAA--TAAAAGGTGAAATATATATTGTTGGAAAAGAAGCTATGAGGTGCAAGA

ACCGATCACATGGAGAAGGCAATGAAAGACAAGGAGGAGCAATGGAAGA-----------

---------GAGAAAATGAGAAGATGGAAGGGATGTGAAAATGTTTGAAAAAGACGAGGG

GATCAGTTTTGAAATACGAATTTAGTATTTTTTTTTTAAGAAAATTCTTTCG-AAAGTCG

TGTTTTAAAACATGACTTTTATT-ATTTGAAGTCG---TGTTCTAAAACATGACTTA--T

TCATATCCTT-AATATTTTT---------AAAATTTATCCATTTGTAATATTTTTTAAAA

ATTGACCCATATATGTAAAATACCCGTCAAGATCTCTTTATTATTTTGAAAGCGAAAGCA

TATCACTTCAAACACAATGGAATCGAGGCTATTGACTAAGTATAAATAGGGAAGACTTCA

TATCGGGGTTCATAATTCATAACAAAGCAAACGAGTATATAAGAAAGCATAAGCCAAATT

TTGAGTAAACTAGTGTGCACACTATCCCATGCCTAGTGGAAGTAGGGATCCTCTCGTTGT

TGGGGGAGTAATTGGGGATGTATTGGATCCTTTTGAATATTCTATTCCTATGAGGGTTAC

CTACAATAACAGAGATGTCAGCAATGGATGTGAATTCAAACCCTCACAAGTTGTCAACCA

ACCAAGGGTAAATATCGGTGGTGATGACC

>CS24.38

TATTAGACTCCTAATTTAATATTCTTGTCTTATTATAATGCTGATAAGTCTTGTAAATAA

GGGTGAGAAGCACGAATAATTAGTTCATGAGATGTGTATAATTATTATCCTACACGACTT

ATCTTTGATATTTCACACAAGTCTTTCAATGTATAACAAAAACTTTTTAGATACATTTAG

ACTATAGAACTAACAAGTTATATTTTGAACC-AAAAAACAAAGAGAGAGAGAGAGGAACA

TAGAGAAAGGAAGATATGAGAGTTTTCTTTTTTTTTTT---CTAAAACAGAAAGAAACTC

ATTATATAATAAACAAATTACTTTGAGACAAATTAACGTATGTAATAAAACAAAATCAAA

GTGGTAATTAAAATTATTTAATGGTAAACAGTCTGATAGTTAAAATAAAAATGGAAATCA

CATAAATTTTGTAATTGGCCCATTAAAAACAACACTAAGCTTTTAATTTGATTTTGAAAT

TCAAAATAATTTTATTAAATCACAAAGTAAAAGGTTTACAAAGCCGATCATGACAGTGCA

TGTGGGAGGCAAATCGGCATATTTGCACTACAAAAGG-ACCCATGAAGTCTCTGAACATG

CACGCAACACTTTAATCTCTTATTAGTTACTTTGAAAGCTTATTTATATATATAGACACG

CGTAAAAACTTCTTAACCAAGATTTTTTT-ACGTGCTTCC-TTCGCGTTTAATTTGGACC

ATCAAACCGTGCTCAACAGATAAAGAAAAGGGTGCTTCTGATTCAAGATATTGGCC-GAA

AAACACAAGATAGATCCTT-CGATAGATTAAGCCACGCACGAAACGCGAATCCAAAGTGA

TGAAGAAGTGCAGATAGATATTCGTTCACCATATAGAGGAGAATATATCATTCCTACAAA

CAAAATTGATGATGTTTCTAGAATAGAAACGTTAGAATCGAATATTCATCTGTTCCGGGT

GGGGAAGGGCTACTGAAAAGCTGAACTTTTTTAAGAGTTCGAACTTCGAAAGTCAATCAA

TGATTCTTAATTGAGAGGGAAAAGCTATAAAGGACGAGAAGGGAAGGAAATGTTTATAGG

GAATGAATTTCTAATGCATGATGGGAATGACAGATATGAGGAATATATAAAGGTGATTCT

TATATACATTGACATGGATCGGAATCTATTAACTTAAAGTTATTGGGGTCGTGAAATTTA

TTTAATTTTTCTAATTCGTGTCACATGTCACGACAATTAGTGGGG-AACATTTATGTATA

TAGAAATTTCAGAAATTTCTAGCAGCGTGATAAATATAAAGATTTTGGCACAATAAGTTT

TTGGATCATAATGAGTTT-CTATTTAATCAA-GGCA-TGTAA-TCTATTTTATTATTTAG

-TAGGG-AAACTGAAAACTTAGGGTTGCTATTTGTAGCTCCCACCCTTCTTAGTTCTTAC

TCTTTTCAATATTTTTTAAAAGTTTTA-TATA-TACCT-AAATT-GCCCTCCTCCT----

----------------------------------------CACCCCATTGCTCTTCCGTT

GCTCCTCCATGTG-AGTTCTTGTCTTTTTTTTT-CTCTGGTAGCGTTTTGCTGCTCCTTT

TTTCACTCAAGTGTTGCCAATTAA-TTGACAAAAAATGGTTTCTGTTTCATATAGAAACT

ATGTTTTTGTTGTGTAGTCATACATTACGGAATCCAGTTT-CCATTAAATAAGTAAC--G

TGAAAAAAAA--TAAAAGGTGAAATATATATTGTTGGAAAAGAAGCTATGAGGTGCAAGA

ACCGATCACATGGAGAAGGCAATGAAAGACAAGGAGGAGCAATGGAAGA-----------

---------GAGAAAATGAGAAGATGGAAGGGATGTGAAAATGTTTGAAAAAAACGAGGT

GATCAGTTTTAAAATACGAATTTAGTATTTTCTTTTTAAGAAAATTCTTTCG-AAAGTCG

TGTTTTAAAACATGACTTTTATC-ATTTGAAGTCG---TGTTCTAAAACATGACTTA--T

TCATATCCTT-AATATTTTT---------AAAATTTATCCATTTGTAATATTTTTTAAAA

ATTGACCCATATATGTAAAATACCCGTCAAGATCTCTTTATTATTTTGAAAGCGAAAGCA

TATCACTTCAAACACAATGGAATCGAGGCTATTGACTAAGTATAAATAGAGAAGACTTCA

TATCGGGGTTCATAATTCATAACAAAGCAAACGAGTATATAAGAAAGCATAAGCCAAGTT

TTGAGTAAACTAGTGTGCACACTATCCCATGTCTAGTGGAAGTAGGGATCCTCTCGTTGT

TGGGGGAGTAATTGGGGATGTATTGGATCCTTTTGAATATTCTATTCCTATGAGGGTTAC

CTACAATAACAGAGATGTCAGCAATGGATGTGAATTCAAACCCTCACAAGTTGTCAACCA

ACCAAGGGTAAATATCGGTGGTGATGACC

>H06.06c

TATTAGACTCCTAATTTAATATTCTTGTTTTATTATAATGCTGATAAGTCTTGTAAATAA

GGGTGAGAAGCACGAATAATTAGTTCATGAGATGTGTATAATTATTATCCTACACGACTT

ATCTTTGATATTTCACACAAGCCTTTCAATGTATAACAAAAACTTTTTAGATACATTTAG

ACTATAGAACTAACAAGTTATATTTTGAACC-AAAAAACAAAGAGAGAGAGAGAGGAACA

TAGAGAAAAGAAGATATGAGAGTTTTTTTTTTTT-------CTAAAACAGAAAGAAACTC

ATTATATAATAAACAAATTACTTTGAGACAAATTAACGTATGTAATAAAACAAAATCAAA

GTGGTAATTAAAATTATTTAATGGTAAACAGTCTAATAGTTAAAATAAAAATGGAAATCA

CATAAATTTTGTAATTGGCCCATTAAAAACAACACTAAGCTTTTAATTTGATTTTGAAAT

TCAAAATAATTTTATTAAATCACAAAGTAAAAGGTTTACGAAGCCGATCATGACAGTGCA

TGTGGGAGGCAAATCGGCATATTTGCACTACAAAAGG-ACCCATGAAGTCCCTGAACATG

CACGCAACACTTTAATCTCTTATTAGTTACTTTGAAAGCTTATTTATATATATAGACACG

CGTAAAAACTTCTTAACCAAGATTTTTTT-ACGTGCTTCC-TTCGCGTTTAATTTGGACC

ATCAAACCGTGCTCAACAGATAAAGAAAAGGGTGCTTTTGATTCAAGATATTGGCC-GAA

AAACACAAGATAGATCCTT-CGATAGATTAAGCCACGCATGAAACGCGAATCCAAAGTGA

TGAAGAAGTGCAGATAGATATTCGTTCACCATATAGAGGAGAATATATCATTCCTACAAA

CAAAATTGATGATGTTTCTAGAATAGAAACGTTAGAATCGAATATTCATCTGTTCCGGGT

GGGGAAGGGCTACTGAAAAGCTGAACTTTTTTAAGAGTTCGAACTTCGAAAGTCAATCAA

TGATTCTTAATTGAGAGGGAAAAGCTATAAAGGACGAGAAGGGAAGGAAATGTTTATAGG

GAATGAGTTTCTAATGCATGATGGGAATGACAGATATGAGGAATATATAAAGGTGATTCT

TATATACATTGACATGGATCGGAATCTATTAACTTAAAGTTATTGGGGTCGTGAAATTTA

TTTAATTTTTCTAATTCGTGTCACATGTCACGACAATTAGTGGGG-AACATTTATGTATA

TAGAAATTTCAGAAATTTCTAGCAGCGTGATAAATATAAAGATTTTGGCACAATAAGTTT

TTGGATCATAATGAGTTT-CTATTTAATCAA-GGCA-TGTAA-TCTATTTTATTATTTAG

-TAGGG-AAACTGAAAACTTAGGGTTGCTATTTGTAGCTCCCACCCTTCTTAGTTCTTAC

TCTTTTCAATATTTTTTAAAAGTTTTA-TATAATACCT-AAATT-GCCCTCCTCCT----

----------------------------------------CACCCCATTGCTCTTCCGTT

GCTCCTCCATGTG-AGTTCTTGTCTTTTTTTTT-CTCTGGTAGCGTTTTGCTGCTCCTTT

TTTCACTCAAGTGTTGCCAATTAA-TTGACAAAAAATGGTTTCTGTTTCATATAGAAACT

ATGTTTTTGTTGTGTAGTCATACATTACGGAATCTAGTTT-CCATTAAATAAGTAAC--G

TGAAAAAAAA--TAAAAGGTGAAATATATATTGTTGGAAAAGAAGCTATGAGGTGCAAGA

ACCGATCACATGGAGAAGGCAATGAAAGACAAGGAGGAGCAATGGAAGA-----------

---------GAGAAAATGAGAAGATGGAAGGGATGTGAAAATGTTTGAAAAAAACGAGGT

GATCAGTTTTAAAATACGAATTTAGTATTTTCTTTTTAAGAAAATTCTTTCG-AAAGTCG

TGTTTTAAAACATGACTTTTATT-ATTTGAAGTCG---TGTTCTAAAACATGACTTA--T

TCATATCCTT-AATATTTTT---------AAAATTTATCCATTTGTAATATTTTTTAAAA

ATTGACCCATATATGTAAAATACCCGTCAAGATCTCTTTATTATTTTGAAAGCGAAAGCA

TATCACTTCAAACACAATGGAATCGAGGCTATTGACTAAGTATAAATAGGGAAGACTTCA

TATCGGGGTTCATAATTCATAACAAAGCAAACGAGTATATAAGAAAGCATAAGCCAAATT

TTGAGTAAACTAGTGTGCACACTATCCCATGCCTAGTGGAAGTAGGGATCCTCTCGTTGT

TGGGGGAGTAATTGGGGATGTATTGGATCCTTTTGAATATTCTATTCCTATGAGGGTTAC

CTACAATAACAGAGATGTCAGCAATGGATGTGAATTCAAACCCTCACAAGTTGTCAACCA

ACCAAGGGTAAATATCGGTGGTGATGACC

>H15.G6

TATTAGACTCCTAATTTAATATTCTTGTTTTATTATAATGCTGATGAGTCTTGTAAATAA

GGGTGAGAAGCACGAATAATTAGTTCATGAGATGTGTATAATTATTATCCTACACGACTT

ATCTTTGATATTTCACACAAGTCTTTCAATGTATAACAAAAACTTTTTAGATACATTTAG

ACTATAGAACTAACAAGTTATATTTTGAACC-AAAAAACAAAGAGAGAGAGAGAGGAACA

TAGAGAAAAGAAGATATGAGAGTTTTTTTTTTTTTTTTT--CTAAAACAGAAAGAAACTC

ATTATATAATAAACAAATTACTTTGAGACAAATTAACGTATGTAATAAAACAAAATCAAA

GTGGTAATTAAAATTATTTAATGGTAAACAGTCTAATAGTTAAAATAAAAATGGAAATCA

CATAAATTTTGTAATTGGCCCATTAAAAACAACACTAAGCTTTTAATTTGATTTTGAAAT

TCAAAATAATTTTATTAAATCACAAAGTAAAAGGTTTACAAAGCCGATCATGACAGTGCA

TGTGGGAGGCAAATCGGCATATTTGCACTACAAAAGG-ACCCATGAAGTCTCTGAACATG

CACGCAACACTTTAATCTCTTATTAGTTACTTTGAAAGCTTATTTATATATATAGACACG

CGTAAAAACTTCTTAACCAAGATTTTTTT-ACGTGCTTCC-TTCGCGTTTAATTTGGACC

ATCAAACCGTGCTCAACAGATAAAGAAAAGGGTGCTTTTGATTCAAGATATTGGCC-GAA

AAACACAAGATAGATCCTT-CGATAGATTAAGCCACGCATGAAACGCGAATCCAAAGTGA

TGAAGAAGTGCAGATAGATATTCGTTCACCATATAGAGGAGAATATATCATTCCTACAAA

CAAAATTGATGATGTTTCTAGAATAGAAACGTTAGAATCGAATATTCATCTGTTCCGGGT

GGGGAAGGGCTACTGAAAAGCTGAACTTTTTTAAGAGTTCGAACTTCGAAAGTCAATCAA

TGATTCTTAATTGAGAGGGAAAAGCTATAAAGGACGAGAAGGGAAGGAAATGTTTATAGG

GAATGAATTTCTAATGCATGATGGGAATGACAGATATGAGGAATATATAAAGGTGATTCT

TATATACATTGACATGGATCGGAATCTATTAACTTAAAGTTATTGGGGTCGTGAAATTTA

TTTAATTTTTCTAATTCGTGTCACATGTCACGACAATTAGTGGGG-AACATTTATGTATA

TAGAAATTTCAGAAATTTCTAGCAGCGTGATAAATATAAAGATTTTGGCACAATAAGTTT

TTGGATCATAATGAGTTT-CTATTTAATCAA-GGCA-TGTAA-TCTATTTTATTATTTAG

-TAGGG-AAACTGAAAACTTAGGGTTGCTATTTGTAGCTCCCACCCTTCTTAGTTCTTAC

TCTTTTCAATATTTTTTAAA-GTTTTA-TATAATACCT-AAATT-GCCCTCCTCCT----

----------------------------------------CACCCCATTGCTCTTCCGTT

GCTCCTCCATGTG-AGTTCTTGTCTTTTTTTTT-CTCTGGTAGCGTTTTGCTGCTCCTTT

TTTCACTCAAGTGTTGCCAATTAA-TTGACAAAAAATGGTTTCTGTTTCATATAGAAACT

ATGTTTTTGTTGTGTAGTCATACATTACGGAATCTAGTTTTCCATTAAATAAGTAAC--G

TGAAAAAAAAA-TAAAAGGTGAAATATATATTGTTGGAAAAGAAGCTATGAGGTGCAAGA

ACCGATCACATGGAGAAGGCAATGAAAGACAAGGAGGAGCAATGGAAGA-----------

---------GAGAAAATGAGAAGATGGAAGGGATGTGAAAATGTTTGAAAAAAACGAGGT

GATCAGTTTTAAAATACGAATTTAGTATTTTCTTTTTAAGAAAATTCTTTCG-AAAGTCG

TGTTTTAAAACATGACTTTTATT-ATTTGAAGTCG---TGTTCTAAAACATGACTTA--T

TCATATCCTT-AATATTTTT---------AAAATTTATCCATTTGTAATATTTTTTAAAA

ATTGACCCATATATGTAAAATACCCGTCAAGATCTCTTTATTATTTTGAAAGCGAAAGCA

TATCACTTCAAACACAATGGAATCGAGGCTATTGACTAAGTATAAATAGAGAAGACTTCA

TATCGGGGTTCATAATTCATAACAAAGCAAACGAGTATATAAGAAAGCATAAGCCAAATT

TTGAGTAAACTAGTGTGCACACTATCCCATGCCTAGTGGAAGTAGGGATCCTCTCGTTGT

TGGGGGAGTAATTGGGGGTGTATTGGATCCTTTTGAATATTCTATTCCTATGAGGGTTAC

CTACAATAACAGAGATGTCAGCAATGGATGTGAATTCAAACCCTCACAAGTTGTCAACCA

ACCAAGGGTAAATATCGGTGGTGATGACC

>CS25.20

TATTAGACTCCTAATTTAATATTCTTGTTTTATTATAATGCTGATAAGTCTTGTAAATAA

GGGTGAGAAGCACGAATAATTAGTTCATGAGATGTGTATAATTATTATCCTACACGACTT

ATCTTTGATATTTCACACAAGTCTTTCAATGTATAACAAAAACTTTTTAGATACATTTAG

ACTATAGAACTAACAAGTTATATTTTGAACC-AAAAAACAAAGAGAGAGAGAGAGGAACA

TAGAGAAAAGAAGATATGAGAGTTTTTTTTTTTTTTT----CTAAAACAGAAAGAAACTC

ATTATATAATAAACAAATTACTTTGAGACAAATTAACGTATGTAATAAAACAAAATCAAA

GTGGTAATTAAAATTATTTAATGGTAAACAGTCTAATAGTTAAAATAAAAATGGAAATCA

CATAAATTTTGTAATTGGCCCATTAAAAACAACACTAAGCTTTTAATTTGATTTTGAAAT

TCAAAATAATTTTATTAAATCACAAAGTAAAAGGTTTACAAAGCCGATCATGACAGTGCA

TGTGGGAGGCAGATCGGCATATTTGCACTACAAAAGG-ACCCATGAAGTCTCTGAACATG

CACGCAACACTTTAATCTCTTATTAGTTACTTTGAAAGCTTATTTATATATATAGACACG

CGTAAAAACTTCTTAACCAAGATTTTTTT-ACGTGCTTCC-TTCGCGTTTAATTTGGACC

ATCAAACCGTGCTCAACAGATAAAGAAAAGGGTGCTTTTGATTCAAGATATTGGCC-GAA

AAACACAAGATAGATCCTT-CGATAGATTAAGCCACGCATGAAACGCGAATCCAAAGTGA

TGAAGAAGTGCAGATAGATATTCGTTCACCATATAGAGGAGAATATATCATTCCTACAAA

CAAAATTGATGATGTTTCTAGAATAGAAACGTTAGAATCGAATATTCATCTGTTCCGGGT

GGGGAAGGGCTACTGAAAAGCTGAACTTTTTTAAGAGTTCGAACTTCGAAAGTCCATCAA

TGATTCTTAATTGAGAGGGAAAAGCTATAAAGGACGAGAAGGGAAGGAAATGTTTATAGG

GAATGAATTTCTAATGCATGATGGGAATGACAGATATGAGGAATATATAAAGGTGATTCT

TATATACATTGACATGGATCGGAATCTATTAACTTAAAGTTATTGGGGTCGTGAAATTTA

TTTAATTTTTCTAATTCGTGTCACATGTCACGACAATTAGTGGGG-AACATTTATGTATA

TAGAAATTTCAGAAATTTCTAGCAGCGTGATAAATATAAAGATTTTGGCACAATAAGTTT

TTGGATCATAATGAGTTT-CTATTTAATCAA-GGCA-TGTAA-TCTATTTTATTATTTAG

-TAGGG-AAACTGAAAACTTAGGGTTGCTATTTGTAGCTCCCACCCTTCTTAGTTCTTAC

TCTTTTCAATATTTTTTAAAAGTTTTA-TATAATACCT-AAATT-GCCCTCCTCCT----

----------------------------------------CACCCCATTGCTCTTCCGTT

GCTCCTCCATGTG-AGTTCTTGTCTTTTTTTTT-CTCTGGTAGCGTTTTGCTGCTCCTTT

TTTCACTCAAGTGTTGCCAATTAA-TTGACAAAAAATGGTTTCTGTTTCATATAGAAACT

ATGTTTTTGTTGTGTAGTCATACATTACGGAATCTAGTTT-CCATTAAATAAGTAAC--G

TGAAAAAAAA--TAAAAGGTGAAATATATATTGTTGGAAAAGAAGCTATGAGGTGTAAGA

ACCGATCACATGGAGAAGGCAATGAAAGACAAGGAGGAGCAATGGAAGA-----------

---------GAGAAAATGAGAAGATGGAAGGGATGTGAAAATGTTTGAAAAAAACGAGGT

GATCAGTTTTAAAATACGAATTTAGTATTTTCTTTTTAAGAAAATTCTTTCG-AAAGTCG

TGTTTTAAAACATGACTTTTATT-ATTTGAAGTCG---TGTTCTAAAACATGACTTA--T

TCATATCCTT-AATATTTTT---------AAAATTTATCCATTTGTAATATTTTTTAAAA

ATTGACCCATATATGTAAAATACCCGTCAAGATCTCTTTATTATTTTGAAAGCGAAAGCA

TATCACTTCAAACACAATGGAATCGAGGCTATTGACTAAGTATAAATAGAGAAGACTTCA

TATCGGGGTTCATAATTCATAACAAAGCAAACGAGTATATAAGAAAGCATAAGCCAAATT

TTGAGTAAACTAGTGTGCACACTATCCCATGCCTAGTGGAAGTAGGGATCCTCTCGTTGT

TGGGGGAGTAATTGGGGATGTATTGGATCCTTTTGAATATTCTATTCCTATGAGGGTTAC

CTACAATAACAGAGATGTCAGCAATGGATGTGAATTCAAACCCTCACAAGTTGTCAACCA

ACCAAGGGTAAATATCGGTGGTGATGACC

>CS38.72

TATTAGACTCCTAATTTAATATTCTTGTTTTATTATAATGCTGATAAGTCTTGTAAATAA

GGGTGAGAAGCACGAATAATTAGTTCATGAGATGTGTATAATTATTATCCTACACGACTT

ATCTTTGATATTTCACACAAGTCTTTCAATGTATAACAAAAACTTTTTAGATACATTTAG

ACTATAGAACTAACAAGTTATATTTTGAACC-AAAAAACAAAGAGAGAGAGAGAGGAACA

TAGAGAAAAGAAGATATGAGAGTTTTTTTTTTTTTTTT---CTAAAACAGAAAGAAACTC

ATTATATAATAAACAAATTACTTTGAGACAAATTAACGTATGTAATAAAACAAAATCAAA

GTGGTAATTAAAATTATTTAATGGTAAACAGTCTAATAGTTAAAATAAAAATGGAAATCA

CATAAATTTTGTAATTGGCCCATTAAAAACAACACTAAGCTTTTAATTTGATTTTGAAAT

TCAAAATAATTTTATTAAATCACAAAGTAAAAGGTTTACAAAGCCGATCATAACAGTGCA

TGTGGGAGGCAAATCGGCATATTTGCACTACAAAAGG-ACCCATGAAGTCTCTGAACATG

CACGCAACACTTTAATCTCTTATTAGTTACTTTGAAAGCTTATTTATATATATAGACACG

CGTAAAAACTTCTTAACCAAGATTTTTTT-ACGTGCTTCC-TTCGCGTTTAATTTGGACC

ATCAAACCGTGCTCAACAGATAAAGAAAAGGGTGCTTTTGATTCAAGATATTGGCC-GAA

AAACACAAGATAGATCCTT-CGATAGATTAAGCCACGCATGAAACGCGAATCCAAAGTGA

TGAAGAAGTGCAGATAGATATTCGTTCACCATATAGAGGAGAATATATCATTCCTACAAA

CAAAATTGATGATGTTTCTAGAATAGAAACGTTAGAATCGAATATTCATCTGTTCCGGGT

GGGGAAGGGCTACTGAAAAGCTGAACTTTTTTAAGAGTTCGAACTTCGAAAGTCAATCAA

TGATTCTTAATTGAGAGGGAAAAGCTATAAAGGACGAGAAGGGAAGGAAATGTTTATAGG

GAATGAATTTCTAATGCATGATGGGAATGACAGATATGAGGAATATATAAAGGTGATTCT

TATATACATTGACATGGATCGGAATCTATTAACTTAAAGTTATTGGGGTCGTGAAATTTA

TTTAATTTTTCTAATTCGTGTCACATGTCACGACAATTAGTGGGG-AACATTTATGTATA

TAGAAATTTCAGAAATTTCTAGCAGCGTGATAAATATAAAGATTTTGGCACAATAAGTTT

TTGGATCATAATGAGTTT-CTATTTAATCAA-GGCA-TGTAA-TCTATTTTATTATTTAG

-TAGGG-AAACTGAAAACTTAGGGTTGCTATTTGTAGCTCCCGCCCTTCTTAGTTCTTAC

TCTTTTCAATATTTTTTAAAAGTTTTA-TATAATACCT-AAATT-GCCCTCCTCCT----

----------------------------------------CACCCCATTGCTCTTCCGTT

GCTC-TCCATGTG-AGTTCTTGTCTTTTTTTTT-CTCTGGTAGCGTTTTGCTGCTCCTTT

TTTCACTCAAGTGTTGCCAATTAA-TTGACAAAAAATGGTTTCTGTTTCATATAGAAACT

ATGTTTTTGTTGTGTAGTCATACATTACGGAATCTAGTTT-CCATTAAATAAGTAAC--G

TGAAAAAAAA--TAAAAGGTGAAATATATATTGTTGGAAAAGAAGCTATGAGGTGCAAGA

ACCGATCACATGGAGAAGGCAATGAAAGACAAGGAGGAGCAATGGAAGA-----------

---------GAGAAAATGAGAAGATGGAAGGGATGTGAAAATGTTTGAAAAAAACGAGGT

GATCAGTTTTAAAATACGAATTTAGTATTTTCTTTTTAAGAAAATTCTTTCG-AAAGTCG

TGTTTTAAAACATGACTTTTATT-ATTTGAAGTCG---TGTTCTAAAACATGACTTA--T

TCATATCCTT-AATATTTTT---------AAAATTTATCCATTTGTAATATTTTTTAAAA

ATTGACCCATATATGTAAAATACCCGTCAAGATCTCTTTATTATTTTGAAAGCGAAAGCA

TATCACTTCAAACACAATGGAATCGAGGCTATTGACTAAGTATAAATAGAGAAGACTTCA

TATCGGGGTTCATAATTCATAACAAAGCAAACGAGTATATAAGAAAGCATAAGCCAAATT

TTGAGTAAACTAGTGTGCACACTATCCCATGCCTAGTGGAAGTAGGGATCCTCTCGTTGT

TGGGGGAGTAATTGGGGATGTATTGGATCCTTTTGAATATTCTATTCCTATGAGGGTTAC

CTACAATAACAGAGATGTCAGCAATGGATGTGAATTCAAACCCTCACAAGTTGTCAACCA

ACCAAGGGTAAATATCGGTGGTGATGACC

>H11.B8

TATTAGACTCCTAATTTAATATTCTTGTTTTATTATAATGCTGATAAGTCTTGTAAATAA

GGGTGAGAAGCACGAATAATTAGTTCATGAGATGTGTATAATTATTATCCTACACGACTT

ATCTTTGATATTTCACACAAGTCTTTCAATGTATAACAAAAACTTTTTAGATACATTTAG

ACTATAGAACTAACAAGTTATATTTTGAACC-AAAAAACAAAGAGAGAGAGAGAGGAACA

TAGAGAAAAGAAGATATGAGAGTTTTTTTTTTTTTTT----CTAAAGCAGAAAGAAACTC

ATTATATAATAAACAAATTACTTTGAGACAAATTAACGTATGTAATAAAACAAAATCAAA

GTGGTAATTAAAATTATTTAATGGTAAACAGTCTAATAGTTAAAATAAAAATGGAAATCA

CATAAATTTTGTAATTGGCCCATTAAAAACAACACTAAGCTTTTAATTTGATTTTGAAAT

TCAAAATAATTTTATTAAATCACAAAGTAAAAGGTTTACAAAGCCGATCATGACAGTGCA

TGTGGGAGGCAAATCGGCATATTTGCACTACAAAAGG-ACCCATGAAGTCTCTGAACATG

CACGCAACACTTTAATCTCTTATTAGTTACTTTGAAAGCTTATTTATATATATAGACACG

CGTAAAAACTTCTTAACCAAGATTTTTTT-ACGTGCTTCC-TTCGCGTTTAATTTGGACC

ATCAAACCGTGCTCAACAGATAAAGAAAAGGGTGCTTTTGATTCAAGATATTGGCC-GAA

AAACACAAGATAGATCCTT-CGATAGATTAAGCCACGCATGAAACGCGAATCCAAAGTGA

TGAAGAAGTGCAGATAGATATTCGTTCACCATATAGAGGAGAATATATCATTCCTACAAA

CAAAATTGATGATGTTTCTAGAATAGAAACGTTAGAATCGAATATTCATCTGTTCCGGGT

GGGGAAGGGCTACTGAAAAGCTGAACTTTTTTAAGAGTTCGAACTTCGAAAGTCAATCAA

TGATTCTTAATTGAGAGGGAAAAGCTATAAAGGACGAGAAGGGAAGGAAATGTTTATAGG

GAATGAATTTCTAATGCATGATGGGAATGACAGATATGAGGAATATATAAAGGTGATTCT

TATATACATTGACATGGATCGGAATCTATTAACTTAAAGTTATTGGGGTCGTGAAATTTA

TTTAATTTTTCTAATTCGTGTCACATGTCACGACAATTAGTGGGG-AACATTTATGTATA

TAGAAATTTCAGAAATTTCTAGCAGCGTGATAAATATAAAGATTTTGGCACAATAAGTTT

TTGGATCATAATGAGTTT-CTATTTAATCAA-GGCA-TGTAA-TCTATTTTATTATTTAG

-TAGGGGAAACTGAAAACTTAGGGTTGCTATTTGTAGCTCCCACCCTTCTTAGTTCTTAC

TCTTTTCAATATTTTTTAAAAGTTTTA-TATAATACCT-AAATT-GCCCTCCTCCT----

----------------------------------------CACCCCATTGCTCTTCCGTT

GCTCCTCCATGTG-AGTTCTTGTCTTTTTTTTT-CTCTGGTAGCGTTTTGCTGCTCCTTT

TTTCACTCAAGTGTTGCCAATTAA-TTGACAAAAAATGGTTTCTGTTTCATATAGAAACT

ATGTTTTTGTTGTGTAGTCATACATTACGGAATCTAGTTT-CCATTAAATAAGTAAC--G

TGAAAAAAAA--TAAAAGGTGAAATATATATTGTTGGAAAAGAAGCTATGAGGTGCAAGA

ACCGATCACATGGAGAAGGCAATGAAAGACAAGGAGGAGCAATGGAAGA-----------

---------GAGAAAATGAGAAGATGGAAGGGATGTGAAAATGTTTGAAAAAAACGAGGT

GATCAGTTTTAAAATACGAATTTAGTATTTTCTTTTTAAGAAAATTCTTTCG-AAAGTCG

TGTTTTAAAACATGACTTTTATT-ATTTGAAGTCG---TGTTCTAAAACATGACTTA--T

TCATATCCTT-AATATTTTT---------AAAATTTATCCATTTGTAATATTTTTTAAAA

ATTGACCCATATATGTAAAATACCCGTCAAGATCTCTTTATTATTTTGAAAGCGAAAGCA

TATCACTTCAAACACAATGGAATCGAGGCTATTGACTAAGTATAAATAGAGAAGACTTCA

TATCGGGGTTCATAATTCATAACAAGGCAAACGAGTATATAAGAAAGCATAAGCCAAATT

TTGAGTAAACTAGTGTGCACACTATCCCATGCCTAGTGGAAGTAGGGATCCTCTCGTTGT

TGGGGGAGTAATTGGGGATGTATTGGATCCTTTTGAATATTCTATTCCTATGAGGGTTAC

CTACAATAACAGAGATGTCAGCAATGGATGTGAATTCAAACCCTCACAAGTTGTCAACCA

ACCAAGGGTAAATATCGGTGGTGATGACC

>H14.11

TATTAGACTCCTAATTTAATATTCTTGTTTTATTATAATGCTGATAAGTCTTGTAAATAA

GGGTGAGAAGCACGAATAATTAGTTCATGAGATGTGTATAATTATTATCCTACACGACTT

ATCTTTGATATTTCACACAAGTCTTTCAATGTATAACAAAAACTTTTTAGATACATTTAG

ACTATAGAACTAACAAGTTATATTTTGAACC-AAAAAACAAAGAGAGAGAGAGAGGAACA

TAGAGAAAAGAAGATATGAGAGTTTTTTTTTTTTTT-----CTAAAACAGAAAGAAACTC

ATTATATAATAAACAAATTACTTTGAGACAAATTAACGTATGTAATAAAACAAAATCAAA

GTGGTAATTAAAATTATTTAATGGTAAACAGTCTAATAGTTAAAATAAAAATGGAAATCA

CATAAATTTTGTAATTGGCCCATTAAAAACAACACTAAGCTTTTAATTTGATTTTGAAAT

TCAAAATAATTTTATTAAATCACAAAGTAAAAGGTTTACAAAGCCGATCATGACAGTGCA

TGTGGGAGGCAAATCGGCATATTTGCACTACAAAAGG-ACCCATGAAGTCTCTGAACATG

CACGCAACACTTTAATCTCTTATTAGTTACTTTGAAAGCTTATTTATATATATAGACACG

CGTAAAAACTTCTTAACCAAGATTTTTTT-ACGTGCTTCC-TTCGCGTTTAATTTGGACC

ATCAAACCGTGCTCAACAGATAAAGAAAAGGGTGCTTTTGATTCAAGATATTGGCC-GAA

AAACACAAGATAGATCCTT-CGATAGATTAAGCCACGCATGAAACGCGAATCCAAAGTGA

TGAAGAAGTGCAGATAGATATTCGTTCACCATATAGAGGAGAATATATCATTCCTACAAA

CAAAATTGATGATGTTTCTAGAATAGAAACGTTAGAATCGAATATTCATCTGTTCCGGGT

GGGGAAGGGCTACTGAAAAGCTGAACTTTTTTAAGAGTTCGAACTTCGAAAGTCAATCAA

TGATTCTTAATTGAGAGGGAAAAGCTATAAAGGACGAGAAGGGAAGGAAATGTTTATAGG

GAATGAATTTCTAATGCATGATGGGAATGACAGATATGAGGAATATATAAAGGTGATTCT

TATATACATTGACATGGATCGGAATCTATTAACTTAAAGTTATTGGGGTCGTGAAATTTA

TTTAATTTTTCTAATTCGTGTCACATGTCACGACAATTAGTGGGG-AACATTTATGTATA

TAGAAATTTCAGAAATTTCTAGCAGCGTGATAAATATAAAGATTTTGGCACAATAAGTTT

TTGGATCATAATGAGTTT-CTATTTAATCAA-GGCA-TGTAA-TCTATTTTATTGTTTAG

-TAGGG-AAACTGAAAACTTAGGGTTGCTATTTGTAGCTCCCACCCTTCTTAGTTCTTAC

TCTTTTCAATATTTTTTAAAGGTTTTA-TATAATACCT-AAATT-GCCCTCCTCCT----

----------------------------------------CACCCCATTGCTCTTCCGTT

GCTCCTCCATGTG-AGTTCTGTCTTTTTTTTTT-CTCTGGTAGCGTTTTGCTGCTCCTTT

TTTCACTCAAGTGTTGCCAATTAA-TTGACAAAAAATGGTTTCTGTTTCATATAGAAACT

ATGTTTTTGTTGTGTAGTCATACATTACGGAATCTAGTTT-CCATTAAATAAGTAAC--G

TGAAAAAAAA--TAAAAGGTGAAATATATATTGTTGGAAAAGAAGCTATGAGGTGCAAGA

ACCGATCACATGGAGAAGGCAATGAAAGACAAGGAGGAGCAATGGAAGA-----------

---------GAGAAAATGAGAAGATGGAAGGGATGTGAAAATGTTTGAAAAAAACGAGGT

GATCAGTTTTAAAATACGAATTTAGTATTTTCTTTTTAAGAAAATTCTTTCG-AAAGTCG

TGTTTTAAAACATGACTTTTATT-ATTTGAAGTCG---TGTTCTAAAACATGACTTA--T

TCATATCCTT-AATATTTTT---------AAAATTTATCCATTTGTAATATTTTTTAAAA

ATTGACCCATATATGTAAAATACCCGTCAAGATCTCTTTATTATTTTGAAAGCGAAAGCA

TATCACTTCAAACACAATGGAATCGAGGCTATTGACTAAGTATAAATAGAGAAGACTTCA

TATCGGGGTTCATAATTCATAACAAAGCAAACGAGTATATAAGAAAGCATAAGCCAAATT

TTGAGTAAACTAGTGTGCACACTATCCCATGCCTAGTGGAAGTAGGGATCCTCTCGTTGT

TGGGGGAGTAATTGGGGATGTATTGGATCCTTTTGAATATTCTATTCCTATGAGGGTTAC

CTACAATAACAGAGATGTCAGCAATGGATGTGAATTCAAACCCTCACAAGTTGTCAACCA

ACCAAGGGTAAATATCGGTGGTGATGACC

>H13.E4

TATTAGGCTCCTAATTTAATATTCTTGTTTTATTATAATGCTGATAAGTCTTGTAAATAA

GGGTGAGAAGCACGAATAATTAGTTCATGAGATGTGTATAATTATTATCCTACACGACTT

ATCTTTGATATTTCACACAAGTCTTTCAATGTATAACAAAAACTTTTTAGATACATTTAG

ACTATAGAACTAACAAGTTATATTTTGAACC-AAAAAACAAAGAGAGAGAGAGAGGAACA

TAGAGAAAAGAAGATATGAGAGTTTTTTTTTTTTTTTT---CTAAAACAGAAAGAAACTC

ATTATATAATAAACAAATTACTTTGAGACAAATTAACGTATGTAATAAAACAAAATCAAA

GTGGTAATTAAAATTATTTAATGGTAAACAGTCTAATAGTTAAAATAAAAATGGAAATCA

CATAAATTTTGTAATTGGCCCATTAAAAACAACACTAAGCTTTTAATTTGATTTTGAAAT

TCAAAATAATTTTATTAAATCACAAAGTAAAAGGTTTACAAAGCCGATCATGACAGTGCA

TGTGGGAGGCAAATCGGCATATTTGCACTACAAAAGG-ACCCATGAAGTCTCTGAACATG

CACGCAACACTTTAATCTCTTATTAGTTACTTTGAAAGCTTATTTATATATATAGACACG

CGTAAAAACTTCTTAACCAAGATTTTTTT-ACGTGCTTCC-TTCGCGTTTAATTTGGACC

ATCAAACCGTGCTCAACAGATAAAGAAAAGGGTGCTTTTGATTCAAGATATTGGCC-GAA

AAACACAAGATAGATCCTT-CGATAGATTAAGCCACGCATGAAACGCGAATCCAAAGTGA

TGAAGAAGTGCAGATAGATATTCGTTCACCATATAGAGGAGAATATATCATTCCTACAAA

CAAAATTGATGATGTTTCTAGAATAGAAACGTTAGAATCGAATATTCATCTGTTCCGGGT

GGGGAAGGGCTACTGAAAAGCTGAACTTTTTTAAGAGTTCGAACTTCGAAAGTCAATCAA

TGATTCTTAATTGAGAGGGAAAAGCTATAAAGGACGAGAAGGGAAGGAAATGTTTATAGG

GAATGAATTTCTAATGCATGATGGGAATGACAGATATGAGGAATATATAAAGGTGATTCT

TATATACATTGACATGGATCGGAATCTATTAACTTAAAGTTATTGGGGTCGTGAAATTTA

TTTAATTTTTCTAATTCGTGTCACATGTCACGACAATTAGTGGGG-AACATTTATGTATA

TAGAAATTTCAGAAATTTCTAGCAGCGTGATAAATATAAAGATTTTGGCACAATAAGTTT

TTGGATCATAATGAGTTT-CTATTTAATCAA-GGCA-TGTAA-TCTATTTTATTATTTAG

-TAGGG-AAACTGAAAACTTAGGGTTGCTATTTGTAGCTCCCACCCTTCTTAGTTCTTAC

TCTTTTCAATATTTTTTAAAAGTTTTA-TATAATACCT-AAATT-GCCCTCCTCCT----

----------------------------------------CACCCCATTGCTCTTCCGTT

GCTCCTCCATGTG-AGTTCTTGTCTTTTTTTTT-CTCTGGTAGCGTTTTGCTGCTCCTTT

TTTCACTCAAGTGTTGCCAATTAA-TTGACAAAAAATGGTTTCTGTTTCATATAGAAACT

ATGTTTTTGTTGTGTAGTCATACATTACGGAATCTAGTTT-CCATTAAATAAGTAAC--G

TGAAAAAAAA--TAAAAGGTGAAATATATATTGTTGGAAAAGAAGCTATGAGGTGCAAGA

ACCGATCACATGGAGAAGGCAATGAAAGACAAGGAGGAGCAATGGAAGA-----------

---------GAGAAAATGAGAAGATGGAAGGGATGTGAAAATGTTTGAAAAAAACGAGGT

GATCAGTTTTAAAATACGAATTTAGTATTTTCTTTTTAAGAAAATTCTTTCG-AAAGTCG

TGTTTTAAAACATGACTTTTATT-ATTTGAAGTCG---TGTTCTAAAACATGACTTA--T

TCATATCCTT-AATATTTTT---------AAAATTTATCCATTTGTAATATTTTTTAAAA

ATTGACCCATATATGTAAAATACCCGTCAAGATCTCTTTATTATTTTGAAAGCGAAAGCA

TATCACTTCAAACACAATGGAATCGAGGCTATTGACTAAGTATAAATAGAGAAGACTTCA

TATCGGGGTTCATAATTCATAACAAAGCAAACGAGTATATAAGAAAGCATAAGCCAAATT

TTGAGTAAACTAGTGTGCACACTATCCCATGCCTAGTGGAAGTAGGGATCCTCTCGTTGT

TGGGGGAGTAATTGGGGATGTATTGGATCCTTTTGAATATTCTATTCCTATGAGGGTTAC

CTACAATAACAGAGATGTCAGCAATGGATGTGAATTCAAACCCTCACAAGTTGTCAACCA

ACCAAGGGTAAATATCGGTGGTGATGACC

>CS36.56

TATTAGACTCCTAATTTAATATTCTTGTTTTATTATAATGCTGATAAGTCTTGTAAATAA

GGGTGAGAAGCACGAATAATTAGTTCATGAGATGTGTATAATTATTATCCTACACGACTT

ATCTTTGATATTTCACACAAGTCTTTCAATGTATAACAAAAACTTTTTAGATACATTTAG

ACTATAGAACTAACAAGTTATATTTTGAACC-AAAAAACAAAGAGAGAGAGAGAGGAACA

TAGAGAAAAGAAGATATGAGAGTTTTTTTTTTTTT------CTAAAACAGAAAGAAACTC

ATTATATAATAAACAAATTACTTTGAGACAAATTAACGTATGTAATAAAACAAAATCAAA

GTGGTAATTAAAATTATTTAATGGTAAACAGTCTAATAGTTAAAATAAAAATGGAAATCA

CATAAATTTTGTAATTGGCCCATTAAAAACAACACTAAGCTTTTAATTTGATTTTGAAAT

TCAAAATAATTTTATTAAATCACAAAGTAAAAGGTTTACAAAGCCGATCATGACAGTGCA

TGTGGGAGGCAAATCGGCATATTTGCACTACAAAAGG-ACCCATGAAGTCTCTGAACATG

CACGCAACACTTTAATCTCTTATTAGTTACTTTGAAAGCTTATTTATATATATAGACACG

CGTAAAAACTTCTTAACCAAGATTTTTTT-ACGTGCTTCC-TTCGCGTTTAATTTGGACC

ATCAAACCGTGCTCAACAGATAAAGAAAAGGGTGCTTTTGATTCAAGATATTGGCC-GAA

AAACACAAGATAGATCCTT-CGATAGATTAAGCCACGCATGAAACGCGAATCCAAAGTGA

TGAAGAAGTGCAGATAGATATTCGTTCACCATATAGAGGAGAATATATCATTCCTACAAA

CAAAATTGATGATGTTTCTAGAATAGAAACGTTAGAATCGAATATTCATCTGTTCCGGGT

GGGGAAGGGCTACTGAAAAGCTGAACTTTTTTAAGAGTTCGAACTTCGAAAGTCAATCAA

TGATTCTTAATTGAGAGGGAAAAGCTATAAAGGACGAGAAGGGAAGGAAATGTTTATAGG

GAATGAATTTCTAATGCATGATGGGAATGACAGATATGAGGAATATATAAAGGTGATTCT

TATATACATTGACATGGATCGGAATCTATTAACTTAAAGTTATTGGGGTCGTGAAATTTA

TTTAATTTTTCTAATTCGTGTCACATGTCACGACAATTAGTGGGG-AACATTTATGTATA

TAGAAATTTCAGAAATTTCTAGCAGCGTGATAAATATAAAGATTTTGGCACAATAAGTTT

TTGGATCATAATGAGTTT-CTATTTAATCAA-GGCA-TGTAA-TCTATTTTATTATTTAG

-TAGGG-AAACTGAAAATTTAGGGTTGCTATTTGTAGCTCCCACCCTTCTTAGTTCTTAC

TCTTTTCAATATTTTTTAAAAGTTTTA-TATAATACCT-AAATT-GCCCTCCTCCT----

----------------------------------------CACCCCATTGCTCTTCCGTT

GCTCCTCCATGTG-AGTTCTTGTCTTTTTTTTT-CTCTGGTAGCGTTTTGCTGCTCCTTT

TT-CACTCAAGTGTTGCCAATTAA-TTGACAAAAAATGGTTTCTGTTTCATATAGAAACT

ATGTTTTTGTTGTGTAGTCATACATTACGGAATCTAGTTT-CCATTAAATAAGTAAC--G

TGAAAAAAAA--TAAAAGGTGAAATATATATTGTTGGAAAAGAAGCTATGAGGTGCAAGA

ACCGATCACATGGAGAAGGCAATGAAAGACAAGGAGGAGCAATGGAAGA-----------

---------GAGAAAATGAGAAGATGGAAGGGATGTGAAAATGTTTGAAAAAAACGAGGT

GATCAGTTTTAAAATACGAATTTAGTATTTTCTTTTTAAGAAAATTCTTTCG-AAAGTCG

TGTTTTAAAACATGACTTTTATT-ATTTGAAGTCG---TGTTCTAAAACATGACTTA--T

TCATATCCTT-AATATTTTT---------AAAATTTATCCATTTGTAATATTTTTTAAAA

ATTGACCCATATATGTAAAATACCCGTCAAGATCTCTTTATTATTTTGAAAGCGAAAGCA

TATCACTTCAAACACAATGGAATCGAGGCTATTGACTAAGTATAAATAGAGAAGACTTCA

TATCGGGGTTCATAACTCATAACAAAGCAAACGAGTATATAAGAAAGCATAAGCCAAATT

TTGAGTAAACTAGTGTGCACACTATCCCATGCCTAGTGGAAGTAGGGATCCTCTCGTTGT

TGGGGGAGTAATTGGGGATGTATTGGATCCTTTTGAATATTCTATTCCTATGAGGGTTAC

CTACAATAACAGAGATGTCAGCAATGGATGTGAATTCAAACCCTCACAAGTTGTCAACCA

ACCAAGGGTAAATATCGGTGGTGATGACC

>CS39.74

TATTAGACTCCTAATTTAATATTCTTGTTTTATTATAATGCTGATAAGTCTTGTAAATAA

GGGTGAGAAGCACGAATAATTAGTTCATGAGATGTGTATAATTATTATCCTACACGACTT

ATCTTTGATATTTCACACAAGTCTTTCAATGTATAACAAAAACTTTTTAGATACATTTAG

ACTATAGAACTAACAAGTTATATTTTGAACC-AAAAAACAAAGAGAGAGAGAGAGGAACA

TAGAGAAAAGAAGATATGAGAGTTTTTTTTTTTTTTTT---CTAAAACAGAAAGAAACTC

ATTATATAATAAACAAATTACTTTGAGACAAATTAACGTATGTAATAAAACAAAATCAAA

GTGGTAATTAAAATTATTTAATGGTAAACAGTCTAATAGTTAAAATAAAAATGGAAATCA

CATAAATTTTGTAATTGGCCCATTAAAAACAACACTAAGCTTTTAATTTGATTTTGAAAT

TCAAAATAATTTTATTAAATCACAAAGTAAAAGGTTTACAAAGCCGATCATGACAGTGCA

TGTGGGAGGCAAATCGGCATATTTGCACTACAAAAGG-ACCCATGAAGTCTCTGAACATG

CACGCAACACTTTAATCTCTTATTAGTTACTTTGAAAGCTTATTTATATATATAGACACG

CGTAAAAACTTCTTAACCAAGATTTTTTT-ACGTGCTTCC-TTCGCGTTTAATTTGGACC

ATCAAACCGTGCTCAACAGATAAAGAAAAGGGTGCTTTTGATTCAAGATATTGGCC-GAA

AAACACAAGATAGATCCTT-CGATAGATTAAGCCACGCATGAAACGCGAATCCAAAGTGA

TGAAGAAGTGCAGATAGATATTCGTTCACCATATAGAGGAGAATATATCATTCCTACAAA

CAAAATTGATGATGTTTCTAGAATAGAAACGTTAGAATCGAATATTCATCTGTTCCGGGT

GGGGAAGGGCTACTGAAAAGCTGAACTTTTTTAAGAGTTCGAACTTCGAAAGTCAATCAA

TGATTCTTAATTGAGAGGGAAAAGCTATAAAGGGCGAGAAGGGAAGGAAATGTTTATAGG

GAATGAATTTCTAATGCATGATGGGAATGACAGATATGAGGAATATATAAAGGTGATTCT

TATATACATTGACATGGATCGGAATCTATTAACTTAAAGTTATTGGGGTCGTGAAATTTA

TTTAATTTTTCTAATTCGTGTCACATGTCACGACAATTAGTGGGG-AACATTTATGTATA

TAGAAATTTCAGAAATTTCTAGCAGCGTGATAAATATAAAGATTTTGGCACAATAAGTTT

TTGGATCATAATGAGTTT-CTATTTAATCAA-GGCA-TGTAA-TCTATTTTATTATTTAG

-TAGGG-AAACTGAAAACTTAGGGTTGCTATTTGTAGCTCCCACCCTTCTTAGTTCTTAC

TCTTTTCAATATTTTTTAAAAGTTTTA-TATAATACCT-AAATT-GCCCTCCTCCT----

----------------------------------------CACCCCATTGCTCTTCCGTT

GCTCCTCCATGTG-AGTTCTTGTCTTTTTTTTT-CTCTGGTAGCGTTTTGCTGCTCCTTT

TTTCACTCAAGTGTTGCCAATTAA-TTGACAAAAAATGGTTTCTGTTTCATATAGAAACT

ATGTTTTTGTTGTGTAGTCATACATTACGGAATCTAGTTT-CCATTAAATAAGTAAC--G

TGAAAAAAAA--TAAAAGGTGAAATATATATTGTTGGAAAAGAAGCTATGAGGTGCAAGA

ACCGATCACATGGAGAAGGCAATGAAAGACAAGGAGGAGCAATGGAAGA-----------

---------GAGAAAATGAGAAGATGGAAGGGATGTGAAAATGTTTGAAAAAAACGAGGT

GATCAGTTTTAAAATACGAATTTAGTATTTTCTTTTTAAGAAAATTCTTTCG-AAAGTCG

TGTTTTAAAACATGACTTTTATT-ATTTGAAGTCG---TGTTCTAAAACATGACTTA--T

TCATATCCTT-AATATTTTT---------AAAATTTATCCATTTGTAATATTTTTTAAAA

ATTGACCCATATATGTAAAATACCCGTCAAGATCTCTTTATTATTTTGAAAGCGAAAGCA

TATCACTTCAAACACAATGGAATCGAGGCTATTGACTAAGTATAAATAGAGAAGACTTCA

TATCGGGGTTCATAATTCATAACAAAGCAAACGAGTATATAAGAAAGCATAAGCCAAATT

TTGAGTAAACTAGTGTGCACACTATCCCATGCCTAGTGGAAGTAGGGATCCTCTCGTTGT

TGGGGGAGTAATTGGGGATGTATTGGATCCTTTTGAATATTCTATTCCTATGAGGGTTAC

CTACAATAACAGAGATGTCAGCAATGGATGTGAATTCAAACCCTCACAAGTTGTCAACCA

ACCAAGGGTAAATATCGGTGGTGATGACC

>CS58.03

TATTAGACTCCTAATTTAATATTCTTGTTTTATTATAATGCTGATAAGTCTTGTAAATAA

GGGTGAGAAGCACGAATAATTAGTTCATGAGATGTGTATAATTATTATCCTACACGACTT

ATCTTTGATATTTCACACAAGTCTTTCAATGTATAACAAAAACTTTTTAGATACATTTAG

ACTATAGAACTAACAAGTTATATTTTGAACC-AAAAAACAAAGAGAGAGAGAGAGGAACA

TAGAGAAAAGAAGATATGAGAGTTTTTTTTTTTTTTTT---CTAAAACAGAAAGAAACTC

ATTATATAATAAACAAATTACTTTGAGACAAATTAACGTATGTAATAAAACAAAATCAAA

GTGGTAATTAAAATTATTTAATGGTAAACAGTCTAATAGTTAAAATAAAAATGGAAATCA

CATAAATTTTGTAATTGGCCCATTAAAAACAACACTAAGCTTTTAATTTGATTTTGAAAT

TCAAAATAATTTTATTAAATCACAAAGTAAAAGGTTTACAAAGCCGATCATGACAGTGCA

TGTGGGAGGCAAATCGGCATATTTGCACTACAAAAGG-ACCCATGAAGTCTCTGAACATG

CACGCAACACTTTAATCTCTTATTAGTTACTTTGAAAGCTTATTTATATATATAGACACG

CGTAAAAACTTCTTAACCAAGATTTTTTT-ACGTGCTTCC-TTCGCGTTTAATTTGGACC

ATCAAACCGTGCTCAACAGATAAAGAAAAGGGTGCTTTTGATTCAAGATATTGGCC-GAA

AAACACAAGATAGATCCTT-CGATAGATTAAGCCACGCATGAAACGCGAATCCAAAGTGA

TGAAGAAGTGCAGATAGATATTCGTTCACCATATAGAGGAGAATATATCATTCCTACAAA

CAAAATTGATGATGTTTCTAGAATAGAAACGTTAGAATCGAATATTCATCTGTTCCGGGT

GGGGAAGGGCTACTGAAAAGCTGAACTTTTTTAAGAGTTCGAACTTCGAAAGTCAACCAA

TGATTCTTAATTGAGAGGGAAAAGCTATAAAGGACGAGAAGGGAAGGAAATGTTTATAGG

GAATGAATTTCTAATGCATGATGGGAATGACAGATATGAGGAATATATAAAGGTGATTCT

TATATACATTGACATGGATCGGAATCTATTAACTTAAAGTTATTGGGGTCGTGAAATTTA

TTTAATTTTTCTAATTCGTGTCACATGTCACGACAATTAGTGGGG-AACATTTATGTATA

TAGAAATTTCAGAAATTTCTAGCAGCGCGATAAATATAAAGATTTTGGCACAATAAGTTT

TTGGATCATAATGAGTTT-CTATTTAATCAA-GGCA-TGTAA-TCTATTTTATTATTTAG

-TAGGG-AAACTGAAAACTTAGGGTTGCTATTTGTAGCTCCCACCCTTCTTAGTTCTTAC

TCTTTTCAATATTTTTTAAAAGTTTTA-TATAATACCT-AAATT-GCCCTCCTCCT----

----------------------------------------CACCCCATTGCTCTTCCGTT

GCTCCTCCATGTG-AGTTCTTGTCTTTTTTTT--CTCTGGTAGCGTTTTGCTGCTCCTTT

TTTCACTCAAGTGTTGCCAATTAA-TTGACAAAAAATGGTTTCTGTTTCATATAGAAACT

ATGTTTTTGTTGTGTAGTCATACATTACGGAATCTAGTTT-CCATTAAATAAGTAAC--G

TGAAAAAAAA--TAAAAGGTGAAATATATATTGTTGGAAAAGAAGCTATGAGGTGCAAGA

ACCGATCACATGGAGAAGGCAATGAAAGACAAGGGGGAGCAATGGAAGA-----------

---------GAGAAAATGAGAAGATGGAAGGGATGTGAAAATGTTTGAAAAAAACGAGGT

GATCAGTTTTAAAATACGAATTTAGTATTTTCTTTTTAAGAAAATTCTTTCG-AAAGTCG

TGTTTTAAAACATGACTTTTATT-ATTTGAAGTCG---TGTTCTAAAACATGACTTA--T

TCATATCCTT-AATATTTTT---------AAAATTTATCCATTTGTAATATTTTTTAAAA

ATTGACCCATATATGTAAAATACCCGTCAAGATCTCTTTATTATTTTGAAAGCGAAAGCA

TATCACTTCAAACACAATGGAATCGAGGCTATTGACTAAGTATAAATAGAGAAGACTTCA

TATCGGGGTTCATAATTCATAACAAAGCAAACGAGTATATAAGAAAGCATAAGCCAAATT

TTGAGTAAACTAGTGTGCACACTATCCCATGCCTAGTGGAAGTAGGGATCCTCTCGTTGT

TGGGGGAGTAATTGGGGATGTATTGGATCCTTTTGAATATTCTATTCCTATGAGGGTTAC

CTACAATAACAGAGATGTCAGCAATGGATGTGAATTCAAACCCTCACAAGTTGTCAACCA

ACCAAGGGTAAATATCGGTGGTGATGACC

>CS58.06

TATTAGACTCCTAATTTAATATTCTTGTTTTATTATAATGCTGATAAGTCTTGTAAATAA

GGGTGAGAAGCACGAATAATTAGTTCATGAGATGTGTATAATTATTATCCTACACGACTT

ATCTTTGATATTTCACACAAGTCTTTCAATGTATAACAAAAACTTTTTAGATACATTTAG

ACTATAGAACTAACAAGTTATATTTTGAACC-AAAAAACAAAGAGAGAGAGAGAGGAACA

TAGAGAAAAGAAGATATGAGAGTTTTTCTTTTTTTTTT---CTAAAACAGAAAGAAACTC

ATTATATAATAAACAAATTACTTTGAGACAAATTAACGTATGTAATAAAACAAAATCAAA

GTGGTAATTAAAATTATTTAATGGTAAACAGTCTAATAGTTAAAATAAAAATGGAAATCA

CATAAATTTTGTAATTGGCCCATTAAAAACAACACTAAGCTTTTAATTTGATTTCGAAAT

TCAAAATAATTTTATTAAATCACAAAGTAAAAGGTTTACAAAGCCGATCATGACAGTGCA

TGTGGGAGGCAAATCGGCATATTTGCACTACAAAAGG-ACCCATGAAGTCTCTGAACATG

CACGCAACACTTTAATCTCTTATTAGTTACTTTGAAAGCTTATTTATATATATAGACACG

CGTAAAAACTTCTTAACCAAGATTTTTTT-ACGTGCTTCC-TTCGCGTTTAATTTGGACC

ATCAAACCGTGCTCAACAGATAAAGAAAAGGGTGCTTTTGATTCAAGATATTGGCC-GAA

AAACACAAGATAGATCCTT-CGATAGATTAAGCCACGCATGAAACGCGAATCCAAAGTGA

TGAAGAAGTGCAGATAGATATTCGTTCACCATATAGAGGAGAATATATCATTCCTACAAA

CAAAATTGATGATGTTTCTAGAATAGAAACGTTAGAATCGAATATTCATCTGTTCCGGGT

GGGGAAGGGCTACTGAAAAGCTGAACTTTTTTAAGAGTTCGAACTTCGAAAGTCAATCAA

TGATTCTTAATTGAGAGGGAAAAGCTATAAAGGACGAGAAGGGAAGGAAATGTTTATAGG

GAATGAATTTCTAATGCATGATGGGAATGACAGATATGAGGAATATATAAAGGTGATTCT

TATATACATTGACATGGATCGGAATCTATTAACTTAAAGTTATTGGGGTCGTGAAATTTA

TTTAATTTTTCTAATTCGTGTCACATGTCACGACAATTAGTGGGG-AACATTTATGTATA

TAGAAATTTCAGAAATTTCTAGCAGCGTGATAAATATAAAGATTTTGGCACAATAAGTTT

TTGGATCATAATGAGTTT-CTATTTAATCAA-GGCA-TGTAA-TCTATTTTATTATTTAG

-TAGGG-AAACTGAAAACTTAGGGTTGCTATTTGTAGCTCCCACCCTTCTTAGTTCTTAC

TCTTTTCAATATTTTTTAAAAGTTTTA-TATAATACCT-AAATT-GCCCTCCTCCT----

----------------------------------------CACCCCATTGCTCTTCCGTT

GCTCCTCCATGTG-AGTTCTTGTCTTTTTTTTT-CTCTGGTAGCGTTTTGCTGCTCCTTT

TTTCACTCAAGTGTTGCCAATTAA-TTGACAAAAAATGGTTTCTGTTTCATATAGAAACT

ATGTTTTTGTTGTGTAGTCCTACATTACGGAATCTAGTTT-CCATTAAATAAGTAAC--G

TGAAAAAAAA--TAAAAGGTGAAATATATATTGTTGGAAAGGAAGCTATGAGGTGCAAGA

ACCGATCACATGGAGAAGGCAATGAAAGACAAGGAGGAGCAGTGGAAGA-----------

---------GAGAAAATGAGAAGATGGAAGGGATGTGAAAATGTTTGAAAAAAACGAGGT

GATCAGTTTTAAAATACGAATTTAGTATTTTCTTTTTAAGAAAATTCTTTCG-AAAGTCG

TGTTTTAAAACATGACTTTTATT-ATTTGAAGTCG---TGTTCTAAAACATGACTTA--T

TCATATCCTT-AATATTTTT---------AAAATTTATCCATTTGTAATATTTTTTAAAA

ATTGACCCATATATGTAAAATACCCGTCAAGATCTCTTTATTATTTTGAAAGCGAAAGCA

TATCACTTCAAACACAATGGAATCGAGGCTATTGACTAAGTATAAATAGAGAAGACTTCA

TATCGGGGTTCATAATTCATAACAAAGCAAACGAGTATATAAGAAAGCATAAGCCAAATT

TTGAGTAAACTAGTGTGCACACTATCCCATGCCTAGTGGAAGTAGGGATCCTCTCGTTGT

TGGGGGAGTAATTGGGGATGTATTGGATCCTTTTGAATATTCTATTCCCATGAGGGTTAC

CTACAATAACAGAGATGTCAGCAATGGATGTGAATTCAAACCCTCACAAGTTGTCAACCA

ACCAAGGGTAAATATCGGTGGTGATGACC

>CS05.46

TATTAGACTCCTAATTTAATATTCTTGTTTTATTATAATGCTGATAAGTCTTGTAAATAA

GGGTGAGAAGCACGAATAATTAGTTCATGAGATGTGTATAATTATTATCCTACACGACTT

ATCTTTGATATTTCACACAAGTCTTTCAATGTATAACAAAAACTTTTTAGATACATTTAG

ACTATAGAACTAACAAGGTATATTTTGAACC-AAAAAACAAAGAGAGAGAGAGAGGAACA

TAGAGAAAAGAAGATATGAGAGTTTTTTTTTTTTTTTT---CTAAAACAGAAAGAAACTC

ATTATATAATAAACAAATTACTTTGAGACAAATTAACGTATGTAATAAAACAAAATCAAA

GTGGTAATTAAAATTATTTAATGGTAAACAGTCTAATAGTTAAAATAAAGATGGAAATCA

CATAAATTTTGTAATTGGCCCATTAAAAACAACACTAAGCTTTTAATTTGATTTTGAAAT

TCAAAATAATTTTATTAAATCACAAAGTAAAAGGTTTACAAAGCCGATCATGACAGTGCA

TGTGGGAGGCAAATCGGCATATTTGCACTACAAAAGG-ACCCATGAAGTCTCTGAACATG

CACGCAACGCTTTAATCTCTTATTAGTTACTTTGAAAGCTTATTTATATATATAGACACG

CGTAAAAACTTCTTAACCAAGATTTTTTT-ACGTGCTTCC-TTCGCGTTTAATTTGGACC

ATCAAACCGTGCTCAACAGATAAAGAAAAGGGTGCTTTTGATTCAAGATATTGGCC-GAA

AAACACAAGATAGATCCTT-CGATAGATTAAGCCACGCATGAAACGCGAATCCAAAGTGA

TGAAGAAGTGCAGATAGATATTCGTTCACCATATAGAGGAGAATATATCATTCCTACAAA

CAAAATTGATGATGTTTCTAGAATAGAAACGTTAGAATCGAATATTCATCTGTTCCGGGT

GGGGAAGGGCTACTGAAAAGCTGAACTTTTTTAAGAGTTCGAACTTCGAAAGTCAATCAA

TGATTCTTAATTGAGAGGGAAAAGCTATAAAGGACGAGAAGGGAAGGAAATGTTTATAGG

GAATGAATTTCTAATGCATGATGGGAATGACAGATATGAGGAATATATAAAGGTGATTCT

TATATACATTGACATGGATCGGAATCTATTAACTTAAAGTTATTGGGGTCGTGAAATTTA

TTTAATTTTTCTAATTCGTGTCACATGTCACGACAATTAGTGGGG-AACATTTATGTATA

TAGAAATTTCAGAAATTTCTAGCAGCGTGATAAATATAAAGATTTTGGCACAATAAGTTT
[truncated: 24,451 more chars]
